# The role of PCNA as a scaffold protein in cellular signaling is functionally conserved between yeast and humans

**DOI:** 10.1002/2211-5463.12442

**Published:** 2018-05-31

**Authors:** Camilla Olaisen, Hans Fredrik N. Kvitvang, Sungmin Lee, Eivind Almaas, Per Bruheim, Finn Drabløs, Marit Otterlei

**Affiliations:** ^1^ Department of Clinical and Molecular Medicine Faculty of Medicine and Health Sciences Norwegian University of Science and Technology (NTNU) Trondheim Norway; ^2^ Department of Biotechnology and Food Science Faculty of Natural Sciences Norwegian University of Science and Technology (NTNU) Trondheim Norway

**Keywords:** conservation, DNA damage response, hypersensitivity, MAPK, PCNA, phosphatidylinositol, signaling

## Abstract

Proliferating cell nuclear antigen (PCNA), a member of the highly conserved DNA sliding clamp family, is an essential protein for cellular processes including DNA replication and repair. A large number of proteins from higher eukaryotes contain one of two PCNA‐interacting motifs: PCNA‐interacting protein box (PIP box) and AlkB homologue 2 PCNA‐interacting motif (APIM). APIM has been shown to be especially important during cellular stress. PIP box is known to be functionally conserved in yeast, and here, we show that this is also the case for APIM. Several of the 84 APIM‐containing yeast proteins are associated with cellular signaling as hub proteins, which are able to interact with a large number of other proteins. Cellular signaling is highly conserved throughout evolution, and we recently suggested a novel role for PCNA as a scaffold protein in cellular signaling in human cells. A cell‐penetrating peptide containing the APIM sequence increases the sensitivity toward the chemotherapeutic agent cisplatin in both yeast and human cells, and both yeast and human cells become hypersensitive when the Hog1/p38 MAPK pathway is blocked. These results suggest that the interactions between APIM‐containing signaling proteins and PCNA during the DNA damage response is evolutionary conserved between yeast and mammals and that PCNA has a role in cellular signaling also in yeast.

**Abbreviations**
APIM: AlkB homologue 2 PCNA‐interacting motif
MAPK: mitogen‐activated protein kinase
PCNA: proliferating cell nuclear antigen
PI: phosphatidylinositol
PIN: protein interaction network
PIP box: PCNA‐interacting protein box
PPI: protein–protein interaction

Proliferating cell nuclear antigen (PCNA) belongs to the conserved DNA sliding clamp family essential for DNA replication and associated processes and has a ring‐like protein structure composed of three monomers assembled in a head‐to‐tail manner 1, 2. More than 600 human proteins contain the PCNA‐binding sequences PIP box or APIM, several of which are mainly localized in the cytosol 3 (Table [Link], [Link]A,B). In accordance with these observations, a role of PCNA as a scaffold protein in cellular signaling independent of chromatin has emerged in recent years: i) Cytosolic PCNA regulates neutrophil survival by binding to procaspases, and thereby inhibiting their activation and preventing apoptosis 4. In neuroblastoma cells, nitric oxide stress led to S‐nitrosylation of PCNA and a decrease in the interaction with caspase‐9 5. In support of a role for PCNA in the regulation of apoptosis, we showed that the treatment of human multiple myeloma cells with a cell‐penetrating APIM‐containing peptide (APIM‐peptide) resulted in caspase‐dependent apoptosis independent of cell cycle phase 6. ii) PCNA on the surface of cancer cells can inhibit natural killer cell cytotoxic function, and this is suggested to be a mechanism for cancer cells to evade antitumor immunity 7, 8. iii) Putative PCNA‐binding proteins involved in regulation of metabolism have been identified by proteomic approaches 9, 10. Furthermore, iv) proteins involved in signal transduction are identified in PCNA complexes 11. Many of these, for example, ERK2, MST4, NF1, FAK1, GSK3β, and PAK1, have a role in mitogen‐activated protein kinase (MAPK) signaling. Moreover, treatment of human monocytes with an APIM‐peptide that blocks the interaction between APIM‐containing proteins and PCNA reduced phosphorylation of AKT and the secretion of several cytokines after stimulation of Toll‐like receptors. Inhibition of p38 MAPK enhanced this effect of the APIM‐peptide 11.

Many proteins from the budding yeast *Saccharomyces cerevisiae* share more than 40% conserved sequence with at least one known or predicted human protein. Many key proteins of the DNA damage response cascade, such as the yeast orthologs of the human ATM and ATR proteins, Tel1 and Mec1 12, 13, the yeast MAPK Fus3 and Kss1 14, and many signaling pathways, including the three‐tiered MAPK module, are highly conserved between yeast and human. This has made yeast a widely used model organism for studies of cellular signaling.

Both human and yeast proteins can interact with PCNA via their conserved PIP box 1. APIM is conserved in mammals, and an increasing amount of evidence has established a functional role of this motif in enabling protein–PCNA interactions during cellular stress 3, 6, 11, 15, 16, 17, 18. Here, we show that APIM is conserved in yeast and is present in important signaling proteins involved in phosphatidylinositol (PI) and MAPK signaling similarly to what is observed in human cells. Because both PI and MAPK signaling are important in cellular stress response and are highly conserved throughout eukaryotic evolution 19, we compared the cisplatin sensitivity of yeast MAPK deletion mutants and MAPK inhibitor‐treated human cells in the presence of the APIM‐peptide. Both yeast and human cells treated with the APIM‐peptide in combination with *Hog1* deletion or p38 inhibition, respectively, becomes hypersensitive to cisplatin, suggesting that the role of APIM–PCNA interactions in cellular signaling is functionally conserved.

## Results and Discussion

### APIM is conserved in yeast

Functional features are often conserved in orthologs, for example, sequence motifs important for protein–protein interactions. To find a motif to be conserved across a set of proteins is therefore a strong indication that the motif is both functional and important. Our search for conserved APIM sequences ([KR][FYW](([LVI][ALVI])|([ALVI][LVI]))[KR]) in yeast identified 84 proteins (Table 1, Table S1C), indicating that the APIM is conserved in yeast. In comparison, mammals have 378 proteins were APIM is conserved in at least three species (Table S1B).

**Table 1:** APIM‐containing yeast proteins and their functional categories (FunCat ID). FunCat IDs: 1: metabolism, 2: energy, 10: cell cycle and DNA processing, 11: transcription, 12: protein synthesis, 14: protein fate (folding, modification, destination), 16: protein with binding function or cofactor requirement (structural or catalytic), 18: regulation of metabolism and protein function, 20: cellular transport, transport facilitation, and transport routes, 30: cellular communication/signal transduction mechanism, 32: cell rescue, defense, and virulence, 34: Interaction with the environment, 40: cell fate, 41: development (systemic), 42: biogenesis of cellular components, 43: cell‐type differentiation

Gene ID	Gene name	Protein name	Associated FunCat IDs^a^
YJL187C	*Swe1*	Mitosis inhibitor protein kinase Swe1	1, 10, 14, 18, 40, 42, 43
YGL163C	*Rad54*	DNA repair and recombination protein Rad54	10, 16, 32, 34, 41, 42
YMR109W	*Myo5*	Myosin‐5	2, 20, 32, 34, 42, 43
YBR073W	*Rdh54*	DNA repair and recombination protein Rdh54	10, 16, 32, 34, 41
YDR457W	*Tom1*	E3 ubiquitin‐protein ligase Tom1	10, 14, 16, 20, 42
YFR019W	*Fab1*	1‐phosphatidylinositol 3‐phosphate 5‐kinase	1, 14, 20, 32, 42
YLR106C	*Mdn1*	Midasin	1, 11, 12, 14, 16
YPL106C	*Sse1*	Heat shock protein Sse1	14, 16, 32, 34, 40
YAL026C	*Drs2*	Probable phospholipid‐transporting ATPase	1, 11, 16, 20
YBR038W	*Chs2*	Chitin synthase 2	1, 10, 32, 43
YBR245C	*Isw1*	Isw1 chromatin‐remodeling complex ATPase Isw1	1, 10, 11, 16
YDR208W	*Mss4*	Probable phosphatidylinositol 4‐phosphate 5‐kinase Mss4	1, 30, 42, 43
YHR099W	*Tra1*	Transcription‐associated protein 1	10, 11, 14, 42
YKL112W	*Abf1*	ARS‐binding factor 1	1, 10, 11, 16
YOR259C	*Rpt4*	26S proteasome subunit Rpt4	1, 11, 14, 16
YGL099W	*Lsg1*	Large subunit GTPase 1	12, 41, 43
YHL030W	*Ecm29*	Proteasome component Ecm9	14, 16, 42
YLR045C	*Stu2*	Protein Stu2	10, 16, 42
YLR382C	*Nam2*	Leucine‐tRNA ligase	11, 12, 16
YOL008W	*Coq10*	Coenzyme Q‐binding protein Coq10	2, 14, 16
YPR119W	*Clb2*	G2/mitotic‐specific cyclin‐2	10, 18, 43
YBL004W	*Utp20*	U3 small nucleolar RNA‐associated protein 20	11, 16
YBL037W	*Apl3*	AP‐2 complex subunit alpha	14, 20
YBR235W	*Vhc1*	Vacuolar cation‐chloride cotransporter 1	20, 34
YCR033W	*Snt1*	Probable DNA‐binding protein Snt1	10, 14
YDL140C	*Rpo21*	DNA‐directed RNA polymerase II subunit Rpb1	11, 16
YDR421W	*Aro80*	Transcriptional activator Aro80	1, 11
YDR489W	*Sld5*	DNA replication complex GINS protein Sld5	10, 16
YFL049W	*Swp82*	SWI/SNF global transcription activator complex subunit Swp82	10, 11
YGL084C	*Gup1*	Glycerol uptake protein 1	1, 20
YJL109C	*Utp10*	U3 small nucleolar RNA‐associated protein 10	11, 16
YKL176C	*Lst4*	Protein Lst4	14, 20
YLL040C	*Vps13*	Vacuolar protein sorting‐associated protein 13	14, 20
YLR256W	*Hap1*	Transposon Ty1‐LR4 Gag‐Pol polyprotein	2, 11
YML098W	*Taf13*	Transcription initiation factor TFIID subunit 13	10, 11
YML127W	*Rsc9*	Chromatin structure‐remodeling complex subunit Rsc9	10, 11
YNL248C	*Rpa49*	DNA‐directed RNA polymerase I subunit Rpa49	11, 16
YNR019W	*Are2*	Sterol O‐acyltransferase 2	1, 43
YOL129W	*Vps68*	Vacuolar protein sorting‐associated protein 68	14, 20
YOR126C	*Iah1*	Isoamyl acetate‐hydrolyzing esterase	1, 2
YOR176W	*Hem15*	Ferrochelatase	1, 34
YOR255W	*Osw1*	Outer spore wall protein 1	42, 43
YPL125W	*Kap120*	Importin beta‐like protein Kap120	14, 20
YPR018W	*Rlf2*	Chromatin assembly factor 1 subunit p90	10, 14
YPR166C	*Mrp2*	37S ribosomal protein MRP2	12, 42
YGR240C	*Pfk1*	ATP‐dependent 6‐phosphofructokinase subunit alpha	1, 2
YBR118W	*Tef2*	Elongation factor 1‐alpha	12
YBR203W	*Cos111*	F‐box protein Cos111	30
YDL164C	*Cdc9*	DNA ligase 1	10
YDL191W	*Rpl35A*	60S ribosomal protein L35‐A	12
YDR125C	*Ecm18*	Extracellular mutant protein 18	42
YDR502C	*Sam2*	S‐adenosylmethionine synthase 2	1
YDL136W	*Rpl35B*	60S ribosomal protein L35‐B	12
YGL137W	*Sec27*	Coatomer subunit beta	20
YGR124W	*Asn2*	Asparagine synthetase	1
YHR116W	*Cox23*	Cytochrome c oxidase‐assembly factor Cox23	2
YHR137W	*Aro9*	Aromatic amino acid aminotransferase 2	1
YJL012C	*Vtc4*	Vacuolar transporter chaperone 4	42
YJL090C	*Dpb11*	DNA replication regulator Dpb11	10
YKL028W	*Tfa1*	Transcription initiation factor IIE subunit alpha	11
YKL103C	*Ape1*	Vacuolar aminopeptidase 1	14
YKR026C	*Gcn3*	Translation initiation factor eIF‐2B subunit alpha	12
YLR089C	*Alt1*	Probable alanine aminotransferas	1
YLR180W	*Sam1*	S‐adenosylmethionine synthase 1	1
YMR162C	*Dnf3*	Probable phospholipid‐transporting ATPase Dnf3	20
YMR176W	*Ecm5*	Extracellular matrix protein 5	42
YOL049W	*Gsh2*	Glutathione synthetase	1
YOR260W	*Gcd1*	Translation initiation factor eIF‐2B subunit gamma	12
YPR031W	*Nto1*	NuA3 HAT complex component Nto1	11
YPR080W	*Tef1*	Elongation factor 1‐alpha	12
YPR105C	*Cog4*	Conserved oligomeric Golgi complex subunit 4	20
YPR145W	*Asn1*	Glutamine‐dependent asparagine synthetase 1	1
YBR108W	*Aim3*	Altered inheritance of mitochondria protein 3	–
YDL169C	*Ugx2*	Protein Ugx2	–
YDR051C	*Det1*	Broad‐range acid phosphatase DET1	–
YER077C	*Mrx1*	Mitochondrial organization of gene expression protein 1	–
YGL131C	*Snt2*	E3 ubiquitin‐protein ligase SNT2	–
YHL029C	*Oca5*	Oxidant‐induced cell cycle arrest protein 5	–
YHR059W	*Fyv4*	Function required for yeast viability protein 4	–
YJL107C	*Yjl107C*	Uncharacterized UPF0442 protein Yjl107C	–
YNL080C	*Eos1*	ER‐localized and oxidants sensitive protein 1	–
YNL193W	*Ynl193W*	Uncharacterized protein Ynl193W	–
YOR112W	*Cex1*	Cytoplasmic export protein 1	–
YPL137C	*Gip3*	GLC7‐interacting protein 3	–

a Number of APIM‐containing proteins in different FunCat IDs and *P*‐values are given in Table S2.

Enrichment analysis with DAVID 20, 21 of the 84 APIM‐containing yeast proteins identified clusters enriched for functional annotations associated with processes such as nucleotide binding, ligase activity, and transcription (Table S2). Some proteins were found in multiple clusters, indicating that they are hub proteins, which means that they can be involved in several processes. Examples of such hub proteins are proteins associated with kinase activities (Fab1, Mss4, Pfk1, Tra1, Swe1), ligase activities (Cdc9, Snt2, Tom1, Gsh2), and DNA repair (Cdc9, Rad54, Rdh54, Tra1).

We also analyzed the 84 APIM‐containing yeast proteins using a gene‐set approach based on protein–protein interactions (PPIs), where PPI clusters were tested for enrichment with respect to APIM‐containing proteins and FunCat functional categories (FunCat IDs) 22 (Table 1 and Table S3). Our analysis showed that PPI clusters significantly enriched for proteins with APIM (*P*‐values from 0.007 to 0.03) also were significantly enriched for specific FunCat IDs, in particular ‘protein synthesis’(ID:12), ‘protein fate—folding, modification, destination’ (ID:14), and ‘protein with binding function or cofactor requirement’ (ID:16) (Table S4). The PPI network was tested for network properties such as degree and node distances for APIM‐containing proteins compared to general proteins, using randomization, but no significant differences could be found (data not shown).

### A potential role for PCNA in cellular signaling in yeast

**Figure 1:**
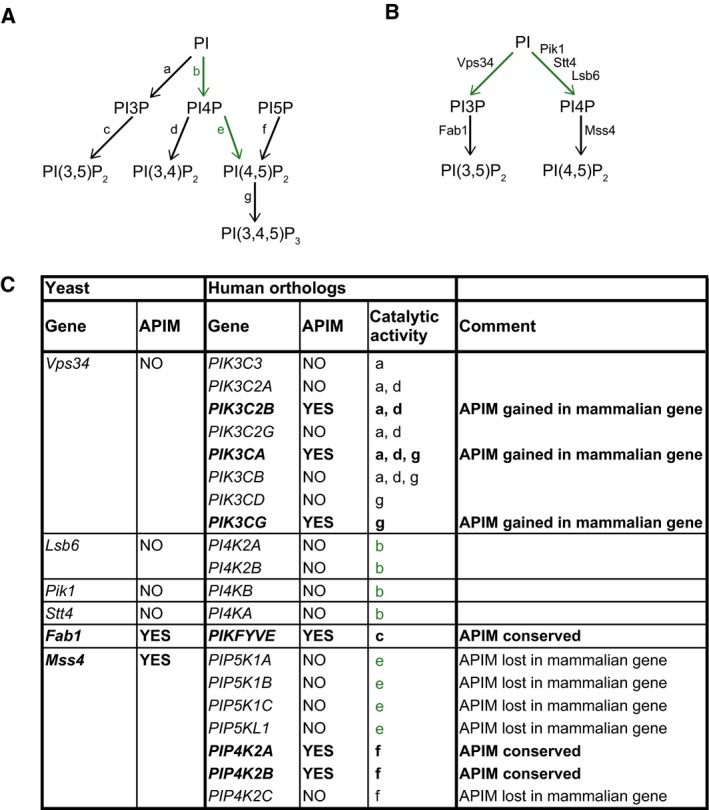
Several human and yeast PI kinases have a conserved APIM sequence. Pathways generating PIs in human (A) and yeast (B) by PI kinases. Black arrows refer to activity that can be performed by APIM‐containing PI kinases, while green arrows and letters refer to activity performed only by non‐APIM‐containing PI kinases. The activity of PI phosphatases is not shown. (C) Conservation of APIM in yeast and human PI kinases. The letters (a–g) in the column for catalytic activity in (C) refer to the arrows in (A).

Nineteen of the 84 yeast APIM‐containing proteins have human orthologs that also contain APIM (Table 2). The conservation of APIM in Fab1 and Mss4 suggests a role for PCNA in signaling also in yeast. Fab1 and Mss4, human PIKFYVE and PIP4K2A/B, respectively, are lipid kinases that phosphorylate PIs on cellular membranes 23, 24. PIs are membrane phospholipids that are important for actin cytoskeleton remodeling, cellular stress response signaling, vesicle trafficking, and protein recruitment to cellular membranes in both yeast and mammals 25, 26. The different PI species present in human and yeast cells are shown in Fig. 1A,B, respectively 23, 24, 27. In addition to the PI kinases mentioned above, the human genome has three APIM‐containing PI kinases (PIK3C2B, PIK3CA, and PIK3CG) (Fig. 1A and C), and two human PI3 lipid phosphatases (myotubularin and myotubularin‐related protein 1, not included in Fig. 1) (http://tare.medisin.ntnu.no/pcna/index.php). Therefore, the regulation of the various PI species strongly depends on APIM‐containing proteins in both humans and in yeast.

**Table 2:** Genes with conserved APIM in both human and yeast

Yeast gene	Human gene	Human protein	Protein function
*Tef2*	*EEF1A1*	Elongation factor 1‐alpha 1	Transcription and translation factor
	*EEF1A2*	Elongation factor 1‐alpha 2	Translation factor
*Rpo21*	*POLR2A*	DNA‐directed RNA polymerase II subunit RPB1	Transcription
*Rpl35a*	*RPL35*	60S ribosomal protein L35	Component of ribosome
*Rpl35b*			
*Tfa1*	*GTF2E1*	General transcription factor IIE subunit 1	Transcription factor
*Cdc9*	*LIG1*	DNA ligase 1	DNA replication and repair
*Tra1*	*TRRAP*	Transformation/transcription domain‐associated protein	Chromatin modification
*Mss4*	*PIP4K2A*	Phosphatidylinositol 5‐phosphate 4‐kinase type‐2 alpha	Lipid kinase
	*PIP4K2B*	Phosphatidylinositol 5‐phosphate 4‐kinase type‐2 beta	Lipid kinase
*Fab1*	*PIKFYVE*	1‐phosphatidylinositol 3‐phosphate 5‐kinase	Lipid kinase
*Lsg1*	*LSG1*	Large subunit GTPase 1 homolog	Nuclear export
*Sec27*	*COPB2*	Coatomer subunit beta	Protein/vesicle transport
*Vhc1*	*SLC12A8*	Solute carrier family 12 member 8	Cation/chloride cotransporter
*Ecm18*	*ABHD4*	Protein ABHD4	Lysophospholipase
*Ecm29*	*ECM29*	Proteasome‐associated protein ECM29 homolog	Component of proteasome
*Rpt4*	*PSMC6*	26S protease regulatory subunit 10B	Degradation of ubiquitinated proteins
*Mdn1*	*MDN1*	Midasin	Nuclear chaperone, nuclear export
*Are2*	*DGAT1*	Diacylglycerol O‐acyltransferase 1	Triacylglycerol synthesis
*Gsh2*	*GSS*	Glutathione synthetase	Glutathione synthesis
*Sam1*	*MAT1A*	S‐adenosylmethionine synthase isoform type‐1	S‐adenosylmethionine synthesis
	*MAT2A*	S‐adenosylmethionine synthase isoform type‐2	S‐adenosylmethionine synthesis

Phosphatidylinositol kinases are closely connected to PI3K/AKT/mTOR and MAPK signaling because the assembly and spatiotemporal organization of multiprotein complexes involved in these pathways depend on direct interaction with PIs 19, 23, 24. For example, a cancer‐causing mutation in the PI‐binding domain of AKT results in enhanced binding affinity for PI(4,5)P_2_ and leads to constitutive activation of AKT 28, 29. In yeast, Sho1, a transmembrane protein and adaptor for the filamentous growth pathway is mislocalized in a Mss4 mutant, leading to a decreased activation of the MAPK signaling and deregulation of filamentous growth 30. Furthermore, the APIM‐containing human eukaryotic translation elongation factors 1α (eEF1A1 and eEF1A2) are described as putative oncogenes with a role in regulating PI signaling 31. The yeast ortholog of eEF1A1/2 (Tef2) also contains APIM (Table 2). Taken together, several APIM‐containing proteins in yeast are involved in PI, MAPK, and downstream signaling similarly to what is observed in human cells.

Further supporting a role for PCNA in cellular signaling is the finding of PCNA double trimers in both human and yeast cells. Because PCNA is loaded on DNA in an orientation‐dependent manner, the double trimer cannot be loaded onto DNA and has therefore been proposed to have a role in cellular signaling in cytoplasm 32, 33, 34.

### Inactivation of Hog1/p38 in combination with the APIM‐peptide makes cells hypersensitive to DNA damage‐induced stress

**Figure 2:**
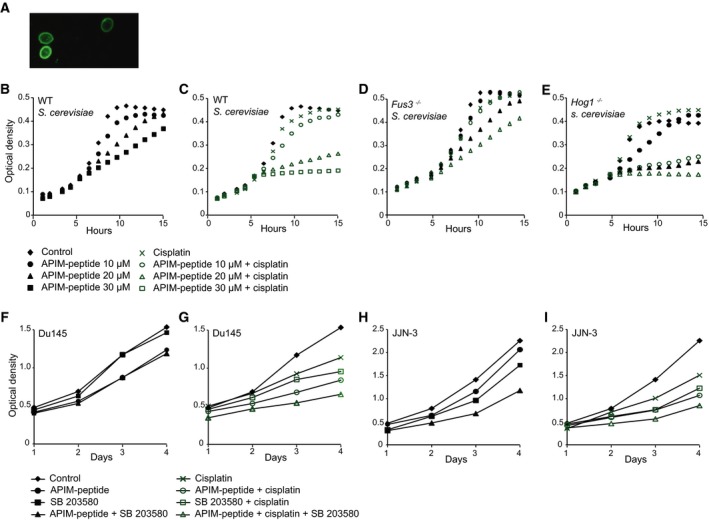
The APIM‐peptide is imported into yeast and affects cellular growth. (A) Confocal image of live *S. cerevisiae* 2–5 minutes after addition of FAM‐labeled APIM‐peptide. (B and C) WT *S. cerevisiae* treated with different concentrations of APIM‐peptide alone (B) and in combination with 125 μm cisplatin (C). (D) *Fus3* ^*−/−*^ and (E) *Hog1* ^*−/−*^ *S. cerevisiae* treated with 125 μm cisplatin in combination with APIM‐peptide. Optical densities were measured every hour for 24 h (15 h are shown in the plot). Concentrations and symbol explanations are indicated in the panel below. Data shown as mean from *n* technical replicates: controls (no treatment; *n* = 9), APIM‐peptide (*n* = 3), cisplatin (*n* = 7), and combinatorial treatment (*n* = 2). Two independent biological replicates were analyzed for the *WT* and *Hog1* ^*−/−*^ strains giving identical results. (F‐I) Cell growth (MTT assay) of Du145 cells (F and G) and JJN‐3 cells (H and I) treated with APIM‐peptide (6 μm), p38 inhibitor SB 203580 (10 μm) (F and H), cisplatin (0.6 μm for Du145; 0.4 μm for JJN‐3), and combinations of these three agents (G and I). Symbols are explained in the panel below. Data shown as mean from at least three parallel wells from one representative experiment of three independent experiments.

Having established that APIM is conserved in yeast, we next explored the effects of cell‐penetrating APIM‐containing peptides on yeast cells during cellular stress. A fluorescently tagged APIM‐peptide 6 was rapidly imported in yeast cells (*S. cerevisiae*), and the cells were sensitive to the APIM‐peptide treatment in a dose–response manner (Fig. 2A,B). Moreover, the APIM‐peptide sensitized the cells to the chemotherapeutic agent cisplatin (Fig. 2C), similar to what we have previously observed with several different chemotherapeutics in multiple human cancer cell lines and preclinical animal models 6, 35. This effect of the APIM‐peptide is dependent upon binding of the peptide to PCNA, because a mutated APIM‐peptide with lower binding capacity for PCNA does not increase cisplatin sensitivity 6. In human cells, added APIM‐peptide is localized intracellularly, whereas in yeast cells, the peptide is also found in the cell membrane. Thus, we cannot exclude a membrane effect of the peptide at this point.

As discussed above, PI and MAPK signaling pathways are closely connected, and many APIM‐containing proteins are participating in these pathways in both yeast and mammalian cells. In addition to the PI kinases, several members of the human MAPK pathways contain APIM. Examples are the MEK‐ERK (MST4, SOS1/2, ERK8), JNK (TAO2), and p38 (MK2 and MK5) pathways 3. Both p38, JNK, and the MEK‐ERK pathway are linked to cellular stress response to the chemotherapeutic agent cisplatin 36, 37, and both p38 and its yeast ortholog Hog1, as well as several other MAPKs, are activated upon oxidative stress 36, 38, 39, 40. Thus, to examine the sensitivity toward cisplatin and APIM‐peptide in the absence of MAPK signaling in yeast, we used the MAPK knockout strains *Hog1* ^*−/−*^ *, Fus3* ^*−/−*^ *, Kss1* ^*−/−*^ *, Smk1* ^*−/−*^ *,* and *Mpk1* ^*−/−*^, in addition to the pseudokinase *Mlp1* ^*−/−*^. *Mpk1* ^*−/−*^ cells have previously been demonstrated to be hypersensitive to genotoxic stress 41; however, in our hands, this strain had a very low growth rate, also in the absence of cisplatin, and it was therefore excluded from further screening (data not shown). The sensitivity toward cisplatin, APIM‐peptide, or the combination of APIM‐peptide and cisplatin was similar or lower compared to the WT strain for the *Fus3* ^*−/−*^ (Fig. 2D), *Kss1* ^*−/−*^ *, Smk1* ^*−/−*^ *,* and *Mlp1* ^*−/−*^ strains (Fig. S1). The *Hog1* ^*−/−*^ strain was equally sensitive as WT toward cisplatin or to a low dose of APIM‐peptide (10 μm). However, this strain was clearly much more sensitive toward cisplatin in combination with APIM‐peptide, and to higher concentrations of the APIM‐peptide (Fig. 2E). Because this effect is only seen in the *Hog1* ^*−/−*^ strain and not the other MAPK knockouts, the main growth‐inhibitory effect of the APIM‐peptide is likely intracellular and not a membrane effect.

Both the prostate cancer cell line Du145 and the multiple myeloma cell line JJN‐3 have previously been found to be sensitive to the APIM‐peptide as a single agent 6. The p38 inhibitor did not increase the sensitivity of Du145 cells toward the APIM‐peptide in the absence of DNA damage; however, it further reduced the cell growth of APIM‐peptide‐treated JJN‐3 cells (Fig. 2F,H). The antigrowth efficacy of cisplatin was increased in combination with either the p38 inhibitor or the APIM‐peptide in both cell lines, and the combination of p38 inhibition and APIM‐peptide further increased the growth‐inhibitory effect of cisplatin (Fig. 2G,I). Collectively, these results suggest that the functional impact of inhibiting interactions between PCNA and APIM‐containing proteins during cellular stress is conserved between yeast and human cells; that is, PCNA likely has a role in cellular signaling also in yeast.

The reasons why we see the hypersensitivity toward cisplatin and APIM‐peptide in the absence of Hog1/p38 MAPK signaling are likely complex; however, this conserved pathway controls cell cycle progression in response to stress in both yeast and human cells. The mammalian p38 substrate MK2, important for G2/M checkpoint regulation, contains APIM 11. The yeast Hog1 substrates Swe1 and Clb2 also contain APIM (Table 1). Hog1 delays cell cycle progression at G2/M by stabilizing the cell cycle inhibitor Swe1 and downregulating the transcription of the G2/M‐specific cyclin Clb2 19, 42. Thus, the G2/M checkpoint is impaired in both *Hog1* ^*−/−*^ *‐* and p38‐inhibited cells. Clb2 has also been shown to act together with Sgs1, ExoI, and Rad53 on recombination structures upon replication fork blocks, and Clb2 deletion mutants exhibit increased sensitivity toward DNA damaging agents 43. The APIM‐peptide could therefore hypothetically impair both APIM‐mediated Swe1‐PCNA and Clb2‐PCNA interactions and thereby affect the stability and/or the functions of Swe1 and Clb2. This could impair both the G2/M checkpoint and the DNA repair of cisplatin‐induced recombination structures caused by the replication blocks. Additionally, the APIM‐containing PI kinase Fab1 is activated upon hyperosmotic stress and PI(3,5)P_2_ is rapidly produced 44, 45, 46. Production of PI(3,5)P_2_ is therefore likely important for proper cellular stress responses, and this regulation might be impaired by the APIM‐peptide. One or all of these effects on top of the *Hog1* deletion likely explains the hypersensitivity of yeast cells to cisplatin.

In summary, here, we show that the PCNA‐binding sequence APIM is conserved in yeast. Many proteins involved in PI and MAPK signaling contain APIM, and we demonstrate that human and yeast cells become hypersensitive toward cisplatin when treated with APIM‐peptide in the absence of functional Hog1/p38 signaling. The observed hypersensitivity is likely due to competitive inhibition of protein interactions with PCNA in the presence of the APIM‐peptide. This impairs the cellular stress response and, when combined with absent of the stress‐activated Hog1/p38 signaling, this becomes lethal. Our data collectively suggest a functional conservation of the role of PCNA as a scaffold/platform protein in cellular signaling between yeast and human cells.

## Material and methods

### Sequence analysis/network analysis

Conserved occurrences of APIM in the proteome of *S. cerevisiae* were identified using the confind software tool as described in 3. This tool identifies potential motif occurrences in protein sequences from the target organism using a regular expression, and it tests hits against phylogenetic conservation in orthologous proteins from suitable reference organisms, so that only evolutionary conserved hits are reported as likely candidates. Mapping of orthologs was taken from the Inparanoid database version 7.0 47, and suitable reference proteomes were selected as fungal proteomes in Inparanoid representing all major phyla in the extensive phylogenetic analysis by Marcet‐Houben and Gabaldón 48. In total, eight reference proteomes were selected (*Kluyveromyces lactis*,* Candida albicans*,* Yarrowia lipolytica*,* Aspergillus fumigatus*,* Neurospora crassa*,* Schizosaccharomyces pombe*,* Cryptococcus neoformans*, and *Rhizopus oryzae*), and it was confirmed with Blast 49 that all selected proteomes had multiple occurrences of APIM. Orthologs were aligned using ClustalW 50 for the identification of conserved motifs.

AlkB homologue 2 PCNA‐interacting motif was initially found in 280 proteins from *S. cerevisiae*, but 60 of these did not have orthologs in any of the reference proteomes, and were removed. For another 136 proteins, the motif did not show sufficient conservation across the reference genomes. This resulted in 84 proteins where one or more APIM‐like motifs seemed to be conserved. Output from confind can be found as Supplementary Material (Table [Link], [Link], [Link]A‐C) and on Web at <http://tare.medisin.ntnu.no/pcna/index.php>.

Proteins containing APIM were first analyzed with DAVID version 6.8 20, 21, using *S. cerevisiae* S288c (default species) as background 51. Data for protein interaction network (PIN) were taken from BioGRID 52 and consisted of 5520 nodes and 56891 edges (high‐throughput, physical). Functional classification was taken from the FunCat Functional Catalogue version 2.1 from MIPS 22. PIN clusters were identified using community detection based on Louvain method 53, and enrichment for function (FunCat) within these clusters was estimated using null hypothesis significant test with *P*‐value 54. Each gene may belong to more than one functional class. Functional class of each individual gene was estimated by three different strategies, using either the most frequently occurring top‐level functional class, the top‐level functional class most frequently associated with sensitivity to methyl methanesulfonate (i.e., most likely to be associated with genomic stress), or just using all functional classes associated with the gene. These three strategies gave very similar results for key properties.

### Peptides

APIM‐peptide (Ac‐MDRWLVKWKKKRKIRRRRRRRRRRR) 6 and APIM‐peptide‐FAM were purchased from Innovagen, Sweden.

### Confocal imaging of yeast cells

The fluorescently labeled APIM‐peptide (APIM‐peptide‐FAM) was added to yeast cells resuspended in phosphate‐buffered saline. The fluorescent live images were acquired 2–5 min after addition, using a Zeiss LSM 510 Meta laser scanning microscope equipped with a Plan‐Apochromate 63 × /1.4 oil immersion objective, excitation λ = 488 nm, and detection λ = 505–530 nm.

### High‐throughput yeast cultivation

Homozygote diploid mutant yeast strains (BY4743 (*WT*), YRL113W (*Hog1* ^*−/−*^), YBL016W (*Fus3* ^*−/−*^), YPR054W (*Smk1* ^*−/−*^), YKL161 (*Mlp1* ^*−/−*^), and YGR040W (*Kss1* ^*−/−*^)) were purchased from EUROSCARF, Institute of Microbiology, University of Frankfurt.

Yeast strain growth studies were performed in 96‐well flat bottom microplates from Greiner. A volume of 20 μL, freshly thawed yeast strains (*WT*,* Hog1* ^*−/−*^, *Fus3* ^*−/−*^, *Smk1* ^*−/−*^, *Mlp1* ^*−/−*^, *Mpk1* ^*−/−*^ *,* and *Kss1* ^*−/−*^) were inoculated in 100 μL 2xMES 1.5xN‐base growth medium 55 and cultivated overnight (ON) at 30 °C, 900 r.p.m. shaking, in a humidified atmosphere (85%). One 96‐well plate was used for each yeast strain. 10 μL from each well with ON culture was transferred by a Beckman Coulter Robotic Core system with an integrated Beckman Coulter NX^P^ robotic liquid handling unit to new wells containing 100 μL fresh 2xMES 1.5xN‐base growth medium. After dilution and distribution, the robotic system was programmed to incubate the plates at 30 °C in a Thermo Cytomat 2 450S integrated robotic incubator equipped with shaking positions for microplates (1000 r.p.m. orbital shaking for 20 s prior to every time point OD measurement). The OD (600 nm) in each well was measured every 60 min using an integrated Beckman Coulter Paradigm microplate reader. A volume of 10 μL containing APIM‐peptide and/or cisplatin in/or 0.9% NaCl solution was added to each well after approximately 5 h of cultivation when the OD had reached approximately 0.15. Further, the growth of the yeast strains in the microplates was monitored for approximately 24 h and the OD was plotted as a function of time.

### Cell lines

The prostate cancer cell line, Du145, and the multiple myeloma cell line, JJN‐3, were cultured in RPMI (Sigma‐Aldrich) supplemented with 10% fetal bovine serum, 2 mm glutamine (Sigma‐Aldrich), 2.5 μg·mL^−1^ amphotericin B (Sigma‐Aldrich), and 100 μg·mL^−1^ gentamicin (Invitrogen). The cells were cultured at 37 °C in a humidified incubator.

### Viability assay

Du145 or JJN‐3 cells were seeded into 96‐well plates and treated with APIM‐peptide (6 μm), cisplatin (0.6 μm for Du145 and 0.4 μm for JJN‐3), and p38 inhibitor SB 203580 (10 μm; Sigma‐Aldrich) alone or in combination. Cells were exposed continuously and harvested on day one to four using the MTT assay as described 3. The average from at least three wells was used to calculate viability.

## Funding

This work was supported by grants from Program for Medical Technology, Norwegian University of Science and Technology (NTNU), Trondheim, Norway, and The Liaison Committee for Education, Research and Innovation in Central Norway, the Norwegian University of Science and Technology (NTNU), Trondheim, Norway. The funders had no role in the study design, data collection and analysis, decision to publish, or preparation of the manuscript.

## Author contributions

MO, PB, and FD planned and initiated the study. FD, SL, and EA performed the bioinformatics analyses. HFK, PB, MO, and CO performed the laboratory experiments. CO and MO wrote the manuscript.

## Data accessibility

Research data pertaining to this article are available as Supporting Information. [Correction added after online publication on 6 June 2018: reference to figshare data removed].

## Supporting information


**Fig. S1.** Smk‐/‐, Kss‐/‐, and Mlp1‐/‐ have similar sensitivity towards the APIM‐peptide as WT *S. cerevisiae*. (A)*Smk‐/‐*, (B) *Kss1‐/‐*, and (C) *Mlp1‐/‐ S. cerevisiae* treated with APIM‐peptide, cisplatin (125 μm), and the combination. Optical densities were measured every hour for 24 h (15 h are shown in the plot). Concentrations and symbol explanations are indicated in the panel below. Data shown as mean from *n* technical replicates from one biological replicate: controls (no treatment; *n* = 9), APIM‐peptide (*n* = 3), cisplatin (*n* = 7), and combinatorial treatment (*n* = 2).

**Table S1.** (A) Output from confind PIP‐hu.

**Table S1.** (B) Output from confind, APIM‐hu.

**Table S1.** (C) Output from confind, APIM‐Sc.

**Table S2.** Annotation clusters from enrichment analysis with DAVID.

**Table S3.** Enrichment for functional categories in APIM‐containing yeast proteins. Multiple FunCat functional categories (FunCat IDs) are considered for each protein, as shown in Table S1. Functional categories found in the PPI network (Table S3) are highlighted in bold.

**Table S4.** Enrichment for APIM‐containing yeast proteins in PPI clusters. Cluster ID 2 and 3 are significantly enriched (highlighted in bold). The third column shows FunCat functional categories (FunCat IDs) of APIM‐containing yeast proteins in each PPI cluster. The star sign means enriched for that category at 5% (*) and 1% (**) level. See Table S2 for FunCat IDs.
