# Supplementary material for: The role of PCNA as a scaffold protein in cellular signaling is functionally conserved between yeast and humans
Source: FEBS Open Bio. 2018 May 31;8(7):1135–45. doi: 10.1002/2211-5463.12442 (PMC6026702; doi:10.1002/2211-5463.12442)
Supplement: Supplementary file 1 — Fig. S1. Smk‐/‐, Kss‐/‐, and Mlp1‐/‐ have similar sensitivity towards the APIM‐peptide as WT S. cerevisiae. (A)Smk‐/‐, (B) Kss1‐/‐, and (C) Mlp1‐/‐ S. cerevisiae treated with APIM‐peptide, cisplatin (125 μm), and the combination. Optical densities were measured every hour for 24 h (15 h are shown in the plot). Concentrations and symbol explanations are indicated in the panel below. Data shown as mean from n technical replicates from one biological replicate: controls (no treatment; n = 9), APIM‐peptide (n = 3), cisplatin (n = 7), and combinatorial treatment (n = 2). [file FEB4-8-1135-s001.pdf]

# The role of PCNA as a scaffold protein in cellular signaling is functionally conserved between yeast and humans

Camilla Olaisen[1], Hans Fredrik N. Kvitvang[2], Sungmin Lee[2], Eivind Almaas[2], Per Bruheim[2], Finn Drabløs[1], and Marit Otterlei[1]*.

[1]Department of Clinical and Molecular Medicine, Faculty of Medicine and Health Sciences, Norwegian University of Science and Technology (NTNU), Trondheim, Norway.

[2]Department of Biotechnology and Food Science, Faculty of Natural Sciences, Norwegian University of Science and Technology (NTNU), Trondheim, Norway.

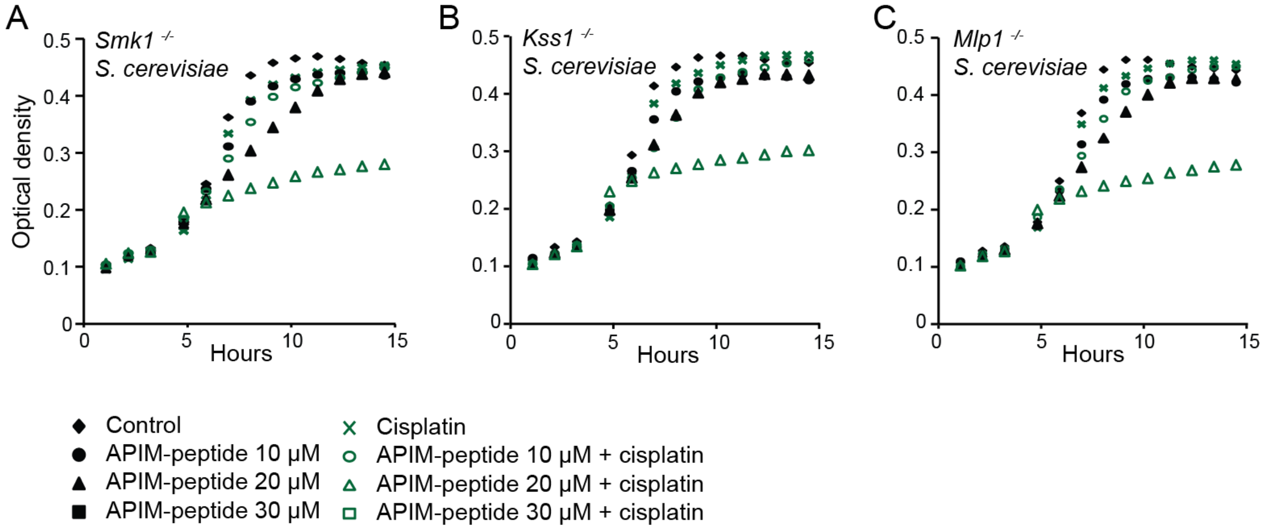


**Supplementary Figure S1.** ***Smk**$^{-/-}$**, Kss**$^{-/-}$***, and *Mlp1*$^{-/-}$ have similar sensitivity towards the APIM-peptide as WT *S. cerevisiae.***

(A) *Smk*$^{-/-}$, (B) *Kss1*$^{-/-}$, and (C) *Mlp1*$^{-/-}$ *S. cerevisiae* treated with APIM-peptide, cisplatin (125 µM), and the combination. Optical densities were measured every hour for 24 hours (15 hours are shown in the plot). Concentrations and symbol explanations are indicated in the panel below. Data shown as mean from n technical replicates from one biological replicate: controls (no treatment; n=9), APIM-peptide (n=3), cisplatin (n=7), and combinatorial treatment (n=2).
