# Supplementary material for: The role of PCNA as a scaffold protein in cellular signaling is functionally conserved between yeast and humans
Source: FEBS Open Bio. 2018 May 31;8(7):1135–45. doi: 10.1002/2211-5463.12442 (PMC6026702; doi:10.1002/2211-5463.12442)
Supplement: Supplementary file 2 — Table S1. (A) Output from confind PIP‐hu. [file FEB4-8-1135-s002.pdf]

The role of PCNA as a scaffold protein in cellular signaling is functionally conserved between yeast and humans

Camilla Olaisen[1], Hans Fredrik N. Kvitvang[2], Sungmin Lee[2], Eivind Almaas[2], Per Bruheim[2], Finn Drabløs[1], and Marit Otterlei[1*].

[1]Department of Clinical and Molecular Medicine, Faculty of Medicine and Health Sciences, Norwegian University of Science and Technology (NTNU), Trondheim, Norway.

[2]Department of Biotechnology and Food Science, Faculty of Natural Sciences, Norwegian University of Science and Technology (NTNU), Trondheim, Norway.

Supplementary Table S1 A: Output from confind PIP-hu

Shown below as pdf. Also found on web at

<http://tare.medisin.ntnu.no/pcna/index.php>

confind

```
confind (c) Finn Drablos, NTNU 2007,2012
Version 02.10.2012
Program started Mon Jul 06 13:34:53 CEST 2015
----------
Configuration file for PIP motif
Version FD 30-01-2012

Datasets are from Inparanoid 7.0. Gene descriptions are
mainly taken from Inparanoid 7.0. However, in case annotations
are not found in Inparanoid 7.0 they can be retrieved from
Ensembl 65 data. Localisation data are from eSLDB.

Genes that are mentioned in the paper are highlighted
in green, in the order they are listed in the paper.

Using K as equivalence for KR in consensus
Using F as equivalence for FYW in consensus
Using L as equivalence for LVI in consensus
----------
Found 21673 sequences in ./lib/H.sapiens.fa
Got match in 686 sequences against pattern Q..[ILM]..[FHD][FY]
Removed 0 entries with no info in ./ref/eSLDB_Homo_sapiens_TS.txt matching .*
There are 686 entries left
Found 562 orthologs in Bt (Bos taurus)
Found 557 orthologs in Rn (Rattus norvegicus)
Found 607 orthologs in Mm (Mus musculus)
Found 474 orthologs in Gg (Gallus gallus)
Found 385 orthologs in Xt (Xenopus tropicalis)
Found 360 orthologs in Dr (Danio rerio)
Found 164 orthologs in Ce (Caenorhabditis elegans)
Found 206 orthologs in Dm (Drosophila melanogaster)
Found 88 orthologs in Sc (Saccharomyces cerevisiae)
Removed 47 entries without orthologs
There are 639 entries left
Created 202 new sequence files
Added 978 new orthologs to these sequence files
Ran ClustalW on 202 library files
Removed 118 sequences where pattern was not conserved
There are 521 entries left
Missing information on gene for 0 proteins
Removed 0 duplicate sequences representing the same gene
There are 521 entries left
Description is missing for 0 genes
ENSG00000189046 from priority list was not found
ENSG00000116205 from priority list was not found
ENSG00000077809 from priority list was not found
ENSG00000131747 from priority list was not found
ENSG00000136936 from priority list was not found
ENSG00000076924 from priority list was not found
ENSG00000143799 from priority list was not found
ENSG00000129484 from priority list was not found
ENSG00000182185 from priority list was not found
ENSG00000009413 from priority list was not found
ENSG00000174405 from priority list was not found
ENSG00000158169 from priority list was not found
ENSG00000034063 from priority list was not found
ENSG00000159459 from priority list was not found
ENSG00000024048 from priority list was not found
----------
Pro - Protein number (green if on priority list)
# - Motif number i protein
CI - Entry is accepted according to Consensus or Individual pattern hits
Protein ID / Gene ID - IDs from input data
Pos - Position of motif in sequence
Alignment - Alignment of motif (UPPER case) and flanking region (lower case)
   lower case only - no hit by motif, "-----" - gaps, "....." - missing protein
Ortho ID - ID of Orthologs, with organism code (see above)
Description
```

Pro	#	CI	Protein ID<br>Gene ID	Pos	Alignment	Ortho ID	Description
1	1	CI	ENSP00000371419<br>ENSG00000102699	318<br>319<br>-<br>326<br>324<br>314<br>-<br>-<br>-<br>-	lkngetaeqlQKMMTEFYrliphkgtmp<br>mrngetqeqlQTMLTEFYrliphraava<br>............................<br>lkngdspgqlQKTMAEFYrllphrhpas<br>lneganetalQEMMMEFYqlirhkteid<br>ldtrtepgdirkamaefyrliphkkplq<br>............................<br>............................<br>............................<br>............................	Hs_ENSP00000371419<br>Bt_ENSBTAP00000035522<br>Rn<br>Mm_ENSMUSP00000066130<br>Gg_ENSGALP00000027638<br>Xt_ENSXETP00000043695<br>Dr<br>Ce<br>Dm<br>Sc	NP_006428 Poly
2	1	CI	ENSP00000264331<br>ENSG00000077097	1037<br>1009<br>-<br>1025<br>1044<br>1014<br>1031<br>1052<br>998<br>991	mgclkkyetvQDILKEFFdlrlsyyglr<br>mgclkkyetvQDILKEFFdlrlsyyglr<br>............................<br>mgclkkyetvQDILKEFFdlrlsyyglr<br>mgclkkyetvQDILKEFFdlrlhyyslr<br>mgclkkyetvQDILKEFFdlrlnyytlr<br>mgcmkryesvQEILKEFFelrlhyyklr<br>agclrtytspeaitqefydsrqekyvqr<br>nnclrrfptaidilkeyyklrreyyarr<br>hgkikkynsvneilsefyyvrleyyqkr	Hs_ENSP00000264331<br>Bt_ENSBTAP00000006030<br>Rn<br>Mm_ENSMUSP00000017629<br>Gg_ENSGALP00000018410<br>Xt_ENSXETP00000015835<br>Dr_ENSDARP00000030178<br>Ce_CE06184<br>Dm_FBpp0080825<br>Sc_YNL088W	NP_001059 DNA topoisomerase 2-beta (EC 5.99.1.3)(DNA topoisomerase II, beta isozyme)
3	1	CI	ENSP00000263274<br>ENSG00000105486	2<br>2<br>1<br>18<br>0<br>0<br>2<br>0<br>2<br>2	---------mQRSIMSFFhpkk-egkak<br>---------mQRSIMSFFqpkk-egkak<br>----------QHSLKSFFqptttegkak<br>rkgetsaanmQRSIMSFFqptk-egkak<br>----------------------------<br>-------------irsffqpklgaevkk<br>---------mQRSIASFFqpmkgkeksd<br>----------------------------<br>---------mQKSITSFFkkks------<br>---------mrrlltgcllssarplksr	Hs_ENSP00000263274<br>Bt_ENSBTAP00000019427<br>Rn_ENSRNOP00000019799<br>Mm_ENSMUSP00000096411<br>Gg_ENSGALP00000039729<br>Xt_ENSXETP00000019357<br>Dr_ENSDARP00000078609<br>Ce_CE37480<br>Dm_FBpp0072041<br>Sc_YDL164C	NP_000225 DNA ligase 1 (EC 6.5.1.1)(DNA ligase I)(Polydeoxyribonucleotide synthase
4	1	CI	ENSP00000276893<br>ENSG00000147854	788<br>788<br>789<br>789<br>741<br>778<br>-<br>-<br>-<br>-	nyimipneilQTLLDLFFpgyskgr---<br>nyimipnavlQTLLDLFFpgyskgr---<br>nyimtlnetlQTVLDLFFpgyskgr---<br>nyvmvlnetlQTLLDLFFpgyskgr---<br>sytmvpnkilQTLLDQFFpgyskgr---<br>synmvpnkvlQTLLDQFFpgyskgr---<br>............................<br>............................<br>............................<br>............................	Hs_ENSP00000276893<br>Bt_ENSBTAP00000027737<br>Rn_ENSRNOP00000015406<br>Mm_ENSMUSP00000025739<br>Gg_ENSGALP00000024227<br>Xt_ENSXETP00000001255<br>Dr<br>Ce<br>Dm<br>Sc	NP_690856 E3 ubiquitin-protein ligase UHRF2 (EC 6.3.2.-)(Ubiquitin-like PHD and RING finger domain-containing protein 2)(Ubiquitin-like-containing PHD and RING finger domains protein 2)(Np95/ICBP90-like RING finger protein)(Np95-like RING finger protein)(Nuclear zinc finger protein Np97)(RING finger protein 107)
5	1	CI	ENSP00000230588<br>ENSG00000112818	341<br>214<br>343<br>353<br>345<br>341<br>312<br>-<br>-<br>-	esrilypkrkQQCLQFFYkmtgspsdrl<br>esrilypkrkQQCLQFFYkmsgspsdrl<br>esrilypkrkQQCLQFFYkmtgspadrl<br>esrilypkrkQQCLQFFYkmtgspadrf<br>esrilypkrtQQCLQFFYkmsgsssdkl<br>esrilypkrseqclqffykmdgssqdrl<br>esrilypkrnQQCLEFFYrmsgepgdkl<br>............................<br>............................<br>............................	Hs_ENSP00000230588<br>Bt_ENSBTAP00000005686<br>Rn_ENSRNOP00000039733<br>Mm_ENSMUSP00000024707<br>Gg_ENSGALP00000026921<br>Xt_ENSXETP00000035476<br>Dr_ENSDARP00000009962<br>Ce<br>Dm<br>Sc	NP_005579 Meprin A subunit alpha Precursor (EC 3.4.24.18)(Endopeptidase-2)(N-benzoyl-L-tyrosyl-P-amino-benzoic acid hydrolase subunit alpha)(PABA peptide hydrolase)(PPH alpha)
6	1	C	ENSP00000338474<br>ENSG00000180881	359<br>294<br>309<br>292<br>293<br>-<br>53<br>-<br>-<br>-	shqcgrrkgkQYRLGDFYvgatltflss<br>chqrgrrkgkqyrladlyvganltflss<br>chqygqrkgeQYELGDFYtgatltflsc<br>hhqcgrrkgkqyelgdvytgatltflsc<br>qhqrgqkkgkaydltdfyiganltfqsi<br>............................<br>wn-----rgrpyclqdisqgaelhfct-<br>............................<br>............................<br>............................	Hs_ENSP00000338474<br>Bt_ENSBTAP00000046639<br>Rn_ENSRNOP00000033147<br>Mm_ENSMUSP00000089815<br>Gg_ENSGALP00000016579<br>Xt<br>Dr_ENSDARP00000075799<br>Ce<br>Dm<br>Sc	NP_115995 Calcyphosin-2 (Calcyphosine-2)
7	1	C	ENSP00000259089<br>ENSG00000136573	170<br>166<br>164	slsvkdvt-tQGELIKHYkircldeggy<br>slsvkdvt-tqgevikhykirsldeggy<br>slsvkdvt-sQGEMVKHYkirsldnggy	Hs_ENSP00000259089<br>Bt_ENSBTAP00000006659<br>Rn_ENSRNOP00000015021	NP_001706 Tyrosine-protein kinase BLK (EC 2.7.10.2)(B lymphocyte kinase)(p55-BLK)

				164<br>167<br>-<br>151<br>-<br>-<br>-	slsvkdit-tqgevvkhykirsldnggy<br>slsvrdsdsahgdiikhyrirsldgggy<br>............................<br>slsirdstpeqgdvvkhykiraldnggy<br>............................<br>............................<br>............................	Mm_ENSMUSP00000014597<br>Gg_ENSGALP00000026839<br>Xt<br>Dr_ENSDARP00000008826<br>Ce<br>Dm<br>Sc	
7	2	CI	ENSP00000259089<br>ENSG00000136573	487<br>483<br>481<br>481<br>484<br>-<br>467<br>-<br>-<br>-	peerptfeflQSVLEDFYtaterqyelq<br>peerptfeflqsvledfhtategqyelq<br>peerptfeflQSVLEDFYtategqyelq<br>peerptfeflQSVLEDFYtategqyelq<br>peerptfeylQSVLEDFYtatekqyeae<br>............................<br>pedrptfeylQDTLNDFFiategqyemq<br>............................<br>............................<br>............................	Hs_ENSP00000259089<br>Bt_ENSBTAP00000006659<br>Rn_ENSRNOP00000015021<br>Mm_ENSMUSP00000014597<br>Gg_ENSGALP00000026839<br>Xt<br>Dr_ENSDARP00000008826<br>Ce<br>Dm<br>Sc	NP_001706 Tyrosine-protein kinase BLK (EC 2.7.10.2)(B lymphocyte kinase)(p55-BLK)
8	1	C	ENSP00000386935<br>ENSG00000197355	16<br>-<br>16<br>16<br>16<br>-<br>15<br>-<br>-<br>-	dvrarlqragqehllrfwaelapepraa<br>............................<br>dvraklqragqdhllrfwadlapelraa<br>dvraqlqragQDHLLRFYadlapearaa<br>evrarleragqghllrfwaeldpaqrae<br>............................<br>eakakleaagqthvlqfwdelsaeergt<br>............................<br>............................<br>............................	Hs_ENSP00000386935<br>Bt<br>Rn_ENSRNOP00000017373<br>Mm_ENSMUSP00000099989<br>Gg_ENSGALP00000033549<br>Xt<br>Dr_ENSDARP00000025374<br>Ce<br>Dm<br>Sc	NP_997192 UDP-N-acetylhexosamine pyrophosphorylase-like protein 1 (EC 2.7.7.-)
9	1	C	ENSP00000328854<br>ENSG00000184967	286<br>298<br>286<br>286<br>289<br>297<br>295<br>228<br>267<br>291	vhdailpqlaQPTLMIDFltracdlg--<br>mhdsilphlaQPSLMIDFltraydig--<br>mhdsilphlaQPTLMIDFltsacdvg--<br>mhdsilphlaQPTLMIDFltsacdvg--<br>lhdsilpymneptlmidfltvaygvg--<br>lhesilphmskptlmidfltaaydvg--<br>lhesilpqmsdpklmmdflsaaydig--<br>itqnvisklkapfksadfffkmfdkt--<br>llervinhlndpiqltdflmdslhqfd-<br>lhkriiphfhtptklmdfltdsynlqss	Hs_ENSP00000328854<br>Bt_ENSBTAP00000015774<br>Rn_ENSRNOP00000053566<br>Mm_ENSMUSP00000038263<br>Gg_ENSGALP00000003557<br>Xt_ENSXETP00000004367<br>Dr_ENSDARP00000067000<br>Ce_CE01409<br>Dm_FBpp0070561<br>Sc_YPR144C	NP_076983 Nucleolar complex protein 4 homolog (NOC4 protein homolog)(NOC4-like protein)(Nucleolar complex-associated protein 4-like protein)
10	1	CI	ENSP00000362401<br>ENSG00000173198	252<br>255<br>266<br>267<br>254<br>250<br>259<br>-<br>-<br>-	flvsfmpyhiQRTIHLHFlhnetkpcds<br>flisfmpyhiQRTIHLHFlhnetkpcds<br>flvsfmpyhiQRAIHLHFlhsetrscds<br>flvsfmpyhiqrtihlhllhsetrpcds<br>flvsftpyhilrtvhlhvlrlrhasced<br>fflsfmpyhiQRTIHLHFlkg-nkncke<br>flisfmpyhiQRTLHLHFksrksatcee<br>............................<br>............................<br>............................	Hs_ENSP00000362401<br>Bt_ENSBTAP00000034249<br>Rn_ENSRNOP00000040761<br>Mm_ENSMUSP00000109108<br>Gg_ENSGALP00000006561<br>Xt_ENSXETP00000022341<br>Dr_ENSDARP00000075538<br>Ce<br>Dm<br>Sc	NP_006630 Cysteinyl leukotriene receptor 1 (CysLTR1)(Cysteinyl leukotriene D4 receptor)(LTD4 receptor)(HG55)(HMTMF81)
11	1	CI	ENSP00000256861<br>ENSG00000123243	251<br>252<br>-<br>251<br>233<br>223<br>294<br>-<br>-<br>-	ptvvqqariaQNGILGDFiirydvnreq<br>psvvqqakiaQNGILGDFivrydvnreq<br>............................<br>ptvvqqakiaQNGILGDFivrydvereq<br>pnvveqtkiarngilgdfivrydvnrel<br>psvvhqaklsQNGILGDFvirydvqrel<br>pniaqqakiatngmlgefvvhydveret<br>............................<br>............................<br>............................	Hs_ENSP00000256861<br>Bt_ENSBTAP00000017293<br>Rn<br>Mm_ENSMUSP00000026886<br>Gg_ENSGALP00000011148<br>Xt_ENSXETP00000003930<br>Dr_ENSDARP00000066933<br>Ce<br>Dm<br>Sc	NP_085046 Inter-alpha-trypsin inhibitor heavy chain H5 Precursor (Inter-alpha-inhibitor heavy chain 5)(ITI heavy chain H5)
11	2	C	ENSP00000256861<br>ENSG00000123243	899<br>898<br>-<br>899<br>880<br>817<br>950<br>-<br>-<br>-	vwkqrkiyngeeqidcwfarnnaaklid<br>vwkqrriyngeeqvdcwfarnnaaglid<br>............................<br>vwkqrkiyngqaqvdcwfdrnnaaklid<br>vwkqrriyngqqqvdcwfaknnadklid<br>----------------------------<br>mkksrriysgkqtvdcwfaknnaaklid<br>............................<br>............................<br>............................	Hs_ENSP00000256861<br>Bt_ENSBTAP00000017293<br>Rn<br>Mm_ENSMUSP00000026886<br>Gg_ENSGALP00000011148<br>Xt_ENSXETP00000003930<br>Dr_ENSDARP00000066933<br>Ce<br>Dm<br>Sc	NP_085046 Inter-alpha-trypsin inhibitor heavy chain H5 Precursor (Inter-alpha-inhibitor heavy chain 5)(ITI heavy chain H5)
12	1	CI	ENSP00000262716<br>ENSG00000074657	945<br>923<br>779	csmcdtvftlQTLLYRHFdqhienqkvs<br>csmcdtvftlQTLLYRHFdqhienqkvs<br>----------------------------	Hs_ENSP00000262716<br>Bt_ENSBTAP00000043725<br>Rn_ENSRNOP00000023048	NP_060651 Zinc finger protein 532

				779<br>848<br>-<br>894<br>-<br>-<br>-	----------------------------<br>----------------------------<br>............................<br>csmcdtvftqQTLLYTHFdqhissqkvs<br>............................<br>............................<br>............................	Mm_ENSMUSP00000036582<br>Gg_ENSGALP00000004487<br>Xt<br>Dr_ENSDARP00000078470<br>Ce<br>Dm<br>Sc	
13	1	CI	ENSP00000362814<br>ENSG00000168528	183<br>-<br>174<br>174<br>-<br>170<br>-<br>-<br>-<br>-	vvgsflfiliQLVLLIDFahswnqrwlg<br>............................<br>vvgsflfiliQLILFIDFahswnqrwlc<br>vvgsflfiliQLILFVDFahswnqrwlc<br>............................<br>mvggflfiliqliliidlahgwsqswlq<br>............................<br>............................<br>............................<br>............................	Hs_ENSP00000362814<br>Bt<br>Rn_ENSRNOP00000017577<br>Mm_ENSMUSP00000075100<br>Gg<br>Xt_ENSXETP00000028280<br>Dr<br>Ce<br>Dm<br>Sc	Serine incorporator 2 (Tumor differentially expressed 2-like)
14	1	CI	ENSP00000310668<br>ENSG00000102900	441<br>441<br>442<br>441<br>441<br>440<br>442<br>413<br>414<br>463	pqdrltlsqfQKQLLEDYgeshftvnqq<br>pqdrltlsqfQKQLLEDYgeshftvnqq<br>pqdrltlsqfQKQLLEDYgeshftvnqq<br>pqdrltlsqfQKQLLEDYgeshftvnqq<br>sqdrltlsqfQKQLLEDYgeshfavnqq<br>pqdrltlpqfQKQLFEDYgeshfavnqq<br>pqdrmtlaqlQKQLLEDYgeshfsashq<br>qltdvlfkevqkavsvdygeqyfmsngp<br>--------klqsmileqygekyfnarqq<br>vyerysledfqniiis-ygpsrfsn---	Hs_ENSP00000310668<br>Bt_ENSBTAP00000008683<br>Rn_ENSRNOP00000025086<br>Mm_ENSMUSP00000078878<br>Gg_ENSGALP00000004745<br>Xt_ENSXETP00000039971<br>Dr_ENSDARP00000013627<br>Ce_CE26832<br>Dm_FBpp0082467<br>Sc_YFR002W	NP_055484 Nuclear pore complex protein Nup93 (Nucleoporin Nup93)(93 kDa nucleoporin)
15	1	CI	ENSP00000352412<br>ENSG00000171505	5<br>23<br>6<br>8<br>-<br>-<br>-<br>-<br>-<br>-	------vmenQSSISEFFlrgisappeq<br>dqrcsrvmenQTSISEFFlrgipgspeq<br>-----ilmenqssvsefflqgisgfpeq<br>---csilmenessvsefflqgisgfseq<br>............................<br>............................<br>............................<br>............................<br>............................<br>............................	Hs_ENSP00000352412<br>Bt_ENSBTAP00000053046<br>Rn_ENSRNOP00000048696<br>Mm_ENSMUSP00000108569<br>Gg<br>Xt<br>Dr<br>Ce<br>Dm<br>Sc	NP_036495 Olfactory receptor 1N1 (Olfactory receptor 1-26) (OR1-26)(Olfactory receptor 1N3)(Olfactory receptor OR9-22)
16	1	CI	ENSP00000310219<br>ENSG00000173110	349<br>347<br>-<br>-<br>-<br>-<br>-<br>-<br>-<br>-	vggstripkvQKLLQDFFngkelnksin<br>vggstripkvQKLLQDFFdgrelnksin<br>............................<br>............................<br>............................<br>............................<br>............................<br>............................<br>............................<br>............................	Hs_ENSP00000310219<br>Bt_ENSBTAP00000051168<br>Rn<br>Mm<br>Gg<br>Xt<br>Dr<br>Ce<br>Dm<br>Sc	NP_002146 Heat shock 70 kDa protein 6 (Heat shock 70 kDa protein B')
17	1	CI	ENSP00000225387<br>ENSG00000108255	138<br>138<br>138<br>138<br>131<br>137<br>113<br>-<br>-<br>-	ifekenfigrQWEISDDYp----slqam<br>ifekenfigrQWEICDDYp----slqam<br>ifekenfigrQWEICDDYp----slqam<br>ifekenfigrQWEICDDYp----slqam<br>vyekdnfigrQWEISDDYpddypslqam<br>vfekenfigrkwdmcddyp----slqam<br>vferenfighQWEMTDDYp----slqam<br>............................<br>............................<br>............................	Hs_ENSP00000225387<br>Bt_ENSBTAP00000007037<br>Rn_ENSRNOP00000011608<br>Mm_ENSMUSP00000077693<br>Gg_ENSGALP00000006416<br>Xt_ENSXETP00000020830<br>Dr_ENSDARP00000048719<br>Ce<br>Dm<br>Sc	NP_005199 Beta-crystallin A3
18	1	CI	ENSP00000362409<br>ENSG00000136830	520<br>512<br>502<br>520<br>151<br>499<br>519<br>-<br>-<br>-	ptckselprfQELIFEDFarfilventy<br>ptckselsrfQELIFEDFarfilventy<br>ptckselprfQELIFEDFarfilventy<br>ptckselprfQELIFEDFarfilventy<br>ptckgelakfQELIFEDFasfilventy<br>ptckaefprfqelifedlsryvlventy<br>pscspdlpkfkelifedfsrfilvenif<br>............................<br>............................<br>............................	Hs_ENSP00000362409<br>Bt_ENSBTAP00000017069<br>Rn_ENSRNOP00000021689<br>Mm_ENSMUSP00000028135<br>Gg_ENSGALP00000039286<br>Xt_ENSXETP00000031228<br>Dr_ENSDARP00000092973<br>Ce<br>Dm<br>Sc	NP_073744 Niban-like protein 1 (Protein FAM129B)(Meg-3)
19	1	CI	ENSP00000321345<br>ENSG00000162594	614<br>637<br>-	elpsintyfpQNILESHFnrisllek--<br>elpsinsyfpQNILESHFnrisllek--<br>............................	Hs_ENSP00000321345<br>Bt_ENSBTAP00000052690<br>Rn	NP_653302 Interleukin-23 receptor Precursor (IL-23R)

				644<br>-<br>-<br>-<br>-<br>-<br>-	dlpsinsyfpQNVLESHFsrislfqk--<br>............................<br>............................<br>............................<br>............................<br>............................<br>............................	Mm_ENSMUSP00000113342<br>Gg<br>Xt<br>Dr<br>Ce<br>Dm<br>Sc	
20	1	CI	ENSP00000267176<br>ENSG00000139697	661<br>663<br>660<br>661<br>659<br>-<br>-<br>-<br>1033<br>-	vstak--gvlQSLIEKHFpapdrkklys<br>vstaknrgvlQSLIEKHFpapdrkklys<br>vstak--gvlQSLIEKHFpapdrkklys<br>vstak--gvlQSLIEKHFpapdrkklys<br>vstak--gvfQSLIEKHFpapdrkklfs<br>............................<br>............................<br>............................<br>vstak--gvfqsfverhfpapdrnrinr<br>............................	Hs_ENSP00000267176<br>Bt_ENSBTAP00000027325<br>Rn_ENSRNOP00000001410<br>Mm_ENSMUSP00000066808<br>Gg_ENSGALP00000005353<br>Xt<br>Dr<br>Ce<br>Dm_FBpp0111762<br>Sc	NP_060653 Protein strawberry notch homolog 1 (Monocyte protein 3)(MOP-3)
20	2	C	ENSP00000267176<br>ENSG00000139697	1117<br>1119<br>1116<br>1116<br>1114<br>-<br>-<br>-<br>1555<br>-	nrilgmevhqqnalfqyfadtltavvqn<br>nrilgmevhqqnalfqyfadtltavvqn<br>nrilgmevhqqnalfqyfadtltavvqn<br>nrilgmevhqqnalfqyfadtltavvqn<br>nrilgmevhqqnalfqyfsdtlnaviqn<br>............................<br>............................<br>............................<br>nrilgcpvdlqnrlfkyftdtmtaiiqq<br>............................	Hs_ENSP00000267176<br>Bt_ENSBTAP00000027325<br>Rn_ENSRNOP00000001410<br>Mm_ENSMUSP00000066808<br>Gg_ENSGALP00000005353<br>Xt<br>Dr<br>Ce<br>Dm_FBpp0111762<br>Sc	NP_060653 Protein strawberry notch homolog 1 (Monocyte protein 3)(MOP-3)
21	1	C	ENSP00000215727<br>ENSG00000099937	229<br>226<br>209<br>208<br>202<br>215<br>237<br>-<br>-<br>-	rsvndlyiqkQFPILLDFktkvreyyfa<br>rsvsdlyvqkqlpvlddfktqvreyyfa<br>qsvndlyiqkQFPIREDFkaamrefyfa<br>rsvnglyiqkQFPIREDFkaamrefyfa<br>rsvndlyirkdfsilndfrnnmktyyfa<br>rsvndiyvkkdfvirepfknnlknyyfa<br>rsvndlyvkrnvqiqdsfradaktyyfa<br>............................<br>............................<br>............................	Hs_ENSP00000215727<br>Bt_ENSBTAP00000018574<br>Rn_ENSRNOP00000031829<br>Mm_ENSMUSP00000023450<br>Gg_ENSGALP00000002121<br>Xt_ENSXETP00000048524<br>Dr_ENSDARP00000017995<br>Ce<br>Dm<br>Sc	NP_000176 Heparin cofactor 2 Precursor (Heparin cofactor II)(HC-II)(Protease inhibitor leuserpin 2)(HLS2)
22	1	CI	ENSP00000257963<br>ENSG00000135503	277<br>260<br>275<br>277<br>289<br>302<br>277<br>-<br>-<br>-	adnkdngtwtQLWLVSDYhehgslfdyl<br>adnkdngtwtQLWLVSDYhehgslfdyl<br>adnkdngtwtQLWLVSDYhehgslfdyl<br>adnkdngtwtQLWLVSDYhehgslfdyl<br>adnkdngtwtQLWLVSDYhehgslfdyl<br>adnkdngtwtQLWLVSDYhehgslfdyl<br>adnkdngtwtQLWLVSDYhehgslfdyl<br>............................<br>............................<br>............................	Hs_ENSP00000257963<br>Bt_ENSBTAP00000043941<br>Rn_ENSRNOP00000009345<br>Mm_ENSMUSP00000000544<br>Gg_ENSGALP00000023536<br>Xt_ENSXETP00000001261<br>Dr_ENSDARP00000004431<br>Ce<br>Dm<br>Sc	NP_004293 Activin receptor type-1B Precursor (EC 2.7.11.30)(ACTR-IB)(Serine/threonine-protein kinase receptor R2)(SKR2)(Activin receptor-like kinase 4)(ALK-4)
23	1	CI	ENSP00000295240<br>ENSG00000163093	174<br>175<br>174<br>174<br>175<br>160<br>175<br>184<br>192<br>-	ingvwnlssdQGNLGTFFitnvrivwha<br>ingvwnlssdQGNLGTFFitnvrivwha<br>ingvwnlssdQGNLGTFFitnvrivwha<br>ingvwnlssdQGNLGTFFitnvrivwha<br>ingvwnlssdQGNLGTFFitnvrvvwha<br>ingvwnlssdQGNLGTFFitnvrivwha<br>ingvwnlssdQGNLGTFFitnvrivwha<br>vngvwnlstetgslgvfvitnirvvwya<br>vqgvwnlssdqgnlgsfvvtnirlvwfa<br>............................	Hs_ENSP00000295240<br>Bt_ENSBTAP00000014216<br>Rn_ENSRNOP00000009396<br>Mm_ENSMUSP00000074494<br>Gg_ENSGALP00000015990<br>Xt_ENSXETP00000041080<br>Dr_ENSDARP00000058254<br>Ce_CE01042<br>Dm_FBpp0078512<br>Sc	NP_689597 Bardet-Biedl syndrome 5 protein
24	1	CI	ENSP00000295694<br>ENSG00000163472	289<br>290<br>286<br>286<br>-<br>-<br>239<br>-<br>-<br>-	ihrryvaqsvQLFILYFFnlavlstylp<br>ihrqyvaqsvQLFILYFFnlavlstylp<br>ihrqyvaqsvQLFILYFFnlavlstylp<br>ihrqyvaqsvQLFILYFFnlavlstylp<br>............................<br>............................<br>vhrhyvreslqlfllyflqlsvmatyth<br>............................<br>............................<br>............................	Hs_ENSP00000295694<br>Bt_ENSBTAP00000012416<br>Rn_ENSRNOP00000026414<br>Mm_ENSMUSP00000001456<br>Gg<br>Xt<br>Dr_ENSDARP00000095983<br>Ce<br>Dm<br>Sc	NP_115699 Transmembrane protein 79
25	1	C	ENSP00000312411<br>ENSG00000175175	175<br>20<br>172	laratsdevlQSDLSAHYipketdg---<br>laratsdevlQSDLSAHYipketdg---<br>laratsdevlqsdlsahcipketdg---	Hs_ENSP00000312411<br>Bt_ENSBTAP00000014279<br>Rn_ENSRNOP00000003859	NP_055721 Protein phosphatase 1E (EC 3.1.3.16)(Ca(2+)/calmodulin-dependent

				172<br>20<br>51<br>81<br>-<br>-<br>-	laratsdevlqsdlsahcipketdg---<br>laratsdevlQSDLSAHYlpkhvdn---<br>laratadeilqsdlsvyyspkhvdg---<br>largtadsilqsdlslyhlnksvedgte<br>............................<br>............................<br>............................	Mm_ENSMUSP00000061278<br>Gg_ENSGALP00000008101<br>Xt_ENSXETP00000002455<br>Dr_ENSDARP00000063550<br>Ce<br>Dm<br>Sc	protein kinase phosphatase N)(CaMKP-N)(CaMKP-nucleus)(CaMKN)(Partner of PIX 1)(Partner of PIX-alpha)(Partner of PIXA)
26	1	CI	ENSP00000350511<br>ENSG00000118194	113<br>-<br>119<br>120<br>118<br>-<br>107<br>-<br>-<br>-	krmekdlnelQALIEAHFenrkkeeeel<br>............................<br>krmekdlnelQTLIEAHFenrkkeeeel<br>krvekdlnelQTLIEAHFenrkkeeeel<br>krmekdlnelQALIEAHFesrkkeeeel<br>............................<br>krmekdlnelQTLIEAHFesrkkeeeei<br>............................<br>............................<br>............................	Hs_ENSP00000350511<br>Bt<br>Rn_ENSRNOP00000057224<br>Mm_ENSMUSP00000107717<br>Gg_ENSGALP00000000400<br>Xt<br>Dr_ENSDARP00000093537<br>Ce<br>Dm<br>Sc	NP_000355 Troponin T, cardiac muscle (TnTc)(Cardiac muscle troponin T)(cTnT)
27	1	CI	ENSP00000354652<br>ENSG00000164128	328<br>-<br>327<br>327<br>330<br>323<br>337<br>-<br>-<br>-	ifygflnknfQRDLQFFFnfcdfrs-rd<br>............................<br>ifygflnknfQRDLQFFFnfcdfrs-rd<br>ifygflnknfQRDLQFFFnfcdfrs-rd<br>ifygflnknfqrdlqflfhfchfrs-re<br>ifygflnknfQRDLQFFFnfcdfrs-re<br>ifygflnrnfqrdlr-afrlckivstre<br>............................<br>............................<br>............................	Hs_ENSP00000354652<br>Bt<br>Rn_ENSRNOP00000018952<br>Mm_ENSMUSP00000045530<br>Gg_ENSGALP00000015445<br>Xt_ENSXETP00000010668<br>Dr_ENSDARP00000054443<br>Ce<br>Dm<br>Sc	NP_000900 Neuropeptide Y receptor type 1 (NPY1-R)
28	1	CI	ENSP00000333947<br>ENSG00000182022	533<br>533<br>533<br>533<br>532<br>-<br>-<br>-<br>-<br>-	rnlgpmwpitQKILRDFYrpfnarlaqv<br>rdlgpmwpvtQRLLRDFYgpfnarlaqv<br>rslgpmwpitQKILRDFYgpfntrlaqv<br>rslgpmwpitQKILREFYgpfntrlaqv<br>rslgpmlpttkailrdfyrpfntklaqv<br>............................<br>............................<br>............................<br>............................<br>............................	Hs_ENSP00000333947<br>Bt_ENSBTAP00000004287<br>Rn_ENSRNOP00000022157<br>Mm_ENSMUSP00000076682<br>Gg_ENSGALP00000015782<br>Xt<br>Dr<br>Ce<br>Dm<br>Sc	NP_056976 N-acetylgalactosamine 4-sulfate 6-O-sulfotransferase (EC 2.8.2.33)(GalNAc4S-6ST)(B-cell RAG-associated gene protein)(hBRAG)
29	1	CI	ENSP00000358305<br>ENSG00000131788	553<br>553<br>544<br>553<br>-<br>562<br>-<br>-<br>-<br>-	gpsvitsldeQDALGHFFqyrgtpshfl<br>gpsvitsldeQDALGHFFqyrgtpshfl<br>spsvitsldeQDTLGHFFqfrgtpphfl<br>gpsvitsldeQDTLGHFFqyrgtpshfl<br>............................<br>spsvitsldeQDALSHFFqyrappghym<br>............................<br>............................<br>............................<br>............................	Hs_ENSP00000358305<br>Bt_ENSBTAP00000028829<br>Rn_ENSRNOP00000028814<br>Mm_ENSMUSP00000069259<br>Gg<br>Xt_ENSXETP00000040400<br>Dr<br>Ce<br>Dm<br>Sc	NP_006090 E3 SUMO-protein ligase PIAS3 (Protein inhibitor of activated STAT protein 3)
30	1	C	ENSP00000375773<br>ENSG00000054392	463<br>-<br>460<br>469<br>414<br>464<br>-<br>478<br>465<br>-	gktywnrifiQGGLFLFFllnpcwetaf<br>............................<br>gktywnriflqg-----------wp---<br>gktywnriflqg-----------wp---<br>----------------------------<br>kkcflcklrfvq----------cwp---<br>............................<br>gaafvqrflieetitfrwp---------<br>gdflmrgaylsgvgn-------------<br>............................	Hs_ENSP00000375773<br>Bt<br>Rn_ENSRNOP00000005223<br>Mm_ENSMUSP00000046686<br>Gg_ENSGALP00000016047<br>Xt_ENSXETP00000000465<br>Dr<br>Ce_CE35145<br>Dm_FBpp0072893<br>Sc	Protein-cysteine N-palmitoyltransferase HHAT (EC 2.3.1.-)(Hedgehog acyltransferase)(Skinny hedgehog protein 1)(Melanoma antigen recognized by T-cells 2)(MART-2)
31	1	CI	ENSP00000240055<br>ENSG00000120837	14<br>14<br>12<br>12<br>12<br>12<br>12<br>-<br>8<br>5	dgdssttdasQLGISADYiggshyviqp<br>dgdssttdasQLGISADYiggshyviqp<br>dgdssttdasQLGISADYiggshyviqp<br>dgdssttdasQLGISADYiggshyviqp<br>dgdssttdasQLGIAGDYiggshyviqp<br>dgdssttdtsQLGISGDYisgghyviqp<br>dgdssttdasqlgmtgeymasshfvlha<br>............................<br>---msnsedsqq-----yln--dmlvke<br>---mntne------------sehvstsp	Hs_ENSP00000240055<br>Bt_ENSBTAP00000041024<br>Rn_ENSRNOP00000014123<br>Mm_ENSMUSP00000100946<br>Gg_ENSGALP00000020733<br>Xt_ENSXETP00000042558<br>Dr_ENSDARP00000090543<br>Ce<br>Dm_FBpp0080827<br>Sc_YBL021C	NP_006157 Nuclear transcription factor Y subunit beta (Nuclear transcription factor Y subunit B)(NF-YB)(CAAT-box DNA-binding protein subunit B)
32	1	CI	ENSP00000292205<br>ENSG00000160710	1092<br>977<br>995	kilrwnvlglQGALLTHFlqpiylksvt<br>kilrwnvlglQGALLTHFlqpvylksvt<br>kilrwnvlglQGALLTHFlqpvylksvt	Hs_ENSP00000292205<br>Bt_ENSBTAP00000009896<br>Rn_ENSRNOP00000028181	NP_001020278 Double-stranded RNA-specific adenosine deaminase (DRADA)

				998<br>676<br>927<br>1165<br>-<br>-<br>-	kilrwnvlglQGALLTHFlqpvylksvt<br>kilrwnvlglQGALLSHFiepvylssvt<br>kilrwnvlglQGGLLTHFmepvylsslt<br>kilrwnvlglQGALLTHFihpiylhsit<br>............................<br>............................<br>............................	Mm_ENSMUSP00000103028<br>Gg_ENSGALP00000039924<br>Xt_ENSXETP00000008359<br>Dr_ENSDARP00000015422<br>Ce<br>Dm<br>Sc	(EC 3.5.4.-)(136 kDa double-stranded RNA-binding protein)(P136)(K88DSRBP)(Interferon-inducible protein 4)(IFI-4)
33	1	CI	ENSP00000327687<br>ENSG00000171189	741<br>687<br>743<br>687<br>695<br>-<br>740<br>-<br>-<br>-	alvrnsdegiQRVLTTDYallmestsie<br>alvknsdegihrvlttdyallmestsie<br>alvknsdegiQRVLTTDYallmestsie<br>alvknsdegiQRVLTTDYallmestsie<br>alvknndegiQRVLTTDYallmestsie<br>............................<br>alvknnregiQRVLTTDYallmestsie<br>............................<br>............................<br>............................	Hs_ENSP00000327687<br>Bt_ENSBTAP00000015562<br>Rn_ENSRNOP00000045594<br>Mm_ENSMUSP00000072107<br>Gg_ENSGALP00000025484<br>Xt<br>Dr_ENSDARP00000004703<br>Ce<br>Dm<br>Sc	Glutamate receptor, ionotropic kainate 1 Precursor (Glutamate receptor 5)(GluR-5)(GluR5)(Excitatory amino acid receptor 3)(EAA3)
34	1	CI	ENSP00000379017<br>ENSG00000141127	149<br>149<br>149<br>149<br>149<br>-<br>137<br>137<br>155<br>-	lthlitmdlhQKEIQGFFnipvdnlras<br>lthlitmdlhQKEIQGFFnipvdnlras<br>lthlitmdlhQKEIQGFFnipvdnlras<br>lthlitmdlhQKEIQGFFnipvdnlras<br>lthlitmdlhQKEIQGFFnipvdnlras<br>............................<br>lthlitmdlhQKEIQGFFnipvdnlras<br>asrlvsldlykkeiqgffscpvdnlras<br>lthiitmdlhQKEIQGFFdipvdnlras<br>............................	Hs_ENSP00000379017<br>Bt_ENSBTAP00000019786<br>Rn_ENSRNOP00000048818<br>Mm_ENSMUSP00000004955<br>Gg_ENSGALP00000008105<br>Xt<br>Dr_ENSDARP00000011444<br>Ce_CE03789<br>Dm_FBpp0085035<br>Sc	NP_002758 Phosphoribosyl pyrophosphate synthetase-associated protein 2 (PRPP synthetase-associated protein 2)(41 kDa phosphoribosypyrophosphate synthetase-associated protein)(PAP41)
35	1	CI	ENSP00000348314<br>ENSG00000152582	1605<br>1509<br>1511<br>875<br>1066<br>-<br>1450<br>-<br>-<br>-	npleplpfnrQEHLIEFFfr-lfadyek<br>npleplpfnrQEHLIEFFfr-lfadcek<br>npleplpfnrQEHLIEFFfr-lfadsek<br>----------------------------<br>sklknipvevkqflssffffslfadtgr<br>............................<br>dptepvpydrlanlkkfffs-lfasths<br>............................<br>............................<br>............................	Hs_ENSP00000348314<br>Bt_ENSBTAP00000009967<br>Rn_ENSRNOP00000023780<br>Mm_ENSMUSP00000035762<br>Gg_ENSGALP00000038362<br>Xt<br>Dr_ENSDARP00000062993<br>Ce<br>Dm<br>Sc	NP_079143 Sperm flagellar protein 2 (Protein KPL2)
36	1	CI	ENSP00000261588<br>ENSG00000047578	1359<br>1022<br>1007<br>1350<br>1293<br>1353<br>-<br>-<br>-<br>-	gpgnchfdfaQEILFVDYlraqllpqpa<br>----------------------------<br>----------------------------<br>gpgnchfdfaQEILFGDYlqtrlppapt<br>gpgnchfdfaQEILFVDYlqpqlinrlq<br>gpgnchfdfaQEILFVDYihekqitdtq<br>............................<br>............................<br>............................<br>............................	Hs_ENSP00000261588<br>Bt_ENSBTAP00000008064<br>Rn_ENSRNOP00000001880<br>Mm_ENSMUSP00000065744<br>Gg_ENSGALP00000028702<br>Xt_ENSXETP00000039706<br>Dr<br>Ce<br>Dm<br>Sc	NP_056017 Uncharacterized protein KIAA0556
37	1	C	ENSP00000379059<br>ENSG00000177427	425<br>416<br>414<br>414<br>310<br>419<br>422<br>-<br>-<br>-	alelligsleQASLPCHFnpsvnlfssl<br>alelllssleraslpshfnrsvnlldgl<br>aleflvgsleQASLPCHFnpsvnllgsf<br>aleflvgsleQASLPCHFnpsvnllgnf<br>vleelvgylengvlpcyfnpkvnlfcel<br>vleeligyldkgflpsyfndklnlfsal<br>aiteligyletgvlpsyfkanvnlfqgf<br>............................<br>............................<br>............................	Hs_ENSP00000379059<br>Bt_ENSBTAP00000022714<br>Rn_ENSRNOP00000023838<br>Mm_ENSMUSP00000018743<br>Gg_ENSGALP00000008000<br>Xt_ENSXETP00000012698<br>Dr_ENSDARP00000075593<br>Ce<br>Dm<br>Sc	NP_631901 Smith-Magenis syndrome chromosome region candidate gene 7 protein
38	1	CI	ENSP00000313513<br>ENSG00000151687	74<br>74<br>145<br>145<br>-<br>141<br>-<br>-<br>-<br>-	ppayydtrigQILINIDYmlkalwhgiy<br>ppayfdtrigQILINIDYmlkalwhgiy<br>ppayydtrvgQILIHIDYmlkalwhgiy<br>ppayydtrvgQILIQIDYmlkalwhgiy<br>............................<br>ppaycdtrvgQIMIAVDYmmkalwhgaf<br>............................<br>............................<br>............................<br>............................	Hs_ENSP00000313513<br>Bt_ENSBTAP00000016667<br>Rn_ENSRNOP00000005314<br>Mm_ENSMUSP00000054056<br>Gg<br>Xt_ENSXETP00000021756<br>Dr<br>Ce<br>Dm<br>Sc	NP_653309 Ankyrin and armadillo repeat-containing protein
39	1	CI	ENSP00000265689<br>ENSG00000110721	324<br>208<br>320	llegrensekQKLMLIDFeyssynyrgf<br>lldgrensekQKLMLIDFeyssynyrgf<br>llegqensekQKLMLIDFeyssynyrgf	Hs_ENSP00000265689<br>Bt_ENSBTAP00000013607<br>Rn_ENSRNOP00000023020	NP_001268 Choline kinase alpha (CK)(EC 2.7.1.32)(CHETK-alpha)

				320<br>319<br>288<br>285<br>-<br>-<br>-	llegqenserrklmlidfeyssynyrgf<br>llegrensekQKLMLIDFeyssynyrgf<br>lldgrensekQKLMLIDFeyssynyrgf<br>llngrentdrQRLMLIDFeyssynyrgf<br>............................<br>............................<br>............................	Mm_ENSMUSP00000025760<br>Gg_ENSGALP00000011197<br>Xt_ENSXETP00000022088<br>Dr_ENSDARP00000060183<br>Ce<br>Dm<br>Sc	
40	1	CI	ENSP00000386082<br>ENSG00000084710	342<br>354<br>225<br>367<br>347<br>401<br>339<br>-<br>373<br>-	vlemfntllrQLRLSIDYaltgsydgav<br>vlemfntllrQLRLSIDYvltgsydgai<br>vlemfntllrQLRLSIDYaltgsydgav<br>vlemfntllrQLRLSIDYaltgsydgav<br>vlevfntllrQLRLSIDYaltgsyncit<br>vlevfntllrQLRLSIDYeltgtydstv<br>vlevfntllrhlrlsvdyeltgsydctn<br>............................<br>aldiinnllthlrtsvs-----------<br>............................	Hs_ENSP00000386082<br>Bt_ENSBTAP00000032868<br>Rn_ENSRNOP00000030729<br>Mm_ENSMUSP00000106809<br>Gg_ENSGALP00000026747<br>Xt_ENSXETP00000038047<br>Dr_ENSDARP00000091534<br>Ce<br>Dm_FBpp0100147<br>Sc	NP_055786 Protein EFR3 homolog B
41	1	C	ENSP00000382394<br>ENSG00000091656	2154<br>935<br>2149<br>2141<br>2118<br>-<br>-<br>-<br>2096<br>-	qemaeksglsqkvikhwfrntlfkerqr<br>qemaeksglsqkvikhwfrntlfkerqr<br>qemaeksglsqkvikhwfrntlfkerqr<br>qemaeksglsqkvikhwfrntlfkerqr<br>qemaeksglsqkvikhwfrntlfkerqr<br>............................<br>............................<br>............................<br>nvygsmndllnqqlenmgsnmgppkkmq<br>............................	Hs_ENSP00000382394<br>Bt_ENSBTAP00000027021<br>Rn_ENSRNOP00000011641<br>Mm_ENSMUSP00000026284<br>Gg_ENSGALP00000025229<br>Xt<br>Dr<br>Ce<br>Dm_FBpp0088139<br>Sc	Zinc finger homeobox protein 4 (Zinc finger homeodomain protein 4)(ZFH-4)
41	2	C	ENSP00000382394<br>ENSG00000091656	2221<br>1002<br>2216<br>2208<br>2185<br>-<br>-<br>-<br>2165<br>-	rssrtrftdyqlrvlqdffdtnaypkdd<br>rssrtrftdyqlrvlqdffdtnaypkdd<br>rssrtrftdyqlrvlqdffdtnaypkdd<br>rssrtrftdyqlrvlqdffdtnaypkdd<br>rssrtrftdyqlrvlqdffdtnaypkdd<br>............................<br>............................<br>............................<br>ranrtrftdyqikvlqeffennsypkds<br>............................	Hs_ENSP00000382394<br>Bt_ENSBTAP00000027021<br>Rn_ENSRNOP00000011641<br>Mm_ENSMUSP00000026284<br>Gg_ENSGALP00000025229<br>Xt<br>Dr<br>Ce<br>Dm_FBpp0088139<br>Sc	Zinc finger homeobox protein 4 (Zinc finger homeodomain protein 4)(ZFH-4)
41	3	CI	ENSP00000382394<br>ENSG00000091656	2709<br>1489<br>2701<br>2693<br>2673<br>-<br>-<br>-<br>2589<br>-	istedggespQKYIYFDYpslpltkidl<br>isaedggdspQKYIYFDYpslpltkidl<br>istedggespQKYIYFDYpslpltkidl<br>istedggespQKYIYFDYpslpltkidl<br>istedggdspQKYIFFDYpslslaktel<br>............................<br>............................<br>............................<br>iipdvpgykps------------lripk<br>............................	Hs_ENSP00000382394<br>Bt_ENSBTAP00000027021<br>Rn_ENSRNOP00000011641<br>Mm_ENSMUSP00000026284<br>Gg_ENSGALP00000025229<br>Xt<br>Dr<br>Ce<br>Dm_FBpp0088139<br>Sc	Zinc finger homeobox protein 4 (Zinc finger homeodomain protein 4)(ZFH-4)
42	1	C	ENSP00000359403<br>ENSG00000102181	218<br>-<br>193<br>194<br>119<br>-<br>211<br>-<br>-<br>-	yqqkkfcfsiQQGLNADYvkgenleavv<br>............................<br>yqhrkfcfsvqrgldaayvkgenleavv<br>yqqkkfcfsiQQGLNADYvkgenleavv<br>yqqkkfcfsiqqglnaeyvkgenmeavv<br>............................<br>yqkkklcfsiqqslnadmvkadapdavv<br>............................<br>............................<br>............................	Hs_ENSP00000359403<br>Bt<br>Rn_ENSRNOP00000024427<br>Mm_ENSMUSP00000042606<br>Gg_ENSGALP00000014751<br>Xt<br>Dr_ENSDARP00000073701<br>Ce<br>Dm<br>Sc	NP_113650 CD99 antigen-like protein 2 Precursor (MIC2-like protein 1)(CD99 antigen)
43	1	CI	ENSP00000359489<br>ENSG00000155966	190<br>0<br>174<br>190<br>0<br>155<br>0<br>-<br>248<br>-	kmqtltqdqsQAKLEDFFvypaeqpqig<br>----------------------------<br>kiqtstqdppQTRLEDFFvypaeqpqig<br>kmqtstqdppQTRLEDFFvypaeqpqig<br>----------------------------<br>----------------------------<br>----------------------------<br>............................<br>nassggvpastplgpplstqmpngreks<br>............................	Hs_ENSP00000359489<br>Bt_ENSBTAP00000027656<br>Rn_ENSRNOP00000041594<br>Mm_ENSMUSP00000033532<br>Gg_ENSGALP00000014918<br>Xt_ENSXETP00000042497<br>Dr_ENSDARP00000068559<br>Ce<br>Dm_FBpp0077319<br>Sc	NP_002016 AF4/FMR2 family member 2 (Fragile X mental retardation 2 protein)(Protein FMR-2)(FMR2P)(Protein Ox19)(Fragile X E mental retardation syndrome protein)
44	1	CI	ENSP00000349087<br>ENSG00000182087	439<br>377<br>430	lalvtswlfiQHSMIYFFhhyelpailq<br>lalvtswlfiQHSMIYFFhhyelpailq<br>lalvtswlfiQHSMIYFFhhyelpailq	Hs_ENSP00000349087<br>Bt_ENSBTAP00000015085<br>Rn_ENSRNOP00000017245	NP_001028198 Membralin

				454	lalvtswlfiQHSMIYFFhhyelpailq	Mm_ENSMUSP00000101014	
				438	lalvtswlfiQHSMIYFFhhyelpailr	Gg_ENSGALP00000003149	
				428	lalvtswlfiQHSMIYFFhhyelpailq	Xt_ENSXETP00000046683	
				420	lalvtswlfiQHSMIYFFhhyelpailq	Dr_ENSDARP00000079593	
				513	lalltssmfilhsmiyffhhyemplily	Ce_CE41955	
				453	lallssylftQHSMVFFFhryelpaima	Dm_FBpp0086352	
				-	....	Sc	
45	1	CI	ENSP00000335321 ENSG00000115524	1277	kiynsiyigsQDALIAHYpriynddknt	Hs_ENSP00000335321	NP_036565 Splicing factor 3B subunit 1 (Pre-mRNA-splicing factor SF3b 155 kDa subunit)(SF3b155)(Spliceosome-associated protein 155)(SAP 155)
				1278	kiynsiyigsQDALIAHYpriynddknt	Bt_ENSBTAP00000011734	
				1277	kiynsiyigsQDALIAHYpriynddknt	Rn_ENSRNOP00000019126	
				1277	kiynsiyigsQDALIAHYpriynddknt	Mm_ENSMUSP00000027127	
				1274	kiynsiyigsQDALIAHYpriyndekn t	Gg_ENSGALP00000013029	
				1267	kiynsiyigsQDALIAHYpriyndeknt	Xt_ENSXETP00000029260	
				1288	kiynsiyigsqdal--------------	Dr_ENSDARP00000073029	
				1295	kvfnnlilgsadaliaayprientptnq	Ce_CE03641	
				1313	kiynslyiggqdaliagypritndpknq	Dm_FBpp0077728	
				946	rvynnmyvmyQDAMVPFYp--vtpdnne	Sc_YMR288W	
46	1	CI	ENSP00000277010 ENSG00000147955	194	faladtvfstQDFLTLFYtlrsyarglr	Hs_ENSP00000277010	NP_005857 Sigma 1-type opioid receptor (Sigma1-receptor)(Sigma1R)(SIG-1R)(hSigmaR1)(SR31747-binding protein)(SR-BP)(Aging-associated gene 8 protein)
				194	faladtvfstQDFLTLFYtlrayarglr	Bt_ENSBTAP00000020990	
				194	falsdtifstQDFLTLFYtlrayarglr	Rn_ENSRNOP00000019795	
				194	faladtffstQDYLTLFYtlrayarglr	Mm_ENSMUSP00000056027	
				-	....	Gg	
				191	faladtifstQDFLTLFYtvkvygkall	Xt_ENSXETP00000034034	
				192	faladtifstQDFLTLFYtarvyvkgmi	Dr_ENSDARP00000011354	
				196	islgeplsviqfymkqaynywnnlshtl	Ce_CE14716	
				-	....	Dm	
				196	fgfldtfsstldlytlyrtvyltar---	Sc_YMR202W	
47	1	C	ENSP00000366477 ENSG00000204741	26	gdhfdwirssQSELSADFehqapprdhs	Hs_ENSP00000366477	Novel MAM domain containing protein Fragment
				26	gddfewtwssrsnlsaefedqappwdht	Bt_ENSBTAP00000020938	
				26	gdhfdwvwssqsdlpaeleqqaptrdht	Rn_ENSRNOP00000049534	
				37	eddldwsigs----iipteglsrdsdht	Mm_ENSMUSP00000097957	
				26	adgfdwirssssslepdfqqqappqdht	Gg_ENSGALP00000013830	
				26	sdsfewmrqsrinvppqyeqqapprdht	Xt_ENSXETP00000036184	
				-	....	Dr	
				-	....	Ce	
				-	....	Dm	
				-	....	Sc	
48	1	CI	ENSP00000329471 ENSG00000105438	187	dliaivaglvQTVLYCDFfylyitkvlk	Hs_ENSP00000329471	NP_006792 ER lumen protein retaining receptor 1 (KDEL endoplasmic reticulum protein retention receptor 1)(KDEL receptor 1)(Putative MAPK-activating protein PM23)
				189	dliaivaglvQTVLYCDFfylyitkvlk	Bt_ENSBTAP00000005857	
				187	dliaivaglvQTVLYCDFfylyitkvlk	Rn_ENSRNOP00000028627	
				187	dliaivaglvQTVLYCDFfylyitkvlk	Mm_ENSMUSP00000002855	
				-	....	Gg	
				187	dliaivaglvQTVLYCDFfylyitkvlk	Xt_ENSXETP00000023269	
				-	....	Dr	
				187	dpisvvagivQTVLYADFfylyitrviq	Ce_CE23635	
				187	dliaifagvvQTVLYCDFfylyitkvlk	Dm_FBpp0079612	
				194	dkiaffagllQTLLYSDFfyiyytkvir	Sc_YBL040C	
49	1	C	ENSP00000387188 ENSG00000105877	2453	kemkavkfpsqgtifdyyvdhktkkllp	Hs_ENSP00000387188	Dynein heavy chain 11, axonemal (Axonemal beta dynein heavy chain 11)(Ciliary dynein heavy chain 11)
				-	....	Bt	
				2415	kemkavkfpsqgtifdyyldhktkkflp	Rn_ENSRNOP00000007233	
				2418	kemkavkfpsqgtifdyyldhktkkflp	Mm_ENSMUSP00000081867	
				-	....	Gg	
				2387	kemrtikfpshgtvfdyyidpdtkrftp	Xt_ENSXETP00000016372	
				2411	kemravkfpsqgsvfdyyidpntkrftp	Dr_ENSDARP00000041556	
				-	....	Ce	
				-	....	Dm	
				-	....	Sc	
49	2	C	ENSP00000387188 ENSG00000105877	2789	egidshmllqQPLIYCHFadrgkdphym	Hs_ENSP00000387188	Dynein heavy chain 11, axonemal (Axonemal beta dynein heavy chain 11)(Ciliary dynein heavy chain 11)
				-	....	Bt	
				2751	kgadantlqqqplvychfasgredpcye	Rn_ENSRNOP00000007233	
				2754	kgvdanallrqplvychfasggedpcym	Mm_ENSMUSP00000081867	
				-	....	Gg	
				2723	egidedallqmplvyshfahgigepryf	Xt_ENSXETP00000016372	
				2747	egidesifinQPLIYSHFahgvgeprya	Dr_ENSDARP00000041556	
				-	....	Ce	
				-	....	Dm	
				-	....	Sc	
50	1	CI	ENSP00000003302 ENSG00000048028	826	tptshsdprlQHVLVYFFqneapkrvve	Hs_ENSP00000003302	NP_065937 Ubiquitin carboxyl-terminal hydrolase 28 (EC 3.1.2.15)(Ubiquitin thioesterase
				830	tptshsdprlQHVLIYFFqneapkrvve	Bt_ENSBTAP00000002998	
				800	tptshsdprlQHVLVYFFqneapkrvve	Rn_ENSRNOP00000009724	

				800<br>795<br>669<br>806<br>-<br>-<br>-	tptshsdprlQHVLVYFFqneapkrvve<br>tptpqndarlqhvlvyflqndapqqive<br>epspssdprlQHVLIYFFqnkapdrvie<br>ettpqqdprlqhvlvylfqnkapervie<br>............................<br>............................<br>............................	Mm_ENSMUSP00000047467<br>Gg_ENSGALP00000011273<br>Xt_ENSXETP00000049661<br>Dr_ENSDARP00000012821<br>Ce<br>Dm<br>Sc	28)(Ubiquitin-specific-processing protease 28)(Deubiquitinating enzyme 28)
51	1	CI	ENSP00000267803<br>ENSG00000140254	172<br>172<br>172<br>172<br>170<br>170<br>-<br>181<br>180<br>-	p-rspcglyrQYRLAGHYtsamlwvafl<br>p-hspcglhgQYRLAGHYtsamlwvafl<br>p-rspcglynQYRLAGHYasamlwvafl<br>p-rspcglynQYRLAGHYasamlwvafl<br>t-qspcnvhrqyrisscyasitlwmalc<br>i-nspcglfqqycistyysseimwvafg<br>............................<br>lnqdsfdwgrhyrvaghythaaiwfafa<br>lgregfswggqyraagyfasimlwasla<br>............................	Hs_ENSP00000267803<br>Bt_ENSBTAP00000035724<br>Rn_ENSRNOP00000024246<br>Mm_ENSMUSP00000106166<br>Gg_ENSGALP00000003862<br>Xt_ENSXETP00000051522<br>Dr<br>Ce_CE30481<br>Dm_FBpp0080238<br>Sc	NP_653166 Dual oxidase maturation factor 1 (Dual oxidase activator 1)(Numb-interacting protein)
52	1	CI	ENSP00000377197<br>ENSG00000157423	4208<br>4196<br>-<br>4242<br>4151<br>-<br>-<br>-<br>-<br>-	tgsitlltpnQTNIINFYevelnecvqc<br>ngsvtlltpnQTTIVNFYevelnecvqc<br>............................<br>mgttilltstqvntinfyevelnecvqc<br>nggvtelsaqevnvidfkevqlneraqr<br>............................<br>............................<br>............................<br>............................<br>............................	Hs_ENSP00000377197<br>Bt_ENSBTAP00000005218<br>Rn<br>Mm_ENSMUSP00000046204<br>Gg_ENSGALP00000003781<br>Xt<br>Dr<br>Ce<br>Dm<br>Sc	NP_116210 Hydrocephalus-inducing protein homolog
53	1	CI	ENSP00000298139<br>ENSG00000165392	144<br>141<br>145<br>138<br>109<br>138<br>109<br>17<br>-<br>-	kkagvgiegdQWKLLRDFdiklknfvel<br>kkagvgieqdQWKLLRDFdinlksivel<br>rkagvgiegdQWKLLRDFdvklesfvel<br>kkagvgiegdQWKLLRDFdvklesfvel<br>kkvgvgiegdhwklmgdfevklksfvel<br>kkvgvgiegdQWKLMSDYelklkgfiel<br>mkvgvgiegdkwkllsdydiklknivdl<br>pstrpgsvn------------eelpet<br>............................<br>............................	Hs_ENSP00000298139<br>Bt_ENSBTAP00000028778<br>Rn_ENSRNOP00000020835<br>Mm_ENSMUSP00000033990<br>Gg_ENSGALP00000016701<br>Xt_ENSXETP00000039653<br>Dr_ENSDARP00000069476<br>Ce_CE31791<br>Dm<br>Sc	NP_000544 Werner syndrome ATP-dependent helicase (EC 3.6.1.-)
54	1	CI	ENSP00000301295<br>ENSG00000160505	512<br>-<br>510<br>510<br>-<br>-<br>-<br>-<br>-<br>-	fltgllnkkeQEKLDAFFgfqlsqeikq<br>............................<br>flfgllheseQEKLEMFFghqlsqeikh<br>fifgllheseQKKLEAFFghqlsqeikr<br>............................<br>............................<br>............................<br>............................<br>............................<br>............................	Hs_ENSP00000301295<br>Bt<br>Rn_ENSRNOP00000046459<br>Mm_ENSMUSP00000046503<br>Gg<br>Xt<br>Dr<br>Ce<br>Dm<br>Sc	NP_604393 NACHT, LRR and PYD domains-containing protein 4 (PYRIN-containing APAF1-like protein 4)(PAAD and NACHT-containing protein 2)(PYRIN and NACHT-containing protein 2)(Ribonuclease inhibitor 2)(Cancer/testis antigen 58)(CT58)
55	1	C	ENSP00000278935<br>ENSG00000110274	11<br>12<br>11<br>11<br>0<br>-<br>-<br>-<br>36<br>-	magrplrigdQLVLEEDYdetyipseqe<br>magrpirigdQLVLEEDYdenyipsehe<br>msrrpillgdqlvleedsdetyvpseqe<br>marrpillgdqlvleedsdetyvpseqe<br>----------------------------<br>............................<br>............................<br>............................<br>tpssssqgsssviceevfdeacfpssee<br>............................	Hs_ENSP00000278935<br>Bt_ENSBTAP00000018792<br>Rn_ENSRNOP00000022583<br>Mm_ENSMUSP00000110275<br>Gg_ENSGALP00000011817<br>Xt<br>Dr<br>Ce<br>Dm_FBpp0073952<br>Sc	NP_055771 Centrosomal protein of 164 kDa (Cep164)
56	1	C	ENSP00000270776<br>ENSG00000142657	418<br>416<br>-<br>387<br>387<br>388<br>415<br>388<br>385<br>386	kdafdrnpelQNLLLDDFfksavencqd<br>kdafdrnpglQNLLLDDFfksavencqd<br>............................<br>kdafernpelQNLLLDDFfksavdncqd<br>kdafdrnpelQNLLLDDFfktavekcqd<br>keafdrnpelqnllldnffksemercqe<br>keafdrnpelqsllldsffskavqdcqd<br>ehafqknkqlsnllldddfftkaiteaqd<br>kdaytsqpelsnllldffkkaiergqd<br>tkayreepdlenllfnkffadavtkaqs	Hs_ENSP00000270776<br>Bt_ENSBTAP00000017988<br>Rn<br>Mm_ENSMUSP00000081141<br>Gg_ENSGALP00000004417<br>Xt_ENSXETP00000047969<br>Dr_ENSDARP00000007588<br>Ce_CE06508<br>Dm_FBpp0070368<br>Sc_YHR183W	NP_002622 6-phosphogluconate dehydrogenase, decarboxylating (EC 1.1.1.44)
57	1	C	ENSP00000357955	186	vlctgkagfsQRGIRLHYknsifhrivq	Hs_ENSP00000357955	NP_775943 Peptidyl-prolyl cis-

			ENSG00000185250	182	ilctgkagfsQSGIKLHYtgsifhrvvr	Bt_ENSBTAP00000010705	trans isomerase-like 6 (PPIase)(Rotamase)(EC 5.2.1.8)(Cyclophilin-like protein PPIL6)
				-	....	Rn	
				188	vlctgtsgfsergtklhykdsifhrvvq	Mm_ENSMUSP00000101146	
				165	alceggvmspssgqeltyknscfhrlvk	Gg_ENSGALP00000024541	
				179	slctgaagvslsglklhykdsifhrivk	Xt_ENSXETP00000046061	
				181	alctgeaglsksnlelsykgsifhrvvp	Dr_ENSDARP00000061788	
				-	....	Ce	
				-	....	Dm	
				-	....	Sc	
58	1	CI	ENSP00000264883 ENSG00000138750	437	fkgrlnelmsQIRMQNHFgavrseeryy	Hs_ENSP00000264883	NP_059122 Nucleoporin p54 (54 kDa nucleoporin)
				418	fkgrlnelmsQIRMQNHFgavrseeryy	Bt_ENSBTAP00000052050	
				440	fkgrlnelmsQIRMQNHFgavkseekyy	Rn_ENSRNOP00000003070	
				440	fkgrlnelmsQIRMQNHFgavkseekyy	Mm_ENSMUSP00000046540	
				431	frgrlnelmsQIRMQNHFgavraeeryy	Gg_ENSGALP00000018737	
				466	fkgrlnelmsQIRMQNHFgavrseekyy	Xt_ENSXETP00000052045	
				252	----------------------------	Dr_ENSDARP00000066691	
				579	vkfyvdkfyeileskpdklqesmwkmfd	Ce_CE23858	
				546	fkgrlsellsqmrmqrnqfaanggaeya	Dm_FBpp0087063	
				469	lwarlailkeraknissqldsklmvfnd	Sc_YGR119C	
59	1	CI	ENSP00000370424 ENSG00000110651	126	lgcygaiqesQCLLGTFFtclvilface	Hs_ENSP00000370424	CD81 antigen (26 kDa cell surface protein TAPA-1)(Target of the antiproliferative antibody 1)(Tetraspanin-28)(Tspan-28)(CD81 antigen)
				-	....	Bt	
				88	lgcygaiqesQCLLGTFFtclvilface	Rn_ENSRNOP00000027760	
				88	lgcygaiqesQCLLGTFFtclvilface	Mm_ENSMUSP00000043768	
				90	lgcygaiqesQCLLGTFFtclvilface	Gg_ENSGALP00000038498	
				88	lgcygaiqesQCLLGTFFaclvilface	Xt_ENSXETP00000052457	
				88	lgcygaiqesQCLLGTFFtclvilface	Dr_ENSDARP00000036831	
				-	....	Ce	
				-	....	Dm	
				-	....	Sc	
60	1	CI	ENSP00000326477 ENSG00000136169	166	ln-lkgenplQLPIKCHFqrrhaktnsh	Hs_ENSP00000326477	NP_114121 Histone-lysine N-methyltransferase SETDB2 (EC 2.1.1.43)(SET domain bifurcated 2)(Chronic lymphocytic leukemia deletion region gene 8 protein)(Lysine N-methyltransferase 1F)
				164	ft-fkgenplQLPIKCHFqrrhaktnsh	Bt_ENSBTAP00000009641	
				164	ls-lkgenplQLPLRCHFqrrhaktnsp	Rn_ENSRNOP00000036240	
				170	ls-lkgenplQLPIRCHFqrrhaktnsh	Mm_ENSMUSP00000093450	
				165	vssykgenplkipilfhfqrrhakadcl	Gg_ENSGALP00000035697	
				180	psllkkenplnlpvscdfqrwrvktngs	Xt_ENSXETP00000034176	
				-	....	Dr	
				0	----------------------------	Ce_CE27623	
				-	....	Dm	
				-	....	Sc	
61	1	CI	ENSP00000227471 ENSG00000110057	83	ltygvylgllQMQLILHYdetyrevkyg	Hs_ENSP00000227471	NP_112192 Protein unc-93 homolog B1 (Protein UNC-93B)(hUNC93B1)
				83	ltygvylgllQMQLILHYdetyrevkyg	Bt_ENSBTAP00000001581	
				83	ltygvylgllQMQLILHYdetyrevkyg	Rn_ENSRNOP00000024018	
				83	ltygvylgllQMQLILHYdetyrevkyg	Mm_ENSMUSP00000040093	
				58	ltysvylgllQMQLILHYdetyrevkys	Gg_ENSGALP00000005384	
				51	ltygvylgllQMQLILHYdetyrdvkys	Xt_ENSXETP00000045093	
				4	-------gllQMQLILHYdetyrevkyg	Dr_ENSDARP00000077600	
				-	....	Ce	
				-	....	Dm	
				-	....	Sc	
62	1	CI	ENSP00000285968 ENSG00000155561	930	lcciscnsniQIKLVGDFthdqsisqkl	Hs_ENSP00000285968	NP_055950 Nuclear pore complex protein Nup205 (Nucleoporin Nup205)(205 kDa nucleoporin)
				931	lcciscnsniQIKLVGDFthdqsvsqkl	Bt_ENSBTAP00000042092	
				923	lcciscnsniQIKMVGDFthdqnvsqkl	Rn_ENSRNOP00000014833	
				989	lcciscnsniQVKMVGDFthdqsvsqkl	Mm_ENSMUSP00000039656	
				930	lcciscnsniQIKLVGDFthdqstsqkl	Gg_ENSGALP00000019040	
				927	lcsiacnskiQEKIVGDFtqdqnvtqkl	Xt_ENSXETP00000023244	
				936	lrritrypniQARLVGDFthdqavserl	Dr_ENSDARP00000062359	
				904	lrdvmatrgaaevkmlellrsrnsaash	Ce_CE02271	
				931	lasvtqlpnvstqilsmygqgsneklei	Dm_FBpp0074567	
				941	gvddqilatnslrilaklsersngsvas	Sc_YJL039C	
62	2	C	ENSP00000285968 ENSG00000155561	1800	vnrdgprqdtqapvvpywrlpglgiiiy	Hs_ENSP00000285968	NP_055950 Nuclear pore complex protein Nup205 (Nucleoporin Nup205)(205 kDa nucleoporin)
				1805	vnrdgprqdtqvpvvpywhlpglgiivy	Bt_ENSBTAP00000042092	
				1793	vnrdgarqdtqvpvvpywrlpglgiiiy	Rn_ENSRNOP00000014833	
				1860	vnrdgarqdtqvpvvpywrlpglgiiiy	Mm_ENSMUSP00000039656	
				1804	tnrdgprqdtqapvvpywrlpglgiiiy	Gg_ENSGALP00000019040	
				1801	ssrdgtrqdsqvsilpswrlpglgivih	Xt_ENSXETP00000023244	
				1779	advs----------ipsarvpslglvll	Dr_ENSDARP00000062359	
				1666	------------------------tvks	Ce_CE02271	
				1903	tlltg-----------kmsskklttimh	Dm_FBpp0074567	
				1662	--------------------------ln	Sc_YJL039C	
63	1	CI	ENSP00000254035	219	klsemteqdqQRLIDDHFlfdkpvspll	Hs_ENSP00000254035	NP_001093205 Creatine kinase,

			ENSG00000131730	219<br>-<br>219<br>219<br>-<br>218<br>-<br>-<br>-	klsemteqdqQRLIDDHFlfdkpvspll<br>............................<br>klsemteqdqQRLIDDHFlfdkpvspll<br>sltnmserdqQQLIDDHFlfdkpvspll<br>............................<br>sltemtdheqQRLIDDHFlfdkpvspll<br>............................<br>............................<br>............................	Bt_ENSBTAP00000001330<br>Rn<br>Mm_ENSMUSP00000022122<br>Gg_ENSGALP00000025110<br>Xt<br>Dr_ENSDARP00000092223<br>Ce<br>Dm<br>Sc	sarcomeric mitochondrial Precursor (EC 2.7.3.2)(S-MtCK)(Basic-type mitochondrial creatine kinase) (Mib-CK)
64	1	CI	ENSP00000285928<br>ENSG00000155530	180<br>186<br>179<br>180<br>78<br>-<br>45<br>-<br>-<br>-	mpyllelnasQNNLTTFFnfkppknlkk<br>mpyllelnasQNHLKTFFnfkppkklkk<br>mpyllelnasQNRLTTFFnfkppqnlkk<br>mpyllelnasQNKLTTFFnfkppqnlkk<br>mpyllelnasnnelttyfgfkppknlke<br>............................<br>mpylitldashnqltdffgfqppknlke<br>............................<br>............................<br>............................	Hs_ENSP00000285928<br>Bt_ENSBTAP00000020944<br>Rn_ENSRNOP00000011692<br>Mm_ENSMUSP00000065146<br>Gg_ENSGALP00000013784<br>Xt<br>Dr_ENSDARP00000092387<br>Ce<br>Dm<br>Sc	NP_653249 Leucine-rich repeats and guanylate kinase domain-containing protein
65	1	CI	ENSP00000254816<br>ENSG00000132481	594<br>605<br>598<br>598<br>-<br>-<br>-<br>-<br>-<br>-	gipaspidpfQSRLDSHFaglfthrlkp<br>gtpaspvdpfQSRLDSHFaglftrrlkp<br>galasptdpfQSRLDSHFsglfnyrlkp<br>galasptdpfQSRLDSHFsglfnhrlkp<br>............................<br>............................<br>............................<br>............................<br>............................<br>............................	Hs_ENSP00000254816<br>Bt_ENSBTAP00000025190<br>Rn_ENSRNOP00000010887<br>Mm_ENSMUSP00000021120<br>Gg<br>Xt<br>Dr<br>Ce<br>Dm<br>Sc	NP_258411 Tripartite motif-containing protein 47 (Gene overexpressed in astrocytoma protein)(RING finger protein 100)
66	1	C	ENSP00000354913<br>ENSG00000082269	1036<br>688<br>1039<br>1031<br>1032<br>881<br>644<br>623<br>1008<br>-	pfsastdivkqglvenyfgsqsstdisd<br>afsaspdmvkqglvenyfgcqsstdvsd<br>slssstdvvkqglvedyfgsqsntdvsd<br>---sstdvvkqglvenyfgsqsttdvsd<br>sptsstdivkqglvenyfgsrsstdisd<br>ssnsssdmvkqglvenyfgsqsstdisd<br>-vtsstdmvkkgfvenyfgsrsstdise<br>----------------------------<br>lalvlappppdefrdpppepapaapiap<br>............................	Hs_ENSP00000354913<br>Bt_ENSBTAP00000038854<br>Rn_ENSRNOP00000018234<br>Mm_ENSMUSP00000027337<br>Gg_ENSGALP00000025684<br>Xt_ENSXETP00000038829<br>Dr_ENSDARP00000087167<br>Ce_CE34256<br>Dm_FBpp0072556<br>Sc	Protein FAM135A
67	1	C	ENSP00000350447<br>ENSG00000163535	201<br>200<br>203<br>201<br>-<br>-<br>-<br>-<br>-<br>-	sgsttqplstqdnsevlflkennqnvyg<br>lvs-arpvstqnnlrllfvkendqnvcs<br>svsrqslslhqcnlevflpqednqktce<br>svsrqpsslhqcnlkafppkednqktcg<br>............................<br>............................<br>............................<br>............................<br>............................<br>............................	Hs_ENSP00000350447<br>Bt_ENSBTAP00000042237<br>Rn_ENSRNOP00000037361<br>Mm_ENSMUSP00000027202<br>Gg<br>Xt<br>Dr<br>Ce<br>Dm<br>Sc	NP_689737 Shugoshin-like 2 (Tripin)
67	2	CI	ENSP00000350447<br>ENSG00000163535	955<br>937<br>854<br>853<br>-<br>-<br>-<br>-<br>-<br>-	vnkskqklecQDIINKHYmevnsnekes<br>in--kqrpegq-avsgycmeinsdeken<br>--------hcQDVMSCDFgkktsneeen<br>--------dcedikscdfgeinsnkken<br>............................<br>............................<br>............................<br>............................<br>............................<br>............................	Hs_ENSP00000350447<br>Bt_ENSBTAP00000042237<br>Rn_ENSRNOP00000037361<br>Mm_ENSMUSP00000027202<br>Gg<br>Xt<br>Dr<br>Ce<br>Dm<br>Sc	NP_689737 Shugoshin-like 2 (Tripin)
68	1	CI	ENSP00000234420<br>ENSG00000116062	4<br>4<br>4<br>4<br>4<br>4<br>4<br>5<br>0<br>7	-------msrQSTLYSFFpkspalsdan<br>-------msrQSTLFSFFpkspavnnan<br>-------msrQSTLYSFFpkspalgdtk<br>-------msrQSTLYSFFpkspalgdtk<br>-------msrQSTLLRFFpkaqqpraaa<br>-------mskQKTLFSFFhksp-pisss<br>-------makQSSLFNFFsksppla vka<br>------mskrQSSLMSFFtktp------<br>----------------------------<br>----mapatpktsktahfengstssq--	Hs_ENSP00000234420<br>Bt_ENSBTAP00000001867<br>Rn_ENSRNOP00000021923<br>Mm_ENSMUSP00000005503<br>Gg_ENSGALP00000014551<br>Xt_ENSXETP00000049000<br>Dr_ENSDARP00000017721<br>Ce_CE28985<br>Dm_FBpp0075399<br>Sc_YDR097C	NP_000170 DNA mismatch repair protein Msh6 (MutS-alpha 160 kDa subunit)(G/T mismatch-binding protein) (GTMBP)(GTBP)(p160)
69	1	C	ENSP00000244007	422	siedhcsiaqqrnmaqyfkkvlgdtllt	Hs_ENSP00000244007	NP_002651 1-

			ENSG00000124181	422<br>422<br>422<br>352<br>217<br>418<br>461<br>426<br>157	siedhcsiaqqrnmaqyfkkvlgdtllt<br>siedhcsiaqQRNMAQHFrkvlgdtllt<br>siedhcsiaqQRNMAQHFrkvlgdtllt<br>siedhcsiaqqrnmaqnfkkvfgdmllt<br>tvlshnarxqQRNMAQHFkkvfgdmllt<br>siedhcsivqQRNMATFFkkvfgemllt<br>siedncsvpaqrllaqelkdilgdyllt<br>sieqncsleqqrnmaqalievfgdmllt<br>eldsvkdirigdtastyqeevdpkrlrs	Bt_ENSBTAP00000023383<br>Rn_ENSRNOP00000021872<br>Mm_ENSMUSP00000099404<br>Gg_ENSGALP00000005944<br>Xt_ENSXETP00000042118<br>Dr_ENSDARP00000073528<br>Ce_CE42905<br>Dm_FBpp0074009<br>Sc_YPL268W	phosphatidylinositol-4,5-bisphosphate phosphodiesterase gamma-1 (EC 3.1.4.11)(Phosphoinositide phospholipase C)(Phospholipase C-gamma-1)(PLC-gamma-1)(PLC-II)(PLC-148)
69	2	CI	ENSP00000244007<br>ENSG00000124181	1265<br>1265<br>1264<br>1264<br>1193<br>1012<br>1287<br>1324<br>1236<br>869	qqpfedfrisQEHLADHFdsrerraprr<br>qqpfedfrisQEHLADHFdgrdrrtprr<br>qqpfedfrisQEHLADHFdsrerrtamg<br>qqpfedfrisQEHLADHFdsrerstsdg<br>qqpfedfrvsQEQLAEHFesrerrvlrr<br>----------------------------<br>qtpledfrvsqealldhenrr----lrr<br>dsaaetssiasgtissrdgkkkqnwlkk<br>----------------------------<br>----------------------------	Hs_ENSP00000244007<br>Bt_ENSBTAP00000023383<br>Rn_ENSRNOP00000021872<br>Mm_ENSMUSP00000099404<br>Gg_ENSGALP00000005944<br>Xt_ENSXETP00000042118<br>Dr_ENSDARP00000073528<br>Ce_CE42905<br>Dm_FBpp0074009<br>Sc_YPL268W	NP_002651 1-phosphatidylinositol-4,5-bisphosphate phosphodiesterase gamma-1 (EC 3.1.4.11)(Phosphoinositide phospholipase C)(Phospholipase C-gamma-1)(PLC-gamma-1)(PLC-II)(PLC-148)
70	1	CI	ENSP00000217446<br>ENSG00000101464	398<br>-<br>398<br>398<br>398<br>398<br>-<br>386<br>390<br>364	yaitltfnvgQILLISDYfyaflrreyy<br>............................<br>yaitltfnvgQILLISDYfyaflrreyy<br>yaitltfnvgQILLISDYfyaflrreyy<br>yaitltfnigQILLISDYfyaflrreyy<br>yaitlsfnvgQILLVSDYfyaflrreyh<br>............................<br>fgttivynvalinlvmdmifvysrrqid<br>fgatlafstgqiflitdllfahvkrefc<br>yaislvyalaiasilvdlnwamlrieyd	Hs_ENSP00000217446<br>Bt<br>Rn_ENSRNOP00000032963<br>Mm_ENSMUSP00000076816<br>Gg_ENSGALP00000001848<br>Xt_ENSXETP00000042186<br>Dr<br>Ce_CE39412<br>Dm_FBpp0079294<br>Sc_YLR459W	NP_536724 Phosphatidylinositol glycan anchor biosynthesis class U protein (GPI transamidase component PIG-U)(Cell division cycle protein 91-like 1)(Protein CDC91-like 1)
71	1	C	ENSP00000323889<br>ENSG00000121060	128<br>143<br>127<br>127<br>41<br>115<br>126<br>-<br>-<br>-	tclvcmasfcQEHLQPHFdspafqdhpl<br>tclvcmasfcqehlrphldspafqdhpl<br>tclvcmasfcqehlrphidspafqdhpl<br>tclvcmasfcQEHLRPHFdspafqdhpl<br>tcltcmasfcpehlrphydspafrhhql<br>scllceahlcenhvlvhsksp---ehvl<br>tcltcmasycedhvrphrenaifrahql<br>............................<br>............................<br>............................	Hs_ENSP00000323889<br>Bt_ENSBTAP00000013121<br>Rn_ENSRNOP00000003186<br>Mm_ENSMUSP00000103528<br>Gg_ENSGALP00000004962<br>Xt_ENSXETP00000041807<br>Dr_ENSDARP00000007921<br>Ce<br>Dm<br>Sc	NP_005073 Tripartite motif-containing protein 25 (Zinc finger protein 147)(Estrogen-responsive finger protein)(Efp)(RING finger protein 147)
72	1	C	ENSP00000323439<br>ENSG00000163029	546<br>555<br>552<br>552<br>554<br>585<br>564<br>547<br>594<br>576	chnhadervlQALMKRFYlpgtsrppii<br>chnhadervlQALMKKFYspgtsrpqii<br>chnhadervlQSLMKKFYppgtsrpqii<br>chnhadervlQSLMKKFYppgtsrpqii<br>cdnhsderilqqlmskyyprg-arpsii<br>cdnhqdermlqnimsreyprg-rrpqii<br>cdnhaderelerimgrfyqrg-rrpqii<br>ccsqedaatlrkifdilkipsndrptiv<br>vgsdrerqslrallqnkfqgg-nmptii<br>vsnpkdnrlfrdimrscgirs----nip	Hs_ENSP00000323439<br>Bt_ENSBTAP00000024303<br>Rn_ENSRNOP00000007073<br>Mm_ENSMUSP00000020931<br>Gg_ENSGALP00000026518<br>Xt_ENSXETP00000033941<br>Dr_ENSDARP00000076074<br>Ce_CE08332<br>Dm_FBpp0083958<br>Sc_YLR383W	NP_078900 Structural maintenance of chromosomes protein 6 (hSMC6)
73	1	CI	ENSP00000005178<br>ENSG00000004799	156<br>151<br>156<br>156<br>179<br>-<br>-<br>-<br>-<br>202	tvdpvtnqnlQYFLDRFYmnristrmlm<br>tvdpvtnqnlQYFLDRFYmnristrmlm<br>tvdpvtnqnlQYFLDRFYmnristrmlm<br>tvdpvtnqnlQYFLDRFYmnristrmlm<br>kvdpvtnqniQYFLDRFYmsristrmlm<br>............................<br>............................<br>............................<br>............................<br>ildlyprekmdqllsdllrarisrrliv	Hs_ENSP00000005178<br>Bt_ENSBTAP00000038686<br>Rn_ENSRNOP00000012759<br>Mm_ENSMUSP00000019721<br>Gg_ENSGALP00000015774<br>Xt<br>Dr<br>Ce<br>Dm<br>Sc_YGL059W	NP_002603 Unknown
74	1	CI	ENSP00000386341<br>ENSG00000134970	266<br>94<br>-<br>94<br>-<br>-<br>-<br>-<br>-<br>-	eddtdealrvQNLLQDDFgikpgiifae<br>eedtgealrvQSLLENDFgikpgiifae<br>............................<br>eddtdealrvQDLLQNDFgirpgivfae<br>............................<br>............................<br>............................<br>............................<br>............................<br>............................	Hs_ENSP00000386341<br>Bt_ENSBTAP00000003042<br>Rn<br>Mm_ENSMUSP00000066239<br>Gg<br>Xt<br>Dr<br>Ce<br>Dm<br>Sc	TIR domain-containing adapter molecule 2 (TICAM-2)(TRIF-related adapter molecule)(Toll/interleukin-1 receptor domain-containing protein)(Toll-like receptor adaptor protein 3)(Putative NF-kappa-B-activating protein 502)
75	1	C	ENSP00000255175	175	migaalfiliQLVLLVDFahswneswvn	Hs_ENSP00000255175	NP_945179 Serine incorporator

			ENSG00000132824	174	mvgaaffiliQLVLLVDFahswneswvn	Bt_ENSBTAP00000002833	3 (Tumor differentially expressed protein 1)
				175	mlgatffifiqlvllvdlahswnelwvn	Rn_ENSRNOP00000013446	
				175	mlgasffiiiqlvllvdmahswnelwvn	Mm_ENSMUSP00000096706	
				175	vcgafcfiliqlvflvdfahswneswvg	Gg_ENSGALP00000006699	
				175	vcgaccfiifQLILLVDFahslneswvn	Xt_ENSXETP00000003695	
				-	....	Dr	
				-	....	Ce	
				-	....	Dm	
				162	vpsgaifilvglillvdfahewaetcis	Sc_YDR105C	
76	1	CI	ENSP00000334934 ENSG00000186509	100	aalsytrcaaQFFLFTFFgsidcyllal	Hs_ENSP00000334934	NP_001005212 Olfactory receptor 9Q1
				-	....	Bt	
				100	aalsyarcvaQFFLFTFFgsidcyllal	Rn_ENSRNOP00000038023	
				100	aalsyarcvaQFFLFTFFgsidcyllal	Mm_ENSMUSP00000067303	
				-	....	Gg	
				-	....	Xt	
				-	....	Dr	
				-	....	Ce	
				-	....	Dm	
				-	....	Sc	
77	1	C	ENSP00000338218 ENSG00000173821	0	----------------------------	Hs_ENSP00000338218	NP_065965 RING finger protein 213
				0	----------------------------	Bt_ENSBTAP00000030425	
				-	....	Rn	
				905	esllrqlgrvkhlvqvdfgnieiihsqd	Mm_ENSMUSP00000091429	
				253	gtflnmcrkvqasvtvyvgevdrqhsed	Gg_ENSGALP00000011305	
				-	....	Xt	
				0	----------------------------	Dr_ENSDARP00000023523	
				-	....	Ce	
				-	....	Dm	
				-	....	Sc	
77	2	CI	ENSP00000338218 ENSG00000173821	33	vtpqapleaiQAYLAGHYrvpkqtlsaa	Hs_ENSP00000338218	NP_065965 RING finger protein 213
				0	----------------------------	Bt_ENSBTAP00000030425	
				-	....	Rn	
				1682	lvpqaplpniQAYLQSHYqvpkrllsaa	Mm_ENSMUSP00000091429	
				1025	mipqrplteiQQYLQHHYrvaqpsnsaa	Gg_ENSGALP00000011305	
				-	....	Xt	
				755	tgitisaersQKYIRHHFkisyelaths	Dr_ENSDARP00000023523	
				-	....	Ce	
				-	....	Dm	
				-	....	Sc	
77	3	C	ENSP00000338218 ENSG00000173821	998	kgicssdilvqdrvqgyfasfakayetv	Hs_ENSP00000338218	NP_065965 RING finger protein 213
				0	----------------------------	Bt_ENSBTAP00000030425	
				-	....	Rn	
				2646	egicssdrlvqdkirgyfapfakayetv	Mm_ENSMUSP00000091429	
				1977	kgiccsargalhkveqyfhhfanayeii	Gg_ENSGALP00000011305	
				-	....	Xt	
				1697	egicsssqpvllkikhllsklakcflsi	Dr_ENSDARP00000023523	
				-	....	Ce	
				-	....	Dm	
				-	....	Sc	
78	1	CI	ENSP00000376352 ENSG00000152256	198	gvdpvtsqnvQYFLDRFYmsrisirmll	Hs_ENSP00000376352	Unknown
				180	gvdpvtsqnvQYFLDRFYmsrisirmll	Bt_ENSBTAP00000038331	
				178	gvdpvtsqnvQYFLDRFYmsrisirmll	Rn_ENSRNOP00000002072	
				178	gvdpvtsqnvQYFLDRFYmsrisirmll	Mm_ENSMUSP00000006669	
				152	gidpvtsqnvQYFLDRFYmsrisirmll	Gg_ENSGALP00000021580	
				113	gvdpvtsqnvQYFLDRFYmsrisirmll	Xt_ENSXETP00000016395	
				147	gtdpitsqnmQYFLDRFYmsrisirmll	Dr_ENSDARP00000022009	
				-	....	Ce	
				-	....	Dm	
				-	....	Sc	
79	1	I	ENSP00000324534 ENSG00000177174	98	ttiskagcvaQVFLVVFFvyvellflti	Hs_ENSP00000324534	NP_001001918 Olfactory receptor 14C36 (Olfactory receptor 5BF1)(Olfactory receptor OR1-59)
				-	....	Bt	
				98	rnislggcaaqiffflfcacvetlflai	Rn_ENSRNOP00000044270	
				98	rnisvagcaaqiflvffcscveiqfltt	Mm_ENSMUSP00000079060	
				106	qaisyagcvaQVFLFVFFisaeysllti	Gg_ENSGALP00000035363	
				-	....	Xt	
				-	....	Dr	
				-	....	Ce	
				-	....	Dm	
				-	....	Sc	
80	1	CI	ENSP00000262126	1930	ereklivsneQEVLRVHYraartlanqt	Hs_ENSP00000262126	NP_056023 Ankyrin repeat

			ENSG00000101745	1918<br>1915<br>1909<br>1909<br>-<br>1266<br>-<br>531<br>-	ereklivsneQEVLRVHYraartlanqt<br>ereklivsneQEVLRVHYraartlanqt<br>ereklivsneQEVLRVHYraartlanqt<br>ereklivsneQEVLRVHYraartlanqt<br>............................<br>----------------------------<br>............................<br>ereklcmnveqeiirvhskaarsisgqp<br>............................	Bt_ENSBTAP00000003572<br>Rn_ENSRNOP00000017613<br>Mm_ENSMUSP00000039035<br>Gg_ENSGALP00000013668<br>Xt<br>Dr_ENSDARP00000088636<br>Ce<br>Dm_FBpp0075653<br>Sc	domain-containing protein 12 (Ankyrin repeat-containing cofactor 2)(GAC-1 protein)
81	1	CI	ENSP00000252137<br>ENSG00000100056	48<br>48<br>51<br>51<br>50<br>54<br>15<br>52<br>61<br>-	ldeeeyieglQTVIQRDFfpdveklqaq<br>ldeeeyieglQTVIQRDFfpdveklqaq<br>ldeeeyieglQTVIQRDFfpdveklqaq<br>ldeeeyieglQTVIQRDFfpdveklqaq<br>ldedayiesleniiqrdffpdveklraq<br>lceetyiqnlQKIIQRDFfpdveklraq<br>ldeeqyieslekiiqrdffpdvsklqaq<br>vpeekyiagldkiiekdyfphlkkmqaq<br>lteekyiemskiiqrdffpdlerlraq<br>............................	Hs_ENSP00000252137<br>Bt_ENSBTAP00000024669<br>Rn_ENSRNOP00000000308<br>Mm_ENSMUSP00000003621<br>Gg_ENSGALP00000009447<br>Xt_ENSXETP00000048453<br>Dr_ENSDARP00000080770<br>Ce_CE37109<br>Dm_FBpp0071145<br>Sc	NP_073210 Protein DGCR14 (DiGeorge syndrome critical region 14)(DiGeorge syndrome critical region 13)(DiGeorge syndrome protein H)(DGS-H)(Protein ES2)
82	1	CI	ENSP00000221476<br>ENSG00000104879	185<br>185<br>185<br>185<br>-<br>185<br>185<br>220<br>-<br>-	plksmtekeqQQLIDDHFlfdkpvspll<br>plksmteqeqQQLIDDHFlfdkpvspll<br>plksmteqeqQQLIDDHFlfdkpvspll<br>plksmteqeqQQLIDDHFlfdkpvspll<br>............................<br>plkdmsdaeqQQLIDDHFlfdkpvspll<br>plksmtdaeqeqliadhflfdkpvspll<br>pldgmtkeiqdqlikdhflfke-gdrfl<br>............................<br>............................	Hs_ENSP00000221476<br>Bt_ENSBTAP00000018492<br>Rn_ENSRNOP00000022895<br>Mm_ENSMUSP00000003643<br>Gg<br>Xt_ENSXETP00000041410<br>Dr_ENSDARP00000037871<br>Ce_CE37112<br>Dm<br>Sc	NP_001815 Creatine kinase M-type (EC 2.7.3.2)(Creatine kinase M chain)(M-CK)
83	1	CI	ENSP00000256689<br>ENSG00000134294	34<br>46<br>34<br>34<br>49<br>31<br>37<br>-<br>-<br>-	dfnys-yptkQAALKSHYadvdpenqnf<br>dfnys-yptkQAALKSHYadvdpenqnf<br>dfnys-yptkQAALKSHYvdvdpenqnf<br>dfnys-yptkQAALKSHYadvdpenqnf<br>dfsyp-yptkpaamkshyadmdpenqnf<br>dlnyseyqpknhpikshy-dmdienvhf<br>ytnyqdhaskvplngiqysdveaesqnf<br>............................<br>............................<br>............................	Hs_ENSP00000256689<br>Bt_ENSBTAP00000014749<br>Rn_ENSRNOP00000031532<br>Mm_ENSMUSP00000023099<br>Gg_ENSGALP00000015772<br>Xt_ENSXETP00000032118<br>Dr_ENSDARP00000067444<br>Ce<br>Dm<br>Sc	NP_061849 Sodium-coupled neutral amino acid transporter 2 (Amino acid transporter A2)(System A amino acid transporter 2)(System N amino acid transporter 2)(System A transporter 1)(Solute carrier family 38 member 2)(Protein 40-9-1)
84	1	CI	ENSP00000290687<br>ENSG00000159495	159<br>159<br>157<br>65<br>-<br>-<br>-<br>-<br>-<br>-	dvylpseillQEYIMRDYgfvykgherf<br>dvylpsetllQEYIMMDYgfvykgherf<br>dvylpseallreyimcdygfvykgqans<br>dvylpsetllreyimsdygfiykgqtns<br>............................<br>............................<br>............................<br>............................<br>............................<br>............................	Hs_ENSP00000290687<br>Bt_ENSBTAP00000020482<br>Rn_ENSRNOP00000053033<br>Mm_ENSMUSP00000106303<br>Gg<br>Xt<br>Dr<br>Ce<br>Dm<br>Sc	NP_443187 Protein-glutamine gamma-glutamyltransferase Z (TGase Z)(TGZ)(TG(Z))(EC 2.3.2.13)(Transglutaminase 7)
85	1	C	ENSP00000308208<br>ENSG00000157227	174<br>173<br>174<br>174<br>-<br>165<br>167<br>-<br>163<br>-	yayireghekQADIMIFFaegfhgdstp<br>yayireghekQADIMIFFaegfhgdstp<br>yayireghekqadimilfaegfhgdstp<br>yayireghekqadimilfaegfhgdstp<br>............................<br>yvdikdgytkhadimlffaegfhgdstp<br>ysdirdkvvdfadimlffadgfhgdasp<br>............................<br>sg--------pvhieikfvesehgdgda<br>............................	Hs_ENSP00000308208<br>Bt_ENSBTAP00000019744<br>Rn_ENSRNOP00000049168<br>Mm_ENSMUSP00000087119<br>Gg<br>Xt_ENSXETP00000048923<br>Dr_ENSDARP00000044263<br>Ce<br>Dm_FBpp0271771<br>Sc	NP_004986 Matrix metalloproteinase-14 Precursor (MMP-14)(EC 3.4.24.80)(Membrane-type matrix metalloproteinase 1)(MT-MMP 1)(MTMMP1)(Membrane-type-1 matrix metalloproteinase)(MT1-MMP)(MT1MMP)(MMP-X1)
86	1	C	ENSP00000352608<br>ENSG00000196218	1230<br>-<br>1231<br>1232<br>-<br>1232<br>1239<br>-<br>-<br>-	egfepfainmqrpvttwfskglpqfepv<br>............................<br>egfepfainmqrpvttwfskslpqfepv<br>egfepfainmqrpvttwfskslpqfepv<br>............................<br>egfepfainmkrdittwfskslpqfvnv<br>egfepfainmkrditmwfskslpqfvpv<br>............................<br>............................<br>............................	Hs_ENSP00000352608<br>Bt<br>Rn_ENSRNOP00000027893<br>Mm_ENSMUSP00000032813<br>Gg<br>Xt_ENSXETP00000006174<br>Dr_ENSDARP00000032856<br>Ce<br>Dm<br>Sc	NP_000531 Ryanodine receptor 1 (RYR-1)(RyR1)(Skeletal muscle-type ryanodine receptor)(Skeletal muscle calcium release channel)

86	2	CI	ENSP00000352608<br>ENSG00000196218	3926<br>-<br>3930<br>3930<br>-<br>3921<br>3958<br>-<br>-<br>-	ictvdyllrlQESISDFYwyysgkdvie<br>............................<br>ictvdyllrlQESISDFYwyysgkdvie<br>ictvdyllrlQESISDFYwyysgkdvie<br>............................<br>ictvdyllrlQESISDFYwyysgkdiid<br>ictvdyllrlQESISDFYwyysgkdiid<br>............................<br>............................<br>............................	Hs_ENSP00000352608<br>Bt<br>Rn_ENSRNOP00000027893<br>Mm_ENSMUSP00000032813<br>Gg<br>Xt_ENSXETP00000006174<br>Dr_ENSDARP00000032856<br>Ce<br>Dm<br>Sc	NP_000531 Ryanodine receptor 1 (RYR-1)(RyR1)(Skeletal muscle-type ryanodine receptor) (Skeletal muscle calcium release channel)
87	1	CI	ENSP00000367830<br>ENSG00000067606	536<br>538<br>-<br>536<br>536<br>-<br>543<br>-<br>-<br>-	lekkqalppfQPQITDDYgldnfdtqft<br>lgkkqalppfQPQITDDYgldnfdtqft<br>............................<br>lekkqtlppfQPQITDDYgldnfdtqft<br>lekkqttppfQPQITDDYgldnfdtqft<br>............................<br>leqkqvtppfkpqitddyglenfdtqft<br>............................<br>............................<br>............................	Hs_ENSP00000367830<br>Bt_ENSBTAP00000018768<br>Rn<br>Mm_ENSMUSP00000030922<br>Gg_ENSGALP00000001936<br>Xt<br>Dr_ENSDARP00000063591<br>Ce<br>Dm<br>Sc	NP_001028753 Protein kinase C zeta type (EC 2.7.11.13) (nPKC-zeta)
88	1	CI	ENSP00000373767<br>ENSG00000189056	2844<br>2672<br>2613<br>2845<br>2736<br>-<br>-<br>-<br>-<br>-	frfyqkysdmQWAIDNFYlgpgcldncr<br>frfyqkhsdmQWAIDNFYlgpecldncr<br>frfyqkysdvQWAIDNFYlgpgcldncg<br>frfyqkysdvQWAIDNFYlgpgcldncg<br>frfyqkysdmQWAIDNFYlgpgclencr<br>............................<br>............................<br>............................<br>............................<br>............................	Hs_ENSP00000373767<br>Bt_ENSBTAP00000004768<br>Rn_ENSRNOP00000010521<br>Mm_ENSMUSP00000058025<br>Gg_ENSGALP00000013329<br>Xt<br>Dr<br>Ce<br>Dm<br>Sc	NP_005036 Reelin Precursor (EC 3.4.21.-)
88	2	CI	ENSP00000373767<br>ENSG00000189056	3442<br>3269<br>3208<br>3443<br>3334<br>-<br>-<br>-<br>-<br>-	kqnymmnfsrQHGLRHFYnrrrrslrry<br>kqnymmnfsrQHGLRHFYnrrrrslrry<br>kqnymmnfsrQHGLRHFYnrrrrslrry<br>kqnymmnfsrQHGLRHFYnrrrrslrry<br>kqnymmnfsrQHGLRHFYnrrrrslrry<br>............................<br>............................<br>............................<br>............................<br>............................	Hs_ENSP00000373767<br>Bt_ENSBTAP00000004768<br>Rn_ENSRNOP00000010521<br>Mm_ENSMUSP00000058025<br>Gg_ENSGALP00000013329<br>Xt<br>Dr<br>Ce<br>Dm<br>Sc	NP_005036 Reelin Precursor (EC 3.4.21.-)
89	1	CI	ENSP00000346305<br>ENSG00000062370	200<br>192<br>187<br>201<br>-<br>-<br>-<br>-<br>-<br>-	shn-yqcrcqQISMKNHFckcdsvswls<br>srn-yqcrcqqvskendfcksdsiswis<br>pcn-clwkgqQTSLRYHFcrcdsigwss<br>pcvtcqwkgqQTSIRNHFcrygsvcwsp<br>............................<br>............................<br>............................<br>............................<br>............................<br>............................	Hs_ENSP00000346305<br>Bt_ENSBTAP00000050759<br>Rn_ENSRNOP00000038599<br>Mm_ENSMUSP00000005413<br>Gg<br>Xt<br>Dr<br>Ce<br>Dm<br>Sc	NP_001076804 Zinc finger protein 112 homolog (Zfp-112) (Zinc finger protein 228)
90	1	CI	ENSP00000257700<br>ENSG00000135249	509<br>509<br>450<br>509<br>485<br>461<br>492<br>-<br>462<br>-	asrklqflelQKDLVDDFrirltqvmke<br>asrklqflelQKDLVDDFrirltqvmke<br>asrklqflelQKDLVDDFrirltqvmke<br>asrklqflelQKDLVDDFrirltqvmke<br>asrklqflglQKELVDDFrirltqvmke<br>asrklkflelQKDLVDDFrirltqvmke<br>qraqlsflalQKELVDDFrirltqvmke<br>............................<br>pghqlqflhlqlelidsfrqrlvqlhss<br>............................	Hs_ENSP00000257700<br>Bt_ENSBTAP00000049041<br>Rn_ENSRNOP00000030177<br>Mm_ENSMUSP00000030852<br>Gg_ENSGALP00000013171<br>Xt_ENSXETP00000050203<br>Dr_ENSDARP00000045638<br>Ce<br>Dm_FBpp0076549<br>Sc	NP_068749 RAD50-interacting protein 1 (RAD50 interactor 1) (Protein RINT-1)(HsRINT-1)
91	1	CI	ENSP00000007708<br>ENSG00000005882	152<br>184<br>152<br>152<br>-<br>152<br>154<br>149<br>-<br>-	gddpvsnqniQYFLDRFYlsrisirmli<br>gddpvsnqniQYFLDRFYlsrisirmli<br>gddpvsnqniQYFLDRFYlsrisirmli<br>gddpvsnqniQYFLDRFYlsrisirmli<br>............................<br>gvdpvtnqnvQYFLDRFYmsrisirmli<br>gvdpvtnqnvQYFLDRFYmsristrmlm<br>gvdiasekgiQYFLDRFYinrisirmlq<br>............................<br>............................	Hs_ENSP00000007708<br>Bt_ENSBTAP00000011523<br>Rn_ENSRNOP00000005641<br>Mm_ENSMUSP00000041447<br>Gg<br>Xt_ENSXETP00000043770<br>Dr_ENSDARP00000071589<br>Ce_CE00397<br>Dm<br>Sc	NP_002602 Unknown

92	1	C	ENSP00000357880<br>ENSG00000123505	311<br>315<br>311<br>311<br>318<br>315<br>311<br>332<br>323<br>378	----fkrldcQSAMFNDYnfvftsfakk<br>----fkrldcQSALFNDYnfvftsfakk<br>----fkrldcQSAMFNDYnfvftsfakk<br>----fkrldcQSAMFNDYnfvftsfakk<br>----fkrldhqiaqfsdynfvftsftkn<br>----frrldrqfaqfndynfvftsfaki<br>----yrlldrqlahfndynfvftsyaks<br>pg--yrrtnvqfvrletetlvyahfvrk<br>qgshwkrtdmqccnfpsynllfaqyshs<br>slpdyikldkivydlddyhlfymklqkk	Hs_ENSP00000357880<br>Bt_ENSBTAP00000004338<br>Rn_ENSRNOP00000000715<br>Mm_ENSMUSP00000097528<br>Gg_ENSGALP00000024221<br>Xt_ENSXETP00000046135<br>Dr_ENSDARP00000064390<br>Ce_CE18705<br>Dm_FBpp0079584<br>Sc_YOL052C	NP_001028231 S-adenosylmethionine decarboxylase proenzyme (AdoMetDC)(SamDC)(EC 4.1.1.50)
93	1	CI	ENSP00000319705<br>ENSG00000140274	172<br>172<br>172<br>172<br>170<br>-<br>-<br>-<br>-<br>-	tpsspcglyhQYHLAGHYasatlwvafc<br>tpsspcgvyrQYRLAGHYasatlwvafc<br>tpnspcglyhqyhyaghyagatlwvafc<br>tpsspcglyhQYHLAGHYaaatlwvafc<br>ttqspcnvhrQYRISSHYasaslwvafc<br>............................<br>............................<br>............................<br>............................<br>............................	Hs_ENSP00000319705<br>Bt_ENSBTAP00000021606<br>Rn_ENSRNOP00000024201<br>Mm_ENSMUSP00000028656<br>Gg_ENSGALP00000003851<br>Xt<br>Dr<br>Ce<br>Dm<br>Sc	NP_997464 Dual oxidase maturation factor 2 (Dual oxidase activator 2)
94	1	C	ENSP00000222124<br>ENSG00000105518	50<br>50<br>50<br>50<br>-<br>50<br>50<br>-<br>-<br>44	slprhtfglvQSKLFPFYfhismgcafi<br>glprhtfglvQSKLFPFYfhismgcafv<br>slprhtfglvqsklfpvyfhvslgcafi<br>slprhtfglvqskvfpvyfhvslgcafi<br>............................<br>gvprhtfglvQSKLFPFYnhivlccsfi<br>qvsmhtfglvqsklfpvyfycllggnav<br>............................<br>............................<br>vlekdqfsalqnkifpyffqmqaasp--	Hs_ENSP00000222124<br>Bt_ENSBTAP00000021848<br>Rn_ENSRNOP00000015684<br>Mm_ENSMUSP00000048832<br>Gg<br>Xt_ENSXETP00000017377<br>Dr_ENSDARP00000064027<br>Ce<br>Dm<br>Sc_YPR098C	NP_940938 Transmembrane protein 205
95	1	CI	ENSP00000257789<br>ENSG00000135336	329<br>308<br>334<br>335<br>335<br>329<br>328<br>-<br>319<br>-	fsvqnfikglQLSLLEHFysqplsvlcc<br>fsiqnfikglQLSLLEHFysqplsvlcc<br>fsiqnfikglklsllehfysqplsvlcc<br>fsiqsfikgiklsllehfysqplsvlcc<br>fsvqnfikgfQLCIVEHFhsnplsvlcc<br>fsvqnfikglqlsvvehfytqplsvlcc<br>fsvqnfvkglQFSMLEHFnsqplsvlcc<br>............................<br>fsihgfiqgfkyclmehffggnafalct<br>............................	Hs_ENSP00000257789<br>Bt_ENSBTAP00000022571<br>Rn_ENSRNOP00000011085<br>Mm_ENSMUSP00000048319<br>Gg_ENSGALP00000025431<br>Xt_ENSXETP00000042849<br>Dr_ENSDARP00000047856<br>Ce<br>Dm_FBpp0086881<br>Sc	NP_862820 Origin recognition complex subunit 3 (Origin recognition complex subunit Latheo)
96	1	CI	ENSP00000357475<br>ENSG00000143515	871<br>858<br>-<br>862<br>845<br>-<br>862<br>787<br>1322<br>-	gvgisgqegiQAVLASDYsfsqfkflqr<br>gvgisgqegiQAVLASDYsfsqfkflqr<br>............................<br>gvgisgqegiQAVLASDYsfsqfkflqr<br>gvgisgqegmQAVLSSDFsfaqfrylqr<br>............................<br>gvgisgqegmQAVLSSDFsfaqfrflqr<br>gvgisgqegmQAVLASDYsigqfkyler<br>gvgisgqeglQAVLSSDYsiaqfryler<br>............................	Hs_ENSP00000357475<br>Bt_ENSBTAP00000006808<br>Rn<br>Mm_ENSMUSP00000103019<br>Gg_ENSGALP00000015054<br>Xt<br>Dr_ENSDARP00000083606<br>Ce_CE27180<br>Dm_FBpp0289580<br>Sc	NP_065185 Probable phospholipid-transporting ATPase ID (EC 3.6.3.1)(ATPase class I type 8B member 2)
97	1	CI	ENSP00000257749<br>ENSG00000112182	378<br>380<br>377<br>377<br>309<br>358<br>341<br>-<br>-<br>-	pacpfdkgitQGDLKTDYtpftg-nygq<br>sacpfdkgitQGDLKTDYapfpg-nygq<br>sacpfnkgisQGDLKTDYtplag-nygq<br>sacpfnkgisQGDLKTDYtplag-nygq<br>pacpfekgttqgdhktdyvpftg-nygq<br>pacpvekratqgdhkteynsfig-nfep<br>linaqdkgttqgdhkkdyrpslgnefgm<br>............................<br>............................<br>............................	Hs_ENSP00000257749<br>Bt_ENSBTAP00000027603<br>Rn_ENSRNOP00000008309<br>Mm_ENSMUSP00000103815<br>Gg_ENSGALP00000025114<br>Xt_ENSXETP00000041466<br>Dr_ENSDARP00000036016<br>Ce<br>Dm<br>Sc	NP_068585 Transcription regulator protein BACH2 (BTB and CNC homolog 2)
98	1	CI	ENSP00000380308<br>ENSG00000077380	240<br>214<br>243<br>240<br>211<br>249<br>254<br>247<br>274<br>-	triveralseQINIFFDYsgrdledkeg<br>triveralseQINIFFDYsgrdledkeg<br>triveralseQINIFFDYsgrdledkeg<br>triveralseQINIFFDYsgrdledkeg<br>triveralseQINIFFDYsgrdledkeg<br>triveralsehvniffdysgrdleekeg<br>triveralsehvdvffdysgrdmeekeg<br>ckvisralneeidicinyt---kdpyek<br>grvieralsenvdiytdyigggdseean<br>............................	Hs_ENSP00000380308<br>Bt_ENSBTAP00000044101<br>Rn_ENSRNOP00000042414<br>Mm_ENSMUSP00000107768<br>Gg_ENSGALP00000015531<br>Xt_ENSXETP00000043756<br>Dr_ENSDARP00000061839<br>Ce_CE16862<br>Dm_FBpp0088428<br>Sc	Cytoplasmic dynein 1 intermediate chain 2 (Cytoplasmic dynein intermediate chain 2)(Dynein intermediate chain 2, cytosolic) (DH IC-2)

99	1	C	ENSP00000384408 ENSG00000214982	731	tyegtieengQGMLQVDFanrfvgggvt	Hs_ENSP00000384408	NP_003622 Poly(ADP-ribose) glycohydrolase (EC 3.2.1.143)
				732	tyegtiegngQGMLQVDFanrfvgggvt	Bt_ENSBTAP00000031225	
				727	tyegtiegngrgmlqvdfanrfvgggvt	Rn_ENSRNOP00000027071	
				724	tyegtiegngrgmlqvdfanrfvgggvt	Mm_ENSMUSP00000022470	
				656	tyegtiesngQGMLQVDFanrfvgggvt	Gg_ENSGALP00000003301	
				-	....	Xt	
				-	....	Dr	
				530	ffdemliedtalctqvdfanehlgggvl	Ce_CE33775	
				361	daegtiedegigllqvdfankylgggvl	Dm_FBpp0070560	
				-	....	Sc	
100	1	CI	ENSP00000357477 ENSG00000111860	400	erirdnelraQHAMLGHYvncedsyvas	Hs_ENSP00000357477	NP_996804 Coiled-coil domain-containing protein C6orf204 (Serologically defined breast cancer antigen NY-BR-15)
				324	erirdnelraQHAMLGHYvncegssgag	Bt_ENSBTAP00000017179	
				279	erirdnelraQHAMLGHYvncedsymss	Rn_ENSRNOP00000000470	
				280	erirdnelraQHAMLGHYvncedsyvsn	Mm_ENSMUSP00000093356	
				375	qkirenelrvqharlshlvnceepymtn	Gg_ENSGALP00000023977	
				367	qalrandlqanqavmghavngedfamhh	Xt_ENSXETP00000028140	
				38	arireneiraqqvlqnqrgryedsyllk	Dr_ENSDARP00000061140	
				-	....	Ce	
				-	....	Dm	
				-	....	Sc	
101	1	CI	ENSP00000260227 ENSG00000137673	36	gmselqweqaQDYLKRFYlydsetknan	Hs_ENSP00000260227	NP_002414 Matrilysin Precursor (EC 3.4.24.23)(Pump-1 protease)(Uterine metalloproteinase)(Matrix metalloproteinase-7)(MMP-7)(Matrin)
				36	gegdprwqlaQDYLKRFYssdskiknan	Bt_ENSBTAP00000006029	
				39	evtalqweqaQNYLRKFYlhdsktkkat	Rn_ENSRNOP00000014041	
				39	dvsahqweqaQNYLRKFYphdsktkkvn	Mm_ENSMUSP00000018767	
				36	lwsnadldivQTYLNKFFpllekhp-av	Gg_ENSGALP00000027717	
				-	....	Xt	
				-	....	Dr	
				-	....	Ce	
				-	....	Dm	
				-	....	Sc	
102	1	CI	ENSP00000364464 ENSG00000148120	619	lrkfvhtfhgQLILSQDFlqmllenipe	Hs_ENSP00000364464	Aminopeptidase O (AP-O)(EC 3.4.11.-)
				-	....	Bt	
				622	lrkfvhlfhgQLILSQDFlqmllesipe	Rn_ENSRNOP00000023638	
				621	lrkfvhlfhgQLILSQDFlqmllenipe	Mm_ENSMUSP00000089148	
				624	lrkfvhkfhgQLVLSQDFlsmlledipe	Gg_ENSGALP00000020566	
				-	....	Xt	
				-	....	Dr	
				-	....	Ce	
				-	....	Dm	
				-	....	Sc	
103	1	CI	ENSP00000260229 ENSG00000137675	34	teneenmqlaQAYLNQFYsleiegnhlv	Hs_ENSP00000260229	NP_071405 Matrix metalloproteinase-27 Precursor (MMP-27)(EC 3.4.24.-)
				0	----------------------------	Bt_ENSBTAP00000004210	
				47	dkneennqlaQAYLNQFYsleiegshlv	Rn_ENSRNOP00000058603	
				48	dkneennqlaQAYLNQFYsleiegshfv	Mm_ENSMUSP00000091423	
				40	kdnkedtklvedylskfytietdsnqrg	Gg_ENSGALP00000027709	
				-	....	Xt	
				-	....	Dr	
				-	....	Ce	
				-	....	Dm	
				-	....	Sc	
104	1	CI	ENSP00000360777 ENSG00000148384	353	sdrrewetrlQETLGPHYvllssaahgv	Hs_ENSP00000360777	NP_063945 72 kDa inositol polyphosphate 5-phosphatase (EC 3.1.3.36) (Phosphatidylinositol-4,5-bisphosphate 5-phosphatase) (Phosphatidylinositol polyphosphate 5-phosphatase type IV)
				365	sdrrewearlQETLGPHYvmlysaahga	Bt_ENSBTAP00000001784	
				357	sdrrewetrlqetlgpqyvllssaahgv	Rn_ENSRNOP00000025818	
				356	sdrrewetrlqetlgpqyvllssaahgv	Mm_ENSMUSP00000028291	
				303	pdrreweirlQETLGPHYvmlysaahgv	Gg_ENSGALP00000003041	
				259	pdrreweirlQETLGPHYvllhssghgv	Xt_ENSXETP00000001666	
				-	....	Dr	
				151	ptfheealrilepvlnghrlylshraws	Ce_CE32212	
				444	pdrfewevtiqetlgpshvlfhattlgt	Dm_FBpp0075739	
				-	....	Sc	
105	1	C	ENSP00000381034 ENSG00000170677	29	keetdfmvvqQPSLASDFgkddslfgsc	Hs_ENSP00000381034	NP_004223 Suppressor of cytokine signaling 6 (SOCS-6)(Suppressor of cytokine signaling 4)(SOCS-4)(Cytokine-inducible SH2 protein 4)(CIS-4)
				29	kedaefmvvqQPALAGDFgkeeslfgsc	Bt_ENSBTAP00000023144	
				29	kdetefmvvqpqslagdfvkddslfgsc	Rn_ENSRNOP00000054982	
				29	kdetefmvvqpqslagdfvkddslfgsc	Mm_ENSMUSP00000064929	
				29	kdesdfvvvqqpsls-efgkddslfgsc	Gg_ENSGALP00000036145	
				29	keendfvmvkqpsmtnnfgkddslfgsc	Xt_ENSXETP00000023594	
				28	kedgdfvmlqqptiaaeftkddtlfggc	Dr_ENSDARP00000075890	
				-	....	Ce	
				-	....	Dm	
				-	....	Sc	

106	1	CI	ENSP00000371089 ENSG00000088833	35	fflesagwdlQIALASFYedggdedivt	Hs_ENSP00000371089	NSFL1 cofactor p47 (p97 cofactor p47)(UBX domain-containing protein 2C)
				35	fflesagwdlQIALASFYedggdedivt	Bt_ENSBTAP00000008580	
				35	fflesagwdlQIALASFYedggdedivt	Rn_ENSRNOP00000011654	
				35	fflesagwdlQIALASFYedggdedivt	Mm_ENSMUSP00000086542	
				35	fflesagwdlQIALASFYedggdedilt	Gg_ENSGALP00000038618	
				-	....	Xt	
				34	fflesagwdlQLALANFFedggeddiat	Dr_ENSDARP00000026327	
				19	----------------------------	Ce_CE27555	
				34	fylsscdwdiehalgnywstqadlpvpv	Dm_FBpp0088069	
				34	qylsefg-dlnealnsyyasqtddqkdr	Sc_YBL058W	
107	1	C	ENSP00000234396 ENSG00000116039	491	fpkemlkripQAVIDEFYsregalqdla	Hs_ENSP00000234396	NP_001683 V-type proton ATPase subunit B, kidney isoform (V-ATPase subunit B 1)(Vacuolar proton pump subunit B 1)(Endomembrane proton pump 58 kDa subunit)
				491	fpkemlkripQNIIDEFFsregapqdte	Bt_ENSBTAP00000014039	
				491	fpkemlkripqsvtdefysrqgaqqdpa	Rn_ENSRNOP00000018302	
				491	fpkemlkripqsmtdefysrqgaqqdpa	Mm_ENSMUSP00000006431	
				-	....	Gg	
				473	fpkellkripesmlaefypresrahg--	Xt_ENSXETP00000046546	
				-	....	Dr	
				-	....	Ce	
				-	....	Dm	
				-	....	Sc	
108	1	CI	ENSP00000351881 ENSG00000186106	18	dssqtnvpllQACIDGDFnyskrllesg	Hs_ENSP00000351881	Ankyrin repeat domain-containing protein 46 (Ankyrin repeat small protein)(ANK-S)
				18	dssqtnvpllQACIDGDFnyskrllesg	Bt_ENSBTAP00000001658	
				18	dssqtnvpllQACIDGDFtyskrllesg	Rn_ENSRNOP00000034048	
				18	dssqtnvpllQACIDGDFtyskrllesg	Mm_ENSMUSP00000052521	
				18	dssqtnvpllQACIDGDFnyskrllesg	Gg_ENSGALP00000028214	
				-	....	Xt	
				18	dssqtsvpllqacidgdlsfarrlletg	Dr_ENSDARP00000068309	
				-	....	Ce	
				-	....	Dm	
				7	----mnaniwvaasdgnldrvehilres	Sc_YCR051W	
109	1	CI	ENSP00000369257 ENSG00000103494	519	lektrnmlimQHKINKDYqmeveavtrk	Hs_ENSP00000369257	NP_056087 Protein fantom (RPGR-interacting protein 1-like protein)(RPGRIP1-like protein)
				519	lektrnmlimQHKINKDYqmeveavtqk	Bt_ENSBTAP00000016585	
				519	lektrnmlimQHKINKDYqmevetvtqk	Rn_ENSRNOP00000015876	
				519	lektrnmlimQHKINKDYqmevetvtqk	Mm_ENSMUSP00000042702	
				522	lektrsmlivqhkinkgyqteieavtqk	Gg_ENSGALP00000038922	
				-	....	Xt	
				-	....	Dr	
				711	lekvrnmlliqydinqkqmkeikllkde	Ce_CE43262	
				-	....	Dm	
				-	....	Sc	
110	1	CI	ENSP00000355121 ENSG00000198885	173	synwltdfpsQEALDSFYkhyvqnaird	Hs_ENSP00000355121	NP_848590 Inositol 1,4,5-triphosphate receptor-interacting protein-like 1 Precursor
				137	synwltdfpsrealesfhkhhvqnvtrd	Bt_ENSBTAP00000046360	
				158	sytwlsgfpsQEALESFYkhyiqnaird	Rn_ENSRNOP00000016172	
				158	sytrlsdfpsQEALEAFYkhyiqnaird	Mm_ENSMUSP00000106016	
				64	------eyfhhsaltryiarrawekmqg	Gg_ENSGALP00000031820	
				-	....	Xt	
				-	....	Dr	
				-	....	Ce	
				-	....	Dm	
				-	....	Sc	
111	1	CI	ENSP00000355533 ENSG00000198626	3880	istvdyllrvQESISDFYwyysgkdvid	Hs_ENSP00000355533	Ryanodine receptor 2 (RYR-2)(RyR2)(hRYR-2)(Cardiac muscle-type ryanodine receptor)(Cardiac muscle ryanodine receptor-calcium release channel)
				-	....	Bt	
				3870	istvdyllrvQESISDFYwyysgkdiid	Rn_ENSRNOP00000023601	
				3906	istvdyllrvQESISDFYwyysgkdiid	Mm_ENSMUSP00000021750	
				3869	istvdyllrvQESISDFYwyysgkdvid	Gg_ENSGALP00000017561	
				-	....	Xt	
				-	....	Dr	
				4194	nctvdyllrlqesvmdfywhysskevid	Ce_CE43332	
				4057	ictvdyllrlQESIMDFYwhysskeiid	Dm_FBpp0087721	
				-	....	Sc	
111	2	C	ENSP00000355533 ENSG00000198626	4486	safwkkiiayqqkllnyfarnfynmrml	Hs_ENSP00000355533	Ryanodine receptor 2 (RYR-2)(RyR2)(hRYR-2)(Cardiac muscle-type ryanodine receptor)(Cardiac muscle ryanodine receptor-calcium release channel)
				-	....	Bt	
				4475	safwkkiiayqqkllnyfarnfynmrml	Rn_ENSRNOP00000023601	
				4511	safwkkiiayqqkllnyfarnfynmrml	Mm_ENSMUSP00000021750	
				4476	safwkkiiayqqkllnyfarnfynmrml	Gg_ENSGALP00000017561	
				-	....	Xt	
				-	....	Dr	
				4758	kiaesnstksrgsilnmlarnfktieki	Ce_CE43332	
				4634	avhqidfsqythravsflarnfynlkyv	Dm_FBpp0087721	
				-	....	Sc	

112	1	C	ENSP00000386239<br>ENSG00000160949	639	rkglspletlqqwvklyrrdldletrqk	Hs_ENSP00000386239	NP_038460 NF-kappa-B inhibitor-like protein 2 (Nuclear factor of kappa light polypeptide gene enhancer in B-cells inhibitor-like 2)(Inhibitor of kappa B-related protein)(I-kappa-B-related protein)(IkappaBR)
				637	rkghnpletlqqwvklygkdldsetqek	Bt_ENSBTAP00000010191	
				489	rkglspletlqqwvklyfrdldletrqk	Rn_ENSRNOP00000019754	
				489	rkglspletlqqwvklyfrdldletrqk	Mm_ENSMUSP00000078735	
				-	....	Gg	
				470	akgvaplgslqdwigtygkhldqetrqn	Xt_ENSXETP00000044257	
				-	....	Dr	
				-	....	Ce	
				651	dynetciagldkwrqgaqlvdgeqaqya	Dm_FBpp0076526	
				-	....	Sc	
113	1	CI	ENSP00000355537<br>ENSG00000077522	422	thetwaygkeQILLQKDYesasltevra	Hs_ENSP00000355537	NP_001094 Alpha-actinin-2 (Alpha-actinin skeletal muscle isoform 2)(F-actin cross-linking protein)
				381	thetwaygkeQILLQKDYesstltevra	Bt_ENSBTAP00000012786	
				422	thetwaygkeQILLQKDYesasltevra	Rn_ENSRNOP00000024098	
				422	thetwaygkeQILLQKDYesasltevra	Mm_ENSMUSP00000067708	
				425	theqwaygkeQILLQKDYesasltevra	Gg_ENSGALP00000023309	
				422	lheswtagkeQLLLQKDYetasltevra	Xt_ENSXETP00000038879	
				423	nheswatgkeemltrkdyesaslmevra	Dr_ENSDARP00000095652	
				446	lheewahgkedalrsndwrscglykika	Ce_CE06539	
				440	ahedwtrgkeemlqsqdfrqcklnelka	Dm_FBpp0070331	
				-	....	Sc	
114	1	CI	ENSP00000283131<br>ENSG00000153147	98	ranrfeyllkQTELFAHFiqpaaqktp-	Hs_ENSP00000283131	NP_003592 SWI/SNF-related matrix-associated actin-dependent regulator of chromatin subfamily A member 5 (SWI/SNF-related matrix-associated actin-dependent regulator of chromatin A5)(EC 3.6.1.-)(Sucrose nonfermenting protein 2 homolog)(hSNF2H)
				98	ranrfeyllkQTELFAHFiqpaaqktp-	Bt_ENSBTAP00000004408	
				97	ranrfeyllkQTELFAHFiqpaaqktp-	Rn_ENSRNOP00000024568	
				97	ranrfeyllkQTELFAHFiqpaaqktp-	Mm_ENSMUSP00000044361	
				83	ranrfeyllkQTELFAHFiqpaaqktp-	Gg_ENSGALP00000016102	
				94	rsnrfeyllkqtevfahfiqpaaqktp-	Xt_ENSXETP00000038157	
				-	....	Dr	
				-	....	Ce	
				53	rsrrfdfllkQTEIFTHFmt-nsaksp-	Dm_FBpp0086954	
				97	tykrfkyllgvtdlfrhfigikakhdkn	Sc_YOR304W	
115	1	CI	ENSP00000318900<br>ENSG00000112320	257	lqfcsakclnQYKMDIFYketqanlpag	Hs_ENSP00000318900	NP_060483 Sine oculis-binding protein homolog (Jackson circler protein 1)
				257	lqfcsakclnQYKMDIFYketqanlpag	Bt_ENSBTAP00000025519	
				-	....	Rn	
				257	lqfcsakclnQYKMDIFYketqanlpag	Mm_ENSMUSP00000040072	
				-	....	Gg	
				195	lqfcsakclnQYKMDIFYketqanlpag	Xt_ENSXETP00000041734	
				-	....	Dr	
				-	....	Ce	
				311	lqfcsekclnqykmqifchetqahl---	Dm_FBpp0087170	
				-	....	Sc	
116	1	CI	ENSP00000345505<br>ENSG00000143061	585	gvtysdsfdlQCIIKPHYpawvpvsvtw	Hs_ENSP00000345505	NP_001007238 Immunoglobulin superfamily member 3 Precursor (EWI-3)
				560	gvtyndsfdlQCIIKPHYlsrvpvsvtw	Bt_ENSBTAP00000006374	
				552	gvtysdsfdlQCIIKPHYparvpvsvtw	Rn_ENSRNOP00000036095	
				565	gvtysdsfdlQCIIKPHYparvpvsvtw	Mm_ENSMUSP00000048900	
				550	gvtynesfdlQCIIKPHYpswvpvsvtw	Gg_ENSGALP00000024898	
				-	....	Xt	
				570	tapidspvkllcive---rpevslavrw	Dr_ENSDARP00000076719	
				-	....	Ce	
				-	....	Dm	
				-	....	Sc	
117	1	CI	ENSP00000318902<br>ENSG00000114861	140	lqqqqalmlqQQQLQEFYkkqqeqlqlq	Hs_ENSP00000318902	NP_116071 Forkhead box protein P1
				139	lqqqqalmlqQQQLQEFYkkqqeqlqlq	Bt_ENSBTAP00000021993	
				171	lqqqqalmlqQQQLQEFYkkqqeqlqlq	Rn_ENSRNOP00000013271	
				170	lqqqqalmlqQQQLQEFYkkqqeqlqlq	Mm_ENSMUSP00000108950	
				169	lqqqqalmlqQQQLQEFYkkqqeqlqlq	Gg_ENSGALP00000012591	
				170	lqqqqalmlqQQQLQEFYkkqqeqlqlq	Xt_ENSXETP00000018629	
				130	lqqqqalmlqQQ-LQEFYkkqqeqlhlq	Dr_ENSDARP00000064937	
				-	....	Ce	
				-	....	Dm	
				-	....	Sc	
118	1	CI	ENSP00000356674<br>ENSG00000185278	441	chvcgksfpfQAILNQHFrknhpgcipl	Hs_ENSP00000356674	NP_001116242 Zinc finger and BTB domain-containing protein 37
				443	chvcgksfpfQAILNQHFrknhpgcipl	Bt_ENSBTAP00000038286	
				443	chvcgksfpfQAILNQHFrknhpgcipl	Rn_ENSRNOP00000036623	
				443	chvcgksfpfQAILNQHFrknhpgcipl	Mm_ENSMUSP00000065974	
				443	chvcgksfpfQAILNQHFrknhpgcvpl	Gg_ENSGALP00000007304	
				443	chvcgksfpfQAILNQHFrknhpgcvpf	Xt_ENSXETP00000043300	
				447	chvcgksfpfQAILNQHFrknhpgcapq	Dr_ENSDARP00000062628	
				-	....	Ce	
				-	....	Dm	
				-	....	Sc	

119	1	CI	ENSP00000356224<br>ENSG00000131018	2404<br>2361<br>2471<br>2402<br>2386<br>-<br>1761<br>2317<br>-<br>-	qlcsktqaslQESLEKHFseesmqefqe<br>qscsktqaslQDSLEKHFn-esmqlfqe<br>qlcsqtqasiQDSLEKHFs-gsmkefqe<br>qlcsqtqariQDSLEKHFs-gsmkefqe<br>qlcsrtqanmQECLEKHFl-ysmqefqe<br>............................<br>qrrsqilgllqeallqrfn-ekahafhg<br>mpistldlnmlqgiednln---------<br>............................<br>............................	Hs_ENSP00000356224<br>Bt_ENSBTAP00000012328<br>Rn_ENSRNOP00000050794<br>Mm_ENSMUSP00000051825<br>Gg_ENSGALP00000021957<br>Xt<br>Dr_ENSDARP00000025366<br>Ce_CE33588<br>Dm<br>Sc	Nesprin-1 (Nuclear envelope spectrin repeat protein 1)(Synaptic nuclear envelope protein 1)(Syne-1)(Myocyte nuclear envelope protein 1)(Myne-1)(Enaptin)
120	1	CI	ENSP00000321424<br>ENSG00000131183	386<br>385<br>384<br>384<br>395<br>-<br>301<br>-<br>-<br>-	llkgqvakviQKVINTDFpapftwvtgy<br>llkgqvakviQKVINTDFptpftwatgy<br>llkgqvmssrrsstqsyfpapftwvtgy<br>llkgqvanviQKVINTDFpapftwvtgy<br>llkgqvakailscamadlphpfswltgy<br>............................<br>llqgqvakaiQKVINTDFpfpfswltgy<br>............................<br>............................<br>............................	Hs_ENSP00000321424<br>Bt_ENSBTAP00000027121<br>Rn_ENSRNOP00000044835<br>Mm_ENSMUSP00000059138<br>Gg_ENSGALP00000004850<br>Xt<br>Dr_ENSDARP00000091302<br>Ce<br>Dm<br>Sc	NP_003043 Sodium-dependent phosphate transport protein 2A (Sodium-phosphate transport protein 2A)(Na(+)-dependent phosphate cotransporter 2A)(Sodium/phosphate cotransporter 2A)(Na(+)/Pi cotransporter 2A)(NaPi-2a)(NaPi-3)(Solute carrier family 34 member 1)
120	2	CI	ENSP00000321424<br>ENSG00000131183	472<br>471<br>470<br>470<br>481<br>-<br>387<br>-<br>-<br>-	spreklssafQIALCHFFfnisgillwy<br>spreklssafQIALCHFFfnisgillwy<br>spreklsssfQIALCHFFfnisgillwy<br>spreklsssfQIALCHFFfnisgillwy<br>spgdklassfQIALCHFFfnisgillwy<br>............................<br>spgdklaaafqvalchfifnilgillwy<br>............................<br>............................<br>............................	Hs_ENSP00000321424<br>Bt_ENSBTAP00000027121<br>Rn_ENSRNOP00000044835<br>Mm_ENSMUSP00000059138<br>Gg_ENSGALP00000004850<br>Xt<br>Dr_ENSDARP00000091302<br>Ce<br>Dm<br>Sc	NP_003043 Sodium-dependent phosphate transport protein 2A (Sodium-phosphate transport protein 2A)(Na(+)-dependent phosphate cotransporter 2A)(Sodium/phosphate cotransporter 2A)(Na(+)/Pi cotransporter 2A)(NaPi-2a)(NaPi-3)(Solute carrier family 34 member 1)
121	1	CI	ENSP00000246957<br>ENSG00000126602	419<br>418<br>421<br>421<br>419<br>-<br>381<br>382<br>407<br>-	alirklrdvlQQRLIKFFidqskkdaek<br>alirklqgvlQQRLIKFFtdqskkdaek<br>alirklrdvlQQRLIKFFidqskkdaek<br>alirklrdvlQQRLIKFFidqskkdaek<br>alirklrdvlQKRLIKFFvdqskkdpek<br>............................<br>alirklrdvlqqrvirflldqskkdpek<br>pvlrklrkiitdkilgslqsemkkdpvk<br>slirklssvistrvirflqerskkqpee<br>............................	Hs_ENSP00000246957<br>Bt_ENSBTAP00000026786<br>Rn_ENSRNOP00000008966<br>Mm_ENSMUSP00000006137<br>Gg_ENSGALP00000012445<br>Xt<br>Dr_ENSDARP00000095645<br>Ce_CE39621<br>Dm_FBpp0085482<br>Sc	NP_057376 Heat shock protein 75 kDa, mitochondrial Precursor (HSP 75)(Tumor necrosis factor type 1 receptor-associated protein)(TRAP-1)(TNFR-associated protein 1)
122	1	C	ENSP00000007633<br>ENSG00000006534	241<br>242<br>242<br>242<br>244<br>242<br>242<br>-<br>307<br>-	vawfryfnagqtcvapdyvlcspemqer<br>vaffrcfnagqtcvapdyvlcspemqaq<br>vawfryfnagqtcvapdyvlcsqemqer<br>vawfryfnagqtcvapdyilcsqemqer<br>vvwgrffnagqtciapeyvlcsvemqek<br>iswsryfnagQTCLAPDYvlctertrdr<br>lvwakffnsgqscvapdyvlctdevkem<br>............................<br>ilwgklincgQTCIAPDYilcskevqek<br>............................	Hs_ENSP00000007633<br>Bt_ENSBTAP00000016003<br>Rn_ENSRNOP00000023789<br>Mm_ENSMUSP00000056276<br>Gg_ENSGALP00000005509<br>Xt_ENSXETP00000027210<br>Dr_ENSDARP00000002746<br>Ce<br>Dm_FBpp0289707<br>Sc	NP_000685 Aldehyde dehydrogenase family 3 member B1 (EC 1.2.1.5)(Aldehyde dehydrogenase 7)
122	2	CI	ENSP00000007633<br>ENSG00000006534	264<br>265<br>265<br>265<br>267<br>265<br>265<br>-<br>330<br>-	emqerllpalQSTITRFYgddpqsspnl<br>emqaqlvpalQSAITRFYgddpqsspnl<br>emqerlvpalQNAITRFYgdnpqtspnl<br>emqerlvpalQNAITRFYgdnpqtspnl<br>emqeklipalreaitefygseprnspdf<br>rtrdrlvkalkatvrefygqdpkqspdl<br>evkemllpfmkealesfygsepqespdy<br>............................<br>evqekfiveakdvlkewygeniqsspdl<br>............................	Hs_ENSP00000007633<br>Bt_ENSBTAP00000016003<br>Rn_ENSRNOP00000023789<br>Mm_ENSMUSP00000056276<br>Gg_ENSGALP00000005509<br>Xt_ENSXETP00000027210<br>Dr_ENSDARP00000002746<br>Ce<br>Dm_FBpp0289707<br>Sc	NP_000685 Aldehyde dehydrogenase family 3 member B1 (EC 1.2.1.5)(Aldehyde dehydrogenase 7)
123	1	CI	ENSP00000205890<br>ENSG00000091536	1910<br>1874<br>1895<br>1894<br>714<br>-	hvlnlaaltlQRCLRGFFikrrfrslrh<br>hvlclaavtlQRCLRGFFvqrrfrslrr<br>rvlnraaltlQRYLRGFFtqrrfrslrq<br>rvqnraaltlQRYLRGFFiqrhfrslrr<br>rahhlaaltlqryartffikkrfrslrr<br>............................	Hs_ENSP00000205890<br>Bt_ENSBTAP00000034896<br>Rn_ENSRNOP00000023018<br>Mm_ENSMUSP00000071777<br>Gg_ENSGALP00000007959<br>Xt	NP_057323 Myosin-XV (Unconventional myosin-15)

				-<br>-<br>851<br>-	............................<br>............................<br>erlrraavsvqrhvrgmlvrrqlarrqa<br>............................	Dr<br>Ce<br>Dm_FBpp0271775<br>Sc	
124	1	CI	ENSP00000370695<br>ENSG00000090989	530<br>536<br>494<br>537<br>538<br>532<br>531<br>490<br>517<br>-	seleplclaeQDFISKFFklqqhqsmpg<br>adhksyclwqHDFSRRTFlrqqiisedy<br>seleplclaeQDFISKFFklqqhqnlsa<br>seleplclaeQDFISKFFklqqhqnmsa<br>seleplclaeQDFISKFFklqqhqsisg<br>seleplclaeQDFISKFFnlsqhqnmsr<br>seleplclaeQDFISKFFklqqiptlaq<br>aelsavidaeqkfvvrffhinsellaqf<br>aelepialqeqlfcinffqmdvisptk<br>............................	Hs_ENSP00000370695<br>Bt_ENSBTAP00000043609<br>Rn_ENSRNOP00000056565<br>Mm_ENSMUSP00000109121<br>Gg_ENSGALP00000022336<br>Xt_ENSXETP00000055965<br>Dr_ENSDARP00000063153<br>Ce_CE10836<br>Dm_FBpp0074993<br>Sc	NP_001020095 Exocyst complex component 1 (Exocyst complex component Sec3)
125	1	CI	ENSP00000299466<br>ENSG00000151514	691<br>676<br>677<br>704<br>716<br>736<br>704<br>317<br>-<br>-	cvichrvlscQSALKMHYrthtgerpfk<br>cvichrvlscQSALKMHYrthtgerpfk<br>cvichrvlscQSALKMHYrthtgerpfk<br>cvichrvlscQSALKMHYrthtgerpfk<br>cvichrvlscQSALKMHYrthtgerpfk<br>cvichrvlscQSALKMHYrthtgerpfk<br>cvichrvlscQSALKMHYrihtgerpfk<br>cilcrrvlscksalqmhyrthtgerpfk<br>............................<br>............................	Hs_ENSP00000299466<br>Bt_ENSBTAP00000014214<br>Rn_ENSRNOP00000045052<br>Mm_ENSMUSP00000025457<br>Gg_ENSGALP00000020631<br>Xt_ENSXETP00000001885<br>Dr_ENSDARP00000076069<br>Ce_CE17673<br>Dm<br>Sc	NP_741996 Sal-like protein 3 (Zinc finger protein SALL3) (hSALL3)
126	1	C	ENSP00000347741<br>ENSG00000196954	70<br>70<br>70<br>70<br>-<br>-<br>-<br>-<br>-<br>-	smqekqrmagQMLLQTFFnidqispnkk<br>sirqknqeagqvfvqtflnidknstsik<br>vlkrkhnqvgkmllqtflnvdsgshhge<br>amkkkhskvgemllqtffsvdpgshhge<br>............................<br>............................<br>............................<br>............................<br>............................<br>............................	Hs_ENSP00000347741<br>Bt_ENSBTAP00000027820<br>Rn_ENSRNOP00000058685<br>Mm_ENSMUSP00000027012<br>Gg<br>Xt<br>Dr<br>Ce<br>Dm<br>Sc	NP_150649 Caspase-4 Precursor (CASP-4)(EC 3.4.22.57)(ICH-2 protease)(TX protease)(ICE(rel)-II)
127	1	CI	ENSP00000237247<br>ENSG00000118473	390<br>382<br>385<br>409<br>341<br>357<br>-<br>-<br>-<br>-	leevqkkvaeQTFIKDDYle-tisspkd<br>leevqkkvaeQNFIKDDYle-tisspkd<br>leevqkkvaeQTFIKDDYle-tlsspke<br>leevqkkvaeQTFIKDDYle-tlsspke<br>ldellkkaleqpyakeenaepavaspke<br>leevqkkimeqvfikddvpe-tvaspkd<br>............................<br>............................<br>............................<br>............................	Hs_ENSP00000237247<br>Bt_ENSBTAP00000030815<br>Rn_ENSRNOP00000008712<br>Mm_ENSMUSP00000099836<br>Gg_ENSGALP00000018081<br>Xt_ENSXETP00000054460<br>Dr<br>Ce<br>Dm<br>Sc	SH3-containing GRB2-like protein 3-interacting protein 1 (Endophilin-3-interacting protein)
128	1	CI	ENSP00000381786<br>ENSG00000056998	261<br>165<br>-<br>-<br>166<br>-<br>-<br>-<br>-<br>189	mehgsfdgadQGLLNSFFrn-wsttdih<br>tdhgsfdgadQGLLNSFFsn-wstadiq<br>............................<br>............................<br>aehgsfdggdQGLLNSFFsn-watadig<br>............................<br>............................<br>............................<br>............................<br>fentsidgsdQGILNQFFnqncctdelv	Hs_ENSP00000381786<br>Bt_ENSBTAP00000017744<br>Rn<br>Mm<br>Gg_ENSGALP00000026833<br>Xt<br>Dr<br>Ce<br>Dm<br>Sc_YKR058W	NP_001073324 Glycogenin-2 (GN-2)(GN2)(EC 2.4.1.186)
129	1	C	ENSP00000381747<br>ENSG00000107745	348<br>350<br>-<br>354<br>350<br>-<br>169<br>429<br>396<br>-	gvqskkltamQRQLKKHFke--gkgltf<br>gvqskkltamQKQLKKHFke--gkgltf<br>............................<br>gvqskkltamQRQLKKHFkd--gkgltf<br>gvqskkltvmlkqlkkhfqd--gegltf<br>............................<br>gvqsrklkqmqknlkrmfkd--aqgitf<br>qinekkqkhmlkrvkrrfkgenlkgisf<br>gyplkkkqkklkrvkrrfrdh-gkgisk<br>............................	Hs_ENSP00000381747<br>Bt_ENSBTAP00000007636<br>Rn<br>Mm_ENSMUSP00000020311<br>Gg_ENSGALP00000006962<br>Xt<br>Dr_ENSDARP00000086953<br>Ce_CE28375<br>Dm_FBpp0111941<br>Sc	NP_006068 Calcium-binding atopy-related autoantigen 1 (Atopy-related autoantigen CALC)(ara CALC)(Allergen Hom s 4)
130	1	CI	ENSP00000258739<br>ENSG00000136240	187<br>187<br>187<br>187<br>187<br>187	dliavvagvvQTILYCDFfylyitkvlk<br>dliavvagvvQTILYCDFfylyitkvlk<br>dliavvagvvQTILYCDFfylyitkaln<br>dliavvagvvQTILYCDFfylyitkvlk<br>dliavvagvvQTVLYCDFfylyvtkvlk<br>dliaivagvvQTILYCDFfylyvtkvlk	Hs_ENSP00000258739<br>Bt_ENSBTAP00000012175<br>Rn_ENSRNOP00000057445<br>Mm_ENSMUSP00000106359<br>Gg_ENSGALP00000011467<br>Xt_ENSXETP00000023270	NP_006845 ER lumen protein retaining receptor 2 (KDEL endoplasmic reticulum protein retention receptor 2)(KDEL

				187	dmiaivagvvQTILYCDFfylyvtkvlk	Dr_ENSDARP00000011420	receptor 2)(ERD2-like protein 1)(ELP-1)
				-	....	Ce	
				-	....	Dm	
				-	....	Sc	
131	1	CI	ENSP00000354368<br>ENSG00000096155	782	hghfqplqrlQPQLRSFFhqhylggqep	Hs_ENSP00000354368	NP_004630 Large proline-rich protein BAT3 (HLA-B-associated transcript 3)(Protein G3)
				778	hghfqplqrlQPQLRSFFhqhylggqep	Bt_ENSBTAP00000035657	
				796	hghfqplqrlQPQLRSFFhqhylggqep	Rn_ENSRNOP00000041318	
				804	hghfqplqrlQPQLRSFFhqhylggqep	Mm_ENSMUSP00000025250	
				-	....	Gg	
				-	....	Xt	
				-	....	Dr	
				-	....	Ce	
				-	....	Dm	
				-	....	Sc	
132	1	CI	ENSP00000348477<br>ENSG00000133195	10	-mlqghssvfQALLGTFFtwgmtaagaa	Hs_ENSP00000348477	Zinc transporter ZIP11 (Zrt- and Irt-like protein 11)(ZIP-11)(Solute carrier family 39 member 11)
				10	-mlhghspvsQALLGTFFtwgltaagrl	Bt_ENSBTAP00000050421	
				10	-mlqgyssvsQALLGTFFtwamtaagaa	Rn_ENSRNOP00000050117	
				10	-mlqgyssvvQALLGTFFtwamtaagaa	Mm_ENSMUSP00000037331	
				10	-mipgqgpvlqavlgtlltwgltaagsa	Gg_ENSGALP00000007021	
				10	-miegyspvlqsllgtlltwgltaagsa	Xt_ENSXETP00000021966	
				-	....	Dr	
				10	-mirginpiyqallaatftwgvtalgas	Ce_CE11422	
				10	-mipgygpvtqallgtlltwgltaagaa	Dm_FBpp0087144	
				-	....	Sc	
133	1	CI	ENSP00000386901<br>ENSG00000138395	328	kgfprdrvsaQEALVHDYfsalpsqlyq	Hs_ENSP00000386901	NP_631897 Serine/threonine-protein kinase PFTAIRE-2 (EC 2.7.11.22)(Serine/threonine-protein kinase ALS2CR7)(Amyotrophic lateral sclerosis 2 chromosomal region candidate gene 7 protein)
				295	----------------------------	Bt_ENSBTAP00000046377	
				328	kgfprdrvsaQEALVHDYfsvlpsqlyq	Rn_ENSRNOP00000038070	
				377	kgfprdrvsaQEALVHDYfsvlpsqlyq	Mm_ENSMUSP00000109886	
				329	tafppgrisaqdallhgffsplppqlyq	Gg_ENSGALP00000013714	
				331	kgcprerisaedallhkffstlpcqlhq	Xt_ENSXETP00000007505	
				346	mmnpkdrisaqdallhpyfntlppplmh	Dr_ENSDARP00000075572	
				-	....	Ce	
				-	....	Dm	
				-	....	Sc	
134	1	CI	ENSP00000351732<br>ENSG00000149488	737	sgknrmydvlQETIENDFptflgkifaf	Hs_ENSP00000351732	NP_542789 Transmembrane channel-like protein 2 (Transmembrane cochlear-expressed protein 2)
				709	sgknrmydviqetvendfptflgkifaf	Bt_ENSBTAP00000025041	
				717	sgknrmydvlhetiendfpkflgkifaf	Rn_ENSRNOP00000009323	
				719	sgknrmydvlhetiendfpkflgkifaf	Mm_ENSMUSP00000077139	
				680	sgkkrmydviQETIELDFplfvakifgy	Gg_ENSGALP00000019465	
				715	sgknrmfdviQETMQNDFppfvgkiiss	Xt_ENSXETP00000031503	
				748	sgkprmfdviQETLETDFpawfskvfsy	Dr_ENSDARP00000073306	
				-	....	Ce	
				-	....	Dm	
				-	....	Sc	
135	1	CI	ENSP00000297151<br>ENSG00000164609	156	niapd--ehvQPQLMFDYdgkrdrwngy	Hs_ENSP00000297151	NP_006416 Pre-mRNA-splicing factor SLU7 (hSlu7)
				156	niapd--ehvQPQLMFDYdgkrdrwngy	Bt_ENSBTAP00000011131	
				156	niapd--ehiQPQLMFDYdgkrdrwngy	Rn_ENSRNOP00000056354	
				156	niapd--ehvQPQLMFDYdgkrdrwngy	Mm_ENSMUSP00000020681	
				156	niapd--ehvQPQLMFDYdgkrdrwngy	Gg_ENSGALP00000002277	
				152	niapd--ehdQPQLMLDYdgkrdrwngy	Xt_ENSXETP00000036042	
				197	giapd--ehqQVQLSMDYdgkrdrwngy	Dr_ENSDARP00000007467	
				151	niaed--dyvqpnltlgfdakrdrwngy	Ce_CE06116	
				154	ivvhd--ehlvneaavnydekrdrwssy	Dm_FBpp0084787	
				160	nsqknngtvlvratdddwdsrkdrwygy	Sc_YDR088C	
136	1	CI	ENSP00000320252<br>ENSG00000112249	189	fdelpingetQKTISLDYkkflnehlqe	Hs_ENSP00000320252	NP_006819 Activating signal cointegrator 1 complex subunit 3 (EC 3.6.1.-)(ASC-1 complex subunit p200)(Trip4 complex subunit p200)(Helicase, ATP binding 1)
				189	fdelpingesQKTISLDYkkfltdhlqd	Bt_ENSBTAP00000027294	
				189	fdelpvngeaQKTISLDYkkflneqfqe	Rn_ENSRNOP00000053848	
				189	fdelpingesQKTISLDYkkflneqfqe	Mm_ENSMUSP00000036726	
				0	----------------------------	Gg_ENSGALP00000024889	
				-	....	Xt	
				-	....	Dr	
				49	------------------------eape	Ce_CE43325	
				188	wmdislltdlapdallknrtvnkfsmkh	Dm_FBpp0082601	
				71	-----------------------eefkd	Sc_YGR271W	
137	1	CI	ENSP00000386538<br>ENSG00000169221	588	etmeqlrnlpQQCMQEDFlvhevtnlpv	Hs_ENSP00000386538	NP_056342 TBC1 domain family member 10B
				447	etmeqlrnlpQQCMQEDFlvhevtnlqv	Bt_ENSBTAP00000020595	
				571	etmeqlrnlpQQCMQEDFlvhevttlpv	Rn_ENSRNOP00000023705	
				574	etmeqlrnlpQQCMQEDFlvhevtnlpv	Mm_ENSMUSP00000113307	
				-	....	Gg	
				315	etmeklrslppqymnedfllpexxslpi	Xt_ENSXETP00000041022	

				-<br>-<br>-<br>-	............................<br>............................<br>............................<br>............................	Dr<br>Ce<br>Dm<br>Sc	
138	1	CI	ENSP00000372857<br>ENSG00000114670	141<br>-<br>-<br>142<br>141<br>-<br>-<br>-<br>-<br>-	penqiiewfiQLLLGVDYmherrilhrd<br>............................<br>............................<br>aenqivewfiQLLLGVDYmherrilhrd<br>tqrqiiewfiqlllgvnymherrilhrd<br>............................<br>............................<br>............................<br>............................<br>............................	Hs_ENSP00000372857<br>Bt<br>Rn<br>Mm_ENSMUSP00000038611<br>Gg_ENSGALP00000018912<br>Xt<br>Dr<br>Ce<br>Dm<br>Sc	NP_079076 Serine/threonine-protein kinase Nek11 (EC 2.7.11.1)(Never in mitosis A-related kinase 11)(NimA-related protein kinase 11)
139	1	CI	ENSP00000256015<br>ENSG00000133639	45<br>45<br>45<br>45<br>45<br>44<br>44<br>33<br>-<br>-	rqlqtfsqslQELLAEHYkhhwfpekpc<br>rqlqtfsqslQELLAEHYkhhwfpekpc<br>rqlqtfsqslQELLAEHYkhhwfpekpc<br>rqlqtfsqslQELLAEHYkhhwfpekpc<br>rqlqtfsqslQELLAEHYkhhwfpekpc<br>ldlqtfnqslQELLADHYkhhwfpekps<br>rqlqtfsqtlqdilaeqykhhwfpdrpn<br>rpvgifgaelgnylvshfsstwdvhhpk<br>............................<br>............................	Hs_ENSP00000256015<br>Bt_ENSBTAP00000027091<br>Rn_ENSRNOP00000005910<br>Mm_ENSMUSP00000038863<br>Gg_ENSGALP00000018378<br>Xt_ENSXETP00000056004<br>Dr_ENSDARP00000031665<br>Ce_CE07874<br>Dm<br>Sc	NP_001722 Protein BTG1 (B-cell translocation gene 1 protein)
140	1	CI	ENSP00000349909<br>ENSG00000163629	532<br>535<br>523<br>523<br>541<br>532<br>-<br>-<br>-<br>-	reialetamtQRKLRNFFgpefvkmtie<br>reialetamtQRKLRNFFgpefvkmtie<br>remaletamtQRKLRNFFgpefvkttve<br>remaletamtQRKLRNFFgpefvkmtve<br>reialetamtQRKLRNFFgpefvkmtie<br>reiamensfaQINLRNFFgpefikmatg<br>............................<br>............................<br>............................<br>............................	Hs_ENSP00000349909<br>Bt_ENSBTAP00000050778<br>Rn_ENSRNOP00000057876<br>Mm_ENSMUSP00000092132<br>Gg_ENSGALP00000018028<br>Xt_ENSXETP00000027463<br>Dr<br>Ce<br>Dm<br>Sc	NP_006255 Tyrosine-protein phosphatase non-receptor type 13 (EC 3.1.3.48)(Protein-tyrosine phosphatase 1E)(PTP-E1)(hPTPE1)(PTP-BAS)(Protein-tyrosine phosphatase PTPL1)(Fas-associated protein-tyrosine phosphatase 1)(FAP-1)
140	2	C	ENSP00000349909<br>ENSG00000163629	1258<br>1260<br>1248<br>1248<br>1270<br>1250<br>-<br>-<br>-<br>-	dsrtesaslsqsqvngffashlgdqtwq<br>dsrtesaslsqsqvngffashaadrswq<br>dsrtesaslsqshvngfftshlgdrgwq<br>dsrtesaslsqsqvngffashlgdrgwq<br>dsrtesaslsqsqtascfgrrasgrsqq<br>dsrtesaslsnsqiksptnsakehiakq<br>............................<br>............................<br>............................<br>............................	Hs_ENSP00000349909<br>Bt_ENSBTAP00000050778<br>Rn_ENSRNOP00000057876<br>Mm_ENSMUSP00000092132<br>Gg_ENSGALP00000018028<br>Xt_ENSXETP00000027463<br>Dr<br>Ce<br>Dm<br>Sc	NP_006255 Tyrosine-protein phosphatase non-receptor type 13 (EC 3.1.3.48)(Protein-tyrosine phosphatase 1E)(PTP-E1)(hPTPE1)(PTP-BAS)(Protein-tyrosine phosphatase PTPL1)(Fas-associated protein-tyrosine phosphatase 1)(FAP-1)
141	1	CI	ENSP00000287908<br>ENSG00000157214	304<br>304<br>303<br>303<br>304<br>293<br>274<br>-<br>-<br>-	wletwlqcrkQLGLLSFFfamvhvaysl<br>wletwlqcrkQLGLLSFFfasvhvaysl<br>wldtwlqcrkQLGLLSFFfavvhvaysl<br>wldtwlqcrkQLGLLSFFfavvhvaysl<br>wldnwlqcrkQLGLLSFFfavvhvaysl<br>wlegwlecrkQLGLLSFFfacvhvvysm<br>wlegwlesrkQLGLLSFFfgcihvlysl<br>............................<br>............................<br>............................	Hs_ENSP00000287908<br>Bt_ENSBTAP00000004557<br>Rn_ENSRNOP00000007977<br>Mm_ENSMUSP00000015797<br>Gg_ENSGALP00000014653<br>Xt_ENSXETP00000016208<br>Dr_ENSDARP00000085788<br>Ce<br>Dm<br>Sc	NP_001035755 Metalloreductase STEAP2 (EC 1.16.1.-)(Six-transmembrane epithelial antigen of prostate 2)(SixTransMembrane protein of prostate 1)(Prostate cancer-associated protein 1)(Protein upregulated in metastatic prostate cancer)(PUMPCn)
142	1	C	ENSP00000300275<br>ENSG00000146839	112<br>112<br>-<br>118<br>74<br>77<br>0<br>-<br>-<br>-	arllspdlweQGPLCVHFahhmfglswg<br>arlrsppiweqgplcvrfayfmfglswg<br>............................<br>arlrspdiweQGPLCVHFafhmfglswg<br>nrlespdivvsekicidfwyymfgsedr<br>vrldspkldisndvcvefwyhmsgsetq<br>----------------------------<br>............................<br>............................<br>............................	Hs_ENSP00000300275<br>Bt_ENSBTAP00000041534<br>Rn<br>Mm_ENSMUSP00000114068<br>Gg_ENSGALP00000011650<br>Xt_ENSXETP00000004563<br>Dr_ENSDARP00000046199<br>Ce<br>Dm<br>Sc	NP_003377 Zonadhesin Precursor
143	1	CI	ENSP00000304704<br>ENSG00000172409	181<br>181<br>181<br>181<br>181	-------fsiQAPLVYHFgsttpgtnik<br>-------fsiQAPLVYHFgsttpgtnik<br>-------fsiQAPLVYHFgsttpgtnik<br>-------fsiQAPLVYHFgsttpgtnik<br>-------fslQAPLVYHFgsttpgtnik	Hs_ENSP00000304704<br>Bt_ENSBTAP00000008507<br>Rn_ENSRNOP00000009726<br>Mm_ENSMUSP00000028475<br>Gg_ENSGALP00000011907	NP_006822 Pre-mRNA cleavage complex II protein Clp1

				191<br>179<br>181<br>176<br>203	-------fsaQAPLVYHFgsttpgtnik<br>-------fsvQAPLVFHFgsttpgtnik<br>-------fernqpivfnfghtspsanls<br>-------faktaplvyhfghkspsgnsv<br>ltsgatllhnkqpmvknfglerinenkd	Xt_ENSXETP00000015427<br>Dr_ENSDARP00000087664<br>Ce_CE17941<br>Dm_FBpp0086820<br>Sc_YOR250C	
144	1	C	ENSP00000290422<br>ENSG00000159266	399<br>443<br>-<br>-<br>-<br>-<br>-<br>337<br>-<br>-	rmhligrsreQLKLLGDYlglcrsgalk<br>----------------------------<br>............................<br>............................<br>............................<br>............................<br>............................<br>aleetrklreklqlvsmylftcresvse<br>............................<br>............................	Hs_ENSP00000290422<br>Bt_ENSBTAP00000027135<br>Rn<br>Mm<br>Gg<br>Xt<br>Dr<br>Ce_CE32757<br>Dm<br>Sc	Pleckstrin homology domain-containing family M member 4
145	1	C	ENSP00000373952<br>ENSG00000187741	956<br>953<br>958<br>950<br>928<br>-<br>855<br>-<br>-<br>-	lsdterqdfhQWAIHEHFlpessasggc<br>lsdterqdfqQWAIHQHFlp-psatggc<br>lsgterhdfhqwaiyerylpapsamggc<br>lsdmerhdfhqwaiyerylpaptalggc<br>lsaserqdfhywaiyqwylpapsasggc<br>............................<br>lataqrnhshhweylsnhmatvslqh-c<br>............................<br>............................<br>............................	Hs_ENSP00000373952<br>Bt_ENSBTAP00000002478<br>Rn_ENSRNOP00000022659<br>Mm_ENSMUSP00000045217<br>Gg_ENSGALP00000000705<br>Xt<br>Dr_ENSDARP00000005795<br>Ce<br>Dm<br>Sc	NP_001018122 Fanconi anemia group A protein (Protein FACA)
146	1	CI	ENSP00000277549<br>ENSG00000148408	225<br>0<br>225<br>225<br>218<br>-<br>219<br>-<br>-<br>-	simkamvpllQIGLLLFFailmfaiigl<br>----------------------------<br>simkamvpllQIGLLLFFailmfaiigl<br>simkamvpllQIGLLLFFailmfaiigl<br>simkamvpllQIGLLLFFaivmfaiigl<br>............................<br>simkamvpllQIGLLLFFailmfaiigl<br>............................<br>............................<br>............................	Hs_ENSP00000277549<br>Bt_ENSBTAP00000045932<br>Rn_ENSRNOP00000006162<br>Mm_ENSMUSP00000091064<br>Gg_ENSGALP00000013765<br>Xt<br>Dr_ENSDARP00000047718<br>Ce<br>Dm<br>Sc	Voltage-dependent N-type calcium channel subunit alpha-1B (Voltage-gated calcium channel subunit alpha Cav2.2) (Calcium channel, L type, alpha-1 polypeptide isoform 5) (Brain calcium channel III) (BIII)
147	1	CI	ENSP00000273859<br>ENSG00000145246	1080<br>1079<br>1080<br>1076<br>1070<br>-<br>-<br>1105<br>1221<br>1197	gigvsgqegmQAVMASDFavsqfkhlsk<br>gigisgqegmQAVMASDFavsqfrhlsk<br>gigvsgqegmQAVMASDFaisqfrhlsk<br>gigvsgqegmQAVMASDFaisqfrhlsk<br>gvgimgqegmQAVMASDFaisqfrhlrk<br>............................<br>............................<br>giglsgqegmQAVMSSDFamarfrflsn<br>gvgisgqegmQAVMAADFtlprfryler<br>gvgiageegrQAVMCSDYaigqfryvtr	Hs_ENSP00000273859<br>Bt_ENSBTAP00000000601<br>Rn_ENSRNOP00000003146<br>Mm_ENSMUSP00000092300<br>Gg_ENSGALP00000022913<br>Xt<br>Dr<br>Ce_CE43310<br>Dm_FBpp0088794<br>Sc_YDR093W	NP_065186 Probable phospholipid-transporting ATPase VD (ATPVD)(EC 3.6.3.1)
148	1	CI	ENSP00000373586<br>ENSG00000172478	14<br>19<br>19<br>19<br>19<br>-<br>-<br>-<br>-<br>-	palptsamavQVPLWHHYlqairsreap<br>pravpaamavQVPLWHHYlqalrsraas<br>pcvfapdmaeQVPLWHHYllaiqsresp<br>pcvfapdmaeQVPLWHHYllaiqsresp<br>rsraapamaaggcrwrsclevvasrqgq<br>............................<br>............................<br>............................<br>............................<br>............................	Hs_ENSP00000373586<br>Bt_ENSBTAP00000016419<br>Rn_ENSRNOP00000030463<br>Mm_ENSMUSP00000035332<br>Gg_ENSGALP00000009723<br>Xt<br>Dr<br>Ce<br>Dm<br>Sc	NP_001078906 Uncharacterized protein C2orf54
149	1	CI	ENSP00000363100<br>ENSG00000147130	747<br>747<br>747<br>747<br>766<br>508<br>-<br>-<br>-<br>-	wrgqirhfcnQQCLLRFYsqqnqpnldt<br>wrgqirhfcnQQCLLRFYsqqnqpnldt<br>wrgqirhfcnQQCLLRFYsqqnqpnldt<br>wrgqirhfcnQQCLLRFYsqqnqpnldt<br>wrgqikhfcnQQCLLRFYnqqnqpnldt<br>wrgeikhfcnQQCLLRFYnqqnqpnldt<br>............................<br>............................<br>............................<br>............................	Hs_ENSP00000363100<br>Bt_ENSBTAP00000011195<br>Rn_ENSRNOP00000004972<br>Mm_ENSMUSP00000068197<br>Gg_ENSGALP00000008859<br>Xt_ENSXETP00000045087<br>Dr<br>Ce<br>Dm<br>Sc	Zinc finger MYM-type protein 3 (Zinc finger protein 261)
150	1	CI	ENSP00000342434<br>ENSG00000009954	664<br>664<br>662<br>665<br>660	vlvillqtllQDEIAEDYgelgmklsei<br>vlvillqtllQDEIAEDYgelgmklsei<br>vlvillqtllQDEIAEDYgelgmklsei<br>vlvillqtllQDEIAEDYgelgmklsei<br>vlvillqtllQDEIAEDYaelgmklsei	Hs_ENSP00000342434<br>Bt_ENSBTAP00000006848<br>Rn_ENSRNOP00000001975<br>Mm_ENSMUSP00000002825<br>Gg_ENSGALP00000039884	NP_115784 Bromodomain adjacent to zinc finger domain protein 1B (Williams-Beuren syndrome chromosomal region

				272<br>663<br>-<br>-<br>591	vlvvllqtllQDEIAEDYgelgmklsei<br>vlvvllqtllqdelaegyseldmplsei<br>............................<br>............................<br>----------kdergegeakqkrnahgy	Xt_ENSXETP00000022743<br>Dr_ENSDARP00000081914<br>Ce<br>Dm<br>Sc_YGL133W	9 protein)(Williams syndrome transcription factor)(hWALP2)
151	1	CI	ENSP00000219837<br>ENSG00000103550	413<br>411<br>268<br>487<br>-<br>-<br>-<br>-<br>-<br>-	algkkaadslQQNLQRDYdramswkysr<br>alskkaadtlQRNLQQDYdralswkysr<br>----------------------------<br>aldkkssemlQQSLQQDYdramswkysh<br>............................<br>............................<br>............................<br>............................<br>............................<br>............................	Hs_ENSP00000219837<br>Bt_ENSBTAP00000037276<br>Rn_ENSRNOP00000021477<br>Mm_ENSMUSP00000111984<br>Gg<br>Xt<br>Dr<br>Ce<br>Dm<br>Sc	NP_001013009 Testis-specific gene 118 protein
152	1	CI	ENSP00000342847<br>ENSG00000188994	1983<br>958<br>1579<br>2105<br>1231<br>2006<br>1691<br>-<br>-<br>-	hqgcfaaftiQQNLILHYqavhksdlpa<br>hqgcfaaftiQQNLILHYqavhksdlpa<br>hqgcfaaftiQQNLILHYqavhksnlpt<br>hqgcfaaftiQQNLILHYqavhksnlpt<br>hqgcfaaftiQQNLILHYqavhksdlpa<br>hqgctaaftiQQNLMLHYqavhks-vsm<br>hegcvaafsiQQNLILHYramhqsecde<br>............................<br>............................<br>............................	Hs_ENSP00000342847<br>Bt_ENSBTAP00000037664<br>Rn_ENSRNOP00000044996<br>Mm_ENSMUSP00000037233<br>Gg_ENSGALP00000025466<br>Xt_ENSXETP00000039125<br>Dr_ENSDARP00000005233<br>Ce<br>Dm<br>Sc	NP_055836 Zinc finger protein 292
153	1	C	ENSP00000367655<br>ENSG00000205038	1240<br>1245<br>1238<br>1238<br>-<br>1199<br>1226<br>-<br>-<br>-	ardafsynclQTPIITDFspkvrtilge<br>aknaysysclqtpvitdfspkvriilge<br>skdsfsysclqtpvvtdfspkertilgt<br>akdsfsysclqtpvvtdfspkertvlgk<br>............................<br>lpssfsynasftpvitaissnrssvlgg<br>fngsftysntmtalitsvspqtttvfgn<br>............................<br>............................<br>............................	Hs_ENSP00000367655<br>Bt_ENSBTAP00000043706<br>Rn_ENSRNOP00000005958<br>Mm_ENSMUSP00000036988<br>Gg<br>Xt_ENSXETP00000039050<br>Dr_ENSDARP00000084832<br>Ce<br>Dm<br>Sc	NP_803875 Fibrocystin-L Precursor (Polycystic kidney and hepatic disease 1-like protein 1)(PKHD1-like protein 1)
154	1	CI	ENSP00000384222<br>ENSG00000151718	96<br>53<br>96<br>96<br>96<br>95<br>-<br>-<br>-<br>-	edprkqwrgeQEKMLKDYlsvaqdalrt<br>edprkqwrreQEKMLKDYlsvaqdalqt<br>edprkqwrgeQEKMLKDYlsvaqdalrt<br>edprkqwrgeQEKMLKDYlsvaqdalrt<br>edprkqwrqeQERMLKDYlmvaqdalst<br>edprklwrneQERMLKDYlmvaqdalst<br>............................<br>............................<br>............................<br>............................	Hs_ENSP00000384222<br>Bt_ENSBTAP00000016172<br>Rn_ENSRNOP00000017966<br>Mm_ENSMUSP00000056121<br>Gg_ENSGALP00000017336<br>Xt_ENSXETP00000030868<br>Dr<br>Ce<br>Dm<br>Sc	NP_079225 Protein WWC2 (WW domain-containing protein 2)(BH-3-only member B)
155	1	C	ENSP00000260453<br>ENSG00000138587	347<br>347<br>346<br>347<br>300<br>331<br>348<br>-<br>-<br>-	keeaekklrkQKEMKQDFeeqmalkelv<br>eeeaeeklrkQKELKQDFmdqmalkeli<br>keeveqrlrkqrdrkqdfkdqmalrevl<br>keeaelrlrrQREMKQDFedqmalkeli<br>maeiekrirqrldlrqmyeeqfalkkia<br>isemekkirqrlemqqtfeeqmafkqiv<br>ieemekrirqrlelqqtqqemmafkklr<br>............................<br>............................<br>............................	Hs_ENSP00000260453<br>Bt_ENSBTAP00000000337<br>Rn_ENSRNOP00000007941<br>Mm_ENSMUSP00000034746<br>Gg_ENSGALP00000000128<br>Xt_ENSXETP00000036083<br>Dr_ENSDARP00000078928<br>Ce<br>Dm<br>Sc	NP_060835 Meiosis-specific nuclear structural protein 1
156	1	CI	ENSP00000254190<br>ENSG00000131873	583<br>-<br>582<br>582<br>-<br>593<br>-<br>-<br>574<br>-	nsdsnpdkakQVELMRDYrikypkadmq<br>............................<br>nsdsnpdkakQVELMRDYrvkypkadmq<br>nsdsnpdkakQVELMRDYrvkypkadmq<br>............................<br>nsdsnpdktrQVELMRDYrvkypkadmq<br>............................<br>............................<br>gspd--elgdhlqllhdlharhvyqqvn<br>............................	Hs_ENSP00000254190<br>Bt<br>Rn_ENSRNOP00000016939<br>Mm_ENSMUSP00000047487<br>Gg<br>Xt_ENSXETP00000040600<br>Dr<br>Ce<br>Dm_FBpp0073880<br>Sc	NP_055733 Chondroitin sulfate synthase 1 (EC 2.4.1.175)(Glucuronosyl-N-acetylgalactosaminyl-proteoglycan 4-beta-N-acetylgalactosaminyltransferase 1)(N-acetylgalactosaminyl-proteoglycan 3-beta-glucuronosyltransferase 1)(EC 2.4.1.226)(Chondroitin glucuronyltransferase II)(N-acetylgalactosaminyltransferase II)

157	1	C	ENSP00000364614<br>ENSG00000183566	37<br>37<br>-<br>-<br>-<br>-<br>-<br>-<br>-<br>-	vssqllndltQGLLRADFlpslqttglq<br>vssqindvltkellsvgflpslqnidlq<br>............................<br>............................<br>............................<br>............................<br>............................<br>............................<br>............................<br>............................	Hs_ENSP00000364614<br>Bt_ENSBTAP00000041945<br>Rn<br>Mm<br>Gg<br>Xt<br>Dr<br>Ce<br>Dm<br>Sc	Latherin Precursor (Breast cancer and salivary gland-expressed protein)
158	1	C	ENSP00000368716<br>ENSG00000172766	377<br>-<br>-<br>377<br>378<br>362<br>-<br>-<br>-<br>387	keppttllwvQYFLAQHFdklgqyslal<br>............................<br>............................<br>keppttliwvQYFLAQHYdklgqyflal<br>reppttllwvryflaqhfdklgqcslal<br>keppttllwvryflaqhfdklgqwslal<br>............................<br>............................<br>............................<br>-qdpipfiwtnyylsqhflflkdfpkaq	Hs_ENSP00000368716<br>Bt<br>Rn<br>Mm_ENSMUSP00000022597<br>Gg_ENSGALP00000027349<br>Xt_ENSXETP00000040998<br>Dr<br>Ce<br>Dm<br>Sc_YDL040C	NP_078837 NMDA receptor-regulated 1-like protein (NARG1-like protein)
158	2	C	ENSP00000368716<br>ENSG00000172766	389<br>-<br>-<br>389<br>390<br>374<br>-<br>-<br>-<br>399	flaqhfdklgQYSLALDYinaaiastpt<br>............................<br>............................<br>flaqhydklgqyflaleyinaviastpt<br>flaqhfdklgQCSLALDYinaaiastpt<br>flaqhfdklgqwslaldrinsaiastpt<br>............................<br>............................<br>............................<br>ylsqhflflkdfpkaqeyidaaldhtpt	Hs_ENSP00000368716<br>Bt<br>Rn<br>Mm_ENSMUSP00000022597<br>Gg_ENSGALP00000027349<br>Xt_ENSXETP00000040998<br>Dr<br>Ce<br>Dm<br>Sc_YDL040C	NP_078837 NMDA receptor-regulated 1-like protein (NARG1-like protein)
159	1	CI	ENSP00000281129<br>ENSG00000100629	175<br>-<br>174<br>174<br>-<br>-<br>-<br>-<br>-<br>-	hqslrdlsseQIRLGDDFnrelsrrsrs<br>............................<br>hqslrdlsseQVRLGDDFnkelfrrsrs<br>hqslrdlsseqvrlgddlnrelfrrsrs<br>............................<br>............................<br>............................<br>............................<br>............................<br>............................	Hs_ENSP00000281129<br>Bt<br>Rn_ENSRNOP00000054567<br>Mm_ENSMUSP00000046574<br>Gg<br>Xt<br>Dr<br>Ce<br>Dm<br>Sc	NP_689659 Uncharacterized protein C14orf145
160	1	C	ENSP00000296350<br>ENSG00000163975	246<br>246<br>246<br>246<br>245<br>240<br>-<br>-<br>266<br>-	tdgktlpswgQALLSQDFellcrdgsra<br>tdgktlpswgQALLSQDFellcqdgtra<br>tdgktlpswgkalmsqdfqllcrdgsra<br>tdgntlpswgkslmsedfqllcrdgsra<br>tdgrtlstwaqqfrskdfqllcrngsta<br>sdgkntdswarevissdyqllcrdgsha<br>............................<br>............................<br>lqttefkniapdt----fellcrdgrra<br>............................	Hs_ENSP00000296350<br>Bt_ENSBTAP00000043824<br>Rn_ENSRNOP00000002373<br>Mm_ENSMUSP00000023464<br>Gg_ENSGALP00000032783<br>Xt_ENSXETP00000041996<br>Dr<br>Ce<br>Dm_FBpp0075698<br>Sc	NP_005920 Melanotransferrin Precursor (Melanoma-associated antigen p97)(CD228 antigen)
161	1	CI	ENSP00000302805<br>ENSG00000170967	306<br>300<br>312<br>312<br>-<br>-<br>-<br>308<br>-<br>-	qriigrvhlaQIQIEGDFlqcsfsiled<br>qrilgrvhlaQIQIEGDFlqcsfsilee<br>qrimgrvhlaQIQIEGDFlqcsfsilee<br>qrimgrvhlaQIQIEGDFlqcsfsilee<br>............................<br>............................<br>............................<br>ekiegkihlcdvkvedahfscpfevmar<br>............................<br>............................	Hs_ENSP00000302805<br>Bt_ENSBTAP00000003750<br>Rn_ENSRNOP00000037523<br>Mm_ENSMUSP00000053223<br>Gg<br>Xt<br>Dr<br>Ce_CE23513<br>Dm<br>Sc	NP_001001711 Protein DDI1 homolog 1
162	1	CI	ENSP00000261965<br>ENSG00000126216	846<br>402<br>841<br>844<br>834<br>845<br>847<br>-<br>840<br>776	fkesipkmcsQLRILTHFyqgivqqflv<br>fresipkmcsQLRILTHFyqgvvqqflv<br>fqdsipkmcsQLRILTHFyqgvvqqflv<br>fqdsipkmcsQLRILTHFyqgvvqqflv<br>fqdsipkmcsQLRILTHFyqgivqqflv<br>fqesipkmrsQLRILTHFyqgiveqflv<br>fkdtipkmcsQLRLLTHFyqgivqeflr<br>............................<br>faekmdiacrgleviatdyekavstflm<br>lnsvyefvkvycnlndigyeifikmnln	Hs_ENSP00000261965<br>Bt_ENSBTAP00000018326<br>Rn_ENSRNOP00000023012<br>Mm_ENSMUSP00000000776<br>Gg_ENSGALP00000027138<br>Xt_ENSXETP00000004033<br>Dr_ENSDARP00000042063<br>Ce<br>Dm_FBpp0073672<br>Sc_YNL126W	NP_006313 Gamma-tubulin complex component 3 (GCP-3) (hGCP3)(Spindle pole body protein Spc98 homolog) (hSpc98)(h104p)

163	1	C	ENSP00000323929 ENSG00000175899	517	thgllvk---QEDMKGHFsisipvksdi	Hs_ENSP00000323929	Alpha-2-macroglobulin Precursor (Alpha-2-M)(C3 and PZP-like alpha-2-macroglobulin domain-containing protein 5)
				517	thtlpve---QGDMQGHFsmsvpvesdi	Bt_ENSBTAP00000006167	
				520	thvlplk---QGQMRGHFsilismetdl	Rn_ENSRNOP00000019346	
				523	thvlpvt---qghkkghfsilismetdl	Mm_ENSMUSP00000032203	
				517	tsildld---kesvngvfilqlpveadf	Gg_ENSGALP00000036406	
				515	khtlalip--nqeaqgnflfdlpvgtnv	Xt_ENSXETP00000052265	
				524	yeklevksssnrvssgtvsfklsvgadv	Dr_ENSDARP00000061097	
				-	....	Ce	
				-	....	Dm	
				-	....	Sc	
164	1	CI	ENSP00000369075 ENSG00000125817	254	agksakpragQAGLPCDYtanskggvtt	Hs_ENSP00000369075	NP_001801 Major centromere autoantigen B (Centromere protein B)(CENP-B)
				-	....	Bt	
				254	agksakpragQGGLPCDYtanskggvtt	Rn_ENSRNOP00000002929	
				254	agksakpragQGGLPCDYtanskggvtt	Mm_ENSMUSP00000086938	
				-	....	Gg	
				-	....	Xt	
				-	....	Dr	
				-	....	Ce	
				131	sirnqk----------------------	Dm_FBpp0078057	
				-	....	Sc	
165	1	CI	ENSP00000361658 ENSG00000095319	94	ldeeqsvqllQCYLQEDYrgtrdsvktv	Hs_ENSP00000361658	NP_056169 Nucleoporin NUP188 homolog (hNup188)
				96	ldeeqsvqllQCYLQEDYrgtrdslktv	Bt_ENSBTAP00000014030	
				94	ldeeqsvqllqcylqeayrgtrdslktv	Rn_ENSRNOP00000032298	
				94	ldeeqsvqllQCYLQEDYrgtrdslktv	Mm_ENSMUSP00000065836	
				94	ldeeqsvqllQCYLQEDYrgtrdslktv	Gg_ENSGALP00000007229	
				87	ldeeqsvellQTYLLYDYrgtqesvkgv	Xt_ENSXETP00000028229	
				94	ldeqqsvqvlQCYLQEDYrgtrntlkav	Dr_ENSDARP00000043430	
				-	....	Ce	
				94	lesaqcweilcyyltqeyrgsaslltql	Dm_FBpp0086987	
				-	....	Sc	
166	1	CI	ENSP00000355353 ENSG00000198569	445	spadrmlsalQVALIHFFfnlagillwy	Hs_ENSP00000355353	NP_543153 Sodium-dependent phosphate transport protein 2C (Sodium-phosphate transport protein 2C)(Na(+)-dependent phosphate cotransporter 2C)(Sodium/phosphate cotransporter 2C)(Na(+)/Pi cotransporter 2C)(NaPi-2c)(Sodium/inorganic phosphate cotransporter IIC)(Solute carrier family 34 member 3)
				-	....	Bt	
				447	spadtllfavQVALIHFFfnlagillwy	Rn_ENSRNOP00000014060	
				447	spadmlifavQVALIHFFfnlagillwy	Mm_ENSMUSP00000006638	
				-	....	Gg	
				-	....	Xt	
				-	....	Dr	
				-	....	Ce	
				-	....	Dm	
				-	....	Sc	
167	1	C	ENSP00000251119 ENSG00000072121	13	hpfgkeeaasQKQLFGFFceclrrgewe	Hs_ENSP00000251119	NP_056161 Zinc finger FYVE domain-containing protein 26 (Spastizin)
				13	hpfgkeeaasQKQLLGFFceclrrgewe	Bt_ENSBTAP00000040846	
				13	ypfgkeetttekqlfeffceclrrgdwe	Rn_ENSRNOP00000016380	
				13	ypfgkeetateeelfeffceclrrgdwe	Mm_ENSMUSP00000021547	
				-	....	Gg	
				13	hpfgeeeeaslrslhgffcwclqrgnwe	Xt_ENSXETP00000016655	
				29	hpfgreeetsrrelfgffrrclqrgewe	Dr_ENSDARP00000070446	
				-	....	Ce	
				5	------medqqeenmqqllnllpkdqrq	Dm_FBpp0081863	
				-	....	Sc	
168	1	CI	ENSP00000359866 ENSG00000112175	298	lvgrqgpqskQPFMVAFFkasevllrsv	Hs_ENSP00000359866	NP_066551 Bone morphogenetic protein 5 Precursor (BMP-5)
				75	lvgrdgpydkQPFMVAFFkasevhvrsa	Bt_ENSBTAP00000025614	
				298	lvgrhgpqskQPFMVAFFkasevllrsv	Rn_ENSRNOP00000014846	
				298	lvgrhgpqskQPFMVAFFkasevllrsv	Mm_ENSMUSP00000012281	
				296	ligrhgpqskQPFMVAFFkasevlfrsv	Gg_ENSGALP00000026226	
				262	ligrhgpqskQPFLVAFFkasevllrsv	Xt_ENSXETP00000015399	
				290	iigrngpqskQPFLVAFFkasevllrsv	Dr_ENSDARP00000089269	
				217	yaraqs-------------aplivfsdl	Ce_CE26386	
				-	....	Dm	
				-	....	Sc	
169	1	CI	ENSP00000262651 ENSG00000101336	508	peerptfeyiQSVLDDFYtatesqyqqq	Hs_ENSP00000262651	NP_002101 Tyrosine-protein kinase HCK (EC 2.7.10.2)(Hemopoietic cell kinase)(p59-HCK/p60-HCK)
				558	peerptfeyiQSVLDDFYtatesqyqqq	Bt_ENSBTAP00000010429	
				506	peerptfeyiQSVLDDFYtatesqyqqq	Rn_ENSRNOP00000012432	
				506	peerptfeyiQSVLDDFYtatesqyqqq	Mm_ENSMUSP00000003370	
				476	pedrptfeymQSILEDFFtategqyqkq	Gg_ENSGALP00000010524	
				-	....	Xt	
				436	pedrptfeylQSVLEDFYtatesqyqqq	Dr_ENSDARP00000075977	

				-<br>-<br>-	............................<br>............................<br>............................	Ce<br>Dm<br>Sc	
170	1	CI	ENSP00000345774<br>ENSG00000072210	239<br>239<br>239<br>239<br>242<br>239<br>242<br>241<br>-<br>271	itwgkymncgQTCIAPDYilceaslqnq<br>iawgkfmncgQTCIAPDYvlcepslqdl<br>itwgkymncgQTCIAPDYilceaslqdq<br>iawgkymncgQTCIAPDYilceaslqnq<br>iawgkymncgQTCIAPDYvlchpsiqsq<br>vtwgkfvncgQTCIAPDYilcdksiqdk<br>iawgkysncgQTCIAPDYilcdpsiqdr<br>iawgkwlncgQTCLAPDYilvnstvkpk<br>............................<br>iffgafgnsgqicvspdyllvhksiypk	Hs_ENSP00000345774<br>Bt_ENSBTAP00000050170<br>Rn_ENSRNOP00000045886<br>Mm_ENSMUSP00000067767<br>Gg_ENSGALP00000007586<br>Xt_ENSXETP00000029530<br>Dr_ENSDARP00000049452<br>Ce_CE33307<br>Dm<br>Sc_YMR110C	NP_001026976 Fatty aldehyde dehydrogenase (EC 1.2.1.3) (Aldehyde dehydrogenase family 3 member A2)(Aldehyde dehydrogenase 10)(Microsomal aldehyde dehydrogenase)
171	1	CI	ENSP00000372336<br>ENSG00000121741	793<br>793<br>792<br>792<br>570<br>708<br>635<br>-<br>-<br>-	wrgemkhfcdQHCLLRFYcqqnepn-mt<br>wrgemkhfcdQHCLLRFYcqqsepn-mt<br>ss--vfhsctlylvlfllmivyspssfl<br>wrgemkhfcdQHCLLRFYcqqnepn-mt<br>wrgemkhfcdQHCLLRFYcqqnepn-la<br>wrgavknfcdQQCLLRFYnqqnepn-ma<br>wraelkqfcdQQCLLRFYcqqnepn-ma<br>............................<br>............................<br>............................	Hs_ENSP00000372336<br>Bt_ENSBTAP00000027190<br>Rn_ENSRNOP00000011684<br>Mm_ENSMUSP00000022511<br>Gg_ENSGALP00000027631<br>Xt_ENSXETP00000005672<br>Dr_ENSDARP00000005018<br>Ce<br>Dm<br>Sc	NP_003444 Zinc finger MYM-type protein 2 (Zinc finger protein 198)(Fused in myeloproliferative disorders protein)(Rearranged in atypical myeloproliferative disorder protein)
172	1	C	ENSP00000335371<br>ENSG00000168538	385<br>385<br>385<br>385<br>385<br>385<br>385<br>402<br>405<br>-	mypnpdpletqtgvldfygqrswrqgil<br>typnpdpletqagvldfygqrswrqgvl<br>vypspdpletptgvldfygqrpwrqgil<br>mypnpdpletqsgvldfygqrpwrqgil<br>vypnpdpletqtgvldfygqrpwrqgtl<br>aypspdpletvngvldfygqrawrqgil<br>gypapdplettsgaldfygqrpwrqghq<br>pypspdplanss-stvffgqrpwrinhe<br>seatptpiqnplslyteffg------ir<br>............................	Hs_ENSP00000335371<br>Bt_ENSBTAP00000009769<br>Rn_ENSRNOP00000018969<br>Mm_ENSMUSP00000047562<br>Gg_ENSGALP00000017312<br>Xt_ENSXETP00000041337<br>Dr_ENSDARP00000097453<br>Ce_CE03884<br>Dm_FBpp0072905<br>Sc	NP_068761 UPF0636 protein C4orf41
172	2	CI	ENSP00000335371<br>ENSG00000168538	1074<br>1074<br>1074<br>1074<br>1073<br>613<br>1075<br>1052<br>1276<br>-	irlrilpgteQEMLYNFYplmagyqqlp<br>irlrilpgteQEMLYNFYplmagyqqlp<br>irlrilpgtkQEMLYNFYplmagyqqlp<br>irlrilpgtkQEMLYNFYplmagyqqlp<br>irlrilpgtqQEVLYNFYplmagyqqlp<br>----------------------------<br>vrmrilpgaeQEMLYNFYplmagyqvlp<br>vtmtvlpgatrrvtvvvmalsagrlnfp<br>ldisimayeekelvynlyplqvgwqelp<br>............................	Hs_ENSP00000335371<br>Bt_ENSBTAP00000009769<br>Rn_ENSRNOP00000018969<br>Mm_ENSMUSP00000047562<br>Gg_ENSGALP00000017312<br>Xt_ENSXETP00000041337<br>Dr_ENSDARP00000097453<br>Ce_CE03884<br>Dm_FBpp0072905<br>Sc	NP_068761 UPF0636 protein C4orf41
173	1	CI	ENSP00000331682<br>ENSG00000185214	290<br>290<br>290<br>290<br>290<br>281<br>-<br>-<br>-<br>-	eslqpslqspQTELRSDFqcvvgfggih<br>eslqpslqgpQTELRSDFqcvvgfggih<br>eslqpslqgphtelrsdfqcvvgfggih<br>eilqpslqgpQTELRSDFqcvvgfggih<br>eclqpmlqssqtqlrsefqcvvgfggmh<br>esmqpvmqgpntqlrsefhcvvgfggmy<br>............................<br>............................<br>............................<br>............................	Hs_ENSP00000331682<br>Bt_ENSBTAP00000026853<br>Rn_ENSRNOP00000002569<br>Mm_ENSMUSP00000087607<br>Gg_ENSGALP00000010351<br>Xt_ENSXETP00000009276<br>Dr<br>Ce<br>Dm<br>Sc	NP_116164 Kelch-like protein 22
174	1	CI	ENSP00000231509<br>ENSG00000113580	58<br>59<br>59<br>59<br>60<br>60<br>-<br>-<br>-<br>-	lavasqsdskQRRLLVDFpkgsvsnaq-<br>laaasqsdskQQRLLVDFpkgsgsnaq-<br>vaaasqadskQQRILLDFskgstsnvqq<br>vaaasqadskQQRILLDFskgsasnaq-<br>lpvssqsdsaqqpaladfskglvnnvp-<br>tstasqsntrqqqqflkqkavtgdstn-<br>............................<br>............................<br>............................<br>............................	Hs_ENSP00000231509<br>Bt_ENSBTAP00000025941<br>Rn_ENSRNOP00000019409<br>Mm_ENSMUSP00000095199<br>Gg_ENSGALP00000011948<br>Xt_ENSXETP00000003968<br>Dr<br>Ce<br>Dm<br>Sc	NP_001019265 Glucocorticoid receptor (GR)(Nuclear receptor subfamily 3 group C member 1)
175	1	CI	ENSP00000225696<br>ENSG00000108559	681<br>682<br>693<br>693<br>662<br>666<br>660	qlrhlgnaikQVTMKKDYqqqkmekvl-<br>qlrhlgnaikQVTMKKDYqqrkmekvl-<br>qlrhlgnaikQVTMKKDYqqrkmekvl-<br>qlrhlgnaikQVTMKKDYqqrkmekvl-<br>qlqhlsnairqvkmkkeyqqkkmekgi-<br>qlqqlgnainqvnrkmsyqekqmekgk-<br>qlrhldngikQVNMKKDYqkkqmnaga-	Hs_ENSP00000225696<br>Bt_ENSBTAP00000001625<br>Rn_ENSRNOP00000053427<br>Mm_ENSMUSP00000048101<br>Gg_ENSGALP00000002634<br>Xt_ENSXETP00000046193<br>Dr_ENSDARP00000025718	NP_002523 Nuclear pore complex protein Nup88 (Nucleoporin Nup88)(88 kDa nuclear pore complex protein)

				-	....	Ce	
				641	vtqslaagletakktfnkqryhiaqsqe	Dm_FBpp0082137	
				-	....	Sc	
176	1	CI	ENSP00000373884 ENSG00000198838	3777	istvdyllrlQESISDFYwyysgkdiid	Hs_ENSP00000373884	NP_001027 Ryanodine receptor 3 (RYR-3)(RyR3)(Brain-type ryanodine receptor)(Brain ryanodine receptor-calcium release channel)
				3226	istvdyllrlQESISDFYwyysgkdiid	Bt_ENSBTAP00000036054	
				-	....	Rn	
				3796	istvdyllrlQESISDFYwyysgkdiid	Mm_ENSMUSP00000089426	
				3783	istvdyllrlQESISDFYwyysgkdvid	Gg_ENSGALP00000005342	
				-	....	Xt	
				3792	istvdyllrlQESISDFYwyysgkdvmd	Dr_ENSDARP00000046552	
				-	....	Ce	
				-	....	Dm	
				-	....	Sc	
177	1	CI	ENSP00000300128 ENSG00000166881	109	eneeklkeleQFSIWNFFs---sflkek	Hs_ENSP00000300128	NP_001124435 Transmembrane protein 194A
				82	enedklkeleQFSIWNFFs---sflkek	Bt_ENSBTAP00000019519	
				-	....	Rn	
				107	dneeklkeleQFSIWNFFs---sflkek	Mm_ENSMUSP00000045988	
				66	gseeelreleeskvwnfls---sllkek	Gg_ENSGALP00000000663	
				99	eneeklkemetfnmfdffs---sflkek	Xt_ENSXETP00000009646	
				-	....	Dr	
				-	....	Ce	
				87	ddysqyggktpeevlqhykdkqslfsit	Dm_FBpp0074150	
				-	....	Sc	
178	1	C	ENSP00000321656 ENSG00000158402	279	itqmleedsnQGHLIGDFskvcalptvs	Hs_ENSP00000321656	NP_001781 M-phase inducer phosphatase 3 (EC 3.1.3.48) (Dual specificity phosphatase Cdc25C)
				283	mtqmleedsnQGPLIGDFskvcalptvs	Bt_ENSBTAP00000006968	
				272	vyqlftrftphhvivnnns-acvlptvp	Rn_ENSRNOP00000042699	
				252	aiqmeeeesgselligdfskvcvlptvp	Mm_ENSMUSP00000055427	
				-	....	Gg	
				359	istvldedcghrqligdfskvyalptvt	Xt_ENSXETP00000014678	
				-	....	Dr	
				-	....	Ce	
				219	imralgdep---eligdlskpctlpcla	Dm_FBpp0080412	
				216	ssplapnsvgvkcfesclaktqipyyyd	Sc_YMR036C	
178	2	CI	ENSP00000321656 ENSG00000158402	350	iqg-alnlysQEELFNFFlkkpivpldt	Hs_ENSP00000321656	NP_001781 M-phase inducer phosphatase 3 (EC 3.1.3.48) (Dual specificity phosphatase Cdc25C)
				354	iqg-alnlhsQEELYNFFlkkpivpwdn	Bt_ENSBTAP00000006968	
				343	ilqgalnlysQKELYEFFlkkpvvpedt	Rn_ENSRNOP00000042699	
				323	ilg-alnlhsQKELHEFFlrkpvvpldi	Mm_ENSMUSP00000055427	
				-	....	Gg	
				430	ikg-alnlhrqeevtdyflkqplapsva	Xt_ENSXETP00000014678	
				-	....	Dr	
				-	....	Ce	
				294	irg-aknlytrgqiqeafp---tltsnq	Dm_FBpp0080412	
				290	iin-svnihsrdeleyefihkvlhsdts	Sc_YMR036C	
179	1	CI	ENSP00000336842 ENSG00000113812	387	yqfrlgdeklQAPMALFYpatfgivgqk	Hs_ENSP00000336842	NP_075050 Actin-related protein 8 (INO80 complex subunit N)
				387	yqfrlgdeklQAPMALFYpatfgivgqk	Bt_ENSBTAP00000014847	
				387	yqfrlgdeklQAPMALFYpatfgivgqk	Rn_ENSRNOP00000020750	
				387	yqfrlgdeklQAPMALFYpatfgivgqk	Mm_ENSMUSP00000016115	
				384	yqfrlgdeklQAPMALFYpatfgivgqk	Gg_ENSGALP00000008637	
				-	....	Xt	
				-	....	Dr	
				-	....	Ce	
				383	ytiqvgdealmaplalfhtellnitgrt	Dm_FBpp0074318	
				606	yefklfdevmlaplalffpqifklirts	Sc_YOR141C	
180	1	CI	ENSP00000357597 ENSG00000189241	371	hepqsfirrnQDLICSFFtwfsdhslpe	Hs_ENSP00000357597	NP_003300 Testis-specific Y-encoded-like protein 1 (TSPY-like protein 1)
				366	hepqsfirrnqevvcnfftwfsdhslpe	Bt_ENSBTAP00000052314	
				313	hepqsfirrnrdlicsfftwfsdhsvpe	Rn_ENSRNOP00000000660	
				313	hepqsfirrnQDLICSFFtwfsdhslpe	Mm_ENSMUSP00000063051	
				-	....	Gg	
				-	....	Xt	
				-	....	Dr	
				-	....	Ce	
				-	....	Dm	
				-	....	Sc	
181	1	CI	ENSP00000295924 ENSG00000163659	286	qkwqsvfndsQEHLERFYcnpendrmrm	Hs_ENSP00000295924	NP_056323 TCDD-inducible poly
				285	qkwqsvsndsQEHLERFYcnpendrmrm	Bt_ENSBTAP00000016071	
				287	qkwqsvsndsQEHLERFYcnpendrmrm	Rn_ENSRNOP00000015387	
				287	qkwqsvsndsQEHLERFYcnpendrmrm	Mm_ENSMUSP00000048051	
				296	qswqsvsndsqehlerlycnpdndrikv	Gg_ENSGALP00000016663	
				295	siwqsvnddsqehverlycspdsdrikm	Xt_ENSXETP00000026790	
				259	qiwqsisddsqeqlerlycnpdnehvrl	Dr_ENSDARP00000083106	

				-	....	Ce	
				-	....	Dm	
				-	....	Sc	
182	1	CI	ENSP00000353826 ENSG00000196365	515	iavsqlrgstQGKILCFYgppgvgktsi	Hs_ENSP00000353826	NP_004784 Lon protease homolog, mitochondrial Precursor (EC 3.4.21.-)(Lon protease-like protein)(LONP)(LONHs)(Mitochondrial ATP-dependent protease Lon)(Serine protease 15)
				516	iavsqlrgstQGKILCFYgppgvgktsi	Bt_ENSBTAP00000002350	
				-	....	Rn	
				507	iavsqlrgstqgkilcfhgppgvgktsi	Mm_ENSMUSP00000094904	
				411	iavsqlrgstQGKILCFYgppgvgktsi	Gg_ENSGALP00000000670	
				447	iavsqlrgstQGKILCFYgppgvgktsi	Xt_ENSXETP00000047371	
				458	iavsqlrgstQGKILCFYgppgvgktsi	Dr_ENSDARP00000096921	
				504	iavnllrksiggkilcfhgppgvgktsi	Ce_CE16894	
				556	iavsslkgstqgkilcfhgppgvgktsi	Dm_FBpp0271918	
				624	iavgkllgkvdgkiicfvgppgvgktsi	Sc_YBL022C	
183	1	CI	ENSP00000370844 ENSG00000132975	230	ickivmrhahQIALQHHFlats-hyvtt	Hs_ENSP00000370844	NP_005279 Probable G-protein coupled receptor 12
				225	ickivmrhahQIALQHHFlats-hyvtt	Bt_ENSBTAP00000014267	
				230	ickivmrhahQIALQHHFlats-hyvtt	Rn_ENSRNOP00000057842	
				230	ickivmrhahQIALQHHFlats-hyvtt	Mm_ENSMUSP00000038245	
				195	ickivmrhahQIALQHHFlats-hyvtt	Gg_ENSGALP00000027573	
				219	icriafrhaqqiavqhqfmats-qasst	Xt_ENSXETP00000054474	
				236	ickivmrhahQIALQHHFlaasphyvtt	Dr_ENSDARP00000085961	
				-	....	Ce	
				-	....	Dm	
				-	....	Sc	
184	1	CI	ENSP00000364133 ENSG00000106799	275	adnkdngtwtQLWLVSDYhehgslfdyl	Hs_ENSP00000364133	NP_004603 TGF-beta receptor type-1 Precursor (EC 2.7.11.30)(Transforming growth factor-beta receptor type I)(TGF-beta receptor type I)(TGF-beta type I receptor)(TbetaR-I)(TGFR-1)(Serine/threonine-protein kinase receptor R4)(SKR4)(Activin receptor-like kinase 5)(ALK-5)
				271	adnkdngtwtQLWLVSDYhehgslfdyl	Bt_ENSBTAP00000024010	
				269	adnkdngtwtQLWLVSDYhehgslfdyl	Rn_ENSRNOP00000009452	
				275	adnkdngtwtQLWLVSDYhehgslfdyl	Mm_ENSMUSP00000007757	
				275	adnkdngtwtQLWLVSDYhehgslfdyl	Gg_ENSGALP00000020569	
				271	adnkdngtwtQLWLVSDYhehgslfdyl	Xt_ENSXETP00000050561	
				254	adnkdngtwtQLWLVSDYhehgslfdyl	Dr_ENSDARP00000061972	
				-	....	Ce	
				394	adnkdngtwtQLWLVTDYhengslfdyl	Dm_FBpp0087739	
				-	....	Sc	
185	1	CI	ENSP00000347978 ENSG00000134899	990	qldaqq---tQLRIDSFFrlaqqekeda	Hs_ENSP00000347978	NP_000114 DNA-repair protein complementing XP-G cells (Xeroderma pigmentosum group G-complementing protein)(DNA excision repair protein ERCC-5)
				1006	qlnvhq---tQLRIDSFFrlaqqekqea	Bt_ENSBTAP00000043672	
				519	qlnahq---tQLRIDSFFrlaqqekqda	Rn_ENSRNOP00000056106	
				989	hlnahq---tQLRIDSFFrlaqqekqda	Mm_ENSMUSP00000027214	
				938	qlnlqq---tQLRIDSFFrlaqhekq--	Gg_ENSGALP00000027186	
				1031	qinaqq---tQLRIDSFFrleqheaig-	Xt_ENSXETP00000005980	
				-	....	Dr	
				719	afekwn---------nflksggqsqmrl	Ce_CE11308	
				1113	kinekk---iqgsirnyftaks-alrvq	Dm_FBpp0099904	
				995	dvnkrkkkgkQKRINEFFpreyisgdk-	Sc_YGR258C	
186	1	CI	ENSP00000360815 ENSG00000085831	368	elmwcftykgQWKMSYFYadllskencw	Hs_ENSP00000360815	Tetratricopeptide repeat protein 39A (TPR repeat protein 39A)(Differentially expressed in MCF7 with estradiol protein 6)(DEME-6)
				327	elmwcftykaQWKMAYFYadllskensw	Bt_ENSBTAP00000003183	
				331	elmwcftykgQWKMAYFYadllskensw	Rn_ENSRNOP00000013459	
				333	elmwcftykgQWKMAYFYadllskensw	Mm_ENSMUSP00000102229	
				310	elmwcftykrQWKMAFFYadllskentw	Gg_ENSGALP00000017136	
				326	qlmwcftykqhwkmayfyadllskensw	Xt_ENSXETP00000006193	
				234	elmwcftfkrywkmayfyadllsqesrw	Dr_ENSDARP00000085503	
				-	....	Ce	
				-	....	Dm	
				465	draitlihlhqydraaedilsllldisdw	Sc_YKR018C	
187	1	CI	ENSP00000385215 ENSG00000135315	1256	vaelnrkiatQEVLIRHFqsqvnelqsk	Hs_ENSP00000385215	NP_055710 Protein QN1 homolog
				1198	vaelnrkiatQEVLIKHFqnqvnelqsk	Bt_ENSBTAP00000004386	
				1173	vaelnrkiatQEVLLKHFqgqvnelqgk	Rn_ENSRNOP00000014165	
				1256	vaelnrkiatQEVLLKHFqgqvnelqgk	Mm_ENSMUSP00000091319	
				1182	vaelngristqeilikhlqeqisehqrh	Gg_ENSGALP00000025490	
				-	....	Xt	
				-	....	Dr	
				-	....	Ce	
				-	....	Dm	
				-	....	Sc	
188	1	CI	ENSP00000265990 ENSG00000095564	1528	qdyyctlsplQVQLYEDFaksrakcdvd	Hs_ENSP00000265990	NP_003963 TATA-binding protein-associated factor 172 (EC 3.6.1.-)(ATP-dependent helicase BTAF1)(TBP-associated factor 172)(TAF-172)
				1483	qdyyctlsplQVQLYEDFaksrakcdvd	Bt_ENSBTAP00000021438	
				1523	qdyyctlsplQVQLYEDFaksrakcdvd	Rn_ENSRNOP00000024465	
				1527	qdyyctlsplQVQLYEDFaksrakcdvd	Mm_ENSMUSP00000097093	
				1522	qdyycvlsplQVQLYEDFaksrakcdid	Gg_ENSGALP00000011221	
				-	....	Xt	

				1532<br>1343<br>1613<br>1532	qdyycnlsplQVQLYEDFaksrakvnvd<br>qdyeceltedqkeiyrfvvdrctssqed<br>qdllcelsplQLRLYEDFsnkhlkdcld<br>qdyycelgdlQKQLYMDFtkk-qknvve	Dr_ENSDARP00000078762<br>Ce_CE42370<br>Dm_FBpp0082668<br>Sc_YPL082C	(TAF(II)170)(B-TFIID transcription factor-associated 170 kDa subunit)
189	1	CI	ENSP00000354923<br>ENSG00000198947	829<br>-<br>819<br>831<br>831<br>-<br>-<br>801<br>782<br>-	lserlnwleyQNNIIAFYnqlqqleqmt<br>............................<br>lservnwleyQNNIITFYnqlqqleqmt<br>lservnwleyQTNIITFYnqlqqleqmt<br>lserlvwleyQNSIIDFYsqlqrleqta<br>............................<br>............................<br>iseamnvltgqeaggngngsee--aava<br>lssqqseshtllakekyynsltgfklvl<br>............................	Hs_ENSP00000354923<br>Bt<br>Rn_ENSRNOP00000029969<br>Mm_ENSMUSP00000109633<br>Gg_ENSGALP00000026200<br>Xt<br>Dr<br>Ce_CE27129<br>Dm_FBpp0110219<br>Sc	NP_003998 Dystrophin
190	1	CI	ENSP00000379773<br>ENSG00000122299	843<br>844<br>843<br>842<br>849<br>856<br>855<br>-<br>-<br>-	asqsnkengkQIHMPTDYaevtvdfhcw<br>asqsskengkQIHMPTDYaevtvdfhcw<br>sshcskengkQIHMPTDYaevtvdfhcw<br>ashcskengkQIHMPTDYaevtvdfhcw<br>aaqankengkQIHMPTDYaevtvdfhcw<br>laqvn----kQIHLPTDYaettvdfhcw<br>sniatrengkQIHMPTDYaeevagnhcw<br>............................<br>............................<br>............................	Hs_ENSP00000379773<br>Bt_ENSBTAP00000019746<br>Rn_ENSRNOP00000003309<br>Mm_ENSMUSP00000041308<br>Gg_ENSGALP00000004959<br>Xt_ENSXETP00000036248<br>Dr_ENSDARP00000003298<br>Ce<br>Dm<br>Sc	NP_054872 Zinc finger CCCH domain-containing protein 7A
191	1	CI	ENSP00000361170<br>ENSG00000132781	523<br>503<br>500<br>494<br>461<br>436<br>-<br>-<br>-<br>-	pcsrkkprmgQQVLDNFFrshistdahs<br>lskrkkpspgQQVLESFFwphvptdaps<br>pssrkkpsrgQQVLDRFFqrhipthkp-<br>pssrkkpslgQQVLDTFFqrhiptdkp-<br>----------------------------<br>----------------------------<br>............................<br>............................<br>............................<br>............................	Hs_ENSP00000361170<br>Bt_ENSBTAP00000014928<br>Rn_ENSRNOP00000024375<br>Mm_ENSMUSP00000099760<br>Gg_ENSGALP00000016605<br>Xt_ENSXETP00000007625<br>Dr<br>Ce<br>Dm<br>Sc	NP_001121897 A/G-specific adenine DNA glycosylase (EC 3.2.2.-)(MutY homolog) (hMYH)
192	1	CI	ENSP00000323036<br>ENSG00000014257	72<br>74<br>71<br>71<br>75<br>-<br>-<br>-<br>103<br>-	swpqgfgqltQLGMEQHYelgeyirkry<br>swpqgfgqltQLGMAQHYelgqyirkry<br>swpqgfgqltkwgmgqhyelgsyirrry<br>swpqgfgqltQWGMEQHYelgsyirkry<br>ewpqgfgqltktgiqqlfelgqytrkry<br>............................<br>............................<br>............................<br>fwptgwgdltnlgkqehydlgkwlrnry<br>............................	Hs_ENSP00000323036<br>Bt_ENSBTAP00000015451<br>Rn_ENSRNOP00000016222<br>Mm_ENSMUSP00000059889<br>Gg_ENSGALP00000019020<br>Xt<br>Dr<br>Ce<br>Dm_FBpp0084975<br>Sc	NP_001127666 Prostatic acid phosphatase Precursor (EC 3.1.3.2)
193	1	CI	ENSP00000217086<br>ENSG00000101115	578<br>584<br>-<br>585<br>471<br>553<br>575<br>-<br>-<br>-	clichrvlscQSSLKMHYrthtgerpfq<br>clichrvlscQSSLKMHYrthtgerpfp<br>............................<br>clichrvlscQSSLKMHYrthtgerpfq<br>clichrvlscQSSLKMHYrthtgerpfk<br>clichrvlscpsslkmhyrthtgerpfk<br>cvichrvlscQSSLKMHYrthtgerpyk<br>............................<br>............................<br>............................	Hs_ENSP00000217086<br>Bt_ENSBTAP00000004035<br>Rn<br>Mm_ENSMUSP00000029061<br>Gg_ENSGALP00000012696<br>Xt_ENSXETP00000026959<br>Dr_ENSDARP00000065330<br>Ce<br>Dm<br>Sc	NP_065169 Sal-like protein 4 (Zinc finger protein SALL4)
194	1	CI	ENSP00000350009<br>ENSG00000077254	570<br>540<br>537<br>537<br>538<br>520<br>525<br>405<br>-<br>-	swfwgpvvtlQDCLAAFFardelkgdnm<br>swfwgpvvtlQDCLAAFFardelkgdnm<br>swfwgpvvtlQDCLAAFFardelkgdnm<br>swfwgpvvtlQDCLAAFFardelkgdnm<br>swfwgpvvtlQDCLAAFFardelkgdnm<br>swfwgptvtlQDCLAAFFardelkgdnm<br>swfwgpvvtlQDCLAAFFardelkgdnm<br>------ktslescldrffqnstlqddnq<br>............................<br>............................	Hs_ENSP00000350009<br>Bt_ENSBTAP00000027667<br>Rn_ENSRNOP00000016022<br>Mm_ENSMUSP00000026507<br>Gg_ENSGALP00000014582<br>Xt_ENSXETP00000028749<br>Dr_ENSDARP00000014516<br>Ce_CE31559<br>Dm<br>Sc	NP_055832 Ubiquitin carboxyl-terminal hydrolase 33 (EC 3.1.2.15)(Ubiquitin thioesterase 33)(Ubiquitin-specific-processing protease 33) (Deubiquitinating enzyme 33) (VHL-interacting deubiquitinating enzyme 1)
195	1	CI	ENSP00000261405<br>ENSG00000110799	890<br>608<br>887<br>893<br>887<br>889	glkylfpgecQYVLVQDYcgsnpgtfri<br>glkylfpgecqyvlvqdhcgsnpgtfrv<br>glkylfpgecQYVLVQDYcggssgtfri<br>glkylfpgecQYVLVQDYcgsnpgtfqi<br>gmkykfpgecQYVLVQDFcknnsgtfri<br>gfrykfpgdcQYVLAQDYcggvtgtfri	Hs_ENSP00000261405<br>Bt_ENSBTAP00000016273<br>Rn_ENSRNOP00000026643<br>Mm_ENSMUSP00000107873<br>Gg_ENSGALP00000036463<br>Xt_ENSXETP00000050938	NP_000543 von Willebrand factor Precursor (vWF)

				-<br>-<br>-<br>-	............................<br>............................<br>............................<br>............................	Dr<br>Ce<br>Dm<br>Sc	
196	1	C	ENSP00000348668<br>ENSG00000139629	87<br>96<br>87<br>87<br>-<br>0<br>82<br>-<br>-<br>-	eaqqtlfsinQSCLPGFYtpaelkpfwe<br>epqqtlastnQSCLPGFYtpaelkpfwe<br>epqqnpvstnhsclpgfytpaelrpfwe<br>eppqtlvstnhsclpgfytpaelkpfwd<br>............................<br>---------------------elkpfme<br>evqp---tpdrncpsgfytqrelkpwie<br>............................<br>............................<br>............................	Hs_ENSP00000348668<br>Bt_ENSBTAP00000023861<br>Rn_ENSRNOP00000042281<br>Mm_ENSMUSP00000056705<br>Gg<br>Xt_ENSXETP00000030934<br>Dr_ENSDARP00000007473<br>Ce<br>Dm<br>Sc	NP_009141 Polypeptide N-acetylgalactosaminyltransferase 6 (EC 2.4.1.41)(Polypeptide GalNAc transferase 6)(pp-GaNTase 6)(GalNAc-T6)(Protein-UDP acetylgalactosaminyltransferase 6)(UDP-GalNAc:polypeptide N-acetylgalactosaminyltransferase 6)
197	1	CI	ENSP00000303830<br>ENSG00000171105	450<br>441<br>417<br>450<br>414<br>-<br>408<br>-<br>-<br>-	dwskhnltitQGKLFFHYnpklclseih<br>dwskhnltitQGKLFFHYnpklclseih<br>dwnkhnltitQGKLFFHYnpklclseih<br>dwskhnltitQGKLFFHYnpklclseih<br>dwskhnltiargklffhynpklclseih<br>............................<br>dwskhnltiqqgrmffhhnsklcmseir<br>............................<br>............................<br>............................	Hs_ENSP00000303830<br>Bt_ENSBTAP00000016858<br>Rn_ENSRNOP00000046668<br>Mm_ENSMUSP00000088837<br>Gg_ENSGALP00000005655<br>Xt<br>Dr_ENSDARP00000023951<br>Ce<br>Dm<br>Sc	NP_000199 Insulin receptor Precursor (IR)(EC 2.7.10.1)(CD220 antigen)
198	1	CI	ENSP00000355205<br>ENSG00000128908	391<br>400<br>393<br>393<br>396<br>-<br>392<br>-<br>424<br>587	qqrklnflitQTELYAHFmsrkrdmghd<br>qqrklnflitQTELYAHFmsrkrdmghd<br>qqrklnflitQTELYAHFmsrkrdmghd<br>qqrklnflitQTELYAHFmsrkrdmghd<br>qqrklnflitQTELYAHFmsrkrdighd<br>............................<br>qqrklnflitQTELYAHFmggkqnaggd<br>............................<br>qqrklnflitQTELYAHFmskklgqgse<br>qakklnflltQTELYSHFigrkiktnel	Hs_ENSP00000355205<br>Bt_ENSBTAP00000013708<br>Rn_ENSRNOP00000035575<br>Mm_ENSMUSP00000051845<br>Gg_ENSGALP00000013917<br>Xt<br>Dr_ENSDARP00000094113<br>Ce<br>Dm_FBpp0083185<br>Sc_YGL150C	NP_060023 Putative DNA helicase INO80 complex homolog 1 (hINO80)(EC 3.6.1.-)
199	1	CI	ENSP00000303469<br>ENSG00000171944	253<br>253<br>253<br>253<br>-<br>-<br>-<br>-<br>-<br>-	tciahicvflQFYLLAFFsffthrfgsh<br>tcithicvflQFYLLGFFsffthrfgsh<br>tciahicvflQFYLLAFFsffthrfgah<br>tciahicvflQFYLLAFFsffthrfgah<br>............................<br>............................<br>............................<br>............................<br>............................<br>............................	Hs_ENSP00000303469<br>Bt_ENSBTAP00000049496<br>Rn_ENSRNOP00000039523<br>Mm_ENSMUSP00000102490<br>Gg<br>Xt<br>Dr<br>Ce<br>Dm<br>Sc	NP_001005160 Olfactory receptor 52A5 (Olfactory receptor OR11-33)(Odorant receptor HOR3'beta5)
200	1	CI	ENSP00000349396<br>ENSG00000119688	215<br>215<br>215<br>215<br>121<br>203<br>-<br>207<br>-<br>-	mgpivmklvhQEKLEGDFrfkhmqirvn<br>mgpivaklvqQEKLEGDFrfkhmqirvn<br>mgpivtklvqQEKLEGDFrfkhmqirvn<br>mgpivtklvqQEKLEGDFrfkhmqirvn<br>mspivsklvqQEKLEGDFrfkhmqirvn<br>mgpvipklvqQEKLEGDFrykhvqvrvn<br>............................<br>lspivqkvseQEKMEGDFrqrhmevrsn<br>............................<br>............................	Hs_ENSP00000349396<br>Bt_ENSBTAP00000019482<br>Rn_ENSRNOP00000016040<br>Mm_ENSMUSP00000021666<br>Gg_ENSGALP00000016610<br>Xt_ENSXETP00000029109<br>Dr<br>Ce_CE05518<br>Dm<br>Sc	NP_005041 ATP-binding cassette sub-family D member 4 (Peroxisomal membrane protein 69)(PMP69)(Peroxisomal membrane protein 1-like)(PXMP1-L)(P70R)
201	1	CI	ENSP00000387040<br>ENSG00000165323	584<br>0<br>583<br>579<br>585<br>0<br>543<br>-<br>-<br>-	nsplfekvacQGVISYDFpvgghitavs<br>----------------------------<br>nsplfekvacQGVISYDFpvgghitais<br>nsplfekvacQGVISYDFpvgghitais<br>nkplfekvacQGVISSDFpvgghitavs<br>----------------------------<br>nkplfekvacvgvvsrelpvgeviatis<br>............................<br>............................<br>............................	Hs_ENSP00000387040<br>Bt_ENSBTAP00000005332<br>Rn_ENSRNOP00000015976<br>Mm_ENSMUSP00000080808<br>Gg_ENSGALP00000027793<br>Xt_ENSXETP00000037341<br>Dr_ENSDARP00000042081<br>Ce<br>Dm<br>Sc	NP_001008781 Protocadherin Fat 3 Precursor (hFat3)(FAT tumor suppressor homolog 3)
202	1	CI	ENSP00000273261<br>ENSG00000144749	576<br>456<br>470	qvtfghegryQCVITNHFgstyshkarl<br>rlsfehegryQCVITNHFgssyshkakl<br>hvtfghegryQCVITNHFgstyshkarl	Hs_ENSP00000273261<br>Bt_ENSBTAP00000030722<br>Rn_ENSRNOP00000017384	NP_056356 Leucine-rich repeats and immunoglobulin-

				578<br>560<br>541<br>-<br>-<br>-<br>-	hvtfghegryQCIITNHFgstyshkarl<br>hvtfahegryQCIITNHFgstysnkarl<br>nltfahegryQCIITNDFgpsysskarl<br>............................<br>............................<br>............................<br>............................	Mm_ENSMUSP00000032105<br>Gg_ENSGALP00000035449<br>Xt_ENSXETP00000005355<br>Dr<br>Ce<br>Dm<br>Sc	like domains protein 1 Precursor (LIG-1)
203	1	CI	ENSP00000345629<br>ENSG00000180815	319<br>209<br>151<br>323<br>200<br>279<br>263<br>314<br>247<br>-	mlp-tcdladQHNIKFHYafalnrrnst<br>mlp-tcdladQHNIKFHYafalnrrnsa<br>mlp-tcdladQHNIKFHYafalnrrnst<br>mlp-tcdladQHNIKFHYafalnrrnst<br>mlp-tcdladQHNIKFHYafalnrrnna<br>mlp-tcdladQHNIKFHYafalnrrnnt<br>mlp-tcdlanQPMIQFHYafalnrrnsp<br>ripdclkvvdtpviryqyafalnrrnkd<br>nipntrkyvetgnmsflyafalnrrnrk<br>............................	Hs_ENSP00000345629<br>Bt_ENSBTAP00000026456<br>Rn_ENSRNOP00000050378<br>Mm_ENSMUSP00000033665<br>Gg_ENSGALP00000026448<br>Xt_ENSXETP00000013645<br>Dr_ENSDARP00000012804<br>Ce_CE33405<br>Dm_FBpp0289484<br>Sc	Mitogen-activated protein kinase kinase kinase 15 (EC 2.7.11.25)(MAPK/ERK kinase kinase 15)(MEK kinase 15) (MEKK 15)
204	1	CI	ENSP00000294383<br>ENSG00000162402	2532<br>1782<br>-<br>2529<br>2524<br>1293<br>2203<br>2850<br>-<br>-	hhwswavqwlQKKMSEHYwtpqsnvsne<br>hhwswavqwlQKKMSEHYwtpqsnvsne<br>............................<br>hhwswavqwlQKKMSEHYwtpqsnvsne<br>hhwswavqwlQKKMSEHYwapqsnvsne<br>----------------------------<br>----------------------------<br>skrfrsifkmsyaendidsdepmdesde<br>............................<br>............................	Hs_ENSP00000294383<br>Bt_ENSBTAP00000013190<br>Rn<br>Mm_ENSMUSP00000092538<br>Gg_ENSGALP00000017547<br>Xt_ENSXETP00000021067<br>Dr_ENSDARP00000078325<br>Ce_CE43363<br>Dm<br>Sc	Ubiquitin carboxyl-terminal hydrolase 24 (EC 3.1.2.15) (Ubiquitin thioesterase 24) (Ubiquitin-specific-processing protease 24)(Deubiquitinating enzyme 24)
205	1	CI	ENSP00000263640<br>ENSG00000115170	278<br>278<br>278<br>278<br>97<br>278<br>275<br>-<br>334<br>-	sdmtsrhsstQLWLITHYhemgslydyl<br>sdmtsrhsstQLWLITHYhemgslydyl<br>sdmtsrhsstQLWLITHYhemgslydyl<br>sdmtsrhsstQLWLITHYhemgslydyl<br>sdmtsrnsstQLWLITHYhemgslydyl<br>sdmtsrnsstQLWLITHYhelgslydyl<br>sdmtsrnsstQLWLITHYhengslydyl<br>............................<br>sdmtsrnsctQLWLMTHYyplgslfdhl<br>............................	Hs_ENSP00000263640<br>Bt_ENSBTAP00000015797<br>Rn_ENSRNOP00000006963<br>Mm_ENSMUSP00000108218<br>Gg_ENSGALP00000020469<br>Xt_ENSXETP00000022799<br>Dr_ENSDARP00000003221<br>Ce<br>Dm_FBpp0087908<br>Sc	NP_001096 Activin receptor type-1 Precursor (EC 2.7.11.30) (Activin receptor type I)(ACTR-I)(Serine/threonine-protein kinase receptor R1)(SKR1) (Activin receptor-like kinase 2) (ALK-2)(TGF-B superfamily receptor type I)(TSR-I)
206	1	CI	ENSP00000263681<br>ENSG00000077514	456<br>468<br>452<br>452<br>450<br>-<br>387<br>-<br>-<br>-	gtaalgkanrQVSITGFFqrk-------<br>gtaamgkanrQVAITGFFqrk-------<br>gaaalgkanrQVSITGFFqkk-------<br>gaaalgkanrQVSITGFFqkk-------<br>gaatasrankqisimgfcqkk-------<br>............................<br>ktsaaskptkQPSIMGFFqkk-------<br>............................<br>............................<br>............................	Hs_ENSP00000263681<br>Bt_ENSBTAP00000022442<br>Rn_ENSRNOP00000024875<br>Mm_ENSMUSP00000102694<br>Gg_ENSGALP00000027913<br>Xt<br>Dr_ENSDARP00000033687<br>Ce<br>Dm<br>Sc	NP_006582 DNA polymerase delta subunit 3 (DNA polymerase delta subunit p66)
207	1	CI	ENSP00000384494<br>ENSG00000204463	812<br>-<br>-<br>-<br>-<br>770<br>833<br>-<br>952<br>-	hghfqplqrlQPQLRSFFhqhylggqep<br>............................<br>............................<br>............................<br>............................<br>hghsqplqnlQPQLRSFFlqeylhqadp<br>hgnpqplsriQPQLTAFFtehylqgrep<br>............................<br>gdtaaspsgeaapiasttpaaplqtvlp<br>............................	Hs_ENSP00000384494<br>Bt<br>Rn<br>Mm<br>Gg<br>Xt_ENSXETP00000005320<br>Dr_ENSDARP00000052114<br>Ce<br>Dm_FBpp0288465<br>Sc	Large proline-rich protein BAT3 (HLA-B-associated transcript 3) (Protein G3)
208	1	CI	ENSP00000384418<br>ENSG00000131473	510<br>510<br>510<br>510<br>510<br>510<br>501<br>510<br>522<br>-	ivwgmqtravQGMLDFDYvcsrdepsva<br>ivwgmqtravQGMLDFDYvcsrdepsva<br>ivwgmqtravQGMLDFDYvcsrdepsva<br>ivwgmqtravQGMLDFDYvcsrdepsva<br>iiwgmqtravQGMLDFDYicsrdepsva<br>ivwgmqtravQGMLDFDYicsrsepsva<br>ivwgmqtravQGMLDFDYvcsrqepsva<br>iiwgqqakaiQGMLDFDYvcrrsspsvv<br>ivwgmqqravQSMLDFDFicrrdepsvv<br>............................	Hs_ENSP00000384418<br>Bt_ENSBTAP00000022256<br>Rn_ENSRNOP00000023447<br>Mm_ENSMUSP00000103012<br>Gg_ENSGALP00000005492<br>Xt_ENSXETP00000004892<br>Dr_ENSDARP00000044161<br>Ce_CE06997<br>Dm_FBpp0289823<br>Sc	NP_001087 ATP-citrate synthase (EC 2.3.3.8)(ATP-citrate (pro-S-)-lyase)(Citrate cleavage enzyme)
209	1	CI	ENSP00000264741<br>ENSG00000144668	195<br>55<br>-	kkygeehgscQAGIAGFFteelvvmgap<br>kkygeehgscQAGIAGFFteelvvmgap<br>............................	Hs_ENSP00000264741<br>Bt_ENSBTAP00000022042<br>Rn	NP_002198 Integrin alpha-9 Precursor (Integrin alpha-RLC)

				196<br>189<br>179<br>-<br>-<br>-<br>-	kkygeehgscQAGIAGFFteelvvmgap<br>kkygeehgscQAGIAGFFteelvimgap<br>kkygeehgscqagiagsfmeelvvmgap<br>............................<br>............................<br>............................<br>............................	Mm_ENSMUSP00000044227<br>Gg_ENSGALP00000019958<br>Xt_ENSXETP00000050595<br>Dr<br>Ce<br>Dm<br>Sc	
210	1	C	ENSP00000374014<br>ENSG00000213996	268<br>268<br>-<br>268<br>-<br>168<br>265<br>-<br>-<br>-	lrdpvaypkvQMLMYMFYvlpfcglaay<br>lrdpvtypkvqmlvnmfyvlpfyglaiy<br>............................<br>lrdpvaypklQMLMYLFYalpfyclaay<br>............................<br>lkdpvaypkvqmlvfmfyvlpflcmsvy<br>lkdpvgfpkvmmllllfhalpmlasfay<br>............................<br>............................<br>............................	Hs_ENSP00000374014<br>Bt_ENSBTAP00000021354<br>Rn<br>Mm_ENSMUSP00000105788<br>Gg<br>Xt_ENSXETP00000031350<br>Dr_ENSDARP00000040600<br>Ce<br>Dm<br>Sc	NP_001001524 Transmembrane 6 superfamily member 2
211	1	CI	ENSP00000378199<br>ENSG00000126803	350<br>-<br>350<br>350<br>350<br>-<br>-<br>-<br>-<br>-	vggstripkiQKLLQDFFngkelnksin<br>............................<br>vggstripkiQKLLQDFFngkelnksin<br>vggstripkiQKLLQDFFngkelnksin<br>vggstripkiQKLLQDFFngkelnksin<br>............................<br>............................<br>............................<br>............................<br>............................	Hs_ENSP00000378199<br>Bt<br>Rn_ENSRNOP00000008504<br>Mm_ENSMUSP00000079306<br>Gg_ENSGALP00000019120<br>Xt<br>Dr<br>Ce<br>Dm<br>Sc	NP_068814 Heat shock-related 70 kDa protein 2 (Heat shock 70 kDa protein 2)
212	1	CI	ENSP00000364801<br>ENSG00000204388	347<br>347<br>348<br>348<br>-<br>-<br>-<br>-<br>-<br>-	vggstripkvQKLLQDFFngrdlnksin<br>vggstripkvQKLLQDFFngrdlnksin<br>vggstripkvQKLLQDFFngrdlnksin<br>vggstripkvQKLLQDFFngrdlnksin<br>............................<br>............................<br>............................<br>............................<br>............................<br>............................	Hs_ENSP00000364801<br>Bt_ENSBTAP00000017500<br>Rn_ENSRNOP00000050605<br>Mm_ENSMUSP00000084586<br>Gg<br>Xt<br>Dr<br>Ce<br>Dm<br>Sc	NP_005336 Heat shock 70 kDa protein 1 (HSP70.1)(HSP70-1/HSP70-2)
213	1	C	ENSP00000354070<br>ENSG00000197520	101<br>-<br>-<br>-<br>-<br>-<br>108<br>-<br>122<br>-	vffgltqpkyQYVLNEFYriqnkksdnk<br>............................<br>............................<br>............................<br>............................<br>............................<br>slfgitsakyqyaideysrsk------r<br>............................<br>hllgitsskyaselenyhrakehgdedl<br>............................	Hs_ENSP00000354070<br>Bt<br>Rn<br>Mm<br>Gg<br>Xt<br>Dr_ENSDARP00000088629<br>Ce<br>Dm_FBpp0070975<br>Sc	NP_997351 Protein FAM177B
214	1	C	ENSP00000275053<br>ENSG00000146263	129<br>129<br>0<br>127<br>130<br>-<br>-<br>-<br>-<br>-	lhckadnirqQCVLFLHYvkvfifrylk<br>lhckahnirqlcvtflhyvkvfifrslq<br>----------------------------<br>lhakadsirqqcvvflhyikvfifrclk<br>lhseaenirrlcinflhyvkvfifryle<br>............................<br>............................<br>............................<br>............................<br>............................	Hs_ENSP00000275053<br>Bt_ENSBTAP00000000830<br>Rn_ENSRNOP00000009545<br>Mm_ENSMUSP00000103857<br>Gg_ENSGALP00000024966<br>Xt<br>Dr<br>Ce<br>Dm<br>Sc	NP_940870 Uncharacterized protein C6orf167
215	1	CI	ENSP00000275874<br>ENSG00000146955	47<br>47<br>47<br>47<br>43<br>45<br>43<br>-<br>-<br>-	fksgvytetqQNTIGVDFtvrsldidgk<br>fksgvymeaqQNTIGVDFtvraleidgk<br>fksgvysesqQNTIGVDFtvrsleidgk<br>fksgvysesqQNTIGVDFtvrsleidgk<br>fktgqfyekqQNTIGVDFtvrsmdidgk<br>fqsgvfahnqQNTIGVDFtvrnmningk<br>frsaevtelqhntigvdftvrsmdvdgr<br>............................<br>............................<br>............................	Hs_ENSP00000275874<br>Bt_ENSBTAP00000016258<br>Rn_ENSRNOP00000012280<br>Mm_ENSMUSP00000031986<br>Gg_ENSGALP00000020930<br>Xt_ENSXETP00000005753<br>Dr_ENSDARP00000080189<br>Ce<br>Dm<br>Sc	NP_001008749 RAB19, member RAS oncogene family
216	1	CI	ENSP00000279934<br>ENSG00000150403	118<br>118<br>118	nsvfqavyglQRALQGDYkdvvnmkess<br>nsvfqavhglQRALQGDYrdvanmkess<br>nsvfqaiyglQRALQGDYrdvvnmkess	Hs_ENSP00000279934<br>Bt_ENSBTAP00000020850<br>Rn_ENSRNOP00000026281	NP_060375 Transmembrane and coiled-coil domain-containing protein 3 Precursor

				118	nsifqaiyglQRALQGDYrdvvnmkess	Mm_ENSMUSP00000040347	(Putative LAG1-interacting protein)
				119	nsvfqaihglQRALQGDYkdvvnmkess	Gg_ENSGALP00000027112	
				120	nsvfqavhglrralqgdykdvvnmkess	Xt_ENSXETP00000038550	
				-	....	Dr	
				-	....	Ce	
				-	....	Dm	
				-	....	Sc	
217	1	CI	ENSP00000368539 ENSG00000148429	324	lsmeelveffQETLAKDFffeddfvieq	Hs_ENSP00000368539	NP_001073960 USP6 N-terminal-like protein (Related to the N-terminus of tre)(RN-tre)
				324	lsmeelveflQETLAKDFffeddfvieq	Bt_ENSBTAP00000025957	
				347	lsmeelveflQETLAKDFffeddfvieq	Rn_ENSRNOP00000046740	
				347	lsmeelveflQETLAKDFffeddfvieq	Mm_ENSMUSP00000043178	
				326	lqmeelveflQETLAKDFfyeddyvieq	Gg_ENSGALP00000010831	
				-	....	Xt	
				-	....	Dr	
				-	....	Ce	
				-	....	Dm	
				-	....	Sc	
218	1	CI	ENSP00000381867 ENSG00000172046	720	lylpvplpqkQKVLPVFYfarephskpi	Hs_ENSP00000381867	Ubiquitin carboxyl-terminal hydrolase 19 (EC 3.1.2.15) (Ubiquitin thioesterase 19) (Ubiquitin-specific-processing protease 19)(Deubiquitinating enzyme 19)(Zinc finger MYND domain-containing protein 9)
				762	lylpvplpqkQKVLPVFYfarephskpi	Bt_ENSBTAP00000025198	
				-	....	Rn	
				761	lylpvplpqkQKVLPIFYfarephskpi	Mm_ENSMUSP00000082119	
				-	....	Gg	
				713	lylpvplpqkqkvltvyyfarephkkpi	Xt_ENSXETP00000018836	
				185	lylpvplpqk------------------	Dr_ENSDARP00000011581	
				606	vylpvpfpknkqstdlifwplenqtkpy	Ce_CE03146	
				-	....	Dm	
				-	....	Sc	
219	1	CI	ENSP00000296543 ENSG00000164134	377	eeppttllwvQYYLAQHYdkigqpsial	Hs_ENSP00000296543	NP_476516 NMDA receptor-regulated protein 1 (N-terminal acetyltransferase)(Protein tubedown-1)(Tbdn100)(Gastric cancer antigen Ga19)
				378	eeppttllwvQYYLAQHYdkigqpsial	Bt_ENSBTAP00000045426	
				359	eeppttllwvQYYLAQHYdkigqpsial	Rn_ENSRNOP00000017379	
				377	eeppttllwvQYYLAQHYdkigqpsial	Mm_ENSMUSP00000029303	
				364	eeppttllwvQYYLAQHYdkigqpslal	Gg_ENSGALP00000015892	
				360	eeppttllwvQYYLAQHYdkigqpsial	Xt_ENSXETP00000036812	
				378	eeppttllwvQYFLAQHYdqigqhsmal	Dr_ENSDARP00000055740	
				386	ceppttalwlyvlashhfdrcgmtqlal	Ce_CE35099	
				380	vepasalvwtalflaqhydymrdtdral	Dm_FBpp0074480	
				-	....	Sc	
220	1	CI	ENSP00000350386 ENSG00000188419	425	kckaiidqfgQRIISEHFlvedsyfpen	Hs_ENSP00000350386	NP_000381 Rab proteins geranylgeranyltransferase component A 1 (Rab escort protein 1)(REP-1) (Choroideraemia protein)(TCD protein)
				386	kckaiidqygQRIISKHFlvedsylsen	Bt_ENSBTAP00000012631	
				406	kckavidqfgQRIISKHFiiedsylsen	Rn_ENSRNOP00000000174	
				434	kckaivdqfgQRIISKHFviedsylsen	Mm_ENSMUSP00000026607	
				-	....	Gg	
				-	....	Xt	
				-	....	Dr	
				328	gkitaviangdrvncryivmsprfvpet	Ce_CE27306	
				299	--efllssagktlraknvvsapgytpvs	Dm_FBpp0085658	
				-	....	Sc	
221	1	CI	ENSP00000227378 ENSG00000109971	347	vggstripkiQKLLQDFFngkelnksin	Hs_ENSP00000227378	NP_006588 Heat shock cognate 71 kDa protein (Heat shock 70 kDa protein 8)
				348	vggstripkiQKLLQDFFngkelnksin	Bt_ENSBTAP00000017497	
				311	vggstripkiQKLLQDFFngkelnksin	Rn_ENSRNOP00000042159	
				347	vggstripkiQKLLQDFFngkelnksin	Mm_ENSMUSP00000015800	
				348	vggstripkiQKLLQDFFngkelnksin	Gg_ENSGALP00000040138	
				347	vggstripkiQKLLQDFFngkelnksin	Xt_ENSXETP00000028570	
				348	vggstripkiqkllqdyfngkelnksin	Dr_ENSDARP00000090766	
				348	vggstripkvqkllsdlfsgkelnksin	Ce_CE09682	
				347	vggstripkvqrllqdlfngkelnksin	Dm_FBpp0082514	
				344	vggstripkvqklvtdyfngkepnrsin	Sc_YAL005C	
222	1	CI	ENSP00000306726 ENSG00000171208	518	iyvrgredsaQASISIDF----------	Hs_ENSP00000306726	NP_060562 Neuropilin and tolloid-like protein 2 Precursor (Brain-specific transmembrane protein containing 2 CUB and 1 LDL-receptor class A domains protein 2)
				519	iyvrgredsaQASISIDF----------	Bt_ENSBTAP00000019107	
				507	iyvrgrddsaQASISIDF----------	Rn_ENSRNOP00000021739	
				546	iyvrgrddsaQASISIDF----------	Mm_ENSMUSP00000105308	
				518	iyvrgredtaQGSLSIDF----------	Gg_ENSGALP00000006464	
				509	iyvrgredcaQGSMSIDF----------	Xt_ENSXETP00000008492	
				528	vfgrgrgvvmQRSLSMDF----------	Dr_ENSDARP00000086783	
				409	----------------------------	Ce_CE40087	
				-	....	Dm	
				-	....	Sc	
223	1	CI	ENSP00000230221 ENSG00000112459	94	lmgngyislvQCILQVFFfialasseva	Hs_ENSP00000230221	NP_112208 Olfactory receptor 14J1 (Olfactory receptor 5U1)
				123	lmnngyisldQCILQVFFfialasseva	Bt_ENSBTAP00000048959	
				93	lmdngfislgQCMLQVFFfialasseva	Rn_ENSRNOP00000000976	

				93	lmdngfislgQCMLQVFFfialasseva	Mm_ENSMUSP00000055221	(Olfactory receptor OR6-25) (Hs6M1-28)
				97	lwhtrhisyagcaaqvffffflfgaeyc	Gg_ENSGALP00000035270	
				-	....	Xt	
				-	....	Dr	
				-	....	Ce	
				-	....	Dm	
				-	....	Sc	
224	1	CI	ENSP00000354039 ENSG00000137073	832	iygydelqmlQSRLPVDYygipfaapta	Hs_ENSP00000354039	NP_060919 Ubiquitin-associated protein 2
				848	iygydelqmlQSRLPMDYygipfatppa	Bt_ENSBTAP00000000930	
				841	iygydelqmlQSRLPMDYygipfaapta	Rn_ENSRNOP00000015920	
				846	iygydelqmlQSRLPMDYygipfaapta	Mm_ENSMUSP00000030143	
				847	iygyddlqmlQSRLPMDYygitfpapat	Gg_ENSGALP00000009276	
				849	iygyddlqmlQSRLPMDYygitfpapat	Xt_ENSXETP00000004605	
				862	iygyedlhmlQSRLPMDYygitfpgpta	Dr_ENSDARP00000086506	
				-	....	Ce	
				966	nnsssassvgaatvaqtatgttaavlas	Dm_FBpp0100030	
				-	....	Sc	
225	1	CI	ENSP00000282041 ENSG00000152223	2067	klpwkdlhpdQMLMEAFFk--vergspk	Hs_ENSP00000282041	NP_066015 UPF0493 protein KIAA1632
				1069	klpwrdlhpdQMLMEAFFk--vergspk	Bt_ENSBTAP00000016012	
				1065	klewkdlhpdQRLMEAFFkncgecgmke	Rn_ENSRNOP00000022767	
				1065	rlewkdlhpdQRLMEAFFk--vergspk	Mm_ENSMUSP00000038681	
				1073	rlpwkemhpdQMLMEEFFk--vergspk	Gg_ENSGALP00000002656	
				-	....	Xt	
				-	....	Dr	
				1071	riafvndthklyeitgiifqayqqqlaa	Ce_CE02566	
				1935	qlpwerflppaqhvellyd--slqkflp	Dm_FBpp0271890	
				-	....	Sc	
226	1	C	ENSP00000261745 ENSG00000111300	771	mggffnsgcsQCQISSFYlvndiyeldt	Hs_ENSP00000261745	NP_079229 TPR repeat-containing protein C12orf30 (p120)
				771	magffnsgcsqcqtssfylvsdiyeldi	Bt_ENSBTAP00000008756	
				771	mgrffssgccqcqvhsfhlvsdvyeldt	Rn_ENSRNOP00000001823	
				771	mgrffssgccqcqvqsfhlvsdmyeldt	Mm_ENSMUSP00000038977	
				771	magffsngscqcqtslfylvsdiyeldt	Gg_ENSGALP00000007621	
				765	lskflssgacqcqkeallllndiydldm	Xt_ENSXETP00000009362	
				-	....	Dr	
				760	lqmvleyleaavklvdildsgehpeksl	Ce_CE31579	
				753	lhllldmpyerffddlaqlvldlqsgsa	Dm_FBpp0083238	
				662	leellnnnltevesasfliffeiyenng	Sc_YOL076W	
227	1	CI	ENSP00000313346 ENSG00000132199	376	aggvglcelvQHLIIFDYisvsaslenr	Hs_ENSP00000313346	Mitochondrial enolase superfamily member 1 (Antisense RNA to thymidylate synthase)(rTS)
				366	aggvglcelvQHLIIFDFisvsaslqdr	Bt_ENSBTAP00000031013	
				359	wwswtlnwlvqhliifdcqslpafktgr	Rn_ENSRNOP00000053091	
				-	....	Mm	
				-	....	Gg	
				366	aggvglcelvQHLILFDYisvsgsldnr	Xt_ENSXETP00000006867	
				365	aggvglcelvQHLILFDYisvsaslsnr	Dr_ENSDARP00000055955	
				-	....	Ce	
				-	....	Dm	
				-	....	Sc	
228	1	CI	ENSP00000386694 ENSG00000135905	2127	rlikedqleyQEELRSHYkdmlselstv	Hs_ENSP00000386694	NP_055504 Dedicator of cytokinesis protein 10 (Zizimin-3)
				2092	rlikedqleyQEELRSHYkdmlselsai	Bt_ENSBTAP00000037982	
				1749	rlikedqleyQEELRSHYkdmlgelsai	Rn_ENSRNOP00000021603	
				2096	rlikedqleyQEELRSHYkdmlselsai	Mm_ENSMUSP00000095286	
				2098	rlikedqfeyQGEMKSHYrdmlselsav	Gg_ENSGALP00000008043	
				2093	rlikedqleyQEEMKSHYreilselsev	Xt_ENSXETP00000011184	
				-	....	Dr	
				-	....	Ce	
				-	....	Dm	
				-	....	Sc	
229	1	CI	ENSP00000355541 ENSG00000119285	1864	vmkkeeltshQSQLTAFFlealdfraqh	Hs_ENSP00000355541	NP_060542 HEAT repeat-containing protein 1 (Protein BAP28)
				-	....	Bt	
				1863	vmkkeellshQSQLTTFFlealdfraqh	Rn_ENSRNOP00000034550	
				1863	vmkkeellshQSQLTTFFlealdfraqh	Mm_ENSMUSP00000054084	
				1882	vmekehlishQVELTAFFmkaldyrteh	Gg_ENSGALP00000000337	
				1879	imekqelsshQSELTAFFlkalnyraeh	Xt_ENSXETP00000019745	
				1867	hmdkdqlnnhQSELTSFFlsaldfraqh	Dr_ENSDARP00000066445	
				1360	ilrksviqlrrtfvsdvitptlivrsqe	Ce_CE05078	
				1823	hnsaaqlqpvqdplselflqalnfrlqv	Dm_FBpp0078971	
				1497	nidkksatsqspiffklllslfefrsis	Sc_YJL109C	
230	1	CI	ENSP00000334879 ENSG00000186212	621	qvwtlfwedpQLALHKDFltgytalhwi	Hs_ENSP00000334879	NP_001025041 Ankyrin repeat domain-containing protein 56
				-	....	Bt	
				595	hvqmlfwedpQLALYRDFltgytalhwi	Rn_ENSRNOP00000002995	

				588<br>285<br>-<br>-<br>-<br>-<br>-	hvltlfwedpQLALHRDFltgytalhwi<br>hvrglfleepelalqrdfmsgftvlhwl<br>............................<br>............................<br>............................<br>............................<br>............................	Mm_ENSMUSP00000055267<br>Gg_ENSGALP00000037124<br>Xt<br>Dr<br>Ce<br>Dm<br>Sc	
231	1	CI	ENSP00000386208<br>ENSG00000221836	271<br>270<br>270<br>270<br>-<br>-<br>-<br>-<br>-<br>-	apksrhpeeqQKVLSLFYslfnpmlnpl<br>apksrhpeeqQKILSLFYslfnpmlnpl<br>apktqhpetqQKILSLFYslfnpmlnpl<br>apksqhpetqQKVLSLFYslfnpmlnpl<br>............................<br>............................<br>............................<br>............................<br>............................<br>............................	Hs_ENSP00000386208<br>Bt_ENSBTAP00000049075<br>Rn_ENSRNOP00000007163<br>Mm_ENSMUSP00000059195<br>Gg<br>Xt<br>Dr<br>Ce<br>Dm<br>Sc	NP_036497 Olfactory receptor 2A5 (Olfactory receptor 7-138/7-141)(OR7-138)(OR7-141)
232	1	C	ENSP00000345102<br>ENSG00000188305	221<br>224<br>342<br>-<br>171<br>5<br>-<br>-<br>-<br>-	dvphpwglelQASLSPHFnlqglcglvp<br>eethtwglelQVSLRPHFnlqglcgllp<br>sqqyyhslavrqslpvhfniqqdcghfl<br>............................<br>lkpgccttemqsslgshcsiqriiscft<br>-----------lslpvhfniqqdcgnfv<br>............................<br>............................<br>............................<br>............................	Hs_ENSP00000345102<br>Bt_ENSBTAP00000021901<br>Rn_ENSRNOP00000022566<br>Mm<br>Gg_ENSGALP00000040423<br>Xt_ENSXETP00000037256<br>Dr<br>Ce<br>Dm<br>Sc	NP_940934 Putative uncharacterized protein C19orf35
233	1	CI	ENSP00000318914<br>ENSG00000176903	346<br>346<br>346<br>346<br>-<br>305<br>-<br>-<br>-<br>-	eeeeeaeatllQLGLEGHF---------<br>eeaaaeaallQLGLEGHF----------<br>eeeeeaeaallQLGLEGHF---------<br>eeeeeaeaallQLGLEGHF---------<br>............................<br>----------------------------<br>............................<br>............................<br>............................<br>............................	Hs_ENSP00000318914<br>Bt_ENSBTAP00000012212<br>Rn_ENSRNOP00000014018<br>Mm_ENSMUSP00000060783<br>Gg<br>Xt_ENSXETP00000050115<br>Dr<br>Ce<br>Dm<br>Sc	NP_006020 Paraneoplastic antigen Ma1 (Neuron- and testis-specific protein 1)(37 kDa neuronal protein)
234	1	I	ENSP00000318997<br>ENSG00000176198	212<br>234<br>216<br>211<br>-<br>-<br>-<br>-<br>-<br>-	apitecifytQSSLVLFFtsmyilrsyi<br>apitefifytQSSLVLFFtimyilrsyt<br>apitefifyaqssfvlfftisyilrsyi<br>apitefifyaqssfvlfftiayilrsyi<br>............................<br>............................<br>............................<br>............................<br>............................<br>............................	Hs_ENSP00000318997<br>Bt_ENSBTAP00000049172<br>Rn_ENSRNOP00000041674<br>Mm_ENSMUSP00000074242<br>Gg<br>Xt<br>Dr<br>Ce<br>Dm<br>Sc	NP_001004479 Olfactory receptor 11H4 (Olfactory receptor OR14-36)
235	1	CI	ENSP00000283147<br>ENSG00000153162	356<br>-<br>349<br>353<br>162<br>284<br>-<br>-<br>-<br>-	lvgrdgpydkQPFMVAFFkvsevhvrtt<br>............................<br>lvgrdgpydkQPFMVAFFkvsevhvrtt<br>lvgrdgpydkQPFMVAFFkvsevhvrtt<br>ligrdgpydkQPFMVAFFkvsevhvrtt<br>lvgrdgpydkQPFMVAFFkasevhlrtt<br>............................<br>............................<br>............................<br>............................	Hs_ENSP00000283147<br>Bt<br>Rn_ENSRNOP00000018359<br>Mm_ENSMUSP00000048158<br>Gg_ENSGALP00000020833<br>Xt_ENSXETP00000053087<br>Dr<br>Ce<br>Dm<br>Sc	NP_001709 Bone morphogenetic protein 6 Precursor (BMP-6)
236	1	CI	ENSP00000239938<br>ENSG00000120738	248<br>248<br>249<br>246<br>220<br>218<br>226<br>-<br>-<br>-	paakgg---fQVPMIPDYlfpqqqgdlg<br>pgakgg---fQVPMIPDYlfpqqqgdlg<br>patkgg---fQVPMIPDYlfpqqqgdls<br>patkgg---fQVPMIPDYlfpqqqgdls<br>ptaktn---fQVPMIPDYlfpqqqseln<br>pvsktt---fQVPMIPDYlfpqqqgdvs<br>psaktcnpsfsvpmipdylftqqqseis<br>............................<br>............................<br>............................	Hs_ENSP00000239938<br>Bt_ENSBTAP00000013284<br>Rn_ENSRNOP00000026303<br>Mm_ENSMUSP00000069616<br>Gg_ENSGALP00000012417<br>Xt_ENSXETP00000047681<br>Dr_ENSDARP00000054459<br>Ce<br>Dm<br>Sc	NP_001955 Early growth response protein 1 (EGR-1)(Protein Krox-24)(Transcription factor Zif268)(Nerve growth factor-induced protein A)(NGFI-A)(Transcription factor ETR103)(Zinc finger protein 225)(AT225)
237	1	CI	ENSP00000314488<br>ENSG00000164953	631<br>-<br>631	alqflhklisQITIDVFFidwerpkgkv<br>............................<br>alqflhklisQITIDIFFidwerpkgkv	Hs_ENSP00000314488<br>Bt<br>Rn_ENSRNOP00000021839	NP_714915 Meckelin (Meckel syndrome type 3 protein)(Transmembrane protein 67)

				697<br>602<br>603<br>-<br>-<br>608<br>-	alqflhkfisQISIDIFFidwerpkgkv<br>alqflqllvsqltvdiffidwerpkgrv<br>alqllykmtllltidvffidwerpkart<br>............................<br>............................<br>flflgihlwrssqlelflidwerprssc<br>............................	Mm_ENSMUSP00000103928<br>Gg_ENSGALP00000025642<br>Xt_ENSXETP00000032198<br>Dr<br>Ce<br>Dm_FBpp0112166<br>Sc	
238	1	CI	ENSP00000160382<br>ENSG00000077080	140<br>140<br>140<br>140<br>-<br>-<br>-<br>149<br>148<br>-	rekltelmfeQYNIPAFFlcktavlta-<br>rekltelmfeQYNIPAFFlcktavltay<br>rekltelmfeQYNIPAFFlcktavlta-<br>rekltelmfeQYNIPAFFlcktavlta-<br>............................<br>............................<br>............................<br>rekltelmfekfqvpayylsknvvlsc-<br>rekltelmfekynvpafflvknavlaa-<br>............................	Hs_ENSP00000160382<br>Bt_ENSBTAP00000010915<br>Rn_ENSRNOP00000001910<br>Mm_ENSMUSP00000031725<br>Gg<br>Xt<br>Dr<br>Ce_CE31753<br>Dm_FBpp0086115<br>Sc	NP_057272 Actin-like protein 6B (53 kDa BRG1-associated factor B)(Actin-related protein Baf53b)(ArpNalpha)
239	1	CI	ENSP00000356236<br>ENSG00000143858	144<br>-<br>147<br>147<br>-<br>-<br>-<br>-<br>-<br>-	ekepenlgklQFSLDYDFqanqltvgvl<br>............................<br>ekepenlgklQFSLDYDFqanqltvgvl<br>ekepenlgklQFSLDYDFqanqltvgvl<br>............................<br>............................<br>............................<br>............................<br>............................<br>............................	Hs_ENSP00000356236<br>Bt<br>Rn_ENSRNOP00000006637<br>Mm_ENSMUSP00000027721<br>Gg<br>Xt<br>Dr<br>Ce<br>Dm<br>Sc	NP_796376 Synaptotagmin-2 (Synaptotagmin II)(SytII)
240	1	CI	ENSP00000342962<br>ENSG00000111897	167<br>167<br>-<br>167<br>175<br>190<br>194<br>170<br>181<br>-	magafcfiliQLVLLIDFahswneswve<br>magafcfiliQLVLLIDFahswneswvv<br>............................<br>magafcfiliQLVLLIDFahswneswve<br>msgafcfiliQLVLLIDFahswneswve<br>mggafcfiliQLVLLIDFahswneswve<br>magafcfiliQLVLLIDFahswneswve<br>liggfmfiliQLILIVDFahglaeawvt<br>ligglafilvQLVIIVDFahslaenwie<br>............................	Hs_ENSP00000342962<br>Bt_ENSBTAP00000049304<br>Rn<br>Mm_ENSMUSP00000020027<br>Gg_ENSGALP00000023943<br>Xt_ENSXETP00000050322<br>Dr_ENSDARP00000027703<br>Ce_CE29890<br>Dm_FBpp0075147<br>Sc	NP_065806 Serine incorporator 1 (Tumor differentially expressed protein 2)(Tumor differentially expressed 1 protein-like)
241	1	CI	ENSP00000311486<br>ENSG00000174206	15<br>15<br>15<br>15<br>15<br>13<br>17<br>-<br>-<br>-	pla-apvpveQAVLETFFshlgifsydk<br>pla-apvpveQAVLETFFshlgifsydk<br>pla-apvpveQAVLETFFshlgifsydk<br>pla-apvpveQAVLETFFshlgifsydk<br>plg-apvpveQAVLETFFshlgifsydk<br>pltsaqvtaeQNVLHTFFshlgnfsydk<br>deelrpvpkeravlesfftqlgmfwfdr<br>............................<br>............................<br>............................	Hs_ENSP00000311486<br>Bt_ENSBTAP00000017932<br>Rn_ENSRNOP00000039477<br>Mm_ENSMUSP00000070834<br>Gg_ENSGALP00000015962<br>Xt_ENSXETP00000046844<br>Dr_ENSDARP00000067309<br>Ce<br>Dm<br>Sc	NP_689653 UPF0536 protein C12orf66
242	1	CI	ENSP00000380692<br>ENSG00000006459	635<br>571<br>-<br>635<br>575<br>-<br>580<br>-<br>-<br>-	gvegveheesQKPLNGFFtrvkselrsr<br>giegveneesqkplnrfltsvkselrnr<br>............................<br>greeaesqgspkplnriftsvrselrsr<br>gfegegnadaQKPLNMFFesvkselrkg<br>............................<br>----------------------------<br>............................<br>............................<br>............................	Hs_ENSP00000380692<br>Bt_ENSBTAP00000033407<br>Rn<br>Mm_ENSMUSP00000002305<br>Gg_ENSGALP00000020923<br>Xt<br>Dr_ENSDARP00000050378<br>Ce<br>Dm<br>Sc	NP_085150 JmjC domain-containing histone demethylation protein 1D
243	1	CI	ENSP00000295373<br>ENSG00000163214	368<br>360<br>371<br>368<br>366<br>-<br>0<br>-<br>310<br>-	rfskdhkypyQAPLVAFYstnenlplac<br>rfskdhkypyQAPLVAFYstnenlplac<br>rfskdhkypyQAPLVAFYstnenlplac<br>rfskdhkypyQAPLVAFYstnenlplac<br>rfpeenkyplQAPLVAFYttdenlplac<br>............................<br>----------------------------<br>............................<br>rfppgsrypyeapfiylkttchdiphel<br>............................	Hs_ENSP00000295373<br>Bt_ENSBTAP00000019540<br>Rn_ENSRNOP00000030935<br>Mm_ENSMUSP00000083742<br>Gg_ENSGALP00000023331<br>Xt<br>Dr_ENSDARP00000074801<br>Ce<br>Dm_FBpp0073259<br>Sc	NP_945314 Putative ATP-dependent RNA helicase DHX57 (EC 3.6.1.-)(DEAH box protein 57)
244	1	CI	ENSP00000233190<br>ENSG00000023228	688<br>688<br>688	qlladplvppQLTIKDFYmtdsisrasq<br>qlladplvppQLTIKDFYmtdsisrasq<br>efladplvppQLTIKDFYmtdsisrasq	Hs_ENSP00000233190<br>Bt_ENSBTAP00000029301<br>Rn_ENSRNOP00000015851	NP_004997 NADH-ubiquinone oxidoreductase 75 kDa subunit, mitochondrial Precursor (EC

				688<br>689<br>687<br>637<br>686<br>691<br>-	evladplvppQLTIKDFYmtdsisrasq<br>qlladplvppQLTIKDFYmtdsisrasq<br>qiladplvppQLTIKDFYmtdpitrasq<br>sllaaplvppqltvkdfymtdpisrasp<br>-sidvdvspvlrelsdyyqtnvisrysr<br>gaidiklk----elrdyfmtdaisrasp<br>............................	Mm_ENSMUSP00000027111<br>Gg_ENSGALP00000013999<br>Xt_ENSXETP00000022294<br>Dr_ENSDARP00000028571<br>Ce_CE21933<br>Dm_FBpp0071128<br>Sc	1.6.5.3)(EC 1.6.99.3)(Complex I-75kD)(CI-75kD)
245	1	CI	ENSP00000347710<br>ENSG00000079482	418<br>-<br>375<br>418<br>418<br>418<br>419<br>-<br>-<br>370	yrtvgsniqvQKLLNAFFdpkcpgdvdf<br>............................<br>yrtvgsniqvQKLLNAFFdpkcpgdvdf<br>yrtvgsniqvQKLLYAFFdpkcpgdvdf<br>yrtvgsniqvQKLLNAFFdpkcpgdvdl<br>yrtvgsniqvQKLLNAFFdpkcpgnvdl<br>yrtvgsniqvQKLLNAFFdskcpadvdf<br>............................<br>............................<br>spflssssntaavpnanlnsatp---sl	Hs_ENSP00000347710<br>Bt<br>Rn_ENSRNOP00000035867<br>Mm_ENSMUSP00000033560<br>Gg_ENSGALP00000007289<br>Xt_ENSXETP00000011092<br>Dr_ENSDARP00000051333<br>Ce<br>Dm<br>Sc_YBR260C	NP_002538 Oligophrenin 1
246	1	CI	ENSP00000378044<br>ENSG00000145362	1397<br>1364<br>1370<br>-<br>1340<br>-<br>1374<br>1410<br>-<br>-	gnlvpltksgQHHIFSFFafkenrlplf<br>gnlvpltksgQHHIFSFFafkenrlplf<br>gnlvpltksgQHHIFSFFafkenrlplf<br>............................<br>gnlvpltksgQHHIFSFFafkenrlplf<br>............................<br>gnlvpltksgqhhvfsfyafkenrlalf<br>gnlvpitksgdqlslfflpfqenrlafm<br>............................<br>............................	Hs_ENSP00000378044<br>Bt_ENSBTAP00000003097<br>Rn_ENSRNOP00000052477<br>Mm<br>Gg_ENSGALP00000019635<br>Xt<br>Dr_ENSDARP00000063587<br>Ce_CE31847<br>Dm<br>Sc	Ankyrin-2 (Brain ankyrin)(Ankyrin-B)(Non-erythroid ankyrin)
246	2	C	ENSP00000378044<br>ENSG00000145362	3746<br>3710<br>2032<br>-<br>3511<br>-<br>3273<br>4587<br>-<br>-	lfpqthk----eqvqqdfsgkmq---dl<br>llpqtqk----eqvqqdfsgkmq---dl<br>----------------------------<br>............................<br>llrqthk----eqveaefsgkpq---dv<br>............................<br>vvgdsthsfemvglrqqfpgtikqgddm<br>virqyhdeppqeieeqtipeevtvlrev<br>............................<br>............................	Hs_ENSP00000378044<br>Bt_ENSBTAP00000003097<br>Rn_ENSRNOP00000052477<br>Mm<br>Gg_ENSGALP00000019635<br>Xt<br>Dr_ENSDARP00000063587<br>Ce_CE31847<br>Dm<br>Sc	Ankyrin-2 (Brain ankyrin)(Ankyrin-B)(Non-erythroid ankyrin)
247	1	CI	ENSP00000260983<br>ENSG00000138411	1321<br>1197<br>1327<br>1327<br>1325<br>-<br>1302<br>-<br>-<br>-	grilglalihQYLLDAFFtrpfykallr<br>grilglalihQYLLDAFFtrpfykallr<br>grilglalihQYLLDAFFtrpfykallr<br>grilglalihQYLLDAFFtrpfykallr<br>grilglalihQYLLDAFFtrpfykallr<br>............................<br>grilglalihQYLLDAFFtrpfykgllr<br>............................<br>............................<br>............................	Hs_ENSP00000260983<br>Bt_ENSBTAP00000038122<br>Rn_ENSRNOP00000017761<br>Mm_ENSMUSP00000113283<br>Gg_ENSGALP00000012889<br>Xt<br>Dr_ENSDARP00000086672<br>Ce<br>Dm<br>Sc	NP_065811 E3 ubiquitin-protein ligase HECW2 (EC 6.3.2.-)(HECT, C2 and WW domain-containing protein 2)(NEDD4-like E3 ubiquitin-protein ligase 2)
248	1	CI	ENSP00000368460<br>ENSG00000067992	148<br>148<br>148<br>148<br>148<br>148<br>147<br>-<br>154<br>-	-fdpfistniQYFLDRFYtnrisfrmli<br>-fdpfissniQYFLDRFYtnrisfrmli<br>-fdpfissniQYFLDRFYtnrisfrmli<br>-fdpfissniQYFLDRFYtnrisfrmli<br>-fdpfvssniQYFLDRFYtnrisfrmli<br>-fdpfvssniQYFLDRFYmnrisfrmli<br>-fdpfvssniQYFLDRFYtnrisfrmli<br>............................<br>qvdaptessiqyfldrlymsrisirmli<br>............................	Hs_ENSP00000368460<br>Bt_ENSBTAP00000023706<br>Rn_ENSRNOP00000017370<br>Mm_ENSMUSP00000036604<br>Gg_ENSGALP00000026284<br>Xt_ENSXETP00000047175<br>Dr_ENSDARP00000015430<br>Ce<br>Dm_FBpp0111805<br>Sc	NP_005382 Unknown
249	1	CI	ENSP00000243108<br>ENSG00000197757	97<br>97<br>97<br>97<br>-<br>97<br>97<br>-<br>-<br>-	crqntlghntQTSIAQDFsseqgrtapq<br>crqntlghntQTSIAQDFsseqgrtapq<br>crqntlghntQTSIAQDFsseqgrtapq<br>crqntlghntQTSIAQDFsseqgrtapq<br>............................<br>crqnsmghntQSSLAQDFsseqsrgngq<br>crqtsmglnaqshvaqeynleqaragtq<br>............................<br>............................<br>............................	Hs_ENSP00000243108<br>Bt_ENSBTAP00000012174<br>Rn_ENSRNOP00000022217<br>Mm_ENSMUSP00000001711<br>Gg<br>Xt_ENSXETP00000050686<br>Dr_ENSDARP00000093809<br>Ce<br>Dm<br>Sc	NP_710160 Homeobox protein Hox-C6 (Hox-3C)(HHO.C8)(CP25)
250	1	CI	ENSP00000343656<br>ENSG00000136237	582<br>668<br>658	fikiaahckaQRNLNSFFaivmglntas<br>fikiaahckaQRNLNSFFaivmglntas<br>fikiaahckaQQNLNSFFaivmglntas	Hs_ENSP00000343656<br>Bt_ENSBTAP00000033741<br>Rn_ENSRNOP00000007112	NP_036426 Rap guanine nucleotide exchange factor 5 (Guanine nucleotide exchange

				663<br>664<br>662<br>-<br>-<br>-<br>-	fikiaahckaQQNLNSFFaivmglntas<br>fikiaahckaQRNLNSFFaivmglntas<br>fikiaahckaQRNLNSFFaiimglntas<br>............................<br>............................<br>............................<br>............................	Mm_ENSMUSP00000105313<br>Gg_ENSGALP00000017724<br>Xt_ENSXETP00000016124<br>Dr<br>Ce<br>Dm<br>Sc	factor for Rap1)(Related to Epac)(Repac)(M-Ras-regulated Rap GEF)(MR-GEF)
251	1	CI	ENSP00000343248<br>ENSG00000188322	157<br>-<br>157<br>157<br>147<br>126<br>130<br>234<br>204<br>-	pedtvkrcvqQLGLALDFmhgrqlvhrd<br>............................<br>pedtvkrcvqQLGLALDFmhsrqlvhrd<br>pedtvkrcvqQLGLALDFmhsrqlvhrd<br>pedmvkrcvqQLGLALDYmhskslvhrd<br>pedmvkrcvqQLGLALDFmhskslvhrd<br>peemvkrcvqQLGLALDFmhskslvhrd<br>geantkkvfaavlsaiefmhdenlvhrn<br>henackliseqlssalgfmhsknlvhrd<br>............................	Hs_ENSP00000343248<br>Bt<br>Rn_ENSRNOP00000025808<br>Mm_ENSMUSP00000060907<br>Gg_ENSGALP00000022893<br>Xt_ENSXETP00000022722<br>Dr_ENSDARP00000073232<br>Ce_CE30846<br>Dm_FBpp0078933<br>Sc	NP_001019572 Serine/threonine-protein kinase SBK1 (EC 2.7.11.1)(SH3-binding kinase 1)
252	1	CI	ENSP00000358064<br>ENSG00000163125	1333<br>1334<br>1352<br>1350<br>1340<br>1300<br>1083<br>-<br>822<br>-	vfpkdhssllQGTLAEHFgvlpgprdhg<br>vfpkdhssllQGTLADHFgvlpgprdhg<br>vfpkdhssllQGTMAEHFgvltgpr---<br>vfpkdhssllQGTMAEHFgvltgpr---<br>afpkehgtvhQGTMKEHFnvhagprept<br>pfspdhgnihQ-SMKDHFglgpglqrdl<br>------------------gkrpgpp---<br>............................<br>----------------------------<br>............................	Hs_ENSP00000358064<br>Bt_ENSBTAP00000019948<br>Rn_ENSRNOP00000033119<br>Mm_ENSMUSP00000088297<br>Gg_ENSGALP00000023574<br>Xt_ENSXETP00000040373<br>Dr_ENSDARP00000075341<br>Ce<br>Dm_FBpp0080620<br>Sc	NP_056018 Regulation of nuclear pre-mRNA domain-containing protein 2
253	1	CI	ENSP00000223127<br>ENSG00000106397	316<br>329<br>319<br>319<br>-<br>-<br>307<br>302<br>304<br>-	qptpflprflQRLLLLDYppdrvtlflh<br>qptpflprflQRLLLLDYppdrvtlflh<br>qptpflprflQRLLLLDYppdrislflh<br>qptpflprflQRLLLLDYppdrislflh<br>............................<br>............................<br>qpmpfleeflerlatlnyphtrirlflh<br>kpipfieevlqkiaefdypkekialyiy<br>qpvpffdqflegieslnypkeklhlliy<br>............................	Hs_ENSP00000223127<br>Bt_ENSBTAP00000000447<br>Rn_ENSRNOP00000001921<br>Mm_ENSMUSP00000004968<br>Gg<br>Xt<br>Dr_ENSDARP00000080246<br>Ce_CE03397<br>Dm_FBpp0075917<br>Sc	NP_001075 Procollagen-lysine,2-oxoglutarate 5-dioxygenase 3 Precursor (EC 1.14.11.4)(Lysyl hydroxylase 3)(LH3)
254	1	CI	ENSP00000275358<br>ENSG00000146530	361<br>-<br>361<br>361<br>361<br>-<br>347<br>394<br>-<br>-	nnqppviqalQDKLQTFYgenfeyqfva<br>............................<br>ynqppviqtlQDKLQVFYgedfeyqfia<br>dnqppmiqtlQDKLEAFYgedfeyhfma<br>nnqppvletfQGMLQTFYgedfvyqfla<br>............................<br>kneppvfhpgqstlkafsgedfvyqfsa<br>vcddgffgdkcqysdictsatclyggtc<br>............................<br>............................	Hs_ENSP00000275358<br>Bt<br>Rn_ENSRNOP00000029955<br>Mm_ENSMUSP00000093817<br>Gg_ENSGALP00000017430<br>Xt<br>Dr_ENSDARP00000071942<br>Ce_CE41525<br>Dm<br>Sc	NP_001129396 Putative EGF-like domain-containing protein FLJ14712
255	1	C	ENSP00000371394<br>ENSG00000149043	122<br>113<br>118<br>118<br>-<br>-<br>-<br>-<br>-<br>-	pgdaqqwgclQLSLEFDFgsqeirvglr<br>pggaqqwg-lqlsleydsgsqeirvglk<br>sggdqpwgqlllsleydfgsqeirvglr<br>sggdqqwgrlllsleydfgsqeirvglr<br>............................<br>............................<br>............................<br>............................<br>............................<br>............................	Hs_ENSP00000371394<br>Bt_ENSBTAP00000052023<br>Rn_ENSRNOP00000027454<br>Mm_ENSMUSP00000101598<br>Gg<br>Xt<br>Dr<br>Ce<br>Dm<br>Sc	NP_612634 Synaptotagmin-8 (Synaptotagmin VIII)(SytVIII)
255	2	C	ENSP00000371394<br>ENSG00000149043	226<br>226<br>222<br>222<br>-<br>-<br>-<br>-<br>-<br>-	lrlplgtvdlqhvlehwyllgppaatqp<br>lslplgsvdlqhvlelwrplgppaaaep<br>lqlplgtvdlqhvleswyqlgppgstes<br>lqlplgtvdlqhvleswyqlgppgttep<br>............................<br>............................<br>............................<br>............................<br>............................<br>............................	Hs_ENSP00000371394<br>Bt_ENSBTAP00000052023<br>Rn_ENSRNOP00000027454<br>Mm_ENSMUSP00000101598<br>Gg<br>Xt<br>Dr<br>Ce<br>Dm<br>Sc	NP_612634 Synaptotagmin-8 (Synaptotagmin VIII)(SytVIII)
256	1	CI	ENSP00000313600<br>ENSG00000118322	1079<br>1076<br>1077	gigisgqegmQAVMSSDFaitrfkhlkk<br>gigisgqegmQAVMSSDFaisrfrhlkk<br>gigisgqegmQAVMSSDFaiarfshlkk	Hs_ENSP00000313600<br>Bt_ENSBTAP00000022402<br>Rn_ENSRNOP00000030760	NP_079429 Probable phospholipid-transporting ATPase VB (EC 3.6.3.1)

				1077<br>1061<br>-<br>-<br>-<br>-<br>-	gigisgqegmQAVMSSDFaiarfshlkk<br>gigisgqegmQAVMASDFaisrfkhlkk<br>............................<br>............................<br>............................<br>............................<br>............................	Mm_ENSMUSP00000076844<br>Gg_ENSGALP00000002536<br>Xt<br>Dr<br>Ce<br>Dm<br>Sc	
257	1	CI	ENSP00000317743<br>ENSG00000180423	341<br>341<br>-<br>341<br>340<br>339<br>341<br>-<br>369<br>-	sldleadrirQELMLTHFs---------<br>sldleadrirQELMLTHFs---------<br>............................<br>sldleadrirQELILTHFs---------<br>smdseacrirQELLLTHFs---------<br>pleseayrmrQELILTHFs---------<br>tddpealrvrQELIQNHFs---------<br>............................<br>spksgddnnrrqfikthfac--------<br>............................	Hs_ENSP00000317743<br>Bt_ENSBTAP00000006085<br>Rn<br>Mm_ENSMUSP00000088098<br>Gg_ENSGALP00000013590<br>Xt_ENSXETP00000004235<br>Dr_ENSDARP00000052322<br>Ce<br>Dm_FBpp0079738<br>Sc	NP_776172 Putative nuclease HARBI1 (EC 3.1.-.-)(Harbinger transposase-derived nuclease)
258	1	C	ENSP00000365834<br>ENSG00000197969	1767<br>1709<br>1760<br>1760<br>1758<br>476<br>-<br>1797<br>1850<br>1788	nwsslinlhcQLELEVHYynemfgvwep<br>nwsslinlhcQLELEVHYynemfgvwep<br>nwrtlinlhcQLELEVHYfnemfgvwep<br>nwlslinlhchlelevhyynemfgvwep<br>nwstlinlhsQLELEVHYfnemfgvwep<br>nwstlinlycqlelevhcfnemfgvwep<br>............................<br>dwssamrvssgvsvqmsyynesvsvwep<br>nwsrsltahgsltlnmnyynqalaewep<br>dwstdfealasletyvnifnysrsswep	Hs_ENSP00000365834<br>Bt_ENSBTAP00000034095<br>Rn_ENSRNOP00000014800<br>Mm_ENSMUSP00000068716<br>Gg_ENSGALP00000024437<br>Xt_ENSXETP00000007804<br>Dr<br>Ce_CE13443<br>Dm_FBpp0087984<br>Sc_YLL040C	Vacuolar protein sorting-associated protein 13A (Chorein)(Chorea-acanthocytosis protein)
258	2	C	ENSP00000365834<br>ENSG00000197969	2763<br>2705<br>2755<br>2754<br>2744<br>1486<br>-<br>2802<br>2903<br>2742	taslvdqs--qvslyeyfhispiklhls<br>ivslvdss--qvslyeyfhispiklhls<br>vvssvdqs--qvnlfeyfhispiklhls<br>vvssvdqs--qvnlfeyfhispiklhls<br>svssmdts--qislyeyfhispiklhls<br>avsstdts--sisvyeyfhispiklhls<br>............................<br>etastyrsqrpksfyndlhispimmhls<br>affeehsleeqksfydnlhlgplkihvs<br>elktagdi-----yfeifhiqptvlhls	Hs_ENSP00000365834<br>Bt_ENSBTAP00000034095<br>Rn_ENSRNOP00000014800<br>Mm_ENSMUSP00000068716<br>Gg_ENSGALP00000024437<br>Xt_ENSXETP00000007804<br>Dr<br>Ce_CE13443<br>Dm_FBpp0087984<br>Sc_YLL040C	Vacuolar protein sorting-associated protein 13A (Chorein)(Chorea-acanthocytosis protein)
258	3	C	ENSP00000365834<br>ENSG00000197969	2842<br>2784<br>2834<br>2833<br>2823<br>1565<br>-<br>2880<br>2972<br>2816	nyqfhttsdlqsevirhyskqaikqmyv<br>nyqfhttaelqsevirhyskqaikqmyv<br>nyqfhttselqsevirhyskqaikqmyv<br>nyqfhttselqsevirhyskqaikqmyv<br>nyhfcttqqlqsavtkhyskqaikqmyv<br>qnqfyttqelqwevirhyskqaikqmyv<br>............................<br>kcvfyspeqlnseiishyakqfikqvyv<br>eyqffsqkqlineitshytgqalkqlyv<br>dnvrvplpilmdhierhyttqfvyqihk	Hs_ENSP00000365834<br>Bt_ENSBTAP00000034095<br>Rn_ENSRNOP00000014800<br>Mm_ENSMUSP00000068716<br>Gg_ENSGALP00000024437<br>Xt_ENSXETP00000007804<br>Dr<br>Ce_CE13443<br>Dm_FBpp0087984<br>Sc_YLL040C	Vacuolar protein sorting-associated protein 13A (Chorein)(Chorea-acanthocytosis protein)
259	1	CI	ENSP00000334393<br>ENSG00000186092	17<br>34<br>59<br>24<br>-<br>-<br>-<br>-<br>-<br>-	flglsdsqelqtflfmlffvfyggivfg<br>flglsnsqelQIFLFVFFfvfyvgivfg<br>figlsdsqelQIFLFVFFlvfyvgivfg<br>lmglsnsrelQIFLFAFFfvfyvgivfg<br>............................<br>............................<br>............................<br>............................<br>............................<br>............................	Hs_ENSP00000334393<br>Bt_ENSBTAP00000017630<br>Rn_ENSRNOP00000053486<br>Mm_ENSMUSP00000077188<br>Gg<br>Xt<br>Dr<br>Ce<br>Dm<br>Sc	NP_001004195 Olfactory receptor 4F4 (Olfactory receptor OR19-3)(HS14a-1-A)
259	2	CI	ENSP00000334393<br>ENSG00000186092	93<br>110<br>135<br>100<br>-<br>-<br>-<br>-<br>-<br>-	kvisfkgclvQIFLLHFFggsemvilia<br>kvisfkgclaQIFLLHFFggselmilia<br>kvisvhgcfaQIFLLHFFggcelvtlia<br>kvisvkgcftQIFLLHFFggsemvtlva<br>............................<br>............................<br>............................<br>............................<br>............................<br>............................	Hs_ENSP00000334393<br>Bt_ENSBTAP00000017630<br>Rn_ENSRNOP00000053486<br>Mm_ENSMUSP00000077188<br>Gg<br>Xt<br>Dr<br>Ce<br>Dm<br>Sc	NP_001004195 Olfactory receptor 4F4 (Olfactory receptor OR19-3)(HS14a-1-A)
260	1	CI	ENSP00000340658<br>ENSG00000183914	443<br>440<br>0	yhfarwedgkQGPLPCFFgaqgpqitrn<br>yhfarwedgkQGPLPCFFgaqgpqitrn<br>----------------------------	Hs_ENSP00000340658<br>Bt_ENSBTAP00000006277<br>Rn_ENSRNOP00000047546	NP_065928 Dynein heavy chain 2, axonemal (Axonemal beta dynein heavy chain 2)(Ciliary

				441<br>-<br>330<br>-<br>-<br>203<br>-	yhfarwedgkQGPLPCFFgaqgpqitrn<br>............................<br>eqfarwqdgkqsqlpcfsgaqgpavtrs<br>............................<br>............................<br>mvfgrldesesipkpqfggtsgtefeat<br>............................	Mm_ENSMUSP00000104299<br>Gg<br>Xt_ENSXETP00000018194<br>Dr<br>Ce<br>Dm_FBpp0289299<br>Sc	dynein heavy chain 2)(Dynein heavy chain domain-containing protein 3)
260	2	C	ENSP00000340658<br>ENSG00000183914	2822<br>2819<br>1620<br>2820<br>-<br>2682<br>-<br>-<br>2492<br>-	assicdyttfqievtkhyrkqefrddik<br>assiceyitfqievtkhyrrqefredik<br>assiceyntfqievtkhyrkqefrddik<br>assicdyntfqievtkhyrkqefrddik<br>............................<br>asyicdykvfqlevtrgyrkqefredik<br>............................<br>............................<br>aafilemavfqievtkkyktgdfredlk<br>............................	Hs_ENSP00000340658<br>Bt_ENSBTAP00000006277<br>Rn_ENSRNOP00000047546<br>Mm_ENSMUSP00000104299<br>Gg<br>Xt_ENSXETP00000018194<br>Dr<br>Ce<br>Dm_FBpp0289299<br>Sc	NP_065928 Dynein heavy chain 2, axonemal (Axonemal beta dynein heavy chain 2)(Ciliary dynein heavy chain 2)(Dynein heavy chain domain-containing protein 3)
260	3	C	ENSP00000340658<br>ENSG00000183914	2988<br>2985<br>1784<br>2986<br>-<br>2848<br>-<br>-<br>2675<br>-	tqenihrkvaqifvtmhwsvaqysqkml<br>tqenihkkvaqifvtmhwsvatysqkml<br>--tqihrkvaqifvtmhwsvaqysqkml<br>tqenihrkvaqifvtmhwsvaqysqkml<br>............................<br>aidgiqgkvarifvtmhrsvaefshrmk<br>............................<br>............................<br>teailqrdiayvfsvihssvakmsenmy<br>............................	Hs_ENSP00000340658<br>Bt_ENSBTAP00000006277<br>Rn_ENSRNOP00000047546<br>Mm_ENSMUSP00000104299<br>Gg<br>Xt_ENSXETP00000018194<br>Dr<br>Ce<br>Dm_FBpp0289299<br>Sc	NP_065928 Dynein heavy chain 2, axonemal (Axonemal beta dynein heavy chain 2)(Ciliary dynein heavy chain 2)(Dynein heavy chain domain-containing protein 3)
261	1	CI	ENSP00000334314<br>ENSG00000066629	547<br>520<br>-<br>544<br>498<br>528<br>497<br>-<br>788<br>-	ligttrnfvlQGTLSGDFtpitqghtde<br>ligttrnfvlQGTLSGDFtpitqghtde<br>............................<br>ligttrnfvlQGTLSGDFtpitqghtde<br>ligttrnfvlQGTLSGDFfpitqghtde<br>ligttrnfvlqgtlsgefntitqghtde<br>ligttknyvlqgslngefipitqghtde<br>............................<br>yvgttrnnilegslqrrftqvvfghgrq<br>............................	Hs_ENSP00000334314<br>Bt_ENSBTAP00000017944<br>Rn<br>Mm_ENSMUSP00000105488<br>Gg_ENSGALP00000018224<br>Xt_ENSXETP00000035919<br>Dr_ENSDARP00000062865<br>Ce<br>Dm_FBpp0288783<br>Sc	NP_001008707 Echinoderm microtubule-associated protein-like 1 (EMAP-1)(HuEMAP-1)
262	1	CI	ENSP00000361328<br>ENSG00000101844	41<br>11<br>-<br>11<br>-<br>14<br>11<br>19<br>-<br>-	mesvlskyedQITIFTDYleeypdtdel<br>mesvlskyenQITIFADYleefpdtdel<br>............................<br>mesvmskyenQILIFPDYleefpdtdel<br>............................<br>pvsdylkhenepileyldleelpdsdep<br>meavlakyenqinvf---ledlpdtdep<br>gvgivetsltfeppfcesferisidnfp<br>............................<br>............................	Hs_ENSP00000361328<br>Bt_ENSBTAP00000018239<br>Rn<br>Mm_ENSMUSP00000108595<br>Gg<br>Xt_ENSXETP00000028090<br>Dr_ENSDARP00000019179<br>Ce_CE42796<br>Dm<br>Sc	NP_443168 Cysteine protease ATG4A (EC 3.4.22.-)(Autophagy-related protein 4 homolog A)(hAPG4A)(Autophagin-2)(Autophagy-related cysteine endopeptidase 2)(AUT-like 2 cysteine endopeptidase)
263	1	CI	ENSP00000386137<br>ENSG00000221938	270<br>271<br>270<br>270<br>-<br>-<br>-<br>-<br>-<br>-	apksrhpeeqQKVLSLFYslfnpmlnpl<br>apksrhpeeqQKVLSLFYslfnpmlnpl<br>apksqhpeeqQKILSLFYslfnpmlnpl<br>apksqhpelqQKILSLFYslfnpmlnpl<br>............................<br>............................<br>............................<br>............................<br>............................<br>............................	Hs_ENSP00000386137<br>Bt_ENSBTAP00000050726<br>Rn_ENSRNOP00000007200<br>Mm_ENSMUSP00000093647<br>Gg<br>Xt<br>Dr<br>Ce<br>Dm<br>Sc	NP_001001659 Olfactory receptor 2A14 (Olfactory receptor OR7-12)(OST182)
264	1	CI	ENSP00000276497<br>ENSG00000147507	564<br>494<br>494<br>494<br>474<br>467<br>492<br>-<br>-<br>-	aeerptfdylQSVLDDFYtategqyqqq<br>aeerptfdylQSVLDDFYtategqyqqq<br>aeerptfdylQSVLDDFYtategqyqqq<br>aeerptfdylQSVLDDFYtategqyqqq<br>akerptfdylQSVLDDFYtategqyqqq<br>aeerptfdylQSVLDDFYtateaqyqqq<br>pedrptfdyiQSVLDDFYtategqyqqq<br>............................<br>............................<br>............................	Hs_ENSP00000276497<br>Bt_ENSBTAP00000026686<br>Rn_ENSRNOP00000011130<br>Mm_ENSMUSP00000038838<br>Gg_ENSGALP00000029288<br>Xt_ENSXETP00000012464<br>Dr_ENSDARP00000040554<br>Ce<br>Dm<br>Sc	NP_001104567 Tyrosine-protein kinase Lyn (EC 2.7.10.2)
265	1	CI	ENSP00000362013<br>ENSG00000124496	229<br>229<br>229	palqvgqhptQGHLYYDYqqplaqvpvq<br>palqvgqhpsQGHLYYDYqqplaqmpvq<br>palpvgqhapQGHLYYDYqqplaqmsvq	Hs_ENSP00000362013<br>Bt_ENSBTAP00000020376<br>Rn_ENSRNOP00000032697	NP_277037 Transcriptional-regulating factor 1 (Transcriptional-regulating

				229<br>222<br>219<br>-<br>140<br>258<br>-	palpvgqhapQGHLYYDYqqplaqmsmq<br>qqmqvmqhq---qlyydyqqhlsqmqih<br>hqmqamqhp-qqhiyyeypqhgsqmhsq<br>............................<br>kcslapnffssqnshhmypdqytprtwq<br>sfkgtgrhvpasglsssaastntngtvs<br>............................	Mm_ENSMUSP00000077103<br>Gg_ENSGALP00000016071<br>Xt_ENSXETP00000000414<br>Dr<br>Ce_CE32433<br>Dm_FBpp0081603<br>Sc	protein 132)(Zinc finger transcription factor TReP-132)(Zinc finger protein rapa)(Breast cancer anti-estrogen resistance 2)
266	1	CI	ENSP00000300283<br>ENSG00000166998	218<br>223<br>219<br>219<br>218<br>218<br>220<br>-<br>-<br>-	rlsemteaeqQQLIDDHFlfdkpvspll<br>rlsemteaeqQQLIDDHFlfdkpvspll<br>rlsemteaeqQQLIDDHFlfdkpvspll<br>rlsemteaeqQQLIDDHFlfdkpvspll<br>rlsemtekeqQQLIDDHFlfdkpvspll<br>sltqmtekqqQQLIDDHFlfdkpvspll<br>sltvmteqeqQQLIDDHFlfdkpvspll<br>............................<br>............................<br>............................	Hs_ENSP00000300283<br>Bt_ENSBTAP00000009871<br>Rn_ENSRNOP00000019890<br>Mm_ENSMUSP00000077349<br>Gg_ENSGALP00000013585<br>Xt_ENSXETP00000057514<br>Dr_ENSDARP00000011271<br>Ce<br>Dm<br>Sc	NP_066270 Creatine kinase, ubiquitous mitochondrial Precursor (EC 2.7.3.2)(U-MtCK)(Acidic-type mitochondrial creatine kinase)(Mia-CK)
267	1	CI	ENSP00000221265<br>ENSG00000006712	187<br>187<br>187<br>187<br>-<br>98<br>68<br>179<br>187<br>169	taiektfedaQKSISQHYskprvtpvev<br>taiektfedaQKSISQHYskprvtpvev<br>taiektfedaQKSISQHYskprvtpvev<br>taiektfedaQKSISQHYskprvtpvev<br>............................<br>saiektfedaQKPISQHYskprvtpvev<br>aaiektfedaQKSISQHYskprvtpvev<br>dainktfedvrkpvkehyskkgvkavee<br>kaiektfsdtkseitkhyskpnvvpvev<br>srvegtfnktdk--wqhpvkkgvkmvkk	Hs_ENSP00000221265<br>Bt_ENSBTAP00000027224<br>Rn_ENSRNOP00000026778<br>Mm_ENSMUSP00000003529<br>Gg<br>Xt_ENSXETP00000006351<br>Dr_ENSDARP00000064443<br>Ce_CE20614<br>Dm_FBpp0078508<br>Sc_YBR279W	NP_061961 RNA polymerase II-associated factor 1 homolog (hPAF1)(Pancreatic differentiation protein 2)
268	1	CI	ENSP00000342481<br>ENSG00000054793	809<br>824<br>-<br>867<br>808<br>807<br>810<br>-<br>-<br>-	gvgvegkegkQASLAADFsitqfkhlgr<br>gvgvegkegkQASLAADFsitqfrhlgr<br>............................<br>gvgvegkegkQASLAADFsitqfkhlgr<br>gvgvegkegkQASLAADFsitqfkhlgr<br>gvgvegkegkQASLAADFsvtqfkhlgr<br>gvgvegkegkQASLAADFsitqfkhlgr<br>............................<br>............................<br>............................	Hs_ENSP00000342481<br>Bt_ENSBTAP00000010474<br>Rn<br>Mm_ENSMUSP00000104805<br>Gg_ENSGALP00000012834<br>Xt_ENSXETP00000026956<br>Dr_ENSDARP00000081097<br>Ce<br>Dm<br>Sc	NP_006036 Probable phospholipid-transporting ATPase IIA (EC 3.6.3.1)(ATPase class II type 9A)(ATPase IIA)
269	1	CI	ENSP00000339109<br>ENSG00000153107	124<br>124<br>0<br>124<br>124<br>124<br>-<br>0<br>165<br>-	ykaftvdspvQQALWCDFiisqdkseka<br>ykaftvdspvQQALWCDFiisqdksdkd<br>----------------------------<br>ykaftvdstvQQALWCDFiisqdkseki<br>ykafsvdspvlqalwcdftiqqeksdka<br>ykaftvdspvvqalwcdftifndkndne<br>............................<br>----------------------------<br>rmcfttdtpvrfacflnrsfvrgrlaql<br>............................	Hs_ENSP00000339109<br>Bt_ENSBTAP00000040146<br>Rn_ENSRNOP00000052727<br>Mm_ENSMUSP00000014499<br>Gg_ENSGALP00000013420<br>Xt_ENSXETP00000001735<br>Dr<br>Ce_CE43328<br>Dm_FBpp0073893<br>Sc	NP_073153 Anaphase-promoting complex subunit 1 (APC1)(Cyclosome subunit 1)(Testis-specific gene 24 protein)(Mitotic checkpoint regulator)
270	1	CI	ENSP00000300209<br>ENSG00000123427	193<br>193<br>-<br>193<br>-<br>194<br>-<br>-<br>-<br>-	rkehgtesffQHLLPQHFqlelaqrded<br>reehgtesffQHLLPQHFqlelaqrded<br>............................<br>raehgtaetffrrllpqhfhlelaqrded<br>............................<br>rqehgtmhffqdilpqyfaselvkrnkd<br>............................<br>............................<br>............................<br>............................	Hs_ENSP00000300209<br>Bt_ENSBTAP00000022493<br>Rn<br>Mm_ENSMUSP00000111939<br>Gg<br>Xt_ENSXETP00000021254<br>Dr<br>Ce<br>Dm<br>Sc	NP_056248 Protein FAM119B (Hepatocellular carcinoma-associated antigen 557a)
271	1	CI	ENSP00000219476<br>ENSG00000103197	446<br>446<br>446<br>446<br>446<br>446<br>-<br>-<br>448<br>-	pakdgwiqnlQALMERFFrsesrgavri<br>pakdgwihnlQLLMERFFrnesrsavri<br>pakdgwiqnlQLLMERFFrnecrsavri<br>pakdgwiqnlQLLMERFFrnecrsavri<br>pakdgwihnlQMLMERFFrnesrsavri<br>pgkegwimnlqklmdryfrsesrsvvrm<br>............................<br>............................<br>atrpdwlqvlaqfvrryyr-msnvnvri<br>............................	Hs_ENSP00000219476<br>Bt_ENSBTAP00000046373<br>Rn_ENSRNOP00000016221<br>Mm_ENSMUSP00000085764<br>Gg_ENSGALP00000009042<br>Xt_ENSXETP00000034777<br>Dr<br>Ce<br>Dm_FBpp0074588<br>Sc	NP_000539 Tuberin (Tuberous sclerosis 2 protein)
272	1	C	ENSP00000363520<br>ENSG00000001461	232<br>232<br>235	avagmlvlsiQGNLQLDYpifyvmfvcm<br>avagmlvlsiQGNLQLDYpifyvmfvcm<br>fisalsfysqn---------fanmhlil	Hs_ENSP00000363520<br>Bt_ENSBTAP00000017344<br>Rn_ENSRNOP00000025075	NP_065181 NIPA-like protein 3

				236	avsgmlvlsiQGNLQLDYpifyvmfvcm	Mm_ENSMUSP00000101482	
				234	avagmivvsirgnlqlnypifyimlvcm	Gg_ENSGALP00000006776	
				225	avagmiivsiqgsmqlgypifyvmfvcm	Xt_ENSXETP00000008479	
				219	avsgmivlsivgplqlsypifyvmfvcm	Dr_ENSDARP00000013139	
				-	....	Ce	
				-	....	Dm	
				-	....	Sc	
273	1	CI	ENSP00000377699 ENSG00000156920	2623	lteriplsnlQTILFNFFgqtslfktkn	Hs_ENSP00000377699	NP_722576 Probable G-protein coupled receptor 112
				-	....	Bt	
				-	....	Rn	
				2574	lrekvpldglQTILFNFFgqtslfkakt	Mm_ENSMUSP00000110410	
				-	....	Gg	
				-	....	Xt	
				-	....	Dr	
				-	....	Ce	
				-	....	Dm	
				-	....	Sc	
274	1	C	ENSP00000367664 ENSG00000221840	98	ktisfqgcmgQLFIDHFFggaevfllvv	Hs_ENSP00000367664	NP_001005272 Olfactory receptor 4A5 (Olfactory receptor OR11-111)
				-	....	Bt	
				98	ktisftaclvqlfvehlfggaevfllvv	Rn_ENSRNOP00000050135	
				-	....	Mm	
				-	....	Gg	
				-	....	Xt	
				-	....	Dr	
				-	....	Ce	
				-	....	Dm	
				-	....	Sc	
275	1	CI	ENSP00000288235 ENSG00000157483	500	gshehfnswnQGFIIHHYagkvsydmdg	Hs_ENSP00000288235	NP_004989 Myosin-Ie (Myosin-Ic)
				500	gnhehfnswnQGFIIHHYagkvsydmdg	Bt_ENSBTAP00000028702	
				501	gshehfnswnQGFIIHHYagkvsydmdg	Rn_ENSRNOP00000016696	
				500	gshehfnswnQGFIIHHYagkvsydmdg	Mm_ENSMUSP00000034745	
				489	gthehfnswnQGFIIHHYagkvsydmdg	Gg_ENSGALP00000038539	
				504	gthehfnswnQGFIIHHYagkvsydvdg	Xt_ENSXETP00000035798	
				500	nnhehfnswnQGFIIHHYagkvsydaeg	Dr_ENSDARP00000052538	
				498	aghphfgpgsdsfvikhyagdvtynvdg	Ce_CE05763	
				-	....	Dm	
				519	-tnphfdlrsnkfvikhyagdvtydidg	Sc_YMR109W	
276	1	CI	ENSP00000305480 ENSG00000168496	337	rlksksrqgstQGRLDDFFk----vtgsl	Hs_ENSP00000305480	NP_004102 Flap endonuclease 1 (FEN-1)(EC 3.1.-.-)(Flap structure-specific endonuclease 1)(Maturation factor 1)(MF1) (hFEN-1)(DNase IV)
				337	rlksksrqgstQGRLDDFFk----vtgsl	Bt_ENSBTAP00000000071	
				337	rlnksrqgstQGRLDDFFk----vtgsl	Rn_ENSRNOP00000027842	
				337	rlksksrqgstQGRLDDFFk----vtgsl	Mm_ENSMUSP00000025651	
				337	rlksksrqgstQGRLDDFFk----vtgsi	Gg_ENSGALP00000005724	
				337	klsknrqgstQGRLDDFFk----vtgsi	Xt_ENSXETP00000014663	
				337	kitksrqgstQGRLDTFFt----vtgsi	Dr_ENSDARP00000004016	
				337	klktsrksgtQGRIDSFFgnstkvtcvt	Ce_CE22109	
				337	klmkskqaqtQVRLDSFFktlpstpnat	Dm_FBpp0086223	
				340	rlkkglksgiQGRLDGFFqvvpktkeql	Sc_YKL113C	
277	1	CI	ENSP00000284509 ENSG00000104043	839	gvgisgqeglQAVLASDYsfaqfrylqr	Hs_ENSP00000284509	NP_079113 Probable phospholipid-transporting ATPase IM (EC 3.6.3.1)(ATPase class I type 8B member 4)
				-	....	Bt	
				831	gigisgqeglQAVLASDYalaqfrylqr	Rn_ENSRNOP00000052828	
				841	gigisgqeglQAVLASDYalaqfrylqr	Mm_ENSMUSP00000046444	
				-	....	Gg	
				852	gvgisgqegmQAVLASDYsfaqfrylqr	Xt_ENSXETP00000019309	
				-	....	Dr	
				-	....	Ce	
				-	....	Dm	
				-	....	Sc	
278	1	CI	ENSP00000291901 ENSG00000105048	79	krmekdllelQTLIDVHFeqrkkeeeel	Hs_ENSP00000291901	NP_003274 Troponin T, slow skeletal muscle (TnTs)(Slow skeletal muscle troponin T) (sTnT)
				83	krmekdllelQTLIDVHFeqrkkeeeel	Bt_ENSBTAP00000008420	
				78	krmekdllelQTLIDVHFeqrkkeeeel	Rn_ENSRNOP00000038327	
				79	krmekdllelQTLIDVHFeqrkkeeeel	Mm_ENSMUSP00000071704	
				-	....	Gg	
				82	krmekdllelQTLIDVHFeqrkkeeeel	Xt_ENSXETP00000006951	
				34	krmekdllelQTLIEAHFeqrkkeeeel	Dr_ENSDARP00000094072	
				-	....	Ce	
				-	....	Dm	
				-	....	Sc	
279	1	CI	ENSP00000326194 ENSG00000128923	509	sdpetvykgqQDQIDQDYlmalslqqeq	Hs_ENSP00000326194	NP_001035540 Protein FAM63B
				510	sdpetvyrgqQDQIDQDYlmalslqqeq	Bt_ENSBTAP00000016444	
				485	sdpetvyxgqQDQIDQDYlmalslqqeq	Rn_ENSRNOP00000020088	

				487<br>238<br>354<br>332<br>309<br>-<br>277	sdpetvykgqQDQIDQDYlmalslqqeq<br>sdpetvyrgqQDQIDQDYlmalslqqeq<br>sdpetvyqgqedqinqdylmalslqqeq<br>sdpetvyrgqQDQIDQDYlmalslqqeq<br>itpptthps-------------------<br>............................<br>vngqqlstdier----------------	Mm_ENSMUSP00000037035<br>Gg_ENSGALP00000033819<br>Xt_ENSXETP00000023734<br>Dr_ENSDARP00000004845<br>Ce_CE25491<br>Dm<br>Sc_YPL191C	
280	1	CI	ENSP00000353129<br>ENSG00000183783	117<br>-<br>117<br>117<br>80<br>70<br>99<br>-<br>-<br>-	ryvldylrdkQLALPEHFpekerllrea<br>............................<br>ryvldylrdkQLALPEHFpekerllrea<br>ryvldylrdkQLALPEHFpekerllrea<br>ryvldylrdkQLALPEHFpekerllrea<br>ryildflrdkQLSLPDHFpekerllrea<br>ryvldflrdrQLVLPEHFpererlqrea<br>............................<br>............................<br>............................	Hs_ENSP00000353129<br>Bt<br>Rn_ENSRNOP00000030153<br>Mm_ENSMUSP00000055326<br>Gg_ENSGALP00000022959<br>Xt_ENSXETP00000018480<br>Dr_ENSDARP00000097460<br>Ce<br>Dm<br>Sc	NP_938167 BTB/POZ domain-containing protein KCTD8
281	1	CI	ENSP00000378757<br>ENSG00000165449	158<br>158<br>-<br>158<br>158<br>158<br>161<br>-<br>-<br>-	svglfiyaalQRMLVEFYgldgcllivg<br>svglfiyaalQRLLIEFYgldgcllivg<br>............................<br>svglfiyaalQRMLIEFYgldgcllivg<br>svglfiyaalqrelielygldgcllivg<br>sigtfvyaslqkvliaiygldgclliig<br>svggfiyatlqnelielfglegclliig<br>............................<br>............................<br>............................	Hs_ENSP00000378757<br>Bt_ENSBTAP00000026369<br>Rn<br>Mm_ENSMUSP00000047912<br>Gg_ENSGALP00000005024<br>Xt_ENSXETP00000011138<br>Dr_ENSDARP00000003362<br>Ce<br>Dm<br>Sc	NP_919274 Monocarboxylate transporter 9 (MCT 9)(Solute carrier family 16 member 9)
282	1	CI	ENSP00000264568<br>ENSG00000138696	274<br>280<br>274<br>274<br>307<br>280<br>-<br>336<br>336<br>-	dikgt-gswtQLYLITDYhengslydyl<br>dikgt-gswtQLYLITDYhengslydyl<br>dikgt-gswtQLYLITDYhengslydyl<br>dikgt-gswtQLYLITDYhengslydyl<br>dikgt-gswtQLYLITDYhengslydyl<br>dikgt-gswtQLYLITDYhengslydyl<br>............................<br>diwseedsmtkmllitdyhelgslsdyl<br>dikgn-gswtQMLLITDYhemgslhdyl<br>............................	Hs_ENSP00000264568<br>Bt_ENSBTAP00000002690<br>Rn_ENSRNOP00000021963<br>Mm_ENSMUSP00000029948<br>Gg_ENSGALP00000019925<br>Xt_ENSXETP00000041649<br>Dr<br>Ce_CE01842<br>Dm_FBpp0078721<br>Sc	NP_001194 Bone morphogenetic protein receptor type-1B Precursor (EC 2.7.11.30)(CDw293 antigen)
283	1	CI	ENSP00000364664<br>ENSG00000144290	1059<br>1068<br>1051<br>1068<br>1036<br>-<br>-<br>-<br>-<br>-	mlamedegtvQLPLEGHYrddpsvinis<br>mlamedegtvQLPLEGHYrddpsvinis<br>mlamedegtvQLPLEGHYrddpsvinis<br>mlamedegtvQLPLEGHYrddpsvinis<br>mlameeegtvQLPLEGHYrddpsvinis<br>............................<br>............................<br>............................<br>............................<br>............................	Hs_ENSP00000364664<br>Bt_ENSBTAP00000020366<br>Rn_ENSRNOP00000007218<br>Mm_ENSMUSP00000108099<br>Gg_ENSGALP00000018095<br>Xt<br>Dr<br>Ce<br>Dm<br>Sc	Sodium-driven chloride bicarbonate exchanger (Solute carrier family 4 member 10)
284	1	CI	ENSP00000358770<br>ENSG00000065613	1218<br>1223<br>1290<br>1216<br>1196<br>1168<br>-<br>-<br>-<br>-	geseclnpstQSRISKFYpipslhstgs<br>geseclnpstQSRISKFYpipslhstgs<br>geseclnpsaQSRISKFYpiptlhstgs<br>geseclnpsaQSRISKFYpiptlhstgs<br>geseclnpsaQSRISKFYpipslhstgs<br>----------------------------<br>............................<br>............................<br>............................<br>............................	Hs_ENSP00000358770<br>Bt_ENSBTAP00000001346<br>Rn_ENSRNOP00000015496<br>Mm_ENSMUSP00000049977<br>Gg_ENSGALP00000013524<br>Xt_ENSXETP00000002688<br>Dr<br>Ce<br>Dm<br>Sc	NP_055535 STE20-like serine/threonine-protein kinase (STE20-like kinase)(hSLK)(EC 2.7.11.1)(STE20-related serine/threonine-protein kinase)(STE20-related kinase)(Serine/threonine-protein kinase 2)(CTCL tumor antigen se20-9)
285	1	C	ENSP00000285238<br>ENSG00000108846	436<br>444<br>435<br>435<br>445<br>446<br>-<br>-<br>-<br>-	flnllwsaplqiilaiyflwqnlgpsvl<br>finllwsaplqiilavyflwqnlgpsvl<br>finllwsaplqvilaiyflwqilgpsal<br>finllwsaplqvilaiyflwqilgpsal<br>flnmlwsaplqtclalyflwqalgpsvl<br>flnmlwsaplqiclalyflwqalgpsvl<br>............................<br>............................<br>............................<br>............................	Hs_ENSP00000285238<br>Bt_ENSBTAP00000026744<br>Rn_ENSRNOP00000003977<br>Mm_ENSMUSP00000103436<br>Gg_ENSGALP00000012150<br>Xt_ENSXETP00000026773<br>Dr<br>Ce<br>Dm<br>Sc	NP_003777 Canalicular multispecific organic anion transporter 2 (ATP-binding cassette sub-family C member 3)(Multidrug resistance-associated protein 3)(Multi-specific organic anion transporter D)(MOAT-D)
286	1	CI	ENSP00000223095<br>ENSG00000106366	130<br>130<br>130	ifvqrdlklvQGFMPHFFrlfrstvkqv<br>ifvqrdlelvhgfmpnffrlfrttvkqv<br>ifvqrdlelvQGFMPHFFklfrttvkqv	Hs_ENSP00000223095<br>Bt_ENSBTAP00000019232<br>Rn_ENSRNOP00000001916	NP_000593 Plasminogen activator inhibitor 1 Precursor

				130<br>-<br>-<br>123<br>-<br>-<br>-	ifvqrdlelvQGFMPHFFklfqtmvkqv<br>............................<br>............................<br>vmvdrkiilekvfrrslskafqsvphqi<br>............................<br>............................<br>............................	Mm_ENSMUSP00000039586<br>Gg<br>Xt<br>Dr_ENSDARP00000073803<br>Ce<br>Dm<br>Sc	(PAI-1)(PAI)(Endothelial plasminogen activator inhibitor)
287	1	CI	ENSP00000333926<br>ENSG00000114770	995<br>788<br>994<br>994<br>993<br>-<br>595<br>918<br>-<br>-	rlpfqaemfiQNVILVFFcvgmiagvfp<br>rlpfqaemfiQNVILVFFcvgmiagvfp<br>rlpfqaemfiQNVILVFFcvgmiagvfp<br>rlpfqaemfiQNVILVFFcvgmiagvfp<br>rlpfqaemfiQNVILVFFcvgvisgvfp<br>............................<br>rlpfqaemfiqnvilvlfclavigsvfp<br>klpftaevflqnmitclgflvvitsvfp<br>............................<br>............................	Hs_ENSP00000333926<br>Bt_ENSBTAP00000023496<br>Rn_ENSRNOP00000002316<br>Mm_ENSMUSP00000078158<br>Gg_ENSGALP00000013610<br>Xt<br>Dr_ENSDARP00000081504<br>Ce_CE23650<br>Dm<br>Sc	NP_005679 Multidrug resistance-associated protein 5 (ATP-binding cassette sub-family C member 5)(Multi-specific organic anion transporter C)(MOAT-C)(pABC11)(SMRP)
288	1	CI	ENSP00000379855<br>ENSG00000145675	241<br>239<br>241<br>241<br>241<br>240<br>241<br>151<br>-<br>-	siphqywltlQYLLKHFFklsqtssknl<br>siphqywltlQYLLKHFFklsqtssknl<br>niphqywltlQYLLKHFFklsqassknl<br>niphqcwltlQYLLKHFFklsqassknl<br>niphqywltlqyllkhflrlcqassknl<br>nvpqqywltlqyllkhflqlcqsssknl<br>alppqywltlqsllrhfshvcqssavnl<br>kndhdwrerlemenlravhlafergakl<br>............................<br>............................	Hs_ENSP00000379855<br>Bt_ENSBTAP00000014594<br>Rn_ENSRNOP00000025687<br>Mm_ENSMUSP00000056774<br>Gg_ENSGALP00000023822<br>Xt_ENSXETP00000046442<br>Dr_ENSDARP00000056212<br>Ce_CE23248<br>Dm<br>Sc	Phosphatidylinositol 3-kinase regulatory subunit alpha (PI3-kinase p85 subunit alpha)(PtdIns-3-kinase p85-alpha)(PI3K)
289	1	CI	ENSP00000286332<br>ENSG00000055208	686<br>686<br>686<br>686<br>690<br>683<br>704<br>-<br>785<br>-	lnhpalirceQCEMPRHF----------<br>lnhpalirceqcemprhl----------<br>lnhpalirceQCEMPRHF----------<br>lnhpalirceQCEMPRHF----------<br>lnhpalnrceQCEMPRHF----------<br>lnhpalnrceQCDMPRHF----------<br>lnhpalnrceecefprnf----------<br>............................<br>rnhpqlniceacenvriqpgmirivpsg<br>............................	Hs_ENSP00000286332<br>Bt_ENSBTAP00000017897<br>Rn_ENSRNOP00000021904<br>Mm_ENSMUSP00000015899<br>Gg_ENSGALP00000020155<br>Xt_ENSXETP00000046262<br>Dr_ENSDARP00000019647<br>Ce<br>Dm_FBpp0085704<br>Sc	NP_055908 Mitogen-activated protein kinase kinase kinase 7-interacting protein 2 (TAK1-binding protein 2)
290	1	CI	ENSP00000259362<br>ENSG00000136839	6<br>-<br>6<br>-<br>-<br>-<br>-<br>-<br>-<br>-	-----mewenQTILVEFFlkghsvhprl<br>............................<br>-----mewenQTYLEEFFlkglsgypgl<br>............................<br>............................<br>............................<br>............................<br>............................<br>............................<br>............................	Hs_ENSP00000259362<br>Bt<br>Rn_ENSRNOP00000024724<br>Mm<br>Gg<br>Xt<br>Dr<br>Ce<br>Dm<br>Sc	NP_001001956 Olfactory receptor 13C9
291	1	CI	ENSP00000255263<br>ENSG00000132912	247<br>247<br>254<br>254<br>247<br>-<br>255<br>211<br>259<br>-	vnltevttlqQRLLQPDFqpvcasqlyp<br>vnltevttlqQRLLQPDFqpvcasqlyp<br>vnltevttlqqrllqpdlqpvsasqlyp<br>vnltevttlqqrllqpdlqpvsasqlyp<br>inltevttlhQRLLQPDFqpicasqlyp<br>............................<br>vnlpevttlrQRLLQPDFqpagasqlhp<br>--tddlrtldqiimqplin--isepllp<br>lnlrnvttitqrhsqpadqptavgslyp<br>............................	Hs_ENSP00000255263<br>Bt_ENSBTAP00000020755<br>Rn_ENSRNOP00000026394<br>Mm_ENSMUSP00000110981<br>Gg_ENSGALP00000007207<br>Xt<br>Dr_ENSDARP00000034920<br>Ce_CE04087<br>Dm_FBpp0088018<br>Sc	NP_057305 Dynactin subunit 4 (Dynactin subunit p62)
292	1	CI	ENSP00000317636<br>ENSG00000108469	964<br>960<br>946<br>955<br>996<br>-<br>-<br>809<br>1031<br>-	pgr-svkeeaQNLIRHFFhgrarcesea<br>pgr-svkeeaqdvikqlfhgrarcesea<br>pgr-svkeeaQSLIKQFFhnrarcesea<br>pgr-svkeeaQSLIKQFFhnrarcesea<br>parktvkeeaQRLIKEFFktrvrceset<br>............................<br>............................<br>----------------------------<br>gdg----dagkcyiketfhglqminndq<br>............................	Hs_ENSP00000317636<br>Bt_ENSBTAP00000015555<br>Rn_ENSRNOP00000007246<br>Mm_ENSMUSP00000021097<br>Gg_ENSGALP00000003806<br>Xt<br>Dr<br>Ce_CE00941<br>Dm_FBpp0075458<br>Sc	NP_004250 ATP-dependent DNA helicase Q5 (EC 3.6.1.-)(RecQ protein-like 5)(RecQ5)
293	1	C	ENSP00000359018<br>ENSG00000162641	25<br>26<br>26	edlpydgdlsQIKIGNDYsftskkdgle<br>edlpydegfsqvklyhgynltsrydild<br>edlpydgdfsqmkictsynftltndtfp	Hs_ENSP00000359018<br>Bt_ENSBTAP00000009644<br>Rn_ENSRNOP00000052712	NP_689976 Uncharacterized protein C1orf62

				-<br>-<br>23<br>-<br>-<br>-<br>-	............................<br>............................<br>edlpydgilecylmpnvnqfdisn----<br>............................<br>............................<br>............................<br>............................	Mm<br>Gg<br>Xt_ENSXETP00000011434<br>Dr<br>Ce<br>Dm<br>Sc	
294	1	C	ENSP00000362762<br>ENSG00000121753	86<br>74<br>74<br>74<br>-<br>-<br>83<br>-<br>-<br>-	kyslylrfnrqeqvcahfaprllpldhy<br>kyslylrfnrqe--------------hs<br>kyslylrfnrqeqvcthfaprllpldhy<br>kyslylrfnrqeqvcthfaprllpldhy<br>............................<br>............................<br>kyslyfrfnrhagaclafsplvlpldhy<br>............................<br>............................<br>............................	Hs_ENSP00000362762<br>Bt_ENSBTAP00000037830<br>Rn_ENSRNOP00000019547<br>Mm_ENSMUSP00000101636<br>Gg<br>Xt<br>Dr_ENSDARP00000079452<br>Ce<br>Dm<br>Sc	NP_001694 Brain-specific angiogenesis inhibitor 2 Precursor
294	2	CI	ENSP00000362762<br>ENSG00000121753	1236<br>1177<br>1231<br>1230<br>-<br>-<br>1213<br>-<br>-<br>-	edspdsckngQLQILSDFekdvdlacqt<br>edspdsckngQLQILSDFekdvdlacqt<br>edspdsckngQLQILSDFekdvdlacqt<br>edspdsckngQLQILSDFekdvdlacqt<br>............................<br>............................<br>enspdsckngQVQIMTDFekdvdlacqt<br>............................<br>............................<br>............................	Hs_ENSP00000362762<br>Bt_ENSBTAP00000037830<br>Rn_ENSRNOP00000019547<br>Mm_ENSMUSP00000101636<br>Gg<br>Xt<br>Dr_ENSDARP00000079452<br>Ce<br>Dm<br>Sc	NP_001694 Brain-specific angiogenesis inhibitor 2 Precursor
295	1	CI	ENSP00000320939<br>ENSG00000204330	206<br>293<br>293<br>-<br>-<br>-<br>144<br>-<br>-<br>-	hitnatseerQKMLRDFYasqypevkef<br>rvtnatseerQKMLRDFYtsqypevref<br>qvtsatseeqQRMLRDFYslqhpevkef<br>............................<br>............................<br>............................<br>eilksdsdqrlgrlrsfysqkspelmni<br>............................<br>............................<br>............................	Hs_ENSP00000320939<br>Bt_ENSBTAP00000027488<br>Rn_ENSRNOP00000025914<br>Mm<br>Gg<br>Xt<br>Dr_ENSDARP00000089485<br>Ce<br>Dm<br>Sc	Uncharacterized protein C9orf102
296	1	CI	ENSP00000362768<br>ENSG00000080839	842<br>842<br>841<br>841<br>831<br>770<br>-<br>-<br>636<br>-	pdlmkdrhldQLLLCAFYimakvt-kee<br>pdlmkdrhldQLLLCAFYimakvt-kee<br>pdlmkdrhldQLLLCAFYimakvt-kee<br>pdlmkdrhldQLLLCAFYimakvt-kee<br>adlmkdrhldQLLLCAFYimakvt-kee<br>tdlmkdrhldqlllcavyimakis-kee<br>............................<br>............................<br>telmkdrhldqnimcaiyiyirvkrmed<br>............................	Hs_ENSP00000362768<br>Bt_ENSBTAP00000015333<br>Rn_ENSRNOP00000009528<br>Mm_ENSMUSP00000029170<br>Gg_ENSGALP00000002030<br>Xt_ENSXETP00000016130<br>Dr<br>Ce<br>Dm_FBpp0070141<br>Sc	NP_002886 Retinoblastoma-like protein 1 (PRB1)(107 kDa retinoblastoma-associated protein)(p107)
297	1	C	ENSP00000298110<br>ENSG00000165370	466<br>371<br>462<br>465<br>-<br>290<br>309<br>-<br>-<br>-	ymhktikkeiQDMLKKFFckekppkeds<br>ymhktikkeiQDMLKKFFckekpp----<br>ymhksikkeikevlkkltckkstsvdds<br>ymhksikkeiqevlkklickksppveds<br>............................<br>----------------------------<br>ymhrsvrkeflallc-------------<br>............................<br>............................<br>............................	Hs_ENSP00000298110<br>Bt_ENSBTAP00000048444<br>Rn_ENSRNOP00000029809<br>Mm_ENSMUSP00000058183<br>Gg<br>Xt_ENSXETP00000007523<br>Dr_ENSDARP00000057295<br>Ce<br>Dm<br>Sc	NP_473362 Probable G-protein coupled receptor 101
298	1	CI	ENSP00000225296<br>ENSG00000005100	255<br>-<br>246<br>246<br>205<br>202<br>228<br>-<br>248<br>-	gapvlylegrQHPIQVFYtkqpqndylh<br>............................<br>gapvlylegrQHPIQIFYtkqpqqdylh<br>rapvlylegrQHPIQIFYtkqpqqdylh<br>gapvlylegrQHPIQVFYtkqpqsdylq<br>gapvlylegrQHPIQIFYtkesqsdylq<br>kspvlylegrqhpiqiyytkqpqsdylq<br>............................<br>-ckgmylegrtypvrvmhtkeehedyih<br>............................	Hs_ENSP00000225296<br>Bt<br>Rn_ENSRNOP00000009468<br>Mm_ENSMUSP00000038018<br>Gg_ENSGALP00000002489<br>Xt_ENSXETP00000020150<br>Dr_ENSDARP00000008078<br>Ce<br>Dm_FBpp0079537<br>Sc	NP_064547 Putative ATP-dependent RNA helicase DHX33 (EC 3.6.1.-)(DEAH box protein 33)
299	1	C	ENSP00000219320<br>ENSG00000103042	3<br>4<br>3	--------maQVSINNDYsewdlstdag<br>-------amaqvsinrdlgewglstdsg<br>--------maQVSINSDYsewgsstdag	Hs_ENSP00000219320<br>Bt_ENSBTAP00000009438<br>Rn_ENSRNOP00000058216	NP_060701 Putative sodium-coupled neutral amino acid transporter 7

				3<br>3<br>5<br>5<br>-<br>-<br>-	--------maQVSINSDYsewasstdag<br>--------qaagsintdyrdwewsadag<br>------mslgnvginady-----swdag<br>------msrpdrainseagdwgysedag<br>............................<br>............................<br>............................	Mm_ENSMUSP00000037023<br>Gg_ENSGALP00000003630<br>Xt_ENSXETP00000034680<br>Dr_ENSDARP00000026295<br>Ce<br>Dm<br>Sc	
300	1	CI	ENSP00000377137<br>ENSG00000128573	157<br>48<br>76<br>157<br>48<br>42<br>132<br>-<br>-<br>-	lqqqqavmlqQQQLQEFYkkqqeqlhlq<br>lqqqqavmlqQQQLQEFYkkqqeqlesk<br>lqqqqavmlqQQQLQEFYkkqqeqlhlq<br>lqqqqavmlqQQQLQEFYkkqqeqlhlq<br>lqqqqavmlqQQQLQEFYkkqqeqlhlq<br>lqqqqavmlqqq----------------<br>lqqqqavmlqQQHLQEFYk---------<br>............................<br>............................<br>............................	Hs_ENSP00000377137<br>Bt_ENSBTAP00000014933<br>Rn_ENSRNOP00000057434<br>Mm_ENSMUSP00000111134<br>Gg_ENSGALP00000012590<br>Xt_ENSXETP00000025917<br>Dr_ENSDARP00000060860<br>Ce<br>Dm<br>Sc	NP_683697 Forkhead box protein P2 (CAG repeat protein 44)(Trinucleotide repeat-containing gene 10 protein)
301	1	CI	ENSP00000284885<br>ENSG00000154646	75<br>75<br>76<br>75<br>-<br>-<br>-<br>-<br>63<br>-	tsgvtynpnlQDKLSVDFkvlafdlqqm<br>isgatynphlQDKLSVDFkvlafdiqqm<br>tsgvtynpnlqdkfsvdfkvlafdlqqm<br>tsgvtynpnlqdkhsvdfkvlafdlqqm<br>............................<br>............................<br>............................<br>............................<br>idgrrvhr--------------------<br>............................	Hs_ENSP00000284885<br>Bt_ENSBTAP00000000788<br>Rn_ENSRNOP00000042615<br>Mm_ENSMUSP00000023566<br>Gg<br>Xt<br>Dr<br>Ce<br>Dm_FBpp0112081<br>Sc	NP_002763 Enteropeptidase Precursor (EC 3.4.21.9) (Enterokinase)(Serine protease 7)
301	2	C	ENSP00000284885<br>ENSG00000154646	861<br>880<br>911<br>910<br>-<br>-<br>-<br>-<br>579<br>-	spqtvprlideivinphynrrrkdndia<br>spqietrlidQIVINPHYnkrrknndia<br>spqvvrrvvdrivinphydkrrkvndia<br>spqvvrrvvdQIVINPHYdrrrkvndia<br>............................<br>............................<br>............................<br>............................<br>gteiqlrvmksyth-pnfdkrtvdsdva<br>............................	Hs_ENSP00000284885<br>Bt_ENSBTAP00000000788<br>Rn_ENSRNOP00000042615<br>Mm_ENSMUSP00000023566<br>Gg<br>Xt<br>Dr<br>Ce<br>Dm_FBpp0112081<br>Sc	NP_002763 Enteropeptidase Precursor (EC 3.4.21.9) (Enterokinase)(Serine protease 7)
302	1	CI	ENSP00000336850<br>ENSG00000175582	43<br>-<br>43<br>43<br>20<br>43<br>43<br>40<br>42<br>-	fmydsfdntyQATIGIDFlsktmyledr<br>............................<br>fmydsfdntyQATIGIDFlsktmyledr<br>fmydsfdntyQATIGIDFlsktmyledr<br>fmydsfdntyQATIGIDFlsktmyledr<br>fmydsfdntyQATIGIDFlsktmyledr<br>fmydsfdntyQATIGIDFlsktmyledr<br>fmydsfdntyQATIGIDFlsktmyledr<br>fmydsfdntyQATIGIDFlsktmyledr<br>............................	Hs_ENSP00000336850<br>Bt<br>Rn_ENSRNOP00000043537<br>Mm_ENSMUSP00000095852<br>Gg_ENSGALP00000027937<br>Xt_ENSXETP00000050153<br>Dr_ENSDARP00000005417<br>Ce_CE07541<br>Dm_FBpp0079943<br>Sc	NP_942599 Ras-related protein Rab-6A (Rab-6)
303	1	CI	ENSP00000267008<br>ENSG00000139567	272<br>272<br>273<br>271<br>-<br>-<br>-<br>-<br>-<br>-	sdmtsrnsstQLWLITHYhehgslydfl<br>sdmtsrnsstQLWLITHYhehgslydfl<br>sdmtsrnsstQLWLITHYhehgslydfl<br>sdmtsrnsstQLWLITHYhehgslydfl<br>............................<br>............................<br>............................<br>............................<br>............................<br>............................	Hs_ENSP00000267008<br>Bt_ENSBTAP00000018383<br>Rn_ENSRNOP00000008673<br>Mm_ENSMUSP00000000542<br>Gg<br>Xt<br>Dr<br>Ce<br>Dm<br>Sc	NP_001070869 Serine/threonine-protein kinase receptor R3 Precursor (SKR3) (EC 2.7.11.30)(Activin receptor-like kinase 1)(ALK-1)(TGF-B superfamily receptor type I) (TSR-I)
304	1	C	ENSP00000315775<br>ENSG00000103051	291<br>287<br>287<br>287<br>273<br>270<br>279<br>297<br>265<br>286	egiarivethqpivetyygpgr--lytl<br>egiarivethqpivetyygpgr--lytl<br>egiarivethqpivetyygpgr--lftl<br>egiarivethqpivetyygpgr--lftl<br>egiarvvethqpivetyygpgr--lytl<br>egiarivethqpiletyygpgr--lyml<br>egiarivethqpivetyygpgr--lhtl<br>egvaeiiesnlpvlehsyglek--lldf<br>enfarvvevnQPIIEAFYgqassslidm<br>kivstiindhskviatcygkkh--mvhv	Hs_ENSP00000315775<br>Bt_ENSBTAP00000017269<br>Rn_ENSRNOP00000023924<br>Mm_ENSMUSP00000034203<br>Gg_ENSGALP00000004091<br>Xt_ENSXETP00000034557<br>Dr_ENSDARP00000070899<br>Ce_CE26151<br>Dm_FBpp0079628<br>Sc_YPR105C	NP_056201 Conserved oligomeric Golgi complex subunit 4 (COG complex subunit 4)(Component of oligomeric golgi complex 4)
305	1	CI	ENSP00000309020<br>ENSG00000162688	179<br>179<br>179	lsrscyslanQLELNPDFsrp-nrkytw<br>lsrscysladQLELNPDFsrp-nkkytw<br>lsrscysladQLELNPDFsrp-nrrytw	Hs_ENSP00000309020<br>Bt_ENSBTAP00000008518<br>Rn_ENSRNOP00000052593	NP_000634 Glycogen debranching enzyme (Glycogen debrancher)

				178	lsrscysladQLELNPDFsrp-skrytw	Mm_ENSMUSP00000044012	
				179	lsrscysladqlevnpefssh-nkkctw	Gg_ENSGALP00000008669	
				-	....	Xt	
				179	esrscysladQLELNPDFsps-gqtytw	Dr_ENSDARP00000006424	
				165	isnssyslsdhhsliktiqsq-dqkfgf	Ce_CE18115	
				288	gsrscyslrdqlkvnshfapqkggkisf	Dm_FBpp0071526	
				192	esnspysiydqlqfdqehfks------p	Sc_YPR184W	
306	1	C	ENSP00000196061 ENSG00000083444	306	qptpfvslffQRLLRLHYpqkhmrlfih	Hs_ENSP00000196061	NP_000293 Procollagen-lysine,2-oxoglutarate 5-dioxygenase 1 Precursor (EC 1.14.11.4)(Lysyl hydroxylase 1)(LH1)
				305	qptpflslffQRLLRLHYpqkrlrlfih	Bt_ENSBTAP00000002658	
				305	qptpflslffrrllhlrypqkqmrlfih	Rn_ENSRNOP00000010433	
				307	qptpflslfflrllrlrypqkqmrlfih	Mm_ENSMUSP00000019199	
				309	qptpflsqfflrlrnlhypkqriqifih	Gg_ENSGALP00000007204	
				310	qptpfvseffkrlnnlnypkkriqlyin	Xt_ENSXETP00000028621	
				309	qptpfvtvfferlfnlkypknrlklfiy	Dr_ENSDARP00000077830	
				-	....	Ce	
				-	....	Dm	
				-	....	Sc	
307	1	CI	ENSP00000226355 ENSG00000079557	120	fshccskvdaQRRLCFFYnkksdvgflp	Hs_ENSP00000226355	NP_001124 Afamin Precursor (Alpha-albumin)(Alpha-Alb)
				-	....	Bt	
				120	fshccrqagfQRRLCFFYnkkanvgflp	Rn_ENSRNOP00000003850	
				120	fshccgkagfprrlcffynkkanvgflp	Mm_ENSMUSP00000108804	
				-	....	Gg	
				-	....	Xt	
				-	....	Dr	
				-	....	Ce	
				-	....	Dm	
				-	....	Sc	
308	1	CI	ENSP00000332756 ENSG00000101974	369	pvsmyvtvemQKFLGSFFiswdkdfyde	Hs_ENSP00000332756	NP_775965 Probable phospholipid-transporting ATPase IG (EC 3.6.3.1)(ATPase class I type 11C)(ATPase IG)(ATPase IQ)(ATPase class VI type 11C)
				369	pvsmyvtvemQKFLGSFFiswdndfyde	Bt_ENSBTAP00000021914	
				357	pvsmyvtvemQKFLGSFFiswdkdffde	Rn_ENSRNOP00000048676	
				366	pvsmyvtvemQKFLGSFFiswdkdffde	Mm_ENSMUSP00000099066	
				364	pvsmyvtvemQKFLGSFFiswdkemyde	Gg_ENSGALP00000010676	
				356	pvsmyvtvemQKFLGSFFiawdmemfde	Xt_ENSXETP00000039647	
				353	pvsmyvtvemQKFLGSFFikwdndffdp	Dr_ENSDARP00000052937	
				-	....	Ce	
				-	....	Dm	
				-	....	Sc	
309	1	CI	ENSP00000380476 ENSG00000130595	84	krqnkdlmelQALIDSHFearkkeeeel	Hs_ENSP00000380476	Troponin T, fast skeletal muscle (TnTf)(Fast skeletal muscle troponin T)(fTnT)(Beta TnTF)
				88	krqnkdlmelQALIDSHFearkkeeeel	Bt_ENSBTAP00000008422	
				86	krqnkdlmelQALIDSHFearkkeeeel	Rn_ENSRNOP00000047850	
				86	krqnkdlmelQALIDSHFearkkeeeel	Mm_ENSMUSP00000101578	
				93	krqnkdlielQALIDSHFearrkeeeel	Gg_ENSGALP00000038406	
				90	krqnkdlielQSLIDTHFearkkeeeel	Xt_ENSXETP00000053620	
				103	krqnkdlvelQALIDAHFehrkkeeeel	Dr_ENSDARP00000035181	
				-	....	Ce	
				72	kqrskeedelkklekqakrkvtraeee	Dm_FBpp0073682	
				-	....	Sc	
310	1	C	ENSP00000384907 ENSG00000196169	614	rslcdeiiqgQRQIIDDYnlavpqrlee	Hs_ENSP00000384907	NP_694941 Kinesin-like protein KIF19
				614	lslceeiiraqrqliqdcnlavpqhlke	Bt_ENSBTAP00000006057	
				548	----------------------------	Rn_ENSRNOP00000035641	
				613	rslcdeiiqgQRQIIDDYnlevprhlee	Mm_ENSMUSP00000081398	
				570	ra--------------------------	Gg_ENSGALP00000007161	
				609	rslcdeiikqqrriiydhnltvphqled	Xt_ENSXETP00000028031	
				604	rhlcheiiqqqrqfiddhsllvpphlqe	Dr_ENSDARP00000069468	
				-	....	Ce	
				630	rrlcediissqrrlieegnvdlpdelre	Dm_FBpp0089282	
				-	....	Sc	
311	1	C	ENSP00000380439 ENSG00000134452	108	tkrgsrgqgsQRCIPEFFlagkqpctnd	Hs_ENSP00000380439	NP_835363 F-box only protein 18 (EC 3.6.1.-)(F-box DNA helicase 1)
				55	tkrgargqgclrysldfflagqqpctnd	Bt_ENSBTAP00000005426	
				57	tkgrnrgrgcQRYISEFFlashqhctnd	Rn_ENSRNOP00000025228	
				90	tkgrnrgrgcQRYISEFFlaghqhctnd	Mm_ENSMUSP00000100031	
				59	trtqpgaqgrqknimdyfkvsqr----q	Gg_ENSGALP00000013249	
				56	kersarg--tilyfreffvlcdnlckne	Xt_ENSXETP00000054197	
				60	tkrrrrg---tvsfissdgagqqkgitd	Dr_ENSDARP00000086063	
				-	....	Ce	
				-	....	Dm	
				-	....	Sc	
312	1	CI	ENSP00000264454 ENSG00000093183	237	flvpfvacifQCYLYLFYspartmkvvl	Hs_ENSP00000264454	NP_116752 Vesicle-trafficking protein SEC22c (SEC22 vesicle-trafficking protein
				237	flipfvacifQCYLYLFYspartmkvvl	Bt_ENSBTAP00000008631	
				-	....	Rn	

				237<br>238<br>229<br>237<br>-<br>-<br>-	ffipsvacivQCYLYLFYspartlkvll<br>fflpflacvfqcylylyyssstrkvktfv<br>fltafsanilqcylylfhcsarkrkacg<br>flvaflccvcqchlylfhtcqkrlksft<br>............................<br>............................<br>............................	Mm_ENSMUSP00000107185<br>Gg_ENSGALP00000008440<br>Xt_ENSXETP00000013555<br>Dr_ENSDARP00000087023<br>Ce<br>Dm<br>Sc	homolog C)(SEC22 vesicle-trafficking protein-like 3)
313	1	CI	ENSP00000264499<br>ENSG00000138686	705<br>707<br>705<br>705<br>705<br>-<br>708<br>708<br>-<br>-	lleilds-ydQNALISFFdaa-------<br>lleilds-ydQNALIAFFdaa-------<br>lleilds-ydQNTLISFFdaa-------<br>lleilds-ydQNTLISFFdaa-------<br>llelldg-ceQERLVAFFeaas------<br>............................<br>llailnn-yeldslmeffsea-------<br>irdlistdyslenmqtlfknamnd----<br>............................<br>............................	Hs_ENSP00000264499<br>Bt_ENSBTAP00000006505<br>Rn_ENSRNOP00000021453<br>Mm_ENSMUSP00000103791<br>Gg_ENSGALP00000019359<br>Xt<br>Dr_ENSDARP00000078268<br>Ce_CE23024<br>Dm<br>Sc	NP_060660 Bardet-Biedl syndrome 7 protein (BBS2-like protein 1)
314	1	CI	ENSP00000333482<br>ENSG00000183909	2<br>36<br>-<br>-<br>-<br>-<br>-<br>-<br>-<br>-	---------mQFFLFLFFslfyvgiilg<br>llgltqslgmQFLLFLFFsvlylgiilg<br>............................<br>............................<br>............................<br>............................<br>............................<br>............................<br>............................<br>............................	Hs_ENSP00000333482<br>Bt_ENSBTAP00000048720<br>Rn<br>Mm<br>Gg<br>Xt<br>Dr<br>Ce<br>Dm<br>Sc	Seven transmembrane helix receptor
315	1	CI	ENSP00000360497<br>ENSG00000125352	198<br>198<br>196<br>196<br>186<br>183<br>186<br>177<br>196<br>140	hlratvrwdyQPDICKDYketgfcgfgd<br>hlratvrwdyQPDICKDYketgfcgfgd<br>hlratvrwdyQPDICKDYketgfcgfgd<br>hlratvrwdyQPDICKDYketgfcgfgd<br>hlratvrwdyQPDICKDYketgfcgfgd<br>hlratvrwdyQPDICKDYketgfcgfgd<br>hlratvrwdyQPDICKDYketgfcgfgd<br>flrqtvrwdfapdickdyketgfctfgd<br>hlratvrwdyQPDICKDYketgycgfgd<br>nirttvlmdfqpdvckdykqtgycgygd	Hs_ENSP00000360497<br>Bt_ENSBTAP00000007927<br>Rn_ENSRNOP00000047839<br>Mm_ENSMUSP00000042569<br>Gg_ENSGALP00000000742<br>Xt_ENSXETP00000004366<br>Dr_ENSDARP00000055277<br>Ce_CE26718<br>Dm_FBpp0083244<br>Sc_YLR323C	NP_008909 RING finger protein 113A (Zinc finger protein 183)
316	1	CI	ENSP00000381577<br>ENSG00000115464	1976<br>1825<br>1370<br>1824<br>1846<br>1981<br>-<br>-<br>2097<br>-	mdkqplntgeQKDMTEFFtdlitkieem<br>mdkqplntgeQKDMTEFFtdlitkieem<br>mdkqplntgeQKDMTEFFtdlitkveem<br>mdkqplntgeQKDMTEFFtdlitkveem<br>mdkqplntgeQKDMTEFFtdlitkieem<br>mdkqplntgeQKDMTEFFtdlitkieem<br>............................<br>............................<br>mdhqplntgeQKDMAEFFidlvskledm<br>............................	Hs_ENSP00000381577<br>Bt_ENSBTAP00000007921<br>Rn_ENSRNOP00000033131<br>Mm_ENSMUSP00000099007<br>Gg_ENSGALP00000007080<br>Xt_ENSXETP00000004725<br>Dr<br>Ce<br>Dm_FBpp0084048<br>Sc	NP_055524 Ubiquitin carboxyl-terminal hydrolase 34 (EC 3.1.2.15)(Ubiquitin thioesterase 34)(Ubiquitin-specific-processing protease 34)(Deubiquitinating enzyme 34)
316	2	C	ENSP00000381577<br>ENSG00000115464	2799<br>2648<br>2193<br>2647<br>2669<br>2804<br>-<br>-<br>3077<br>-	epaiatnhnkqallsfwynvcadcpeni<br>epaiatnhnkqallsfwynvcadcpeni<br>epaiatnhnkqallsfwynvctecpeni<br>epaiatnhnkqallsfwynvcadcpeni<br>epaiatnhnkqallsfwynvcvdcpenv<br>epaiatnhnkqallsfwynvcvdcpenv<br>............................<br>............................<br>epsvpahhnkhalltfwhhslvdcpena<br>............................	Hs_ENSP00000381577<br>Bt_ENSBTAP00000007921<br>Rn_ENSRNOP00000033131<br>Mm_ENSMUSP00000099007<br>Gg_ENSGALP00000007080<br>Xt_ENSXETP00000004725<br>Dr<br>Ce<br>Dm_FBpp0084048<br>Sc	NP_055524 Ubiquitin carboxyl-terminal hydrolase 34 (EC 3.1.2.15)(Ubiquitin thioesterase 34)(Ubiquitin-specific-processing protease 34)(Deubiquitinating enzyme 34)
317	1	CI	ENSP00000240333<br>ENSG00000121073	190<br>223<br>190<br>190<br>196<br>210<br>197<br>197<br>189<br>193	ltldgltgvsQDHMRAHYqt--------<br>ltldgltgvsQDHMRAHYqt--------<br>ltldgltgvsQDHMRAHYqt--------<br>ltldgltgvsQDHMRAHYqt--------<br>ltldgltgvsQDHMRAHYqt--------<br>ltldgltgvsQDHMRAHFqt--------<br>ltldgltgvvqdhmrgrfqt--------<br>lamdgtttsiqdrikksyqr--------<br>lsmdgltgavqeriraasap--------<br>lfldgltnatqdkllkankakekgkqtl	Hs_ENSP00000240333<br>Bt_ENSBTAP00000009034<br>Rn_ENSRNOP00000006179<br>Mm_ENSMUSP00000021243<br>Gg_ENSGALP00000039561<br>Xt_ENSXETP00000033116<br>Dr_ENSDARP00000094832<br>Ce_CE24129<br>Dm_FBpp0083420<br>Sc_YPL244C	NP_005818 Solute carrier family 35 member B1 (UDP-galactose transporter-related protein 1)(UGTrel1)(hUGTrel1)
318	1	CI	ENSP00000306888<br>ENSG00000162391	137<br>138<br>141	qwldavlgssQKGIKLDFknikavgpsl<br>qwleavlassQKGIKLDFksikavgpsl<br>ewleavlassQKGIKLDFkslkavgpsl	Hs_ENSP00000306888<br>Bt_ENSBTAP00000026380<br>Rn_ENSRNOP00000010288	NP_788954 Protein FAM151A

				141	ewleavlassQKGIKLDFkslkavgpsl	Mm_ENSMUSP00000047860	
				-	....	Gg	
				87	nwlnsvlkstk-gikldfksiqavgpsl	Xt_ENSXETP00000043235	
				132	ewldavlkskkvgvkldfksisavepsl	Dr_ENSDARP00000075469	
				-	....	Ce	
				-	....	Dm	
				-	....	Sc	
319	1	C	ENSP00000313420 ENSG00000121031	2517	qglidenpglqliirnfwshetrlpsnt	Hs_ENSP00000313420	NP_008835 DNA-dependent protein kinase catalytic subunit (DNA-PK catalytic subunit) (DNA-PKcs)(EC 2.7.11.1) (DNPK1)(p460)
				2522	qgltdenpglqltirnfwshetrlpsnt	Bt_ENSBTAP00000022631	
				2512	qglidenlglqliirnfwshetrlpsnt	Rn_ENSRNOP00000037412	
				2514	qglidenvglqliirnfwshetrlpsnt	Mm_ENSMUSP00000023352	
				2520	qglidenaelqlivrnfwsdetrlpani	Gg_ENSGALP00000024527	
				-	....	Xt	
				-	....	Dr	
				-	....	Ce	
				-	....	Dm	
				-	....	Sc	
319	2	C	ENSP00000313420 ENSG00000121031	3563	fvarikskldqggviqdfinaldqlsnp	Hs_ENSP00000313420	NP_008835 DNA-dependent protein kinase catalytic subunit (DNA-PK catalytic subunit) (DNA-PKcs)(EC 2.7.11.1) (DNPK1)(p460)
				3563	fvarikakldregvvqdfinaleqlsnp	Bt_ENSBTAP00000022631	
				3565	fverikskldqggviqgfinaldqlsnp	Rn_ENSRNOP00000037412	
				3564	fverikskldhgevihsfinaldqlsnp	Mm_ENSMUSP00000023352	
				3575	fvasiknkldrggvvqdfihaleqlsnp	Gg_ENSGALP00000024527	
				-	....	Xt	
				-	....	Dr	
				-	....	Ce	
				-	....	Dm	
				-	....	Sc	
320	1	CI	ENSP00000344984 ENSG00000187878	62	seqelpfyflQKLLMMDYelrylvfkdd	Hs_ENSP00000344984	Interferon-induced very large GTPase 1
				39	serelpfyflQKLLMLDYglryliikhe	Bt_ENSBTAP00000049852	
				-	....	Rn	
				-	....	Mm	
				-	....	Gg	
				39	eekqlpfyflQKLLMLDYsaryvqckki	Xt_ENSXETP00000006920	
				78	geeeliqtyiqkllmmnykaryitvkit	Dr_ENSDARP00000066051	
				-	....	Ce	
				-	....	Dm	
				-	....	Sc	
321	1	CI	ENSP00000261866 ENSG00000104133	2354	vpdwaeilyqQVILKGDFnyleefkqqr	Hs_ENSP00000261866	NP_079413 Spatacsin (Spastic paraplegia 11 protein) (Colorectal carcinoma-associated protein)
				2342	vpdwaeilyqQVILKGDFnyleefkqqr	Bt_ENSBTAP00000000470	
				1538	vpdwaevlyqQVILKGDFsyleefkqqk	Rn_ENSRNOP00000022875	
				2341	vpdwaevlyqQVILKGDFsyleefkqqk	Mm_ENSMUSP00000037543	
				2359	vpdwaevlyqqviakgdfsyleefkqqr	Gg_ENSGALP00000013297	
				-	....	Xt	
				-	....	Dr	
				-	....	Ce	
				1713	diswseailsqfvvlqgvnylqeylchq	Dm_FBpp0071821	
				-	....	Sc	
322	1	CI	ENSP00000371958 ENSG00000010278	86	lgccgavqesQCMLGLFFgfllvifaie	Hs_ENSP00000371958	NP_001760 CD9 antigen (p24) (Leukocyte antigen MIC3) (Motility-related protein)(MRP-1)(Tetraspanin-29)(Tspan-29) (5H9 antigen)(Cell growth-inhibiting gene 2 protein)(CD9 antigen)
				84	lgccgavqesQCMLGLFFsfllvifaie	Bt_ENSBTAP00000019643	
				84	lgccgavqesQCMLGLFFgfllvifaie	Rn_ENSRNOP00000026633	
				84	lgccgavqesQCMLGLFFgfllvifaie	Mm_ENSMUSP00000032492	
				84	lgccgalqesQCMLGLFFvflfvifale	Gg_ENSGALP00000029405	
				84	lgccgaiqesecmlglffafllvifave	Xt_ENSXETP00000050947	
				84	fgccgaiqespcmlglfffllvifave	Dr_ENSDARP00000019805	
				-	....	Ce	
				-	....	Dm	
				-	....	Sc	
323	1	CI	ENSP00000334176 ENSG00000168280	649	rdlnqklqleQEKLSSDYnklkiedqer	Hs_ENSP00000334176	NP_004513 Kinesin heavy chain isoform 5C (Kinesin heavy chain neuron-specific 2)
				-	....	Bt	
				745	rdlnqklqleQERLSSDYnklkiedqer	Rn_ENSRNOP00000006341	
				745	rdlnqklqleQERLSSDYnklkiedqer	Mm_ENSMUSP00000028102	
				784	rdmnqklqleQEKLSADYdklkiedqer	Gg_ENSGALP00000037000	
				-	....	Xt	
				-	....	Dr	
				-	....	Ce	
				-	....	Dm	
				-	....	Sc	
324	1	CI	ENSP00000297761 ENSG00000127359	178	mhflenqvrhQLEMPGHYshlaafy---	Hs_ENSP00000297761	NP_001073861 Protein LCHN
				90	mhflenqvrhqletpghyshlaafy---	Bt_ENSBTAP00000022453	
				178	mhflenqvrhQLEMPGHYshlaafy---	Rn_ENSRNOP00000015921	

				178<br>111<br>-<br>-<br>-<br>-<br>153	mhflenqvrhQLEMPGHYshlaafy---<br>mhflenqvrhQLEMPGHYshleafy---<br>............................<br>............................<br>............................<br>............................<br>kkhfykeiyhayaanryssylesllgqw	Mm_ENSMUSP00000099031<br>Gg_ENSGALP00000020971<br>Xt<br>Dr<br>Ce<br>Dm<br>Sc_YKL047W	
325	1	CI	ENSP00000369719<br>ENSG00000080603	865<br>872<br>855<br>847<br>-<br>-<br>797<br>805<br>-<br>-	hvircrlskrQRCLYDDFmaqtttketl<br>hvircrlskrQRCLYDDFmaqtttketl<br>hvircrlskrQRCLYDDFmaqtttketl<br>hvircrlskrQRCLYDDFmaqtttketl<br>............................<br>............................<br>hvvrcrlskrQRFLYDDFmaqastretl<br>hivncslskrQRYLYDDFmsrrstkenl<br>............................<br>............................	Hs_ENSP00000369719<br>Bt_ENSBTAP00000018503<br>Rn_ENSRNOP00000051879<br>Mm_ENSMUSP00000081611<br>Gg<br>Xt<br>Dr_ENSDARP00000070685<br>Ce_CE40241<br>Dm<br>Sc	NP_006653 Helicase SRCAP (EC 3.6.1.-)(Snf2-related CBP activator)(Domino homolog 2)
326	1	C	ENSP00000297313<br>ENSG00000147509	9<br>9<br>0<br>9<br>0<br>0<br>0<br>-<br>-<br>-	--mpqlsqdnQECLQKHFsrpsiwtqfl<br>--mprlsqdnqqghqkhfsrpsrriqfl<br>----------------------------<br>--mpslsqenqgyleehltrqaaqaqll<br>----------------------------<br>----------------------------<br>----------------------------<br>............................<br>............................<br>............................	Hs_ENSP00000297313<br>Bt_ENSBTAP00000004487<br>Rn_ENSRNOP00000010682<br>Mm_ENSMUSP00000113398<br>Gg_ENSGALP00000030270<br>Xt_ENSXETP00000017746<br>Dr_ENSDARP00000056734<br>Ce<br>Dm<br>Sc	NP_733466 Regulator of G-protein signaling 20 (RGS20)(Regulator of Gz-selective protein signaling 1)(Gz-selective GTPase-activating protein)(G(z)GAP)
327	1	CI	ENSP00000341985<br>ENSG00000156162	418<br>418<br>412<br>417<br>418<br>415<br>-<br>-<br>-<br>-	psqdfflrltQSSLLPFYilvliicfls<br>psqdfflrltQSSLLPFYilvliicfls<br>psqdfflrltQSSLLPFYvlvlticlls<br>psqdfffrltQSSLLPFYvlvliicIls<br>psqdfflrltQSSLLPFYilvliiclfs<br>psqdyllrltQSSLLPFYilvlficmcs<br>............................<br>............................<br>............................<br>............................	Hs_ENSP00000341985<br>Bt_ENSBTAP00000018107<br>Rn_ENSRNOP00000034948<br>Mm_ENSMUSP00000081954<br>Gg_ENSGALP00000025695<br>Xt_ENSXETP00000014585<br>Dr<br>Ce<br>Dm<br>Sc	NP_861452 Protein dpy-19 homolog 4 (Dpy-19-like protein 4)
328	1	CI	ENSP00000349325<br>ENSG00000206190	1055<br>1060<br>1073<br>1069<br>1062<br>1042<br>-<br>-<br>-<br>-	gvgisgqegmQAVMASDFavpkfryler<br>gvgisgqegmQAVMASDFavpkfryler<br>gvgisgqegmQAVMASDFavprfryler<br>gvgisgqegmQAVMASDFavprfryler<br>gvgisgqegmQAVMASDFaiprfrhlek<br>gvgisgqegmQAVMASDFavprfrhlek<br>............................<br>............................<br>............................<br>............................	Hs_ENSP00000349325<br>Bt_ENSBTAP00000035070<br>Rn_ENSRNOP00000023888<br>Mm_ENSMUSP00000055608<br>Gg_ENSGALP00000026980<br>Xt_ENSXETP00000052504<br>Dr<br>Ce<br>Dm<br>Sc	NP_077816 Probable phospholipid-transporting ATPase VA (ATPVA)(EC 3.6.3.1)(Aminophospholipid translocase VA)
328	2	C	ENSP00000349325<br>ENSG00000206190	1483<br>1492<br>1505<br>1501<br>1501<br>1299<br>-<br>-<br>-<br>-	vqphsgrsglqgpdhrlligassrrsq-<br>appdqdlcdlsgqttdcf----------<br>pqpdqdlhnlqgqvtgyf----------<br>pqpeqdlhsfqgqvtgyl----------<br>sslqedfkdlkeqssnnfqdkmkgtp--<br>----------------------------<br>............................<br>............................<br>............................<br>............................	Hs_ENSP00000349325<br>Bt_ENSBTAP00000035070<br>Rn_ENSRNOP00000023888<br>Mm_ENSMUSP00000055608<br>Gg_ENSGALP00000026980<br>Xt_ENSXETP00000052504<br>Dr<br>Ce<br>Dm<br>Sc	NP_077816 Probable phospholipid-transporting ATPase VA (ATPVA)(EC 3.6.3.1)(Aminophospholipid translocase VA)
329	1	CI	ENSP00000262188<br>ENSG00000082014	401<br>404<br>401<br>401<br>380<br>374<br>376<br>364<br>-<br>411	kihetiesinQLKIQRDFmlsfsrdpkg<br>kihetiesinQLKIQRDFmlsfsrdpkg<br>kihetiesinQLKIQRDFmlsfsrdpkg<br>kihetiesinQLKIQRDFmlsfsrdpkg<br>kihetiesinQLKIQRDFmlsfskdpkg<br>kihetiesinQLKIQRDFmlsfsrdpkg<br>kihetiesinQLKIQRDFmlsfsrdpkg<br>kifdlvdqinemklrrdfflrfsnepsg<br>............................<br>eitklalqlnssaqkyqffhelslhpre	Hs_ENSP00000262188<br>Bt_ENSBTAP00000019109<br>Rn_ENSRNOP00000013876<br>Mm_ENSMUSP00000030791<br>Gg_ENSGALP00000021267<br>Xt_ENSXETP00000031534<br>Dr_ENSDARP00000056640<br>Ce_CE01687<br>Dm<br>Sc_YCR052W	NP_001003801 SWI/SNF-related matrix-associated actin-dependent regulator of chromatin subfamily D member 3 (60 kDa BRG-1/Brm-associated factor subunit C)(BRG1-associated factor 60C)
329	2	C	ENSP00000262188<br>ENSG00000082014	456<br>459<br>456	raefyhqpwsqeavsryfyckiqqrrqe<br>raefyhqpwsqeavsryfyckiqqrrqe<br>raefyhqpwsqeavsryfyckiqqrrqe	Hs_ENSP00000262188<br>Bt_ENSBTAP00000019109<br>Rn_ENSRNOP00000013876	NP_001003801 SWI/SNF-related matrix-associated actin-dependent regulator of

				456<br>435<br>429<br>431<br>419<br>-<br>467	raefyhqpwsqeavsryfyckiqqrqe<br>raefyhepwsqeavsryfyckiqqrqe<br>radfyqepwsqeavsryfyckiqqrqe<br>raefynqpwsqeavsryfyckiqqrqe<br>yattysttdtdegvsrymyqkiqqkrae<br>............................<br>tsdiysnnnndrslmgnisllysqgrl-	Mm_ENSMUSP00000030791<br>Gg_ENSGALP00000021267<br>Xt_ENSXETP00000031534<br>Dr_ENSDARP00000056640<br>Ce_CE01687<br>Dm<br>Sc_YCR052W	chromatin subfamily D member 3 (60 kDa BRG-1/Brm-associated factor subunit C) (BRG1-associated factor 60C)
330	1	CI	ENSP00000314973<br>ENSG00000161542	137<br>166<br>166<br>166<br>164<br>164<br>-<br>-<br>-<br>-	lthiitmdlhQKEIQGFFsfpvdnlras<br>lthiitmdlhQKEIQGFFsfpvdnlras<br>lthiitmdlhQKEIQGFFcfpvdnlras<br>lthiitmdlhQKEIQGFFsfpvdnlras<br>lthiitmdlhQKEIQGFFsfpvdnlras<br>lthiitmdlhQKEIQGFFnfpvdnlras<br>............................<br>............................<br>............................<br>............................	Hs_ENSP00000314973<br>Bt_ENSBTAP00000018338<br>Rn_ENSRNOP00000013573<br>Mm_ENSMUSP00000101999<br>Gg_ENSGALP00000002881<br>Xt_ENSXETP00000049047<br>Dr<br>Ce<br>Dm<br>Sc	NP_002757 Phosphoribosyl pyrophosphate synthetase-associated protein 1 (PRPP synthetase-associated protein 1) (39 kDa phosphoribosypyrophosphate synthetase-associated protein) (PAP39)
331	1	C	ENSP00000366387<br>ENSG00000165072	409<br>369<br>195<br>409<br>357<br>362<br>404<br>-<br>-<br>-	lygpslpgnlQYCLRFHYaiygflkmsd<br>lygpslpgnqqyclrfyyaisgflkmsd<br>lygpslpgnmqycvrfhyaiigflkmsd<br>lygpslpgnmqycvrfhyaifgflkmsd<br>lygptlpgnlqfclrfyyalygffkmsg<br>lygpslpgniQYCLRFFYslygfykttd<br>lfgpflpgnhkyclrfyyalhgfmkidn<br>............................<br>............................<br>............................	Hs_ENSP00000366387<br>Bt_ENSBTAP00000023092<br>Rn_ENSRNOP00000034701<br>Mm_ENSMUSP00000045432<br>Gg_ENSGALP00000024341<br>Xt_ENSXETP00000003548<br>Dr_ENSDARP00000077638<br>Ce<br>Dm<br>Sc	NP_694999 MAM domain-containing protein 2 Precursor
332	1	CI	ENSP00000273600<br>ENSG00000114841	2511<br>-<br>2745<br>2510<br>2394<br>-<br>-<br>-<br>-<br>-	evtfnkvcpfQPILYGDFmspgsdvksy<br>............................<br>gvafnkvcpfQPILYGDFmspgsdvksy<br>gvafnkvcpfQPILYGDFmspgsdvksy<br>dttfdevvpsQPVLFGDFmepgahikly<br>............................<br>............................<br>............................<br>............................<br>............................	Hs_ENSP00000273600<br>Bt<br>Rn_ENSRNOP00000032434<br>Mm_ENSMUSP00000043281<br>Gg_ENSGALP00000006832<br>Xt<br>Dr<br>Ce<br>Dm<br>Sc	Dynein heavy chain 1, axonemal (Axonemal beta dynein heavy chain 1)(Ciliary dynein heavy chain 1)(Heat shock regulated protein 1) (XLHSRF-1)(hDHC7)
333	1	C	ENSP00000362206<br>ENSG00000102271	93<br>93<br>0<br>93<br>0<br>92<br>-<br>-<br>-<br>-	ahqravqnlqQHNLIVHFqanedtpksv<br>ahqralqnwhsgkliehlranedtpkav<br>----------------------------<br>thqrvlqnwhqqnvivhlqanedtpkav<br>----------------------------<br>aynrsiqswqqqniienfqasedipkpg<br>............................<br>............................<br>............................<br>............................	Hs_ENSP00000362206<br>Bt_ENSBTAP00000006877<br>Rn_ENSRNOP00000006992<br>Mm_ENSMUSP00000038080<br>Gg_ENSGALP00000011127<br>Xt_ENSXETP00000034677<br>Dr<br>Ce<br>Dm<br>Sc	NP_476503 Kelch-like protein 4
334	1	CI	ENSP00000221494<br>ENSG00000104897	205<br>205<br>205<br>195<br>-<br>205<br>205<br>-<br>206<br>-	fwthwnretkQFFLQFHFkmekppapps<br>fwthwnretkQFFLQFHFkmekppapps<br>fwthwnretkQFFLQFHFkmekppapps<br>f----------fflqfhfkmekppapps<br>............................<br>fwthwnretkQFFLQFHFkmekppqapt<br>fwthwnretkQFFLQFHFkmekalvaps<br>............................<br>fwthwnrdtkqfflqfafkfep----ki<br>............................	Hs_ENSP00000221494<br>Bt_ENSBTAP00000035656<br>Rn_ENSRNOP00000026202<br>Mm_ENSMUSP00000020432<br>Gg<br>Xt_ENSXETP00000053596<br>Dr_ENSDARP00000003884<br>Ce<br>Dm_FBpp0075676<br>Sc	NP_009096 Splicing factor 3A subunit 2 (Spliceosome-associated protein 62)(SAP 62) (SF3a66)
335	1	CI	ENSP00000325690<br>ENSG00000142453	159<br>128<br>160<br>160<br>-<br>130<br>134<br>-<br>154<br>-	yfqfygylsqQQNMMQDYvrtgtyqrai<br>yfqfygylsqQQNMMQDYvrtgtyqrai<br>yfqfygylsqQQNMMQDYvrtgtyqrai<br>yfqfygylsqQQNMMQDYvrtgtyqrai<br>............................<br>yfqfygylsqQQNMMQDYvrtgtyqrai<br>yfqfygylsqQQNMMQDYvrtgtyqrai<br>............................<br>yfqfygylsqQQNMMQDYvrtstyqrai<br>............................	Hs_ENSP00000325690<br>Bt_ENSBTAP00000015985<br>Rn_ENSRNOP00000049428<br>Mm_ENSMUSP00000111052<br>Gg<br>Xt_ENSXETP00000020625<br>Dr_ENSDARP00000016959<br>Ce<br>Dm_FBpp0081628<br>Sc	NP_954592 Histone-arginine methyltransferase CARM1 (EC 2.1.1.125)(EC 2.1.1.-)(Protein arginine N-methyltransferase 4) (Coactivator-associated arginine methyltransferase 1)
336	1	CI	ENSP00000383365<br>ENSG00000184470	458<br>444<br>461	vkmvclreppQLVLGLHFlgpnagevtq<br>ikmvclreppQLVLGLHFlgpnageviq<br>ikmvcmreppQLVLGLHFlgpnagevtq	Hs_ENSP00000383365<br>Bt_ENSBTAP00000053158<br>Rn_ENSRNOP00000002593	NP_006431 Thioredoxin reductase 2, mitochondrial Precursor (EC 1.8.1.9)

				463<br>465<br>438<br>-<br>-<br>530<br>-	ikmvcmreppQLVLGLHFlgpnagevtq<br>ikmvclrereQRILGLHFigpnageviq<br>ikiiclrtqdQRILGLHFtgpnageviq<br>............................<br>............................<br>lkavaerhgdqrvyglhyigpvageviq<br>............................	Mm_ENSMUSP00000081006<br>Gg_ENSGALP00000003091<br>Xt_ENSXETP00000013826<br>Dr<br>Ce<br>Dm_FBpp0071116<br>Sc	(Thioredoxin reductase TR3)(TR-beta)(Selenoprotein Z)(SelZ)
337	1	CI	ENSP00000290291<br>ENSG00000060491	229<br>221<br>219<br>219<br>223<br>-<br>236<br>-<br>-<br>-	slgelglehfQAPLVRFFleetlvrrel<br>slgelglahyQAPLARFFleetlvrrql<br>slgelglehyQAPLVRFFleetlvqhkl<br>slgelglehyQAPLVRFFleetlvqhkl<br>clgemgyehyQVHLVKFFltetlvnetl<br>............................<br>clgtlglehyQAPLVKFFlfetlvqgel<br>............................<br>............................<br>............................	Hs_ENSP00000290291<br>Bt_ENSBTAP00000020676<br>Rn_ENSRNOP00000012612<br>Mm_ENSMUSP00000029087<br>Gg_ENSGALP00000038915<br>Xt<br>Dr_ENSDARP00000075122<br>Ce<br>Dm<br>Sc	NP_031372 Opioid growth factor receptor (OGFr)(Zeta-type opioid receptor)(7-60 protein)
338	1	CI	ENSP00000325691<br>ENSG00000176395	82<br>-<br>64<br>64<br>-<br>-<br>-<br>-<br>-<br>-	iavspgtaieQKDMVTFYcttkdvniti<br>............................<br>ltvrqrvvteQRDMADFYcatnavnati<br>ltvnqstvteQREMAVFYcntnadniti<br>............................<br>............................<br>............................<br>............................<br>............................<br>............................	Hs_ENSP00000325691<br>Bt<br>Rn_ENSRNOP00000026060<br>Mm_ENSMUSP00000092344<br>Gg<br>Xt<br>Dr<br>Ce<br>Dm<br>Sc	NP_001096070 Carcinoembryonic antigen-related cell adhesion molecule 20 Precursor
339	1	C	ENSP00000225927<br>ENSG00000108784	186<br>186<br>184<br>184<br>69<br>-<br>188<br>192<br>205<br>-	qrvylalgltQAEINEFFtgpaflawgr<br>qrvylalgltqaeideyftgpaflawgr<br>qrvylalgltqseidnyftgpaflawgr<br>qrvylalgltqseidtyftgpaflawgr<br>qrvyralglnQTEIDAHFtgpaflawnr<br>............................<br>qevylslglnQTELDRFFsgpaflawnr<br>rdifmglgvqrdeldsyftsqaylawhr<br>vkvytdmglrmeeidehlagpafqawqr<br>............................	Hs_ENSP00000225927<br>Bt_ENSBTAP00000034678<br>Rn_ENSRNOP00000051302<br>Mm_ENSMUSP00000001802<br>Gg_ENSGALP00000035042<br>Xt<br>Dr_ENSDARP00000048045<br>Ce_CE32692<br>Dm_FBpp0079316<br>Sc	NP_000254 Alpha-N-acetylglucosaminidase Precursor (EC 3.2.1.50)(N-acetyl-alpha-glucosaminidase)(NAG)
339	2	C	ENSP00000225927<br>ENSG00000108784	569<br>554<br>566<br>567<br>444<br>-<br>570<br>578<br>589<br>-	dlldltrqavqelvslyyeearsaylsk<br>----------------------------<br>dlldvtrqavqelvsscyeeartaflnq<br>dlldvtrqavqelvslcyeeartaylkq<br>dladvtrqavqqlvadyyqrirdsfqrr<br>............................<br>dlvdvtrqalqllttefykdiksafqtq<br>dlndvmremtqfemgneaalsmseaflm<br>dlvditrqflqisadqlyinlrsayrkr<br>............................	Hs_ENSP00000225927<br>Bt_ENSBTAP00000034678<br>Rn_ENSRNOP00000051302<br>Mm_ENSMUSP00000001802<br>Gg_ENSGALP00000035042<br>Xt<br>Dr_ENSDARP00000048045<br>Ce_CE32692<br>Dm_FBpp0079316<br>Sc	NP_000254 Alpha-N-acetylglucosaminidase Precursor (EC 3.2.1.50)(N-acetyl-alpha-glucosaminidase)(NAG)
340	1	CI	ENSP00000377473<br>ENSG00000113645	92<br>0<br>92<br>92<br>92<br>-<br>-<br>-<br>140<br>-	edprvqwrreQEHMLKDYlvvaqealsa<br>----------------------------<br>edprvqwrreQEHMLKDYlvvaqealsa<br>edprvqwrreQEHMLKDYlvvaqealsa<br>edprvqwrreQEHMLKDYlvlaqeaiaa<br>............................<br>............................<br>............................<br>edprqewktvQEQMLSDYlsaaqdqlen<br>............................	Hs_ENSP00000377473<br>Bt_ENSBTAP00000051440<br>Rn_ENSRNOP00000011061<br>Mm_ENSMUSP00000018993<br>Gg_ENSGALP00000002819<br>Xt<br>Dr<br>Ce<br>Dm_FBpp0099467<br>Sc	Protein WWC1 (WW domain-containing protein 1)(Kidney and brain protein)(KIBRA)(HBeAg-binding protein 3)
341	1	CI	ENSP00000242576<br>ENSG00000076248	4<br>0<br>4<br>4<br>4<br>4<br>4<br>4<br>-<br>21	-------migQKTLYSFFspsparkrha<br>--------------mgvlcpgplgwgrk<br>-------migQKTLYSFFsptptgkrtt<br>-------migQKTLYSFFsptptgkrtt<br>-------migQKTLHCFFstapp-rkrs<br>-------migQRTINSFFgpaakkraap<br>-------migQKSIKSFFspaskkrnid<br>-------msktvripdmflkasaaskrk<br>............................<br>svmtvarkrkQTTIEDFFgtkkstneap	Hs_ENSP00000242576<br>Bt_ENSBTAP00000042650<br>Rn_ENSRNOP00000000872<br>Mm_ENSMUSP00000031587<br>Gg_ENSGALP00000033675<br>Xt_ENSXETP00000048248<br>Dr_ENSDARP00000062358<br>Ce_CE22594<br>Dm<br>Sc_YML021C	NP_550433 Uracil-DNA glycosylase (UDG)(EC 3.2.2.-)
342	1	C	ENSP00000373702<br>ENSG00000183977	4<br>9<br>0	-------nifhpvcrvfwksrewnmkts<br>--knwlvrqsqpvhrvfwkarqlytrkp<br>--------------rvflkskqwdekts	Hs_ENSP00000373702<br>Bt_ENSBTAP00000005303<br>Rn_ENSRNOP00000006325	PP2C-like domain-containing protein C3orf48

				7	----mnwelyssplrvfwrskvwdekts	Mm_ENSMUSP00000056682	
				0	----------------------------	Gg_ENSGALP00000030536	
				4	-------rknrnilrnfwng--------	Xt_ENSXETP00000013604	
				-	....	Dr	
				-	....	Ce	
				-	....	Dm	
				-	....	Sc	
343	1	CI	ENSP00000263243 ENSG00000100403	844	tpissregekQIQMPTDYadimmgyhcw	Hs_ENSP00000263243	Zinc finger CCCH domain-containing protein 7B (Rotavirus 'X'-associated non-structural protein)(RoXaN)
				846	tpvgsregekQIQMPTDYadimmgyhcw	Bt_ENSBTAP00000003608	
				845	tpissregekQIQMPTDYadimmgyhcw	Rn_ENSRNOP00000026205	
				829	tpissregekQIQMPTDYadimmgyhcw	Mm_ENSMUSP00000105181	
				848	tpltsregekQIQMPTDYadimmgyhcw	Gg_ENSGALP00000019488	
				795	asgpqketekQILMPTDYadlmagfhcy	Xt_ENSXETP00000013876	
				-	....	Dr	
				-	....	Ce	
				-	....	Dm	
				-	....	Sc	
344	1	CI	ENSP00000346733 ENSG00000131584	245	emerkhaaiqQRTLLQDFsydeskvefd	Hs_ENSP00000346733	Centaurin-beta-5 (Cnt-b5)
				232	emerkhaaiqQRTLLQDFsydepkvefd	Bt_ENSBTAP00000013003	
				245	emerkhaaiqQRTLLQDFsydepkvefd	Rn_ENSRNOP00000033674	
				245	emerkhaaiqQRTLLQDFsydepkvefd	Mm_ENSMUSP00000101209	
				245	emehkhamiqQRTLLQDFsyddskvefn	Gg_ENSGALP00000002713	
				230	emehkhatiqqr----dfsyddskadfn	Xt_ENSXETP00000002225	
				-	....	Dr	
				-	....	Ce	
				-	....	Dm	
				-	....	Sc	
345	1	CI	ENSP00000315664 ENSG00000154957	322	scqasgevpsQASLRGFFtedepgcfge	Hs_ENSP00000315664	NP_653281 Zinc finger protein 18 (Zinc finger protein KOX11) (Heart development-specific gene 1 protein)(Zinc finger protein with KRAB and SCAN domains 6)
				193	dppalgepppqaplsglfsedeprpfge	Bt_ENSBTAP00000006133	
				330	fdqapgeappQTALSDFFgesephhfg-	Rn_ENSRNOP00000005518	
				330	fcqasgeappQTALSDFFgesephrfg-	Mm_ENSMUSP00000071406	
				-	....	Gg	
				-	....	Xt	
				-	....	Dr	
				-	....	Ce	
				-	....	Dm	
				-	....	Sc	
346	1	CI	ENSP00000342268 ENSG00000078053	317	pkvtptkelqQENIISFFednfvpeisv	Hs_ENSP00000342268	Amphiphysin
				-	....	Bt	
				294	pkvtptkelqQENIINFFednfvpeinv	Rn_ENSRNOP00000017102	
				317	pkvtptkelkQENIINFFednfvpeinv	Mm_ENSMUSP00000003345	
				317	pkltptkelqqeniinlfddnfvpeinv	Gg_ENSGALP00000018798	
				-	....	Xt	
				317	pkvtptrelqqeqiidlfdggfp-eisv	Dr_ENSDARP00000006811	
				-	....	Ce	
				-	....	Dm	
				213	pilfs---leaefvkplfvsfyfmqlni	Sc_YDR388W	
347	1	CI	ENSP00000384421 ENSG00000100109	629	lgelvinp-hQQHMDAFYwvidweg---	Hs_ENSP00000384421	NP_036275 Tuftelin-interacting protein 11 (Septin and tuftelin-interacting protein 1)(STIP-1)
				629	lgelvinp-hQQHMDAFYwvidweg---	Bt_ENSBTAP00000016296	
				629	lgelvinp-hQQHMDAFYwvmdweg---	Rn_ENSRNOP00000000828	
				630	lgelvinp-hQQHMDAFYwvmdweg---	Mm_ENSMUSP00000031288	
				619	lneliinp-hQQHMDAFYwvidweg---	Gg_ENSGALP00000009008	
				569	lsefvinp-hqqhmevfhwvtdweg---	Xt_ENSXETP00000008231	
				622	lgelvvnp-hqqlldpfnwvmdwec---	Dr_ENSDARP00000095416	
				636	ldtmelnptmnpeypewtacmewle---	Ce_CE41502	
				648	lgeliinp-mhqdlelwqqvwewhe---	Dm_FBpp0078765	
				475	lwerhndp--iyeledwkekqewkekds	Sc_YLR424W	
348	1	CI	ENSP00000336701 ENSG00000108384	222	rcrdytellaQVYLLPDFlsehs--kvr	Hs_ENSP00000336701	NP_002867 DNA repair protein RAD51 homolog 3 (R51H3) (RAD51-like protein 2)
				223	rcrdytellaQVYLLSDFlsehs--kvr	Bt_ENSBTAP00000020012	
				213	rchdytellaQVYLLPDFlsdhs--kvq	Rn_ENSRNOP00000008846	
				231	rchdytellaQVYLLPDFlsdhp--kvq	Mm_ENSMUSP00000007790	
				218	rcrdyiellaQVYLLPDFlsehskievr	Gg_ENSGALP00000038538	
				208	schdyiellaQINLLPDFlsshp--kvk	Xt_ENSXETP00000002461	
				205	rchdyvellaevyllpdflsehp--evr	Dr_ENSDARP00000090614	
				-	....	Ce	
				156	rcpkldqlmatvlschrhlvdhp--dik	Dm_FBpp0084486	
				-	....	Sc	
349	1	CI	ENSP00000363345 ENSG00000119457	71	sprgaledqqQRAISNFYiiynlvvgls	Hs_ENSP00000363345	NP_149040 Thymic stromal cotransporter homolog (Solute carrier family 46 member 2)
				71	sprgaledeqQRAISNFYivyhlvmglt	Bt_ENSBTAP00000018168	
				73	slgerqedqqQKAISNFYiiynlvvglt	Rn_ENSRNOP00000023588	

				73<br>-<br>60<br>67<br>-<br>-<br>-	slveyqedqqqkaisnfniiynlvlglt<br>............................<br>deehsrdddlQKAISNFYiiynvvmgmt<br>s------nqaqamssrflliqsvlssvm<br>............................<br>............................<br>............................	Mm_ENSMUSP00000030081<br>Gg<br>Xt_ENSXETP00000004759<br>Dr_ENSDARP00000003366<br>Ce<br>Dm<br>Sc	
350	1	C	ENSP00000309772<br>ENSG00000106034	479<br>298<br>-<br>479<br>480<br>-<br>307<br>-<br>-<br>-	llfpsttpgiQSLMHEFYdvanpvgnpg<br>llfpsttpgfQSLIGEFYgmtspmgkpg<br>............................<br>llfpssvpgirslmgefydmat----pg<br>llypsaapkiqtllrdlyhmvdpmrrlg<br>............................<br>lkkplsaqdyg-----------------<br>............................<br>............................<br>............................	Hs_ENSP00000309772<br>Bt_ENSBTAP00000003820<br>Rn<br>Mm_ENSMUSP00000111041<br>Gg_ENSGALP00000014633<br>Xt<br>Dr_ENSDARP00000067250<br>Ce<br>Dm<br>Sc	NP_001099003 Uncharacterized protein C7orf58 Precursor
351	1	C	ENSP00000264381<br>ENSG00000114200	379<br>379<br>380<br>380<br>379<br>381<br>-<br>-<br>432<br>-	nnsiitrkefQEGLKIFFpgvsefgkes<br>nnsiitrkefQEGLKIFFpgvsefgkes<br>ndslitrrefqeglnmyfpgvsslgkea<br>ndslitrkefqeglnmyfpgvsrlgkea<br>sdslinktqfevaltlsfpqvsklaies<br>hesfinrtqfhksvklafpkatelaids<br>............................<br>............................<br>datalprdkyleimnnifgkatqaerea<br>............................	Hs_ENSP00000264381<br>Bt_ENSBTAP00000014794<br>Rn_ENSRNOP00000013279<br>Mm_ENSMUSP00000029367<br>Gg_ENSGALP00000015414<br>Xt_ENSXETP00000014224<br>Dr<br>Ce<br>Dm_FBpp0082248<br>Sc	NP_000046 Cholinesterase Precursor (EC 3.1.1.8) (Acylcholine acylhydrolase) (Choline esterase II) (Butyrylcholine esterase) (Pseudocholinesterase)
352	1	CI	ENSP00000268482<br>ENSG00000140829	904<br>906<br>904<br>905<br>850<br>-<br>910<br>814<br>906<br>738	vlllkslgv-QDLLQFHFmdpppednml<br>vlllkslgv-QDLLQFHFmdpppednml<br>vlllkslgv-QDLLQFHFmdpppednml<br>vlllkslgv-QDLLQFHFmdpppednml<br>------------liefygsep--afhwl<br>............................<br>vlllkslgv-QDLLLFHFmdpppednml<br>vlllkslgv-ddllkfhfmdappqdnml<br>vlllkslgv-vdllqfhfmdpppqdnil<br>llllksldvtdelskfpfidkpplqtfl	Hs_ENSP00000268482<br>Bt_ENSBTAP00000017640<br>Rn_ENSRNOP00000019810<br>Mm_ENSMUSP00000047865<br>Gg_ENSGALP00000037931<br>Xt<br>Dr_ENSDARP00000070535<br>Ce_CE01027<br>Dm_FBpp0073717<br>Sc_YKR086W	NP_054722 Pre-mRNA-splicing factor ATP-dependent RNA helicase PRP16 (EC 3.6.1.-) (ATP-dependent RNA helicase DHX38)(DEAH box protein 38)
353	1	CI	ENSP00000360340<br>ENSG00000101144	274<br>-<br>273<br>273<br>276<br>267<br>268<br>-<br>299<br>-	ligrhgpqnkQPFMVAFFkatevhfrsi<br>............................<br>ligrhgpqnkQPFMVAFFkatevhlrsi<br>ligrhgpqnkQPFMVAFFkatevhlrsi<br>ligrhgpqnkqpftvaffkatevhlrsi<br>ligtngphnkQPFMVAFFkateihlrsi<br>lvgrsgpqskQPFMVAFFkatevhirsi<br>............................<br>lihrkvddefQPFMIGFFrgpelikata<br>............................	Hs_ENSP00000360340<br>Bt<br>Rn_ENSRNOP00000009656<br>Mm_ENSMUSP00000009143<br>Gg_ENSGALP00000012416<br>Xt_ENSXETP00000024604<br>Dr_ENSDARP00000086646<br>Ce<br>Dm_FBpp0072036<br>Sc	NP_001710 Bone morphogenetic protein 7 Precursor (BMP-7)(Osteogenic protein 1)(OP-1)(Eptotermin alfa)
354	1	CI	ENSP00000326759<br>ENSG00000139263	580<br>580<br>580<br>580<br>559<br>537<br>-<br>593<br>771<br>-	evefasegkyQCVISNHFgssysvkakl<br>nveftsegkyQCVISNHFgssysvkakl<br>nvefasegryQCVISNHFgssysvkarl<br>nveftsegkyQCVISNHFgssysvkakl<br>nvefssegkyQCVISNHFgssysikakl<br>nvefinegkyQCVISNHFgptysvkakl<br>............................<br>nvamtdnseyqcvarnrfgsdfsthvkl<br>nvtyesagryqcvvsnafgttyaqkfki<br>............................	Hs_ENSP00000326759<br>Bt_ENSBTAP00000002883<br>Rn_ENSRNOP00000054820<br>Mm_ENSMUSP00000074360<br>Gg_ENSGALP00000015856<br>Xt_ENSXETP00000046465<br>Dr<br>Ce_CE28415<br>Dm_FBpp0086374<br>Sc	NP_700356 Leucine-rich repeats and immunoglobulin-like domains protein 3 Precursor (LIG-3)
355	1	CI	ENSP00000268489<br>ENSG00000140836	1794<br>1794<br>1799<br>1799<br>1792<br>-<br>-<br>732<br>-<br>-	tetllqlqqqQHLLFPFYipsaefqlnp<br>tetllqlqqqQHLLFPFYipsaefqlnp<br>tetllqlqqqhhllfpfyipsaefqlnp<br>tetllqlqqqQHLLFPFYipsaefqlnp<br>tetllqlqqqQHLLFPFYipsaefqlnp<br>............................<br>............................<br>qqisgilaln-----lhyeeshsskips<br>............................<br>............................	Hs_ENSP00000268489<br>Bt_ENSBTAP00000019489<br>Rn_ENSRNOP00000019408<br>Mm_ENSMUSP00000044612<br>Gg_ENSGALP00000001007<br>Xt<br>Dr<br>Ce_CE28153<br>Dm<br>Sc	NP_008816 Zinc finger homeobox protein 3 (Zinc finger homeodomain protein 3)(ZFH-3)(Alpha-fetoprotein enhancer-binding protein)(AT motif-binding factor)(AT-binding transcription factor 1)
355	2	C	ENSP00000268489<br>ENSG00000140836	2186<br>2186<br>2197	kemadksglpqkvikhwfrntlfkerqr<br>kemadksglpqkvikhwfrntlfkerqr<br>kemadksglpqkvikhwfrntlfkerqr	Hs_ENSP00000268489<br>Bt_ENSBTAP00000019489<br>Rn_ENSRNOP00000019408	NP_008816 Zinc finger homeobox protein 3 (Zinc finger homeodomain protein 3)(ZFH-

				2193<br>2181<br>-<br>-<br>992<br>-<br>-	kemadksglpqkvikhwfrntlfkerqr<br>kemadksglpqkvikhwfrntlfkerqr<br>............................<br>............................<br>kkpepksqtpsssssqlnlqamlsqmqg<br>............................<br>............................	Mm_ENSMUSP00000044612<br>Gg_ENSGALP00000001007<br>Xt<br>Dr<br>Ce_CE28153<br>Dm<br>Sc	3)(Alpha-fetoprotein enhancer-binding protein)(AT motif-binding factor)(AT-binding transcription factor 1)
355	3	C	ENSP00000268489<br>ENSG00000140836	2253<br>2253<br>2264<br>2260<br>2248<br>-<br>-<br>1050<br>-<br>-	rssrtrftdyqlrvlqdffdanaypkdd<br>rssrtrftdyqlrvlqdffdanaypkdd<br>rssrtrftdyqlrvlqdffdanaypkdd<br>rssrtrftdyqlrvlqdffdanaypkdd<br>rssrtrftdyqlrvlqdffdanaypkdd<br>............................<br>............................<br>ranrtrftdfqlrtlqqffdkqaypkdd<br>............................<br>............................	Hs_ENSP00000268489<br>Bt_ENSBTAP00000019489<br>Rn_ENSRNOP00000019408<br>Mm_ENSMUSP00000044612<br>Gg_ENSGALP00000001007<br>Xt<br>Dr<br>Ce_CE28153<br>Dm<br>Sc	NP_008816 Zinc finger homeobox protein 3 (Zinc finger homeodomain protein 3)(ZFH-3)(Alpha-fetoprotein enhancer-binding protein)(AT motif-binding factor)(AT-binding transcription factor 1)
356	1	CI	ENSP00000364403<br>ENSG00000127481	1132<br>1131<br>1133<br>1131<br>1140<br>1110<br>1109<br>0<br>1158<br>-	----aaiskvQVSLDEHFskmaaetdph<br>----aavskvQVSLDEHFskvaaetdpq<br>----aavskvQVSLDEHFskmaaetdph<br>----aavskvQVSLDEHFskmaaetdph<br>----aaiskiQVSLDEHFtklaaetdph<br>----avickvQVSLDEHFsrlstetdpq<br>----aviskvQVSLDEHFskiaadtdpn<br>----------------------------<br>geqsagtsltsssndpelyssnesiddq<br>............................	Hs_ENSP00000364403<br>Bt_ENSBTAP00000020277<br>Rn_ENSRNOP00000032156<br>Mm_ENSMUSP00000095433<br>Gg_ENSGALP00000006213<br>Xt_ENSXETP00000044990<br>Dr_ENSDARP00000034465<br>Ce_CE23594<br>Dm_FBpp0079167<br>Sc	NP_065816 E3 ubiquitin-protein ligase UBR4 (EC 6.3.2.-)(N-recognin-4)(Zinc finger UBR1-type protein 1)(Retinoblastoma-associated factor of 600 kDa)(600 kDa retinoblastoma protein-associated factor)(RBAF600)(p600)
357	1	CI	ENSP00000364404<br>ENSG00000115827	377<br>377<br>377<br>377<br>379<br>-<br>-<br>-<br>-<br>-	klteiennssQHQISEDFvilanrenhk<br>klsevennsaQHQISEDFvilanrernk<br>klsevennssQHQISEDFviwanredrk<br>klsevendssQHQISEDFviwakredrk<br>klkevenntdQIEIAEDFiitanrencv<br>............................<br>............................<br>............................<br>............................<br>............................	Hs_ENSP00000364404<br>Bt_ENSBTAP00000010105<br>Rn_ENSRNOP00000012721<br>Mm_ENSMUSP00000107791<br>Gg_ENSGALP00000015546<br>Xt<br>Dr<br>Ce<br>Dm<br>Sc	NP_079276 Transmembrane protein C2orf37
358	1	CI	ENSP00000316357<br>ENSG00000124486	808<br>808<br>808<br>808<br>808<br>809<br>808<br>-<br>884<br>-	tnlgprlqvnQVVIHEDFiqscfdrlka<br>tnlgprlqvnQVVIHEDFiqscfdrlka<br>tnlgprlqvnQVVIHEDFiqscfdrlka<br>tnlgprlqvnQVVIHEDFiqscfdrlka<br>tnlgprlqvnQVVIHEDFiqscfdrlka<br>tnlgprlqvnQVEIHEDFiqscfdrlka<br>tnlgpklqanQVEIHEDFiqscfdrlka<br>............................<br>talgprlqeniaefhemfigeccsrlrt<br>............................	Hs_ENSP00000316357<br>Bt_ENSBTAP00000047095<br>Rn_ENSRNOP00000004814<br>Mm_ENSMUSP00000111126<br>Gg_ENSGALP00000035868<br>Xt_ENSXETP00000054783<br>Dr_ENSDARP00000026588<br>Ce<br>Dm_FBpp0085202<br>Sc	NP_001034679 Probable ubiquitin carboxyl-terminal hydrolase FAF-X (EC 3.1.2.15)(Ubiquitin thioesterase FAF-X)(Ubiquitin-specific-processing protease FAF-X)(Deubiquitinating enzyme FAF-X)(Fat facets protein-related, X-linked)(Ubiquitin-specific protease 9, X chromosome)
359	1	CI	ENSP00000360757<br>ENSG00000165716	176<br>169<br>176<br>176<br>176<br>152<br>175<br>-<br>-<br>-	dlpslpalvgQVLLMADFnkdnrvslae<br>dlpslpalvgQVLLMADFnkdsrvslae<br>dlpslpalvdQILLMADFnkdsrvslae<br>dlpslpalvdQILLMADFnkdsrvslae<br>dqtslaalvsqiitmadvnrdgkvslae<br>eqvsltalvsqiltmadvnndgkvslae<br>eqsslstlvtrvisladvngdgkvslae<br>............................<br>............................<br>............................	Hs_ENSP00000360757<br>Bt_ENSBTAP00000029233<br>Rn_ENSRNOP00000006186<br>Mm_ENSMUSP00000073860<br>Gg_ENSGALP00000003836<br>Xt_ENSXETP00000048300<br>Dr_ENSDARP00000078196<br>Ce<br>Dm<br>Sc	NP_689634 Protein FAM69B
360	1	C	ENSP00000358513<br>ENSG00000118432	75<br>-<br>76<br>76<br>77<br>66<br>86<br>-<br>-<br>-	npql-vpa-dQVNITEFYnkslssfken<br>............................<br>nspl-vpagdttnitefynkslssfken<br>nspl-vpagdttnitefynkslssfken<br>dpllsiipsdQINITEFYnkslstfken<br>-----ldtldqinatdfynksi--fkdn<br>elivkglpfyptnssdvfgnws---hae<br>............................<br>............................<br>............................	Hs_ENSP00000358513<br>Bt<br>Rn_ENSRNOP00000010850<br>Mm_ENSMUSP00000055797<br>Gg_ENSGALP00000036378<br>Xt_ENSXETP00000011832<br>Dr_ENSDARP00000007647<br>Ce<br>Dm<br>Sc	NP_057167 Cannabinoid receptor 1 (CB1)(CB-R)(CANN6)

361	1	CI	ENSP00000258869 ENSG00000136367	306<br>1363<br>0<br>1355<br>-<br>211<br>-<br>-<br>-<br>-	esllklq--qQQLLLPFYlhdlkvgpkl<br>esllklq--qQQLLLPFYlhdlkvgpkl<br>----------------------------<br>esllklq--qQQLLLPFYlhdlkvgpkl<br>............................<br>eallklqrqqQQLLLPFYlqelkvs---<br>............................<br>............................<br>............................<br>............................	Hs_ENSP00000258869<br>Bt_ENSBTAP00000009273<br>Rn_ENSRNOP00000034719<br>Mm_ENSMUSP00000045156<br>Gg<br>Xt_ENSXETP00000048837<br>Dr<br>Ce<br>Dm<br>Sc	Zinc finger homeobox protein 2 (Zinc finger homeodomain protein 2)(ZFH-2)
362	1	CI	ENSP00000296513 ENSG00000164113	398<br>-<br>440<br>440<br>323<br>376<br>-<br>-<br>-<br>-	kltrwevlgvQGALLSHFiqpvyissil<br>............................<br>kltrwevlgvQGALLSHFiqpvyissil<br>kltrwevlgvQGALLSHFiqpvyissil<br>kltkwevvgvqgallsyfiepvyintil<br>tclrlklt---cstlnffvas-filfyv<br>............................<br>............................<br>............................<br>............................	Hs_ENSP00000296513<br>Bt<br>Rn_ENSRNOP00000023282<br>Mm_ENSMUSP00000029274<br>Gg_ENSGALP00000019325<br>Xt_ENSXETP00000003358<br>Dr<br>Ce<br>Dm<br>Sc	NP_640336 Adenosine deaminase domain-containing protein 1 (Testis nuclear RNA-binding protein)
363	1	C	ENSP00000296596 ENSG00000164181	37<br>37<br>37<br>37<br>37<br>75<br>37<br>-<br>35<br>-	dwllmssplpQTILLGFYvyfvtslgpk<br>dwllmssplpQTIILGFYvyfvtslgpk<br>nwllmssplpqtiilglyvyfvtslgpk<br>dyllmssplpqtiilglyvyfvtslgpk<br>gwplmsspfpttfiigtyiyfvtslgpk<br>dwplmstpipqtiiigayiyfvtslgpr<br>gwllmsnpipQMLIIVFYiyfvislgpk<br>............................<br>dyplmsspfptiaisltyayivkvlgpk<br>............................	Hs_ENSP00000296596<br>Bt_ENSBTAP00000010129<br>Rn_ENSRNOP00000014074<br>Mm_ENSMUSP00000022207<br>Gg_ENSGALP00000023707<br>Xt_ENSXETP00000021127<br>Dr_ENSDARP00000091465<br>Ce<br>Dm_FBpp0081313<br>Sc	NP_001098028 Elongation of very long chain fatty acids protein 7
364	1	CI	ENSP00000379228 ENSG00000002746	1355<br>1352<br>1330<br>1353<br>1352<br>1079<br>1300<br>624<br>-<br>-	grilglalihQYLLDAFFtrpfykallr<br>grilglalihQYLLDAFFtrpfykallr<br>grilglalihQYLLDAFFtrpfykgllk<br>grilglalihQYLLDAFFtrpfykgllk<br>grilglalihQYLLDAFFtrpfykallr<br>grilglalihQYLLDAFFtrpfykallr<br>grilglalihQYLLDAFFtrpfykallr<br>grvlalavihrcyidvfftnvfykslqk<br>............................<br>............................	Hs_ENSP00000379228<br>Bt_ENSBTAP00000028268<br>Rn_ENSRNOP00000021703<br>Mm_ENSMUSP00000048972<br>Gg_ENSGALP00000020143<br>Xt_ENSXETP00000034467<br>Dr_ENSDARP00000031155<br>Ce_CE01588<br>Dm<br>Sc	NP_055867 E3 ubiquitin-protein ligase HECW1 (EC 6.3.2.-)(HECT, C2 and WW domain-containing protein 1)(NEDD4-like E3 ubiquitin-protein ligase 1)(hNEDL1)
365	1	CI	ENSP00000340736 ENSG00000163754	164<br>164<br>162<br>200<br>164<br>164<br>164<br>163<br>-<br>189	seqgsfdggdQGILNTFFs---swattd<br>seqgsfdggdQGLLNTFFs---swattd<br>seqgsfdggdqgllntyfs---gwattd<br>seqgsfdggdqgllntyfs---gwattd<br>tekgsfdgadQGLLNTFFs---swattd<br>tqkgsfdggdQGLLNTFFd---twatkd<br>seggsfdggdQGVLNSFFs---dwatad<br>vthgsydggdQGLLNDFFs---nwrdlp<br>............................<br>iktvsidgadqgifnqffnpicnyskev	Hs_ENSP00000340736<br>Bt_ENSBTAP00000002261<br>Rn_ENSRNOP00000014837<br>Mm_ENSMUSP00000019672<br>Gg_ENSGALP00000016983<br>Xt_ENSXETP00000035050<br>Dr_ENSDARP00000011595<br>Ce_CE04664<br>Dm<br>Sc_YJL137C	NP_004121 Glycogenin-1 (EC 2.4.1.186)
366	1	CI	ENSP00000320794 ENSG00000176542	1563<br>1197<br>1541<br>1543<br>1491<br>-<br>-<br>-<br>-<br>-	gqphphhqqmQQQMQQHFgssqtekscе<br>gqphphhqqmQQQMQQHFgssqaeksce<br>sqphphhqqmQQQLQQHFassqpeksce<br>sqphphhqqmQQQLQQHFassqpeksce<br>gqphphhqqmQQQMQQHFgasqpeknce<br>............................<br>............................<br>............................<br>............................<br>............................	Hs_ENSP00000320794<br>Bt_ENSBTAP00000027966<br>Rn_ENSRNOP00000040722<br>Mm_ENSMUSP00000112620<br>Gg_ENSGALP00000023862<br>Xt<br>Dr<br>Ce<br>Dm<br>Sc	NP_001009899 Basic helix-loop-helix domain-containing protein KIAA2018
366	2	CI	ENSP00000320794 ENSG00000176542	2104<br>1689<br>2082<br>2080<br>2034<br>-<br>-<br>-<br>-<br>-	rtpalipvdpQNTLPSFYppyspahptl<br>rtpalipvdpQNTLPSFYppyspahptl<br>rapalipvdpQNTLPSFYppyspahptl<br>rapalipvdpQNTLPSFYppyspahptl<br>rtpalipvdpQNTLPSFYppyspahptl<br>............................<br>............................<br>............................<br>............................<br>............................	Hs_ENSP00000320794<br>Bt_ENSBTAP00000027966<br>Rn_ENSRNOP00000040722<br>Mm_ENSMUSP00000112620<br>Gg_ENSGALP00000023862<br>Xt<br>Dr<br>Ce<br>Dm<br>Sc	NP_001009899 Basic helix-loop-helix domain-containing protein KIAA2018

367	1	CI	ENSP00000328174<br>ENSG00000140451	337<br>349<br>340<br>346<br>319<br>338<br>337<br>378<br>335<br>372	rrqqnkpfggiQLIICGDFlqlppvtk--<br>rqqnkpfggiQLIICGDFlqlppvtk--<br>rqqkkpfggiQLIICGDFlqlppvtk--<br>rqqkkpfggiQLIICGDFlqlppvtk--<br>rkrdepfggiQLIICGDFlqlppvck--<br>rgkdepfggiqlivcgdflqlppvtq--<br>rrstepfggiqlivcgdflqlppvtk--<br>rnndkpfggiQLIITGDFfqlppvsk--<br>rrndrpfggiQLILCGDFlqlppvikgd<br>rknhqpfggiqlifcgdffqlppvskd-	Hs_ENSP00000328174<br>Bt_ENSBTAP00000010047<br>Rn_ENSRNOP00000021265<br>Mm_ENSMUSP00000049046<br>Gg_ENSGALP00000014682<br>Xt_ENSXETP00000021069<br>Dr_ENSDARP00000022087<br>Ce_CE30311<br>Dm_FBpp0077258<br>Sc_YML061C	ATP-dependent DNA helicase PIF1 (EC 3.6.1.-)(PIF1/RRM3 DNA helicase-like protein)
368	1	CI	ENSP00000376634<br>ENSG00000198420	879<br>878<br>879<br>881<br>-<br>-<br>-<br>-<br>-<br>-	lwvkmfshqvQKNLAPFFeawawpiqke<br>lwvkmfshqvQKNLAPFFeawawpiqke<br>lwvkmfsqqvQKNLAPFFeawawpiqke<br>lwvkmfsqqvQRNLAPFFeawgwpiqke<br>............................<br>............................<br>............................<br>............................<br>............................<br>............................	Hs_ENSP00000376634<br>Bt_ENSBTAP00000050827<br>Rn_ENSRNOP00000024428<br>Mm_ENSMUSP00000046137<br>Gg<br>Xt<br>Dr<br>Ce<br>Dm<br>Sc	NP_055534 Protein FAM115A
369	1	CI	ENSP00000297205<br>ENSG00000164647	163<br>163<br>163<br>163<br>-<br>-<br>-<br>-<br>-<br>-	wldkwmltrkQFGLLSFFfavlhaiysl<br>wldrwmvtrkQFGLLSFFfavlhaiysl<br>wldrwmlarkQFGLLSFFfavlhaiysl<br>wldrwmlarkQFGLLSFFfavlhavysl<br>............................<br>............................<br>............................<br>............................<br>............................<br>............................	Hs_ENSP00000297205<br>Bt_ENSBTAP00000020911<br>Rn_ENSRNOP00000000018<br>Mm_ENSMUSP00000015796<br>Gg<br>Xt<br>Dr<br>Ce<br>Dm<br>Sc	NP_036581 Metalloreductase STEAP1 (EC 1.16.1.-)(Six-transmembrane epithelial antigen of prostate 1)
370	1	CI	ENSP00000198939<br>ENSG00000085872	710<br>710<br>718<br>719<br>698<br>719<br>643<br>507<br>715<br>-	drprnsegweQNGLYEFFrakmrarrrk<br>drprnsegweQNGLYEFFrakmrarrrk<br>drprnsegweQNGLYEFFrakmrarrrk<br>drprnsegweQNGLYEFFrakmrarrrk<br>drprnsegweQNGLYEFFrakmrarrrk<br>drprnsegweQNGLYEFFrakmrarrrk<br>----------------------------<br>p--------------enlredsearrsn<br>drprdnegweklglyeyykvknaarkqk<br>............................	Hs_ENSP00000198939<br>Bt_ENSBTAP00000006959<br>Rn_ENSRNOP00000017424<br>Mm_ENSMUSP00000078469<br>Gg_ENSGALP00000006050<br>Xt_ENSXETP00000004447<br>Dr_ENSDARP00000030934<br>Ce_CE28038<br>Dm_FBpp0075048<br>Sc	NP_006378 Calcium homeostasis endoplasmic reticulum protein (SR-related CTD associated factor 6)(ERPROT 213-21)
371	1	C	ENSP00000266546<br>ENSG00000139182	367<br>372<br>367<br>367<br>375<br>300<br>-<br>-<br>-<br>-	ggpsglgsgpQDSLSDHFtlsfwmkhg-<br>ggaaglgsgppdslsdhftlsfwmkhg-<br>ggpaglgsgpQDGLSDHFtlsfwmkhs-<br>ggpaglgsgpqdgfsdhftlsfwmkhs-<br>mn----glnaheglsdhftlsvwmkha-<br>sh-----lpaggrtadhltlsfwlkhag<br>............................<br>............................<br>............................<br>............................	Hs_ENSP00000266546<br>Bt_ENSBTAP00000010687<br>Rn_ENSRNOP00000015570<br>Mm_ENSMUSP00000008297<br>Gg_ENSGALP00000023600<br>Xt_ENSXETP00000051586<br>Dr<br>Ce<br>Dm<br>Sc	NP_055533 Calsyntenin-3 Precursor (Alcadein-beta)(Alc-beta)
372	1	CI	ENSP00000366641<br>ENSG00000142583	206<br>206<br>204<br>205<br>209<br>210<br>-<br>-<br>-<br>-	lgltgvpaalQLLLLPFFpesprylliq<br>lgltgipavlqllflpffpesprylliq<br>lgltgvpaglQLLLLPFFpesprylliq<br>lgltgvpaglQLLLLPFFpesprylliq<br>lgltgipsalqlltlpffpesprylliq<br>laltgipavlelaflpffpesprytllh<br>............................<br>............................<br>............................<br>............................	Hs_ENSP00000366641<br>Bt_ENSBTAP00000009606<br>Rn_ENSRNOP00000024054<br>Mm_ENSMUSP00000030826<br>Gg_ENSGALP00000003884<br>Xt_ENSXETP00000026017<br>Dr<br>Ce<br>Dm<br>Sc	NP_003030 Solute carrier family 2, facilitated glucose transporter member 5 (Glucose transporter type 5, small intestine)(GLUT-5)(Fructose transporter)
373	1	C	ENSP00000283195<br>ENSG00000153201	571<br>571<br>-<br>-<br>574<br>570<br>-<br>-<br>640<br>-	tlraqekhglqpallvhwaeclqktg--<br>tlrgqektglqpallvhwakclqktg--<br>............................<br>............................<br>tlralekhglqpaliihwarslqktg--<br>tlraqekhglqpavlvhwarslhkkg--<br>............................<br>............................<br>avrgvngpkadaiiifqlgkilnsrsdr<br>............................	Hs_ENSP00000283195<br>Bt_ENSBTAP00000042908<br>Rn<br>Mm<br>Gg_ENSGALP00000004070<br>Xt_ENSXETP00000034934<br>Dr<br>Ce<br>Dm_FBpp0084188<br>Sc	NP_006258 E3 SUMO-protein ligase RanBP2 (Ran-binding protein 2)(Nuclear pore complex protein Nup358)(Nucleoporin Nup358)(358 kDa nucleoporin)(p270)

374	1	CI	ENSP00000304051<br>ENSG00000170881	73<br>73<br>-<br>73<br>14<br>67<br>67<br>-<br>145<br>-	fassivlilsQRSLFKFYtyssafllaa<br>fvssivlilsQRSLFKFYmyssafllaa<br>............................<br>vvssivlilsQRSLFKFYmyssafllaa<br>fvssivlvlqQRALFKFYmiasafllaa<br>vasivvlvlsrralfkfymitaalalaa<br>vvsgvvlvlsQKSLFKFYtlfmavllga<br>............................<br>lsaacifmlwtrhlvmvymfltslgltf<br>............................	Hs_ENSP00000304051<br>Bt_ENSBTAP00000027187<br>Rn<br>Mm_ENSMUSP00000046467<br>Gg_ENSGALP00000026304<br>Xt_ENSXETP00000037995<br>Dr_ENSDARP00000053621<br>Ce<br>Dm_FBpp0089379<br>Sc	NP_009149 RING finger protein 139 (Translocation in renal carcinoma on chromosome 8)
375	1	C	ENSP00000229134<br>ENSG00000111536	82<br>-<br>-<br>-<br>82<br>-<br>-<br>-<br>-<br>-	kkqfmkncqfQEQLLSFFmedvfgqlql<br>............................<br>............................<br>............................<br>kmlfmtncnvrdqllsfymknvfshlgm<br>............................<br>............................<br>............................<br>............................<br>............................	Hs_ENSP00000229134<br>Bt<br>Rn<br>Mm<br>Gg_ENSGALP00000034723<br>Xt<br>Dr<br>Ce<br>Dm<br>Sc	NP_060872 Interleukin-26 Precursor (AK155 protein)
376	1	CI	ENSP00000290551<br>ENSG00000159388	43<br>45<br>43<br>43<br>47<br>-<br>43<br>-<br>-<br>-	qrlkvfsgalQEALTEHYkhhwfpekps<br>qrlqvfrgalQAALTEHYkhhwfpekps<br>qrlkvfsralQDALTDHYkhhwfpekps<br>qrlkvfsralQDALTDHYkhhwfpekps<br>qqlqvfsgalrealaehykhhwfpekpf<br>............................<br>aqlqvfrdglaqalsehyqhhwfpdrpq<br>............................<br>............................<br>............................	Hs_ENSP00000290551<br>Bt_ENSBTAP00000020860<br>Rn_ENSRNOP00000004408<br>Mm_ENSMUSP00000020692<br>Gg_ENSGALP00000005580<br>Xt<br>Dr_ENSDARP00000006800<br>Ce<br>Dm<br>Sc	NP_006754 Protein BTG2 (BTG family member 2)(NGF-inducible anti-proliferative protein PC3)
377	1	CI	ENSP00000229135<br>ENSG00000111537	69<br>69<br>68<br>67<br>72<br>-<br>77<br>-<br>-<br>-	---esdrkimQSQIVSFYfklfknfkdd<br>---esdkkiiQSQIVSFYfklfenlkdn<br>---dgntkilesqiisfylrlfevlkdn<br>---dgdmkilQSQIISFYlrlfevlkdn<br>---rnekriilsqivsmylemlentdks<br>............................<br>nleeseqnllmsivmdtysriftrmqnd<br>............................<br>............................<br>............................	Hs_ENSP00000229135<br>Bt_ENSBTAP00000016634<br>Rn_ENSRNOP00000009917<br>Mm_ENSMUSP00000063800<br>Gg_ENSGALP00000016086<br>Xt<br>Dr_ENSDARP00000030124<br>Ce<br>Dm<br>Sc	NP_000610 Interferon gamma Precursor (IFN-gamma) (Immune interferon)
378	1	C	ENSP00000221307<br>ENSG00000186529	238<br>238<br>238<br>238<br>-<br>-<br>223<br>-<br>-<br>-	lsalvtkrhqQILLYIDFlyyltpdgqr<br>lsalvakryqQIFLHMDFlyyltpdgwr<br>lsalvarrhqslllyvdlfyhltrdgmr<br>lstlvarrhqrlllhvdlfyylthdgmr<br>............................<br>............................<br>lsrllvqrqhylpyhwdwlywrsaqgrr<br>............................<br>............................<br>............................	Hs_ENSP00000221307<br>Bt_ENSBTAP00000036243<br>Rn_ENSRNOP00000021609<br>Mm_ENSMUSP00000003574<br>Gg<br>Xt<br>Dr_ENSDARP00000063441<br>Ce<br>Dm<br>Sc	NP_000887 Cytochrome P450 4F3 (EC 1.14.13.30)(CYPIVF3)(Leukotriene-B(4) omega-hydroxylase)(Leukotriene-B(4) 20-monooxygenase)(Cytochrome P450-LTB-omega)
379	1	CI	ENSP00000352516<br>ENSG00000130816	180<br>164<br>161<br>169<br>-<br>152<br>160<br>-<br>-<br>-	spritrkstrQTTITSHFakgpakrkpq<br>spritrkttrQTTITSHFprgpakrkpe<br>ssvatrrttrQTTITSHF-kgpakrkpk<br>ssvatrrttrQTTITAHFtkgptkrkpk<br>............................<br>arssrsttgkqatilsmfskgsnkrkss<br>srvtr-ntgkqqtivsmfsr-vpkrks-<br>............................<br>............................<br>............................	Hs_ENSP00000352516<br>Bt_ENSBTAP00000003549<br>Rn_ENSRNOP00000057906<br>Mm_ENSMUSP00000004202<br>Gg<br>Xt_ENSXETP00000047703<br>Dr_ENSDARP00000013243<br>Ce<br>Dm<br>Sc	NP_001124295 DNA (cytosine-5)-methyltransferase 1 (Dnmt1)(EC 2.1.1.37)(MCMT)(DNA methyltransferase HsaI)(DNA MTase HsaI)(M.HsaI)(CXXC-type zinc finger protein 9)
380	1	CI	ENSP00000363680<br>ENSG00000158813	132<br>0<br>132<br>132<br>96<br>-<br>-<br>-<br>-<br>-	alhsdsqdghQMALLNFFfpdekpysee<br>----------------------------<br>plppdsqdrhQMALLNFFfpdekpysee<br>plppdsqdrhQMALLNFFfpdekaysee<br>gvvvp-----elallnffhpeeklhvge<br>............................<br>............................<br>............................<br>............................<br>............................	Hs_ENSP00000363680<br>Bt_ENSBTAP00000016649<br>Rn_ENSRNOP00000004505<br>Mm_ENSMUSP00000109409<br>Gg_ENSGALP00000007125<br>Xt<br>Dr<br>Ce<br>Dm<br>Sc	NP_001390 Ectodysplasin-A (Ectodermal dysplasia protein) (EDA protein)

381	1	CI	ENSP00000357307 ENSG00000132718	121	rgpssgscidQLPIKMDYg---eelrsp	Hs_ENSP00000357307	NP_689493 Synaptotagmin-11 (Synaptotagmin XI)(SytXI)
				121	kgpssgscvdQLPIKVDYg---eelrsp	Bt_ENSBTAP00000014885	
				120	rgpspascidQLPIKRDYg---eelrsp	Rn_ENSRNOP00000027474	
				120	rgpspascmdQLPIKRDYg---eelrsp	Mm_ENSMUSP00000103129	
				110	dgpntaphidQLPIKVDYg---del-sp	Gg_ENSGALP00000023561	
				-	....	Xt	
				146	sghevsrlerelpvradyccldsssass	Dr_ENSDARP00000073004	
				-	....	Ce	
				-	....	Dm	
				-	....	Sc	
382	1	CI	ENSP00000322915 ENSG00000146463	131	ene----iqiQNKLKKDFpkqfdqvsvf	Hs_ENSP00000322915	NP_005086 Zinc finger MYM-type protein 4 (Zinc finger protein 262)
				102	ene----iqiQNKLKKDFpkqfdqvsvf	Bt_ENSBTAP00000027998	
				76	ene----iqiQSQLKKDFpkqfdqvsvf	Rn_ENSRNOP00000016992	
				130	ene----iqiQNQLKKDFpkqfdqvsvf	Mm_ENSMUSP00000101714	
				117	ekd----iqienaiqkdltspfeqgpvf	Gg_ENSGALP00000003928	
				0	----------------------------	Xt_ENSXETP00000021311	
				52	ehe----srttknqk-------------	Dr_ENSDARP00000002457	
				-	....	Ce	
				128	edeqpqteavrvdpqeepeeaeeetdal	Dm_FBpp0111770	
				-	....	Sc	
383	1	CI	ENSP00000233573 ENSG00000115232	204	kkfgenfascQAGISSFYtkdlivmgap	Hs_ENSP00000233573	Integrin alpha-4 Precursor (Integrin alpha-IV)(VLA-4)(CD49 antigen-like family member D)(CD49d antigen)
				199	kqfgenfascQAGISSFYtedlivmgap	Bt_ENSBTAP00000012194	
				209	rkfgenfascQAGISSFYtqdlivmgap	Rn_ENSRNOP00000006880	
				205	rkfgenfascQAGISSFYtqdlivmgap	Mm_ENSMUSP00000028395	
				197	rkfgenhgscQAGMSSFYigdliimgap	Gg_ENSGALP00000014591	
				199	rkfgdsygscQAGISTFYvedvivmgap	Xt_ENSXETP00000030728	
				-	....	Dr	
				-	....	Ce	
				-	....	Dm	
				-	....	Sc	
384	1	CI	ENSP00000316284 ENSG00000181733	159	wvvgvlnasiQTSITLHFpycasrivdh	Hs_ENSP00000316284	NP_001004699 Olfactory receptor 2Z1 (Olfactory receptor OR19-4)
				159	wlagvlnaciQTSITLHFpycashtvdh	Bt_ENSBTAP00000050025	
				159	wlagvlnaliqtsitlnfsycasrivdh	Rn_ENSRNOP00000019271	
				159	wfsgalvasiltsitlqfpycashtvdh	Mm_ENSMUSP00000074128	
				-	....	Gg	
				-	....	Xt	
				-	....	Dr	
				-	....	Ce	
				-	....	Dm	
				-	....	Sc	
385	1	CI	ENSP00000258301 ENSG00000135823	197	sqriggeleeQAVMLEDFshelestqsr	Hs_ENSP00000258301	NP_005810 Syntaxin-6
				197	sqriggeleeQAVMLDDFshelestqsr	Bt_ENSBTAP00000027262	
				197	sqriggeleeQAVMLDDFshelestqsr	Rn_ENSRNOP00000037536	
				197	sqriggeleeQAVMLDDFshelestqsr	Mm_ENSMUSP00000107409	
				197	sqriggeleeQAVMLDDFsheldsthsr	Gg_ENSGALP00000006160	
				199	sqrigseldeQAVMLDDFsheldtaqsr	Xt_ENSXETP00000050036	
				198	sqrigqeldeQAVMLDDFshemdstqsr	Dr_ENSDARP00000062726	
				-	....	Ce	
				-	....	Dm	
				-	....	Sc	
386	1	CI	ENSP00000360235 ENSG00000177853	14	eqkqlfcdekQTTLKKDYdvkneivd--	Hs_ENSP00000360235	NP_055618 Zinc finger protein 518A
				14	eqkhlffdekQNTLKKDYdvknevvdsl	Bt_ENSBTAP00000049132	
				14	eqtqlfcdekqtilkk-idtrneipdt-	Rn_ENSRNOP00000051600	
				14	eqtqlfcdekptilkk-ldtrneiadt-	Mm_ENSMUSP00000055956	
				0	----------------------------	Gg_ENSGALP00000011270	
				0	----------------------------	Xt_ENSXETP00000026591	
				-	....	Dr	
				-	....	Ce	
				-	....	Dm	
				-	....	Sc	
387	1	CI	ENSP00000358400 ENSG00000112159	935	kglsvnkntvQG-IINFYtalrkesg-t	Hs_ENSP00000358400	NP_055426 Midasin (MIDAS-containing protein)
				936	kglsvnkntvQG-IITFYtavrkesg-t	Bt_ENSBTAP00000002157	
				931	kglsvsrsavQG-IVNFYtalrkesk-t	Rn_ENSRNOP00000037995	
				931	kglnvskntvQG-IVNFYtalrkesg-t	Mm_ENSMUSP00000071569	
				933	rglnvsknptvQG-IVNFYlavrkeae-t	Gg_ENSGALP00000025327	
				-	....	Xt	
				-	....	Dr	
				618	p--tvsqqfven-lvkfylsakqlyp--	Ce_CE30377	
				1005	antgiqrksvhn-ivqlykslrklse-l	Dm_FBpp0111985	
				930	gkysvsdewvgndiaelyleakklsdnn	Sc_YLR106C	

387	2	CI	ENSP00000358400<br>ENSG00000112159	1320<br>1321<br>1316<br>1316<br>1318<br>-<br>-<br>960<br>1386<br>1310	vrkqeeidviQEVLEKHFkkklcpqslf<br>vrkqeevvviQEVLEKHFkkklcpqslf<br>vrkqeeadviQEVLEKHFkkklcpqslf<br>vrkqeeadviQEVLEKHFkkklcpqslf<br>vrkqeevdviQRVIEKHFkkriypeslf<br>............................<br>............................<br>crnekddvtvvetlekvikrkidkealf<br>vrsqeeheliektlfanfrkkldlqtlf<br>crtpqekvtvkktlekvmkvkldmdqyy	Hs_ENSP00000358400<br>Bt_ENSBTAP00000002157<br>Rn_ENSRNOP00000037995<br>Mm_ENSMUSP00000071569<br>Gg_ENSGALP00000025327<br>Xt<br>Dr<br>Ce_CE30377<br>Dm_FBpp0111985<br>Sc_YLR106C	NP_055426 Midasin (MIDAS-containing protein)
387	3	C	ENSP00000358400<br>ENSG00000112159	2584<br>2585<br>2579<br>2579<br>2589<br>-<br>-<br>2113<br>2655<br>2498	gavsnvfkilqpnttdefvipldprwnm<br>sgvsnvikilqpnvtdesmipldprwnm<br>agvsnvlkilQTNITDDFvipwdprwnm<br>agvsnvlkilQTNITDDFvipldprwnm<br>sgisnmikllqptmtdehvmpldprwnv<br>............................<br>............................<br>tlvrsiietfktspsistnayrevcisi<br>elsqnlynclqkrsntdtgtlanlpwnr<br>hgkideltyielsaaafngrhlknipri	Hs_ENSP00000358400<br>Bt_ENSBTAP00000002157<br>Rn_ENSRNOP00000037995<br>Mm_ENSMUSP00000071569<br>Gg_ENSGALP00000025327<br>Xt<br>Dr<br>Ce_CE30377<br>Dm_FBpp0111985<br>Sc_YLR106C	NP_055426 Midasin (MIDAS-containing protein)
387	4	C	ENSP00000358400<br>ENSG00000112159	2942<br>2943<br>2936<br>2936<br>2946<br>-<br>-<br>2372<br>3009<br>2778	pameylamlwrykvtadfmaqa-clrrc<br>pvmeylavlwqykvtadfmtqa-clrrs<br>pvteylavlwqyrvtagfvtqa-clrrn<br>paaeylamlwqyrvtadfiaqa-clr--<br>pamecmgvlwQYKLTADYvaka-yarre<br>............................<br>............................<br>ptmsnlsiahlqkfpikelgtklwrlsv<br>tntpklliaalrelfmerlltg-tlrdd<br>kltsftstifssqfledvvrksnnlksf	Hs_ENSP00000358400<br>Bt_ENSBTAP00000002157<br>Rn_ENSRNOP00000037995<br>Mm_ENSMUSP00000071569<br>Gg_ENSGALP00000025327<br>Xt<br>Dr<br>Ce_CE30377<br>Dm_FBpp0111985<br>Sc_YLR106C	NP_055426 Midasin (MIDAS-containing protein)
388	1	CI	ENSP00000306296<br>ENSG00000106443	612<br>607<br>604<br>605<br>566<br>561<br>564<br>418<br>421<br>-	--------hkQPALTADFvnyyfernmr<br>--------hkQPALTADFvnyyfernmr<br>--------hkQPALTADFvnyyfernmr<br>--------hkQPALTADFvnyyfernmr<br>--------hkQPALTADFvnyylernmr<br>--------hkQPALSADFvnyylernmr<br>--------hkQPALTADFvnyylernmr<br>skeyielftktneifsgissfmqirdsq<br>--------hiapafsveftayymdrivr<br>............................	Hs_ENSP00000306296<br>Bt_ENSBTAP00000015687<br>Rn_ENSRNOP00000007707<br>Mm_ENSMUSP00000111173<br>Gg_ENSGALP00000017413<br>Xt_ENSXETP00000048143<br>Dr_ENSDARP00000082096<br>Ce_CE26433<br>Dm_FBpp0077145<br>Sc	NP_001007158 PHD finger protein 14
389	1	CI	ENSP00000368809<br>ENSG00000135473	889<br>633<br>889<br>888<br>884<br>888<br>-<br>829<br>931<br>820	egvth----qQWYLFNDFliepidkhea<br>qgvth----qQWYLFNDFliepidkhea<br>egvth----qQWYLFNDFliepidkyea<br>egvth----qQWYLFNDFliepidkyea<br>egvth----qQWYLFNDFliepvdkcea<br>egvth----qQWYLFNDFliepvdkcea<br>............................<br>---------sswtlineqlvsrlhdhea<br>laesaddpqsQWYIFNDFsispvspqes<br>--------cfkwlmfndylvveiteeea	Hs_ENSP00000368809<br>Bt_ENSBTAP00000005740<br>Rn_ENSRNOP00000039314<br>Mm_ENSMUSP00000100871<br>Gg_ENSGALP00000004379<br>Xt_ENSXETP00000020788<br>Dr<br>Ce_CE01269<br>Dm_FBpp0087735<br>Sc_YGL094C	NP_001120932 PAB-dependent poly(A)-specific ribonuclease subunit 2 (hPan2)(EC 3.1.13.4) (Inactive ubiquitin carboxyl-terminal hydrolase 52)
390	1	CI	ENSP00000317790<br>ENSG00000137877	871<br>1020<br>838<br>-<br>832<br>-<br>-<br>1013<br>1004<br>-	dfdpntilqtQDHLSQDYeslralaqlr<br>gfdpntilqtQDRLNQDYeglraqaerr<br>hfdsnailqaqadlrqnyenlralaklh<br>............................<br>hfvpeniwktqdeidslyenlqsmaedr<br>............................<br>............................<br>etkssvvadrQHKISNDYrelkrladvr<br>nenadsvekrqqrinqtydelqemaqkr<br>............................	Hs_ENSP00000317790<br>Bt_ENSBTAP00000008931<br>Rn_ENSRNOP00000053108<br>Mm<br>Gg_ENSGALP00000014517<br>Xt<br>Dr<br>Ce_CE41581<br>Dm_FBpp0289294<br>Sc	NP_057726 Spectrin beta chain, brain 4 (Spectrin, non-erythroid beta chain 4)(Beta-V spectrin) (BSPECV)
390	2	C	ENSP00000317790<br>ENSG00000137877	1672<br>1816<br>1627<br>-<br>1638<br>-<br>-<br>1815<br>1810<br>-	lrlinkhqalqeelaiywssmeeldqta<br>irlikkhqalqqelarcwnsveeldrra<br>fglirkhqmlrqeialcwgsmedleqrf<br>............................<br>lkllqkhkvleheiavyqdlikelnesv<br>............................<br>............................<br>rkllskhralcedmttyrqwleklevkc<br>aklltkhktieleldtysgivtemghsc<br>............................	Hs_ENSP00000317790<br>Bt_ENSBTAP00000008931<br>Rn_ENSRNOP00000053108<br>Mm<br>Gg_ENSGALP00000014517<br>Xt<br>Dr<br>Ce_CE41581<br>Dm_FBpp0289294<br>Sc	NP_057726 Spectrin beta chain, brain 4 (Spectrin, non-erythroid beta chain 4)(Beta-V spectrin) (BSPECV)

390	3	C	ENSP00000317790<br>ENSG00000137877	2446<br>2591<br>2393<br>-<br>2426<br>-<br>-<br>2595<br>2594<br>-	-aahglrhrqqevaeswwqlrsraqkrr<br>-vahglshqqqemmdswrqlqsriqkwk<br>-atgglrhkqqemmdswwkvwhkarkwr<br>............................<br>-indkltmkqqemknnwlrlqgqakqrk<br>............................<br>............................<br>-lretvldslkkleesweqlskaaelrn<br>rgaqhierkleelhkswgnlqalsvkrq<br>............................	Hs_ENSP00000317790<br>Bt_ENSBTAP00000008931<br>Rn_ENSRNOP00000053108<br>Mm<br>Gg_ENSGALP00000014517<br>Xt<br>Dr<br>Ce_CE41581<br>Dm_FBpp0289294<br>Sc	NP_057726 Spectrin beta chain, brain 4 (Spectrin, non-erythroid beta chain 4)(Beta-V spectrin) (BSPECV)
391	1	CI	ENSP00000313280<br>ENSG00000175841	129<br>132<br>-<br>-<br>-<br>-<br>-<br>-<br>-<br>-	lqhgsqipciQMALQAHYdvivlnpndn<br>lqhgsqipciQMALQAHYdvivlnpndn<br>............................<br>............................<br>............................<br>............................<br>............................<br>............................<br>............................<br>............................	Hs_ENSP00000313280<br>Bt_ENSBTAP00000048190<br>Rn<br>Mm<br>Gg<br>Xt<br>Dr<br>Ce<br>Dm<br>Sc	Putative UPF0528 protein FAM172B
392	1	CI	ENSP00000354719<br>ENSG00000184209	124<br>119<br>119<br>119<br>124<br>-<br>118<br>-<br>-<br>-	yrdadglvidQHEIFVDYelertlkgwi<br>yrdadglvidQHEIFVDYelertlkgwi<br>yrdadglvidQHEIFVDYelertlrgwi<br>yrdadglvidQHEIFVDYelertlrgwi<br>hrdanrlvidqhevfvdfelertlkgwi<br>............................<br>wrdankliidqyellvdveqertlpgwr<br>............................<br>............................<br>............................	Hs_ENSP00000354719<br>Bt_ENSBTAP00000043747<br>Rn_ENSRNOP00000001402<br>Mm_ENSMUSP00000031349<br>Gg_ENSGALP00000005248<br>Xt<br>Dr_ENSDARP00000065242<br>Ce<br>Dm<br>Sc	NP_851034 U11/U12 small nuclear ribonucleoprotein 35 kDa protein (U11/U12 snRNP 35 kDa protein)(U11/U12-35K)(U1 snRNP-binding protein homolog)(Protein HM-1)
393	1	CI	ENSP00000344314<br>ENSG00000046651	354<br>355<br>354<br>356<br>344<br>351<br>349<br>-<br>-<br>-	rklknellkyQLELKDDYiirtnrlied<br>qklknellkyQLELKEDYitrtnrlied<br>qklktellkyQLELKDDYitrtnkllee<br>qklktellkyQLELKDDYitrtnkllee<br>qklktellkyqlelkeeyiartnkvted<br>qklknellrhqielneeyikrtkkvsed<br>qklksellkyqlelkeenmkrteklten<br>............................<br>............................<br>............................	Hs_ENSP00000344314<br>Bt_ENSBTAP00000005850<br>Rn_ENSRNOP00000006074<br>Mm_ENSMUSP00000041744<br>Gg_ENSGALP00000026704<br>Xt_ENSXETP00000029253<br>Dr_ENSDARP00000090445<br>Ce<br>Dm<br>Sc	NP_003602 Oral-facial-digital syndrome 1 protein (Protein 71-7A)
394	1	CI	ENSP00000272322<br>ENSG00000143952	836<br>841<br>836<br>836<br>839<br>832<br>847<br>918<br>804<br>782	ahfearlppkQYSMLRHFdhitkdyhdh<br>ahfearlqpkQYSMLRHFdhitkdyhdh<br>ahfearlppkQWSLLRHFdhitkdyhdh<br>ahfearlppkQWSMLRHFdhitkdyhdh<br>ahfearlqpkQFSMLRHFdhitkdyhdh<br>ahfearmqpkQYSMLRHFdhitkdyndh<br>ahfetrlqpkQYSILRHFdhitkdyndh<br>demdrvlpenrksllryfkqveseyrdh<br>ehfqamsg---------yetierdyqgh<br>vikelmvls---------inlpsiylsn	Hs_ENSP00000272322<br>Bt_ENSBTAP00000042231<br>Rn_ENSRNOP00000010264<br>Mm_ENSMUSP00000006221<br>Gg_ENSGALP00000014362<br>Xt_ENSXETP00000028501<br>Dr_ENSDARP00000068523<br>Ce_CE31365<br>Dm_FBpp0079409<br>Sc_YDR027C	NP_057600 Vacuolar protein sorting-associated protein 54 (Hepatocellular carcinoma protein 8)(HOM-HCC-8) (Tumor antigen SLP-8p)
395	1	CI	ENSP00000376932<br>ENSG00000067715	147<br>147<br>146<br>146<br>149<br>91<br>143<br>164<br>197<br>-	pkeeeklgklQYSLDYDFqnnqllvgii<br>pkeeeklgklQYSLDYDFqnnqllvgii<br>pkeeeklgklQYSLDYDFqnnqllvgii<br>pkeeeklgklQYSLDYDFqnnqllvgii<br>pkeveklgkiQYSLDYDFqnnqllvgii<br>pkeveklgklQYSLDYDFqnnqlmvgii<br>pkeeeklgklqysmdynftentlivgii<br>ekeevklgriQYKLDYDFqqgqltvtvi<br>kqseqklgrlnfkleydfnsnslavtvi<br>............................	Hs_ENSP00000376932<br>Bt_ENSBTAP00000008338<br>Rn_ENSRNOP00000049624<br>Mm_ENSMUSP00000100912<br>Gg_ENSGALP00000016781<br>Xt_ENSXETP00000008811<br>Dr_ENSDARP00000041860<br>Ce_CE28229<br>Dm_FBpp0077410<br>Sc	NP_005630 Synaptotagmin-1 (Synaptotagmin I)(SytI)(p65)
396	1	CI	ENSP00000220959<br>ENSG00000104517	878<br>852<br>806<br>878<br>858<br>-<br>880<br>906<br>912<br>-	imavekqtlmQHILRCDYeacrqylmnl<br>imavekqtlmQHILRCDYeacrqylmnl<br>imavekqtlmQHILRCDYeacrqylvnl<br>imavekqtlmQHILRCDYeacrqylvnl<br>imavekqtlmQHILRCDFeacrqylmnl<br>............................<br>imavekqtlmQHVLRCDYeacrqylisl<br>hfikdgsslmqmvlycdadgvsqfldrl<br>allfdtqklmphilrcdvknsfaalgrl<br>............................	Hs_ENSP00000220959<br>Bt_ENSBTAP00000028262<br>Rn_ENSRNOP00000009115<br>Mm_ENSMUSP00000105965<br>Gg_ENSGALP00000025803<br>Xt<br>Dr_ENSDARP00000033510<br>Ce_CE24948<br>Dm_FBpp0081568<br>Sc	NP_056986 E3 ubiquitin-protein ligase UBR5 (EC 6.3.2.-)(E3 ubiquitin-protein ligase, HECT domain-containing 1)(Hyperplastic discs protein homolog)(hHYD) (Progestin-induced protein)

397	1	CI	ENSP00000349962<br>ENSG00000183087	134<br>131<br>131<br>131<br>49<br>-<br>123<br>-<br>-<br>-	pcdrkgtqacQDLMGNFFclckagwggr<br>pcnkkgthvcQDLMGNFYcqcrdgwagr<br>pcdkkgtqlcQDLMGNFFclckdgwggr<br>pcdkkgthicQDLMGNFFcvctdgwggr<br>pcykegtvrcedlkgdfyceckrgwqgk<br>............................<br>pcyhygtvrcedkkgefrchcftgwsga<br>............................<br>............................<br>............................	Hs_ENSP00000349962<br>Bt_ENSBTAP00000035185<br>Rn_ENSRNOP00000024677<br>Mm_ENSMUSP00000033828<br>Gg_ENSGALP00000027103<br>Xt<br>Dr_ENSDARP00000018668<br>Ce<br>Dm<br>Sc	Growth arrest-specific protein 6 Precursor (GAS-6)(AXL receptor tyrosine kinase ligand)
398	1	C	ENSP00000220509<br>ENSG00000104142	352<br>352<br>352<br>352<br>352<br>318<br>352<br>298<br>380<br>287	rveavctltgQVVLRDHFlek-fgplkh<br>rveavctltgQVVLRDHFlek-fgplkh<br>rveavctltgQVVLRDHFlek-fgplrh<br>rveavctltgQVVLRDHFlek-fgplrh<br>rvmavctlneqvvfqdlflek-fgtltr<br>rikaicilngqvvfedvftek-fgplkk<br>rvrgictlngqvvhedvfpek-fgtlqk<br>rvlalsllpphdvifedpwnpelggalg<br>hvraicllnqeqvyqeafdearvgkpls<br>tvtmvsqlnndvvfhetiprhqltgsnt	Hs_ENSP00000220509<br>Bt_ENSBTAP00000015368<br>Rn_ENSRNOP00000018717<br>Mm_ENSMUSP00000036915<br>Gg_ENSGALP00000013826<br>Xt_ENSXETP00000046616<br>Dr_ENSDARP00000094129<br>Ce_CE32071<br>Dm_FBpp0070259<br>Sc_YLR148W	NP_065908 Vacuolar protein sorting-associated protein 18 homolog (hVPS18)
399	1	CI	ENSP00000355038<br>ENSG00000105639	418<br>418<br>556<br>625<br>-<br>-<br>-<br>-<br>-<br>-	fdsflltvcvQNPLGPDYkgclirrspt<br>fdsylltvcvltplgpdykgclircdpt<br>ydsflltacvQTPLGPDYkgclirqdps<br>ydsflltacvQTPLGPDYkgclirqdps<br>............................<br>............................<br>............................<br>............................<br>............................<br>............................	Hs_ENSP00000355038<br>Bt_ENSBTAP00000027857<br>Rn_ENSRNOP00000032301<br>Mm_ENSMUSP00000040236<br>Gg<br>Xt<br>Dr<br>Ce<br>Dm<br>Sc	NP_000206 Tyrosine-protein kinase JAK3 (EC 2.7.10.2) (Janus kinase 3)(JAK-3) (Leukocyte janus kinase)(L-JAK)
400	1	C	ENSP00000383143<br>ENSG00000184564	783<br>756<br>782<br>782<br>768<br>-<br>764<br>-<br>-<br>-	eylrkniaqlQPDMEAHYpgaheelklm<br>eylrkniaqlqpdtevhypgaheelklm<br>eylrkniaqlqpdvevnypgaheelklm<br>eylrknlaqlqpevevnypgaheelklm<br>eylrknivqlQPEMEVHYpgtheelklm<br>............................<br>eylrknisqlqpavdmqvpnhhkelklm<br>............................<br>............................<br>............................	Hs_ENSP00000383143<br>Bt_ENSBTAP00000005757<br>Rn_ENSRNOP00000034471<br>Mm_ENSMUSP00000077492<br>Gg_ENSGALP00000027274<br>Xt<br>Dr_ENSDARP00000094556<br>Ce<br>Dm<br>Sc	NP_115605 SLIT and NTRK-like protein 6 Precursor
401	1	CI	ENSP00000308820<br>ENSG00000166405	243<br>202<br>222<br>241<br>250<br>237<br>-<br>273<br>-<br>-	ydlsdcikrrQETILVDYpdpkelsaee<br>ydlsdcikhrQETILVDYpdskepsaee<br>---------------------kmp----<br>ydlsdgikrrQETILVDYpdlkepsaee<br>ydnsdcfkrkQDTILVDYpdlsqpsaee<br>ydpsdc-krtqqtilvdcsalnrpsaeq<br>............................<br>skeydkakmkklkrkdsssdedeedeee<br>............................<br>............................	Hs_ENSP00000308820<br>Bt_ENSBTAP00000006392<br>Rn_ENSRNOP00000020137<br>Mm_ENSMUSP00000056990<br>Gg_ENSGALP00000009586<br>Xt_ENSXETP00000042624<br>Dr<br>Ce_CE27448<br>Dm<br>Sc	Protein RIC-3 Precursor
402	1	CI	ENSP00000235329<br>ENSG00000116688	538<br>538<br>538<br>538<br>538<br>535<br>-<br>549<br>580<br>-	lncdklcadfQEDIEFHFslgwtmlvnr<br>lncdklcadfQEDIEFHFslgwtmlvnr<br>lncdklcadfQEDIEFHFslgwtmlvnr<br>lncdklcadfQEDIEFHFslgwtmlvnr<br>lncdklcadfQEDIEFHFslgwtmlvnr<br>lscdklcadfqedvefhfslgwtmlvnr<br>............................<br>vdvpalvndfhedlefrftfglhaiirr<br>lncqnlcadfqedlefkfswgiaamiqr<br>............................	Hs_ENSP00000235329<br>Bt_ENSBTAP00000043671<br>Rn_ENSRNOP00000052539<br>Mm_ENSMUSP00000101341<br>Gg_ENSGALP00000007187<br>Xt_ENSXETP00000028580<br>Dr<br>Ce_CE02898<br>Dm_FBpp0070873<br>Sc	NP_055689 Mitofusin-2 (EC 3.6.5.-)(Transmembrane GTPase MFN2)
403	1	CI	ENSP00000356545<br>ENSG00000198216	223<br>-<br>238<br>224<br>134<br>236<br>52<br>-<br>-<br>-	simkamvpllQIGLLLFFailmfaiigl<br>............................<br>simkamvpllQIGLLLFFailmfaiigl<br>simkamvpllQIGLLLFFailmfaiigl<br>simkamvpllQIGLLLFFailmfaiigl<br>simkamvpllQIGLLLFFailmfaiigl<br>simkamvpllQIGLLLFFailmfaiigl<br>............................<br>............................<br>............................	Hs_ENSP00000356545<br>Bt<br>Rn_ENSRNOP00000003928<br>Mm_ENSMUSP00000004214<br>Gg_ENSGALP00000038886<br>Xt_ENSXETP00000027314<br>Dr_ENSDARP00000084405<br>Ce<br>Dm<br>Sc	Voltage-dependent R-type calcium channel subunit alpha-1E (Voltage-gated calcium channel subunit alpha Cav2.3) (Calcium channel, L type, alpha-1 polypeptide, isoform 6) (Brain calcium channel II)(BII)

404	1	CI	ENSP00000373522<br>ENSG00000165097	373<br>371<br>377<br>379<br>168<br>372<br>-<br>343<br>-<br>-	ntgvlsvgadQYLLPKDYhnksviiiga<br>ntgvlsvgpdQHLLPKDYhnksviiiga<br>ntgvltvgagQHLLPKHYhnksvlvvga<br>ntgvltvaagQHLLPKHYhnksvlvvga<br>ntgilsvstdqyllpkeyhnksviivga<br>ntgvlsvspgqyllpkeyhnksviviga<br>............................<br>nygafdfr----idplngmrpkiaiiga<br>............................<br>............................	Hs_ENSP00000373522<br>Bt_ENSBTAP00000019452<br>Rn_ENSRNOP00000022244<br>Mm_ENSMUSP00000038373<br>Gg_ENSGALP00000020669<br>Xt_ENSXETP00000033669<br>Dr<br>Ce_CE41445<br>Dm<br>Sc	Flavin-containing amine oxidase domain-containing protein 1 (EC 1.-.-.-)
405	1	CI	ENSP00000329906<br>ENSG00000183775	93<br>93<br>84<br>93<br>93<br>-<br>94<br>98<br>-<br>-	ryildylrdrQVVLPDHFpekgrlkrea<br>ryildylrdrQVVLPDHFpergrlkrea<br>ryildylrdrQVVLPDHFpergrlkrea<br>ryildylrdrQVVLPDHFpergrlkrea<br>ryildylrdkQVVLPDHFpekgrlkrea<br>............................<br>ryvldylrdktvvlpdyfpekgrlkrea<br>ayvlhflrtdklslpeqfrevarlkdea<br>............................<br>............................	Hs_ENSP00000329906<br>Bt_ENSBTAP00000025305<br>Rn_ENSRNOP00000028520<br>Mm_ENSMUSP00000089547<br>Gg_ENSGALP00000020111<br>Xt<br>Dr_ENSDARP00000031234<br>Ce_CE40627<br>Dm<br>Sc	NP_065819 BTB/POZ domain-containing protein KCTD16
406	1	C	ENSP00000342828<br>ENSG00000119919	33<br>34<br>32<br>32<br>-<br>47<br>40<br>86<br>-<br>-	h-qhfhgahlQADLEHHFhs-apcmlaa<br>qqqhfhgahlQADLEHHFhs-apcmlaa<br>r--hfhgahlqaeleqhlhs-apcmlaa<br>r--hfhgahlQAELEQHFhs-apcmlat<br>............................<br>qcsrahpahpaqdldsdfqq-ascmlaa<br>lhpashhaqlhpelqdrfqapsscflgg<br>stneieqtafnfnfdpkmnp--isallq<br>............................<br>............................	Hs_ENSP00000342828<br>Bt_ENSBTAP00000028522<br>Rn_ENSRNOP00000051598<br>Mm_ENSMUSP00000050933<br>Gg<br>Xt_ENSXETP00000039924<br>Dr_ENSDARP00000057093<br>Ce_CE30891<br>Dm<br>Sc	NP_660328 Homeobox protein Nkx-2.3 (Homeobox protein NK-2 homolog C)
407	1	CI	ENSP00000384245<br>ENSG00000121988	519<br>455<br>353<br>518<br>517<br>-<br>-<br>-<br>-<br>-	alfthfekekQHDIRSFFvpqpkk-rql<br>mlfthfekekQRDIRSFFlpnakk-rql<br>-vfthfekekQHDIRSFFlpkprk-rpl<br>-vfthfekekQHDIRSFFlpklkk-rql<br>flfthfekerQHDIRSFFspkssagkkh<br>............................<br>............................<br>............................<br>............................<br>............................	Hs_ENSP00000384245<br>Bt_ENSBTAP00000038188<br>Rn_ENSRNOP00000005355<br>Mm_ENSMUSP00000083806<br>Gg_ENSGALP00000019932<br>Xt<br>Dr<br>Ce<br>Dm<br>Sc	NP_115519 Zinc finger Ran-binding domain-containing protein 3 (EC 3.6.1.-)
408	1	CI	ENSP00000247182<br>ENSG00000126778	85<br>158<br>85<br>85<br>-<br>85<br>85<br>94<br>179<br>-	fsphnhpklqQLWLKAHYveaeklrgrp<br>fsphnhpklqQLWLKAHYveaeklrgrp<br>fsphnhpklqQLWLKAHYveaeklrgrp<br>fsphnhpklqQLWLKAHYveaeklrgrp<br>............................<br>fsphnhpklqQLWLKAHYveaeklrgrp<br>fsphnhpklqQLWLKAHYieaeklrgrp<br>fasehhlplqewwlnahyheaekirgrq<br>fsaqnhaklqalwlkahyveaeklrgrp<br>............................	Hs_ENSP00000247182<br>Bt_ENSBTAP00000016104<br>Rn_ENSRNOP00000037345<br>Mm_ENSMUSP00000059026<br>Gg<br>Xt_ENSXETP00000036917<br>Dr_ENSDARP00000028379<br>Ce_CE08078<br>Dm_FBpp0089177<br>Sc	NP_005973 Homeobox protein SIX1 (Sine oculis homeobox homolog 1)
409	1	CI	ENSP00000322061<br>ENSG00000112936	189<br>189<br>187<br>-<br>185<br>-<br>191<br>-<br>-<br>-	lsgnvlsytfQVKINNDFnyefynstws<br>lsgnilsytfQVKINNDFnnefynstwa<br>lsgnilsytfQVKIDNDFnyefynssws<br>............................<br>lsesvlaytfQVKIQNDFsyeffnssws<br>............................<br>lpqnllrysfqvsvendftddyydssws<br>............................<br>............................<br>............................	Hs_ENSP00000322061<br>Bt_ENSBTAP00000047583<br>Rn_ENSRNOP00000057091<br>Mm<br>Gg_ENSGALP00000023901<br>Xt<br>Dr_ENSDARP00000074170<br>Ce<br>Dm<br>Sc	NP_000578 Complement component C7 Precursor
410	1	CI	ENSP00000264161<br>ENSG00000115866	255<br>255<br>255<br>255<br>257<br>279<br>290<br>285<br>285<br>307	ylaqspqlykQMCICADFekvfsigpvf<br>ylaqspqlykQMCICADFekvfcigpvf<br>ylaqspqlykQMCICADFekvfcigpvf<br>ylaqspqlykQMCICADFekvfcigpvf<br>ylaqspqlykQMCICADFekvfcvgpvf<br>ylaqspqlykQMCICADFekvfcigpvf<br>ylaqspqlykQMCICADFdkvfcvgpvf<br>ylaqspqlykQMAIAGDFekvytigpvf<br>ylaqspqlykQMAIAADFdkvytvgavf<br>ylaqspqfnkQQLIVADFervyeigpvf	Hs_ENSP00000264161<br>Bt_ENSBTAP00000013123<br>Rn_ENSRNOP00000005127<br>Mm_ENSMUSP00000027602<br>Gg_ENSGALP00000020154<br>Xt_ENSXETP00000053730<br>Dr_ENSDARP00000093243<br>Ce_CE00015<br>Dm_FBpp0086897<br>Sc_YLL018C	NP_001340 Aspartyl-tRNA synthetase, cytoplasmic (EC 6.1.1.12)(Aspartate--tRNA ligase)(AspRS)(Cell proliferation-inducing gene 40 protein)

411	1	C	ENSP00000378312<br>ENSG00000176927	454<br>71<br>120<br>226<br>220<br>-<br>-<br>-<br>-<br>-	ltelwgdmdnqkhiyegfdkvllemntl<br>lpefwgdmdnQKHIYEDFdnvllemntl<br>ltefwgdmdikkhiyenfdelllkmnml<br>liefwgdmdikkhiyedfdalllkmnml<br>ledlwggledqqgyeepgeaeiserdvs<br>............................<br>............................<br>............................<br>............................<br>............................	Hs_ENSP00000378312<br>Bt_ENSBTAP00000025455<br>Rn_ENSRNOP00000038171<br>Mm_ENSMUSP00000104037<br>Gg_ENSGALP00000006625<br>Xt<br>Dr<br>Ce<br>Dm<br>Sc	NP_940931 EF-hand calcium-binding domain-containing protein 5
412	1	CI	ENSP00000309189<br>ENSG00000174015	109<br>109<br>-<br>72<br>0<br>-<br>-<br>-<br>-<br>-	pldpmerpmsQADLELDYnpprvqlsde<br>pldpmerptsQADLELDYnpprvqlsde<br>............................<br>pfdpmerptsQADLELDYnpprvqlsde<br>----------------------------<br>............................<br>............................<br>............................<br>............................<br>............................	Hs_ENSP00000309189<br>Bt_ENSBTAP00000024729<br>Rn<br>Mm_ENSMUSP00000046259<br>Gg_ENSGALP00000027383<br>Xt<br>Dr<br>Ce<br>Dm<br>Sc	NP_689932 Spermatid-associated protein (Protein chibby homolog 2)
413	1	C	ENSP00000216487<br>ENSG00000100599	95<br>0<br>84<br>95<br>68<br>47<br>-<br>-<br>-<br>-	flvrrdssskQLVLCVHFpslnessaev<br>----------------------------<br>flvcrdsslkrlvlcvhfpslkntstev<br>flvcrdnnlkQLVLCVHFpslkgssaev<br>flvrkegnannmvlavrmp-vqsdapgv<br>flitrdverkcmvlwvhvtsnkeq-adv<br>............................<br>............................<br>............................<br>............................	Hs_ENSP00000216487<br>Bt_ENSBTAP00000013748<br>Rn_ENSRNOP00000009266<br>Mm_ENSMUSP00000060771<br>Gg_ENSGALP00000017549<br>Xt_ENSXETP00000056717<br>Dr<br>Ce<br>Dm<br>Sc	NP_079108 Ras and Rab interactor 3 (Ras interaction/interference protein 3)
414	1	CI	ENSP00000347205<br>ENSG00000107331	1491<br>-<br>1460<br>1459<br>1398<br>848<br>1439<br>-<br>-<br>-	arrnskalfsQILLPAFFvcvamtvals<br>............................<br>arrnskalcsQILLPAFFvcvamtvals<br>arrnskalcsQILLPAFFvcvamtvals<br>akrntkalfsQILLPAFFvcvamtvals<br>akrntkalfsQILLPAFFvcvamtvals<br>akrntkgifsQILLPAFFvcvamtvals<br>............................<br>............................<br>............................	Hs_ENSP00000347205<br>Bt<br>Rn_ENSRNOP00000020339<br>Mm_ENSMUSP00000099983<br>Gg_ENSGALP00000039707<br>Xt_ENSXETP00000008641<br>Dr_ENSDARP00000085176<br>Ce<br>Dm<br>Sc	NP_997698 ATP-binding cassette sub-family A member 2 (ATP-binding cassette transporter 2)(ATP-binding cassette 2)
415	1	CI	ENSP00000360163<br>ENSG00000102038	101<br>29<br>104<br>105<br>25<br>-<br>22<br>55<br>-<br>-	rakrfefllkQTELFAHFiqpsaqkspt<br>rakrfefllkQTELFAHFiqpsaqkspt<br>rakrfefllkQTELFAHFiqpsaqkspt<br>rakrfefllkQTELFAHFiqpsaqkspt<br>rakrfefllkQTELFAHFiqpaaqkspt<br>............................<br>ranrfefllkQTELFAHFiqpasqkspt<br>sfkrferllqktenfshclssgdaklat<br>............................<br>............................	Hs_ENSP00000360163<br>Bt_ENSBTAP00000002973<br>Rn_ENSRNOP00000005111<br>Mm_ENSMUSP00000099138<br>Gg_ENSGALP00000013722<br>Xt<br>Dr_ENSDARP00000023313<br>Ce_CE29792<br>Dm<br>Sc	NP_003060 Probable global transcription activator SNF2L1 (EC 3.6.1.-)(Nucleosome-remodeling factor subunit SNF2L)(ATP-dependent helicase SMARCA1)(SWI/SNF-related matrix-associated actin-dependent regulator of chromatin subfamily A member 1)
416	1	C	ENSP00000264169<br>ENSG00000115947	263<br>263<br>261<br>260<br>263<br>261<br>-<br>-<br>285<br>321	ylsed--rsvQEVLQKHFnisknlrslh<br>slsed--rsvkevlqkhfnvsknlrslh<br>clsed--stvlevlqkhfnvnknlqslh<br>clsed--stvlevlqkhfsvnknlqslh<br>llted--ktvqdilqnlfhyskdlrsln<br>slves--kmvedilqkqysaskdvrslh<br>............................<br>............................<br>kvlat--qqarstlqalhdfdiseaylk<br>kelsdprsnlnrhirmnfetfrslptlk	Hs_ENSP00000264169<br>Bt_ENSBTAP00000020335<br>Rn_ENSRNOP00000006937<br>Mm_ENSMUSP00000028098<br>Gg_ENSGALP00000020323<br>Xt_ENSXETP00000046974<br>Dr<br>Ce<br>Dm_FBpp0072330<br>Sc_YPR162C	NP_859526 Origin recognition complex subunit 4
417	1	CI	ENSP00000316589<br>ENSG00000141084	70<br>121<br>98<br>98<br>90<br>-<br>58<br>60	kdkynyiglsQGNLRVHYkg---hgknh<br>kdkynyiglsQGNLRVHYkg---hgknh<br>kdkynyiglsQGNLRVHYkg---hgknh<br>kdkynyiglsQGNLRVHYkg---hgknh<br>kdkynyiglsQGNLRVHYkg---hgknh<br>............................<br>kdkysyiglsQNNLRVHYkg---hgknh<br>rqassyldisqsgygvtfkknvtvaedk	Hs_ENSP00000316589<br>Bt_ENSBTAP00000043820<br>Rn_ENSRNOP00000024891<br>Mm_ENSMUSP00000040045<br>Gg_ENSGALP00000002189<br>Xt<br>Dr_ENSDARP00000083940<br>Ce_CE20318	NP_065901 Ran-binding protein 10 (RanBP10)

				-<br>39	............................<br>rgdvsyggddtdelnmdiml--------	Dm<br>Sc_YMR171C	
418	1	CI	ENSP00000285208<br>ENSG00000154917	43<br>43<br>43<br>43<br>-<br>-<br>43<br>-<br>-<br>40	fmydsfdntyQATIGIDFlsktmyledr<br>fmydsfdntyQATIGIDFlsktmyledr<br>fmydsfdntyQATIGIDFlsktmyledr<br>fmydsfdntyQATIGIDFlsktmyledr<br>............................<br>............................<br>fmydsfdntyQATIGIDFlsktmyledr<br>............................<br>............................<br>fmydtfddhyQATIGIDFlsktmylddk	Hs_ENSP00000285208<br>Bt_ENSBTAP00000001199<br>Rn_ENSRNOP00000012219<br>Mm_ENSMUSP00000035155<br>Gg<br>Xt<br>Dr_ENSDARP00000049176<br>Ce<br>Dm<br>Sc_YLR262C	NP_057661 Ras-related protein Rab-6B
419	1	CI	ENSP00000254998<br>ENSG00000132661	55<br>55<br>55<br>55<br>-<br>-<br>-<br>-<br>-<br>-	lvwngnavsgQESLSEFFemlpssefqi<br>lvwngnavsgQESLSEFFemlpssefqi<br>lvwngnavsgQESLSEFFemlpssefqi<br>lvwngnavsgQESLSEFFemlpssefqi<br>............................<br>............................<br>............................<br>............................<br>............................<br>............................	Hs_ENSP00000254998<br>Bt_ENSBTAP00000006568<br>Rn_ENSRNOP00000006205<br>Mm_ENSMUSP00000105587<br>Gg<br>Xt<br>Dr<br>Ce<br>Dm<br>Sc	NP_037380 NTF2-related export protein 1 (p15)
420	1	CI	ENSP00000379042<br>ENSG00000197930	66<br>66<br>66<br>66<br>64<br>29<br>58<br>-<br>75<br>-	fnnyrlfprlQKLLESDYfryykvnlkr<br>fnnyrlfprlQKLLESDYfryykvnlkr<br>fnnyrlfprlQKLLESDYfryykvnlrk<br>fnnyrlfprlQKLLESDYfryykvnlkk<br>fnnyklfprlnqllesdyfryykvnlkk<br>fnnyglfpklqqlvasdyfryykanlkk<br>fnnkdifpklQKLLSSDYfrfykvnlnn<br>............................<br>fnnmkiyprlqsllvknffrfykvnlrq<br>............................	Hs_ENSP00000379042<br>Bt_ENSBTAP00000020878<br>Rn_ENSRNOP00000009404<br>Mm_ENSMUSP00000022378<br>Gg_ENSGALP00000020229<br>Xt_ENSXETP00000026655<br>Dr_ENSDARP00000015478<br>Ce<br>Dm_FBpp0073072<br>Sc	ERO1-like protein alpha Precursor (ERO1-Lalpha)(ERO1-L)(EC 1.8.4.-)(Oxidoreductin-1-Lalpha)(Endoplasmic oxidoreductin-1-like protein)
421	1	CI	ENSP00000217173<br>ENSG00000185019	534<br>502<br>531<br>532<br>527<br>532<br>484<br>-<br>479<br>-	ctacqrpvasQDVLRVHF----------<br>ctacqqpfasQDILRVHF----------<br>ctacqqpvtsQDVLRVHF----------<br>ctacrqpvtsQDVLRVHF----------<br>cvsckrsvathdirrvhf----------<br>cnncnrsvatrdvqrvhl----------<br>cpacgssassrditrvhh----------<br>............................<br>ckvffrcadveryhkldp----------<br>............................	Hs_ENSP00000217173<br>Bt_ENSBTAP00000020438<br>Rn_ENSRNOP00000028834<br>Mm_ENSMUSP00000028761<br>Gg_ENSGALP00000025737<br>Xt_ENSXETP00000057045<br>Dr_ENSDARP00000075886<br>Ce<br>Dm_FBpp0084991<br>Sc	NP_055763 RING finger protein 37 (Ubiquitin-conjugating enzyme 7-interacting protein 5)(U-box domain-containing protein 5)
422	1	CI	ENSP00000276390<br>ENSG00000147416	497<br>511<br>506<br>497<br>497<br>490<br>496<br>477<br>477<br>478	fpkemlkripQSTLSEFYprds-akh--<br>fpkemlkripQSTLSEFYprds-akh--<br>fpkemlkripQSTLSEFYprds-akh--<br>fpkemlkripQSTLSEFYprds-akh--<br>fpkemlkripQTTLAEFYprdstakh--<br>fpkellkripQSTLAEFYprdssakh--<br>fpkemlkripQSTLAEFYprds--kh--<br>fpremlkripestlekyyprgg-ake--<br>fpkemlkripasilaefyprds--rh--<br>ypkemlnrispkildefydrarddaded	Hs_ENSP00000276390<br>Bt_ENSBTAP00000024812<br>Rn_ENSRNOP00000015931<br>Mm_ENSMUSP00000006435<br>Gg_ENSGALP00000040078<br>Xt_ENSXETP00000035945<br>Dr_ENSDARP00000063805<br>Ce_CE04424<br>Dm_FBpp0082139<br>Sc_YBR127C	NP_001684 V-type proton ATPase subunit B, brain isoform (V-ATPase subunit B 2)(Vacuolar proton pump subunit B 2)(Endomembrane proton pump 58 kDa subunit)(HO57)
423	1	C	ENSP00000313504<br>ENSG00000102893	532<br>507<br>532<br>524<br>525<br>529<br>-<br>294<br>519<br>-	qvepiqiwpqqelvkaylqlgineklgl<br>qvepiqiwaqqelvkayfhlgvneklgl<br>qvepiqiwpqqelvkayfhlgineklgl<br>qvepiqiwpqqelvkayfhlgineklgl<br>qvepiqiwaqkelvkayfhlgindklgl<br>qvepiqiwpqqelvkaylhlgvnnklgl<br>............................<br>evepvqiwpswrmvkvfeclgrdkkldl<br>evepvqiwsstelikvyqhlgvnnkvgl<br>............................	Hs_ENSP00000313504<br>Bt_ENSBTAP00000006312<br>Rn_ENSRNOP00000039778<br>Mm_ENSMUSP00000050788<br>Gg_ENSGALP00000006368<br>Xt_ENSXETP00000008502<br>Dr<br>Ce_CE41734<br>Dm_FBpp0079254<br>Sc	NP_000284 Phosphorylase b kinase regulatory subunit beta (Phosphorylase kinase subunit beta)
424	1	CI	ENSP00000303960<br>ENSG00000006071	1197<br>1200<br>1201<br>1203<br>1195<br>-<br>-<br>-	rdlqqlddttQLPLLSHFaetvegltti<br>rdlqqlddttQLPLLSHFaetvegltti<br>rdlqqlddttQLPLLSHFaetvegltti<br>rdlqqlddttQLPLLSHFaetvegltti<br>rdlqqlddstQLPLLSHFsetvegltti<br>............................<br>............................<br>............................	Hs_ENSP00000303960<br>Bt_ENSBTAP00000043950<br>Rn_ENSRNOP00000041893<br>Mm_ENSMUSP00000033123<br>Gg_ENSGALP00000009950<br>Xt<br>Dr<br>Ce	ATP-binding cassette transporter sub-family C member 8 (Sulfonylurea receptor 1)

				-<br>-	............................<br>............................	Dm<br>Sc	
425	1	CI	ENSP00000286719<br>ENSG00000156194	59<br>59<br>59<br>59<br>61<br>64<br>57<br>122<br>45<br>-	qsieyagqqdQVKLHDFFsylmdhfips<br>qsieyagqqdQVKLHNFFsylvdhftps<br>qsieyagqqdQVKLHEFFsylvdhftps<br>qsieyagqqdQVKLHEFFsylvdhftps<br>qsieyaceqdQIKLHNFFsylmdnftps<br>qsieyaeeqaQLQLSNFFtfmmdhfahw<br>qsieyageqdQLQLSSFFtfmldnftqi<br>taleyageqdQLKLYDFFadviramaee<br>qnleyaseqdQAELYKFFndlikhmpqa<br>............................	Hs_ENSP00000286719<br>Bt_ENSBTAP00000020539<br>Rn_ENSRNOP00000003122<br>Mm_ENSMUSP00000031359<br>Gg_ENSGALP00000018767<br>Xt_ENSXETP00000014755<br>Dr_ENSDARP00000009463<br>Ce_CE27999<br>Dm_FBpp0074602<br>Sc	NP_006230 Serine/threonine-protein phosphatase with EF-hands 2 (PPEF-2)(EC 3.1.3.16)
426	1	CI	ENSP00000382387<br>ENSG00000137822	353<br>353<br>353<br>353<br>352<br>352<br>353<br>-<br>346<br>-	lmveesdllgQLKIIKDFyllgrgelfq<br>lmveesdllgQLKIIKDFyllgrgelfq<br>lmveesdllgQLKIIKDFyllgrgelfq<br>lmveesdllgQLKIIKDFyllgrgelfq<br>lmveesdllgQLKIIKDFyllgrgelfq<br>lmveesdllgQLKIIKDFyllgrgelfq<br>lmveesdllgQLKIIKDFfllgrgelyq<br>............................<br>iavnevdlerQMGLIKDFfllgrgefyl<br>............................	Hs_ENSP00000382387<br>Bt_ENSBTAP00000019254<br>Rn_ENSRNOP00000018460<br>Mm_ENSMUSP00000044049<br>Gg_ENSGALP00000013911<br>Xt_ENSXETP00000010219<br>Dr_ENSDARP00000025789<br>Ce<br>Dm_FBpp0079623<br>Sc	Gamma-tubulin complex component 4 (GCP-4)(hGCP4)(Hgrip76)(h76p)
426	2	CI	ENSP00000382387<br>ENSG00000137822	644<br>643<br>644<br>644<br>642<br>642<br>647<br>-<br>631<br>-	rnhqinsdlaQLLLRLDYnkyytqaggt<br>rnhqinsdlaQLLLRLDYnkyytqaggt<br>rnhqinsdlaQLLLRLDYnkyytqaggt<br>rnhqinsdlaQLLLRLDYnkyytqaggt<br>rnhqinsdlaQLLLRLDYnkyytqaggt<br>rnhqinpdlaQLLLRLDYnkyytqaggt<br>rnhqinsdlaQLLLRLDYnkyytqaggt<br>............................<br>ksasclgplsQLLLRLDFncwfsashnt<br>............................	Hs_ENSP00000382387<br>Bt_ENSBTAP00000019254<br>Rn_ENSRNOP00000018460<br>Mm_ENSMUSP00000044049<br>Gg_ENSGALP00000013911<br>Xt_ENSXETP00000010219<br>Dr_ENSDARP00000025789<br>Ce<br>Dm_FBpp0079623<br>Sc	Gamma-tubulin complex component 4 (GCP-4)(hGCP4)(Hgrip76)(h76p)
427	1	CI	ENSP00000359805<br>ENSG00000151914	5003<br>1490<br>4888<br>4932<br>4879<br>4549<br>-<br>2497<br>6161<br>-	ensiaklkslQKEMDQHFgmvellnnta<br>enalarlkalQKEMDQHFglveqlsgaa<br>ersitelktlQKEMDHHFgtlellnnsa<br>essiaelkslQKEMDHHFgmlellnnta<br>ensvvqvrawqkdldkhhgivellnnta<br>eaqllrakalqtdidkkrsllemmnsaa<br>............................<br>pevcdalklveltmarrladvdalnavm<br>etrlrdlqtfrsevwkhsgefentkglg<br>............................	Hs_ENSP00000359805<br>Bt_ENSBTAP00000028013<br>Rn_ENSRNOP00000051106<br>Mm_ENSMUSP00000095392<br>Gg_ENSGALP00000026214<br>Xt_ENSXETP00000016061<br>Dr<br>Ce_CE35152<br>Dm_FBpp0086747<br>Sc	NP_065121 Bullous pemphigoid antigen 1, isoforms 1/2/3/4/5/8 Fragment (230 kDa bullous pemphigoid antigen)(BPA)(Hemidesmosomal plaque protein)(Dystonia musculorum protein)(Dystonin)
427	2	C	ENSP00000359805<br>ENSG00000151914	5938<br>2410<br>5713<br>5758<br>5815<br>5483<br>-<br>3427<br>7087<br>-	serylqleraqslvnqfwetyeelwpwl<br>serylqleraqslvsqfwetyeelwpwl<br>serhlqleraqslvsqfwetyeelwpwl<br>serhlqleraqslvsqfwetyeelwpwl<br>sernlqleraqslvnqfwetyeelwpwl<br>seryarleraqvlvgqfwetyeelipwi<br>............................<br>adrtetleralalmeqltsqfdelnkwl<br>serlgileqalplsehfadshqgltawl<br>............................	Hs_ENSP00000359805<br>Bt_ENSBTAP00000028013<br>Rn_ENSRNOP00000051106<br>Mm_ENSMUSP00000095392<br>Gg_ENSGALP00000026214<br>Xt_ENSXETP00000016061<br>Dr<br>Ce_CE35152<br>Dm_FBpp0086747<br>Sc	NP_065121 Bullous pemphigoid antigen 1, isoforms 1/2/3/4/5/8 Fragment (230 kDa bullous pemphigoid antigen)(BPA)(Hemidesmosomal plaque protein)(Dystonia musculorum protein)(Dystonin)
428	1	C	ENSP00000334219<br>ENSG00000105429	67<br>0<br>0<br>0<br>-<br>-<br>-<br>-<br>69<br>-	cewlieapspQHRILLDFlfldtectyd<br>----------------------------<br>----------------------------<br>----------------------------<br>............................<br>............................<br>............................<br>............................<br>cewlikarndsqyitltfhsmgtecsyd<br>............................	Hs_ENSP00000334219<br>Bt_ENSBTAP00000041399<br>Rn_ENSRNOP00000027831<br>Mm_ENSMUSP00000083124<br>Gg<br>Xt<br>Dr<br>Ce<br>Dm_FBpp0079163<br>Sc	NP_001401 Multiple epidermal growth factor-like domains 8 (EGF-like domain-containing protein 4)(Multiple EGF-like domain protein 4)
429	1	CI	ENSP00000376822<br>ENSG00000115107	411<br>306<br>-<br>342<br>301<br>-<br>-<br>-	wldhwlqhrkQIGLLSFFcaalhalysf<br>wldhwlqhrkQIGLLSFFcaalhalysl<br>............................<br>wldhwlqhrkQIGLLSFFfamlhalysf<br>wldqwlqhrkQIGLLSFFcaalhavysl<br>............................<br>............................<br>............................	Hs_ENSP00000376822<br>Bt_ENSBTAP00000009356<br>Rn<br>Mm_ENSMUSP00000056248<br>Gg_ENSGALP00000019763<br>Xt<br>Dr<br>Ce	NP_060704 Metalloreductase STEAP3 (EC 1.16.1.-)(Six-transmembrane epithelial antigen of prostate 3)(Tumor suppressor-activated pathway protein 6)(hTSAP6)(pHyde)(hpHyde)(Dudulin-2)

				-<br>-	............................<br>............................	Dm<br>Sc	
430	1	C	ENSP00000359478<br>ENSG00000023839	447<br>377<br>-<br>445<br>466<br>466<br>467<br>-<br>-<br>-	fmhmlwssvlQIVLSIFFlwrelgpsvl<br>fihllwsnvlqialaiyflwaelgpsvl<br>............................<br>yihllwssvlQIALSIFFlwrelgpsil<br>fvhqlwssplqiilsivflwgelgpsvl<br>fihllwssplqiaisivflweelgpsvl<br>fihllwscplqialsiaflwielgpsvl<br>............................<br>............................<br>............................	Hs_ENSP00000359478<br>Bt_ENSBTAP00000044493<br>Rn<br>Mm_ENSMUSP00000026208<br>Gg_ENSGALP00000011951<br>Xt_ENSXETP00000008095<br>Dr_ENSDARP00000025026<br>Ce<br>Dm<br>Sc	NP_000383 Canalicular multispecific organic anion transporter 1 (ATP-binding cassette sub-family C member 2)(Multidrug resistance-associated protein 2) (Canalicular multidrug resistance protein)
431	1	CI	ENSP00000287482<br>ENSG00000156876	73<br>73<br>73<br>73<br>77<br>73<br>73<br>-<br>87<br>-	dfq---slkfQQGLLVDFlafpqkfidl<br>dfq---slkfQQGLLVDFlafpqkfidl<br>dfq---slklQQGLLVDFlsfpqkfidl<br>dfq---slklQQGLLVDFlafpqkfidl<br>dfqrqvnlksQQGLLVDFsafpqkfidl<br>dfq---slknqqgllvefsafpqrfidl<br>dfq---slkvQQGLLIDFtsfpqkfidl<br>............................<br>sfq---dlkqdqslnvsfsgfidnvvrm<br>............................	Hs_ENSP00000287482<br>Bt_ENSBTAP00000020951<br>Rn_ENSRNOP00000020757<br>Mm_ENSMUSP00000029571<br>Gg_ENSGALP00000008489<br>Xt_ENSXETP00000020161<br>Dr_ENSDARP00000075634<br>Ce<br>Dm_FBpp0084912<br>Sc	NP_919268 Spindle assembly abnormal protein 6 homolog (HsSAS-6)
432	1	CI	ENSP00000308714<br>ENSG00000186513	100<br>104<br>100<br>100<br>-<br>-<br>-<br>-<br>-<br>-	ttisqarcaaQFFLFTFFasidcyllai<br>vvlsrarcatQFFLFTFFasmdcyllai<br>ttisqvrcavQFFLFTFFasidcyllai<br>stisqvrcavQFFLFTFFasidcyllai<br>............................<br>............................<br>............................<br>............................<br>............................<br>............................	Hs_ENSP00000308714<br>Bt_ENSBTAP00000036889<br>Rn_ENSRNOP00000017615<br>Mm_ENSMUSP00000053465<br>Gg<br>Xt<br>Dr<br>Ce<br>Dm<br>Sc	NP_001005283 Olfactory receptor 9Q2
433	1	CI	ENSP00000290374<br>ENSG00000159248	192<br>192<br>192<br>192<br>175<br>175<br>175<br>-<br>-<br>-	rtasksklrrQEGISRFYiiqvvfrnal<br>rtasrsklrrQEGISRFYiiqvvfrnal<br>rtaarsklrrQEGISRFYiiqvvfrnal<br>rtaarsklrrQEGISRFYiiqvvfrnal<br>rtt-kskmrrQEGISRFYiiqvvfrnal<br>rtt-kskmrrQEGISRFYiiqvvfrnal<br>rtt-kskmrrQEGISRFYiiqvvfrnal<br>............................<br>............................<br>............................	Hs_ENSP00000290374<br>Bt_ENSBTAP00000028966<br>Rn_ENSRNOP00000011078<br>Mm_ENSMUSP00000087742<br>Gg_ENSGALP00000015989<br>Xt_ENSXETP00000028243<br>Dr_ENSDARP00000095009<br>Ce<br>Dm<br>Sc	NP_065711 Gap junction delta-2 protein (Gap junction alpha-9 protein)(Connexin-36)(Cx36)
434	1	CI	ENSP00000356846<br>ENSG00000143194	147<br>147<br>147<br>147<br>148<br>-<br>-<br>-<br>-<br>-	ceigcvkyslQEGIMADFhsfinpgeip<br>ceigcvkyslQEGIMADFhsfinpgeip<br>ceigcvkyslQEGIMADFhsfinpgeip<br>ceigcvkyslQEGIMADFhsfihpgeip<br>ceigcvkyslQEGIMADFhhfidsevpp<br>............................<br>............................<br>............................<br>............................<br>............................	Hs_ENSP00000356846<br>Bt_ENSBTAP00000022014<br>Rn_ENSRNOP00000005060<br>Mm_ENSMUSP00000045828<br>Gg_ENSGALP00000036066<br>Xt<br>Dr<br>Ce<br>Dm<br>Sc	NP_116247 Maelstrom homolog
435	1	CI	ENSP00000373863<br>ENSG00000079337	748<br>748<br>752<br>701<br>629<br>677<br>-<br>-<br>-<br>-	fiklaahlkeQKNLNSFFavmfglsnsa<br>fiklaahlkeQKNLNSFFaimfglsnsa<br>fiklaahlkeQKNLNSFFavmfglsnsa<br>fiklaahlkeQKNLNSFFavmfglsnsa<br>fiklaahlkeQKNLNSFFavmfgvsnta<br>fiklaaylkeQKNLNSFFavmfglsnta<br>............................<br>............................<br>............................<br>............................	Hs_ENSP00000373863<br>Bt_ENSBTAP00000008976<br>Rn_ENSRNOP00000010295<br>Mm_ENSMUSP00000023109<br>Gg_ENSGALP00000010246<br>Xt_ENSXETP00000009345<br>Dr<br>Ce<br>Dm<br>Sc	NP_001092002 Rap guanine nucleotide exchange factor 3 (cAMP-regulated guanine nucleotide exchange factor I) (cAMP-GEFI)(Exchange factor directly activated by cAMP 1) (Epac 1)(Rap1 guanine-nucleotide-exchange factor directly activated by cAMP)
436	1	CI	ENSP00000308716<br>ENSG00000175189	111<br>111<br>111<br>112<br>-<br>-<br>-	faetglstinQTRLDFHFssdrtagdre<br>faetglcnttQTRLDFHFssdssaggle<br>fadtglsninQTRLEFHFs-drttggve<br>fadtdlssinQTRLEFHFs-grmasgme<br>............................<br>............................<br>............................	Hs_ENSP00000308716<br>Bt_ENSBTAP00000025401<br>Rn_ENSRNOP00000010240<br>Mm_ENSMUSP00000026472<br>Gg<br>Xt<br>Dr	NP_005529 Inhibin beta C chain Precursor (Activin beta-C chain)

				-<br>-<br>-	............................<br>............................<br>............................	Ce<br>Dm<br>Sc	
437	1	C	ENSP00000219334<br>ENSG00000103056	475<br>474<br>473<br>473<br>480<br>477<br>379<br>-<br>-<br>-	pqedsaircgqldllqdwladfrkstss<br>lsedsdirceqlnmlqdwladfrkstss<br>ppedsairceqldllqdwladfrkstss<br>ppedsavrceqldllqdwladfrkstss<br>iagdttvrceqldmlqdwlsefrkstss<br>lagdamtrceqldmlqewvsefrkstss<br>iegdaavrceqldmllewgaefrrvtsc<br>............................<br>............................<br>............................	Hs_ENSP00000219334<br>Bt_ENSBTAP00000012702<br>Rn_ENSRNOP00000000274<br>Mm_ENSMUSP00000069255<br>Gg_ENSGALP00000001129<br>Xt_ENSXETP00000014618<br>Dr_ENSDARP00000093978<br>Ce<br>Dm<br>Sc	NP_061137 Sphingomyelin phosphodiesterase 3 (EC 3.1.4.12)(Neutral sphingomyelinase II)(Neutral sphingomyelinase 2)(nSMase-2)(nSMase2)
437	2	CI	ENSP00000219334<br>ENSG00000103056	525<br>524<br>523<br>523<br>530<br>527<br>431<br>-<br>-<br>-	ncssddkleqQHSLFTHYrdpcrlgpge<br>ncssddkleqqhslftrykdpcrlgpge<br>ncssddkleqqhslftrykdpcrlgpge<br>ncssddkleqqhslftrykdpcrlgpge<br>ncssedkleqQHSLFTHYkdpcrigpge<br>ncssedkleqQHSLFTHYkdpcrlspge<br>ncssedkleqQHSLFTHYkdpcrlgpge<br>............................<br>............................<br>............................	Hs_ENSP00000219334<br>Bt_ENSBTAP00000012702<br>Rn_ENSRNOP00000000274<br>Mm_ENSMUSP00000069255<br>Gg_ENSGALP00000001129<br>Xt_ENSXETP00000014618<br>Dr_ENSDARP00000093978<br>Ce<br>Dm<br>Sc	NP_061137 Sphingomyelin phosphodiesterase 3 (EC 3.1.4.12)(Neutral sphingomyelinase II)(Neutral sphingomyelinase 2)(nSMase-2)(nSMase2)
438	1	CI	ENSP00000342343<br>ENSG00000148297	84<br>84<br>84<br>84<br>84<br>84<br>84<br>98<br>87<br>-	slmklvsdlkQFLILNDFpsvneaidqr<br>slmklvsdlkQFLILNDFpsvneaidqr<br>slmklvsdlkQFLILNDFpsvneaidqr<br>slmklvsdlkQFLILNDFpsvneaidqr<br>slmklvsdlkQFLILNDFpsvneainqr<br>slmklvsdlkQFLILNDFpsvnesinqr<br>slmklvsdlkQFLILNDFpsvneaislr<br>ellkltadlkeflilhdfhflthnikqa<br>slmklvadlkQYLILNDFhsvneaitnn<br>............................	Hs_ENSP00000342343<br>Bt_ENSBTAP00000015357<br>Rn_ENSRNOP00000006720<br>Mm_ENSMUSP00000015920<br>Gg_ENSGALP00000038549<br>Xt_ENSXETP00000010946<br>Dr_ENSDARP00000011330<br>Ce_CE02403<br>Dm_FBpp0070160<br>Sc	NP_598395 Mediator of RNA polymerase II transcription subunit 22 (Mediator complex subunit 22)(Surfeit locus protein 5)(Surf-5)
439	1	C	ENSP00000311682<br>ENSG00000141342	924<br>-<br>927<br>942<br>887<br>860<br>713<br>-<br>577<br>-	rmhligrrreQLKLLGDYlglcrsg---<br>............................<br>rmhligrsreQLKLLGDYlglcrsg---<br>rmhligrsreQLKLLGDYlglcrsg---<br>-vgfvgtsgsamrllgtvsalrspgafi<br>tmhnishsreklrllaeylqtcrsg---<br>imakvqnlrqrlrflgdyvllcrsg---<br>............................<br>amaelqslrirlnfiraylytcaps---<br>............................	Hs_ENSP00000311682<br>Bt<br>Rn_ENSRNOP00000034752<br>Mm_ENSMUSP00000047327<br>Gg_ENSGALP00000039923<br>Xt_ENSXETP00000007501<br>Dr_ENSDARP00000086673<br>Ce<br>Dm_FBpp0071672<br>Sc	NP_055613 Pleckstrin homology domain-containing family M member 1 (162 kDa adapter protein)(AP162)
440	1	CI	ENSP00000278550<br>ENSG00000149256	888<br>724<br>949<br>957<br>665<br>954<br>951<br>-<br>-<br>-	iiqetqvpvsQQNLHSFYdrikflvgrd<br>iiqetqapvsQQNLHSFYdrikflvgrd<br>iiqetqapvsQQNLHSFYdrikflvgrd<br>iiqetqapvsQQNLNSFYdrikflvgrd<br>iiqetqapisQQSLHSFYdrikfligkd<br>iiqetqapvphqnlqsfynrvkfligkd<br>iiqetqisssIstlqsfyqrihflvgrd<br>............................<br>............................<br>............................	Hs_ENSP00000278550<br>Bt_ENSBTAP00000045891<br>Rn_ENSRNOP00000052240<br>Mm_ENSMUSP00000102787<br>Gg_ENSGALP00000027844<br>Xt_ENSXETP00000033455<br>Dr_ENSDARP00000016395<br>Ce<br>Dm<br>Sc	NP_001092286 Teneurin-4 (Ten-4)(Tenascin-M4)(Ten-m4)(Protein Odd Oz/ten-m homolog 4)
441	1	CI	ENSP00000378611<br>ENSG00000138639	719<br>632<br>719<br>718<br>708<br>510<br>631<br>-<br>-<br>-	edaekrndmlQKEMEQFFstfgeltvep<br>edaekrndmlQKEMEQFFstfgeltvep<br>edaekrndmlQKEMEQFFstfgdltvep<br>edaekrndmlQKEMEQFFstfgdltvep<br>edaekrndmlQKEMEQFFstfgeltvep<br>edaekrndmlQKEMEQFFstfgdltvep<br>ddaekrnemlQKEMEQFFstfgdltadp<br>............................<br>............................<br>............................	Hs_ENSP00000378611<br>Bt_ENSBTAP00000005165<br>Rn_ENSRNOP00000002857<br>Mm_ENSMUSP00000092138<br>Gg_ENSGALP00000018112<br>Xt_ENSXETP00000013631<br>Dr_ENSDARP00000078930<br>Ce<br>Dm<br>Sc	NP_001020787 Rho GTPase-activating protein 24 (Rho-type GTPase-activating protein 24)(Filamin-A-associated RhoGAP)(FilGAP)(RhoGAP of 73 kDa)(p73RhoGAP)(RAC1- and CDC42-specific GTPase-activating protein of 72 kDa)(RC-GAP72)(Sarcoma antigen NY-SAR-88)
442	1	CI	ENSP00000254436<br>ENSG00000132109	406<br>418<br>413<br>409<br>-	tplhlqvppcQVGIFLDYeagmvsfyni<br>tplhlqvppnrigifldyeastvsfyni<br>ttlhiqvppcQIGIFVDYeagivsfyni<br>ttlhiqvppcQIGIFVDYeagvvsfyni<br>............................	Hs_ENSP00000254436<br>Bt_ENSBTAP00000018459<br>Rn_ENSRNOP00000024992<br>Mm_ENSMUSP00000033264<br>Gg	NP_003132 52 kDa Ro protein (Sjoegren syndrome type A antigen)(SS-A)(Ro(SS-A))(52 kDa ribonucleoprotein

				-<br>-<br>-<br>-<br>-	............................<br>............................<br>............................<br>............................<br>............................	Xt<br>Dr<br>Ce<br>Dm<br>Sc	autoantigen Ro/SS-A)(Tripartite motif-containing protein 21)(RING finger protein 81)
443	1	CI	ENSP00000364118<br>ENSG00000120458	486<br>256<br>480<br>486<br>479<br>-<br>-<br>-<br>-<br>-	ylgiaevrtlQQCLFLHFqantktfskd<br>ylgiaevrtlQQCLFLHFqantktfskd<br>ylgiaevrtlQQCLFLHFqantktfskd<br>ylgiaevrtlQQCLFLHFqanaktfske<br>ylgiaevrtlQQCLFLHFqantktfske<br>............................<br>............................<br>............................<br>............................<br>............................	Hs_ENSP00000364118<br>Bt_ENSBTAP00000014937<br>Rn_ENSRNOP00000013370<br>Mm_ENSMUSP00000043329<br>Gg_ENSGALP00000033978<br>Xt<br>Dr<br>Ce<br>Dm<br>Sc	Uncharacterized protein C11orf61
444	1	CI	ENSP00000254806<br>ENSG00000132471	68<br>68<br>68<br>68<br>68<br>68<br>68<br>68<br>66<br>-	flsk-gkdamQSFMMPFYlmkdceikqp<br>flsk-ardamQSFMMPFYlmkdceikqp<br>flsk-gkdamrsfmmpfylmkdcevkqp<br>flsk-gkdamQSFMMPFYlmkdceikqp<br>fvsk-gkdalqsfgmafyllkdyeikqp<br>fvsk-grdpmQSFMMPFYlmkdceikqp<br>fltk-gkdplQSFMMPFYlmkgcevkqp<br>fmneskkdefksfampfnsvrdvkleqp<br>fnskkssdsmqsfsapfvalsdveieqp<br>............................	Hs_ENSP00000254806<br>Bt_ENSBTAP00000011385<br>Rn_ENSRNOP00000010826<br>Mm_ENSMUSP00000102054<br>Gg_ENSGALP00000003470<br>Xt_ENSXETP00000027547<br>Dr_ENSDARP00000089702<br>Ce_CE04043<br>Dm_FBpp0075666<br>Sc	NP_036610 WW domain-binding protein 2 (WBP-2)
445	1	C	ENSP00000261461<br>ENSG00000066027	77<br>80<br>77<br>81<br>67<br>66<br>69<br>-<br>-<br>-	qqelfcqklqQCCILFDFmdsvsdlksk<br>qqelfcqklqQCCILFDFmdsvsdlksk<br>qqelfcqklqqccvlfdfmdsvsdlksk<br>qqelfcqklqqccvlfdfmdsvsdlksk<br>qqdlfcqklqqccvlfdfmdsvsdlkgk<br>qqdlfcqklqqccvlfdfmdsisdlksk<br>qhelfmqklqqccklfdfydtvtdlksk<br>............................<br>............................<br>............................	Hs_ENSP00000261461<br>Bt_ENSBTAP00000038304<br>Rn_ENSRNOP00000000078<br>Mm_ENSMUSP00000106510<br>Gg_ENSGALP00000015963<br>Xt_ENSXETP00000031452<br>Dr_ENSDARP00000019419<br>Ce<br>Dm<br>Sc	NP_006234 Serine/threonine-protein phosphatase 2A 56 kDa regulatory subunit alpha isoform (PP2A, B subunit, B' alpha isoform)(PP2A, B subunit, B56 alpha isoform)(PP2A, B subunit, PR61 alpha isoform)(PP2A, B subunit, R5 alpha isoform)
446	1	CI	ENSP00000371514<br>ENSG00000168263	135<br>147<br>142<br>143<br>95<br>45<br>118<br>-<br>-<br>-	rlatstsrsrQLSLCDDYeeqtdeyffd<br>rlatstsrsrQLGLCDDYevqtdeyffd<br>rlvtstsrncQLGLCDDYeaktdeyffd<br>rlatsttrrgQLGLCDDYeaqtdeyffd<br>rlatstdrrcQLGLCDDYaaqvdeyffd<br>rlatytdrqrkldlcddyspkedvyffd<br>rlamstdlnmkmdlcddyvvkdneyffd<br>............................<br>............................<br>............................	Hs_ENSP00000371514<br>Bt_ENSBTAP00000023770<br>Rn_ENSRNOP00000016757<br>Mm_ENSMUSP00000055091<br>Gg_ENSGALP00000016520<br>Xt_ENSXETP00000025601<br>Dr_ENSDARP00000085905<br>Ce<br>Dm<br>Sc	NP_598004 Potassium voltage-gated channel subfamily V member 2 (Voltage-gated potassium channel subunit Kv8.2)
447	1	CI	ENSP00000251020<br>ENSG00000103449	718<br>710<br>709<br>717<br>703<br>769<br>688<br>-<br>836<br>-	ciichrvlscQSALKMHYrthtgerpfk<br>ciichrvlscQSALKMHYrthtgerpfk<br>cvichrvlscQSALKMHYrthtgerpfk<br>ciichrvlscQSALKMHYrthtgerpfk<br>ciichrvlscQSALKMHYrthtgerpfk<br>cvicrrvlscQSALKMHYrthtgerpfk<br>cvichrilscQSALKMHYrthtgerpfk<br>............................<br>cvvcdrvlscksalqmhyrthtgerpfk<br>............................	Hs_ENSP00000251020<br>Bt_ENSBTAP00000011264<br>Rn_ENSRNOP00000018628<br>Mm_ENSMUSP00000034090<br>Gg_ENSGALP00000005923<br>Xt_ENSXETP00000039895<br>Dr_ENSDARP00000047181<br>Ce<br>Dm_FBpp0088852<br>Sc	NP_002959 Sal-like protein 1 (Zinc finger protein SALL1)(Spalt-like transcription factor 1)(HSal1)
447	2	CI	ENSP00000251020<br>ENSG00000103449	1013<br>1005<br>1004<br>1012<br>998<br>1064<br>984<br>-<br>1115<br>-	cdicgktfacQSALDIHYrshtkerpfi<br>cdicgktfacQSALDIHYrshtkerpfi<br>cdicgktfacQSALDIHYrshtkerpfi<br>cdicgktfacQSALDIHYrshtkerpfi<br>cdicgktfacQSALDIHYrshtkerpfi<br>cdicgktfacQSALDIHYrshtkerpfi<br>cdicgktfacQSALDIHYrshtkerpfi<br>............................<br>kpvsppslprspsgsshasaniltsplp<br>............................	Hs_ENSP00000251020<br>Bt_ENSBTAP00000011264<br>Rn_ENSRNOP00000018628<br>Mm_ENSMUSP00000034090<br>Gg_ENSGALP00000005923<br>Xt_ENSXETP00000039895<br>Dr_ENSDARP00000047181<br>Ce<br>Dm_FBpp0088852<br>Sc	NP_002959 Sal-like protein 1 (Zinc finger protein SALL1)(Spalt-like transcription factor 1)(HSal1)
448	1	CI	ENSP00000344504<br>ENSG00000189060	51<br>51<br>-<br>-	nragssrqsiQKYIKSHYkvgen---ad<br>nragssrqsiQKYIKSHYkvgen---ad<br>............................<br>............................	Hs_ENSP00000344504<br>Bt_ENSBTAP00000049194<br>Rn<br>Mm	NP_005309 Histone H1.0 (Histone H1(0))(Histone H1')

				52<br>51<br>50<br>64<br>-<br>70	srggssrqsiQKYIKSHYkvghn---ad<br>srsgssrqsiQKYIKNHYkvgen---ad<br>srggasrqsiqkyvkhhykvgdn---ad<br>drkgaskqailkfisqnyklgdnviqin<br>............................<br>erkgssrpalkkfikenypivgsasnfd	Gg_ENSGALP00000020094<br>Xt_ENSXETP00000034845<br>Dr_ENSDARP00000056285<br>Ce_CE12450<br>Dm<br>Sc_YPL127C	
449	1	C	ENSP00000317123<br>ENSG00000144028	2089<br>2089<br>2092<br>2089<br>-<br>2089<br>-<br>2091<br>2093<br>2125	krltlqqkak---vkldfvapatgahny<br>krltlqqkak---vkldfvapatgahny<br>krltlqqkak---vkldfvapatgghny<br>krltlqqkak---vkldfvapatgghny<br>............................<br>krltlqqkakkdfgkldfvapatgnhny<br>............................<br>krlvinekss---vqldfaaprpghhkf<br>krltlqqkak---vkldfvapspgkhdy<br>kkvtlnketqq--yelefdtptsgkhnl	Hs_ENSP00000317123<br>Bt_ENSBTAP00000001130<br>Rn_ENSRNOP00000048598<br>Mm_ENSMUSP00000099509<br>Gg<br>Xt_ENSXETP00000002684<br>Dr<br>Ce_CE21971<br>Dm_FBpp0075282<br>Sc_YER172C	NP_054733 U5 small nuclear ribonucleoprotein 200 kDa helicase (EC 3.6.1.-)(U5 snRNP-specific 200 kDa protein)(U5-200KD)(Activating signal cointegrator 1 complex subunit 3-like 1)(BRR2 homolog)
450	1	C	ENSP00000361154<br>ENSG00000132763	98<br>98<br>98<br>98<br>95<br>41<br>102<br>115<br>-<br>-	--vreslpelQIEIIADYevhpnrrpki<br>--vreslpelqieviadyevhpnrrpki<br>--vtekfpelhieviadyevhpnrrpki<br>--vtekfpevhmeviadyevhpnrrpki<br>--vkeifpdqkvdvifdyeilpsrrpkf<br>--vkekypsgkldviydyelhpnrrpkv<br>c-islcfanqfvdvsydyemlpsrkpkf<br>mseklrkveenfeilhdysmtpqrrpki<br>............................<br>............................	Hs_ENSP00000361154<br>Bt_ENSBTAP00000022638<br>Rn_ENSRNOP00000023178<br>Mm_ENSMUSP00000030453<br>Gg_ENSGALP00000016616<br>Xt_ENSXETP00000007572<br>Dr_ENSDARP00000064426<br>Ce_CE34461<br>Dm<br>Sc	NP_056321 Methylmalonic aciduria and homocystinuria type C protein
451	1	CI	ENSP00000249601<br>ENSG00000128805	674<br>532<br>626<br>678<br>575<br>-<br>523<br>-<br>-<br>-	edaerrnqllQREMEEFFstlgsltvga<br>edaekrnqllQREMEEFFstlgsltvga<br>edaerrnqllQREMEEFFatlgsltagt<br>edaerrnqllQREMEEFFstlgslttgt<br>edaekrnhllQKEMEEFFstlgcltggs<br>............................<br>edaetrnrllQNEMEEFFstlgdltlgt<br>............................<br>............................<br>............................	Hs_ENSP00000249601<br>Bt_ENSBTAP00000010363<br>Rn_ENSRNOP00000031083<br>Mm_ENSMUSP00000107587<br>Gg_ENSGALP00000009890<br>Xt<br>Dr_ENSDARP00000012969<br>Ce<br>Dm<br>Sc	NP_067049 Rho GTPase-activating protein 22 (Rho-type GTPase-activating protein 22)
452	1	CI	ENSP00000351539<br>ENSG00000179456	395<br>395<br>391<br>395<br>386<br>316<br>401<br>-<br>-<br>-	nkvfpsphilQIHLSTHFreqdgirskp<br>nkvfpsphilQIHLSTHFreqdglrskp<br>nkvfpsphilQIHLSTHFreqdgirskp<br>nkvfpsphilQIHLSTHFreqdgirskp<br>nkvfpsphilQIHLSTHFreqdgirskp<br>----------------------------<br>nkvfpsphilQIHLSTHFreqegvrakp<br>............................<br>............................<br>............................	Hs_ENSP00000351539<br>Bt_ENSBTAP00000000497<br>Rn_ENSRNOP00000005849<br>Mm_ENSMUSP00000091831<br>Gg_ENSGALP00000017393<br>Xt_ENSXETP00000031817<br>Dr_ENSDARP00000056134<br>Ce<br>Dm<br>Sc	NP_006343 Zinc finger protein 238 (Transcriptional repressor RP58)(58 kDa repressor protein)(Zinc finger protein C2H2-171)(Translin-associated zinc finger protein 1)(TAZ-1)(Zinc finger and BTB domain-containing protein 18)
453	1	CI	ENSP00000310431<br>ENSG00000140506	462<br>439<br>439<br>440<br>-<br>-<br>-<br>-<br>-<br>-	gqpprassclQPGIFLFYlliqtvgffg<br>grsprvssclQPGIFLFFlfiqtvgffc<br>gwlpgsstclrtsiflffllliqtvgffc<br>gwllgsstclhtsiflffllllqtvgffc<br>............................<br>............................<br>............................<br>............................<br>............................<br>............................	Hs_ENSP00000310431<br>Bt_ENSBTAP00000028561<br>Rn_ENSRNOP00000026192<br>Mm_ENSMUSP00000091352<br>Gg<br>Xt<br>Dr<br>Ce<br>Dm<br>Sc	NP_068591 Protein ERGIC-53-like Precursor (ERGIC53-like protein)(Lectin mannose-binding 1-like)(LMAN1-like protein)
454	1	CI	ENSP00000318262<br>ENSG00000121749	575<br>559<br>558<br>558<br>556<br>-<br>553<br>-<br>-<br>-	lccailesekQQIMEKHYgfneilkhin<br>lccailesekQQIMEKHYgfneilkhin<br>lccailesekQQIMEKHYgfneilkhin<br>lccailesekQQIMAKHYgfneilkhin<br>lccailesekqqimekqygfneilkhin<br>............................<br>vccaildsekqkimdrkygfneilkhin<br>............................<br>............................<br>............................	Hs_ENSP00000318262<br>Bt_ENSBTAP00000030401<br>Rn_ENSRNOP00000005207<br>Mm_ENSMUSP00000020339<br>Gg_ENSGALP00000016547<br>Xt<br>Dr_ENSDARP00000074271<br>Ce<br>Dm<br>Sc	NP_073608 TBC1 domain family member 15
455	1	CI	ENSP00000283684<br>ENSG00000081923	917<br>917<br>917<br>917	gvgisgqegmQAVMSSDYsfaqfrylqr<br>gvgisgqegmQAVMSSDYsfaqfrylqr<br>gvgisgqegmQAVMSSDYsfaqfrylqr<br>gvgisgqegmQAVMSSDYsfaqfrylqr	Hs_ENSP00000283684<br>Bt_ENSBTAP00000007542<br>Rn_ENSRNOP00000038207<br>Mm_ENSMUSP00000025482	NP_005594 Probable phospholipid-transporting ATPase IC (EC 3.6.3.1)

				921	gvgisgqegmQAVMSSDYsfgqfrylqr	Gg_ENSGALP00000005136	(Familial intrahepatic cholestasis type 1)(ATPase class I type 8B member 1)
				736	gvgisgqegmQAVMSSDYsfaqfrylqr	Xt_ENSXETP00000045089	
				-	....	Dr	
				-	....	Ce	
				-	....	Dm	
				-	....	Sc	
456	1	CI	ENSP00000362296 ENSG00000196767	35	mqqgspfrnpQKLLQSDYlqgvpsnghp	Hs_ENSP00000362296	NP_000298 POU domain, class 3, transcription factor 4 (Brain-specific homeobox/POU domain protein 4)(Brain-4)(Brn-4)
				36	mqqgspfrnpQKLLQSDYlqgvpsnghp	Bt_ENSBTAP00000020869	
				35	mqqgspfrnpQKLLQSDYlqgvpsnghp	Rn_ENSRNOP00000003724	
				35	mqqgspfrnpQKLLQSDYlqgvpsnghp	Mm_ENSMUSP00000077354	
				-	....	Gg	
				34	mqqgspfrnpQKLLQSDYlqgvpsnghp	Xt_ENSXETP00000031753	
				-	....	Dr	
				-	....	Ce	
				-	....	Dm	
				-	....	Sc	
457	1	CI	ENSP00000349708 ENSG00000163867	1131	slinelnlslQGTLTTFFnlcnkidvfk	Hs_ENSP00000349708	NP_009098 Zinc finger MYM-type protein 6 (Zinc finger protein 258)
				975	----------------------------	Bt_ENSBTAP00000029263	
				1055	slinklnsslQGTMTTFFslynkveifk	Rn_ENSRNOP00000019135	
				1055	slinklnsslQGTMTTFFnlynkvdvfq	Mm_ENSMUSP00000045366	
				-	....	Gg	
				-	....	Xt	
				-	....	Dr	
				-	....	Ce	
				-	....	Dm	
				-	....	Sc	
458	1	CI	ENSP00000304500 ENSG00000166377	898	gigiegkegkQASLAADFsitqfrhigr	Hs_ENSP00000304500	NP_940933 Probable phospholipid-transporting ATPase IIB (EC 3.6.3.1)
				898	gigiegkegrQASLAADFsitrfkhvgr	Bt_ENSBTAP00000010477	
				898	gigiegkegkQASLAADFsitqfrhigr	Rn_ENSRNOP00000040756	
				897	gigiegkegkQASLAADFsitqfrhigr	Mm_ENSMUSP00000025458	
				892	gigiegkegkQASLAADFsitqfkhigr	Gg_ENSGALP00000020629	
				823	gigiegkegkQASLAADFsitqfkhigr	Xt_ENSXETP00000001891	
				870	gigiegkegkQASLAADFsitqfkhigr	Dr_ENSDARP00000084909	
				837	gigidanegkQASLAADFsitqfshvcr	Ce_CE34183	
				1019	gvgiegregrQASLAGDFsipqfshiak	Dm_FBpp0080040	
				906	gvgivgkegkQASLAADFsitqfchlte	Sc_YIL048W	
459	1	CI	ENSP00000383376 ENSG00000154721	76	rs--vsfvyyQQTLQGDFknraemidfn	Hs_ENSP00000383376	Junctional adhesion molecule B Precursor (JAM-B)(Junctional adhesion molecule 2)(Vascular endothelial junction-associated molecule)(VE-JAM)(CD322 antigen)
				76	rg--vsfvyyQQALQGDFkdraemidfs	Bt_ENSBTAP00000000795	
				-	....	Rn	
				77	qg--vslvyyQQALQGDFkdraemidfn	Mm_ENSMUSP00000109833	
				74	sq-evsfvyyngeftgdlkdraemlntg	Gg_ENSGALP00000025347	
				32	pngdisfiyynntlaadlrgraemiess	Xt_ENSXETP00000029831	
				70	kekdvsfvyygerfvgpfqdradiegat	Dr_ENSDARP00000076426	
				-	....	Ce	
				-	....	Dm	
				-	....	Sc	
460	1	CI	ENSP00000304502 ENSG00000170577	85	fsphnhaklqQLWLKAHYieaeklrgrp	Hs_ENSP00000304502	NP_058628 Homeobox protein SIX2 (Sine oculis homeobox homolog 2)
				85	fsphnhaklqQLWLKAHYieaeklrgrp	Bt_ENSBTAP00000005445	
				85	fsphnhaklqQLWLKAHYieaeklrgrp	Rn_ENSRNOP00000034707	
				85	fsphnhaklqQLWLKAHYieaeklrgrp	Mm_ENSMUSP00000024947	
				-	....	Gg	
				-	....	Xt	
				85	fsphnhpklqQLWLKAHYieaeklrgrp	Dr_ENSDARP00000075251	
				-	....	Ce	
				-	....	Dm	
				-	....	Sc	
461	1	CI	ENSP00000300035 ENSG00000166803	62	pvcvrptpkwQKGIGEFFr-lspkdsek	Hs_ENSP00000300035	NP_055551 PCNA-associated factor (p15PAF)(Overexpressed in anaplastic thyroid carcinoma 1)(OEATC-1)(Hepatitis C virus NS5A-transactivated protein 9)(HCV NS5A-transactivated protein 9)
				-	....	Bt	
				61	pvcvrptpkwQKGIGEFFr-lspkdskk	Rn_ENSRNOP00000022256	
				61	pvcvrptpkwQKGIGEFFr-lspkeskk	Mm_ENSMUSP00000038877	
				-	....	Gg	
				68	pvcvrptptwQKGIGEFFgspstsqpek	Xt_ENSXETP00000057278	
				-	....	Dr	
				-	....	Ce	
				-	....	Dm	
				-	....	Sc	
462	1	CI	ENSP00000300404 ENSG00000167080	550	tyrsntltrvQFKLALHYfknhlqcaa-	Hs_ENSP00000300404	NP_703147 Beta-1,4 N-acetylgalactosaminyltransferase 2 (EC 2.4.1.-)(Sd(a) beta-1,4-
				494	iyrantndkiQFKLALHYfknhlqcat-	Bt_ENSBTAP00000013010	
				-	....	Rn	
				494	kyrantnsviqfkvalqyfknhlycst-	Mm_ENSMUSP00000037239	

				-	....	Gg	GalNAc transferase)(UDP-GalNAc:Neu5Aca2-3Galb-R b1,4-N-acetylgalactosaminyltransferase)
				-	....	Xt	
				-	....	Dr	
				-	....	Ce	
				-	....	Dm	
				-	....	Sc	
463	1	CI	ENSP00000321195<br>ENSG00000058063	364	pislyvtvemQKFLGSFFigwdldlyhe	Hs_ENSP00000321195	NP_055431 Probable phospholipid-transporting ATPase IF (EC 3.6.3.1)(ATPase class I type 11B)(ATPase IR)
				355	pislyvtvelQKFLGSFFigwdldlyhe	Bt_ENSBTAP00000032012	
				364	pislyvtvemQKFLGSFFigwdldlyhe	Rn_ENSRNOP00000048475	
				364	pislyvtvemQKFLGSFFigwdldlyhe	Mm_ENSMUSP00000029257	
				364	pislyvtvemQKFLGSFFigwdldlyhe	Gg_ENSGALP00000014347	
				355	pislyvtvemQKFLGSFFigwdldlyhe	Xt_ENSXETP00000036663	
				288	pislyvtvelQKFLGSFFigwdldlyhe	Dr_ENSDARP00000070629	
				-	....	Ce	
				389	pislyvtielqrvigswfmewdlelyen	Dm_FBpp0074048	
				-	....	Sc	
464	1	CI	ENSP00000298834<br>ENSG00000110497	506	nnsgsirhelQCDLRRFFleydrlqeld	Hs_ENSP00000298834	activating molecule in beclin-1-regulated autophagy
				-	....	Bt	
				501	nnsgnirhelQCDLRRFFleydrlqeld	Rn_ENSRNOP00000039742	
				501	nnsgsirhelQCDLRRFFleydrlqeld	Mm_ENSMUSP00000049258	
				507	snpasirnelQCDLRRFFleydrlqeld	Gg_ENSGALP00000013594	
				503	vhptttrtelQCDLRRFFleydrlhele	Xt_ENSXETP00000054310	
				468	sssssihsvlrcnlyryfmdyegtqdtv	Dr_ENSDARP00000019906	
				-	....	Ce	
				-	....	Dm	
				-	....	Sc	
465	1	C	ENSP00000356779<br>ENSG00000117477	188	nernmfgklsQLCISNDFgqedvllslt	Hs_ENSP00000356779	Uncharacterized protein C1orf114
				188	nesnvsgklsqlcisnefrqenvilslt	Bt_ENSBTAP00000027004	
				188	netnmsgklsqlcisgdlgqesmlmsvt	Rn_ENSRNOP00000003844	
				188	netnmsgklsqlcisgdleqesvlvsvt	Mm_ENSMUSP00000027867	
				202	gggdvstklsqlhisnetgpesaslslp	Gg_ENSGALP00000024519	
				181	adeeivdglsqlhlsnatgtnehrd--d	Xt_ENSXETP00000022502	
				-	....	Dr	
				-	....	Ce	
				-	....	Dm	
				-	....	Sc	
466	1	CI	ENSP00000215904<br>ENSG00000221912	275	qayla---agQHDLVPHYyvesiadlte	Hs_ENSP00000215904	NP_064711 Pyridoxal phosphate phosphatase (PLP phosphatase)(EC 3.1.3.74)
				275	qayla---agQHDLVPHYyvesiadlme	Bt_ENSBTAP00000016505	
				271	qayla---agQHDLVPHYyvesiadlme	Rn_ENSRNOP00000050300	
				271	qaylt---agQRDLVPHYyvesiadlme	Mm_ENSMUSP00000086796	
				216	laymasdsaaakdmvpnyyvnsiadlip	Gg_ENSGALP00000020277	
				-	....	Xt	
				283	qryrdsqspeqkdcapdfvvesvadflq	Dr_ENSDARP00000055881	
				-	....	Ce	
				-	....	Dm	
				-	....	Sc	
467	1	C	ENSP00000373718<br>ENSG00000164068	31	eksrvtg-ivQEKLLNDYlnr---ifss	Hs_ENSP00000373718	E3 ubiquitin-protein ligase RNF123 (EC 6.3.2.-)(RING finger protein 123)(Kip1 ubiquitination-promoting complex protein 1)
				31	ersrvtg-ivhekllsdylhr---vfas	Bt_ENSBTAP00000042771	
				31	eksrvtg-ivQEKLLSDYlyr---ifss	Rn_ENSRNOP00000049507	
				31	eksrvtg-ivQEKLLSDYlyr---ifsp	Mm_ENSMUSP00000040803	
				32	ekskvaegivnsrllndylhr---vfss	Gg_ENSGALP00000003908	
				31	tnrklcs-ivnerllnlylqr---ifpa	Xt_ENSXETP00000013085	
				0	----------------------------	Dr_ENSDARP00000044428	
				-	....	Ce	
				25	ifeqvdhdnlysdldddlegancstvpd	Dm_FBpp0082488	
				-	....	Sc	
468	1	CI	ENSP00000384801<br>ENSG00000126773	63	vwggvgtllyQLGILKDYytaalsgglm	Hs_ENSP00000384801	NP_071940 Pecanex-like protein C14orf135 (Hepatitis C virus F protein-binding protein 2)(HCV F protein-binding protein 2)
				66	ilggigtllyQLGILEDYhtaalsgglm	Bt_ENSBTAP00000018737	
				66	alggtgtllyQLDILRDYtaaalsgglm	Rn_ENSRNOP00000007369	
				66	alggtgtllyQLDILRDYtaaalsgglm	Mm_ENSMUSP00000038916	
				64	vlggvgtllyQLDIMKDYytaalsgglm	Gg_ENSGALP00000019425	
				63	llggvgtvlgevkvlqdyytavfsgglm	Xt_ENSXETP00000036936	
				63	llggigtllyqlrvldeafagivsgalm	Dr_ENSDARP00000080453	
				-	....	Ce	
				-	....	Dm	
				-	....	Sc	
469	1	CI	ENSP00000367046<br>ENSG00000136122	157	ridkdvedkrQKAIEEFFtkdvivpspw	Hs_ENSP00000367046	NP_079084 Protein aurora borealis (HsBora)
				159	ridkdvedkrQKAIEEFFtkdvivpspw	Bt_ENSBTAP00000026496	
				97	ridkdvedkrQKAIEEFFtkdvivpspw	Rn_ENSRNOP00000035098	
				97	ridkdvedkrQKAIEEFFtkdvivpspw	Mm_ENSMUSP00000022656	

				90<br>93<br>97<br>-<br>144<br>-	ridketeerrQKAIEEFFtkslivpspw<br>ktdketeerrQKAIEEFFtkrtivpspw<br>radseieekrQHAIEQFFtkgaivpspw<br>............................<br>spdpeqeskaQLAISAFFkeslivpspv<br>............................	Gg_ENSGALP00000035725<br>Xt_ENSXETP00000027003<br>Dr_ENSDARP00000062627<br>Ce<br>Dm_FBpp0074874<br>Sc	
470	1	CI	ENSP00000278483<br>ENSG00000149196	143<br>143<br>143<br>143<br>143<br>143<br>143<br>118<br>143<br>182	--------ftQKMLDNFYnfassfavsq<br>--------ftQKMLDNFYnfassfavsq<br>--------ftQKMLDNFYnfassfalsq<br>--------ftQKMLDNFYnfassfalsq<br>--------ftQKMLDNFYnfassfavtq<br>--------ftQKMLDNFYnfatsfavsq<br>--------ftqkmldslfnftssfalsq<br>--------fdn-qmqlyssgsaqigina<br>--------fgQRMLENFFnyassfgvaa<br>lgiirnittagqlaqvypsltqelaaki	Hs_ENSP00000278483<br>Bt_ENSBTAP00000026632<br>Rn_ENSRNOP00000023468<br>Mm_ENSMUSP00000077951<br>Gg_ENSGALP00000002814<br>Xt_ENSXETP00000022906<br>Dr_ENSDARP00000059340<br>Ce_CE34559<br>Dm_FBpp0072703<br>Sc_YOL032W	NP_057485 Uncharacterized protein C11orf73
471	1	CI	ENSP00000353362<br>ENSG00000141837	228<br>232<br>-<br>230<br>-<br>-<br>222<br>310<br>171<br>-	simkamipllQIGLLLFFailifaiigl<br>simkamipllQIGLLLFFailifaiigl<br>............................<br>simkamipllQIGLLLFFailifaiigl<br>............................<br>............................<br>simkamipllQIGLLLFFailmfaiigl<br>silcamapllqigllvlfaiiifaiigl<br>siikamapllqigllvlfaivifaiigl<br>............................	Hs_ENSP00000353362<br>Bt_ENSBTAP00000019752<br>Rn<br>Mm_ENSMUSP00000112436<br>Gg<br>Xt<br>Dr_ENSDARP00000043134<br>Ce_CE42155<br>Dm_FBpp0099702<br>Sc	NP_001120694 Voltage-dependent P/Q-type calcium channel subunit alpha-1A (Voltage-gated calcium channel subunit alpha Cav2.1)(Calcium channel, L type, alpha-1 polypeptide isoform 4)(Brain calcium channel I)(BI)
472	1	C	ENSP00000353731<br>ENSG00000197635	527<br>526<br>503<br>521<br>522<br>482<br>-<br>-<br>500<br>574	kf------wyQMILPPHFdk--skkypl<br>kl------wylcilvphfpy--skeypm<br>rf------wyQMILPPHFdk--skkypl<br>rf------wyQMILPPHFdk--skkypl<br>nl------wyqmilpphlds--skkypl<br>el------wyqltlppnfdk--skkypl<br>............................<br>............................<br>sig-----yaklalppnfve--tkkypl<br>efgkdilvnsyeilpndfdetlsdhypv	Hs_ENSP00000353731<br>Bt_ENSBTAP00000049191<br>Rn_ENSRNOP00000045536<br>Mm_ENSMUSP00000044050<br>Gg_ENSGALP00000018078<br>Xt_ENSXETP00000035347<br>Dr<br>Ce<br>Dm_FBpp0078771<br>Sc_YHR028C	NP_001926 Dipeptidyl peptidase 4 (EC 3.4.14.5)(Dipeptidyl peptidase IV)(DPP IV)(T-cell activation antigen CD26)(TP103)(Adenosine deaminase complexing protein 2)(ADABP)(CD26 antigen)
473	1	CI	ENSP00000264350<br>ENSG00000138646	484<br>499<br>-<br>-<br>-<br>-<br>-<br>-<br>-<br>-	lkrlpfhsppQEALEIFFllpecpmmhi<br>lknlpfnsphQEALEVFFllpecpvmhd<br>............................<br>............................<br>............................<br>............................<br>............................<br>............................<br>............................<br>............................	Hs_ENSP00000264350<br>Bt_ENSBTAP00000027367<br>Rn<br>Mm<br>Gg<br>Xt<br>Dr<br>Ce<br>Dm<br>Sc	NP_057407 Probable E3 ubiquitin-protein ligase HERC5 (EC 6.3.2.-)(HECT domain and RCC1-like domain-containing protein 5)(Cyclin-E-binding protein 1)
474	1	CI	ENSP00000380708<br>ENSG00000082213	356<br>299<br>347<br>355<br>373<br>281<br>347<br>-<br>376<br>-	-evpdyemvhQAGLTCDYselphhiste<br>----------------------------<br>-evpdyemvhQAGLTCDYselphhiste<br>-evpdyemvhQAGLTCDYselphhiste<br>-espdyemvhQAGLTCDYvelphhvstk<br>-eapdyemihQAGLTCDYaelphhvste<br>-sppdyemvhQAGLTCDYselphhissd<br>............................<br>ddeidwhlifdsgsttdnnglphhisia<br>............................	Hs_ENSP00000380708<br>Bt_ENSBTAP00000023356<br>Rn_ENSRNOP00000032531<br>Mm_ENSMUSP00000055656<br>Gg_ENSGALP00000021023<br>Xt_ENSXETP00000012906<br>Dr_ENSDARP00000004506<br>Ce<br>Dm_FBpp0074793<br>Sc	NP_060826 UPF0489 protein C5orf22
475	1	CI	ENSP00000370713<br>ENSG00000185736	568<br>530<br>575<br>574<br>574<br>522<br>546<br>-<br>-<br>-	kiarwnvlglQGALLSHFvepvylqsiv<br>kiarwnvlglQGALLCHFiepvylhsiv<br>kiaswnvlglQGALLCHFiepvylhsii<br>kiaswnvlglQGALLCHFiepvylhsii<br>kiarwnilglqgallssfiepmylhsiv<br>kitrwnilglQGALLSHFidpiylqsii<br>kisrwnvlglQGALLSHFvepvylyslt<br>............................<br>............................<br>............................	Hs_ENSP00000370713<br>Bt_ENSBTAP00000018870<br>Rn_ENSRNOP00000047468<br>Mm_ENSMUSP00000064775<br>Gg_ENSGALP00000010979<br>Xt_ENSXETP00000039188<br>Dr_ENSDARP00000097291<br>Ce<br>Dm<br>Sc	NP_061172 Double-stranded RNA-specific editase B2 (EC 3.5.-.-)(dsRNA adenosine deaminase B2)(RNA-dependent adenosine deaminase 3)(RNA-editing deaminase 2)(RNA-editing enzyme 2)
476	1	CI	ENSP00000299198<br>ENSG00000166165	185<br>208<br>189<br>185	alksmteaeqQQLIDDHFlfdkpvspll<br>alksmteaeqQQLIDDHFlfdkpvspll<br>alksmteaeqQQLIDDHFlfdkpvspll<br>alksmteaeqQQLIDDHFlfdkpvspll	Hs_ENSP00000299198<br>Bt_ENSBTAP00000004079<br>Rn_ENSRNOP00000015122<br>Mm_ENSMUSP00000001304	NP_001814 Creatine kinase B-type (EC 2.7.3.2)(Creatine kinase B chain)(B-CK)

				193<br>185<br>185<br>183<br>385<br>-	alrnmtdaeqQQLIDDHFlfdkpvspll<br>alntmtdqeqQQLIDDHFlfdkpvspll<br>alkdmteeeqQQLIDDHFlfdkpvspll<br>pltgmdeetkkkliadhflfke-gdrfl<br>pltgmekavqQQLIDDHFlfke-gdrfl<br>............................	Gg_ENSGALP00000018743<br>Xt_ENSXETP00000049818<br>Dr_ENSDARP00000024900<br>Ce_CE06583<br>Dm_FBpp0076270<br>Sc	
477	1	C	ENSP00000278409<br>ENSG00000149133	24<br>24<br>-<br>-<br>-<br>-<br>-<br>-<br>-<br>-	llgladtlelQIILFLFFlviytltvlg<br>llgladtlelqailfslflviytltvvg<br>............................<br>............................<br>............................<br>............................<br>............................<br>............................<br>............................<br>............................	Hs_ENSP00000278409<br>Bt_ENSBTAP00000007721<br>Rn<br>Mm<br>Gg<br>Xt<br>Dr<br>Ce<br>Dm<br>Sc	NP_003688 Olfactory receptor 5F1 (Olfactory receptor OR11-167)(Olfactory receptor 11-10)(OR11-10)
478	1	CI	ENSP00000292090<br>ENSG00000160606	187<br>153<br>187<br>187<br>186<br>-<br>192<br>223<br>-<br>-	vmyflfrlapQAYLTHFFlryvnqr--t<br>----------------------------<br>vmyflfrlapQAYLTKFFlqyagqr--t<br>vmyflfrlapQAYLTKFFlqyagqr--t<br>vmyfafrlapqvyltwyfvryvevq--g<br>............................<br>vtyvtfrlgaqfyltwyltyhyssl--d<br>itlvvfrlivnvymiyfvfasfsmspwy<br>............................<br>............................	Hs_ENSP00000292090<br>Bt_ENSBTAP00000008914<br>Rn_ENSRNOP00000016840<br>Mm_ENSMUSP00000090556<br>Gg_ENSGALP00000006318<br>Xt<br>Dr_ENSDARP00000009170<br>Ce_CE33422<br>Dm<br>Sc	NP_612472 TLC domain-containing protein 1 Precursor
479	1	CI	ENSP00000354871<br>ENSG00000086717	56<br>55<br>47<br>56<br>58<br>-<br>-<br>-<br>-<br>-	qsieyadeqgQMQLSTFFsfmlenythi<br>qsieyadeqgQLQLSNFFsfmlenytnv<br>qsieyadeqgQMQLSSFFsfmlenytnt<br>qsieysdeqgQMQLSSFFsfmlenytkt<br>qsieyadeqeQTQLSNFFtfmlehcvhr<br>............................<br>............................<br>............................<br>............................<br>............................	Hs_ENSP00000354871<br>Bt_ENSBTAP00000012557<br>Rn_ENSRNOP00000037078<br>Mm_ENSMUSP00000071191<br>Gg_ENSGALP00000026611<br>Xt<br>Dr<br>Ce<br>Dm<br>Sc	NP_006231 Serine/threonine-protein phosphatase with EF-hands 1 (PPEF-1)(EC 3.1.3.16)(Protein phosphatase with EF calcium-binding domain)(PPEF)(Serine/threonine-protein phosphatase 7)(PP7)
479	2	CI	ENSP00000354871<br>ENSG00000086717	131<br>129<br>123<br>134<br>135<br>-<br>-<br>-<br>-<br>-	idllleafkeQQILHAHYvlevlfetkk<br>idllieafkqqqilhayyvlevlfetkk<br>inlllqafkqQQTLHAHYvlevlfeark<br>ihillqafkqQQILHAHYvlevlfeark<br>aktllnalkdrqllharyvlqllcetrr<br>............................<br>............................<br>............................<br>............................<br>............................	Hs_ENSP00000354871<br>Bt_ENSBTAP00000012557<br>Rn_ENSRNOP00000037078<br>Mm_ENSMUSP00000071191<br>Gg_ENSGALP00000026611<br>Xt<br>Dr<br>Ce<br>Dm<br>Sc	NP_006231 Serine/threonine-protein phosphatase with EF-hands 1 (PPEF-1)(EC 3.1.3.16)(Protein phosphatase with EF calcium-binding domain)(PPEF)(Serine/threonine-protein phosphatase 7)(PP7)
480	1	CI	ENSP00000364008<br>ENSG00000148123	42<br>42<br>42<br>42<br>44<br>-<br>-<br>-<br>-<br>-	ayyfectdtfQVHIQGFFcqdgdlmkpy<br>ayyfectdtfQVHIQGFFcqdgdlmkpy<br>ayyfectdtfQVHIQGFFcqdgdlmkpy<br>ayyfectdtfQVHIQGFFcqdgdlmkpy<br>ayyfectdtfQVHIQGFFcqdgnlmkpy<br>............................<br>............................<br>............................<br>............................<br>............................	Hs_ENSP00000364008<br>Bt_ENSBTAP00000004081<br>Rn_ENSRNOP00000009987<br>Mm_ENSMUSP00000075966<br>Gg_ENSGALP00000025008<br>Xt<br>Dr<br>Ce<br>Dm<br>Sc	NP_060223 Lipid phosphate phosphatase-related protein type 1 (Plasticity-related gene 3 protein)(PRG-3)
481	1	C	ENSP00000368109<br>ENSG00000205126	37<br>58<br>57<br>57<br>-<br>-<br>-<br>-<br>-<br>-	tqlleitlhlQQAMTEHFvqltsr---q<br>tqlmemvltlqqtvedhimqlttrpqqr<br>kilqekmlklqcvfmdhflqrtsq-qmq<br>kfvqekmlkfqhvirnqflqqisq-qmq<br>............................<br>............................<br>............................<br>............................<br>............................<br>............................	Hs_ENSP00000368109<br>Bt_ENSBTAP00000015998<br>Rn_ENSRNOP00000012214<br>Mm_ENSMUSP00000097281<br>Gg<br>Xt<br>Dr<br>Ce<br>Dm<br>Sc	NP_001027025 1-aminocyclopropane-1-carboxylate synthase-like protein 2
482	1	CI	ENSP00000381026<br>ENSG00000142185	380<br>383<br>379<br>379	pvsditisliQQKLSVFFqemfetftes<br>piseitisliQQKLSMFFqgmfetftes<br>pvseitisliQQKLSVFFqemfetften<br>pvseitisliQQKLSIFFqemfetften	Hs_ENSP00000381026<br>Bt_ENSBTAP00000029198<br>Rn_ENSRNOP00000001631<br>Mm_ENSMUSP00000020505	Transient receptor potential cation channel subfamily M member 2 (EC 3.6.1.13)(Long

				380<br>-<br>-<br>-<br>-<br>-	pvseitialirkklsvlfhdtyeqfteg<br>............................<br>............................<br>............................<br>............................<br>............................	Gg_ENSGALP00000009021<br>Xt<br>Dr<br>Ce<br>Dm<br>Sc	transient receptor potential channel 2)(LTrpC-2)(LTrpC2)(Transient receptor potential channel 7)(TrpC7)(Estrogen-responsive element-associated gene 1 protein)
483	1	CI	ENSP00000265081<br>ENSG00000113318	21<br>21<br>21<br>21<br>0<br>0<br>20<br>-<br>-<br>4	laasssaparQAVLSRFFqstgslksts<br>aapanpasarQAVLSRFFqstgslksta<br>snaagpdpgrQVVLSRFFksagslrssv<br>staagpgpgrQTVLSRFFrsagslrssa<br>----------------------------<br>----------------------------<br>asksskksstQTTISRFFtnasdraqpt<br>............................<br>............................<br>-------magQPTISRFFkkavkselth	Hs_ENSP00000265081<br>Bt_ENSBTAP00000010107<br>Rn_ENSRNOP00000041452<br>Mm_ENSMUSP00000022220<br>Gg_ENSGALP00000025089<br>Xt_ENSXETP00000049618<br>Dr_ENSDARP00000086717<br>Ce<br>Dm<br>Sc_YCR092C	NP_002430 DNA mismatch repair protein Msh3 (Divergent upstream protein)(DUP)(Mismatch repair protein 1)(MRP1)
484	1	CI	ENSP00000385989<br>ENSG00000178729	447<br>-<br>111<br>111<br>488<br>489<br>488<br>-<br>-<br>-	etnpyqhhfiQHFLREHYnewitniyvk<br>............................<br>etnpyqhhfiQHFLREHYnewitniyvk<br>etnpyqhhfiQHFLREHYnewitniyvk<br>etnpyqhhfiQHFLREHYnewitniyvk<br>epdpyenhfiQHFLREHYnewitntyvk<br>dsvpyqnhfiQHFLREHYtewitntyvk<br>............................<br>............................<br>............................	Hs_ENSP00000385989<br>Bt<br>Rn_ENSRNOP00000016940<br>Mm_ENSMUSP00000047640<br>Gg_ENSGALP00000026940<br>Xt_ENSXETP00000024731<br>Dr_ENSDARP00000002938<br>Ce<br>Dm<br>Sc	NP_001013754 Patched domain-containing protein C6orf138
485	1	CI	ENSP00000371483<br>ENSG00000157765	486<br>485<br>486<br>486<br>483<br>478<br>467<br>401<br>-<br>-	spgnalrsslQIALCHFFfnisgillwy<br>spgstlksslQIALCHFFfnisgiilwy<br>spgntlrsslQIALCHFFfnisgillwy<br>spgntlrsslQIALCHFFfnisgillwy<br>spgstlkyslQIALCHFFfnvsgiilfy<br>spgetlqnsvQIALCHFFfnisgiiiwy<br>spgetlanslQISLCHFFfniagillwy<br>tdpsrfekalhmamcqviyniigtclfy<br>............................<br>............................	Hs_ENSP00000371483<br>Bt_ENSBTAP00000002023<br>Rn_ENSRNOP00000006477<br>Mm_ENSMUSP00000092380<br>Gg_ENSGALP00000023177<br>Xt_ENSXETP00000002069<br>Dr_ENSDARP00000053526<br>Ce_CE31273<br>Dm<br>Sc	NP_006415 Sodium-dependent phosphate transport protein 2B (Sodium-phosphate transport protein 2B)(Na(+)-dependent phosphate cotransporter 2B)(Sodium/phosphate cotransporter 2B)(Na(+)/Pi cotransporter 2B)(NaPi-2b)(NaPi3b)(Solute carrier family 34 member 2)
486	1	CI	ENSP00000248846<br>ENSG00000128159	725<br>725<br>-<br>724<br>726<br>650<br>194<br>-<br>-<br>-	kdqerrqaarQEELDDDFsyarelrdre<br>kdqerrraarQEELDEDFsyarelrdre<br>............................<br>kdqerrlaarQEELDDDFsyarelrdre<br>kdqerrlairQEEIDDDFsyarelrere<br>kdqerrlaakqeeadddfsyareirdre<br>ldqewrstarrkeqeddfsyarelrdre<br>............................<br>............................<br>............................	Hs_ENSP00000248846<br>Bt_ENSBTAP00000000853<br>Rn<br>Mm_ENSMUSP00000104977<br>Gg_ENSGALP00000014085<br>Xt_ENSXETP00000048191<br>Dr_ENSDARP00000072923<br>Ce<br>Dm<br>Sc	NP_065194 Gamma-tubulin complex component 6 (GCP-6)
487	1	C	ENSP00000348573<br>ENSG00000127914	280<br>288<br>231<br>246<br>273<br>-<br>-<br>-<br>-<br>-	ilthqqqleeQDHLLEDYqkkkedftmq<br>ilthqqqleeQDHLLEDYhrkkedfkmq<br>iftlqqqlq-------dyqkkeedvqaq<br>iftqqqqlq-------dyqkkeedlqaq<br>ilshqqqledqecllknyqrkneefevq<br>............................<br>............................<br>............................<br>............................<br>............................	Hs_ENSP00000348573<br>Bt_ENSBTAP00000009791<br>Rn_ENSRNOP00000010118<br>Mm_ENSMUSP00000046129<br>Gg_ENSGALP00000015160<br>Xt<br>Dr<br>Ce<br>Dm<br>Sc	NP_005742 A-kinase anchor protein 9 (Protein kinase A-anchoring protein 9)(PRKA9)(A-kinase anchor protein 450 kDa)(AKAP 450)(A-kinase anchor protein 350 kDa)(AKAP 350)(hgAKAP 350)(AKAP 120-like protein)(Protein hyperion)(Protein yotiao)(Centrosome- and Golgi-localized PKN-associated protein)(CG-NAP)
488	1	CI	ENSP00000371897<br>ENSG00000100852	137<br>137<br>137<br>137<br>0<br>137<br>136<br>-	aeklmyictdQLGLEQDFeqkqmpegkl<br>aeklmyictdQLGLEQDFeqkqmpegkl<br>aeklmyictdQLGLEQDFeqkqmpegkl<br>aeklmyictdQLGLEQDFeqkqmpegkl<br>----------------------------<br>aeklmyictdQLGLEQDFeqkqmpegkl<br>aeklmyictdQLGLEQDFdqkqmpdgkl<br>............................	Hs_ENSP00000371897<br>Bt_ENSBTAP00000038500<br>Rn_ENSRNOP00000006241<br>Mm_ENSMUSP00000106353<br>Gg_ENSGALP00000016217<br>Xt_ENSXETP00000030817<br>Dr_ENSDARP00000081131<br>Ce	NP_001025226 Rho GTPase-activating protein 5 (Rho-type GTPase-activating protein 5)(p190-B)

				-<br>-	............................<br>............................	Dm<br>Sc	
489	1	CI	ENSP00000358529<br>ENSG00000134198	85<br>85<br>85<br>85<br>85<br>85<br>86<br>-<br>-<br>-	fgccgamresQCVLGSFFtcllvifaae<br>fgccgatresQCVLGSFFtcllvifaae<br>fgccgamresQCVLGSFFtcllvifaae<br>fgccgamresQCVLGSFFtcllvifaae<br>fgccgaaresQFLLGAFFacllvifaae<br>fgccgaaresQCLLGAFFacllvifaae<br>fgcfgavresQCLLGSFFaclllifgae<br>............................<br>............................<br>............................	Hs_ENSP00000358529<br>Bt_ENSBTAP00000021222<br>Rn_ENSRNOP00000033076<br>Mm_ENSMUSP00000029451<br>Gg_ENSGALP00000004028<br>Xt_ENSXETP00000036257<br>Dr_ENSDARP00000094156<br>Ce<br>Dm<br>Sc	NP_005716 Tetraspanin-2 (Tspan-2)
490	1	C	ENSP00000361001<br>ENSG00000159658	227<br>226<br>226<br>226<br>228<br>246<br>-<br>-<br>-<br>-	dehkktmellQSDMNQHFlketpgsnqi<br>dehkktvellqsdmnqhtlkesngsnqi<br>dehkatlellqgsv------etngsnqi<br>dehrttlgllqgsm------enngsnqi<br>eeykksleilqndv-----------kel<br>eeqkkkvetlqkdm--------------<br>............................<br>............................<br>............................<br>............................	Hs_ENSP00000361001<br>Bt_ENSBTAP00000000106<br>Rn_ENSRNOP00000013471<br>Mm_ENSMUSP00000074025<br>Gg_ENSGALP00000016989<br>Xt_ENSXETP00000035699<br>Dr<br>Ce<br>Dm<br>Sc	NP_055589 Uncharacterized calcium-binding protein KIAA0494
491	1	C	ENSP00000234389<br>ENSG00000116032	361<br>366<br>358<br>361<br>-<br>362<br>-<br>-<br>-<br>-	tgpvwvtgssQVHMSRHFkvwslrrdpr<br>tgpvwvtnssqvhisrrfrlwslrrdlr<br>tgavwvtgssqvhvsrhfkvwslrrdpl<br>tgavwvagssqvhvsrhfkvwslrrdpl<br>............................<br>tgqiyvsnn-lihtehhyriwslvrdsl<br>............................<br>............................<br>............................<br>............................	Hs_ENSP00000234389<br>Bt_ENSBTAP00000035962<br>Rn_ENSRNOP00000017064<br>Mm_ENSMUSP00000048576<br>Gg<br>Xt_ENSXETP00000046705<br>Dr<br>Ce<br>Dm<br>Sc	NP_619635 Glutamate
492	1	CI	ENSP00000224764<br>ENSG00000107779	304<br>306<br>304<br>304<br>305<br>-<br>307<br>-<br>-<br>-	adikgtgswtQLYLITDYhengslydfl<br>adikgtgswtQLYLITDYhengslydfl<br>adikgtgswtQLYLITDYhengslydfl<br>adikgtgswtQLYLITDYhengslydfl<br>adikgtgswtQLYLITDYhengslydfl<br>............................<br>adikgtgtftQLFLITDYhengslcdyl<br>............................<br>............................<br>............................	Hs_ENSP00000224764<br>Bt_ENSBTAP00000000282<br>Rn_ENSRNOP00000015047<br>Mm_ENSMUSP00000035900<br>Gg_ENSGALP00000003114<br>Xt<br>Dr_ENSDARP00000096398<br>Ce<br>Dm<br>Sc	NP_004320 Bone morphogenetic protein receptor type-1A Precursor (EC 2.7.11.30)(Serine/threonine-protein kinase receptor R5)(SKR5)(Activin receptor-like kinase 3)(ALK-3)(CD292 antigen)
493	1	CI	ENSP00000324820<br>ENSG00000160007	138<br>138<br>138<br>138<br>138<br>138<br>138<br>145<br>128<br>-	aeklmyfctdQLGLEQDFeqkqmpdgkl<br>aeklmyfctdQLGLEQDFeqkqmpdgkl<br>aeklmyfctdQLGLEQDFeqkqmpdgkl<br>aeklmyfctdQLGLEQDFeqkqmpdgkl<br>aeklmyfctdQLGLEQDFeqkqmpdgkl<br>aeklmyfctdQLGLEQDFeqkqmpegkl<br>aeklmyfctdQLGLEQDFeqkqmpegkl<br>rdklmyiqkeqlglesefpqqllpngkf<br>aeklmyicknqlgiekeyeqkvmpdgrl<br>............................	Hs_ENSP00000324820<br>Bt_ENSBTAP00000020651<br>Rn_ENSRNOP00000021223<br>Mm_ENSMUSP00000075242<br>Gg_ENSGALP00000019092<br>Xt_ENSXETP00000039627<br>Dr_ENSDARP00000085108<br>Ce_CE36176<br>Dm_FBpp0074255<br>Sc	Glucocorticoid receptor DNA-binding factor 1 (Glucocorticoid receptor repression factor 1)(GRF-1)(Rho GAP p190A)(p190-A)
494	1	CI	ENSP00000355938<br>ENSG00000162769	481<br>482<br>-<br>486<br>405<br>419<br>449<br>-<br>-<br>-	qifgilftlaQGKLTSDYg-pkagnifl<br>qifgilftlaqgkltsayg-prtgnvfl<br>............................<br>qilgifftlaQGKITTDYnspeagnifl<br>qifgivftlvQGKLTTDYs-pragnlfl<br>qifgilftlaQGKLTTDYn-pqagnifl<br>qvfgiiftliQGKLTTDYn-plsgnifl<br>............................<br>............................<br>............................	Hs_ENSP00000355938<br>Bt_ENSBTAP00000021251<br>Rn<br>Mm_ENSMUSP00000082777<br>Gg_ENSGALP00000015935<br>Xt_ENSXETP00000031412<br>Dr_ENSDARP00000041396<br>Ce<br>Dm<br>Sc	NP_054772 Feline leukemia virus subgroup C receptor-related protein 1 (Feline leukemia virus subgroup C receptor)(hFLVCR)
495	1	CI	ENSP00000266517<br>ENSG00000139163	401<br>352<br>312<br>312<br>313<br>-<br>309<br>292	eveilfiqvnQFALASHFfwglwaliqa<br>eveilfiqvnQFALASHFfwglwaliqa<br>evetlfiqvnQFALASHFfwglwaliqa<br>evetlfiqvnQFALASHFfwglwaliqa<br>evevlyvqvnQFALASHFfwglwaliqa<br>............................<br>evellyvqvnrfalashffwglwaliqa<br>riatmfknlllfeaaahlfwavwalvqa	Hs_ENSP00000266517<br>Bt_ENSBTAP00000007669<br>Rn_ENSRNOP00000020042<br>Mm_ENSMUSP00000032413<br>Gg_ENSGALP00000021538<br>Xt<br>Dr_ENSDARP00000019763<br>Ce_CE07553	NP_061108 Ethanolamine kinase 1 (EKI 1)(EC 2.7.1.82)

				464<br>-	evellyvqvnqfalashifwtvwsllqa<br>............................	Dm_FBpp0073994<br>Sc	
496	1	CI	ENSP00000362185<br>ENSG00000163873	727<br>727<br>727<br>727<br>699<br>-<br>-<br>-<br>-<br>-	alvknneegiQRALTADYallmesttie<br>alvknneegiQRTLTADYallmesttie<br>alvknneegiQRTLTADYallmesttie<br>alvknneegiQRTLTADYallmesttie<br>alvknneegiQRTLTADYallmesttie<br>............................<br>............................<br>............................<br>............................<br>............................	Hs_ENSP00000362185<br>Bt_ENSBTAP00000012214<br>Rn_ENSRNOP00000012065<br>Mm_ENSMUSP00000030676<br>Gg_ENSGALP00000003269<br>Xt<br>Dr<br>Ce<br>Dm<br>Sc	NP_000822 Glutamate receptor, ionotropic kainate 3 Precursor (Glutamate receptor 7)(GluR-7)(GluR7)(Excitatory amino acid receptor 5)(EAA5)
497	1	CI	ENSP00000239138<br>ENSG00000120063	339<br>-<br>259<br>339<br>339<br>339<br>339<br>-<br>419<br>-	cfrnkrrdqqQKPLYHHFttaintenir<br>............................<br>cfrgkrrdqqQRPLYHHFttaintenir<br>cfrgkrrdqqQRPLYHHFttaintenir<br>cfrtkrrdqqQKPLYHHFttaintenir<br>cfrnkrrdqqQKPLYHHFttainte-ir<br>cfrnkrrdqqQKPLYHHFttaintenir<br>............................<br>mfmsvrrsssisriyhhfttaidtrnin<br>............................	Hs_ENSP00000239138<br>Bt<br>Rn_ENSRNOP00000051938<br>Mm_ENSMUSP00000020930<br>Gg_ENSGALP00000006526<br>Xt_ENSXETP00000002710<br>Dr_ENSDARP00000036006<br>Ce<br>Dm_FBpp0110418<br>Sc	NP_006563 Guanine nucleotide-binding protein alpha-13 subunit (G alpha-13)
498	1	C	ENSP00000294664<br>ENSG00000162643	375<br>-<br>192<br>192<br>375<br>342<br>330<br>-<br>436<br>-	rvhfsgklllqpslilfwsfsdpihpql<br>............................<br>rvqnssrlllqpslilfwsfsdpihpql<br>rvqnsgrlllqpslllfwsfsdpihpql<br>rtk-----vqhdsviifwsffdpihpql<br>rvnisskillkpslilfwsftdplhpql<br>ridsstklllnpshilfwsfsdpinpql<br>............................<br>rvdyvqravlepnpvllwsfsdnlnykl<br>............................	Hs_ENSP00000294664<br>Bt<br>Rn_ENSRNOP00000020141<br>Mm_ENSMUSP00000056965<br>Gg_ENSGALP00000014139<br>Xt_ENSXETP00000018866<br>Dr_ENSDARP00000012648<br>Ce<br>Dm_FBpp0076513<br>Sc	NP_660155 WD repeat-containing protein 63 (Testis development protein NYD-SP29)
499	1	CI	ENSP00000304807<br>ENSG00000197532	199<br>194<br>199<br>-<br>-<br>-<br>-<br>-<br>-<br>-	llnvscedasQAEMVDFFlalmviaipl<br>llniscedssQAELVDFFlalmviavpl<br>llnvscedssQAELVDFFlalmviavpl<br>............................<br>............................<br>............................<br>............................<br>............................<br>............................<br>............................	Hs_ENSP00000304807<br>Bt_ENSBTAP00000050997<br>Rn_ENSRNOP00000037680<br>Mm<br>Gg<br>Xt<br>Dr<br>Ce<br>Dm<br>Sc	NP_001005189 Olfactory receptor 6Y1 (Olfactory receptor OR1-11)
500	1	CI	ENSP00000290567<br>ENSG00000159398	105<br>106<br>105<br>105<br>-<br>-<br>-<br>-<br>-<br>-	lqnsewllldQHMLKVHYpkfgvsedcl<br>fqnsewlftdQHILKVHYpkfrvsedcl<br>fqnlewlfiyQNLLKVHYpklgvsedcl<br>fqnlewlfiyqnllkvsypilgmsedcl<br>............................<br>............................<br>............................<br>............................<br>............................<br>............................	Hs_ENSP00000290567<br>Bt_ENSBTAP00000014830<br>Rn_ENSRNOP00000040270<br>Mm_ENSMUSP00000076988<br>Gg<br>Xt<br>Dr<br>Ce<br>Dm<br>Sc	Carboxylesterase 7 Precursor (EC 3.1.1.1)(Carboxylesterase-like urinary excreted protein homolog)(Cauxin)
501	1	CI	ENSP00000346265<br>ENSG00000129990	113<br>113<br>113<br>113<br>-<br>-<br>-<br>-<br>-<br>-	vadkhelgrlQYSLDYDFqsgqllvgil<br>vaeknelgrlQYSLDYDFqtgqllvgiv<br>vldkhqlgrlQYSLDYDFqtgqllvgil<br>vsdkhqlgrlQYSLDYDFqtgqllvgil<br>............................<br>............................<br>............................<br>............................<br>............................<br>............................	Hs_ENSP00000346265<br>Bt_ENSBTAP00000003272<br>Rn_ENSRNOP00000024547<br>Mm_ENSMUSP00000070322<br>Gg<br>Xt<br>Dr<br>Ce<br>Dm<br>Sc	NP_003171 Synaptotagmin-5 (Synaptotagmin V)(SytV)
502	1	CI	ENSP00000205061<br>ENSG00000090863	359<br>365<br>351<br>355<br>320<br>-<br>-<br>289	ekcrealttrQKLIAQDYkvsyslaksc<br>ekcrealttrQKLIAQDYkvsyslaksc<br>ekcrealttrQKLIAQDYkvsyslaksc<br>ekcrealttrQKLIAQDYkvsyslaksc<br>ekcrdalttrQKLIAQDYkvsyslaksc<br>............................<br>............................<br>pecgnllaeraylmgrdyrmahpltkac	Hs_ENSP00000205061<br>Bt_ENSBTAP00000002972<br>Rn_ENSRNOP00000025570<br>Mm_ENSMUSP00000003404<br>Gg_ENSGALP00000039178<br>Xt<br>Dr<br>Ce_CE03205	NP_036333 Golgi apparatus protein 1 Precursor (Golgi sialoglycoprotein MG-160)(E-selectin ligand 1)(ESL-1)(Cysteine-rich fibroblast growth factor receptor)(CFR-1)

				266<br>-	sqcsaritlrdqqigrdyrvshglakac<br>............................	Dm_FBpp0078043<br>Sc	
502	2	C	ENSP00000205061<br>ENSG00000090863	550<br>556<br>542<br>546<br>511<br>-<br>-<br>490<br>468<br>-	edcehrllelqyfisrdwkldpvlyrkc<br>edcehrllelqyfisrdwkldpvlyrkc<br>edcehrllelqyfisrdwkldpvlyrkc<br>edcehrllelqyfisrdwkldpvlyrkc<br>edcehrllelqyfisrdwkldvvlyrkc<br>............................<br>............................<br>pecekrllevqyfmardwtmdpqlyeac<br>pdceqalliieyfvardfkldpqlykhc<br>............................	Hs_ENSP00000205061<br>Bt_ENSBTAP00000002972<br>Rn_ENSRNOP00000025570<br>Mm_ENSMUSP00000003404<br>Gg_ENSGALP00000039178<br>Xt<br>Dr<br>Ce_CE03205<br>Dm_FBpp0078043<br>Sc	NP_036333 Golgi apparatus protein 1 Precursor (Golgi sialoglycoprotein MG-160)(E-selectin ligand 1)(ESL-1)(Cysteine-rich fibroblast growth factor receptor)(CFR-1)
503	1	CI	ENSP00000205064<br>ENSG00000103091	642<br>611<br>633<br>611<br>611<br>610<br>-<br>-<br>649<br>705	----------QVSISSFYyker------<br>----------QVSISSFYyker------<br>----------QVSISSFYykermsprsa<br>----------QVSISSFYykermsprsa<br>----------QVSISSFYyker------<br>----------QVSISSFYykertpplsa<br>............................<br>............................<br>----------reanasfylqer------<br>yvdtlglgggtngdsrtyfddetssdds	Hs_ENSP00000205064<br>Bt_ENSBTAP00000022452<br>Rn_ENSRNOP00000039886<br>Mm_ENSMUSP00000043671<br>Gg_ENSGALP00000004419<br>Xt_ENSXETP00000053713<br>Dr<br>Ce<br>Dm_FBpp0079784<br>Sc_YDR128W	NP_085058 WD repeat-containing protein 59
504	1	CI	ENSP00000367714<br>ENSG00000197921	112<br>76<br>112<br>112<br>111<br>111<br>109<br>-<br>-<br>-	ltlhaa-sdtQMKLLYHFqrppaapaap<br>ltlhaa-sdtQMKLLYHFqrppaataap<br>ltlhaa-sdtQMKLLYHFqrpp-apaap<br>ltlhaa-sdtQMKLLYHFqrpp-apaap<br>lslhsantetQMKLICHFqrsq----am<br>lcyypesgetqmkllkhlqapqklsvap<br>lslhsn-----agelqhlhsgpktnstm<br>............................<br>............................<br>............................	Hs_ENSP00000367714<br>Bt_ENSBTAP00000003221<br>Rn_ENSRNOP00000018769<br>Mm_ENSMUSP00000051118<br>Gg_ENSGALP00000001713<br>Xt_ENSXETP00000057999<br>Dr_ENSDARP00000044079<br>Ce<br>Dm<br>Sc	NP_001010926 Transcription factor HES-5 (Hairy and enhancer of split 5)
505	1	I	ENSP00000222823<br>ENSG00000106100	251<br>-<br>251<br>251<br>256<br>233<br>120<br>-<br>-<br>-	cfkesdrlclQDLLFKHYcyperdpe-e<br>............................<br>cfkesdtltlQDLLFKHFcypeqdpe-e<br>cfkesdmlslQDLLFKHFcypeqdpe-e<br>cfkedeavclkdllfryncypdqdpt-e<br>lfkkttlitledllfkyncnpdhepe-e<br>afketdeislkdlifkhncypdgdpdne<br>............................<br>............................<br>............................	Hs_ENSP00000222823<br>Bt<br>Rn_ENSRNOP00000014324<br>Mm_ENSMUSP00000055747<br>Gg_ENSGALP00000018785<br>Xt_ENSXETP00000047642<br>Dr_ENSDARP00000052748<br>Ce<br>Dm<br>Sc	NP_006083 Nucleotide-binding oligomerization domain-containing protein 1 (Caspase recruitment domain-containing protein 4)
506	1	CI	ENSP00000315569<br>ENSG00000144535	627<br>627<br>625<br>625<br>630<br>-<br>388<br>563<br>-<br>-	mlsdlvefcdQMGLPVDFssagalnksl<br>mlndlvefcdQMGLPMDFssagalnksl<br>mlsdlvefcdqmglpmdvssagalnksl<br>mlsdlvefcdqmglpmdvssagalnksl<br>llndlmefchQVGLEIDFssagslhksl<br>............................<br>mvddmqeffdQMGLDIDFsssgalhrsl<br>mikdvaeqcarigfpldgrtsgllstsl<br>............................<br>............................	Hs_ENSP00000315569<br>Bt_ENSBTAP00000037928<br>Rn_ENSRNOP00000025779<br>Mm_ENSMUSP00000070506<br>Gg_ENSGALP00000012585<br>Xt<br>Dr_ENSDARP00000077707<br>Ce_CE02758<br>Dm<br>Sc	NP_689596 DIS3-like exonuclease 2 (EC 3.1.13.-)
507	1	CI	ENSP00000332915<br>ENSG00000185480	489<br>288<br>563<br>564<br>571<br>564<br>563<br>-<br>-<br>-	ctakdklisgQAKLTQFFrl--------<br>cttkdklitgQTKLTQFFrl--------<br>cttkdklipgQTKLTRFFml--------<br>cpargklipgQTKLTQFFml--------<br>tasknklivgQAKLTQFFkltarplkdh<br>sssknkliagQAKLTSFFrv--------<br>avsk-kliagQGKLTGFFrl--------<br>............................<br>............................<br>............................	Hs_ENSP00000332915<br>Bt_ENSBTAP00000015550<br>Rn_ENSRNOP00000006249<br>Mm_ENSMUSP00000038375<br>Gg_ENSGALP00000020788<br>Xt_ENSXETP00000005393<br>Dr_ENSDARP00000041528<br>Ce<br>Dm<br>Sc	NP_060385 UPF0419 protein C12orf48
508	1	C	ENSP00000380638<br>ENSG00000120899	812<br>812<br>812<br>812<br>774<br>650<br>801<br>-	vkmrqildkqQKQMVEDYqwlrqeeksl<br>lkmrqlldkqQKQMVEDYqwlrqeekal<br>ikmrqvldrqqkqmvedsqwlrreercl<br>ikmkqvlerqqkqmvedsqwlrreercl<br>lkmrqvldkqQKQMVEDYqwlrqeeksl<br>dkmlqvlnrqqqqmeedgkwlkqeeksl<br>drlqdtlkmqkeqmeedtkwlkkeekll<br>............................	Hs_ENSP00000380638<br>Bt_ENSBTAP00000007819<br>Rn_ENSRNOP00000031615<br>Mm_ENSMUSP00000022622<br>Gg_ENSGALP00000026682<br>Xt_ENSXETP00000038080<br>Dr_ENSDARP00000057818<br>Ce	NP_004094 Protein tyrosine kinase 2 beta (EC 2.7.10.2)(Focal adhesion kinase 2)(FADK 2)(Proline-rich tyrosine kinase 2)(Cell adhesion kinase beta)(CAK beta)(Calcium-dependent tyrosine kinase)

				-<br>-	............................<br>............................	Dm<br>Sc	(CADTK)(Related adhesion focal tyrosine kinase)(RAFTK)
509	1	C	ENSP00000278379<br>ENSG00000110436	262<br>264<br>261<br>262<br>256<br>253<br>242<br>225<br>249<br>-	fgiamgkmgdQAKLMVDFfnilneivmk<br>fgiamgkmgeqaklmveffnilneivmk<br>fgiamgkmgeqaklmveffnilneivmk<br>fgiamgkmgeqaklmveffnilneivmk<br>fgiamgkmgdQAKMMVDFfnilneivmk<br>fgiamgkmgeqarlmveffnilneivmk<br>fgicmgkmgekaklmvdffntlneivmr<br>igislsqlgqeahvmvqffvimdkvimk<br>fgtflgtigqkgqvvvdffaaifevimk<br>............................	Hs_ENSP00000278379<br>Bt_ENSBTAP00000016762<br>Rn_ENSRNOP00000046952<br>Mm_ENSMUSP00000106842<br>Gg_ENSGALP00000012767<br>Xt_ENSXETP00000016454<br>Dr_ENSDARP00000068422<br>Ce_CE29083<br>Dm_FBpp0077707<br>Sc	NP_004162 Excitatory amino acid transporter 2 (Sodium-dependent glutamate/aspartate transporter 2) (Glutamate/aspartate transporter II)(Solute carrier family 1 member 2)
510	1	CI	ENSP00000301807<br>ENSG00000168016	1435<br>1543<br>406<br>366<br>1534<br>-<br>-<br>-<br>-<br>-	lsgiaeahflQGVILRDFqklrdaffkf<br>vsgiaqahflQGVIMRDFlklkdaflkf<br>lagiaeaqflQGIILRDFqklrdafikf<br>lagiaeaqfllgiilrdfqklrdafikf<br>ksgiaealflqgapkrdftklsnayfqf<br>............................<br>............................<br>............................<br>............................<br>............................	Hs_ENSP00000301807<br>Bt_ENSBTAP00000028598<br>Rn_ENSRNOP00000040406<br>Mm_ENSMUSP00000077697<br>Gg_ENSGALP00000019669<br>Xt<br>Dr<br>Ce<br>Dm<br>Sc	Unknown
511	1	CI	ENSP00000260187<br>ENSG00000036672	143<br>144<br>144<br>144<br>132<br>122<br>13<br>-<br>-<br>-	vtltqkld-sQSDLARDFsslrtsdsyr<br>vtltqkksnsQSDLARDFsslqtsdsyr<br>vtlgqkksnsQSDLARDFsslrtsdsyr<br>vtlsqkksnsQSDLARDFsslrtsdgyr<br>tllsrrravshsdlaqefsglhtsdsay<br>tsltrrkssshtdlshglselrasdtyy<br>----------------------------<br>............................<br>............................<br>............................	Hs_ENSP00000260187<br>Bt_ENSBTAP00000012857<br>Rn_ENSRNOP00000009975<br>Mm_ENSMUSP00000034508<br>Gg_ENSGALP00000010901<br>Xt_ENSXETP00000046564<br>Dr_ENSDARP00000012439<br>Ce<br>Dm<br>Sc	NP_004196 Ubiquitin carboxyl-terminal hydrolase 2 (EC 3.1.2.15)(Ubiquitin thioesterase 2)(Ubiquitin-specific-processing protease 2)(Deubiquitinating enzyme 2)(41 kDa ubiquitin-specific protease)
512	1	CI	ENSP00000343636<br>ENSG00000187871	168<br>166<br>170<br>168<br>170<br>167<br>-<br>-<br>-<br>-	ngnpcdlkqcQAAIRFFYqnipfniaqm<br>dgklcdvkhcQAAIRFFYqnmpfniaqm<br>ngnlcdvkhcQAAIRFFYqnmpfntaqm<br>ngnlcdvkhcQAAIRFFYqnmpfntaqm<br>n-kkcnmeecQAAMRFFYhnmpfevaqm<br>ngnvcnitgcqratksfyttmpfrvsel<br>............................<br>............................<br>............................<br>............................	Hs_ENSP00000343636<br>Bt_ENSBTAP00000017248<br>Rn_ENSRNOP00000043286<br>Mm_ENSMUSP00000074421<br>Gg_ENSGALP00000036182<br>Xt_ENSXETP00000003417<br>Dr<br>Ce<br>Dm<br>Sc	NP_997293 GDNF family receptor alpha-like Precursor
513	1	CI	ENSP00000347778<br>ENSG00000112053	145<br>144<br>142<br>143<br>83<br>-<br>-<br>-<br>-<br>171	viyvifgschQMSIGSFFlvsallinvl<br>viygifgschQMSIGTFFlvsalainvl<br>viyvifgschQMSIGPFFlvsalminvl<br>viyvifgschQMSIGPFFlvsalminvl<br>mlymvfgsshhisigsfsilnlvvknil<br>............................<br>............................<br>............................<br>............................<br>fvygilgsvpqmivgpesaislvvgqav	Hs_ENSP00000347778<br>Bt_ENSBTAP00000022931<br>Rn_ENSRNOP00000000615<br>Mm_ENSMUSP00000110412<br>Gg_ENSGALP00000001235<br>Xt<br>Dr<br>Ce<br>Dm<br>Sc_YPR003C	NP_443193 Testis anion transporter 1 (Anion exchange transporter)(Solute carrier family 26 member 8)
514	1	CI	ENSP00000385420<br>ENSG00000197312	298<br>298<br>-<br>298<br>296<br>247<br>308<br>-<br>303<br>266	qkiigrvhlaQVQIEGDFlpcsfsilee<br>qkiigrvhlaQVQIEGDFlacsfsilee<br>............................<br>qkiigrvhlaQVQIEGDFlacsfsilee<br>qkiigrvhlaQVQIEGDFlacsfsilee<br>qkiigrvhlaQVQIEGDFlpcsfsilee<br>qkiigrvhlaQVQIEGDFlpcsfsiled<br>............................<br>qpilgrihmvqlqiendhltssftvlgq<br>gkiigrihqaqvkietqyipcsftvldt	Hs_ENSP00000385420<br>Bt_ENSBTAP00000024915<br>Rn<br>Mm_ENSMUSP00000099542<br>Gg_ENSGALP00000028982<br>Xt_ENSXETP00000029458<br>Dr_ENSDARP00000011673<br>Ce<br>Dm_FBpp0074054<br>Sc_YER143W	NP_115717 Protein DDI1 homolog 2
515	1	CI	ENSP00000258399<br>ENSG00000135913	522<br>524<br>522<br>522<br>530<br>526<br>508<br>-	rkkplpprsiQDSLDLFFraeeleysce<br>rkkplpprsiQDSLDLFFraeeleysce<br>rkkplpprsiQDSLDLFFraeeleysce<br>rkkplpprsiQDSLDLFFraeeleysce<br>rkklfpsrsiQDSLDLFFraeeieysce<br>rkkpspsrsiQDSLDLFFrledleyace<br>rrktlpmrsiQDSLDLFFrmeeieysce<br>............................	Hs_ENSP00000258399<br>Bt_ENSBTAP00000038058<br>Rn_ENSRNOP00000033308<br>Mm_ENSMUSP00000035445<br>Gg_ENSGALP00000018614<br>Xt_ENSXETP00000032749<br>Dr_ENSDARP00000060051<br>Ce	NP_065986 Ubiquitin carboxyl-terminal hydrolase 37 (EC 3.1.2.15)(Ubiquitin thioesterase 37)(Ubiquitin-specific-processing protease 37)(Deubiquitinating enzyme 37)

				-<br>-	............................<br>............................	Dm<br>Sc	
516	1	CI	ENSP00000279024<br>ENSG00000149633	938<br>845<br>914<br>928<br>825<br>-<br>-<br>-<br>-<br>-	aafastqrafQAELTHFYmaaerqrtdl<br>aafaftqrafQAKLTHFYmaaerqrtdl<br>vafastqqafQARLTHFYmaaerqrtdl<br>aafastqqafQARLTHFYmaaerqrtdl<br>eplqaavalfrtklmsfqqrmerrqael<br>............................<br>............................<br>............................<br>............................<br>............................	Hs_ENSP00000279024<br>Bt_ENSBTAP00000025819<br>Rn_ENSRNOP00000019453<br>Mm_ENSMUSP00000040546<br>Gg_ENSGALP00000038446<br>Xt<br>Dr<br>Ce<br>Dm<br>Sc	NP_001025035 Uncharacterized protein KIAA1755
517	1	CI	ENSP00000261200<br>ENSG00000069431	1168<br>1195<br>-<br>1165<br>1176<br>859<br>1177<br>-<br>1605<br>-	kdlqelddstQLPLLCHFsetaegltti<br>kdlqelddstQLPLLCHFsetaegltti<br>............................<br>kdlqelddstQLPLLCHFsetaegltti<br>kdlqelddstllpllchfsetaegltti<br>----------------------------<br>kdlqelddstQLPLLCHFsetaegltti<br>............................<br>relqrienatnspvishlsetiqgvtti<br>............................	Hs_ENSP00000261200<br>Bt_ENSBTAP00000040663<br>Rn<br>Mm_ENSMUSP00000098390<br>Gg_ENSGALP00000021593<br>Xt_ENSXETP00000001948<br>Dr_ENSDARP00000014386<br>Ce<br>Dm_FBpp0288707<br>Sc	NP_064693 ATP-binding cassette transporter sub-family C member 9 (Sulfonylurea receptor 2)
518	1	CI	ENSP00000379122<br>ENSG00000108557	43<br>43<br>43<br>43<br>-<br>43<br>-<br>-<br>-<br>-	psqaglscdrQRLLAKDYynpqpypsye<br>psqaglscdrQRLLTKDYygpqpfpgye<br>pgqaglscdrQRLLAKDYynpqpytgye<br>pgqaglscdrQRLLAKDYyspqpytgye<br>............................<br>qsqpalncerhrqvtksyyahtsyqgye<br>............................<br>............................<br>............................<br>............................	Hs_ENSP00000379122<br>Bt_ENSBTAP00000006264<br>Rn_ENSRNOP00000030733<br>Mm_ENSMUSP00000070896<br>Gg<br>Xt_ENSXETP00000026563<br>Dr<br>Ce<br>Dm<br>Sc	Retinoic acid-induced protein 1
519	1	CI	ENSP00000011619<br>ENSG00000010017	182<br>0<br>91<br>107<br>112<br>37<br>64<br>-<br>435<br>-	kdkfsyiglsQNNLRVHYkghgktpkda<br>-----------vflpvpdaghgktpkda<br>kdkfsyiglsQNNLRVHYkghgktpkda<br>kdkfsyiglsQNNLRVHYkghgktpkda<br>kdkfsyiglsQNNLRVHYkghgktpkda<br>rdkcsylglshgnlrvhykghgktskda<br>kdkfsyiglsQNNLRVHYkghgktpkda<br>............................<br>hdkclsiglsqnnlrvtykgvgkqhsda<br>............................	Hs_ENSP00000011619<br>Bt_ENSBTAP00000028721<br>Rn_ENSRNOP00000024174<br>Mm_ENSMUSP00000045791<br>Gg_ENSGALP00000020696<br>Xt_ENSXETP00000004846<br>Dr_ENSDARP00000081043<br>Ce<br>Dm_FBpp0099887<br>Sc	NP_005484 Ran-binding protein 9 (RanBP9)(RanBP7)(Ran-binding protein M)(RanBPM)(BPM90)(BPM-L)
520	1	CI	ENSP00000261244<br>ENSG00000100578	250<br>265<br>249<br>-<br>305<br>-<br>-<br>-<br>-<br>-	irhleklqqqQIDIQTHFisaalktssf<br>irhleklqqqQIDIQAHFisaalktssf<br>irhleklqqqmdiqvsvitaalkassl<br>............................<br>ihhleklqehQMNIQSHFissavnmggl<br>............................<br>............................<br>............................<br>............................<br>............................	Hs_ENSP00000261244<br>Bt_ENSBTAP00000008528<br>Rn_ENSRNOP00000011056<br>Mm<br>Gg_ENSGALP00000036550<br>Xt<br>Dr<br>Ce<br>Dm<br>Sc	Uncharacterized protein KIAA0586
520	2	C	ENSP00000261244<br>ENSG00000100578	587<br>600<br>576<br>-<br>626<br>-<br>-<br>-<br>-<br>-	nksviprkhsQKQIEEHFrnlpmrgmpa<br>nnsvilrkhsqkqkedhlrnppirsmpa<br>nrsiiprshyqkqtqeqftsppvrsvpa<br>............................<br>kkplsaakslrnhvedniskegfrtyfs<br>............................<br>............................<br>............................<br>............................<br>............................	Hs_ENSP00000261244<br>Bt_ENSBTAP00000008528<br>Rn_ENSRNOP00000011056<br>Mm<br>Gg_ENSGALP00000036550<br>Xt<br>Dr<br>Ce<br>Dm<br>Sc	Uncharacterized protein KIAA0586
521	1	CI	ENSP00000269856<br>ENSG00000141965	343<br>327<br>-<br>328<br>356<br>-<br>-<br>-	geylpkpeppQLVLAYDYsrevntteel<br>geylpkpeppQLVLAYDYsrevntteel<br>............................<br>ggylpkpepqQLVLAYDYsrevttpqel<br>gqylpkpeprQLVLAYDYsrevssleel<br>............................<br>............................<br>............................	Hs_ENSP00000269856<br>Bt_ENSBTAP00000004519<br>Rn<br>Mm_ENSMUSP00000057996<br>Gg_ENSGALP00000039928<br>Xt<br>Dr<br>Ce	NP_061178 Protein fem-1 homolog A (FEM1-alpha)(FEM1a)(Prostaglandin E receptor 4-associated protein)

				-	....	Dm	
				-	....	Sc	
