# Supplementary material for: The role of PCNA as a scaffold protein in cellular signaling is functionally conserved between yeast and humans
Source: FEBS Open Bio. 2018 May 31;8(7):1135–45. doi: 10.1002/2211-5463.12442 (PMC6026702; doi:10.1002/2211-5463.12442)
Supplement: Supplementary file 3 — Table S1. (B) Output from confind, APIM‐hu. [file FEB4-8-1135-s003.pdf]

The role of PCNA as a scaffold protein in cellular signaling is functionally conserved between yeast and humans

Camilla Olaisen[1], Hans Fredrik N. Kvitvang[2], Sungmin Lee[2], Eivind Almaas[2], Per Bruheim[2], Finn Drabløs[1], and Marit Otterlei[1*].

[1]Department of Clinical and Molecular Medicine, Faculty of Medicine and Health Sciences, Norwegian University of Science and Technology (NTNU), Trondheim, Norway.

[2]Department of Biotechnology and Food Science, Faculty of Natural Sciences, Norwegian University of Science and Technology (NTNU), Trondheim, Norway.

Supplementary Table S1 B: Output from confind, APIM-hu

Shown below as pdf. Also found on web at

<http://tare.medisin.ntnu.no/pcna/index.php>

confind

```
confind (c) Finn Drablos, NTNU 2007,2012
Version 02.10.2012
Program started Mon Jul 06 13:19:04 CEST 2015
----------
Configuration file for Alkbh2 motif
Version FD 23-01-2012

Datasets are from Inparanoid 7.0. Gene descriptions are
mainly taken from Inparanoid 7.0. However, in case annotations
are not found in Inparanoid 7.0 they can be retrieved from
Ensembl 65 data. Localisation data are from eSLDB.

Genes that are mentioned in the paper are highlighted
in green, in the order they are listed in the paper.

Using K as equivalence for KR in consensus
Using F as equivalence for FYW in consensus
Using L as equivalence for LVI in consensus
----------
Found 21673 sequences in ./lib/H.sapiens.fa
Got match in 657 sequences against pattern [KR][FYW](([LVI][ALVI])|([ALVI][LVI]))[KR]
Removed 0 entries with no info in ./ref/eSLDB_Homo_sapiens_TS.txt matching .*
There are 657 entries left
Found 532 orthologs in Bt (Bos taurus)
Found 528 orthologs in Rn (Rattus norvegicus)
Found 577 orthologs in Mm (Mus musculus)
Found 447 orthologs in Gg (Gallus gallus)
Found 370 orthologs in Xt (Xenopus tropicalis)
Found 354 orthologs in Dr (Danio rerio)
Found 186 orthologs in Ce (Caenorhabditis elegans)
Found 232 orthologs in Dm (Drosophila melanogaster)
Found 96 orthologs in Sc (Saccharomyces cerevisiae)
Removed 48 entries without orthologs
There are 609 entries left
Created 164 new sequence files
Added 868 new orthologs to these sequence files
Ran ClustalW on 164 library files
Removed 106 sequences where pattern was not conserved
There are 503 entries left
Missing information on gene for 0 proteins
Removed 0 duplicate sequences representing the same gene
There are 503 entries left
Description is missing for 0 genes
----------
Pro - Protein number (green if on priority list)
# - Motif number i protein
CI - Entry is accepted according to Consensus or Individual pattern hits
Protein ID / Gene ID - IDs from input data
Pos - Position of motif in sequence
Alignment - Alignment of motif (UPPER case) and flanking region (lower case)
   lower case only - no hit by motif, "-----" - gaps, "....." - missing protein
Ortho ID - ID of Orthologs, with organism code (see above)
Description
```

Pro	#	CI	Protein ID<br>Gene ID	Pos	Alignment	Ortho ID	Description
1	1	CI	ENSP00000343021<br>ENSG00000189046	3<br>3<br>3<br>3<br>3<br>0<br>3<br>-<br>-<br>-	--------mdRFLVKgaqggllrkq<br>--------mdRFLVKgavgslkrrm<br>--------mdRFLVRpdrgdiqgaa<br>--------mdKFLVRpdlrdlqggg<br>--------mdRFVVKrsaeepggdg<br>-------------------------<br>--------mdkfltrkrkrsseepd<br>.........................<br>.........................<br>.........................	Hs_ENSP00000343021<br>Bt_ENSBTAP00000026442<br>Rn_ENSRNOP00000030705<br>Mm_ENSMUSP00000056043<br>Gg_ENSGALP00000007984<br>Xt_ENSXETP00000048249<br>Dr_ENSDARP00000078120<br>Ce<br>Dm<br>Sc	NP_001001655 Alpha-ketoglutarate-dependent dioxygenase alkB homolog 2 (EC 1.14.11.-)(Alkylated DNA repair protein alkB homolog 2)(Oxy DC1)
2	1	CI	ENSP00000360382<br>ENSG00000116205	33<br>3	qgsrspltmdKFVIRtpriqnspqk<br>--------mdKFVIRtpriqsspqk	Hs_ENSP00000360382<br>Bt_ENSBTAP00000012535	NP_694580 Uncharacterized protein C1orf83

				3<br>3<br>3<br>3<br>3<br>-<br>-<br>-	--------mdKFVIRtpriqnspkk<br>--------mdKFVIRtpriqnspkk<br>--------meRFVVRrarspqsprr<br>--------mdRFVIRkpkseaaeek<br>--------mdKFVVRlpk-ddmmkq<br>.........................<br>.........................<br>.........................	Rn_ENSRNOP00000049524<br>Mm_ENSMUSP00000054142<br>Gg_ENSGALP00000017466<br>Xt_ENSXETP00000043216<br>Dr_ENSDARP00000085930<br>Ce<br>Dm<br>Sc	
3	1	CI	ENSP00000322542<br>ENSG00000077809	430<br>430<br>411<br>430<br>406<br>374<br>-<br>-<br>-<br>-	erillakeriRFVIKkhellnstre<br>erillakeriRFVIKkhellnstre<br>erillakeriRFVIKkhellnstre<br>erillakeriRFVIKkhellnstre<br>erilvakeriRFVIKkhellnstqe<br>erillareriRFVIKkhdlln-ard<br>.........................<br>.........................<br>.........................<br>.........................	Hs_ENSP00000322542<br>Bt_ENSBTAP00000012902<br>Rn_ENSRNOP00000002020<br>Mm_ENSMUSP00000049625<br>Gg_ENSGALP00000001923<br>Xt_ENSXETP00000039944<br>Dr<br>Ce<br>Dm<br>Sc	NP_127492 General transcription factor II-I (GTFII-I)(TFII-I)(Bruton tyrosine kinase-associated protein 135)(BTK-associated protein 135)(BAP-135)(SRF-Phox1-interacting protein)(SPIN)(Williams-Beuren syndrome chromosomal region 6 protein)
3	2	CI	ENSP00000322542<br>ENSG00000077809	535<br>535<br>516<br>535<br>511<br>478<br>-<br>-<br>-<br>-	ekiiqvgnriKFVIKrpellthstt<br>ekiiqvgnriKFVIKrpellthstt<br>ekiiqvgnriKFVIKrpellthstt<br>ekiiqvgnriKFVIKrpellthstt<br>ekiiqvgnriKFVIKrpelltlssp<br>ekiiqvgnriKFVIKkpelltqsva<br>.........................<br>.........................<br>.........................<br>.........................	Hs_ENSP00000322542<br>Bt_ENSBTAP00000012902<br>Rn_ENSRNOP00000002020<br>Mm_ENSMUSP00000049625<br>Gg_ENSGALP00000001923<br>Xt_ENSXETP00000039944<br>Dr<br>Ce<br>Dm<br>Sc	NP_127492 General transcription factor II-I (GTFII-I)(TFII-I)(Bruton tyrosine kinase-associated protein 135)(BTK-associated protein 135)(BAP-135)(SRF-Phox1-interacting protein)(SPIN)(Williams-Beuren syndrome chromosomal region 6 protein)
3	3	CI	ENSP00000322542<br>ENSG00000077809	640<br>640<br>621<br>640<br>616<br>583<br>-<br>-<br>-<br>-	erivrgsnkiKFVVKkpelvisylp<br>erivrgsnkiKFVVKkpelvisylp<br>erivrgsnkiKFVVKkpelvvsylp<br>erivrgsnkiKFVVKkpelvvsylp<br>erivrgsnkiRFVVKkpelvasylp<br>erivrgsakiKFIVKkpqlvathip<br>.........................<br>.........................<br>.........................<br>.........................	Hs_ENSP00000322542<br>Bt_ENSBTAP00000012902<br>Rn_ENSRNOP00000002020<br>Mm_ENSMUSP00000049625<br>Gg_ENSGALP00000001923<br>Xt_ENSXETP00000039944<br>Dr<br>Ce<br>Dm<br>Sc	NP_127492 General transcription factor II-I (GTFII-I)(TFII-I)(Bruton tyrosine kinase-associated protein 135)(BTK-associated protein 135)(BAP-135)(SRF-Phox1-interacting protein)(SPIN)(Williams-Beuren syndrome chromosomal region 6 protein)
3	4	CI	ENSP00000322542<br>ENSG00000077809	802<br>802<br>783<br>802<br>779<br>738<br>-<br>-<br>-<br>-	ekilrnkakiKFIIKkpemfetaik<br>ekilrnkakiKFIIKkpemfetaik<br>ekilrnkakiKFIIKkpemfetaik<br>ekilrnkakiKFIIKkpemfetaik<br>ekilrnkskiKFIIKkpemfeaavk<br>ekilrnrakiKFVIKkpemientak<br>.........................<br>.........................<br>.........................<br>.........................	Hs_ENSP00000322542<br>Bt_ENSBTAP00000012902<br>Rn_ENSRNOP00000002020<br>Mm_ENSMUSP00000049625<br>Gg_ENSGALP00000001923<br>Xt_ENSXETP00000039944<br>Dr<br>Ce<br>Dm<br>Sc	NP_127492 General transcription factor II-I (GTFII-I)(TFII-I)(Bruton tyrosine kinase-associated protein 135)(BTK-associated protein 135)(BAP-135)(SRF-Phox1-interacting protein)(SPIN)(Williams-Beuren syndrome chromosomal region 6 protein)
4	1	CI	ENSP00000269577<br>ENSG00000131747	1047<br>-<br>956<br>966<br>989<br>-<br>-<br>-<br>-<br>-	yreyhtdttvKFVVKmteeklaeae<br>.........................<br>yreyhtdttvKFVIKmteeklaeae<br>yreyhtdttvKFVIKmteeklaeae<br>ykeyhtdttvKFVVKmseeklaeae<br>.........................<br>.........................<br>.........................<br>.........................<br>.........................	Hs_ENSP00000269577<br>Bt<br>Rn_ENSRNOP00000040257<br>Mm_ENSMUSP00000068896<br>Gg_ENSGALP00000006239<br>Xt<br>Dr<br>Ce<br>Dm<br>Sc	TOP2A_HUMAN Isoform 2 of P11388 - Homo sapiens (Human)
5	1	CI	ENSP00000364270<br>ENSG00000136936	163<br>163<br>163<br>162<br>-<br>154<br>-<br>129<br>184<br>250	cdlekrepplKFIVKknphhsqwgd<br>cdlekrepalKFIVKknphhsqwgd<br>cdlekrepalRFIVKknprhsqwgd<br>cdlekrepalRFLVKknprhsqwgd<br>.........................<br>ikeekrnyhfkkkslnkvnisflsc<br>.........................<br>cdldlrkpklrywakknphnprygd<br>cdfdkrepklryisrknphnvrwge<br>pelnded-lfhrlekpnphsgtfar	Hs_ENSP00000364270<br>Bt_ENSBTAP00000012834<br>Rn_ENSRNOP00000012779<br>Mm_ENSMUSP00000050453<br>Gg<br>Xt_ENSXETP00000022457<br>Dr<br>Ce_CE06130<br>Dm_FBpp0070638<br>Sc_YMR201C	NP_000371 DNA-repair protein complementing XP-A cells (Xeroderma pigmentosum group A-complementing protein)
6	1	C	ENSP00000351137<br>ENSG00000076924	563<br>563	vsdiwstyltKFIAR-yggrklera<br>vsdiwstyltKFIAR-yggrklera	Hs_ENSP00000351137<br>Bt_ENSBTAP00000015097	NP_064581 Pre-mRNA-splicing factor SYF1 (XPA-binding protein

				563	vsdiwstyltkfisr-yggrklera	Rn_ENSRNOP00000001309	2)(Protein HCNP)
				563	vsdiwstyltkfisr-yggrklera	Mm_ENSMUSP00000019614	
				-	....	Gg	
				429	vydiwstylsKFIAR-yggkklera	Xt_ENSXETP00000005592	
				558	vhdiwntyltkfidr-yggkklera	Dr_ENSDARP00000010070	
				567	vfdiwntylvkfikr-yggkklera	Ce_CE08909	
				562	vydiwnsyltkfler-yggtklera	Dm_FBpp0086749	
				630	qyelwieylevatshqlsslspehi	Sc_YDR416W	
7	1	CI	ENSP00000355759 ENSG00000143799	1000	ivydiaqvnlKYLLKlkfnfkts--	Hs_ENSP00000355759	NP_001609 Poly
				1005	ivydiaqvhlKYLLKlkfnfkts--	Bt_ENSBTAP00000001113	
				1000	ivydiaqvnlKYLLKlkfnfkts--	Rn_ENSRNOP00000004232	
				1000	ivydiaqvnlKYLLKlkfnfkts--	Mm_ENSMUSP00000027777	
				1001	ivydvaqvnlKYLLKlkfnykts--	Gg_ENSGALP00000038037	
				981	ivydvaqvnlKYLLKlkfnykdkgg	Xt_ENSXETP00000048934	
				999	ivydvsqvnlKYLLKirfnyqts--	Dr_ENSDARP00000008364	
				932	ivydvdqiqlKYLVRvkmhharhl-	Ce_CE25556	
				983	ivydvaqvniqylfrmefkysy---	Dm_FBpp0112608	
				-	....	Sc	
8	1	CI	ENSP00000250416 ENSG00000129484	570	ivynpnqvrmRYLLKvqfnflqlw-	Hs_ENSP00000250416	NP_005475 Poly
				551	ivynpnqvrmRYLLKvqfnflqlw-	Bt_ENSBTAP00000012338	
				545	ivyspsqvhmRYLLKiqfnflqlw-	Rn_ENSRNOP00000011840	
				546	ivyspnqvrmRYLLKiqfnflqlw-	Mm_ENSMUSP00000048877	
				-	....	Gg	
				545	ivydphqvrmkyllqvhfnysslw-	Xt_ENSXETP00000003642	
				-	....	Dr	
				-	....	Ce	
				-	....	Dm	
				-	....	Sc	
9	1	CI	ENSP00000371419 ENSG00000102699	559	vvyktnqvkmKYIIKfsmpgdqikd	Hs_ENSP00000371419	NP_006428 Poly
				560	vvyktnqvkmKYLVKfsvpgdeikd	Bt_ENSBTAP00000035522	
				-	....	Rn	
				567	vvyktnqvkmKYIVKfctpgdqike	Mm_ENSMUSP00000066130	
				568	vvykttqvkiRYVVKfclaedqikq	Gg_ENSGALP00000027638	
				556	vvykkdqikmryavqfctdkdqvnm	Xt_ENSXETP00000043695	
				-	....	Dr	
				-	....	Ce	
				-	....	Dm	
				-	....	Sc	
10	1	CI	ENSP00000264331 ENSG00000077097	988	ykeyhtdttvKFVVKmt--eeklaq	Hs_ENSP00000264331	NP_001059 DNA topoisomerase 2-beta (EC 5.99.1.3)(DNA topoisomerase II, beta isozyme)
				960	ykeyhtdttvKFVVKmt--eeklaq	Bt_ENSBTAP00000006030	
				-	....	Rn	
				976	ykeyhtdttvKFVVKmt--eeklaq	Mm_ENSMUSP00000017629	
				995	ykeyhtdttvKFVVKmt--eeklaq	Gg_ENSGALP00000018410	
				965	ykeyhtdttvKFVVKmt--eeklaq	Xt_ENSXETP00000015835	
				982	ykeyhtdttvKFVVRmt--eeklaq	Dr_ENSDARP00000030178	
				1002	ykeyhtdttvKFVVKls--pgklre	Ce_CE06184	
				947	yreyhtdttvrfvisfapgeferih	Dm_FBpp0080825	
				942	meeqhddn-ikfiitls--peemak	Sc_YNL088W	
11	1	C	ENSP00000385485 ENSG00000182185	233	qlqgnlkernKFLAReasslkylae	Hs_ENSP00000385485	NP_598193 DNA repair protein RAD51 homolog 2 (R51H2) (RAD51-like protein 1)(Rad51B)
				233	qlqgnvrernKFLAReaaslkylae	Bt_ENSBTAP00000025252	
				233	qlqgnikernkflgkgasllkylsg	Rn_ENSRNOP00000016459	
				233	klqgnikernkflgkgasllkylag	Mm_ENSMUSP00000078490	
				233	klqgnlaersnflargasllkyvae	Gg_ENSGALP00000015437	
				-	....	Xt	
				229	slpgnlthrsnflgqeaavlkylsq	Dr_ENSDARP00000053846	
				-	....	Ce	
				-	....	Dm	
				-	....	Sc	
12	1	I	ENSP00000357795 ENSG00000009413	1240	kiksqsgaevKFVLKhqnvsefass	Hs_ENSP00000357795	NP_002903 DNA polymerase zeta catalytic subunit (hREV3)(EC 2.7.7.7)
				-	....	Bt	
				1240	ttkpps--eaeltpsqrsvpeltss	Rn_ENSRNOP00000000725	
				1240	kmkphs--eaeltpnhqsvseltss	Mm_ENSMUSP00000019986	
				1200	kgnnhsatevKFVLKhqdlpemsyn	Gg_ENSGALP00000024199	
				-	....	Xt	
				-	....	Dr	
				179	-------------------------	Ce_CE39187	
				779	-------------------------	Dm_FBpp0087907	
				330	-------------------------	Sc_YPL167C	
13	1	CI	ENSP00000263274 ENSG00000105486	440	lfvacrhseaRFIARslsgrlrlgl	Hs_ENSP00000263274	NP_000225 DNA ligase 1 (EC 6.5.1.1)(DNA ligase I)
				440	lfvacrhseaRFIARalsgrlrlgl	Bt_ENSBTAP00000019427	

				434<br>454<br>184<br>589<br>585<br>171<br>274<br>300	lfvacryseaRFIARslsgrlrlgl<br>lfvacrhseaRYIARslsgrlrlgl<br>lfvacrhseaRYLVRslsgklrigl<br>lfvacrhseaRYIARslggklrigl<br>lfvacrfseaRYIVRslagklrigl<br>lliacqgieaRFLVRmlagkmrigl<br>mfvacrssearffirsligklrigi<br>mltackgieaKFLIRsleskl rigl	Rn_ENSRNOP00000019799<br>Mm_ENSMUSP00000096411<br>Gg_ENSGALP00000039729<br>Xt_ENSXETP00000019357<br>Dr_ENSDARP00000078609<br>Ce_CE37480<br>Dm_FBpp0072041<br>Sc_YDL164C	(Polydeoxyribonucleotide synthase
14	1	CI	ENSP00000349393<br>ENSG00000174405	184<br>189<br>184<br>184<br>189<br>189<br>184<br>158<br>178<br>190	litqssaleqKWLIRmiikdlk--l<br>litqssaleqKWLIRmivkdlk--l<br>litqcsaleqKWLIRmiikdlk--l<br>litqssaleqKWLIRmiikdlk--l<br>litqstaleqKWLIRmiikdlk--l<br>lianttaleqKWLIRmiikdmk--l<br>litqssaleqKWLIRmilkdmk--l<br>lvkrcsedelewifnvilknvesal<br>fteqaspeeqKWLIRlllkslg--l<br>cvenmsfvelkyffdivlknrv--i	Hs_ENSP00000349393<br>Bt_ENSBTAP00000021092<br>Rn_ENSRNOP00000019615<br>Mm_ENSMUSP00000093130<br>Gg_ENSGALP00000027179<br>Xt_ENSXETP00000005699<br>Dr_ENSDARP00000080083<br>Ce_CE34522<br>Dm_FBpp0073678<br>Sc_YOR005C	NP_001091738 DNA ligase 4 (EC 6.5.1.1)(DNA ligase IV)(Polydeoxyribonucleotide synthase
15	1	C	ENSP00000364454<br>ENSG00000158169	334<br>220<br>335<br>335<br>337<br>331<br>332<br>-<br>-<br>-	ealekaskqlRFALKtyfpytspsl<br>ealekenkqlKFALKtyfpyaspal<br>ealrkenkqlKFALKtyfpysapcl<br>ealkkenkqltfalrtyfpygapcl<br>qvqqnenkqhkfplkayfphhhqpl<br>qlyqknntqvrfplkayfphsnrsl<br>qrltgqnsqdrlplraffpnvmpsl<br>.........................<br>.........................<br>.........................	Hs_ENSP00000364454<br>Bt_ENSBTAP00000022853<br>Rn_ENSRNOP00000022884<br>Mm_ENSMUSP00000021916<br>Gg_ENSGALP00000020570<br>Xt_ENSXETP00000057461<br>Dr_ENSDARP00000089800<br>Ce<br>Dm<br>Sc	NP_000127 Fanconi anemia group C protein (Protein FACC)
16	1	CI	ENSP00000381295<br>ENSG00000034063	590<br>585<br>-<br>581<br>533<br>-<br>575<br>-<br>-<br>-	ekgksgflvwRYLLRrdddepgpwt<br>ekgksgflvwRFLLRrddvepgpwt<br>.........................<br>ergksgflvwRYLLRrddtepepwt<br>etgksgflvwRYLLRrddeepapwt<br>.........................<br>ekgksgflvwRYLLKrndeesapwt<br>.........................<br>.........................<br>.........................	Hs_ENSP00000381295<br>Bt_ENSBTAP00000002877<br>Rn<br>Mm_ENSMUSP00000001258<br>Gg_ENSGALP00000006565<br>Xt<br>Dr_ENSDARP00000018915<br>Ce<br>Dm<br>Sc	NP_001041666 E3 ubiquitin-protein ligase UHRF1 (EC 6.3.2.-)(Ubiquitin-like PHD and RING finger domain-containing protein 1)(Ubiquitin-like-containing PHD and RING finger domains protein 1)(Inverted CCAAT box-binding protein of 90 kDa)(Transcription factor ICBP90)(Nuclear zinc finger protein Np95)(Nuclear protein 95)(HuNp95)(RING finger protein 106)
17	1	CI	ENSP00000276893<br>ENSG00000147854	607<br>608<br>608<br>608<br>564<br>604<br>-<br>-<br>-<br>-	issshgflvwRYLLRrddvepapwt<br>issshgflvwRYLLRrddvepapwt<br>issshgflvwRYLLRrddvepapwt<br>issshgflvwRYLLRrddvepapwt<br>ig-kcgflvwRYLLRrddvepapwt<br>ig-kcgflvwRYLLRrddaepapwt<br>.........................<br>.........................<br>.........................<br>.........................	Hs_ENSP00000276893<br>Bt_ENSBTAP00000027737<br>Rn_ENSRNOP00000015406<br>Mm_ENSMUSP00000025739<br>Gg_ENSGALP00000024227<br>Xt_ENSXETP00000001255<br>Dr<br>Ce<br>Dm<br>Sc	NP_690856 E3 ubiquitin-protein ligase UHRF2 (EC 6.3.2.-)(Ubiquitin-like PHD and RING finger domain-containing protein 2)(Ubiquitin-like-containing PHD and RING finger domains protein 2)(Np95/ICBP90-like RING finger protein)(Np95-like RING finger protein)(Nuclear zinc finger protein Np97)(RING finger protein 107)
18	1	CI	ENSP00000290650<br>ENSG00000159459	312<br>301<br>312<br>312<br>287<br>288<br>-<br>-<br>-<br>-	vlhseimahqKFALRlgswmnkims<br>vlhsevmahqKFALRlgswmnkims<br>vlhsvvmahqKFALRlgswmnkims<br>vlhsvvmahqKFALRlgswmnkims<br>vlhadvmahqKFALRlgswlnklms<br>vlhghvmahqsfalclaiwlnklla<br>.........................<br>.........................<br>.........................<br>.........................	Hs_ENSP00000290650<br>Bt_ENSBTAP00000028086<br>Rn_ENSRNOP00000015086<br>Mm_ENSMUSP00000028728<br>Gg_ENSGALP00000015034<br>Xt_ENSXETP00000002119<br>Dr<br>Ce<br>Dm<br>Sc	NP_777576 E3 ubiquitin-protein ligase UBR1 (EC 6.3.2.-)(N-recognin-1)(Ubiquitin-protein ligase E3-alpha-1)(Ubiquitin-protein ligase E3-alpha-I)
19	1	CI	ENSP00000361992<br>ENSG00000024048	990<br>994<br>990<br>990<br>989	pylevhkdmiRWILKtfnavkkmre<br>pylevhkdmiKWILKtfnaikklre<br>pslevhkdmiRWLLKmfntikkire<br>psleahkdmiRWLLKmfnaikkire<br>phlevhkdmiRWILKtfntikklre	Hs_ENSP00000361992<br>Bt_ENSBTAP00000007833<br>Rn_ENSRNOP00000021158<br>Mm_ENSMUSP00000075230<br>Gg_ENSGALP00000016092	E3 ubiquitin-protein ligase UBR2 (EC 6.3.2.-)(N-recognin-2)(Ubiquitin-protein ligase E3-alpha-

				992<br>-<br>1091<br>994<br>1128	ahlevhkdmiRWILKmvsnikkire<br>.........................<br>pessicpillevtvekyrklikara<br>prleahydfvlwtierfkqlqakqa<br>adyllekmimkkpnelfesliasfg	Xt_ENSXETP00000000438<br>Dr<br>Ce_CE40441<br>Dm_FBpp0074189<br>Sc_YGR184C	2)(Ubiquitin-protein ligase E3-alpha-II)
20	1	CI	ENSP00000313299<br>ENSG00000181827	149<br>150<br>150<br>246<br>-<br>154<br>-<br>-<br>152<br>-	qpfdtvlelaRFLVKshyigtksma<br>qpfdtvlelaRFLVKshyigtksma<br>qpfdtvlelahflvkshyigtksma<br>qpfdtvlelahflvkshyigtksma<br>.........................<br>qpfdsvldlaRFLVKshyigtksma<br>.........................<br>.........................<br>sanaaglafanafsfstaqqlglgk<br>.........................	Hs_ENSP00000313299<br>Bt_ENSBTAP00000000738<br>Rn_ENSRNOP00000008644<br>Mm_ENSMUSP00000091338<br>Gg<br>Xt_ENSXETP00000036081<br>Dr<br>Ce<br>Dm_FBpp0081227<br>Sc	NP_073752 Regulatory factor X domain-containing protein 2
21	1	CI	ENSP00000359924<br>ENSG00000134602	352<br>343<br>258<br>272<br>272<br>272<br>268<br>-<br>-<br>-	rptakellkhKFIVKnskktsylte<br>rptakellkhKFIVKnskktsylte<br>rptakellkhKFIVKnskktsylte<br>rptakellkhKFIVKnskktsylte<br>rptakellkhkfimknakktsylte<br>rptarellkhKYIVKnakrtsylte<br>rptakellkhKFIVKyakktsylte<br>.........................<br>.........................<br>.........................	Hs_ENSP00000359924<br>Bt_ENSBTAP00000026189<br>Rn_ENSRNOP00000010596<br>Mm_ENSMUSP00000033444<br>Gg_ENSGALP00000009728<br>Xt_ENSXETP00000011925<br>Dr_ENSDARP00000079474<br>Ce<br>Dm<br>Sc	NP_057626 Serine/threonine-protein kinase MST4 (EC 2.7.11.1)(Mammalian STE20-like protein kinase 4)(STE20-like kinase MST4)(MST-4)(Serine/threonine-protein kinase MASK)(Mst3 and SOK1-related kinase)
22	1	CI	ENSP00000297512<br>ENSG00000213199	322<br>322<br>324<br>321<br>-<br>-<br>-<br>-<br>446<br>-	lmgcrlacetRYVARkcgcrmvymp<br>lmgcrlacqsRYVARkcgcrmmhmp<br>ligcrlacesRYVARkcgcrmmhmp<br>ligcrlacesRYVARkcgcrmmhmp<br>.........................<br>.........................<br>.........................<br>.........................<br>qsnceleclanftltkcgcvkfsmp<br>.........................	Hs_ENSP00000297512<br>Bt_ENSBTAP00000010209<br>Rn_ENSRNOP00000011300<br>Mm_ENSMUSP00000039914<br>Gg<br>Xt<br>Dr<br>Ce<br>Dm_FBpp0271902<br>Sc	NP_064717 Amiloride-sensitive cation channel 3 (Neuronal amiloride-sensitive cation channel 3)(Acid-sensing ion channel 3)(ASIC3)(hASIC3)(Testis sodium channel 1)(hTNaC1)
22	2	CI	ENSP00000297512<br>ENSG00000213199	391<br>391<br>393<br>390<br>-<br>-<br>-<br>-<br>532<br>-	mvripsraaaRFLARklnrsea-yi<br>mvrmpsrpaaRYLARkhnrsea-yi<br>mvripsrasaRYLARkynrses-yi<br>mvripsrasaRYLARkynrset-yi<br>.........................<br>.........................<br>.........................<br>.........................<br>qadfdyktvintdspegkeeqskrq<br>.........................	Hs_ENSP00000297512<br>Bt_ENSBTAP00000010209<br>Rn_ENSRNOP00000011300<br>Mm_ENSMUSP00000039914<br>Gg<br>Xt<br>Dr<br>Ce<br>Dm_FBpp0271902<br>Sc	NP_064717 Amiloride-sensitive cation channel 3 (Neuronal amiloride-sensitive cation channel 3)(Acid-sensing ion channel 3)(ASIC3)(hASIC3)(Testis sodium channel 1)(hTNaC1)
23	1	I	ENSP00000376943<br>ENSG00000153094	188<br>129<br>186<br>186<br>-<br>-<br>-<br>-<br>-<br>-	hprmvilrllRYIVRlvwrmh----<br>hpqmvllrvlRYIVRlvwrmq----<br>hpqmvilqllrfifrlvwrrh----<br>hpqmvilqllrfifrlvwrrh----<br>.........................<br>.........................<br>.........................<br>.........................<br>.........................<br>.........................	Hs_ENSP00000376943<br>Bt_ENSBTAP00000046015<br>Rn_ENSRNOP00000022596<br>Mm_ENSMUSP00000105970<br>Gg<br>Xt<br>Dr<br>Ce<br>Dm<br>Sc	NP_619527 Bcl-2-like protein 11 (Bcl2-interacting mediator of cell death)
24	1	CI	ENSP00000262795<br>ENSG00000099882	733<br>1124<br>-<br>-<br>-<br>-<br>1189<br>-<br>-<br>-	lsiphepkevRFVVRsvsarsrsps<br>lsiphepkevRFVVRsvsarsrsps<br>.........................<br>.........................<br>.........................<br>.........................<br>lslphepkevrfvmrsssarsrsps<br>.........................<br>.........................<br>.........................	Hs_ENSP00000262795<br>Bt_ENSBTAP00000040212<br>Rn<br>Mm<br>Gg<br>Xt<br>Dr_ENSDARP00000086888<br>Ce<br>Dm<br>Sc	NP_001073889 SH3 and multiple ankyrin repeat domains protein 3 (Shank3)(Proline-rich synapse-associated protein 2)(ProSAP2)
25	1	CI	ENSP00000345834<br>ENSG00000130396	125<br>97<br>0<br>-<br>129	lnwnkddregRFVLKnendaippkk<br>lnwnkddregRFVLKnendavpakk<br>-------------------------<br>.........................<br>lnwnkddregRFVLKnendtlppkk	Hs_ENSP00000345834<br>Bt_ENSBTAP00000007309<br>Rn_ENSRNOP00000048254<br>Mm<br>Gg_ENSGALP00000018461	NP_001035090 Afadin (Protein AF-6)

				-<br>91<br>120<br>125<br>-	.........................<br>lnwnkddregRFVLKnendimp-kr<br>lqwhkddregRFLLK----------<br>lnwhiddregRFLLKnidqkttpie<br>.........................	Xt<br>Dr_ENSDARP00000039753<br>Ce_CE26729<br>Dm_FBpp0289752<br>Sc	
26	1	CI	ENSP00000283875<br>ENSG00000153767	19<br>19<br>19<br>19<br>20<br>19<br>19<br>32<br>33<br>14	evpaalkrlaKYVIRgfygiehala<br>evpaalkrlaKYVIRgfygiehala<br>evpaalkrlaKYVIRgfygiehvla<br>evpaalkrlaKYVIRgfygiehvla<br>evpaalkrlaKYVVRgfygiehala<br>evpavlkrlaKYVVRgfyglehala<br>evpaslkrlakqvvrgfygiehala<br>eipealntillmvvknffssehfii<br>evpsslkqlarlvvrgfysledali<br>piddivknllKFVVRgfyggsfvlv	Hs_ENSP00000283875<br>Bt_ENSBTAP00000050375<br>Rn_ENSRNOP00000003513<br>Mm_ENSMUSP00000023525<br>Gg_ENSGALP00000024066<br>Xt_ENSXETP00000021858<br>Dr_ENSDARP00000001151<br>Ce_CE16735<br>Dm_FBpp0075839<br>Sc_YKL028W	NP_005504 General transcription factor IIE subunit 1 (Transcription initiation factor IIE subunit alpha)(TFIIE-alpha)(General transcription factor IIE 56 kDa subunit)
27	1	CI	ENSP00000356140<br>ENSG00000163531	762<br>-<br>762<br>835<br>777<br>734<br>762<br>-<br>-<br>-	natsafgpnlRYIVKwrrretreaw<br>.........................<br>natsafgpnlRYIVKwrrretretw<br>natsafgpnlRYIVKwrrretretw<br>natqaygpnlRYIVRwrrrdprgsw<br>nstqaygpnlrynvkwrqrhtgepw<br>qnterngpnlryvlswrrkdteeew<br>.........................<br>.........................<br>.........................	Hs_ENSP00000356140<br>Bt<br>Rn_ENSRNOP00000057594<br>Mm_ENSMUSP00000035454<br>Gg_ENSGALP00000034591<br>Xt_ENSXETP00000009563<br>Dr_ENSDARP00000081155<br>Ce<br>Dm<br>Sc	Neurofascin Precursor
28	1	CI	ENSP00000273432<br>ENSG00000144893	1244<br>550<br>1244<br>1244<br>1212<br>-<br>-<br>-<br>1418<br>-	ietanlreyaRYVLRticqqewvge<br>ietanlreyaRYVLRticqqewvge<br>ietanlreyaRYVLRticqqewvge<br>ietanlreyaRYVLRticqqewvge<br>ietaslseyaRYVLRticqqewvge<br>.........................<br>.........................<br>.........................<br>meqisllefaqavlkqicaqehvle<br>.........................	Hs_ENSP00000273432<br>Bt_ENSBTAP00000010239<br>Rn_ENSRNOP00000014494<br>Mm_ENSMUSP00000042269<br>Gg_ENSGALP00000016876<br>Xt<br>Dr<br>Ce<br>Dm_FBpp0074653<br>Sc	NP_443728 Mediator of RNA polymerase II transcription subunit 12-like protein (Mediator complex subunit 12-like protein)(Thyroid hormone receptor-associated-like protein)(Trinucleotide repeat-containing gene 11 protein-like)
29	1	C	ENSP00000366549<br>ENSG00000115525	396<br>369<br>368<br>395<br>341<br>338<br>341<br>-<br>-<br>-	qtmhnvttetKFLLKlvkegvvkdl<br>qtmhnvttetnfllklvregvvrdl<br>qvmhnvttetqflqklikegvvqdl<br>qvmhnvttetKFLLKllkegvvedl<br>qtmhnvtgetkllqklvkekvvkdl<br>qpmhdvtkekkmlrtlvkegvvrdl<br>eamhnvqketvflkrlvasgsitdl<br>.........................<br>.........................<br>.........................	Hs_ENSP00000366549<br>Bt_ENSBTAP00000015414<br>Rn_ENSRNOP00000042426<br>Mm_ENSMUSP00000070414<br>Gg_ENSGALP00000031388<br>Xt_ENSXETP00000004222<br>Dr_ENSDARP00000054689<br>Ce<br>Dm<br>Sc	NP_003887 Lactosylceramide alpha-2,3-sialyltransferase (EC 2.4.99.9)(CMP-NeuAc:lactosylceramide alpha-2,3-sialyltransferase)(Ganglioside GM3 synthase)(ST3Gal V)(Sialyltransferase 9)
30	1	CI	ENSP00000325485<br>ENSG00000139624	66<br>66<br>66<br>66<br>67<br>58<br>58<br>-<br>62<br>86	giffvrllfeRFIAKpcalcigied<br>gvfsvrllfeRFIAKpcalhvgiqd<br>glfsvrmlfeRFIAKpcalrigied<br>cifsvrmlfeRFIAKpcalrvgikd<br>gifavrllfeRFIAKpcainlgiqd<br>gifavrllfeRFIAKpcalrvgiqs<br>gifavrifferfiagpcasmlqiqt<br>.........................<br>vvmlvrytlerfwispvgkslgirs<br>reisyrhawiaplmiliavysayft	Hs_ENSP00000325485<br>Bt_ENSBTAP00000023129<br>Rn_ENSRNOP00000026223<br>Mm_ENSMUSP00000023762<br>Gg_ENSGALP00000009966<br>Xt_ENSXETP00000029263<br>Dr_ENSDARP00000002973<br>Ce<br>Dm_FBpp0070898<br>Sc_YKL008C	NP_671723 LAG1 longevity assurance homolog 5
31	1	CI	ENSP00000329586<br>ENSG00000185238	367<br>346<br>347<br>347<br>332<br>329<br>331<br>-<br>337<br>-	mldsvlyaknKYLAKggsvypdict<br>mldsvlyaknKYLAKggsvypdict<br>mldsvlyaksKYLAKggsvypdict<br>mldsvlyaksKYLAKggsvypdict<br>mldsviyakdkylaeggsvypdict<br>mldsvicardkylnedgavypdtct<br>mldsvlyardryladdglvfpdrcs<br>.........................<br>mldsiiyarenhlnpngiilpsrct<br>.........................	Hs_ENSP00000329586<br>Bt_ENSBTAP00000017861<br>Rn_ENSRNOP00000039267<br>Mm_ENSMUSP00000082373<br>Gg_ENSGALP00000006299<br>Xt_ENSXETP00000030989<br>Dr_ENSDARP00000003829<br>Ce<br>Dm_FBpp0082588<br>Sc	Protein arginine N-methyltransferase 3 (EC 2.1.1.-)(Heterogeneous nuclear ribonucleoprotein methyltransferase-like protein 3)
32	1	CI	ENSP00000225719<br>ENSG00000108582	289<br>287<br>289<br>288<br>263	ysktsddevfKYLAKayasnhpimk<br>ysktsddevfRYLAKayashhpimk<br>ysktsddevfRYLAKayasnhpimr<br>ysktsddevfRYLAKayasnhpimk<br>ysksaddevfKYLAKayashhpimr	Hs_ENSP00000225719<br>Bt_ENSBTAP00000016421<br>Rn_ENSRNOP00000005262<br>Mm_ENSMUSP00000021201<br>Gg_ENSGALP00000006827	NP_001295 Carboxypeptidase D Precursor (EC 3.4.17.22)(Metallocarboxypeptidase D)(gp180)

				-<br>-<br>0<br>249<br>-	.........................<br>.........................<br>-------------------------<br>esltpddrvfkqlahtysdnhpimr<br>.........................	Xt<br>Dr<br>Ce_CE30548<br>Dm_FBpp0089126<br>Sc	
33	1	I	ENSP00000346193<br>ENSG00000188877	215<br>193<br>196<br>195<br>-<br>-<br>-<br>-<br>-<br>-	avhgqkqrmvKFLIKkkanlnaldr<br>giserkqqmveflvkkeadihavdk<br>alkqnkekmaeflihnganaktcdf<br>alkenkiemaKFLVKmganihvfdd<br>.........................<br>.........................<br>.........................<br>.........................<br>.........................<br>.........................	Hs_ENSP00000346193<br>Bt_ENSBTAP00000006062<br>Rn_ENSRNOP00000037532<br>Mm_ENSMUSP00000028577<br>Gg<br>Xt<br>Dr<br>Ce<br>Dm<br>Sc	NP_001005365 ANKRD26-like family A member 1 (Prostate, ovary, testis-expressed protein on chromosome 8)(POTE-8)
34	1	CI	ENSP00000342053<br>ENSG00000188848	412<br>417<br>364<br>-<br>255<br>259<br>-<br>-<br>-<br>-	sskkdgrrllRYLIRfvfttdelky<br>sskkdgrrllRYLIRfvfttdelky<br>sskkdgrrllRYLIRfvfttdelky<br>.........................<br>sskkdgrrllRYLIRfvfttdelky<br>sskkdgrrllRYLIRfvfttdelky<br>.........................<br>.........................<br>.........................<br>.........................	Hs_ENSP00000342053<br>Bt_ENSBTAP00000046715<br>Rn_ENSRNOP00000041878<br>Mm<br>Gg_ENSGALP00000022991<br>Xt_ENSXETP00000045548<br>Dr<br>Ce<br>Dm<br>Sc	NP_997289 Coiled-coil domain-containing protein 4
35	1	CI	ENSP00000325448<br>ENSG00000065427	271<br>269<br>272<br>270<br>240<br>255<br>256<br>246<br>259<br>246	dlilndfvrqKFIIRskiityirsf<br>dlilndfvrqKFIIRskiityirsf<br>dlilndfvrqKFIIRskiityirsf<br>dlilndfvrqKFIVRskiityirsf<br>dlilndyvrqkfitrakivtyirsf<br>dlilndyvrqKFIVRaklitylrkf<br>dlilndfvrqkfvtrskiitylrsf<br>dlilnprvkdnfvirskiitflrry<br>dlilnnnvrekfqirakiisyvrqf<br>dlimnkdarnrfitrseiiryirrf	Hs_ENSP00000325448<br>Bt_ENSBTAP00000020818<br>Rn_ENSRNOP00000044454<br>Mm_ENSMUSP00000090808<br>Gg_ENSGALP00000001332<br>Xt_ENSXETP00000054902<br>Dr_ENSDARP00000064925<br>Ce_CE28247<br>Dm_FBpp0071302<br>Sc_YDR037W	NP_001123561 Lysyl-tRNA synthetase (EC 6.1.1.6)(Lysine--tRNA ligase)(LysRS)
36	1	CI	ENSP00000309561<br>ENSG00000174917	10<br>10<br>-<br>10<br>-<br>-<br>10<br>-<br>-<br>-	-mvarvwslmRFLIKgsvaggavyl<br>-mvprvwslmRFLIKgsvaggavyl<br>.........................<br>-mvarvwslmRFLIKgsvaggavyl<br>.........................<br>.........................<br>-maakifpvvkfatkvtiaggalyv<br>.........................<br>.........................<br>.........................	Hs_ENSP00000309561<br>Bt_ENSBTAP00000002345<br>Rn<br>Mm_ENSMUSP00000052908<br>Gg<br>Xt<br>Dr_ENSDARP00000096088<br>Ce<br>Dm<br>Sc	NP_991330 Protein QIL1 (Protein P117)
37	1	CI	ENSP00000309520<br>ENSG00000174611	532<br>532<br>534<br>532<br>526<br>375<br>-<br>690<br>770<br>-	elkvqlphagKFALK----------<br>elkvqlphagKFALKifvkkrqepg<br>elkvqlphagKFALKifvkkrqeqg<br>elkvqlphagKFALKifvkkrqeqg<br>efkvhlphagsfvlkfftkkksdpg<br>tislhlpesgyyklsilsratgspd<br>.........................<br>yiqiefpgegqygldiytr------<br>tfiinfpeegqygldiytrelggp-<br>.........................	Hs_ENSP00000309520<br>Bt_ENSBTAP00000041518<br>Rn_ENSRNOP00000030738<br>Mm_ENSMUSP00000036032<br>Gg_ENSGALP00000040409<br>Xt_ENSXETP00000009480<br>Dr<br>Ce_CE32687<br>Dm_FBpp0085567<br>Sc	NP_848649 Kyphoscoliosis peptidase (EC 3.4.-.-)
38	1	CI	ENSP00000311399<br>ENSG00000157538	175<br>170<br>175<br>175<br>175<br>172<br>-<br>-<br>160<br>-	--vkerallpKFLLRghlnstncvi<br>--vkerallpKFLIRghlnstncvi<br>--vkeraslpKFLIRghlnstncai<br>--vkeraslpkffirghlnstncai<br>--vkeraslpKFLIRghlsstncvi<br>--vkeraslpRFLIRghldstncmi<br>.........................<br>.........................<br>asakerlsmprflitgrldrsefcv<br>.........................	Hs_ENSP00000311399<br>Bt_ENSBTAP00000047205<br>Rn_ENSRNOP00000002284<br>Mm_ENSMUSP00000023615<br>Gg_ENSGALP00000025816<br>Xt_ENSXETP00000028561<br>Dr<br>Ce<br>Dm_FBpp0077938<br>Sc	NP_006043 Down syndrome critical region protein 3 (Down syndrome critical region protein A)
39	1	CI	ENSP00000315417<br>ENSG00000180318	7<br>7<br>7<br>7<br>0	----meflseKFALKsppsknsdfy<br>----meflseKFALKsppsknsdfy<br>----meflseKFALKsppsknsdfy<br>----meflseKFALKsppsknsdfy<br>------------alksqpsknsdfy	Hs_ENSP00000315417<br>Bt_ENSBTAP00000019938<br>Rn_ENSRNOP00000006007<br>Mm_ENSMUSP00000042512<br>Gg_ENSGALP00000018154	NP_008913 ALX homeobox protein 1 (Cartilage homeoprotein 1)(CART-1)

				7	----meflteKFALKnqpskasdfy	Xt_ENSXETP00000046264	
				7	----meylsdkfslkspaikgsdyy	Dr_ENSDARP00000085677	
				0	-------------------------	Ce_CE42059	
				-	....	Dm	
				-	....	Sc	
40	1	CI	ENSP00000253458 ENSG00000131149	496	q---rtneeeKWLARqrrlrqeked	Hs_ENSP00000253458	NP_055430 Genetic suppressor element 1
				418	q---rtneeeKWLARqrrlrqeked	Bt_ENSBTAP00000013088	
				492	q---rtneeeKWLARqrrlrqeked	Rn_ENSRNOP00000023607	
				506	q---rtneeeKWLARqrrlrqeked	Mm_ENSMUSP00000034279	
				418	h---rtneeeKWLARqrrlrqeked	Gg_ENSGALP00000023057	
				494	q---rtqeeeKWLARqrrlrqeked	Xt_ENSXETP00000009697	
				487	atmlqrteeeRWLARqrklrqeked	Dr_ENSDARP00000083583	
				-	....	Ce	
				-	....	Dm	
				-	....	Sc	
41	1	CI	ENSP00000305875 ENSG00000171984	139	itqlrgqesqKYALRsfqmarvifn	Hs_ENSP00000305875	NP_689717 Uncharacterized protein C20orf196
				140	inelkdqenqtytlrsfqmarvifn	Bt_ENSBTAP00000039142	
				141	iselegqesqKYALRsfqmarvifs	Rn_ENSRNOP00000028891	
				140	iselegqesqRYALRsfqmaqvifs	Mm_ENSMUSP00000057009	
				143	iseladrdgtkyalhclqmaqlvln	Gg_ENSGALP00000031324	
				-	....	Xt	
				-	....	Dr	
				-	....	Ce	
				-	....	Dm	
				-	....	Sc	
42	1	CI	ENSP00000264951 ENSG00000114127	338	sghlnlprfeKYLVKlsdfdrehfs	Hs_ENSP00000264951	NP_061874 5'-3' exoribonuclease 1 (EC 3.1.11.-)(Strand-exchange protein 1 homolog)
				338	sghlnlprfeKYLVKlsdfdrehfs	Bt_ENSBTAP00000015318	
				338	sghlnlprfeRYLVKlsdfdrehfs	Rn_ENSRNOP00000014481	
				338	sghlnlprfeRYLVKlsdfdrehfs	Mm_ENSMUSP00000034981	
				338	nghlnlkrfekyltrlsdfdrehfs	Gg_ENSGALP00000004257	
				-	....	Xt	
				-	....	Dr	
				347	agylnlsrfeaflaelalndktsfm	Ce_CE42924	
				334	ngklnlrrlqifisalteveldhfk	Dm_FBpp0074553	
				337	hgkinlkrlgvwlnylsqfellnfe	Sc_YGL173C	
43	1	CI	ENSP00000360171 ENSG00000009694	2527	avpsvfgkgiKFAIKdgivtadiig	Hs_ENSP00000360171	NP_055068 Teneurin-1 (Ten-1)(Tenascin-M1)(Ten-m1)(Protein Odd Oz/ten-m homolog 1)
				-	....	Bt	
				2356	avpsvfgkgiKFAIKegivtadiig	Rn_ENSRNOP00000058433	
				2533	avpsvfgkgiKFAIKegivtadiig	Mm_ENSMUSP00000110711	
				2538	aipsvfgkgiKFAIKdgivtadiig	Gg_ENSGALP00000031671	
				-	....	Xt	
				2188	alssifgkgvKFAIRdgvvateiig	Dr_ENSDARP00000040183	
				-	....	Ce	
				-	....	Dm	
				-	....	Sc	
44	1	CI	ENSP00000333940 ENSG00000081803	295	kmakerk-fpKFIAKdmenmyieel	Hs_ENSP00000333940	NP_060424 Calcium-dependent secretion activator 2 (Calcium-dependent activator protein for secretion 2)(CAPS-2)
				294	kmakerr-fpKFIAKemesvyieel	Bt_ENSBTAP00000006737	
				298	kmakerr-fpqfiskemetmyieel	Rn_ENSRNOP00000010537	
				298	kmakerr-fprfiskemesmyieel	Mm_ENSMUSP00000018122	
				182	qmarerk-fpkfitremenmyieel	Gg_ENSGALP00000014492	
				-	....	Xt	
				-	....	Dr	
				-	....	Ce	
				380	emeknrklmpKFVLKemeslyveel	Dm_FBpp0088329	
				-	....	Sc	
45	1	CI	ENSP00000381210 ENSG00000100099	76	pptlvrlrklKFAIKvdgdylwvlg	Hs_ENSP00000381210	Hermansky-Pudlak syndrome 4 protein (Light-ear protein homolog)
				81	ppalvrlrklKFAVKvdgdylwvlg	Bt_ENSBTAP00000050301	
				81	ppmlirlrnlKFALRvdgdylwalg	Rn_ENSRNOP00000000824	
				81	ppmlirmrnlKFAIRadgdylwalg	Mm_ENSMUSP00000107978	
				81	ppslirlkklkfavildgdylwvlg	Gg_ENSGALP00000008962	
				-	....	Xt	
				78	pvrllrlrksKYAVRmkdnflwals	Dr_ENSDARP00000012323	
				-	....	Ce	
				95	p-rilalqngKFVLKefgrfilaig	Dm_FBpp0086017	
				-	....	Sc	
46	1	CI	ENSP00000302051 ENSG00000171551	258	tvslddrnssRYVIRidqdgltlpe	Hs_ENSP00000302051	NP_004817 Endothelin-converting enzyme-like 1 (EC 3.4.24.-)(Xce protein)
				252	tvslddrnssRYVIRvtep--repq	Bt_ENSBTAP00000028037	
				258	tvslddrnssRYVIRidqdgltlpe	Rn_ENSRNOP00000026388	
				258	tvslddrnssRYVIRidqdgltlpe	Mm_ENSMUSP00000027463	
				213	tvslddrntsRYVIRecactlegac	Gg_ENSGALP00000012777	

				247<br>87<br>-<br>-<br>-	tvslddknssRYIIridqdgltlpe<br>tvsvddknssrnairidqegltlpe<br>.........................<br>.........................<br>.........................	Xt_ENSXETP00000049287<br>Dr_ENSDARP00000079822<br>Ce<br>Dm<br>Sc	
47	1	CI	ENSP00000323580<br>ENSG00000032742	701<br>693<br>693<br>693<br>692<br>-<br>693<br>681<br>734<br>-	rkfpenveclRFLVRlctdlglkda<br>rkfpenveclRFLVRlctdiglkev<br>rkfpenveclRFLVRlctdiglkev<br>rkfpenveclRFLVRlctdiglkev<br>qkfpenveclRFLVRlctdmglkev<br>.........................<br>rkfpenveclRFLVRlctdmglkev<br>rkfpqdldclKFLVRiagdlgmtey<br>arfpdnltciralmqvtkslelgdl<br>.........................	Hs_ENSP00000323580<br>Bt_ENSBTAP00000018980<br>Rn_ENSRNOP00000012988<br>Mm_ENSMUSP00000113768<br>Gg_ENSGALP00000027626<br>Xt<br>Dr_ENSDARP00000034047<br>Ce_CE28361<br>Dm_FBpp0085279<br>Sc	NP_006522 Intraflagellar transport protein 88 homolog (Tetratricopeptide repeat protein 10)(TPR repeat protein 10)(Recessive polycystic kidney disease protein Tg737 homolog)
48	1	CI	ENSP00000302874<br>ENSG00000168447	50<br>11<br>5<br>5<br>11<br>10<br>-<br>70<br>-<br>-	hregatmhvkKYLLKglhrlqkgpg<br>vsagatmhvkKYLLKglhrlqkgpg<br>------mpvkKYLLKclhrlqkgpg<br>------mpvkKYLLKclhrlqkgpg<br>pladnrmnlkryfvralhrlqkgpg<br>-lqvkmkrlkryftralhriqkgpg<br>.........................<br>phfeeeddrsklhasalyserrtss<br>.........................<br>.........................	Hs_ENSP00000302874<br>Bt_ENSBTAP00000016301<br>Rn_ENSRNOP00000031470<br>Mm_ENSMUSP00000033161<br>Gg_ENSGALP00000009996<br>Xt_ENSXETP00000027176<br>Dr<br>Ce_CE26381<br>Dm<br>Sc	NP_000327 Amiloride-sensitive sodium channel subunit beta (Epithelial Na(+) channel subunit beta)(Beta-ENaC)(ENaCB)(SCNEB)(Nonvoltage-gated sodium channel 1 subunit beta)(Beta-NaCH)
49	1	CI	ENSP00000312419<br>ENSG00000111642	1808<br>1742<br>1789<br>1790<br>1759<br>-<br>1799<br>1583<br>1811<br>-	nrgnfleiknKFLARrfkllleqalv<br>nrgnfleiknKFLARrfkllleqalv<br>nrgnfleiknKFLARrfkllleqalv<br>nrgnfleiknKFLARrfkllleqalv<br>nrgnfleiknKFLARrfkllleqalv<br>.........................<br>nrgnfleiknKFLARrfkllleqalv<br>aeatgsdikakfmqrrfklieqslv<br>gkgnfleiknKFLARrfkllleqalv<br>.........................	Hs_ENSP00000312419<br>Bt_ENSBTAP00000019612<br>Rn_ENSRNOP00000024864<br>Mm_ENSMUSP00000108009<br>Gg_ENSGALP00000023294<br>Xt<br>Dr_ENSDARP00000087355<br>Ce_CE17716<br>Dm_FBpp0099808<br>Sc	Chromodomain-helicase-DNA-binding protein 4 (CHD-4)(EC 3.6.1.-)(ATP-dependent helicase CHD4)(Mi-2 autoantigen 218 kDa protein)(Mi2-beta)
50	1	C	ENSP00000354612<br>ENSG00000095303	179<br>195<br>181<br>182<br>171<br>215<br>180<br>-<br>-<br>-	qlpdaqllarRFLLRrkfipdpqgt<br>qlpdaeflsrRFLLRrkfipdpqgt<br>qlpdihllaqrlllrrefipapqgt<br>qlpdvqllaqqlllrrefipapqgt<br>qlpdaqllaqRFLLRhkfeadprgt<br>elpdasllvetflkrekfipdpqgt<br>klpdpkllvekfmlrrnfrldpqgt<br>.........................<br>.........................<br>.........................	Hs_ENSP00000354612<br>Bt_ENSBTAP00000008833<br>Rn_ENSRNOP00000010218<br>Mm_ENSMUSP00000059977<br>Gg_ENSGALP00000034260<br>Xt_ENSXETP00000035660<br>Dr_ENSDARP00000068435<br>Ce<br>Dm<br>Sc	NP_000953 Prostaglandin G/H synthase 1 Precursor (EC 1.14.99.1)(Cyclooxygenase-1)(COX-1)(Prostaglandin-endoperoxide synthase 1)(Prostaglandin H2 synthase 1)(PGH synthase 1)(PGHS-1)(PHS 1)
51	1	CI	ENSP00000337679<br>ENSG00000135250	217<br>-<br>165<br>173<br>173<br>126<br>-<br>-<br>-<br>255	vfevlghhllKWIIKsnyqglpvrc<br>.........................<br>vfevlghhllKWIIKsnyqglpvrc<br>vfevlghhllKWIIKsnyqglpvrc<br>vfevlghhllKWIIKsnyqglpirc<br>vlevlghqllKWIIKsnyegvplpc<br>.........................<br>.........................<br>.........................<br>vfevlgenllalikkyehrgipliy	Hs_ENSP00000337679<br>Bt<br>Rn_ENSRNOP00000014307<br>Mm_ENSMUSP00000085734<br>Gg_ENSGALP00000013230<br>Xt_ENSXETP00000001634<br>Dr<br>Ce<br>Dm<br>Sc_YMR216C	NP_872634 Serine/threonine-protein kinase SRPK2 (EC 2.7.11.1)(Serine/arginine-rich protein-specific kinase 2)(SR-protein-specific kinase 2)(SFRS protein kinase 2)
52	1	CI	ENSP00000296754<br>ENSG00000164307	579<br>580<br>568<br>568<br>440<br>561<br>593<br>-<br>-<br>-	fitsksdmvhRFLLKtktdvlilpe<br>fitsksdavqRFLLKtrtdvlilpe<br>fitsksdsvqRFLLKtktdviilpe<br>fitsksdsvqRFLLKtktdvlilpe<br>yitsksytverflmrtktdviilpe<br>yitsksntvqrfllmsktdvlvlae<br>yitsdsttvhRFLLKtktdvlylpe<br>.........................<br>.........................<br>.........................	Hs_ENSP00000296754<br>Bt_ENSBTAP00000018026<br>Rn_ENSRNOP00000013625<br>Mm_ENSMUSP00000022070<br>Gg_ENSGALP00000023617<br>Xt_ENSXETP00000028979<br>Dr_ENSDARP00000089298<br>Ce<br>Dm<br>Sc	NP_001035548 Endoplasmic reticulum aminopeptidase 1 (EC 3.4.11.-)(Adipocyte-derived leucine aminopeptidase)(A-LAP)(ARTS-1)(Aminopeptidase PILS)(Puromycin-insensitive leucyl-specific aminopeptidase)(PILS-AP)(Type 1 tumor necrosis factor receptor shedding aminopeptidase regulator)
53	1	CI	ENSP00000302468<br>ENSG00000168268	242<br>208<br>275	myqwieqdmeKYILRgdetfavlsr<br>myqwierdmeKYILRgdetfavlsr<br>myqwieqdmeKYILRgdetfavlsr	Hs_ENSP00000302468<br>Bt_ENSBTAP00000019665<br>Rn_ENSRNOP00000024840	NP_001127703 5'-nucleotidase domain-containing protein 2

				112<br>272<br>240<br>328<br>-<br>-<br>-	myqwieqdmeKYILRgdetfavlsr<br>mykwiekdmeqyilhgdeiyavlnr<br>mykwimqdleKYILRgdetyavlhr<br>mykwimqdleKYILRgdetyavlhr<br>.........................<br>.........................<br>.........................	Mm_ENSMUSP00000087680<br>Gg_ENSGALP00000002380<br>Xt_ENSXETP00000051665<br>Dr_ENSDARP00000077543<br>Ce<br>Dm<br>Sc	
54	1	I	ENSP00000313513<br>ENSG00000151687	881<br>881<br>964<br>965<br>-<br>823<br>-<br>-<br>-<br>-	qkafleksltKYLLKllkafqidvk<br>qrafleksls KYLLKllkafqidvk<br>qkaflerkltkdllkllkafqidvk<br>qkeflereltkdllkllqafqidvk<br>.........................<br>-------------------------<br>.........................<br>.........................<br>.........................<br>.........................	Hs_ENSP00000313513<br>Bt_ENSBTAP00000016667<br>Rn_ENSRNOP00000005314<br>Mm_ENSMUSP00000054056<br>Gg<br>Xt_ENSXETP00000021756<br>Dr<br>Ce<br>Dm<br>Sc	NP_653309 Ankyrin and armadillo repeat-containing protein
55	1	CI	ENSP00000361275<br>ENSG00000173846	634<br>635<br>635<br>636<br>626<br>649<br>648<br>-<br>-<br>-	gcspdlrqrlRYALRllrdrspa--<br>gcspdlrqrlhyalrllrdrcpv--<br>gcspdlrqrlRYALRllrdrspa--<br>gcspdlrqrlRYALRllrdqspa--<br>gcspelqhclRYILKllqewada--<br>gcssemyhrlrytvkllqqkaes--<br>gcspelrqrlhyvvqllqhytna--<br>.........................<br>.........................<br>.........................	Hs_ENSP00000361275<br>Bt_ENSBTAP00000008055<br>Rn_ENSRNOP00000025197<br>Mm_ENSMUSP00000076130<br>Gg_ENSGALP00000016453<br>Xt_ENSXETP00000029784<br>Dr_ENSDARP00000056597<br>Ce<br>Dm<br>Sc	NP_004064 Serine/threonine-protein kinase PLK3 (EC 2.7.11.21)(Polo-like kinase 3)(PLK-3)(Cytokine-inducible serine/threonine-protein kinase)(FGF-inducible kinase)(Proliferation-related kinase)
56	1	CI	ENSP00000355712<br>ENSG00000112584	142<br>149<br>142<br>142<br>-<br>142<br>143<br>170<br>-<br>-	fipsglavftRFALKtlgqetlcsl<br>fipsglaiftRFALKalgqetlcsl<br>fipsglaiftRFALKslgqetfcsl<br>fipsglaiftRFALKtlgqetfcsl<br>.........................<br>fipsgistftksalkslgqevilsh<br>clpsglatfsRFALKslgqetfcsv<br>aklggttvmkwfgnvvtpegkvcfv<br>.........................<br>.........................	Hs_ENSP00000355712<br>Bt_ENSBTAP00000005065<br>Rn_ENSRNOP00000002036<br>Mm_ENSMUSP00000054420<br>Gg<br>Xt_ENSXETP00000048740<br>Dr_ENSDARP00000049327<br>Ce_CE09907<br>Dm<br>Sc	NP_115824 Constitutive coactivator of peroxisome proliferator-activated receptor gamma (Constitutive coactivator of PPAR-gamma)(Protein FAM120B)
57	1	CI	ENSP00000386869<br>ENSG00000187605	799<br>4<br>926<br>84<br>90<br>31<br>217<br>-<br>-<br>-	gkssrgcpiaKWVIRrhtleekllc<br>-------paspqvirrhtleekllc<br>gkssqgcpiakwvyrrssteekllc<br>gkssqgcpvaKWVIRrsgpeeklic<br>gkssqgcpiaKWVIRrssdeekllc<br>gkssrgcpiaKWVIRrqsedeklmc<br>gkssqgcpiaKWVLRrssekekvlc<br>.........................<br>.........................<br>.........................	Hs_ENSP00000386869<br>Bt_ENSBTAP00000044564<br>Rn_ENSRNOP00000048956<br>Mm_ENSMUSP00000097267<br>Gg_ENSGALP00000006468<br>Xt_ENSXETP00000026387<br>Dr_ENSDARP00000085229<br>Ce<br>Dm<br>Sc	NP_659430 Protein TET3
58	1	CI	ENSP00000376016<br>ENSG00000092820	254<br>254<br>253<br>254<br>251<br>254<br>254<br>-<br>-<br>-	eirnisfndkKFVIKpidkkapdfv<br>eirnisfndkKFVIKpidkkapdfv<br>eirnisfndkKFVIKpidkkapdfv<br>eirnisfndkKFVIKpidkkapdfv<br>eirnisfndkKFVIKpidkkapdfv<br>eirnisfndkKFVIKpidkkapdfv<br>eirnisfndkKFVIKpidkkapdfv<br>.........................<br>.........................<br>.........................	Hs_ENSP00000376016<br>Bt_ENSBTAP00000013663<br>Rn_ENSRNOP00000025122<br>Mm_ENSMUSP00000063734<br>Gg_ENSGALP00000022293<br>Xt_ENSXETP00000021538<br>Dr_ENSDARP00000021505<br>Ce<br>Dm<br>Sc	Ezrin (p81)(Cytovillin)(Villin-2)
59	1	CI	ENSP00000356070<br>ENSG00000162889	89<br>90<br>86<br>75<br>17<br>70<br>70<br>-<br>45<br>-	lqifnkrtqeKFALKmlqdcpkarr<br>lqifskrtqeKFALKmlqdcpkarr<br>lrifdkrtqqKFALKmlqdcp-arr<br>lrifdkrtqqKFALKmlqdcpkarr<br>leifskksgeKFALKmlqdcpkarr<br>leifskksgekfamkmlqdcakarr<br>leifykrsgeklalkmlhdcpkarr<br>.........................<br>vqcthrrtqqnyalkvlldserarr<br>.........................	Hs_ENSP00000356070<br>Bt_ENSBTAP00000015530<br>Rn_ENSRNOP00000057786<br>Mm_ENSMUSP00000016672<br>Gg_ENSGALP00000001295<br>Xt_ENSXETP00000051087<br>Dr_ENSDARP00000020470<br>Ce<br>Dm_FBpp0070802<br>Sc	NP_116584 MAP kinase-activated protein kinase 2 (MAPK-activated protein kinase 2)(MAPKAP kinase 2)(MAPKAPK-2)(MK2)(EC 2.7.11.1)
60	1	CI	ENSP00000366886<br>ENSG00000162413	159<br>168<br>159	scsglasaaqRFILRhvgelga-eq<br>scaglasaaqRFILRhvgelga-eq<br>scsglasaaqRFILRhvgelga-eq	Hs_ENSP00000366886<br>Bt_ENSBTAP00000010998<br>Rn_ENSRNOP00000004508	NP_055666 Kelch-like protein 21

				159<br>161<br>169<br>161<br>-<br>-<br>-	scsglasaaqRFILRhvgelga-eq<br>acpalaaaahRFILRhvselg--aq<br>acqglaestkrfilahmaqlaqahe<br>acrelaasarRFVLKnivelaksmd<br>.........................<br>.........................<br>.........................	Mm_ENSMUSP00000095380<br>Gg_ENSGALP00000000899<br>Xt_ENSXETP00000024281<br>Dr_ENSDARP00000057356<br>Ce<br>Dm<br>Sc	
61	1	CI	ENSP00000362748<br>ENSG00000138336	1486<br>-<br>1393<br>-<br>-<br>-<br>-<br>-<br>1512<br>-	gksshgcpiaKWVLRrssdeekvlc<br>.........................<br>gkssqgcpvaKWVIRrsgpeekvic<br>.........................<br>.........................<br>.........................<br>.........................<br>.........................<br>anpqsnggqgtgnqpgqlmssnssl<br>.........................	Hs_ENSP00000362748<br>Bt<br>Rn_ENSRNOP00000054747<br>Mm<br>Gg<br>Xt<br>Dr<br>Ce<br>Dm_FBpp0288425<br>Sc	NP_085128 Protein TET1 (Ten-eleven translocation 1 gene protein)(CXXC-type zinc finger protein 6)(Leukemia-associated protein with a CXXC domain)
62	1	CI	ENSP00000377112<br>ENSG00000221955	215<br>113<br>176<br>176<br>192<br>0<br>-<br>185<br>231<br>-	allginlagvKWIIRlqllllflla<br>allginlagvKWIIRlqlllllla<br>allginlagvKWIIRlqlllslla<br>allginlagvKWIIRlqlllllla<br>gllginlagvKWIIRlqllllflla<br>-------------------------<br>.........................<br>vltavntagvtwvvrlqivllltia<br>llgcinvagvKWVIKlqfillmill<br>.........................	Hs_ENSP00000377112<br>Bt_ENSBTAP00000018208<br>Rn_ENSRNOP00000002439<br>Mm_ENSMUSP00000112439<br>Gg_ENSGALP00000019644<br>Xt_ENSXETP00000050523<br>Dr<br>Ce_CE04364<br>Dm_FBpp0070226<br>Sc	NP_078904 Solute carrier family 12 member 8 (Cation-chloride cotransporter 9)
63	1	CI	ENSP00000304668<br>ENSG00000170445	53<br>56<br>53<br>53<br>51<br>0<br>60<br>59<br>78<br>52	gpdes---kqKFVLKtpkgtrdysp<br>gpdeg---kpKFVLKtpkgtrdysp<br>ghdeg---kqKFVLKtpksnhhyfv<br>gqdeg---kqKFVLKtpkgtrdysp<br>ggdeg---khKFVLKtpkgtrdygp<br>-------------------------<br>ggdeg---khvfvlktakgtrdynp<br>getgapekpgKFVLKtgkgtrdygp<br>ggdaap-tnqkftlktpkgtrdygp<br>apkkg---klqvslktpkgtkdwad	Hs_ENSP00000304668<br>Bt_ENSBTAP00000025094<br>Rn_ENSRNOP00000034118<br>Mm_ENSMUSP00000001416<br>Gg_ENSGALP00000001150<br>Xt_ENSXETP00000005727<br>Dr_ENSDARP00000024132<br>Ce_CE33829<br>Dm_FBpp0074366<br>Sc_YPR033C	NP_002100 Histidyl-tRNA synthetase, cytoplasmic (EC 6.1.1.21)(Histidine--tRNA ligase)(HisRS)
64	1	C	ENSP00000301293<br>ENSG00000167680	456<br>456<br>-<br>457<br>452<br>442<br>429<br>-<br>476<br>-	flgseagtvlKFLVRpnastsgtsg<br>flgseagtvlkflvwpnasasgttg<br>.........................<br>flgseagtvlKFLVKpnasvsgttg<br>flgsstgtilkflihpnatsspapa<br>flasdsgtvlkyllipkmsgseigt<br>flgssrgtilkflvtpnrdntvtnn<br>.........................<br>fvgtdhgkiiksvnaesadsadkvt<br>.........................	Hs_ENSP00000301293<br>Bt_ENSBTAP00000003621<br>Rn<br>Mm_ENSMUSP00000001256<br>Gg_ENSGALP00000001772<br>Xt_ENSXETP00000047775<br>Dr_ENSDARP00000083460<br>Ce<br>Dm_FBpp0110230<br>Sc	NP_115484 Semaphorin-6B Precursor (Semaphorin-Z)(Sema Z)
65	1	CI	ENSP00000201979<br>ENSG00000088320	270<br>270<br>269<br>269<br>181<br>-<br>270<br>-<br>418<br>-	rpaslaqrarRFLARltarsarrra<br>rraslgqrarRFLARltarsarrra<br>rraslgqrarRFLARltarsarrra<br>rraslgqrarRFLARltarsarrra<br>rkesltkrarrfldrmvarnsskva<br>.........................<br>rkesitkkarrfldrlvaknnkkma<br>.........................<br>spahwyksrsvmlasmkarqmltwi<br>.........................	Hs_ENSP00000201979<br>Bt_ENSBTAP00000018843<br>Rn_ENSRNOP00000010149<br>Mm_ENSMUSP00000000369<br>Gg_ENSGALP00000038582<br>Xt<br>Dr_ENSDARP00000005231<br>Ce<br>Dm_FBpp0111558<br>Sc	NP_054731 GTP-binding protein REM 1 (Rad and Gem-like GTP-binding protein 1)(GTPase-regulating endothelial cell sprouting)
66	1	CI	ENSP00000301624<br>ENSG00000078687	1479<br>697<br>-<br>1639<br>1473<br>1477<br>-<br>-<br>1001<br>-	tinttiqdvnRYLLKsgg-------<br>tinttiqdvnRYLLKsggssppssq<br>.........................<br>tinttiqdvnRYLLKsgg-------<br>tinttiqdvnRYLLKsgg-------<br>tinttiqdvnRYLLKsggnglsncn<br>.........................<br>.........................<br>lsinstpkdadifantgkn------<br>.........................	Hs_ENSP00000301624<br>Bt_ENSBTAP00000034597<br>Rn<br>Mm_ENSMUSP00000026658<br>Gg_ENSGALP00000002643<br>Xt_ENSXETP00000042192<br>Dr<br>Ce<br>Dm_FBpp0088168<br>Sc	NP_061869 Trinucleotide repeat-containing gene 6C protein
67	1	CI	ENSP00000295133<br>ENSG00000163013	144<br>133<br>142	epglvpaaaaRYALReieiplgelf<br>epglvp--aaRYALReieiplgelf<br>epglvp--aaRYALReieiplgelf	Hs_ENSP00000295133<br>Bt_ENSBTAP00000016959<br>Rn_ENSRNOP00000042876	NP_001073879 F-box only protein 41

				142<br>103<br>98<br>-<br>-<br>-<br>-	epglvp--aaRYALReieiplgelf<br>papsss--pspryvheieipltеif<br>psss-------rytqeveipladif<br>.........................<br>.........................<br>.........................<br>.........................	Mm_ENSMUSP00000058653<br>Gg_ENSGALP00000025874<br>Xt_ENSXETP00000025759<br>Dr<br>Ce<br>Dm<br>Sc	
67	2	CI	ENSP00000295133<br>ENSG00000163013	580<br>569<br>575<br>578<br>506<br>514<br>-<br>-<br>-<br>-	lhaaevcrdwRFVARhpavwtrvll<br>lhaaevcrdwRFVARhpavwtrvll<br>lhaaevcrdwRFVARhpavwtrvll<br>lhaaevcrdwRFVARhpavwtrvll<br>lraaevckdwKFVARhpavwtrvll<br>lraaevsrdwKFVARhpavwtrvfl<br>.........................<br>.........................<br>.........................<br>.........................	Hs_ENSP00000295133<br>Bt_ENSBTAP00000016959<br>Rn_ENSRNOP00000042876<br>Mm_ENSMUSP00000058653<br>Gg_ENSGALP00000025874<br>Xt_ENSXETP00000025759<br>Dr<br>Ce<br>Dm<br>Sc	NP_001073879 F-box only protein 41
68	1	CI	ENSP00000326022<br>ENSG00000112214	19<br>19<br>19<br>19<br>19<br>-<br>-<br>-<br>-<br>-	qyctasllgkKYVLKddspycvtcy<br>qycmasllgkKYVLKddnpycvscy<br>qyctasllgkKYVLKddnlyciscy<br>qyctssligkKYVLKddnlyciscy<br>hyclqslrgrKYALKeenaycvrcy<br>.........................<br>.........................<br>.........................<br>.........................<br>.........................	Hs_ENSP00000326022<br>Bt_ENSBTAP00000002359<br>Rn_ENSRNOP00000047630<br>Mm_ENSMUSP00000103839<br>Gg_ENSGALP00000025060<br>Xt<br>Dr<br>Ce<br>Dm<br>Sc	NP_065228 Four and a half LIM domains protein 5 (FHL-5) (Activator of cAMP-responsive element modulator in testis) (Activator of CREM in testis)
69	1	CI	ENSP00000247087<br>ENSG00000126705	381<br>-<br>-<br>380<br>-<br>-<br>-<br>-<br>-<br>-	hgppgpeghpKYALRrtdrpkilcr<br>.........................<br>.........................<br>hgppgpeghpKYALRrtdrpkilcr<br>.........................<br>.........................<br>.........................<br>.........................<br>.........................<br>.........................	Hs_ENSP00000247087<br>Bt<br>Rn<br>Mm_ENSMUSP00000101536<br>Gg<br>Xt<br>Dr<br>Ce<br>Dm<br>Sc	NP_001025053 A.T hook DNA-binding motif-containing protein 1
70	1	CI	ENSP00000295956<br>ENSG00000136068	2342<br>2347<br>2342<br>2342<br>2246<br>2360<br>2248<br>-<br>-<br>-	echvselepdKYAVRfiphengvht<br>echvselepdKYAVRfiphengiht<br>echvselepdKYAVRfiphengiht<br>echvselepdKYAVRfiphengiht<br>echvselepdKYAVRfiphengihs<br>ecfvtelepdKYAVRfmprengihm<br>ecavseleqdKYAIRfiprengihv<br>.........................<br>.........................<br>.........................	Hs_ENSP00000295956<br>Bt_ENSBTAP00000029348<br>Rn_ENSRNOP00000012796<br>Mm_ENSMUSP00000052020<br>Gg_ENSGALP00000009093<br>Xt_ENSXETP00000018966<br>Dr_ENSDARP00000080431<br>Ce<br>Dm<br>Sc	NP_001448 Filamin-B (FLN-B) (Beta-filamin)(Actin-binding-like protein)(Thyroid autoantigen) (Truncated actin-binding protein) (Truncated ABP)(ABP-280 homolog)(ABP-278)(Filamin 3) (Filamin homolog 1)(Fh1)
71	1	CI	ENSP00000268124<br>ENSG00000140521	1138<br>1122<br>1116<br>1116<br>858<br>1104<br>1108<br>985<br>1042<br>895	rfcisihdevRYLVReedryraala<br>rfcisihdevRYLVReedryraala<br>rfcisihdevRYLVReedryraala<br>rfcisihdevRYLVReedryraala<br>rfcisihdevrylvqeqdryraala<br>rfcisihdevrylvqskdryraala<br>rfcisihdevrylvtsedryraala<br>rfvisihdevrymckepdaprlala<br>rfclsfhdelRYLVKeelspkaala<br>rlcisihdeirflvsekdkyraama	Hs_ENSP00000268124<br>Bt_ENSBTAP00000011993<br>Rn_ENSRNOP00000047900<br>Mm_ENSMUSP00000035345<br>Gg_ENSGALP00000037807<br>Xt_ENSXETP00000038527<br>Dr_ENSDARP00000080832<br>Ce_CE25501<br>Dm_FBpp0080148<br>Sc_YOR330C	NP_001119603 DNA polymerase subunit gamma-1 (EC 2.7.7.7) (Mitochondrial DNA polymerase catalytic subunit)(PolG-alpha)
72	1	CI	ENSP00000353408<br>ENSG00000147065	254<br>254<br>253<br>254<br>254<br>254<br>254<br>256<br>326<br>-	eirnisfndkKFVIKpidkkap--d<br>eirnisfndkKFVIKpidkkap--d<br>eirnisfndkKFVIKpidkkap--d<br>eirnisfndkKFVIKpidkkap--d<br>eirnisfndkKFVIKpidkkap--d<br>eirnisfndkKFVIKpidkkap--d<br>eirnisfndkKFVIKpidkksppqd<br>eirnisfndkKFVIKpidkkah--d<br>eirnisfsekKFIIKpidkkap--d<br>.........................	Hs_ENSP00000353408<br>Bt_ENSBTAP00000017503<br>Rn_ENSRNOP00000044138<br>Mm_ENSMUSP00000113071<br>Gg_ENSGALP00000007490<br>Xt_ENSXETP00000021526<br>Dr_ENSDARP00000042830<br>Ce_CE07806<br>Dm_FBpp0071213<br>Sc	NP_002435 Moesin (Membrane-organizing extension spike protein)
73	1	CI	ENSP00000360431<br>ENSG00000138193	2230<br>1827<br>2209	qaqskwkgagKFILKlkeqvqasre<br>qaqskwkgagKFILKlkeqvqasre<br>qaqskwkgagKFILKlkeqvqasre	Hs_ENSP00000360431<br>Bt_ENSBTAP00000025249<br>Rn_ENSRNOP00000019771	NP_057425 1-phosphatidylinositol-4,5-bisphosphate phosphodiesterase

				2210<br>-<br>-<br>-<br>1828<br>-<br>-	qaqskwkgagKFILKlkeqvqasre<br>.........................<br>.........................<br>.........................<br>tpkypnnrttsrvlgqnenvwkaqs<br>.........................<br>.........................	Mm_ENSMUSP00000025962<br>Gg<br>Xt<br>Dr<br>Ce_CE28227<br>Dm<br>Sc	epsilon-1 (EC 3.1.4.11) (Phospholipase C-epsilon-1)(PLC-epsilon-1)(Phosphoinositide-specific phospholipase C epsilon-1)(Pancreas-enriched phospholipase C)
74	1	CI	ENSP00000268129<br>ENSG00000140526	53<br>53<br>53<br>53<br>55<br>53<br>59<br>-<br>40<br>-	dlyfqdsglsRFLLKscplltkeyi<br>dlyfqdsglsRFLLKscplltkeyi<br>dlyfqdsglsRFLLKscplltkeyi<br>dlyfqdsglsRFLLKscplltkeyi<br>eliyqdsalsRFLLKscplltkeyi<br>ellyqdtaltRYLLKscplltkeyi<br>ditcqdtplnhyllkscpvltkeyi<br>.........................<br>svwcldahfldclykiapvlrepyi<br>.........................	Hs_ENSP00000268129<br>Bt_ENSBTAP00000026579<br>Rn_ENSRNOP00000023506<br>Mm_ENSMUSP00000038361<br>Gg_ENSGALP00000010798<br>Xt_ENSXETP00000038546<br>Dr_ENSDARP00000067326<br>Ce<br>Dm_FBpp0077265<br>Sc	NP_690888 Abhydrolase domain-containing protein 2 (EC 3.1.1.-) (Lung alpha/beta hydrolase 2) (Protein PHPS1-2)
75	1	CI	ENSP00000360065<br>ENSG00000152763	620<br>625<br>578<br>579<br>503<br>486<br>563<br>-<br>-<br>-	vsisadgrisKWVIRkgldcydlmr<br>vsisadgrisKWVIRkgldchdlmr<br>vsisadgrisKWVIRkgldchdlmh<br>vsisadgrisKWIIRkgldchgheh<br>isisadgritqwfiqeglgcidlmk<br>vsvcadgritrwhirkgldcndlmk<br>vsvssdgriskwihyksmecvdlmk<br>.........................<br>.........................<br>.........................	Hs_ENSP00000360065<br>Bt_ENSBTAP00000007160<br>Rn_ENSRNOP00000048851<br>Mm_ENSMUSP00000112018<br>Gg_ENSGALP00000018083<br>Xt_ENSXETP00000043934<br>Dr_ENSDARP00000065205<br>Ce<br>Dm<br>Sc	NP_996897 WD repeat-containing protein 78
76	1	CI	ENSP00000254854<br>ENSG00000132518	677<br>682<br>680<br>680<br>-<br>-<br>-<br>-<br>-<br>-	lksrncivdgRFVLKitdhghgrll<br>lksrncvvdgRFVLKvtdhghgrll<br>lksrncvvdgRFVLKvtdhghgrll<br>lksrncvvdgRFVLKvtdhghgrll<br>.........................<br>.........................<br>.........................<br>.........................<br>.........................<br>.........................	Hs_ENSP00000254854<br>Bt_ENSBTAP00000011564<br>Rn_ENSRNOP00000010790<br>Mm_ENSMUSP00000104305<br>Gg<br>Xt<br>Dr<br>Ce<br>Dm<br>Sc	NP_000171 Retinal guanylyl cyclase 1 Precursor (RETGC-1) (EC 4.6.1.2)(Guanylate cyclase 2D, retinal)(Rod outer segment membrane guanylate cyclase) (ROS-GC)
77	1	CI	ENSP00000285518<br>ENSG00000155189	116<br>43<br>116<br>116<br>116<br>115<br>116<br>125<br>-<br>-	qnalg---hvRYVLKeglkwlplyg<br>qsalg---hvRYVLKdglkwlplyg<br>qdalg---hvRYVLKdglkwlplyg<br>qdalg---hvRYVLKdklkwlplyg<br>qnalg---hvRYVLKdglkwlplyg<br>qnalg---hvRYVLKdglkflplyg<br>qnaig---hvRYVLKdglkwlplyg<br>hgdqgneqafrvmvknsihlvpmfg<br>.........................<br>.........................	Hs_ENSP00000285518<br>Bt_ENSBTAP00000006476<br>Rn_ENSRNOP00000037069<br>Mm_ENSMUSP00000033847<br>Gg_ENSGALP00000026290<br>Xt_ENSXETP00000034836<br>Dr_ENSDARP00000086538<br>Ce_CE37364<br>Dm<br>Sc	NP_060831 1-acyl-sn-glycerol-3-phosphate acyltransferase epsilon (EC 2.3.1.51)(1-AGP acyltransferase 5)(1-AGPAT 5) (Lysophosphatidic acid acyltransferase epsilon)(LPAAT-epsilon)(1-acylglycerol-3-phosphate O-acyltransferase 5)
78	1	CI	ENSP00000360069<br>ENSG00000124203	940<br>-<br>486<br>917<br>-<br>-<br>-<br>-<br>-<br>-	saladnafspKYLLRlpqaetplpl<br>.........................<br>taqsgvsfapKYLLRlpqgenplsl<br>narsggffppKYLLRlpqgenpspi<br>.........................<br>.........................<br>.........................<br>.........................<br>.........................<br>.........................	Hs_ENSP00000360069<br>Bt<br>Rn_ENSRNOP00000030895<br>Mm_ENSMUSP00000060255<br>Gg<br>Xt<br>Dr<br>Ce<br>Dm<br>Sc	NP_848552 Zinc finger protein 831
79	1	CI	ENSP00000354912<br>ENSG00000198934	916<br>-<br>892<br>877<br>-<br>-<br>-<br>-<br>-<br>-	rletskmkalRYVARihrkepqdwp<br>.........................<br>rletskmkalRYVARihrkepqdwp<br>rletskmkalRYVARihrkepedwp<br>.........................<br>.........................<br>.........................<br>.........................<br>.........................<br>.........................	Hs_ENSP00000354912<br>Bt<br>Rn_ENSRNOP00000054396<br>Mm_ENSMUSP00000094078<br>Gg<br>Xt<br>Dr<br>Ce<br>Dm<br>Sc	NP_065983 Melanoma-associated antigen E1 (MAGE-E1 antigen) (Hepatocellular carcinoma-associated protein 1)
80	1	CI	ENSP00000381107<br>ENSG00000150760	747<br>747<br>733	kamkalesifKFIVRsrilfnqlye<br>kamkaleyifKFIVRsrilfnqlye<br>kaikaleyifKFIVRsrvlfnqlye	Hs_ENSP00000381107<br>Bt_ENSBTAP00000032659<br>Rn_ENSRNOP00000051781	Dedicator of cytokinesis protein 1 (180 kDa protein downstream of

				747	kaikaleyifKFIVRsrvlfnqlye	Mm_ENSMUSP00000081531	CRK)(DOCK180)
				732	kamkaleyifKFIVRsrilfnqlye	Gg_ENSGALP00000001117	
				729	kamkaleyifKFIVRsrvlfnqlye	Xt_ENSXETP00000011369	
				-	....	Dr	
				735	nafkvmgplfklvvvskkcgikfee	Ce_CE24779	
				774	kttrylhyvmKFVIRsrvlyaemnc	Dm_FBpp0083850	
				-	....	Sc	
80	2	CI	ENSP00000381107 ENSG00000150760	1227	killehcrkhKYLAKtg-etfvklv	Hs_ENSP00000381107	Dedicator of cytokinesis protein 1 (180 kDa protein downstream of CRK)(DOCK180)
				1161	killehcrkhKYLAKtg-etfvklv	Bt_ENSBTAP00000032659	
				1147	killehcrkhKYLAKtg-etfvklv	Rn_ENSRNOP00000051781	
				1161	killehcrkhKYLAKtg-etfvklv	Mm_ENSMUSP00000081531	
				1219	killehcrkhKYLAKsg-etfvklv	Gg_ENSGALP00000001117	
				1206	killehcrkhKYLAKng-enfvtlv	Xt_ENSXETP00000011369	
				-	....	Dr	
				1168	qlsitlcqsdkelmanggeelieri	Ce_CE24779	
				1205	timlercaahntlnvdg-tafvqmv	Dm_FBpp0083850	
				-	....	Sc	
81	1	CI	ENSP00000261800 ENSG00000086570	394	vrvtpafpnlqyvlkpssenvgfkl	Hs_ENSP00000261800	NP_001438 Protocadherin Fat 2 Precursor (hFat2)(Multiple epidermal growth factor-like domains 1)
				390	vkvtpafpnlKYVLKpssesaafkl	Bt_ENSBTAP00000004553	
				394	vkvttalpnlryslkpssrntafkl	Rn_ENSRNOP00000055459	
				394	vrvttalptlRYALKpssgstvfkl	Mm_ENSMUSP00000067556	
				393	vkitpvlpnlKYVLRptpdsthfki	Gg_ENSGALP00000006868	
				-	....	Xt	
				388	vsvglslknttfsiqenpdsykfki	Dr_ENSDARP00000014029	
				-	....	Ce	
				-	....	Dm	
				-	....	Sc	
81	2	C	ENSP00000261800 ENSG00000086570	888	tghldresepRYILKveardqpskg	Hs_ENSP00000261800	NP_001438 Protocadherin Fat 2 Precursor (hFat2)(Multiple epidermal growth factor-like domains 1)
				884	tghldresepRYILKveardqprkg	Bt_ENSBTAP00000004553	
				888	tghldresesqyilkaeardqptkg	Rn_ENSRNOP00000055459	
				888	tgqldresepqyilkaeardqptkg	Mm_ENSMUSP00000067556	
				886	igaldrelwpyyalkikardqpktg	Gg_ENSGALP00000006868	
				-	....	Xt	
				880	igpldretfrrydltieardqakpd	Dr_ENSDARP00000014029	
				-	....	Ce	
				-	....	Dm	
				-	....	Sc	
81	3	CI	ENSP00000261800 ENSG00000086570	2979	rktldrehtaKYLLRvtasdgkfqa	Hs_ENSP00000261800	NP_001438 Protocadherin Fat 2 Precursor (hFat2)(Multiple epidermal growth factor-like domains 1)
				2977	rktldrehtaKYLLRitasdgkfqa	Bt_ENSBTAP00000004553	
				2983	rktldrehiaKYLLRvtasdgkfqa	Rn_ENSRNOP00000055459	
				2981	rktldrehiaKYLLRitasdgkfqa	Mm_ENSMUSP00000067556	
				2976	kkpldredteKYLLKvmasdgkfqa	Gg_ENSGALP00000006868	
				-	....	Xt	
				2964	kspldredkdkytlkiiatdgrfqt	Dr_ENSDARP00000014029	
				-	....	Ce	
				-	....	Dm	
				-	....	Sc	
82	1	C	ENSP00000354957 ENSG00000198887	618	rlkqiytaeeKYVVKtsfysn-kvi	Hs_ENSP00000354957	NP_055925 Structural maintenance of chromosomes protein 5 (hSMC5)
				-	....	Bt	
				621	rlkqiytaeeKYVLKtsfysn-kvi	Rn_ENSRNOP00000047365	
				618	rlkqiytaeeKYVLKtsvysn-kvi	Mm_ENSMUSP00000045389	
				598	klkqiytaeekytikvstytk-lsf	Gg_ENSGALP00000037418	
				-	....	Xt	
				-	....	Dr	
				586	rysvfltanircqnsksryan-ntl	Ce_CE27096	
				582	r--vyfggskkfvvtasryrs-dti	Dm_FBpp0089228	
				620	lfkriihgnrlvdikqsaygskqvf	Sc_YOL034W	
83	1	CI	ENSP00000261804 ENSG00000113716	1222	vcphqvvcgsKYLVRgesardhvdl	Hs_ENSP00000261804	Protein SMF
				1204	vcphqvvcgsKYLVRgesaldhvdl	Bt_ENSBTAP00000035035	
				1070	vcphqvvcgsKYLVRgesardhvdl	Rn_ENSRNOP00000024704	
				1071	vcphqvvcgsKYLVRgesardhvdl	Mm_ENSMUSP00000025524	
				1157	vcphqvvcgsKYIVRgenardhvdl	Gg_ENSGALP00000009253	
				1031	vcphqvacgsKYIVRgesprdhvdl	Xt_ENSXETP00000034446	
				-	....	Dr	
				-	....	Ce	
				-	....	Dm	
				-	....	Sc	
84	1	CI	ENSP00000238522 ENSG00000164309	3240	tddgtgiyfeKYILKddilhdtslt	Hs_ENSP00000238522	NP_705838 Cardiomyopathy-associated protein 5 (Myospryn)(Dystrobrevin-binding protein 2)
				2692	tddgtgiyfeKYILKddilhdtcvt	Bt_ENSBTAP00000008967	
				2859	tddgtgmyfeKYVLKddilhdesvn	Rn_ENSRNOP00000038462	

				2805<br>-<br>-<br>-<br>-<br>-<br>-	tddgpgmyfeKYILKddilhdetvt<br>.........................<br>.........................<br>.........................<br>.........................<br>.........................<br>.........................	Mm_ENSMUSP00000050408<br>Gg<br>Xt<br>Dr<br>Ce<br>Dm<br>Sc	(SPRY domain-containing protein 2)(Tripartite motif-containing protein 76)(Genethonin-3)
85	1	C	ENSP00000379399<br>ENSG00000160746	189<br>190<br>189<br>189<br>185<br>156<br>189<br>-<br>187<br>-	lkkledtwytRFALKyqp----ids<br>lkkledtwytrftlkfqp----idr<br>lkkledtwytRFALKyqp----ids<br>lkkledtwytRFALKyqp----ids<br>lkklrhtwygrvkigyqpl---ade<br>lkrlrhqwylriklafqp----idq<br>lkrlsfswykkiklsfqp----ldd<br>.........................<br>fplhdilylerfnwnlkrtklpied<br>.........................	Hs_ENSP00000379399<br>Bt_ENSBTAP00000011710<br>Rn_ENSRNOP00000029074<br>Mm_ENSMUSP00000045214<br>Gg_ENSGALP00000018747<br>Xt_ENSXETP00000047668<br>Dr_ENSDARP00000094943<br>Ce<br>Dm_FBpp0074146<br>Sc	Anoctamin-10 (Transmembrane protein 16K)
86	1	CI	ENSP00000359370<br>ENSG00000075826	610<br>610<br>439<br>595<br>614<br>543<br>-<br>-<br>-<br>-	gtdllkqtqeRYLAKkktkisslla<br>gsdllkqaqeRYLAKkktgiaplla<br>daellkwtqeRYLAKrrtktsslla<br>daellkwtqeRYLAKrrtktsslla<br>genllketqkryfakrktklsllls<br>gedllretqqwyfsqqknkittlls<br>.........................<br>.........................<br>.........................<br>.........................	Hs_ENSP00000359370<br>Bt_ENSBTAP00000000099<br>Rn_ENSRNOP00000038038<br>Mm_ENSMUSP00000064900<br>Gg_ENSGALP00000039343<br>Xt_ENSXETP00000031054<br>Dr<br>Ce<br>Dm<br>Sc	NP_056305 Protein transport protein Sec31B (SEC31-related protein B)(SEC31B-1)(SEC31-like 2)
87	1	CI	ENSP00000218172<br>ENSG00000102053	218<br>222<br>-<br>281<br>283<br>212<br>267<br>-<br>-<br>-	qgrrvvcyddRFIVKlafdsdgiiv<br>qgrrvvcyddRFIVKlafdsdgiiv<br>.........................<br>qgrrvvcyddRFIVKlafdsdgiiv<br>qgrrvvcyddRFIVKlafdsdgiiv<br>qgrrvvcyddRFIVKlafdcdgiiv<br>qgrrvvcyddRFIVKlacdsdgiiv<br>.........................<br>.........................<br>.........................	Hs_ENSP00000218172<br>Bt_ENSBTAP00000002858<br>Rn<br>Mm_ENSMUSP00000080820<br>Gg_ENSGALP00000007499<br>Xt_ENSXETP00000038586<br>Dr_ENSDARP00000084808<br>Ce<br>Dm<br>Sc	Zinc finger CCCH domain-containing protein 12B (MCP-induced protein 2)
88	1	CI	ENSP00000314543<br>ENSG00000176774	135<br>132<br>119<br>119<br>-<br>-<br>-<br>-<br>-<br>-	kepitkgdmiKFVIRkdkchfneil<br>repikkadmlKFVIKqyknhfneil<br>kevitkadmlksviktsknhfneil<br>kevitkadmlKYVIKtsknhfneil<br>.........................<br>.........................<br>.........................<br>.........................<br>.........................<br>.........................	Hs_ENSP00000314543<br>Bt_ENSBTAP00000049423<br>Rn_ENSRNOP00000034674<br>Mm_ENSMUSP00000109588<br>Gg<br>Xt<br>Dr<br>Ce<br>Dm<br>Sc	NP_775970 Melanoma-associated antigen B18 (MAGE-B18 antigen)
89	1	CI	ENSP00000304590<br>ENSG00000169575	66<br>-<br>66<br>66<br>-<br>-<br>-<br>-<br>-<br>-	wyqqrpghppRFLLRyfsqsdksqg<br>.........................<br>wyqqrpghpptfllrffshsdklqg<br>wyqqrpghppRFLLRyfshsdkhqg<br>.........................<br>.........................<br>.........................<br>.........................<br>.........................<br>.........................	Hs_ENSP00000304590<br>Bt<br>Rn_ENSRNOP00000002517<br>Mm_ENSMUSP00000074537<br>Gg<br>Xt<br>Dr<br>Ce<br>Dm<br>Sc	NP_009059 Immunoglobulin iota chain Precursor (V(pre)B protein)(VpreB protein)(CD179 antigen-like family member A)(CD179a antigen)
90	1	CI	ENSP00000242591<br>ENSG00000122970	120<br>120<br>120<br>120<br>120<br>120<br>120<br>121<br>-<br>-	nelkkraylaRFLIKlevpseflqd<br>nelkkraylaRFLIKlevpseflqd<br>selkkraylaRFLIKlevpseflqd<br>nelkkraylaRFLIKlevpseflqd<br>nelkkraylaRFLVKldvpveflqd<br>ndlkkraylsRFLVRlevpeeflqd<br>pelkkraylaRFLVKlevpaeflqd<br>eglkeraylskylmktdvpgeffdy<br>.........................<br>.........................	Hs_ENSP00000242591<br>Bt_ENSBTAP00000043560<br>Rn_ENSRNOP00000001742<br>Mm_ENSMUSP00000031426<br>Gg_ENSGALP00000006116<br>Xt_ENSXETP00000045267<br>Dr_ENSDARP00000015953<br>Ce_CE43247<br>Dm<br>Sc	NP_054774 Intraflagellar transport protein 81 homolog (Carnitine deficiency-associated protein expressed in ventricle 1)(CDV-1)
91	1	CI	ENSP00000352276<br>ENSG00000000460	184<br>209<br>243	mdhafhantwKFIIKqslkhqsiik<br>mdhafhantwKFIIKqslkhqsvik<br>mdkafhantwKFIIKqslkhqsvir	Hs_ENSP00000352276<br>Bt_ENSBTAP00000019683<br>Rn_ENSRNOP00000003534	NP_060656 Uncharacterized protein C1orf112

				241<br>240<br>183<br>234<br>-<br>-<br>-	mdqafhantwKFIIKqslkhhsvik<br>mdhalhantwKFIIKqslkhhallq<br>mdhalhantwKFIIKqslkhhasve<br>ldialhantwKFIIKqsikyqslve<br>.........................<br>.........................<br>.........................	Mm_ENSMUSP00000095101<br>Gg_ENSGALP00000005314<br>Xt_ENSXETP00000010267<br>Dr_ENSDARP00000061728<br>Ce<br>Dm<br>Sc	
92	1	CI	ENSP00000377862<br>ENSG00000196159	1509<br>1509<br>1509<br>1509<br>1487<br>-<br>0<br>-<br>1581<br>-	ndqavpietrRYALKnvtilvtdln<br>ndqavpietrRYALKnvtilvtdln<br>ndqavpietrRYALKnvtilvtdln<br>ndqavpietrRYALKnvtilvtdln<br>tdqavpvesrRFALKnvtilvmdqn<br>.........................<br>-------------------------<br>.........................<br>tdraqpserqlsteklvtvivedin<br>.........................	Hs_ENSP00000377862<br>Bt_ENSBTAP00000013316<br>Rn_ENSRNOP00000036219<br>Mm_ENSMUSP00000061836<br>Gg_ENSGALP00000019273<br>Xt<br>Dr_ENSDARP00000086913<br>Ce<br>Dm_FBpp0077167<br>Sc	NP_078858 Protocadherin Fat 4 Precursor (hFat4)(FAT tumor suppressor homolog 4)(Fat-like cadherin protein FAT-J)
93	1	CI	ENSP00000352608<br>ENSG00000196218	3539<br>-<br>3538<br>3540<br>-<br>3533<br>3575<br>-<br>-<br>-	dqdlitlaktRYALKdtdeevrefl<br>.........................<br>dqdlivlakaRYALKdtdeevrefl<br>dqdlivlakaRYALKdtdeevrefl<br>.........................<br>dqelitlaktryslkdtdeevrefl<br>dqelinlaktryslkdtdeevrefl<br>.........................<br>.........................<br>.........................	Hs_ENSP00000352608<br>Bt<br>Rn_ENSRNOP00000027893<br>Mm_ENSMUSP00000032813<br>Gg<br>Xt_ENSXETP00000006174<br>Dr_ENSDARP00000032856<br>Ce<br>Dm<br>Sc	NP_000531 Ryanodine receptor 1 (RYR-1)(RyR1)(Skeletal muscle-type ryanodine receptor)(Skeletal muscle calcium release channel)
94	1	CI	ENSP00000373767<br>ENSG00000189056	1867<br>1687<br>1634<br>1868<br>1759<br>-<br>-<br>-<br>-<br>-	tntmyvqfslRFIAKstpershsil<br>tntmyvqfslRFIAKstpershsil<br>tntmyvqfslRFIAKgtpershsil<br>tntmyvqfslRFIAKgtpershsil<br>tntvyiqfsfKFIAKgtpershsil<br>.........................<br>.........................<br>.........................<br>.........................<br>.........................	Hs_ENSP00000373767<br>Bt_ENSBTAP00000004768<br>Rn_ENSRNOP00000010521<br>Mm_ENSMUSP00000058025<br>Gg_ENSGALP00000013329<br>Xt<br>Dr<br>Ce<br>Dm<br>Sc	NP_005036 Reelin Precursor (EC 3.4.21.-)
95	1	CI	ENSP00000284384<br>ENSG00000154229	38<br>0<br>0<br>38<br>38<br>-<br>-<br>180<br>47<br>-	qknvhevkdhKFIARffkqptfcsh<br>-------------------------<br>-------------------------<br>qknvhevkdhKFIARffkqptfcsh<br>qknvhevkehKFIARffkqptfcsh<br>.........................<br>.........................<br>qknvheikshKFIARffkqptfcsh<br>kknvfnvkdhcfiarffkqptfcsh<br>.........................	Hs_ENSP00000284384<br>Bt_ENSBTAP00000001407<br>Rn_ENSRNOP00000051949<br>Mm_ENSMUSP00000062392<br>Gg_ENSGALP00000006278<br>Xt<br>Dr<br>Ce_CE30929<br>Dm_FBpp0086197<br>Sc	NP_002728 Protein kinase C alpha type (PKC-alpha)(PKC-A)(EC 2.7.11.13)
96	1	CI	ENSP00000232978<br>ENSG00000114857	294<br>294<br>-<br>294<br>293<br>-<br>288<br>-<br>-<br>-	peeippvpenRFLLRrdmpvvtaep<br>peeippvpenRFLLRrdmplvtvep<br>.........................<br>peeippvpenRFLLRrdmpaitvep<br>peeippvpenRFLLRrdvpvvstep<br>.........................<br>peeippvpenRFLLRrdmptqeeai<br>.........................<br>.........................<br>.........................	Hs_ENSP00000232978<br>Bt_ENSBTAP00000030831<br>Rn<br>Mm_ENSMUSP00000035112<br>Gg_ENSGALP00000015981<br>Xt<br>Dr_ENSDARP00000076525<br>Ce<br>Dm<br>Sc	NP_005376 NK-tumor recognition protein (Natural-killer cells cyclophilin-related protein)(NK-TR protein)
97	1	CI	ENSP00000357880<br>ENSG00000123505	76<br>80<br>76<br>76<br>83<br>78<br>76<br>91<br>74<br>96	sessmfvskrRFILKtcgttlllka<br>sessmfvskrRFILKtcgttlllka<br>sessmfvskrRFILKtcgttlllka<br>sessmfvskrRFILKtcgttlllka<br>sessmfvskrRFILKtcgttlllqa<br>sessmfvskrRFILKtcgttlllqa<br>sessmfvskrRFILKtcgttlllqa<br>sesslfisdnrvilktcgttrllaa<br>sessmfvskrRWILKtcgtttplkc<br>sesslfvfdhkltmktcgttttlfc	Hs_ENSP00000357880<br>Bt_ENSBTAP00000004338<br>Rn_ENSRNOP00000000715<br>Mm_ENSMUSP00000097528<br>Gg_ENSGALP00000024221<br>Xt_ENSXETP00000046135<br>Dr_ENSDARP00000064390<br>Ce_CE18705<br>Dm_FBpp0079584<br>Sc_YOL052C	NP_001028231 S-adenosylmethionine decarboxylase proenzyme (AdoMetDC)(SamDC)(EC 4.1.1.50)
98	1	CI	ENSP00000311505<br>ENSG00000173226	429<br>429<br>429	aavtlqraalKFLAKcrkkkklfap<br>aavilqratlKFLAKcrkkkklfap<br>aavtlqravlKFLAKcrkkkklfas	Hs_ENSP00000311505<br>Bt_ENSBTAP00000017379<br>Rn_ENSRNOP00000003223	NP_001018864 IQ calmodulin-binding motif-containing protein 1 (Nephrocystin-5)

				429<br>429<br>426<br>430<br>-<br>-<br>-	aavtlqravlKFLAKcrkkkklfas<br>aaviiqraackflek-rrrkrtlss<br>aavciqraalrflnrrrkmresltp<br>aavtlqravllflkrrkaqrniltp<br>.........................<br>.........................<br>.........................	Mm_ENSMUSP00000023535<br>Gg_ENSGALP00000019025<br>Xt_ENSXETP00000030447<br>Dr_ENSDARP00000075835<br>Ce<br>Dm<br>Sc	
99	1	CI	ENSP00000278456<br>ENSG00000149177	1219<br>-<br>1099<br>1233<br>1250<br>-<br>-<br>-<br>-<br>-	pdttdllinfRYLVRdymkqsppes<br>.........................<br>-------------------------<br>pdttdllinfRYLVRdymkqippes<br>pettdllinfrhlvheyssqnpids<br>.........................<br>.........................<br>.........................<br>.........................<br>.........................	Hs_ENSP00000278456<br>Bt<br>Rn_ENSRNOP00000054119<br>Mm_ENSMUSP00000026352<br>Gg_ENSGALP00000010295<br>Xt<br>Dr<br>Ce<br>Dm<br>Sc	NP_002834 Receptor-type tyrosine-protein phosphatase eta Precursor (Protein-tyrosine phosphatase eta)(R-PTP-eta)(EC 3.1.3.48)(HPTP eta)(Protein-tyrosine phosphatase receptor type J)(Density-enhanced phosphatase 1)(DEP-1)(CD148 antigen)
100	1	CI	ENSP00000309757<br>ENSG00000175445	40<br>43<br>40<br>40<br>12<br>37<br>59<br>-<br>-<br>-	qrrdfidiesKFALRtpedtaedtc<br>ggkdfrdiesKFALRtpedtaedtc<br>ggrdfsdiesKFALRtpedtaedtc<br>agrdfsdiesKFALRtpedtaedtc<br>----fegieskfslrtpaepdedvc<br>--tdfnsieskfslrtleepdddtc<br>wmmdftdieskfsfrtleepeddlc<br>.........................<br>.........................<br>.........................	Hs_ENSP00000309757<br>Bt_ENSBTAP00000017086<br>Rn_ENSRNOP00000016543<br>Mm_ENSMUSP00000015712<br>Gg_ENSGALP00000036979<br>Xt_ENSXETP00000056503<br>Dr_ENSDARP00000096482<br>Ce<br>Dm<br>Sc	NP_000228 Lipoprotein lipase Precursor (LPL)(EC 3.1.1.34)
101	1	CI	ENSP00000237353<br>ENSG00000118557	913<br>915<br>928<br>929<br>-<br>-<br>-<br>-<br>-<br>-	klgnqlreqvKYIAKlsgekdhlhs<br>klgnklreqvKYIAKltgekdhlhn<br>klgnklreqvKYIAKltgekdhlhn<br>klgnklreqvKYIAKltgekdhlhn<br>.........................<br>.........................<br>.........................<br>.........................<br>.........................<br>.........................	Hs_ENSP00000237353<br>Bt_ENSBTAP00000017642<br>Rn_ENSRNOP00000056035<br>Mm_ENSMUSP00000034162<br>Gg<br>Xt<br>Dr<br>Ce<br>Dm<br>Sc	NP_112583 Polyamine-modulated factor 1-binding protein 1 (PMF-1-binding protein)
102	1	CI	ENSP00000264326<br>ENSG00000114480	162<br>0<br>162<br>162<br>148<br>147<br>0<br>141<br>141<br>150	eilyrispwaKYVVRe------gdn<br>------------------------x<br>eilyrispwaKYVVRe------nnn<br>eilyrispwaKYVVRe------nnn<br>ellyrispwaRYVVRy------egk<br>etlyrispwaKYVIRe------dnk<br>-------------------------<br>thfk-lspwatfvtcp----npket<br>qlldrlspwakyvvqppk--sanqg<br>skifrlpawitratqpsketskqfg	Hs_ENSP00000264326<br>Bt_ENSBTAP00000047423<br>Rn_ENSRNOP00000039448<br>Mm_ENSMUSP00000023393<br>Gg_ENSGALP00000024956<br>Xt_ENSXETP00000008729<br>Dr_ENSDARP00000064740<br>Ce_CE01076<br>Dm_FBpp0086845<br>Sc_YEL011W	NP_000149 1,4-alpha-glucan-branching enzyme (EC 2.4.1.18) (Glycogen-branching enzyme) (Brancher enzyme)
103	1	CI	ENSP00000364464<br>ENSG00000148120	594<br>-<br>597<br>596<br>599<br>-<br>-<br>-<br>-<br>-	vhylkgyfllRFLAKrlgdetyfsf<br>.........................<br>vhylkgyfllRFLARtlgeetyfpf<br>vhylkgyfllrfltrtlgekiyfpf<br>vhylkgffllrflastlgeasylas<br>.........................<br>.........................<br>.........................<br>.........................<br>.........................	Hs_ENSP00000364464<br>Bt<br>Rn_ENSRNOP00000023638<br>Mm_ENSMUSP00000089148<br>Gg_ENSGALP00000020566<br>Xt<br>Dr<br>Ce<br>Dm<br>Sc	Aminopeptidase O (AP-O)(EC 3.4.11.-)
104	1	CI	ENSP00000368976<br>ENSG00000140859	255<br>192<br>222<br>253<br>-<br>149<br>134<br>120<br>-<br>-	aslraqsppvKYVIKtvevessktk<br>aslraqsppiKYVIKtvevessktk<br>aslkaqsppvKYVIKtvevessktk<br>aslraqsppvKYVIKtvevessktk<br>.........................<br>q------pqikymvktvevdsaktk<br>rr-httvpqvkyvtktvevesaqck<br>rqlrtgrppppstqrstatsslkps<br>.........................<br>.........................	Hs_ENSP00000368976<br>Bt_ENSBTAP00000032379<br>Rn_ENSRNOP00000019582<br>Mm_ENSMUSP00000034240<br>Gg<br>Xt_ENSXETP00000030134<br>Dr_ENSDARP00000071718<br>Ce_CE12298<br>Dm<br>Sc	NP_005541 Kinesin-like protein KIFC3
105	1	I	ENSP00000385543<br>ENSG00000010932	387<br>387<br>387	smiptgetqaRWAVRvlkgvnklpp<br>sllptgdtqaRWAVRvlkgviklpp<br>smiptgetqarwvvqvlkgattlpp	Hs_ENSP00000385543<br>Bt_ENSBTAP00000028540<br>Rn_ENSRNOP00000051361	NP_002012 Dimethylaniline monooxygenase

				387	smvptgetqarwvvqvlkgattlpp	Mm_ENSMUSP00000037259	
				-	....	Gg	
				-	....	Xt	
				-	....	Dr	
				-	....	Ce	
				-	....	Dm	
				-	....	Sc	
106	1	CI	ENSP00000337455 ENSG00000115568	1577	ceqcgkafktRFLLRthlrkhseak	Hs_ENSP00000337455	NP_001099007 Zinc finger protein 142 (HA4654)
				1556	ceqcgkafktRFLLRthlrkhseak	Bt_ENSBTAP00000041539	
				1614	ceqcgkafktRFLLRthlrkhseak	Rn_ENSRNOP00000035123	
				1742	ceqcgkafktRFLLRthlrkhseak	Mm_ENSMUSP00000109368	
				1626	ceqcgkafktRFLLKthlkkhseek	Gg_ENSGALP00000018561	
				1347	ceqcgkafktRFLLKthmrkhseek	Xt_ENSXETP00000030278	
				-	....	Dr	
				1231	hsicqsakfrlengviqipefqtek	Ce_CE30487	
				-	....	Dm	
				-	....	Sc	
107	1	CI	ENSP00000381034 ENSG00000170677	505	mqvrslqylcRFVIRqytridliqk	Hs_ENSP00000381034	NP_004223 Suppressor of cytokine signaling 6 (SOCS-6)(Suppressor of cytokine signaling 4)(SOCS-4)(Cytokine-inducible SH2 protein 4)(CIS-4)
				495	mqvrslqylcRFVIRqytridliqk	Bt_ENSBTAP00000023144	
				502	mqvrslqylcRFVIRqytridliqk	Rn_ENSRNOP00000054982	
				503	mqvrslqylcRFVIRqytridliqk	Mm_ENSMUSP00000064929	
				504	mqvrslqylcRFVIRqytridliqk	Gg_ENSGALP00000036145	
				503	mqvrslqylcRFVIRqytridliqk	Xt_ENSXETP00000023594	
				489	mqvrslqylcRFVIRqytridliqk	Dr_ENSDARP00000075890	
				-	....	Ce	
				-	....	Dm	
				-	....	Sc	
108	1	CI	ENSP00000306344 ENSG00000166575	406	evqtlrpsywKFLLRltkgkfavmn	Hs_ENSP00000306344	NP_075069 Transmembrane protein 135
				363	evqtlrpsywKFLLRltkgrfavmn	Bt_ENSBTAP00000051499	
				406	evqnlrpsywKFLLRltkgrfalmn	Rn_ENSRNOP00000022578	
				406	evqnlrpsywKFLLRltkgrfalmn	Mm_ENSMUSP00000042783	
				406	evqnlrpsywKFLLRltkgrfalmn	Gg_ENSGALP00000027818	
				218	evqnlrpsywkflqrltkgrfalmn	Xt_ENSXETP00000022899	
				413	evqnlrpsywKFLLRltkgrk----	Dr_ENSDARP00000091203	
				393	eprairrgylnflfglvggkislfn	Ce_CE31029	
				404	epkslrpsyykflqaisgdrlskfn	Dm_FBpp0081358	
				-	....	Sc	
109	1	CI	ENSP00000302655 ENSG00000170085	615	kqphnvrdviKWLVKavtedgltqp	Hs_ENSP00000302655	Uncharacterized protein C5orf25
				388	kqphnvrdviKWLIRavtedrliqp	Bt_ENSBTAP00000020753	
				371	kqphnvrdviKWLVKavtenaltpp	Rn_ENSRNOP00000043680	
				1101	kqphnvrdviKWLVKavteneltpp	Mm_ENSMUSP00000113676	
				-	....	Gg	
				-	....	Xt	
				-	....	Dr	
				-	....	Ce	
				-	....	Dm	
				-	....	Sc	
110	1	CI	ENSP00000354476 ENSG00000198911	635	ryslqklrlvRWLLKkvfqcrratp	Hs_ENSP00000354476	NP_004590 Sterol regulatory element-binding protein 2 (SREBP-2)(Sterol regulatory element-binding transcription factor 2)
				605	ryslqklrlvRWLLKkvfqhrraap	Bt_ENSBTAP00000018956	
				627	ryslqklrlvRWLLKkvfqrwratp	Rn_ENSRNOP00000052893	
				624	ryslqklrlvRWLLKkvfqrwratp	Mm_ENSMUSP00000023100	
				404	ryslqklalvRWLLKrtshqwrare	Gg_ENSGALP00000019416	
				595	rcslqkislvRWLLKhspg-----y	Xt_ENSXETP00000037964	
				619	ryclrrpaplgwlvrlvggrhe---	Dr_ENSDARP00000087122	
				638	rhllnwlwigRYIARrrrsttkp--	Ce_CE19180	
				-	....	Dm	
				-	....	Sc	
111	1	CI	ENSP00000350786 ENSG00000072182	523	mvripnrgsaRYLARkynrnetyir	Hs_ENSP00000350786	NP_061144 Amiloride-sensitive cation channel 4 (Amiloride-sensitive cation channel 4, pituitary)(Acid-sensing ion channel 4)
				396	mvripnrgsaRYLARkynrnetyir	Bt_ENSBTAP00000027364	
				396	mvkipnrgsaRYLARkynrnetyir	Rn_ENSRNOP00000027135	
				396	mvkipnrgsaRYLARkynrnetyir	Mm_ENSMUSP00000045598	
				-	....	Gg	
				-	....	Xt	
				394	mvkipsrgsarylsrkyqkseeyir	Dr_ENSDARP00000095487	
				-	....	Ce	
				-	....	Dm	
				-	....	Sc	
112	1	CI	ENSP00000348580 ENSG00000196656	38	rcvpkdkaikKFVIRniveaaavrd	Hs_ENSP00000348580	NP_001087200 40S ribosomal protein S26
				-	....	Bt	
				-	....	Rn	

				38	rcvpkdkaikKFVIRniveaaavrd	Mm_ENSMUSP00000076447	
				-	....	Gg	
				-	....	Xt	
				38	rcvpkdkaikKFVIRniveaaavrd	Dr_ENSDARP00000053878	
				-	....	Ce	
				-	....	Dm	
				38	ksipkdkaikrmairniveaaavrd	Sc_YGL189C	
113	1	CI	ENSP00000240285 ENSG00000121039	24	vlwafvlaaaRWLVRpkeksvagqv	Hs_ENSP00000240285	NP_742034 Retinol dehydrogenase 10 (EC 1.1.1.n2)
				25	vlwafvlaaaRWLVRpkeksvagqv	Bt_ENSBTAP00000026830	
				24	vlwafvlaaaRWLVRpkeksvagqv	Rn_ENSRNOP00000009096	
				24	vlwafvlaaaRWLVRpkeksvagqv	Mm_ENSMUSP00000027053	
				24	vlwafvlaaaKWLVRpkeksvagqv	Gg_ENSGALP00000025169	
				24	vlwafvlaaaKWLVRpkdksvagqv	Xt_ENSXETP00000025854	
				24	vcwaivmagfKWLIRpkeksvagqv	Dr_ENSDARP00000066702	
				-	....	Ce	
				-	....	Dm	
				-	....	Sc	
114	1	CI	ENSP00000327054 ENSG00000176619	500	geeiaykftpKYILRagqmvtvwaa	Hs_ENSP00000327054	NP_116126 Lamin-B2
				513	geeisykftpKYVLRagqtvtvwaa	Bt_ENSBTAP00000018110	
				516	gediaykftpKYVLRagqtvtvwaa	Rn_ENSRNOP00000022336	
				496	gediaykftpKYVLRagqtvtvwaa	Mm_ENSMUSP00000057291	
				516	geeiaykftpKYVLRagqtvtiwga	Gg_ENSGALP00000034565	
				526	eeeivykftpKYVLKagqsvkiysa	Xt_ENSXETP00000003751	
				493	geeivykfspKFVLKagqtvtvwsa	Dr_ENSDARP00000004139	
				514	krenvytlprdfvlragktlkifar	Ce_CE31506	
				-	....	Dm	
				-	....	Sc	
115	1	CI	ENSP00000379203 ENSG00000155097	245	ddfrhkarenKFIVRdfqyneeemk	Hs_ENSP00000379203	NP_001686 V-type proton ATPase subunit C 1 (V-ATPase subunit C 1)(Vacuolar proton pump subunit C 1)
				245	ddfrhkarenKFIVRdfqyneeemk	Bt_ENSBTAP00000017968	
				256	ddfrhkarenKFIVRdfqyneeemr	Rn_ENSRNOP00000054207	
				245	ddfrhkarenKFIVRdfqyneeemk	Mm_ENSMUSP00000022904	
				251	ddfkhkareyKFLVRdfqyneeemk	Gg_ENSGALP00000025836	
				245	ddfrhkarenKFVVRdfqyneeemk	Xt_ENSXETP00000007595	
				245	ddfrhkarenkftvrdfqyneeemk	Dr_ENSDARP00000029343	
				247	defkntarenKFIVRdfvydeetlk	Ce_CE29997	
				694	eefklharerKFIVRdfvyneeela	Dm_FBpp0288470	
				254	qefttaarekkfiprefnyseelid	Sc_YKL080W	
116	1	CI	ENSP00000261366 ENSG00000113368	487	--svsykytsRYVLKagqtvtiwaa	Hs_ENSP00000261366	NP_005564 Lamin-B1
				487	--svsykytsRYVLKagqtvtvssa	Bt_ENSBTAP00000003740	
				487	--svsykytsRYVLKagqtvtvwaa	Rn_ENSRNOP00000019351	
				488	--svsykytsRYVLKagqtvtvwaa	Mm_ENSMUSP00000025486	
				487	--sasyrytsRYVLKagqtvtiwaa	Gg_ENSGALP00000036945	
				487	--svnfkftsRYVLKagqtvtiwaa	Xt_ENSXETP00000011292	
				503	--tatykftakynlkagqkvtiwas	Dr_ENSDARP00000065036	
				492	--easfqfssrmklaphasatvwsa	Ce_CE15746	
				520	gpsttykfhrsvriepngvitvwsa	Dm_FBpp0078733	
				-	....	Sc	
117	1	CI	ENSP00000234313 ENSG00000115956	265	ghrrknwkvrKFILRedpaylhyyd	Hs_ENSP00000234313	NP_002655 Pleckstrin (Platelet p47 protein)
				267	ghrrknwkvrKFILRedpaylhyyd	Bt_ENSBTAP00000012724	
				265	ghrrknwkvrKFILRedpaylhyyd	Rn_ENSRNOP00000007044	
				265	ghrrknwkvrKFILRedpaylhyyd	Mm_ENSMUSP00000099945	
				267	ghrrknwkvrKFVLRedpaylhyyd	Gg_ENSGALP00000014229	
				278	ghrrknwkvrKFVLRddpaylhyyd	Xt_ENSXETP00000037796	
				262	ghrrknwkvrKFILRddpayihyyd	Dr_ENSDARP00000003766	
				-	....	Ce	
				-	....	Dm	
				-	....	Sc	
118	1	CI	ENSP00000251334 ENSG00000108506	689	ssslmdqipiKFLIRqaqglqqelg	Hs_ENSP00000251334	NP_065799 Integrator complex subunit 2 (Int2)
				683	ssslmdqipiKFLIRqaqglqqelg	Bt_ENSBTAP00000016011	
				683	ssslmdqipiKFLIRqaqglqqelg	Rn_ENSRNOP00000004876	
				683	ssslmdqipiKFLIRqaqglqqelg	Mm_ENSMUSP00000103674	
				682	ssalmdqipiKYLIRqaqglqqelg	Gg_ENSGALP00000008442	
				680	ssammdqipiKYLIRqaqglqqelg	Xt_ENSXETP00000023464	
				692	saalmdqipikhlirqaqglqqelg	Dr_ENSDARP00000091001	
				707	dksiflklpvryilsvmeyqhekfe	Ce_CE15203	
				652	snnflgglplkyllqkahhyhndyl	Dm_FBpp0074254	
				-	....	Sc	
119	1	C	ENSP00000317027 ENSG00000176928	399	svciygaaelRWLIKdghwfankfd	Hs_ENSP00000317027	NP_057675 Beta-1,3-galactosyl-O-glycosyl-glycoprotein beta-1,6-N-acetylglucosaminyltransferase 4
				313	svciygaaelrwlmkyghwfankfd	Bt_ENSBTAP00000003605	
				387	svciygaaelRWLIKeghwfankfd	Rn_ENSRNOP00000047764	

				-<br>400<br>300<br>386<br>-<br>-<br>-	.........................<br>svciygaaelrwlmnyghwfankfd<br>svciygagelrwlmtyghwfankfd<br>svciygaaelrwlledghwfankfd<br>.........................<br>.........................<br>.........................	Mm<br>Gg_ENSGALP00000024061<br>Xt_ENSXETP00000011078<br>Dr_ENSDARP00000088235<br>Ce<br>Dm<br>Sc	(EC 2.4.1.102)(Core 2-branching enzyme 3)(Core2-GlcNAc-transferase 3)(C2GnT3)
120	1	C	ENSP00000307443<br>ENSG00000196600	173<br>-<br>173<br>-<br>-<br>-<br>-<br>-<br>-<br>-	yghlsdrfgrKFVLRwsylqlaivg<br>.........................<br>ygpltdrfgrrlilicaslqmavte<br>.........................<br>.........................<br>.........................<br>.........................<br>.........................<br>.........................<br>.........................	Hs_ENSP00000307443<br>Bt<br>Rn_ENSRNOP00000024234<br>Mm<br>Gg<br>Xt<br>Dr<br>Ce<br>Dm<br>Sc	NP_955384 Solute carrier family 22 member 25 (Organic anion transporter UST6)
121	1	CI	ENSP00000220676<br>ENSG00000104237	1745<br>-<br>1715<br>1709<br>1632<br>-<br>-<br>-<br>-<br>-	ieegvlidkgKWLLKenhllrmss-<br>.........................<br>kekgklidngKWLLRenhlwrvsl-<br>kengklidngKWLLRenhlwrvss-<br>edegvlidkgKWLLKenhlirrspp<br>.........................<br>.........................<br>.........................<br>.........................<br>.........................	Hs_ENSP00000220676<br>Bt<br>Rn_ENSRNOP00000040735<br>Mm_ENSMUSP00000111202<br>Gg_ENSGALP00000024639<br>Xt<br>Dr<br>Ce<br>Dm<br>Sc	NP_006260 Oxygen-regulated protein 1 (Retinitis pigmentosa RP1 protein)(Retinitis pigmentosa 1 protein)
122	1	CI	ENSP00000355533<br>ENSG00000198626	1385<br>-<br>1370<br>1412<br>1375<br>-<br>-<br>1428<br>1435<br>-	aqekpsrlkqRFLLRr---------<br>.........................<br>aqekpsrlkqRFLLRr---------<br>aqekpsrlkqRFLLRr---------<br>vqekpsrlkqrfmlrr---------<br>.........................<br>.........................<br>hsekpkkggllsrlrdssntrknfr<br>mddkkkrgrspfkffs---------<br>.........................	Hs_ENSP00000355533<br>Bt<br>Rn_ENSRNOP00000023601<br>Mm_ENSMUSP00000021750<br>Gg_ENSGALP00000017561<br>Xt<br>Dr<br>Ce_CE43332<br>Dm_FBpp0087721<br>Sc	Ryanodine receptor 2 (RYR-2)(RyR2)(hRYR-2)(Cardiac muscle-type ryanodine receptor)(Cardiac muscle ryanodine receptor-calcium release channel)
123	1	CI	ENSP00000228468<br>ENSG00000110881	388<br>387<br>419<br>419<br>408<br>393<br>390<br>-<br>-<br>-	mvkipskasaKYLAKkfnkseqyig<br>mvkipskasaKYLAKkfnkseqyig<br>mvkipskasaKYLAKkfnkseqyig<br>mvkipskasaKYLAKkfnkseqyig<br>mvkipskasaKYLAKkynkseqyig<br>mvkipskasaRYLAKkynkseqyia<br>fvkipskasvKYLAKkyskseqyit<br>.........................<br>.........................<br>.........................	Hs_ENSP00000228468<br>Bt_ENSBTAP00000001283<br>Rn_ENSRNOP00000041786<br>Mm_ENSMUSP00000104680<br>Gg_ENSGALP00000040499<br>Xt_ENSXETP00000029317<br>Dr_ENSDARP00000011782<br>Ce<br>Dm<br>Sc	NP_064423 amiloride-sensitive cation channel 2, neuronal isoform a
124	1	CI	ENSP00000262461<br>ENSG00000064651	844<br>836<br>835<br>838<br>728<br>-<br>502<br>795<br>765<br>-	emsidqakyqRWLIKnkmkafyapv<br>emsidqakyqRWLIKnkmkafyapv<br>emsidqakyqRWLIKnkmkafyapv<br>emsidqaryqRWLIKnkmkafyapv<br>elstdlakyqRWLIKnkmkafyapv<br>.........................<br>elnsdmlryqrwllnnnskafytcv<br>vmkrldeninvwlrkrhlkafyrav<br>tylqerag--nwfrkhrvkgfyalv<br>.........................	Hs_ENSP00000262461<br>Bt_ENSBTAP00000012701<br>Rn_ENSRNOP00000021921<br>Mm_ENSMUSP00000111023<br>Gg_ENSGALP00000036922<br>Xt<br>Dr_ENSDARP00000076759<br>Ce_CE40530<br>Dm_FBpp0075719<br>Sc	NP_001037 Solute carrier family 12 member 2 (Bumetanide-sensitive sodium-(potassium)-chloride cotransporter 1)(Basolateral Na-K-Cl symporter)
125	1	CI	ENSP00000307078<br>ENSG00000170759	28<br>28<br>28<br>28<br>28<br>28<br>-<br>-<br>32<br>-	lnesevnrgdKYIAKfqg---edtv<br>lnesevnrgdKYIAKfqg---edtv<br>lnesevnrgdKYVAKfqg---edtv<br>lnesevnrgdKYVAKfqg---edtv<br>lnesevtrgdKYVAKfqg---edtv<br>lndsevtrgdKYVAKfqg---edtv<br>.........................<br>.........................<br>lndseekagsKFVVKfpnnveenci<br>.........................	Hs_ENSP00000307078<br>Bt_ENSBTAP00000033805<br>Rn_ENSRNOP00000023860<br>Mm_ENSMUSP00000025083<br>Gg_ENSGALP00000011666<br>Xt_ENSXETP00000025503<br>Dr<br>Ce<br>Dm_FBpp0086328<br>Sc	NP_004512 Kinesin-1 heavy chain (Ubiquitous kinesin heavy chain)(UKHC)(Conventional kinesin heavy chain)
126	1	CI	ENSP00000386609<br>ENSG00000167716	960<br>749<br>-	akalgpknanKYLLKpligayesp-<br>akalgpknanKYLLKpligayesp-<br>.........................	Hs_ENSP00000386609<br>Bt_ENSBTAP00000015274<br>Rn	WD repeat-containing protein 81

				956<br>-<br>-<br>-<br>729<br>971<br>-	akalgpknanKYLLKpligayesp-<br>.........................<br>.........................<br>.........................<br>trsls-qsaledlinpmieliqcet<br>aqalgvsqtqqhllqpllklydves<br>.........................	Mm_ENSMUSP00000113939<br>Gg<br>Xt<br>Dr<br>Ce_CE42003<br>Dm_FBpp0079896<br>Sc	
127	1	CI	ENSP00000307449<br>ENSG00000103154	167<br>92<br>133<br>170<br>165<br>-<br>91<br>-<br>-<br>-	ggsnvdqfvtRFLLKetanqiqsll<br>ggsnvdqfvtRFLLKetanqiqsll<br>ggnnvdqfvtRFLLKetanqiqsll<br>ggsnvdqfvtRFLLKetanqiqsll<br>sgsnvdqfvtRFLLKetanqtqsll<br>.........................<br>rgtnveqfvtRFLLKesanqiqsll<br>.........................<br>.........................<br>.........................	Hs_ENSP00000307449<br>Bt_ENSBTAP00000045654<br>Rn_ENSRNOP00000020341<br>Mm_ENSMUSP00000095966<br>Gg_ENSGALP00000005234<br>Xt<br>Dr_ENSDARP00000091626<br>Ce<br>Dm<br>Sc	NP_061938 N-terminal EF-hand calcium-binding protein 2 (EF-hand calcium-binding protein 2)(Neuronal calcium-binding protein 2)(Synaptotagmin-interacting protein 2)(Stip-2)
128	1	C	ENSP00000328938<br>ENSG00000183077	31<br>30<br>-<br>29<br>0<br>28<br>0<br>12<br>22<br>-	eelenqycpsRWVVRlgaeealrty<br>eeldnqyspsRWVVRlgaeeamrty<br>.........................<br>eelekqyspsrwvihtkpeevvgnf<br>---------------mdsesviqah<br>eelehqyspsqwsprmdkdavikah<br>---------------msaddvirah<br>nemtllyspscwvpgknrdevmndf<br>dyfpsyhttrfqdqpepnlavlehf<br>.........................	Hs_ENSP00000328938<br>Bt_ENSBTAP00000048024<br>Rn<br>Mm_ENSMUSP00000073102<br>Gg_ENSGALP00000000157<br>Xt_ENSXETP00000006552<br>Dr_ENSDARP00000043266<br>Ce_CE04291<br>Dm_FBpp0078895<br>Sc	Probable arylformamidase (EC 3.5.1.9)(Kynurenine formamidase)(KF)
129	1	CI	ENSP00000352572<br>ENSG00000160299	3210<br>1682<br>2794<br>2791<br>1792<br>-<br>2966<br>-<br>2772<br>-	vrvviailrlRFLVKkwqevdrk--<br>-------------------------<br>vrvviavlrlRFLVKkwqevdrk--<br>vrvviavlrlRFLVKkwqevdrk--<br>vraviavsrlKFLVKkwnklnrkna<br>.........................<br>vrvviaisrlkflnrkwqratrk--<br>.........................<br>alaiiaiqrikyigriwhtgkrivs<br>.........................	Hs_ENSP00000352572<br>Bt_ENSBTAP00000029328<br>Rn_ENSRNOP00000039329<br>Mm_ENSMUSP00000001179<br>Gg_ENSGALP00000011342<br>Xt<br>Dr_ENSDARP00000095611<br>Ce<br>Dm_FBpp0099786<br>Sc	NP_006022 Pericentrin (Pericentrin B)(Kendrin)
130	1	CI	ENSP00000352121<br>ENSG00000105851	472<br>472<br>472<br>472<br>475<br>475<br>467<br>-<br>-<br>-	yyvnlllidhRFLLRrgeyvlhmwq<br>yyvnlllidhRFLLRhgeyvlhmwq<br>yyvnlllidhRFLLRhggyvlhmwq<br>yyvnlllidhRFLLRhgdyvlhmwq<br>yyvnlllidhRFLLRsgeyvlhmwk<br>yyvnlllidhRFLLRqgeyvlhmwk<br>yyvnlllvdhrsllrqgefilhmwk<br>.........................<br>.........................<br>.........................	Hs_ENSP00000352121<br>Bt_ENSBTAP00000027780<br>Rn_ENSRNOP00000047877<br>Mm_ENSMUSP00000020890<br>Gg_ENSGALP00000013101<br>Xt_ENSXETP00000013019<br>Dr_ENSDARP00000002818<br>Ce<br>Dm<br>Sc	NP_002640 Phosphatidylinositol-4,5-bisphosphate 3-kinase catalytic subunit gamma isoform (EC 2.7.1.153)(PI3-kinase p110 subunit gamma)(PtdIns-3-kinase subunit p110)(PI3Kgamma)(PI3K)(p120-PI3K)
130	2	CI	ENSP00000352121<br>ENSG00000105851	679<br>679<br>679<br>679<br>682<br>682<br>674<br>-<br>-<br>-	fepyhdsalaRFLLKrglrnkrigh<br>fepyhdsalaRFLLKrglrnkrigh<br>fepyhdsalaRFLLKrglrnkrigh<br>fepyhdsalaRFLLKrglrnkrigh<br>fepyhdsalaRFLLKrglrnkrigh<br>fepyhdsalsRFLLKralqskrigh<br>fepyhdsalaRFLLKralrskrigh<br>.........................<br>.........................<br>.........................	Hs_ENSP00000352121<br>Bt_ENSBTAP00000027780<br>Rn_ENSRNOP00000047877<br>Mm_ENSMUSP00000020890<br>Gg_ENSGALP00000013101<br>Xt_ENSXETP00000013019<br>Dr_ENSDARP00000002818<br>Ce<br>Dm<br>Sc	NP_002640 Phosphatidylinositol-4,5-bisphosphate 3-kinase catalytic subunit gamma isoform (EC 2.7.1.153)(PI3-kinase p110 subunit gamma)(PtdIns-3-kinase subunit p110)(PI3Kgamma)(PI3K)(p120-PI3K)
131	1	C	ENSP00000335557<br>ENSG00000149346	5<br>0<br>0<br>5<br>6<br>-<br>6<br>-<br>-<br>-	------maskKFAVKcgnfavlvdl<br>-------------------------<br>--------------qcgnfavlvdl<br>------maskKFAVKcgnfavlvdl<br>-----lavkgkysekcgnfavlvdv<br>.........................<br>-----phpslflsqkcgnyavlvdl<br>.........................<br>.........................<br>.........................	Hs_ENSP00000335557<br>Bt_ENSBTAP00000046353<br>Rn_ENSRNOP00000009743<br>Mm_ENSMUSP00000065166<br>Gg_ENSGALP00000014652<br>Xt<br>Dr_ENSDARP00000088058<br>Ce<br>Dm<br>Sc	NP_001009608 UPF0492 protein C20orf94
132	1	CI	ENSP00000373200<br>ENSG00000175161	392<br>-<br>359	vtlcsifllgRYLARhkgtyltnea<br>.........................<br>vtlcsifllgRYLARhkgtyltnea	Hs_ENSP00000373200<br>Bt<br>Rn_ENSRNOP00000040899	NP_694854 Cell adhesion molecule 2 Precursor

				390<br>319<br>368<br>325<br>-<br>-<br>-	vtlcsifllgRYLARhkgtyltnea<br>vtlcsiillgRYLARhkgtyltnea<br>vtlcsiiligRYLARhkgtyltnea<br>atlcliivlgRYLARhkgtyltnea<br>.........................<br>.........................<br>.........................	Mm_ENSMUSP00000113500<br>Gg_ENSGALP00000024947<br>Xt_ENSXETP00000041944<br>Dr_ENSDARP00000056368<br>Ce<br>Dm<br>Sc	(Immunoglobulin superfamily member 4D)(Nectin-like protein 3)
133	1	C	ENSP00000315160<br>ENSG00000149292	580<br>580<br>579<br>579<br>578<br>502<br>584<br>-<br>-<br>-	flktggetasRYAIKilaictnsyh<br>flkaggqtasRYAVKilaictnsyq<br>flktggptasryamktlavctnsyh<br>flrmggqtasRYAIKilaictnsch<br>flkaggqttssyalktlsictrgsr<br>llkaggkrtsvfaakvlavctkvds<br>mlkaggemssrysikaltvctasip<br>.........................<br>.........................<br>.........................	Hs_ENSP00000315160<br>Bt_ENSBTAP00000013201<br>Rn_ENSRNOP00000011454<br>Mm_ENSMUSP00000056378<br>Gg_ENSGALP00000012695<br>Xt_ENSXETP00000025518<br>Dr_ENSDARP00000073892<br>Ce<br>Dm<br>Sc	Tetratricopeptide repeat protein 12 (TPR repeat protein 12)
134	1	CI	ENSP00000205890<br>ENSG00000091536	140<br>135<br>136<br>137<br>0<br>-<br>-<br>-<br>0<br>-	astainwltkKFLLKkaeesgseqa<br>astainwltkKFLIKkaeesgstqa<br>astainwltkKFLLKkaeesgseqa<br>astainwltkKFLLKkaeesgseqa<br>-------------------------<br>.........................<br>.........................<br>.........................<br>-------------------------<br>.........................	Hs_ENSP00000205890<br>Bt_ENSBTAP00000034896<br>Rn_ENSRNOP00000023018<br>Mm_ENSMUSP00000071777<br>Gg_ENSGALP00000007959<br>Xt<br>Dr<br>Ce<br>Dm_FBpp0271775<br>Sc	NP_057323 Myosin-XV (Unconventional myosin-15)
134	2	CI	ENSP00000205890<br>ENSG00000091536	2166<br>2130<br>2151<br>2150<br>952<br>-<br>-<br>-<br>1119<br>-	sgfapspcfnKYLLKfvsdygrngf<br>sgfapsprldKYLLKfvsdhgrggf<br>sgfapsshldKFLLKfvsdygqngf<br>sgfapsphldKFLLKfvsdygqngf<br>safppsaafdKYLLKspcdhsgsaw<br>.........................<br>.........................<br>.........................<br>ccfqpsaafskylmrfvddeapesl<br>.........................	Hs_ENSP00000205890<br>Bt_ENSBTAP00000034896<br>Rn_ENSRNOP00000023018<br>Mm_ENSMUSP00000071777<br>Gg_ENSGALP00000007959<br>Xt<br>Dr<br>Ce<br>Dm_FBpp0271775<br>Sc	NP_057323 Myosin-XV (Unconventional myosin-15)
135	1	CI	ENSP00000294638<br>ENSG00000162624	85<br>104<br>106<br>106<br>76<br>103<br>61<br>-<br>18<br>-	cnscgleivdKYLLKvndlcwhvrc<br>csscgleivdKYLLKvndlcwhvrc<br>csscgleivdKYLLKvndlcwhvrc<br>csscgleivdKYLLKvndlcwhvrc<br>csscgleivdKYLLKvndlcwhvrc<br>csncgleivdKYLLKvndlcwhvrc<br>ctscgteivdKYLLKvndmcwhvrc<br>.........................<br>caacgepisdrfflevggcswhahc<br>.........................	Hs_ENSP00000294638<br>Bt_ENSBTAP00000025544<br>Rn_ENSRNOP00000009370<br>Mm_ENSMUSP00000029852<br>Gg_ENSGALP00000018523<br>Xt_ENSXETP00000006533<br>Dr_ENSDARP00000028001<br>Ce<br>Dm_FBpp0073014<br>Sc	NP_001001933 LIM/homeobox protein Lhx8 (LIM homeobox protein 8)
136	1	CI	ENSP00000263119<br>ENSG00000099991	498<br>497<br>497<br>498<br>497<br>499<br>-<br>-<br>-<br>-	mmrylkamghKFLVRwppglaevvl<br>mmrflksmghRFLLRwppglaevvl<br>mmrylksmghKFLLKwppglaevvl<br>mmrylksmghKFLLKwppgladvvl<br>mmrylkvisqKFLVKwppglsevvl<br>mmrylkviaqKFLVKwppgltdvvl<br>.........................<br>.........................<br>.........................<br>.........................	Hs_ENSP00000263119<br>Bt_ENSBTAP00000018360<br>Rn_ENSRNOP00000001659<br>Mm_ENSMUSP00000001712<br>Gg_ENSGALP00000010272<br>Xt_ENSXETP00000009292<br>Dr<br>Ce<br>Dm<br>Sc	NP_036427 Calcineurin-binding protein cabin-1 (Calcineurin inhibitor)(CAIN)
137	1	CI	ENSP00000305954<br>ENSG00000171658	30<br>30<br>-<br>-<br>-<br>-<br>-<br>-<br>-<br>-	svaraileskKFAVRavtr-----n<br>lcgramlegkKYVVRaltrdvtqpk<br>.........................<br>.........................<br>.........................<br>.........................<br>.........................<br>.........................<br>.........................<br>.........................	Hs_ENSP00000305954<br>Bt_ENSBTAP00000015016<br>Rn<br>Mm<br>Gg<br>Xt<br>Dr<br>Ce<br>Dm<br>Sc	Putative nmrA-like family domain-containing protein ENSP00000305954
138	1	CI	ENSP00000326624<br>ENSG00000118492	196<br>-<br>171	ghmplfnsygKYVVKlywmgcwrki<br>.........................<br>ghmplfnsygKYVVKlywmgcwrki	Hs_ENSP00000326624<br>Bt<br>Rn_ENSRNOP00000019078	NP_078970 Uncharacterized protein C6orf103

				-<br>-<br>-<br>-<br>-<br>-<br>-	.........................<br>.........................<br>.........................<br>.........................<br>.........................<br>.........................<br>.........................	Mm<br>Gg<br>Xt<br>Dr<br>Ce<br>Dm<br>Sc	
139	1	CI	ENSP00000343274<br>ENSG00000164941	148<br>148<br>147<br>148<br>148<br>150<br>148<br>136<br>196<br>-	ttamavllynRWAIRtivqssfpvk<br>ttamaillynRWAIRtivqssfpvk<br>ttamaillynRWAIRtivqssfpvk<br>ttamaillynRWAIRtivqssfpvk<br>ttamaillynRWAIRtivkssfpvk<br>ttvmavtlynRWAIRtivqsgfpvk<br>ttamavvfynRWAIRtivlssfpek<br>wlfyrfvlsidrrnrlappaprati<br>aarfaltiyhrmllrmqlikeqalk<br>.........................	Hs_ENSP00000343274<br>Bt_ENSBTAP00000006451<br>Rn_ENSRNOP00000010789<br>Mm_ENSMUSP00000038418<br>Gg_ENSGALP00000025712<br>Xt_ENSXETP00000014604<br>Dr_ENSDARP00000095264<br>Ce_CE41281<br>Dm_FBpp0086186<br>Sc	NP_060334 Integrator complex subunit 8 (Int8)(Kaonashi protein 1)(Kaonashi-1)
140	1	CI	ENSP00000370288<br>ENSG00000073169	593<br>409<br>593<br>594<br>568<br>429<br>597<br>-<br>-<br>606	hvrvmhannpKYVLRnyiaqnaiea<br>rtrvmrannpKYVLRnyiaqgaiea<br>rvrimhannpKYVLRnyiaqkaiea<br>rvrvmrannpKYVLRnyiaqkaiea<br>rvkvmnsnnpRYILRnyiaqnaiea<br>----mqqvnpaiiprnhrveaalqa<br>rvrvmnnnnphvvlrnyiaqnaiaa<br>.........................<br>.........................<br>raslakkanplfvprswvleevvdd	Hs_ENSP00000370288<br>Bt_ENSBTAP00000000851<br>Rn_ENSRNOP00000007695<br>Mm_ENSMUSP00000081020<br>Gg_ENSGALP00000014135<br>Xt_ENSXETP00000018757<br>Dr_ENSDARP00000090475<br>Ce<br>Dm<br>Sc_YPL222W	NP_113642 Selenoprotein O (SelO)
141	1	CI	ENSP00000343686<br>ENSG00000189195	340<br>352<br>-<br>339<br>324<br>-<br>-<br>-<br>-<br>-	gveslfadcmKWIVKhfarfwsers<br>gveslfadcmKWIVKhfarfwsers<br>.........................<br>gveslfadcmnciishfarfwsers<br>gvehlyaacmkw-------------<br>.........................<br>.........................<br>.........................<br>.........................<br>.........................	Hs_ENSP00000343686<br>Bt_ENSBTAP00000048144<br>Rn<br>Mm_ENSMUSP00000092119<br>Gg_ENSGALP00000009648<br>Xt<br>Dr<br>Ce<br>Dm<br>Sc	NP_899065 BTB/POZ domain-containing protein 8
142	1	CI	ENSP00000264669<br>ENSG00000124279	320<br>320<br>316<br>321<br>224<br>228<br>-<br>-<br>-<br>-	qafpliiklgKYVVRhvphftneel<br>qalplviklsKYVVRhiarftseel<br>harplliklgksvvryiprftneel<br>halplliklgKYVVRyiprftneel<br>qvvplilalckhsvkhvpyftsdel<br>qalplvinlcknsircmphftdeel<br>.........................<br>.........................<br>.........................<br>.........................	Hs_ENSP00000264669<br>Bt_ENSBTAP00000043394<br>Rn_ENSRNOP00000029498<br>Mm_ENSMUSP00000022005<br>Gg_ENSGALP00000021276<br>Xt_ENSXETP00000015438<br>Dr<br>Ce<br>Dm<br>Sc	NP_076996 FAST kinase domain-containing protein 3
143	1	C	ENSP00000254636<br>ENSG00000132305	21<br>21<br>21<br>21<br>21<br>20<br>20<br>21<br>21<br>-	taaaqsclcgKFVLRplrpcrryst<br>tstaqnclcgKFVLRplrpcrryst<br>tvaaqsclcgKFVLRplrpcrryst<br>tvaaqsclcgKFVLRplrpcrryst<br>avaaqncwcrraslrpprlsrgyat<br>rmvgqeclcsrvslrplraarsyst<br>haaarqcvcgkvkahplqhcrrytt<br>sqtlkrarqqsnqaaappkppvqpp<br>kcalqrtlqqttannrqfggsssgs<br>.........................	Hs_ENSP00000254636<br>Bt_ENSBTAP00000004280<br>Rn_ENSRNOP00000054924<br>Mm_ENSMUSP00000109793<br>Gg_ENSGALP00000025404<br>Xt_ENSXETP00000038976<br>Dr_ENSDARP00000074570<br>Ce_CE33696<br>Dm_FBpp0288710<br>Sc	NP_001093639 Mitochondrial inner membrane protein (Mitofilin)(p87/89)(Cell proliferation-inducing gene 4 protein)
144	1	CI	ENSP00000368459<br>ENSG00000083635	475<br>475<br>466<br>464<br>370<br>436<br>-<br>-<br>476<br>-	hernvilqcvRYIIKkdffgldtns<br>hernvilqcvRYIIKkdffglnttt<br>hernvilqcvRYIIKkdffgltags<br>hernvilqcvRYIIKkdffglntds<br>hernvilqcvRYIVRndvfglhska<br>hernvvlqcvryilqndffdkpvdi<br>.........................<br>.........................<br>hernvllqcvrhvcernffgigqak<br>.........................	Hs_ENSP00000368459<br>Bt_ENSBTAP00000012392<br>Rn_ENSRNOP00000001366<br>Mm_ENSMUSP00000022586<br>Gg_ENSGALP00000027372<br>Xt_ENSXETP00000032368<br>Dr<br>Ce<br>Dm_FBpp0074855<br>Sc	NP_036477 Nuclear fragile X mental retardation-interacting protein 1 (Nuclear FMRP-interacting protein 1)
145	1	CI	ENSP00000381707<br>ENSG00000167210	718<br>721<br>717	vieqhkfeahRWLARgkednelvve<br>viqeykfdahrwlaqgkednelvve<br>vivqykfvanRWLARgkednelvve	Hs_ENSP00000381707<br>Bt_ENSBTAP00000020236<br>Rn_ENSRNOP00000055374	Lipoxygenase homology domain-containing protein 1

				716<br>493<br>-<br>-<br>-<br>-<br>-	vivqykfdvnRWLARgkednelvve<br>-------------cniqsemrmrie<br>.........................<br>.........................<br>.........................<br>.........................<br>.........................	Mm_ENSMUSP00000094294<br>Gg_ENSGALP00000002753<br>Xt<br>Dr<br>Ce<br>Dm<br>Sc	
145	2	CI	ENSP00000381707<br>ENSG00000167210	999<br>1003<br>999<br>998<br>744<br>-<br>-<br>-<br>-<br>-	lgkcmtfpcgRWLAKneddgsiird<br>lgkcmtfpcgRWLAKneddgtivrd<br>lgkcmtfpcgRWLAKneddgsivrd<br>lgkcmtfpcgRWLAKneddgsivrd<br>nqevatftygdwlskvknakgslvc<br>.........................<br>.........................<br>.........................<br>.........................<br>.........................	Hs_ENSP00000381707<br>Bt_ENSBTAP00000020236<br>Rn_ENSRNOP00000055374<br>Mm_ENSMUSP00000094294<br>Gg_ENSGALP00000002753<br>Xt<br>Dr<br>Ce<br>Dm<br>Sc	Lipoxygenase homology domain-containing protein 1
146	1	CI	ENSP00000354778<br>ENSG00000174469	462<br>-<br>430<br>462<br>13<br>-<br>-<br>-<br>462<br>-	glndgqwhevRFLAKenfailtidg<br>.........................<br>glndgqwhevRFLAKenfavltidg<br>glndgqwhevRFLAKenfavltidg<br>glndgqwhevRFLAKenfavltidg<br>.........................<br>.........................<br>.........................<br>qfndgkwhsfvisieknrlilnid-<br>.........................	Hs_ENSP00000354778<br>Bt<br>Rn_ENSRNOP00000008688<br>Mm_ENSMUSP00000110288<br>Gg_ENSGALP00000020203<br>Xt<br>Dr<br>Ce<br>Dm_FBpp0075730<br>Sc	NP_054860 Contactin-associated protein-like 2 Precursor (Cell recognition molecule Caspr2)
147	1	CI	ENSP00000358879<br>ENSG00000196924	2387<br>2390<br>2387<br>2387<br>-<br>-<br>-<br>2353<br>2142<br>-	ecyvteidqdKYAVRfiprengvyl<br>ecyvteidqdKYAVRfiprengiyl<br>ecyvteidqdKYAVRfiprengvyl<br>ecyvteidqdKYAVRfiprengiyl<br>.........................<br>.........................<br>.........................<br>gpvvedmgqgkyrvrfnpdqpgkys<br>dcfiqvidgemysvrfyprengiha<br>.........................	Hs_ENSP00000358879<br>Bt_ENSBTAP00000030179<br>Rn_ENSRNOP00000008910<br>Mm_ENSMUSP00000033699<br>Gg<br>Xt<br>Dr<br>Ce_CE42067<br>Dm_FBpp0288453<br>Sc	NP_001104026 Filamin-A (Alpha-filamin)(Filamin-1)(Endothelial actin-binding protein)(Actin-binding protein 280)(ABP-280)(Non-muscle filamin)
148	1	CI	ENSP00000265727<br>ENSG00000008277	213<br>84<br>-<br>211<br>105<br>105<br>85<br>-<br>-<br>-	efqqvnitpsKFILKprpkrskrql<br>efqqvnitppKFILKprpkrskrql<br>.........................<br>efqrvnitppqfilkprlkrrkrql<br>efwhmnatsqKFVVKprhkrrrrqv<br>efwplndtsprlsqqtrnqrkkrqt<br>vhlaeslfenpplfsaahrrkkrqi<br>.........................<br>.........................<br>.........................	Hs_ENSP00000265727<br>Bt_ENSBTAP00000009397<br>Rn<br>Mm_ENSMUSP00000086140<br>Gg_ENSGALP00000014588<br>Xt_ENSXETP00000016173<br>Dr_ENSDARP00000079811<br>Ce<br>Dm<br>Sc	NP_068369 ADAM 22 Precursor (A disintegrin and metalloproteinase domain 22)(Metalloproteinase-like, disintegrin-like, and cysteine-rich protein 2)(Metalloproteinase-disintegrin ADAM22-3)
149	1	CI	ENSP00000344789<br>ENSG00000132142	754<br>680<br>679<br>679<br>680<br>685<br>663<br>615<br>906<br>-	vdveliyegvKYVLKvtrqspnsyv<br>vdveliyegvKYVLKvtrqspnsyv<br>vdveliyegiKYVLKvtrqspnsyv<br>vdveliyegiKYVLKvtrqspnsyv<br>vdveliyegrKYVLKvtrqspnsyv<br>vdveliyegeKYVLKvtrqspnsyv<br>vdveliyegtKYVLKvtrqspnsyv<br>fifdlvkdlniyslkvvrsaentfv<br>vdvelindgirykvqaaksgansyf<br>.........................	Hs_ENSP00000344789<br>Bt_ENSBTAP00000023364<br>Rn_ENSRNOP00000043972<br>Mm_ENSMUSP00000099490<br>Gg_ENSGALP00000008719<br>Xt_ENSXETP00000019619<br>Dr_ENSDARP00000077514<br>Ce_CE14734<br>Dm_FBpp0111967<br>Sc	NP_942133 Acetyl-CoA carboxylase 1 (EC 6.4.1.2)(ACC-alpha)
150	1	CI	ENSP00000348849<br>ENSG00000197728	38<br>38<br>38<br>38<br>-<br>37<br>37<br>-<br>38<br>38	rcvpkdkaikKFVIRniveaaavrd<br>rcvpkdkaikKFVIRniveaaavrd<br>rcvpkdkaikKFVIRniveaaavrd<br>rcvpkdkaikKFVIRniveaaavrd<br>.........................<br>rcvpkdkaikKFVIRniveaaavrd<br>rcvpkdkaikKFVIRniveaaavrd<br>.........................<br>rcvpkdkaikKFVIRniveaaavrd<br>ksipkdkaikrmairniveaaavrd	Hs_ENSP00000348849<br>Bt_ENSBTAP00000051979<br>Rn_ENSRNOP00000007304<br>Mm_ENSMUSP00000026420<br>Gg<br>Xt_ENSXETP00000020851<br>Dr_ENSDARP00000043988<br>Ce<br>Dm_FBpp0080639<br>Sc_YER131W	NP_001087200 40S ribosomal protein S26
151	1	CI	ENSP00000330694<br>ENSG00000185115	265<br>236<br>317	nletskmkvlKFVAKvhnqdpkdwp<br>nletskmkvlKFVAKihhqdpkdwp<br>nletskmkvlKFVAKvhnqdpkdwp	Hs_ENSP00000330694<br>Bt_ENSBTAP00000050241<br>Rn_ENSRNOP00000022069	NP_619649 Melanoma-associated antigen G1 (MAGE-G1 antigen)(Necdin-like protein 2)

				239<br>-<br>206<br>230<br>-<br>-<br>-	nletskmkvlKFVAKvhnqdpkdwp<br>.........................<br>fketskmqvlefvskiqqkdpkswt<br>ekevsklkllefvgelfdqdpqnwt<br>.........................<br>.........................<br>.........................	Mm_ENSMUSP00000091889<br>Gg<br>Xt_ENSXETP00000047694<br>Dr_ENSDARP00000075481<br>Ce<br>Dm<br>Sc	(Hepatocellular carcinoma-associated protein 4)
152	1	CI	ENSP00000359520<br>ENSG00000198018	64<br>-<br>63<br>64<br>-<br>64<br>-<br>-<br>-<br>0	aslprdrqyeRYLARvgeleatdte<br>.........................<br>tslprdrqyeRYLARvgdleatnte<br>tslprdrqyeRYLARvgdleatnte<br>.........................<br>srafrdrqfrryldqiedleatdtk<br>.........................<br>.........................<br>.........................<br>---------------------mlie	Hs_ENSP00000359520<br>Bt<br>Rn_ENSRNOP00000023111<br>Mm_ENSMUSP00000079864<br>Gg<br>Xt_ENSXETP00000008111<br>Dr<br>Ce<br>Dm<br>Sc_YER005W	NP_065087 Ectonucleoside triphosphate diphosphohydrolase 7 (NTPDase 7)(EC 3.6.1.-) (Lysosomal apyrase-like protein 1)
153	1	CI	ENSP00000359153<br>ENSG00000111799	1595<br>1594<br>1595<br>1595<br>1595<br>1603<br>987<br>-<br>-<br>-	fwepvpgkvrKYIVRyktpee-dvk<br>awepapgkvrKYIVRyktpee-eak<br>iwepvlgkvrkytvryktpee-efk<br>vwepvlgkvrKYIVRyktpde-efk<br>lwdpapgkvrKYIIRykiadeadvk<br>lwsaapgnvlKYIVRykteeeeefk<br>twvpgavdvpahriiystnhgsdvk<br>.........................<br>.........................<br>.........................	Hs_ENSP00000359153<br>Bt_ENSBTAP00000026725<br>Rn_ENSRNOP00000051534<br>Mm_ENSMUSP00000080194<br>Gg_ENSGALP00000036300<br>Xt_ENSXETP00000007774<br>Dr_ENSDARP00000054640<br>Ce<br>Dm<br>Sc	NP_004361 Collagen alpha-1(XII) chain Precursor
154	1	CI	ENSP00000259469<br>ENSG00000136942	118<br>118<br>117<br>118<br>122<br>117<br>118<br>118<br>118<br>115	qrkerlyplrKYAVKa---------<br>qrkerlyplrKYAVKa---------<br>qrkerlyplrKYAVKa---------<br>qrkerlyplrKYAVKa---------<br>qrkerlypvrKYAIKa---------<br>qrkdrlfsarKFAVKa---------<br>qrksrlysirKFAVKa---------<br>qaksrnqavrKFAVKa---------<br>irkrsvfpqrKFAVKa---------<br>rkkqiafpqrKYAIKa---------	Hs_ENSP00000259469<br>Bt_ENSBTAP00000004161<br>Rn_ENSRNOP00000019162<br>Mm_ENSMUSP00000099843<br>Gg_ENSGALP00000037906<br>Xt_ENSXETP00000036714<br>Dr_ENSDARP00000018594<br>Ce_CE00450<br>Dm_FBpp0070766<br>Sc_YDL191W	NP_009140 60S ribosomal protein L35
155	1	CI	ENSP00000359119<br>ENSG00000184343	173<br>172<br>172<br>172<br>-<br>-<br>151<br>-<br>-<br>-	vlevlghqllKWIIKsnyqglpvpc<br>vlevlghqllKWIIKsnyqglpvpc<br>vlevlghqllKWIIKsnyqglpvpc<br>vlevlghqllKWIIKsnyqglpvpc<br>.........................<br>.........................<br>vlevlghqllKWIIKsnymglplic<br>.........................<br>.........................<br>.........................	Hs_ENSP00000359119<br>Bt_ENSBTAP00000001404<br>Rn_ENSRNOP00000026211<br>Mm_ENSMUSP00000002081<br>Gg<br>Xt<br>Dr_ENSDARP00000016135<br>Ce<br>Dm<br>Sc	NP_055185 Serine/threonine-protein kinase SRPK3 (EC 2.7.11.1)(Serine/arginine-rich protein specific kinase 3) (Serine/threonine-protein kinase 23)(Muscle-specific serine kinase 1)(MSSK-1)
156	1	CI	ENSP00000362861<br>ENSG00000106852	109<br>109<br>80<br>109<br>80<br>56<br>107<br>108<br>-<br>-	csscgleildRYLLKvnnliwh--v<br>csscgleildRYLLKvnnliwh--v<br>csscgleildRYLLKvnnliwh--v<br>csscgleildRYLLKvnnliwh--v<br>csscgleildRYLLKsnnciwhsgl<br>csscgleildRYLLKvnnliwh--v<br>cascgleildRYLLKvnnliwh--v<br>ctqcqhqiqdkfflsidgrnyh--e<br>.........................<br>.........................	Hs_ENSP00000362861<br>Bt_ENSBTAP00000007266<br>Rn_ENSRNOP00000008817<br>Mm_ENSMUSP00000108587<br>Gg_ENSGALP00000002055<br>Xt_ENSXETP00000035623<br>Dr_ENSDARP00000017657<br>Ce_CE25675<br>Dm<br>Sc	NP_055183 LIM/homeobox protein Lhx6.1 (LIM homeobox protein 6.1)(Lhx6)
157	1	CI	ENSP00000200135<br>ENSG00000086827	250<br>250<br>249<br>250<br>249<br>247<br>251<br>235<br>253<br>-	klksfgqmllKYILRplascpslha<br>klksfgqmllKYILKplascpslla<br>klksfgqmllKYILKplvtcpslha<br>klksfgqmllKYILKplvtcpslha<br>klktfgklllKYILKplisypflqp<br>klkffsqlllnyilkplvtypslha<br>kiklfgqvlfKYILKplilypslvv<br>rldawkivilnvfceaivasrdgvd<br>rlcef---lldnciepvimrpvmad<br>.........................	Hs_ENSP00000200135<br>Bt_ENSBTAP00000026733<br>Rn_ENSRNOP00000010595<br>Mm_ENSMUSP00000034803<br>Gg_ENSGALP00000011993<br>Xt_ENSXETP00000049699<br>Dr_ENSDARP00000020574<br>Ce_CE04434<br>Dm_FBpp0070425<br>Sc	NP_004715 Centromere/kinetochore protein zw10 homolog
158	1	CI	ENSP00000304743<br>ENSG00000163539	436<br>209<br>208	vmatsgcaaiRFIIRhthvprlipl<br>vmatsgcaaiRFIIRhthvprlipl<br>vmatsgcaaiRFIIRhthvprlipl	Hs_ENSP00000304743<br>Bt_ENSBTAP00000015071<br>Rn_ENSRNOP00000012545	CLIP-associating protein 2 (Cytoplasmic linker-associated protein 2)(hOrbit2)

				208	vmatsgcaaiRFIIRhthvprlipl	Mm_ENSMUSP00000107469	
				432	vmatsgcaaiRFIIRhthvprlipl	Gg_ENSGALP00000019543	
				-	....	Xt	
				435	vmatsgtaairiiirhthvprlipl	Dr_ENSDARP00000039870	
				-	....	Ce	
				-	....	Dm	
				-	....	Sc	
159	1	CI	ENSP00000356155<br>ENSG00000133056	938	yecyldsplvRFLLKravsdlrvth	Hs_ENSP00000356155	NP_002637 Phosphatidylinositol-4-phosphate 3-kinase C2 domain-containing beta polypeptide (EC 2.7.1.154)(Phosphoinositide 3-Kinase-C2-beta)(PtdIns-3-kinase C2 beta)(PI3K-C2beta)(C2-PI3K)
				635	yecyldsplvRFLLKravsdlrvth	Bt_ENSBTAP00000017388	
				948	yecyldsplvRFLLKraisdlrvth	Rn_ENSRNOP00000051488	
				937	yecyldsplvRFLLKraisdlrvth	Mm_ENSMUSP00000076911	
				645	yecyldsplvrflmkraicdlkith	Gg_ENSGALP00000000882	
				-	....	Xt	
				664	yecyldshlvRFLLRraigdvriah	Dr_ENSDARP00000080539	
				-	....	Ce	
				1163	hdtyegsamarfllskclesprfah	Dm_FBpp0075818	
				-	....	Sc	
160	1	C	ENSP00000273406<br>ENSG00000144867	265	vgsadiqdleKWLAKia--------	Hs_ENSP00000273406	NP_067026 Signal recognition particle receptor subunit beta (SR-beta)(Protein APMCF1)
				-	....	Bt	
				-	....	Rn	
				263	tgsadiqdleKWLAKia--------	Mm_ENSMUSP00000035157	
				-	....	Gg	
				258	dgdanisnvegwlaklv--------	Xt_ENSXETP00000025837	
				261	dgdadidalekclakl---------	Dr_ENSDARP00000047012	
				236	--dfgvheiasfvra----------	Ce_CE12874	
				238	--dteldpltdwlarll--------	Dm_FBpp0076325	
				238	---rkisqwrewidekl--------	Sc_YKL154W	
161	1	CI	ENSP00000384675<br>ENSG00000115904	914	ahelsedhykKYLAKlrsinppcvp	Hs_ENSP00000384675	NP_005624 Son of sevenless homolog 1 (SOS-1)
				914	ahelsedhykKYLAKlrsinppcvp	Bt_ENSBTAP00000015471	
				936	ahelsedhykKYLAKlrsinppcvp	Rn_ENSRNOP00000009359	
				914	ahelsedhykKYLAKlrsinppcvp	Mm_ENSMUSP00000067786	
				914	ayelsedhykKYLAKlrsinppcvp	Gg_ENSGALP00000021567	
				914	ahelsedhqkKYLAKlrsinppcvp	Xt_ENSXETP00000018371	
				-	....	Dr	
				1031	fntlcerrwqemqkrlssinppcip	Ce_CE36328	
				-	....	Dm	
				-	....	Sc	
162	1	CI	ENSP00000305027<br>ENSG00000168502	747	qniflfyvklRWLLKhwr---qgkq	Hs_ENSP00000305027	Uncharacterized protein KIAA0802
				648	qniflfyvklRWLLKhwr---qgrq	Bt_ENSBTAP00000012453	
				1033	qniflfyvklRWLLKhwr---qgkq	Rn_ENSRNOP00000042113	
				1036	qniflfyvklRWLLKhwr---qgkq	Mm_ENSMUSP00000083899	
				730	qdtflvyvklKWLLKhwr---qgkr	Gg_ENSGALP00000021759	
				606	envfviyaklkrllkhwq---qckg	Xt_ENSXETP00000009342	
				85	eslllislklRWLLKqwr---lgkk	Dr_ENSDARP00000086285	
				742	qemknvhqkldkiknvtpntglsvk	Ce_CE43250	
				-	....	Dm	
				-	....	Sc	
163	1	CI	ENSP00000247986<br>ENSG00000117245	132	fesvqcaentKFLVRasyleiyned	Hs_ENSP00000247986	NP_065867 Kinesin-like protein KIF17 (KIF3-related motor protein)
				132	fesvqcaentKFLVRasyleiyned	Bt_ENSBTAP00000023470	
				132	fesvqcaentKFLVRasyleiyned	Rn_ENSRNOP00000020215	
				132	fesvqcaentKFLVRasyleiyned	Mm_ENSMUSP00000030539	
				-	....	Gg	
				-	....	Xt	
				-	....	Dr	
				128	ftatattenvkflvhcsyleiynee	Ce_CE31568	
				129	ferismttnvrylalvtyleiyner	Dm_FBpp0088260	
				-	....	Sc	
164	1	CI	ENSP00000347672<br>ENSG00000173218	513	seefidpkshKFVLRlqsetsv---	Hs_ENSP00000347672	NP_620409 Vang-like protein 1 (Van Gogh-like protein 1)(Strabismus 2)(Loop-tail protein 2 homolog)(LPP2)
				513	seefidpkshKFVLRlqsetsv---	Bt_ENSBTAP00000007608	
				515	seefidpkshKFVLRlqsetsv---	Rn_ENSRNOP00000022182	
				515	seefidpkshKFVLRlqsetsv---	Mm_ENSMUSP00000029453	
				514	seefidpkshKFVLRlqsetsv---	Gg_ENSGALP00000024186	
				-	....	Xt	
				516	seeyvhpkshkfvlqlqsetsv---	Dr_ENSDARP00000091694	
				521	teqak-pgnekfslkisnesav---	Ce_CE06708	
				570	aeevvdpksnKFVLKlnsetsv---	Dm_FBpp0087647	
				-	....	Sc	
165	1	CI	ENSP00000357669<br>ENSG00000169418	671	lkssncvvdgRFVLKitdyglesfr	Hs_ENSP00000357669	NP_000897 Atrial natriuretic peptide receptor A Precursor (EC 4.6.1.2)(Atrial natriuretic peptide
				668	lkssncvvdgRFVLKitdyglesfr	Bt_ENSBTAP00000008184	
				667	lkssncvvdgRFVLKitdyglesfr	Rn_ENSRNOP00000020307	

				667<br>-<br>431<br>678<br>865<br>731<br>-	lkssncvvdgRFVLKitdyglesfr<br>.........................<br>lkssncvvdsRFVLKitdyglasfr<br>lkssncvvdnRFVLKitdyglssir<br>lkssncvvdsRFVLKvtdfglhrlh<br>lkssncvvdsRFVLKitdfglhtlr<br>.........................	Mm_ENSMUSP00000029540<br>Gg<br>Xt_ENSXETP00000031275<br>Dr_ENSDARP00000010052<br>Ce_CE33583<br>Dm_FBpp0082641<br>Sc	A-type receptor)(ANPRA)(ANP-A)(NPR-A)(Guanylate cyclase)(GC-A)
166	1	CI	ENSP00000364240<br>ENSG00000162543	173<br>104<br>175<br>170<br>173<br>-<br>104<br>-<br>-<br>-	sraracaverKFIVRtkkqgssrag<br>--------errvlvrterqtssrvg<br>srtqvcalekKFIIRtkrqsssras<br>srtqvcalekKFIIRtkrqsssras<br>vpneealcapeklgrkmrqegplls<br>.........................<br>------kcsqcvsrhrkcqhtnarl<br>.........................<br>.........................<br>.........................	Hs_ENSP00000364240<br>Bt_ENSBTAP00000052187<br>Rn_ENSRNOP00000000167<br>Mm_ENSMUSP00000101437<br>Gg_ENSGALP00000038843<br>Xt<br>Dr_ENSDARP00000068895<br>Ce<br>Dm<br>Sc	NP_689589 UBX domain-containing protein 10 (UBX domain-containing protein 3)
167	1	CI	ENSP00000381630<br>ENSG00000214597	183<br>183<br>-<br>-<br>-<br>-<br>-<br>-<br>-<br>-	ehyeqmeylgRYIARklninyfdyl<br>eryeqmeylgRYIARklninyfdcl<br>.........................<br>.........................<br>.........................<br>.........................<br>.........................<br>.........................<br>.........................<br>.........................	Hs_ENSP00000381630<br>Bt_ENSBTAP00000046511<br>Rn<br>Mm<br>Gg<br>Xt<br>Dr<br>Ce<br>Dm<br>Sc	Putative transmembrane protein C8orfK29
168	1	CI	ENSP00000354251<br>ENSG00000061676	64<br>22<br>58<br>64<br>58<br>58<br>64<br>56<br>57<br>-	lidknlesavKFIVRkfpavetrnn<br>lidknlesavKFIVRkfpavetrnn<br>lidknlesavKFIVRkfpavetrnn<br>lidknlesavKFIVRkfpavetrnn<br>lidknlesavKFIVRkfpavetrnn<br>lidknlesavKFIVRkfpavetrnn<br>lidknlesavKFIVRkfpavetrnn<br>lsdkkmegaikhivrkfpvvdcrsn<br>lsekslessiKFIVKrfpnidvkg-<br>.........................	Hs_ENSP00000354251<br>Bt_ENSBTAP00000044947<br>Rn_ENSRNOP00000010177<br>Mm_ENSMUSP00000107390<br>Gg_ENSGALP00000004335<br>Xt_ENSXETP00000002843<br>Dr_ENSDARP00000080601<br>Ce_CE05750<br>Dm_FBpp0078162<br>Sc	NP_995314 Nck-associated protein 1 (NAP 1)(p125Nap1)(Membrane-associated protein HEM-2)
169	1	CI	ENSP00000202788<br>ENSG00000089022	41<br>37<br>47<br>47<br>50<br>44<br>47<br>-<br>-<br>-	-vcvkkstqeRFALK-illdrpkar<br>-vcvkkstqeRFALK-ilfdrpkar<br>-vcvkkstqeRFALK-illdrpkar<br>-vcvkkstqeRFALK-illdrpkar<br>rvcvkkssqeRFALK-illdrpkar<br>----lvtfmpqfqlkyvlldypkn-<br>-vcvkkstqeRFALK-ilidrpkar<br>.........................<br>.........................<br>.........................	Hs_ENSP00000202788<br>Bt_ENSBTAP00000012303<br>Rn_ENSRNOP00000001817<br>Mm_ENSMUSP00000031410<br>Gg_ENSGALP00000007550<br>Xt_ENSXETP00000025545<br>Dr_ENSDARP00000035123<br>Ce<br>Dm<br>Sc	NP_620777 MAP kinase-activated protein kinase 5 (MAPK-activated protein kinase 5)(MAPKAP kinase 5)(EC 2.7.11.1)(p38-regulated/activated protein kinase)
170	1	CI	ENSP00000354255<br>ENSG00000109625	647<br>639<br>647<br>649<br>638<br>539<br>455<br>-<br>-<br>-	yftslsthrpRWLLKy---------<br>yftslgqhqpRWLLKy---------<br>yftslsphkpRWLLKy---------<br>yftslspykpRWLLKy---------<br>yfssldlhkplwllkqh--------<br>-------------------------<br>tvmeklnf-----------------<br>.........................<br>.........................<br>.........................	Hs_ENSP00000354255<br>Bt_ENSBTAP00000019695<br>Rn_ENSRNOP00000012109<br>Mm_ENSMUSP00000039804<br>Gg_ENSGALP00000025103<br>Xt_ENSXETP00000033656<br>Dr_ENSDARP00000069307<br>Ce<br>Dm<br>Sc	NP_001014447 Carboxypeptidase Z Precursor (CPZ)(EC 3.4.17.-)
171	1	CI	ENSP00000323184<br>ENSG00000136449	92<br>92<br>-<br>76<br>4<br>62<br>-<br>-<br>-<br>-	ekedkrvitqKFIIRklkpmdprrk<br>etedkhvitqRFIIRkpkpkd-hrk<br>.........................<br>etgekpvvtqKFIIRklkpkdsskr<br>-------vrrKFLVRkcqpretrsa<br>keeqniagtnhvlvrkhnaeg--rk<br>.........................<br>.........................<br>.........................<br>.........................	Hs_ENSP00000323184<br>Bt_ENSBTAP00000019089<br>Rn<br>Mm_ENSMUSP00000091477<br>Gg_ENSGALP00000012396<br>Xt_ENSXETP00000022000<br>Dr<br>Ce<br>Dm<br>Sc	NP_115509 MYCBP-associated protein (AMY-1-binding protein 1)(AMAP-1)(AMAM-1)
172	1	CI	ENSP00000323555<br>ENSG00000181689	297<br>298<br>298	liyslrnkdvKYALRrtwnnlcnif<br>liyslrnkdvkcalhrvwkkscnlf<br>liyslrnkdvKFALKrtlnnlcklf	Hs_ENSP00000323555<br>Bt_ENSBTAP00000004342<br>Rn_ENSRNOP00000039249	NP_001005202 Olfactory receptor 8K3 (Olfactory receptor OR11-181)

				298	liyslrnkdvKYALKrtlnnlcklf	Mm_ENSMUSP00000097485	
				-	....	Gg	
				-	....	Xt	
				-	....	Dr	
				-	....	Ce	
				-	....	Dm	
				-	....	Sc	
173	1	CI	ENSP00000265245 ENSG00000041802	533	hgqpdqprsaRYILKdyvsgkllyc	Hs_ENSP00000265245	NP_060855 Large subunit GTPase 1 homolog (hLsg1)(EC 3.6.1.-)
				528	hgqpdqprsaRYILKdyvngkllyc	Bt_ENSBTAP00000011134	
				531	hgqpdqprsaRYILKdyvrgkllyc	Rn_ENSRNOP00000002354	
				519	hgqpdqprsaRYILKdyvggkllyc	Mm_ENSMUSP00000112860	
				529	hgqpdqprsaRYVLKdyvsgkllyc	Gg_ENSGALP00000011420	
				468	hgqpdqprsaRYVLKdyvsgkllyc	Xt_ENSXETP00000007467	
				518	hgqpdqsrsaRYVLKdyvsgkllyc	Dr_ENSDARP00000012550	
				-	....	Ce	
				484	ngqpdqarsaRYVLKdyvngrllya	Dm_FBpp0070284	
				-	....	Sc	
174	1	CI	ENSP00000365711 ENSG00000165309	51	ilakaceaiyKFALKgeenkttlle	Hs_ENSP00000365711	Armadillo repeat-containing protein 3 (Beta-catenin-like protein)(KU-CT-1)(Cancer/testis antigen 81)(CT81)
				51	ilakaceaiyRFALKgeenkatlle	Bt_ENSBTAP00000044562	
				51	ilakaceaiyKFALKgeenkatlle	Rn_ENSRNOP00000022475	
				51	ilakaceaiyKFALKgeenkatlle	Mm_ENSMUSP00000110287	
				51	vlakacdaiykfaskgdenkvallg	Gg_ENSGALP00000012748	
				51	ilakscdalykfakkgeenklalle	Xt_ENSXETP00000013016	
				-	....	Dr	
				-	....	Ce	
				60	vlltifshitdfarrqdvnvlelka	Dm_FBpp0086892	
				-	....	Sc	
175	1	CI	ENSP00000361287 ENSG00000151224	299	kvdrsaayaaRWVAKslvkaglcrr	Hs_ENSP00000361287	NP_000420 S-adenosylmethionine synthetase isoform type-1 (AdoMet synthetase 1)(EC 2.5.1.6)(Methionine adenosyltransferase 1)(Methionine adenosyltransferase I/III)(MAT-I/III)
				300	kvdrsaayaaRWVAKslvkaglcrr	Bt_ENSBTAP00000031255	
				-	....	Rn	
				300	kvdrsaayaaRWVAKslvkaglcrr	Mm_ENSMUSP00000044288	
				300	kvdrsaayaaRWVAKslvkaglcrr	Gg_ENSGALP00000003899	
				288	kvdrsaayaaRWVAKslvhaklchr	Xt_ENSXETP00000007635	
				294	kvdrsaayaaRWVAKslvksklcrr	Dr_ENSDARP00000057868	
				-	....	Ce	
				-	....	Dm	
				286	kvdrsaayaaRWVAKslvaaglckr	Sc_YLR180W	
176	1	CI	ENSP00000365757 ENSG00000150867	141	garfhtsydkRYIIKtitsedvaem	Hs_ENSP00000365757	NP_005019 Phosphatidylinositol-5-phosphate 4-kinase type-2 alpha (EC 2.7.1.149)(Phosphatidylinositol-5-phosphate 4-kinase type II alpha)(1-phosphatidylinositol-5-phosphate 4-kinase 2-alpha)(PtdIns(5)P-4-kinase isoform 2-alpha)(PIP4KII-alpha)(Diphosphoinositide kinase 2-alpha)(PtdIns(4)P-5-kinase B isoform)(PIP5KIII)(PtdIns(4)P-5-kinase C isoform)
				93	garfhtsydkRYIIKtitsedvaem	Bt_ENSBTAP00000024007	
				-	....	Rn	
				141	garfhtsydkRYVIKtitsedvaem	Mm_ENSMUSP00000006912	
				93	garfhtsydkRYIIKtitsedvaem	Gg_ENSGALP00000012779	
				-	....	Xt	
				93	garfhtsydkRYVIKtissedvaem	Dr_ENSDARP00000003352	
				138	tprffisydkKFVIKsmdseavael	Ce_CE27279	
				-	....	Dm	
				-	....	Sc	
177	1	CI	ENSP00000369409 ENSG00000205436	575	raqetlqevhRFVVReylaralrpr	Hs_ENSP00000369409	NP_001071062 SEC6-like protein C14orf73
				582	laqetlqevhRYVVReyleqalrpr	Bt_ENSBTAP00000038287	
				577	laqdtlqevhRFVVReylgqvlrph	Rn_ENSRNOP00000013460	
				577	laqetlqevhRFVVReylgqvlrph	Mm_ENSMUSP00000021709	
				449	lgqellchvhKYVVReyivqiikpk	Gg_ENSGALP00000018636	
				-	....	Xt	
				436	yrqafvnevhffvvkeyvsqlmknn	Dr_ENSDARP00000075431	
				-	....	Ce	
				-	....	Dm	
				-	....	Sc	
178	1	CI	ENSP00000355315 ENSG00000198918	11	msshktfrikRFLAKkqkqnrpipq	Hs_ENSP00000355315	NP_000991 60S ribosomal protein L39
				11	msshktfrikRFLAKkqkqnrpipq	Bt_ENSBTAP00000040870	
				11	msshktfrikRFLAKkqkqnrpipq	Rn_ENSRNOP00000045098	
				11	msshktfrikRFLAKkqkqnrpipq	Mm_ENSMUSP00000110886	
				11	msshktfkikRFLAKkqkqnrpipq	Gg_ENSGALP00000038282	
				-	....	Xt	
				10	-ashktfrikRFLAKkqkqnrpipq	Dr_ENSDARP00000052760	
				11	msalkksfikrklakkqkqnrpmpq	Ce_CE06883	

				-	....	Dm	
				11	maaqksfrikqkmakakkqnrplpq	Sc_YJL189W	
179	1	CI	ENSP00000359417 ENSG00000063601	480	vekewisfghRFALRvghgndnhad	Hs_ENSP00000359417	NP_003819 Myotubularin-related protein 1 (EC 3.1.3.-)
				500	iekewisfghRFALRvghgndnhad	Bt_ENSBTAP00000012726	
				479	iekewisfghRFALRvghgddnhad	Rn_ENSRNOP00000003638	
				484	iekewisfghRFALRvghgddnhad	Mm_ENSMUSP00000110248	
				487	iekewisfghrfamrvghggddhad	Gg_ENSGALP00000014766	
				497	lekewisfghrfamrvghgddnhad	Xt_ENSXETP00000016892	
				497	lekewisfghkfaarighgdenhan	Dr_ENSDARP00000073674	
				-	....	Ce	
				-	....	Dm	
				-	....	Sc	
180	1	CI	ENSP00000335371 ENSG00000168538	670	vpgktrkllfKFVAKtedvgkkiei	Hs_ENSP00000335371	NP_068761 UPF0636 protein C4orf41
				670	vpgktrkflfKFVAKtedvgkkiei	Bt_ENSBTAP00000009769	
				670	vpgktrkllfKFVAKtedvgkkiei	Rn_ENSRNOP00000018969	
				670	vpgktrklsfKFVAKtedvgkkiei	Mm_ENSMUSP00000047562	
				670	vpgktrkftfKFVAKtedvgkkiei	Gg_ENSGALP00000017312	
				613	-------------------------	Xt_ENSXETP00000041337	
				670	vpgktrkycfnfvaktedvgkkiei	Dr_ENSDARP00000097453	
				681	skskplqrvvlldlknakqnwivtv	Ce_CE03884	
				701	epgsyyqlfcsteaqqfhentqlri	Dm_FBpp0072905	
				-	....	Sc	
181	1	CI	ENSP00000310565 ENSG00000171451	359	gpesqlvfldKFILKngagnwlaqq	Hs_ENSP00000310565	NP_115536 Dermatan-sulfate epimerase-like protein Precursor
				330	gpesqlvfldKFVLKngagnwlaqq	Bt_ENSBTAP00000045859	
				-	....	Rn	
				344	gpesqlvfldkfilqngagnwlaqq	Mm_ENSMUSP00000043570	
				351	gpesqlvfldKFVLKngagnwlaqq	Gg_ENSGALP00000036143	
				-	....	Xt	
				289	gpesqlvfldayvlrngsgnwlaeq	Dr_ENSDARP00000082121	
				-	....	Ce	
				-	....	Dm	
				-	....	Sc	
182	1	CI	ENSP00000339063 ENSG00000156508	423	esfsdyppglRFAVRdmrqtvavgv	Hs_ENSP00000339063	NP_001393 Elongation factor 1-alpha 1 (EF-1-alpha-1)(Elongation factor 1 A-1)(eEF1A-1)(Elongation factor Tu)(EF-Tu)(Leukocyte receptor cluster member 7)
				423	esfsdyppglRFAVRdmrqtvavgv	Bt_ENSBTAP00000019318	
				423	esfsdyppglRFAVRdmrqtvavgv	Rn_ENSRNOP00000013608	
				423	esfsdyppglRFAVRdmrqtvavgv	Mm_ENSMUSP00000042457	
				423	esfsdyppglRFAVRdmrqtvavgv	Gg_ENSGALP00000025606	
				423	esfsdyppglRFAVRdmrqtvavgv	Xt_ENSXETP00000002561	
				423	esfseyppglRFAVRdmrqtvavgv	Dr_ENSDARP00000002751	
				-	....	Ce	
				423	eafqefppglRFAVRdmrqtvavgv	Dm_FBpp0087142	
				421	eafseyppglRFAVRdmrqtvavgv	Sc_YBR118W	
183	1	CI	ENSP00000290390 ENSG00000159239	106	reamlqitewRFLARdegesavaed	Hs_ENSP00000290390	Uncharacterized protein C2orf81
				-	....	Bt	
				8	---mlqitewRFLARdegesavaed	Rn_ENSRNOP00000013311	
				8	---mlqitewRFLARdegesavaed	Mm_ENSMUSP00000032106	
				-	....	Gg	
				-	....	Xt	
				-	....	Dr	
				-	....	Ce	
				-	....	Dm	
				-	....	Sc	
183	2	C	ENSP00000290390 ENSG00000159239	451	sklplpnsrirflthpvlpdvars	Hs_ENSP00000290390	Uncharacterized protein C2orf81
				-	....	Bt	
				343	snlpflspgfRFLARnlvppdvasa	Rn_ENSRNOP00000013311	
				343	snvpflspgfrflprnpippdvast	Mm_ENSMUSP00000032106	
				-	....	Gg	
				-	....	Xt	
				-	....	Dr	
				-	....	Ce	
				-	....	Dm	
				-	....	Sc	
184	1	CI	ENSP00000366898 ENSG00000124440	305	skgqavtgqyRFLARsggylwtqtq	Hs_ENSP00000366898	NP_690008 Hypoxia-inducible factor 3 alpha (HIF-3 alpha)(HIF3 alpha 1)(Member of PAS protein 7)(Basic-helix-loop-helix-PAS protein MOP7)(Inhibitory PAS domain protein)(IPAS)
				305	skgqavtgqyRFLARsggylwtqtq	Bt_ENSBTAP00000025217	
				303	skgqavtgqyRFLARtggylwtqtq	Rn_ENSRNOP00000023490	
				305	skgqavtgqyRFLARtggylwtqtq	Mm_ENSMUSP00000104132	
				-	....	Gg	
				-	....	Xt	
				-	....	Dr	
				-	....	Ce	

				-<br>-	.........................<br>.........................	Dm<br>Sc	
185	1	CI	ENSP00000263791<br>ENSG00000128829	1124<br>1116<br>1124<br>1123<br>854<br>-<br>-<br>1126<br>1034<br>1100	lpfdlripfaRYVARnnilnlkryc<br>lpfdlrvpfaRYVARnnilnlkryc<br>lpfdlrvpfaRYVARnnilnlkryc<br>lpfdlrvpfaRYVARnnilnlkryc<br>-------------------------<br>.........................<br>.........................<br>lpmdlrqnfvrfcvrnsvqrmkrfn<br>lpcdlrtqfarhvtmnsvnlirryc<br>lqydltypmarylsknpsliskqyr	Hs_ENSP00000263791<br>Bt_ENSBTAP00000019083<br>Rn_ENSRNOP00000009222<br>Mm_ENSMUSP00000005233<br>Gg_ENSGALP00000015647<br>Xt<br>Dr<br>Ce_CE23118<br>Dm_FBpp0085148<br>Sc_YDR283C	NP_001013725 Eukaryotic translation initiation factor 2-alpha kinase 4 (EC 2.7.11.1)(GCN2-like protein)
185	2	C	ENSP00000263791<br>ENSG00000128829	1289<br>1281<br>1289<br>1288<br>854<br>-<br>-<br>1292<br>1205<br>1267	kqktgiaqlvkyglkdleevvg---<br>kqktgvaqlvkyglkdleevvg---<br>kqktgiaqlvkyslkdleevvg---<br>kqktgvaqlvkyslkdledvvg---<br>-------------------------<br>.........................<br>.........................<br>lkvdaarvlvdkairdleeivgtfk<br>rgkgeaaslargalreletvvg---<br>idsphlkkieds-lshiskvls---	Hs_ENSP00000263791<br>Bt_ENSBTAP00000019083<br>Rn_ENSRNOP00000009222<br>Mm_ENSMUSP00000005233<br>Gg_ENSGALP00000015647<br>Xt<br>Dr<br>Ce_CE23118<br>Dm_FBpp0085148<br>Sc_YDR283C	NP_001013725 Eukaryotic translation initiation factor 2-alpha kinase 4 (EC 2.7.11.1)(GCN2-like protein)
186	1	CI	ENSP00000325794<br>ENSG00000179580	45<br>46<br>45<br>46<br>-<br>-<br>67<br>-<br>-<br>-	shifckkcilRWLAR--qktcpccr<br>shifckkcilRWLAR--qktcpccr<br>shifckkcifqwlar--qntcpccr<br>shifckkcifRWLAR--qntcpccr<br>.........................<br>.........................<br>ghifcaqcvkaytenggsskcplds<br>.........................<br>.........................<br>.........................	Hs_ENSP00000325794<br>Bt_ENSBTAP00000044518<br>Rn_ENSRNOP00000018964<br>Mm_ENSMUSP00000008626<br>Gg<br>Xt<br>Dr_ENSDARP00000040549<br>Ce<br>Dm<br>Sc	NP_777563 RING finger protein 151
187	1	CI	ENSP00000377944<br>ENSG00000156697	648<br>647<br>642<br>645<br>-<br>640<br>649<br>-<br>654<br>810	kpsakkrr--RFLIKapegpprkdk<br>kpsakkrr--RFLIKapegpprkdk<br>kpsakkrr--RFLIKapegpprkdk<br>kpsarkrr--RFLIKapegpprkdk<br>.........................<br>kvskkkkr--KFIIKappapprkdq<br>kpsrrkrk--rftkkmdpppprqdr<br>.........................<br>skevmerrnkrlllklaapekrrde<br>kpknkkrkfikkvkgvvnkdkrrdk	Hs_ENSP00000377944<br>Bt_ENSBTAP00000017524<br>Rn_ENSRNOP00000051553<br>Mm_ENSMUSP00000079538<br>Gg<br>Xt_ENSXETP00000016264<br>Dr_ENSDARP00000062348<br>Ce<br>Dm_FBpp0075317<br>Sc_YML093W	U3 small nucleolar RNA-associated protein 14 homolog A (Serologically defined colon cancer antigen 16)(Antigen NY-CO-16)
188	1	CI	ENSP00000257414<br>ENSG00000092421	449<br>449<br>449<br>449<br>449<br>449<br>-<br>448<br>-<br>-	flgsekgiilKFLARignsgflnds<br>flgsekgiilKFLARtgnsgflnds<br>flgsekgiilKFLARigssgflngs<br>flgsekgiilKFLARigssgflngs<br>flgsekgiilKFLARtgnsgflnds<br>flgsergivlKFLARtgysgflsds<br>.........................<br>figtsdgkvlkvvevdgnatviqsa<br>.........................<br>.........................	Hs_ENSP00000257414<br>Bt_ENSBTAP00000041442<br>Rn_ENSRNOP00000005476<br>Mm_ENSMUSP00000019791<br>Gg_ENSGALP00000003601<br>Xt_ENSXETP00000040545<br>Dr<br>Ce_CE19225<br>Dm<br>Sc	Semaphorin-6A Precursor (Semaphorin VIA)(Sema VIA) (Semaphorin-6A-1)(SEMA6A-1)
189	1	CI	ENSP00000346839<br>ENSG00000115414	640<br>502<br>640<br>640<br>645<br>636<br>638<br>-<br>-<br>-	wnapqpshisKYILRwrpknsvgrw<br>wsapesshisKYILRwkpknspnrw<br>wnapepshitKYILRwrpktstgrw<br>wnapepshitKYILRwrpktstgrw<br>wnapktshisKYILRwrpkisgrhw<br>wnapqpshiknyilrwkpklktgpw<br>wnapasahitqyilkwrpknthiqw<br>.........................<br>.........................<br>.........................	Hs_ENSP00000346839<br>Bt_ENSBTAP00000010925<br>Rn_ENSRNOP00000019772<br>Mm_ENSMUSP00000054499<br>Gg_ENSGALP00000005654<br>Xt_ENSXETP00000023818<br>Dr_ENSDARP00000061037<br>Ce<br>Dm<br>Sc	NP_473375 Fibronectin Precursor (FN)(Cold-insoluble globulin) (CIG)
190	1	C	ENSP00000370430<br>ENSG00000006757	244<br>244<br>243<br>-<br>244<br>244<br>145<br>260	lyqcgfddtvKFLLKenwfe-----<br>lyrrgfddasRFLLKenwfe-----<br>iygmgmtdtvrflrrerwsq-----<br>.........................<br>lyqngfddavyfllkenwfe-----<br>ifqdgykdavnflqlekwyehc---<br>-------------------------<br>yyemgisdadifikknl--------	Hs_ENSP00000370430<br>Bt_ENSBTAP00000021067<br>Rn_ENSRNOP00000033754<br>Mm<br>Gg_ENSGALP00000026775<br>Xt_ENSXETP00000017908<br>Dr_ENSDARP00000092733<br>Ce_CE35445	NP_004641 Patatin-like phospholipase domain-containing protein 4 (EC 3.1.1.3)(Protein GS2)

				-<br>-	.........................<br>.........................	Dm<br>Sc	
191	1	CI	ENSP00000270747<br>ENSG00000142632	591<br>594<br>595<br>591<br>314<br>299<br>-<br>-<br>-<br>-	gkifplisqaRWLVRhgelvelapl<br>gkifplisqaRWLVRhgelvelapl<br>gkifplisqaRWLVRhgelvelapl<br>gkifplisqaRWLVRhgelvelapl<br>ckifplvsqsrrlvkcgeltaldfn<br>ckifplisqsrrlikhsevsalemn<br>.........................<br>.........................<br>.........................<br>.........................	Hs_ENSP00000270747<br>Bt_ENSBTAP00000007450<br>Rn_ENSRNOP00000033283<br>Mm_ENSMUSP00000006618<br>Gg_ENSGALP00000027876<br>Xt_ENSXETP00000040520<br>Dr<br>Ce<br>Dm<br>Sc	NP_694945 Rho guanine nucleotide exchange factor 19
192	1	CI	ENSP00000370803<br>ENSG00000103540	936<br>930<br>931<br>928<br>928<br>912<br>-<br>-<br>666<br>-	kaaemgmpnkKFLVKqnpsetrvlq<br>kaaemgmpskKFLVKqnpsetrvlq<br>kvaemgmankKFLLKqnpsetrvlq<br>kvaemgmpnkKFLLKqnpsetrvlq<br>kasemgmpskkivikqrspesrvlq<br>rvvhigmpkkklqpkprasenrilq<br>.........................<br>.........................<br>-------------------------<br>.........................	Hs_ENSP00000370803<br>Bt_ENSBTAP00000005166<br>Rn_ENSRNOP00000035030<br>Mm_ENSMUSP00000102167<br>Gg_ENSGALP00000011207<br>Xt_ENSXETP00000047018<br>Dr<br>Ce<br>Dm_FBpp0077044<br>Sc	Centrosomal protein of 110 kDa (Cep110)
193	1	CI	ENSP00000360811<br>ENSG00000107187	46<br>46<br>46<br>46<br>38<br>0<br>-<br>58<br>132<br>-	cagcdqhildRFILKaldrhwhskc<br>cagcdqhildRFILKaldrhwhskc<br>cagcdqhildRFILKaldrhwhskc<br>cagcdqhildRFILKaldrhwhskc<br>cagcnqhivdRFILKvldrhwhskc<br>-------------------------<br>.........................<br>cslcdkkirdrfvskvngrcyhssc<br>cggchelildRFILKvlertwhakc<br>.........................	Hs_ENSP00000360811<br>Bt_ENSBTAP00000051711<br>Rn_ENSRNOP00000044069<br>Mm_ENSMUSP00000028302<br>Gg_ENSGALP00000002660<br>Xt_ENSXETP00000001613<br>Dr<br>Ce_CE28335<br>Dm_FBpp0080712<br>Sc	NP_055379 LIM/homeobox protein Lhx3 (LIM homeobox protein 3)
194	1	CI	ENSP00000182377<br>ENSG00000064763	443<br>443<br>443<br>443<br>-<br>-<br>-<br>-<br>-<br>-	yienyvlgvkKYLLKedmagipkak<br>yienyvlgvkKYLLKedmagipeak<br>yienyvlgvkKYLLKedlagipkak<br>yienyvlgvkKYLLKedlagipkak<br>.........................<br>.........................<br>.........................<br>.........................<br>.........................<br>.........................	Hs_ENSP00000182377<br>Bt_ENSBTAP00000014725<br>Rn_ENSRNOP00000002528<br>Mm_ENSMUSP00000032443<br>Gg<br>Xt<br>Dr<br>Ce<br>Dm<br>Sc	NP_060569 Fatty acyl-CoA reductase 2 (EC 1.2.1.-)(Male sterility domain-containing protein 1)
195	1	CI	ENSP00000312395<br>ENSG00000221879	6<br>6<br>6<br>6<br>6<br>6<br>6<br>0<br>5<br>-	-----makhlKFIARtvmvqegnve<br>-----makhlKFIARtvmvqegnve<br>-----makhlKFIARtvmvqegnve<br>-----makhlKFIARtvmvqegnve<br>-----manhlrfigrtvmvqngnvd<br>-----manhlKFIARtvmvpsgnvd<br>-----manhlrfvsrtvmvqngnvd<br>-----------------mvqnndvd<br>------mrhvqflartvlvqnnnve<br>.........................	Hs_ENSP00000312395<br>Bt_ENSBTAP00000033682<br>Rn_ENSRNOP00000038594<br>Mm_ENSMUSP00000066990<br>Gg_ENSGALP00000000617<br>Xt_ENSXETP00000058197<br>Dr_ENSDARP00000095428<br>Ce_CE09788<br>Dm_FBpp0082232<br>Sc	NP_114107 28S ribosomal protein S21, mitochondrial (S21mt)(MRP-S21)
196	1	CI	ENSP00000364585<br>ENSG00000179051	293<br>198<br>198<br>291<br>198<br>284<br>266<br>-<br>260<br>-	ygqlghnsdgKFIARaqrieydcel<br>ygqlghnsdgKFIARaqrieydcel<br>ygqlghnsdgKFIARaqrieydcel<br>ygqlghnsdgKFIARaqrieydcel<br>ygqlghnsdgKFIARaqrieydcel<br>ygqlghnsdgKYIARaqrieydcel<br>ygqlghnsdgKFIARaqriefdcel<br>.........................<br>ygqlghntdakyfvnanklsfhfet<br>.........................	Hs_ENSP00000364585<br>Bt_ENSBTAP00000011314<br>Rn_ENSRNOP00000008940<br>Mm_ENSMUSP00000071163<br>Gg_ENSGALP00000039872<br>Xt_ENSXETP00000032386<br>Dr_ENSDARP00000004838<br>Ce<br>Dm_FBpp0078816<br>Sc	NP_001129676 Protein RCC2 (Telophase disk protein of 60 kDa)(RCC1-like protein TD-60)
197	1	C	ENSP00000364586<br>ENSG00000136531	1918<br>-<br>1920<br>1919<br>1926<br>1925<br>1910<br>-	saiiiqrayrRYLLK----------<br>.........................<br>saiviqrayrRYLLK----------<br>saiviqrayrRYLLK----------<br>savviqrafrrhllr----------<br>aaiviqrcyrcyalr----------<br>savviqrafrrhrir----------<br>.........................	Hs_ENSP00000364586<br>Bt<br>Rn_ENSRNOP00000007069<br>Mm_ENSMUSP00000028377<br>Gg_ENSGALP00000017912<br>Xt_ENSXETP00000019653<br>Dr_ENSDARP00000085427<br>Ce	NP_001035232 Sodium channel protein type 2 subunit alpha (Sodium channel protein type II subunit alpha)(Voltage-gated sodium channel subunit alpha Nav1.2)(Sodium channel protein, brain II subunit alpha)(HBSC II)

				2000	carliqhawrkhkargegggsfepd	Dm_FBpp0110321	
				-	....	Sc	
198	1	CI	ENSP00000360815 ENSG00000085831	436	kiagkslpteKFAIRksrryfssnp	Hs_ENSP00000360815	Tetratricopeptide repeat protein 39A (TPR repeat protein 39A) (Differentially expressed in MCF7 with estradiol protein 6)(DEME-6)
				396	kiagkslpteKFAIRksrrylspkp	Bt_ENSBTAP00000003183	
				399	kiagkslpteKFAIRksrrylspnp	Rn_ENSRNOP00000013459	
				401	kiagkslpteKFAIRksrrylspnp	Mm_ENSMUSP00000102229	
				378	kiagkslpteKFAIRkarrylssnp	Gg_ENSGALP00000017136	
				394	kiagkslpteKFAIRksrrylaqkl	Xt_ENSXETP00000006193	
				302	kiagksppteKFAIRkarrykasnp	Dr_ENSDARP00000085503	
				-	....	Ce	
				-	....	Dm	
				544	tfksknlpldRFILRkveqfkakke	Sc_YKR018C	
199	1	CI	ENSP00000364178 ENSG00000155657	11532	dikprdqgeyRFIAKdkearaklel	Hs_ENSP00000364178	titin isoform novex-3
				-	....	Bt	
				-	....	Rn	
				14771	dikprdqgeyRFIAKdkearaklel	Mm_ENSMUSP00000097561	
				-	....	Gg	
				-	....	Xt	
				2707	eirprdqgeyrviakdkdaraklel	Dr_ENSDARP00000001468	
				1542	iidndtivpqllqvaaaanklklen	Ce_CE36727	
				-	....	Dm	
				-	....	Sc	
199	2	I	ENSP00000364178 ENSG00000155657	22641	idncdrndagKYILKlenssgsksa	Hs_ENSP00000364178	titin isoform novex-3
				-	....	Bt	
				-	....	Rn	
				25881	idncdrndagKYILKlenssgsksa	Mm_ENSMUSP00000097561	
				-	....	Gg	
				-	....	Xt	
				13835	inicdrndagkytltlqnsagtksa	Dr_ENSDARP00000001468	
				11375	eksikapnkskvttsfades--lts	Ce_CE36727	
				-	....	Dm	
				-	....	Sc	
199	3	C	ENSP00000364178 ENSG00000155657	31419	dttvssdsvaKFAVKatgeprptai	Hs_ENSP00000364178	titin isoform novex-3
				-	....	Bt	
				-	....	Rn	
				34659	dttvssdsvakftikvtgepqptit	Mm_ENSMUSP00000097561	
				-	....	Gg	
				-	....	Xt	
				22500	-------------------------	Dr_ENSDARP00000001468	
				18562	-------------------------	Ce_CE36727	
				-	....	Dm	
				-	....	Sc	
200	1	CI	ENSP00000240361 ENSG00000121101	507	kdrtmnlqdiRYILKndlk-----d	Hs_ENSP00000240361	Testis-expressed protein 14 (Testis-expressed sequence 14) (Protein kinase-like protein SgK307)(Sugen kinase 307)
				461	kdrtmnlqdiryimkndlk-----d	Bt_ENSBTAP00000013424	
				283	knrtmnlqdiRYILKndlkasleie	Rn_ENSRNOP00000009077	
				501	knrtmnlqdiRYILKndlk-----e	Mm_ENSMUSP00000054444	
				507	knrsmtlqdiqyilkndlk-----d	Gg_ENSGALP00000008062	
				-	....	Xt	
				-	....	Dr	
				-	....	Ce	
				-	....	Dm	
				-	....	Sc	
201	1	CI	ENSP00000248975 ENSG00000128245	106	vcndvlslldKFLIKncndfqyesk	Hs_ENSP00000248975	NP_003396 14-3-3 protein eta (Protein AS1)
				77	vcndvlalldKFLIKncndfqyesk	Bt_ENSBTAP00000041575	
				106	vcndvlalldKFLIKncndfqyesk	Rn_ENSRNOP00000024388	
				106	vcndvlalldKFLIKncndfqyesk	Mm_ENSMUSP00000019109	
				106	vcndvlalldKYLIKncndfqyesk	Gg_ENSGALP00000010888	
				106	vcsevlslldKFLIKncndfqyesk	Xt_ENSXETP00000009115	
				-	....	Dr	
				-	....	Ce	
				-	....	Dm	
				-	....	Sc	
202	1	CI	ENSP00000084795 ENSG00000063177	33	iylrllvklyRFLARrtnstfnqvv	Hs_ENSP00000084795	NP_000970 60S ribosomal protein L18
				33	iylrllvklyRFLARrtnstfnqvv	Bt_ENSBTAP00000020452	
				32	iylrllvklyRFLARrtnstfnqvv	Rn_ENSRNOP00000028555	
				33	iylrllvklyRFLARrtnstfnqvv	Mm_ENSMUSP00000103365	
				-	....	Gg	
				32	iylrllvklyRFLARrtnssfnrvv	Xt_ENSXETP00000006884	
				33	iylrllvklyrflsrrsdapfnkvi	Dr_ENSDARP00000043172	
				33	pylrllsklyaflarrtgekfnaiv	Ce_CE16650	

				33	vylrllvklyrflqrrtnkkfnrii	Dm_FBpp0076602	
				34	vylkllvklytflarrtdapfnkvv	Sc_YNL301C	
203	1	CI	ENSP00000350049 ENSG00000215271	384	peiiqwfgdtRYALKhgqlkwfrdn	Hs_ENSP00000350049	NP_065885 Homeobox and leucine zipper protein Homez (Homeodomain leucine zipper-containing factor)
				-	....	Bt	
				377	peiiqwfgdtRYALKhgqlkwfrdn	Rn_ENSRNOP00000019979	
				378	peiiqwfgdtRYALKhgqlkwfrdn	Mm_ENSMUSP00000079929	
				-	....	Gg	
				-	....	Xt	
				533	adviqwfgdtRYAVKnghvrwvhad	Dr_ENSDARP00000042099	
				-	....	Ce	
				-	....	Dm	
				-	....	Sc	
204	1	CI	ENSP00000313822 ENSG00000102984	215	lleaqtpsvrKWALRrqneplevrl	Hs_ENSP00000313822	NP_060000 Zinc finger protein 821
				257	lleaqtpsvrKWALRrqneplevrl	Bt_ENSBTAP00000032245	
				257	lletqtpsvrKWALRrqneplevrl	Rn_ENSRNOP00000000279	
				258	lletqtpsvrKWALRrqneplevrl	Mm_ENSMUSP00000034163	
				256	lleaqtpgmrKWALRrqneplevrl	Gg_ENSGALP00000001189	
				244	lmtdqtptvrkwaarrqnepaaarv	Xt_ENSXETP00000050590	
				-	....	Dr	
				-	....	Ce	
				-	....	Dm	
				-	....	Sc	
205	1	CI	ENSP00000386350 ENSG00000138430	262	sekdyirkknKWLIKikewvdkydp	Hs_ENSP00000386350	NP_037473 Obg-like ATPase 1 (EC 3.6.3.-)(GTP-binding protein 9)
				242	sekdyirkknKWLIKikewvdkydp	Bt_ENSBTAP00000009160	
				242	sekdyirkknKWLIKikewvdksdp	Rn_ENSRNOP00000026040	
				242	sekdyirkknKWLIKikewvdkydp	Mm_ENSMUSP00000028517	
				242	sekdyirkknKWLIKikewvdkhdp	Gg_ENSGALP00000015171	
				242	sekdyirkknKWLIKikewvdkhdp	Xt_ENSXETP00000018601	
				242	sekdyirkknKWLVKikewvdahdp	Dr_ENSDARP00000045298	
				239	sekdyirkknkwlpkikawidtnda	Ce_CE14708	
				240	sdkdfirkknkwlpkikewidkndp	Dm_FBpp0088409	
				244	serdyirkknkhllrikewvdkysp	Sc_YBR025C	
206	1	C	ENSP00000261407 ENSG00000111684	175	dqnslsseqqKYAIRgvpsllevag	Hs_ENSP00000261407	NP_005759 Lysophosphatidylcholine acyltransferase (EC 2.3.1.23) (Membrane-bound O-acyltransferase domain-containing protein 5)(O-acyltransferase domain-containing protein 5)
				173	dqksltseqqiyaiwgvpslleisg	Bt_ENSBTAP00000037616	
				175	drnslsseqqkyailgvpsllevag	Rn_ENSRNOP00000017090	
				175	dgnsltseqqKYAIRgvpsllevag	Mm_ENSMUSP00000004381	
				157	dpelltpeqrRFAVRgvptllevsg	Gg_ENSGALP00000023450	
				156	dkesmteeqmryavpgvpslmevcg	Xt_ENSXETP00000051573	
				-	....	Dr	
				151	kpehlkpdqkltaisdkpglleiaa	Ce_CE32713	
				163	eeselskdqketalkkppsllella	Dm_FBpp0074786	
				-	....	Sc	
207	1	CI	ENSP00000351100 ENSG00000120314	248	nwngfgatsdRFALRaes-idcmvp	Hs_ENSP00000351100	NP_060176 WD repeat-containing protein 55
				248	nwdgfgatsdRFALRaes-idcmvp	Bt_ENSBTAP00000020021	
				-	....	Rn	
				249	nwngfgatsdRFALRaes-idcivp	Mm_ENSMUSP00000039010	
				-	....	Gg	
				-	....	Xt	
				245	nwngfgatsdrfavqaes-vdcivp	Dr_ENSDARP00000018714	
				222	nlneygnllervesgfemgvngive	Ce_CE16634	
				364	nwgqfgyhcdmypgiksp-islmip	Dm_FBpp0081907	
				-	....	Sc	
208	1	CI	ENSP00000320893 ENSG00000177981	255	sapgtlktlaRYAVRrslglqylpd	Hs_ENSP00000320893	NP_077000 Ankyrin repeat and SOCS box protein 8 (ASB-8)
				255	sapgtlktlsRYAVRrslglqylpd	Bt_ENSBTAP00000000361	
				255	sapgtlktlaRYAVRrslglqylpd	Rn_ENSRNOP00000013704	
				255	sapgtlktlaRYAVRrslglqylpd	Mm_ENSMUSP00000057864	
				249	sapgtlqtlsRYAVRrslgvrflpe	Gg_ENSGALP00000021645	
				247	sypcslkaisrrvvrgslgecllsk	Xt_ENSXETP00000022739	
				256	aqppslralarcavrsslgvqylps	Dr_ENSDARP00000012241	
				-	....	Ce	
				-	....	Dm	
				-	....	Sc	
209	1	CI	ENSP00000376774 ENSG00000105929	128	kqsfleltelKYLLKktqdffetet	Hs_ENSP00000376774	NP_065683 V-type proton ATPase 116 kDa subunit a isoform 4 (V-ATPase 116 kDa isoform a4) (Vacuolar proton translocating ATPase 116 kDa subunit a isoform 4)(Vacuolar proton translocating
				128	kknfleltefkhllkktqdffetea	Bt_ENSBTAP00000005588	
				128	kksfleltelKYLLKktqdffetet	Rn_ENSRNOP00000018180	
				128	kksfleltelKYLLKktqdffetet	Mm_ENSMUSP00000110558	
				129	kqnflelmelkhllkktqdffeaet	Gg_ENSGALP00000020870	
				-	....	Xt	
				-	....	Dr	
				-	....	Ce	

				-<br>-	.........................<br>.........................	Dm<br>Sc	ATPase 116 kDa subunit a kidney isoform)
210	1	CI	ENSP00000328236<br>ENSG00000184445	866<br>864<br>864<br>866<br>862<br>-<br>-<br>856<br>816<br>-	n--keimrvvRYILKqdvpssleda<br>n--keimrvvRYILKqdvpssleda<br>n--keimrvvRYILKqdipssleda<br>n--keimrviRYILKqdipssleda<br>kdkemimrlvKYILKqdtptsleda<br>.........................<br>.........................<br>d----iellttpdsnmdiiltircm<br>in---lelfmmrivklnlpdmlddi<br>.........................	Hs_ENSP00000328236<br>Bt_ENSBTAP00000025676<br>Rn_ENSRNOP00000050210<br>Mm_ENSMUSP00000031366<br>Gg_ENSGALP00000007179<br>Xt<br>Dr<br>Ce_CE07281<br>Dm_FBpp0085156<br>Sc	NP_055523 Kinetochore-associated protein 1 (Rough deal homolog)(HsROD)(hRod)(Rod)
211	1	CI	ENSP00000366783<br>ENSG00000173064	2349<br>1371<br>-<br>2490<br>1369<br>1373<br>-<br>-<br>-<br>-	assavgtalpKFAIRgmlktfglhg<br>asstvgtalpKFAIRgmlktfglhg<br>.........................<br>asstvgtalpKFAIRgmlktfslhg<br>tssavgtalpKFAIRgmlktfglhg<br>agttvgtalpKFAIRgmlktfslhg<br>.........................<br>.........................<br>.........................<br>.........................	Hs_ENSP00000366783<br>Bt_ENSBTAP00000011545<br>Rn<br>Mm_ENSMUSP00000112647<br>Gg_ENSGALP00000023401<br>Xt_ENSXETP00000009363<br>Dr<br>Ce<br>Dm<br>Sc	NP_001103132 Transmembrane protein C12orf51
212	1	CI	ENSP00000387040<br>ENSG00000165323	544<br>0<br>543<br>539<br>545<br>0<br>503<br>-<br>-<br>-	ldfesspeiyRFIVRasdwgspyrh<br>-------------------------<br>ldfesspetyRFIVRasdwgspyrh<br>ldfesspetyRFIVRasdwgspyrh<br>ldfesspesyRFIVRasdwgspyrh<br>-------------------------<br>ldfesslesfsfvvrasdwgspyrr<br>.........................<br>.........................<br>.........................	Hs_ENSP00000387040<br>Bt_ENSBTAP00000005332<br>Rn_ENSRNOP00000015976<br>Mm_ENSMUSP00000080808<br>Gg_ENSGALP00000027793<br>Xt_ENSXETP00000037341<br>Dr_ENSDARP00000042081<br>Ce<br>Dm<br>Sc	NP_001008781 Protocadherin Fat 3 Precursor (hFat3)(FAT tumor suppressor homolog 3)
213	1	CI	ENSP00000383392<br>ENSG00000154654	256<br>150<br>238<br>256<br>256<br>255<br>227<br>-<br>277<br>-	gklieen--eKYILKgsnteltvrn<br>gkfieen--ekyslrgsnteltvrn<br>gklieen--eKYILKgsnteltvrn<br>gklieen--eKYILKgsnteltvrn<br>gklieen--eKYVLRgsnaeltird<br>gklveen--ekydlrednteltvkn<br>gvqlqes--eryvmrargttltvrn<br>.........................<br>atqlnvatadrfqvnpqtglvtiss<br>.........................	Hs_ENSP00000383392<br>Bt_ENSBTAP00000034315<br>Rn_ENSRNOP00000002895<br>Mm_ENSMUSP00000063468<br>Gg_ENSGALP00000025332<br>Xt_ENSXETP00000015379<br>Dr_ENSDARP00000091452<br>Ce<br>Dm_FBpp0070633<br>Sc	NP_004531 Neural cell adhesion molecule 2 Precursor (N-CAM 2)
214	1	CI	ENSP00000252595<br>ENSG00000130304	342<br>343<br>342<br>342<br>348<br>-<br>343<br>-<br>411<br>-	tvvqyigeicRYLLKqpvreaerrh<br>tvvqyigeicRYLLKqpvreaegrh<br>tvvqyigeicRYLLRqpvrdverrh<br>tvvqyigeicRYLLRqpvrdveqrh<br>tiiqyigeicryllnqpvresetqh<br>.........................<br>tvvqyigeicryllaqpvrpserqh<br>.........................<br>tvgqyigemaryilatpsaphdrnh<br>.........................	Hs_ENSP00000252595<br>Bt_ENSBTAP00000022311<br>Rn_ENSRNOP00000024659<br>Mm_ENSMUSP00000034267<br>Gg_ENSGALP00000021686<br>Xt<br>Dr_ENSDARP00000006540<br>Ce<br>Dm_FBpp0071865<br>Sc	NP_940982 Long-chain fatty acid transport protein 1 (Fatty acid transport protein 1)(FATP-1)(EC 6.2.1.-)(Solute carrier family 27 member 1)
215	1	CI	ENSP00000373770<br>ENSG00000204764	331<br>333<br>330<br>331<br>-<br>-<br>-<br>-<br>-<br>-	sdpgnyhefcRFLARlktnyqlgel<br>sdpgnyhefcRFLARlktnyqlgel<br>sdpgnyhefcRFLARlktnyqlgel<br>sdpgnyhefcRFLARlktnyqlgel<br>.........................<br>.........................<br>.........................<br>.........................<br>.........................<br>.........................	Hs_ENSP00000373770<br>Bt_ENSBTAP00000034423<br>Rn_ENSRNOP00000006952<br>Mm_ENSMUSP00000099879<br>Gg<br>Xt<br>Dr<br>Ce<br>Dm<br>Sc	NP_075048 Ran-binding protein 17
216	1	C	ENSP00000215591<br>ENSG00000099821	511<br>496<br>481<br>483<br>291<br>495<br>531<br>-	arelsartfsrhvvqrqrvsgqvqa<br>ahelgvr-----vlkrkrlrnqvee<br>aqnlglqvfnrhlvkqkqvtnhvqk<br>ahnlglrvlnrhlvkqkqvtnhvqk<br>arelgskvynryltqrklhsgqlek<br>srdmgtkinnrysihkklqeshinr<br>ademgnrvynlycirqksqsqmvek<br>.........................	Hs_ENSP00000215591<br>Bt_ENSBTAP00000020034<br>Rn_ENSRNOP00000032448<br>Mm_ENSMUSP00000020580<br>Gg_ENSGALP00000002096<br>Xt_ENSXETP00000014328<br>Dr_ENSDARP00000082602<br>Ce	NP_005026 DNA-directed RNA polymerase, mitochondrial Precursor (EC 2.7.7.6)(MtRPOL)

				627<br>540	ykelgqkvqqryqieqkkhngtlek<br>rtaravisvgkaiemefrseqvlks	Dm_FBpp0077654<br>Sc_YFL036W	
216	2	C	ENSP00000215591<br>ENSG00000099821	1185<br>1174<br>1153<br>1155<br>971<br>1172<br>1203<br>-<br>1316<br>1223	hsepilqdlsRFLVKrfcsep----<br>hsqpilhdlsRFLIKrfcsdsr---<br>hsepiledlakflekrfcsvs--ks<br>hsqpiledlakflkkrfcsvssiks<br>hsqkiledlskfmlenycspdresr<br>hsqpilqnlsqfllqkycqtlssdk<br>hsqpileelswfllqkysrrpp---<br>.........................<br>hsqpileqlsefmrhtysfkdsdfi<br>hevdlvlrlkeefdqryknyvkigk	Hs_ENSP00000215591<br>Bt_ENSBTAP00000020034<br>Rn_ENSRNOP00000032448<br>Mm_ENSMUSP00000020580<br>Gg_ENSGALP00000002096<br>Xt_ENSXETP00000014328<br>Dr_ENSDARP00000082602<br>Ce<br>Dm_FBpp0077654<br>Sc_YFL036W	NP_005026 DNA-directed RNA polymerase, mitochondrial Precursor (EC 2.7.7.6)(MtRPOL)
217	1	CI	ENSP00000284395<br>ENSG00000154237	1054<br>1000<br>1003<br>1030<br>987<br>1033<br>967<br>1417<br>1391<br>-	msfvpvgfwqRFIARmlis------<br>msfvpvgfwqRFIARmlis------<br>msfvpvgfwqRFIARmlis------<br>msfvpvgfwqRFIARmlis------<br>msfvpvgfwqRFIARmlis------<br>msfvpvgfwqRFIARmlis------<br>msfvpagfweRFIARmlis------<br>layipsgfwsrlvtrivgdknvcaa<br>mtyfpsgfwsrlitriladeqiiea<br>.........................	Hs_ENSP00000284395<br>Bt_ENSBTAP00000002052<br>Rn_ENSRNOP00000016959<br>Mm_ENSMUSP00000015277<br>Gg_ENSGALP00000011551<br>Xt_ENSXETP00000034737<br>Dr_ENSDARP00000033785<br>Ce_CE41939<br>Dm_FBpp0111700<br>Sc	Leucine-rich repeat serine/threonine-protein kinase 1 (EC 2.7.11.1)
218	1	CI	ENSP00000311513<br>ENSG00000048649	89<br>-<br>88<br>94<br>-<br>-<br>-<br>-<br>-<br>-	gksvtadrweKYLIKicqefnstwa<br>.........................<br>gksvtadrweKYLIKicqefnstwa<br>gksvtadrweKYLIKicqefnstwa<br>.........................<br>.........................<br>.........................<br>.........................<br>.........................<br>.........................	Hs_ENSP00000311513<br>Bt<br>Rn_ENSRNOP00000031549<br>Mm_ENSMUSP00000037409<br>Gg<br>Xt<br>Dr<br>Ce<br>Dm<br>Sc	NP_057662 Remodeling and spacing factor 1 (Rsf-1)(Hepatitis B virus X-associated protein)(HBV pX-associated protein 8)(p325 subunit of RSF chromatin-remodeling complex)
219	1	CI	ENSP00000319303<br>ENSG00000166435	222<br>11<br>227<br>227<br>114<br>11<br>-<br>-<br>-<br>-	easvtsltskRYILRfpaletlmld<br>easvtslttkRYILRfpaletlmld<br>easvtsltskKYILRfpaletlmld<br>easmtsltskKYILRfpaletlmld<br>nasspspvshqthlrfqslevllld<br>elsasatqkdlsltlfsaleilmld<br>.........................<br>.........................<br>.........................<br>.........................	Hs_ENSP00000319303<br>Bt_ENSBTAP00000042414<br>Rn_ENSRNOP00000032195<br>Mm_ENSMUSP00000035929<br>Gg_ENSGALP00000027918<br>Xt_ENSXETP00000006580<br>Dr<br>Ce<br>Dm<br>Sc	NP_892014 X-ray radiation resistance-associated protein 1
220	1	CI	ENSP00000319799<br>ENSG00000178804	94<br>95<br>89<br>88<br>-<br>80<br>102<br>-<br>-<br>-	kyptvdvlrfKYLLKqalatgmrrg<br>kyptvdalrlnhllkqalatglhrg<br>kyptvgttrfKYLLKqaletgmrrg<br>kyptvdttrfKYLLKqaletgvrrg<br>.........................<br>ahptvdplrlKFLLRtalnkglekg<br>kyttvdetrlkymvrralnkgmdtg<br>.........................<br>.........................<br>.........................	Hs_ENSP00000319799<br>Bt_ENSBTAP00000002369<br>Rn_ENSRNOP00000048205<br>Mm_ENSMUSP00000036951<br>Gg<br>Xt_ENSXETP00000003084<br>Dr_ENSDARP00000059040<br>Ce<br>Dm<br>Sc	NP_722575 Histone H1oo (Oocyte-specific histone H1)(Oocyte-specific linker histone H1)(osH1)
221	1	CI	ENSP00000274364<br>ENSG00000145703	1074<br>1026<br>-<br>1074<br>-<br>-<br>-<br>-<br>-<br>-	ssldllpyglRYIAKvlknsihekf<br>ssldllpyglRYIAKvlknsihekf<br>.........................<br>ssldllpyglRYIAKvlknsirekf<br>.........................<br>.........................<br>.........................<br>.........................<br>.........................<br>.........................	Hs_ENSP00000274364<br>Bt_ENSBTAP00000001190<br>Rn<br>Mm_ENSMUSP00000067685<br>Gg<br>Xt<br>Dr<br>Ce<br>Dm<br>Sc	NP_006624 Ras GTPase-activating-like protein IQGAP2
222	1	C	ENSP00000295851<br>ENSG00000138443	154<br>154<br>83<br>154<br>154<br>154<br>117<br>156	tilddighgvKWLLRfkvstqnmkm<br>tilddighgvk------vstqnmkm<br>tilddighgvk------vstqnmkm<br>tilddighgvKWLLRfkvstqnmkm<br>tilddighgvk------vstqnmkm<br>tilddtghgvk------vstqnmkm<br>smlddighgvKWLLRfkvntqnmkm<br>svldgighgvrtsdppraapisrat	Hs_ENSP00000295851<br>Bt_ENSBTAP00000014635<br>Rn_ENSRNOP00000025215<br>Mm_ENSMUSP00000084679<br>Gg_ENSGALP00000014166<br>Xt_ENSXETP00000009002<br>Dr_ENSDARP00000042013<br>Ce_CE29545	Abl interactor 2 (Abelson interactor 2)(Abi-2)(Abl-binding protein 3)(AblBP3)(Arg-binding protein 1)(ArgBP1)

				166<br>-	smldeighginsaqhsqvrqkhrgs<br>.........................	Dm_FBpp0082371<br>Sc	
223	1	CI	ENSP00000374875<br>ENSG00000211705	28<br>28<br>-<br>-<br>-<br>-<br>-<br>-<br>-<br>-	pvkagvtqtpRYLIKtrgqqvtlsc<br>lvdsgvtqipKYLIKsrkqqvilrc<br>.........................<br>.........................<br>.........................<br>.........................<br>.........................<br>.........................<br>.........................<br>.........................	Hs_ENSP00000374875<br>Bt_ENSBTAP00000048427<br>Rn<br>Mm<br>Gg<br>Xt<br>Dr<br>Ce<br>Dm<br>Sc	T-cell receptor beta V gene segment
224	1	C	ENSP00000368984<br>ENSG00000123737	12<br>12<br>12<br>12<br>0<br>12<br>12<br>12<br>18<br>13	ketplsncerRFLLRaieekkrldg<br>ketplsncerRFLLRaieekkrldg<br>ketplsncerRFLLRaieekkrldg<br>ketplsncerRFLLRaieekkrldg<br>-------------------------<br>ketplsncerafllqaiaerkrldg<br>rdtplsncerlfllkaikekkrldg<br>rvqpvtncekavilealkigkrfdf<br>fepgkskpersfvqlavkqnqrldg<br>kdieisaseskfilealrqnyrldg	Hs_ENSP00000368984<br>Bt_ENSBTAP00000006502<br>Rn_ENSRNOP00000020662<br>Mm_ENSMUSP00000029269<br>Gg_ENSGALP00000019364<br>Xt_ENSXETP00000045663<br>Dr_ENSDARP00000005262<br>Ce_CE26424<br>Dm_FBpp0074129<br>Sc_YDR280W	NP_001029366 Exosome complex exonuclease RRP45 (EC 3.1.13.-)(Exosome component 9)(Polymyositis/scleroderma autoantigen 1)(Autoantigen PM/Scl 1)(Polymyositis/scleroderma autoantigen 75 kDa)(PM/Scl-75)(P75 polymyositis-scleroderma overlap syndrome-associated autoantigen)
225	1	CI	ENSP00000289968<br>ENSG00000140750	380<br>363<br>363<br>408<br>375<br>-<br>355<br>-<br>381<br>335	nfvnfr-yliKFLAKlaqtsdvnkm<br>nfvnfr-yliKFLAKlaqtsdinkm<br>nfvnfr-yliKFLAKlaqtsdvnkm<br>nfvnfr-yliKFLAKlaqtsdvnkm<br>yhanfryyliKFLAKlaqnsdvnkm<br>.........................<br>nktnfr-ylvKFLAKlalesdvnkm<br>.........................<br>nyanlr-yltrflsivqqrsalnkm<br>----ltiylldllslfarqsqfnlm	Hs_ENSP00000289968<br>Bt_ENSBTAP00000026703<br>Rn_ENSRNOP00000038504<br>Mm_ENSMUSP00000102050<br>Gg_ENSGALP00000009572<br>Xt<br>Dr_ENSDARP00000084198<br>Ce<br>Dm_FBpp0083230<br>Sc_YDR389W	NP_001006635 Rho GTPase-activating protein 17 (Rho-type GTPase-activating protein 17)(RhoGAP interacting with CIP4 homologs protein 1)(RICH-1)
226	1	CI	ENSP00000302251<br>ENSG00000168505	333<br>334<br>333<br>333<br>-<br>325<br>327<br>-<br>407<br>-	ivvpipvhvsRFAIRsqhqqleqar<br>ivvpipvhvsRFAIRsqhqqleqar<br>ivvpipvhvsRFAIRsqhqqleqar<br>ivvpipvhvsRFAIRsqhqqleqar<br>.........................<br>ivvpipvhvsRFAIRsqhqqleqar<br>ivvpipvhvsRFAIRsqhqqleqar<br>.........................<br>ivvpipvhvnRFAVRsqhqqlekmc<br>.........................	Hs_ENSP00000302251<br>Bt_ENSBTAP00000011716<br>Rn_ENSRNOP00000026419<br>Mm_ENSMUSP00000048508<br>Gg<br>Xt_ENSXETP00000007133<br>Dr_ENSDARP00000004972<br>Ce<br>Dm_FBpp0087667<br>Sc	NP_001476 Homeobox protein GBX-2 (Gastrulation and brain-specific homeobox protein 2)
227	1	CI	ENSP00000240662<br>ENSG00000121361	35<br>35<br>35<br>35<br>35<br>0<br>35<br>-<br>-<br>-	prirdrlpkaRFIAKsgacnlahkn<br>prirdrlpkaRFIAKsgacnlahkn<br>prirdrlpkaRFIAKsgacnlahkn<br>prirdrlpkaRFIAKsgacnlahkn<br>prirdrprkaRFIAKngacnlahkn<br>-------------------------<br>pvfrdrvnkaRFIAKsgacnlahkn<br>.........................<br>.........................<br>.........................	Hs_ENSP00000240662<br>Bt_ENSBTAP00000003307<br>Rn_ENSRNOP00000018057<br>Mm_ENSMUSP00000032374<br>Gg_ENSGALP00000021601<br>Xt_ENSXETP00000026069<br>Dr_ENSDARP00000067030<br>Ce<br>Dm<br>Sc	NP_004973 ATP-sensitive inward rectifier potassium channel 8 (Potassium channel, inwardly rectifying subfamily J member 8)(Inwardly rectifier K(+) channel Kir6.1)(uKATP-1)
228	1	CI	ENSP00000296953<br>ENSG00000164463	474<br>474<br>475<br>475<br>471<br>480<br>-<br>-<br>-<br>-	myqknglhhgKYAVKksrrtdvedl<br>myqknglhhgKYAVKksrrtdvedl<br>myqknglhhgKYAVKksrrtdvedl<br>myqknglhhgKYAVKksrrtdvedl<br>myqknglhhgkyaakksrrtdvedl<br>myqknglhhgkytvkksrrtdvedl<br>.........................<br>.........................<br>.........................<br>.........................	Hs_ENSP00000296953<br>Bt_ENSBTAP00000002618<br>Rn_ENSRNOP00000028186<br>Mm_ENSMUSP00000059102<br>Gg_ENSGALP00000004529<br>Xt_ENSXETP00000027827<br>Dr<br>Ce<br>Dm<br>Sc	NP_705835 UPF0474 protein C5orf41
229	1	CI	ENSP00000344860<br>ENSG00000188624	92<br>-<br>91<br>91<br>-	ccpesfgpqqKFLVKlrvlgmksqc<br>.........................<br>ccpesysakkKFVVKlkvhgkrshc<br>ccpesyspkkKFIVKlkvhgershc<br>.........................	Hs_ENSP00000344860<br>Bt<br>Rn_ENSRNOP00000051528<br>Mm_ENSMUSP00000083258<br>Gg	NP_997276 Insulin growth factor-like family member 3 Precursor

				-<br>-<br>-<br>-<br>-	.........................<br>.........................<br>.........................<br>.........................<br>.........................	Xt<br>Dr<br>Ce<br>Dm<br>Sc	
230	1	CI	ENSP00000348510<br>ENSG00000077235	1321<br>1306<br>1314<br>1315<br>1304<br>-<br>-<br>-<br>1063<br>-	ktshsvgrraRYIVKnpqay-----<br>ktslsvgrraRYIIKnpqay-----<br>ktshsvgrraRYIVKnpqaf-----<br>ktshsvgrraRYIVKnpqaf-----<br>ktshsvgrraRYIVRnpqty-----<br>.........................<br>.........................<br>.........................<br>kttqacvrrvqfmvkmkrdipdvps<br>.........................	Hs_ENSP00000348510<br>Bt_ENSBTAP00000008061<br>Rn_ENSRNOP00000022271<br>Mm_ENSMUSP00000056719<br>Gg_ENSGALP00000011931<br>Xt<br>Dr<br>Ce<br>Dm_FBpp0080077<br>Sc	NP_001511 General transcription factor 3C polypeptide 1 (Transcription factor IIIC subunit alpha)(TF3C-alpha)(TFIIIC 220 kDa subunit)(TFIIIC220)(TFIIIC box B-binding subunit)
231	1	CI	ENSP00000223608<br>ENSG00000106771	432<br>432<br>429<br>429<br>436<br>-<br>394<br>377<br>397<br>-	lapwpivglgKFLLKvdsklwhwln<br>lapwpiiglgKFLLKvdsklwhwln<br>lapwpitglgKFLLKvds-------<br>lapwpiiglgKFLLKvds-------<br>laprpirglgrvmlkldsklwhwln<br>.........................<br>llpgpikglthfllrvdtklwhwln<br>tvagplrqfvkmlfssdk-------<br>vlplclpgvlelnykint-------<br>.........................	Hs_ENSP00000223608<br>Bt_ENSBTAP00000047510<br>Rn_ENSRNOP00000035996<br>Mm_ENSMUSP00000103234<br>Gg_ENSGALP00000021416<br>Xt<br>Dr_ENSDARP00000094183<br>Ce_CE28063<br>Dm_FBpp0081280<br>Sc	Transmembrane protein C9orf5 (Protein CG-2)
232	1	CI	ENSP00000358547<br>ENSG00000130830	254<br>254<br>-<br>254<br>256<br>249<br>256<br>-<br>-<br>-	pfgkkkkykdKYLAKhssifdqldv<br>pfgkkkkykdKYLAKhsaifdqldv<br>.........................<br>pfgkkkkckdKYLAKhssifdqldv<br>pfgkkkkykdKYLAKhssifdqldv<br>pfskkkkckdKYLAKhssifdqldv<br>pfgkkkkckdKYLAKhssifdqldv<br>.........................<br>.........................<br>.........................	Hs_ENSP00000358547<br>Bt_ENSBTAP00000017340<br>Rn<br>Mm_ENSMUSP00000033775<br>Gg_ENSGALP00000008114<br>Xt_ENSXETP00000034012<br>Dr_ENSDARP00000090307<br>Ce<br>Dm<br>Sc	NP_002427 55 kDa erythrocyte membrane protein (p55)(Membrane protein, palmitoylated 1)
233	1	CI	ENSP00000269122<br>ENSG00000141367	945<br>933<br>949<br>949<br>932<br>862<br>946<br>947<br>946<br>951	nenslfkslsRYLVRrkdpelwgsv<br>nenslfkslsRYLVRrkdpelwgsv<br>nenslfkslsRYLVRrkdpelwgsv<br>nenslfkslsRYLVRrkdpelwgsv<br>nenslfkslsRYLVRrkdpelwasv<br>nenslfkslsRYLVRrkdpelwasv<br>nenslfkslsRYLVRrkdpelwasv<br>nenslfknlaRYLVKrrdftlweqv<br>nenslfksearylvgrrdaelwaev<br>nensmykyqaryllersdldlwnkv	Hs_ENSP00000269122<br>Bt_ENSBTAP00000022210<br>Rn_ENSRNOP00000052656<br>Mm_ENSMUSP00000050220<br>Gg_ENSGALP00000008236<br>Xt_ENSXETP00000023489<br>Dr_ENSDARP00000073045<br>Ce_CE00480<br>Dm_FBpp0073966<br>Sc_YGL206C	NP_004850 Clathrin heavy chain 1 (CLH-17)
234	1	C	ENSP00000261377<br>ENSG00000005189	344<br>300<br>325<br>340<br>338<br>299<br>-<br>-<br>-<br>344	eqgrrfk--lKFLAKvilgkdiqcp<br>eqgrrfk--lKFLAKailgkdiqcp<br>kqgrrfk--ltflakvilgkdiqcp<br>kqgrrfk--ltflarvilgkdiqcp<br>negrrfk--lKFLAKavlgkeiqce<br>eygrkfr--lkflaqavlgreiqtd<br>.........................<br>.........................<br>.........................<br>kagdpfkpslkylsetflnksiqng	Hs_ENSP00000261377<br>Bt_ENSBTAP00000008458<br>Rn_ENSRNOP00000019654<br>Mm_ENSMUSP00000102130<br>Gg_ENSGALP00000003498<br>Xt_ENSXETP00000036499<br>Dr<br>Ce<br>Dm<br>Sc_YGR276C	NP_112203 Putative RNA exonuclease NEF-sp (EC 3.1.-.-)
235	1	C	ENSP00000323339<br>ENSG00000168014	93<br>0<br>0<br>93<br>0<br>0<br>-<br>-<br>-<br>-	tepkavrtttRYAIRcgpkqftsyl<br>-------------------------<br>-------------------------<br>tepkavrtttrygircgpkqftsyl<br>-------------------------<br>-------------------------<br>.........................<br>.........................<br>.........................<br>.........................	Hs_ENSP00000323339<br>Bt_ENSBTAP00000003775<br>Rn_ENSRNOP00000023802<br>Mm_ENSMUSP00000062637<br>Gg_ENSGALP00000027928<br>Xt_ENSXETP00000018742<br>Dr<br>Ce<br>Dm<br>Sc	NP_056346 C2 domain-containing protein 3
236	1	CI	ENSP00000355910<br>ENSG00000042781	1986<br>1987<br>1714<br>1978<br>1992	epvvr-gvieKYILKaysedstrpp<br>epvvargvieKYILRaysedalgpp<br>epamvkgvleKYILKaysedsa-qp<br>epavvkgvleKYVLKaysedss-qp<br>kpagvigvieKYILKayeedgp---	Hs_ENSP00000355910<br>Bt_ENSBTAP00000045409<br>Rn_ENSRNOP00000031076<br>Mm_ENSMUSP00000027905<br>Gg_ENSGALP00000015712	Usherin Precursor (Usher syndrome type-2A protein)(Usher syndrome type IIa protein)

				-<br>359<br>-<br>-<br>-	.........................<br>epaevrgvieKYVLKaysrdrp--s<br>.........................<br>.........................<br>.........................	Xt<br>Dr_ENSDARP00000080636<br>Ce<br>Dm<br>Sc	
236	2	C	ENSP00000355910<br>ENSG00000042781	2213<br>2214<br>1940<br>2204<br>2216<br>-<br>581<br>-<br>-<br>-	hmlqyvlpgnKYLIKlgactgggct<br>htlqyllpgnKYLIKlaactgggct<br>hllyhlspgclyliklgactgggct<br>hvlhhlspgglylirlrvctgggct<br>ftirhlfpgteylikllaactgggcs<br>.........................<br>ytvrnlvpgstylfqiaactgggct<br>.........................<br>.........................<br>.........................	Hs_ENSP00000355910<br>Bt_ENSBTAP00000045409<br>Rn_ENSRNOP00000031076<br>Mm_ENSMUSP00000027905<br>Gg_ENSGALP00000015712<br>Xt<br>Dr_ENSDARP00000080636<br>Ce<br>Dm<br>Sc	Usherin Precursor (Usher syndrome type-2A protein)(Usher syndrome type IIa protein)
236	3	C	ENSP00000355910<br>ENSG00000042781	4388<br>3749<br>4115<br>4380<br>4396<br>-<br>2746<br>-<br>-<br>-	pptvqngkitKYLVRydnkeslagq<br>-------------------------<br>ppsvpngkiakyllhcdgeehlagq<br>ppsipngkivkyllqcdgeehlagq<br>ppqiqngditKYILKlnneeyypgk<br>.........................<br>vpsfqngeivvyilkvnndevyrgk<br>.........................<br>.........................<br>.........................	Hs_ENSP00000355910<br>Bt_ENSBTAP00000045409<br>Rn_ENSRNOP00000031076<br>Mm_ENSMUSP00000027905<br>Gg_ENSGALP00000015712<br>Xt<br>Dr_ENSDARP00000080636<br>Ce<br>Dm<br>Sc	Usherin Precursor (Usher syndrome type-2A protein)(Usher syndrome type IIa protein)
237	1	CI	ENSP00000334876<br>ENSG00000185974	34<br>34<br>34<br>34<br>-<br>-<br>-<br>-<br>-<br>-	gsssqpsrdkKYLAKlklpplskce<br>assgpasrdrKYLARlklpplskce<br>gsstpssrdkKYLAKlrlpplskce<br>gsstpssrdkKYLAKlrlpplskce<br>.........................<br>.........................<br>.........................<br>.........................<br>.........................<br>.........................	Hs_ENSP00000334876<br>Bt_ENSBTAP00000026589<br>Rn_ENSRNOP00000024999<br>Mm_ENSMUSP00000033827<br>Gg<br>Xt<br>Dr<br>Ce<br>Dm<br>Sc	NP_002920 Rhodopsin kinase Precursor (RK)(EC 2.7.11.14)(G protein-coupled receptor kinase 1)
238	1	CI	ENSP00000382104<br>ENSG00000142197	97<br>97<br>97<br>97<br>97<br>97<br>-<br>-<br>102<br>109	yeiifkivgtKWLAKdlflys---c<br>yeiifkivgtKWLAKdlflyspqsc<br>yeiifkivgtKWLAKdlflys---c<br>yeiifkivgtKWLAKdlflys---c<br>yeiifkiigtKWLAKdlflys---s<br>yeiifkiigtkclakdlflys---s<br>.........................<br>.........................<br>ysvifsktgperlatefiysa----<br>ytyifehigletlatecniwip---	Hs_ENSP00000382104<br>Bt_ENSBTAP00000019018<br>Rn_ENSRNOP00000002302<br>Mm_ENSMUSP00000044437<br>Gg_ENSGALP00000025787<br>Xt_ENSXETP00000030816<br>Dr<br>Ce<br>Dm_FBpp0085810<br>Sc_YDR141C	NP_005119 Protein dopey-2
239	1	CI	ENSP00000386616<br>ENSG00000178199	181<br>182<br>80<br>239<br>197<br>-<br>-<br>-<br>-<br>-	hgkrlvcyddRYIVKvayeqdgviv<br>sgkrvvcyddRYIVKvaydldgviv<br>ngkrvvcyddRYIVKlayekdgviv<br>ngkrvvcyddRYIVKvayekdgiiv<br>kgkrvvcyddRYIVKvayekdgviv<br>.........................<br>.........................<br>.........................<br>.........................<br>.........................	Hs_ENSP00000386616<br>Bt_ENSBTAP00000009713<br>Rn_ENSRNOP00000021463<br>Mm_ENSMUSP00000040217<br>Gg_ENSGALP00000020159<br>Xt<br>Dr<br>Ce<br>Dm<br>Sc	NP_997243 Zinc finger CCCH domain-containing protein 12D (MCP-induced protein 4)(p34)
240	1	CI	ENSP00000359234<br>ENSG00000186862	41<br>40<br>41<br>41<br>0<br>4<br>-<br>-<br>-<br>-	lgsdsgstatRYLLRkqqrllngpp<br>lgsesg-satRYLLRkq-rllngph<br>lgsdsastatRYLLRkqqrllngps<br>lgsdsgstatRYLLRkqqrllngps<br>-------------------------<br>-------tasrylfrkqnrqgnglp<br>.........................<br>.........................<br>.........................<br>.........................	Hs_ENSP00000359234<br>Bt_ENSBTAP00000032834<br>Rn_ENSRNOP00000049968<br>Mm_ENSMUSP00000075780<br>Gg_ENSGALP00000038510<br>Xt_ENSXETP00000046257<br>Dr<br>Ce<br>Dm<br>Sc	NP_079171 PDZ domain-containing protein 7
241	1	CI	ENSP00000224337<br>ENSG00000095585	450<br>453<br>494<br>451<br>546	t-------rlKYAVKvs--------<br>t-------rlKYAVKvs--------<br>tfppflllrklrvlavshqr-----<br>t-------rlKYAVKvs--------<br>t-------klKYIVRvs--------	Hs_ENSP00000224337<br>Bt_ENSBTAP00000051686<br>Rn_ENSRNOP00000019014<br>Mm_ENSMUSP00000057844<br>Gg_ENSGALP00000011282	NP_037446 B-cell linker protein (Cytoplasmic adapter protein)(B-cell adapter containing a SH2 domain protein)(B-cell adapter

				523<br>487<br>-<br>-<br>-	t-------klkqvikvp--------<br>t-------klrhavkp---------<br>.........................<br>.........................<br>.........................	Xt_ENSXETP00000026586<br>Dr_ENSDARP00000062683<br>Ce<br>Dm<br>Sc	containing a Src homology 2 domain protein)(Src homology 2 domain-containing leukocyte protein of 65 kDa)(SLP-65)
242	1	CI	ENSP00000341083<br>ENSG00000159899	655<br>655<br>655<br>655<br>-<br>-<br>-<br>-<br>-<br>-	lkssncvvdsRFVLKitdyglasfr<br>lkssncvvdsRFVLKitdyglasfr<br>lkssncvvdsRFVLKitdyglasfr<br>lkssncvvdsRFVLKitdyglasfr<br>.........................<br>.........................<br>.........................<br>.........................<br>.........................<br>.........................	Hs_ENSP00000341083<br>Bt_ENSBTAP00000015204<br>Rn_ENSRNOP00000021802<br>Mm_ENSMUSP00000030191<br>Gg<br>Xt<br>Dr<br>Ce<br>Dm<br>Sc	NP_003986 Atrial natriuretic peptide receptor B Precursor (ANP-B)(ANPRB)(GC-B)(Guanylate cyclase B)(EC 4.6.1.2)(NPR-B)(Atrial natriuretic peptide B-type receptor)
243	1	CI	ENSP00000341044<br>ENSG00000076555	821<br>637<br>822<br>813<br>658<br>702<br>605<br>-<br>-<br>629	vdveliyggvKYILKvarqsltmfv<br>vdveliyggvKYILKvarqsptmfv<br>vdveliyggiKYVLKvarqsltmfv<br>vdveliyggiKYALKvarqsltmfv<br>idveliyegtkyvlqvarqslttyv<br>vdveliservkyklkvarqslttyv<br>vnvdliydgvkyclkvarqspttyv<br>.........................<br>.........................<br>fpvdfihegkrykftvaksgndryt	Hs_ENSP00000341044<br>Bt_ENSBTAP00000029559<br>Rn_ENSRNOP00000000821<br>Mm_ENSMUSP00000099642<br>Gg_ENSGALP00000021884<br>Xt_ENSXETP00000019624<br>Dr_ENSDARP00000083511<br>Ce<br>Dm<br>Sc_YNR016C	Acetyl-CoA carboxylase 2 (EC 6.4.1.2)(ACC-beta)
244	1	CI	ENSP00000366221<br>ENSG00000130940	466<br>466<br>245<br>465<br>-<br>328<br>484<br>-<br>-<br>-	avtedvniyqKYIARfsgsqhcghi<br>avtedvniyqKYIARfsgsqhcghi<br>avtedvniyqKYIARfsgsqhcghi<br>avtedvniyqKYIARfsgsqhcghi<br>.........................<br>--pedvniyqKYIARfsgsqhcghi<br>-atedvnvyqKYIARfsgsqhcghv<br>.........................<br>.........................<br>.........................	Hs_ENSP00000366221<br>Bt_ENSBTAP00000026402<br>Rn_ENSRNOP00000018000<br>Mm_ENSMUSP00000112978<br>Gg<br>Xt_ENSXETP00000013829<br>Dr_ENSDARP00000017782<br>Ce<br>Dm<br>Sc	NP_001073312 Zinc finger protein castor homolog 1 (Castor-related protein)(Zinc finger protein 693)
245	1	C	ENSP00000366673<br>ENSG00000102805	282<br>282<br>216<br>216<br>217<br>213<br>193<br>-<br>-<br>-	etwfdsydcsKFVLRtfnklaefga<br>erwfesydcsKFVLRtyeklaelga<br>qtwfesydcsnfvlrtyeklaefgt<br>qtwfesydcsnfvlrtykklaefgt<br>vvwfdsyecsKFILRtyqkladlga<br>svwfdsydcasfvlrtyqklfelga<br>ttwfdsydcsqfvhrtykklmdlgt<br>.........................<br>.........................<br>.........................	Hs_ENSP00000366673<br>Bt_ENSBTAP00000025093<br>Rn_ENSRNOP00000013381<br>Mm_ENSMUSP00000022721<br>Gg_ENSGALP00000027298<br>Xt_ENSXETP00000033758<br>Dr_ENSDARP00000080737<br>Ce<br>Dm<br>Sc	NP_006484 Ceroid-lipofuscinosis neuronal protein 5 Precursor (Protein CLN5)
246	1	CI	ENSP00000318176<br>ENSG00000180228	187<br>187<br>187<br>187<br>-<br>-<br>-<br>-<br>-<br>-	kkqakrnaaeKFLAKfsnispenhi<br>kkqakrnaaeKFLAKfsnispenhi<br>kkqakrnaaeKFLAKfsnispenhi<br>kkqakrnaaeKFLAKfsnispenhi<br>.........................<br>.........................<br>.........................<br>.........................<br>.........................<br>.........................	Hs_ENSP00000318176<br>Bt_ENSBTAP00000001451<br>Rn_ENSRNOP00000015624<br>Mm_ENSMUSP00000002808<br>Gg<br>Xt<br>Dr<br>Ce<br>Dm<br>Sc	NP_003681 Interferon-inducible double stranded RNA-dependent protein kinase activator A (Protein kinase, interferon-inducible double stranded RNA-dependent activator)(Protein activator of the interferon-induced protein kinase)(PKR-associated protein X)(PKR-associating protein X)
247	1	CI	ENSP00000314897<br>ENSG00000091879	368<br>371<br>368<br>368<br>365<br>370<br>354<br>281<br>-<br>-	efvsqltnqqRYVLKihlkdwegne<br>efvsqvtgqkRYVLKihlrdwegne<br>efvsqltsghRYVLKiqlkdwegse<br>efvsqltgqhRYVLKiqlkdwegne<br>efvsqltnqkRYVLKiilkdwegne<br>efvhqltahgsyalriqlrdwdgne<br>eiiskltqekqhtlridlmdwegnm<br>dnmnhlsgakpyrlqidlccgtllv<br>.........................<br>.........................	Hs_ENSP00000314897<br>Bt_ENSBTAP00000014656<br>Rn_ENSRNOP00000022774<br>Mm_ENSMUSP00000033846<br>Gg_ENSGALP00000026291<br>Xt_ENSXETP00000009548<br>Dr_ENSDARP00000024418<br>Ce_CE19151<br>Dm<br>Sc	NP_001112359 Angiopoietin-2 Precursor (ANG-2)
248	1	CI	ENSP00000329632<br>ENSG00000183580	357<br>325<br>357<br>369<br>266	hcgrvtdvgiRYVAKycsklrylna<br>hcgrvtdvgiRYVAKycgklrylna<br>hcgritdvgiRYVAKycsklrylna<br>hcgritdvgiRYVAKycsklrylna<br>hcgritdvgiRYIAKycsklrylna	Hs_ENSP00000329632<br>Bt_ENSBTAP00000004177<br>Rn_ENSRNOP00000033571<br>Mm_ENSMUSP00000061305<br>Gg_ENSGALP00000021116	NP_036436 F-box/LRR-repeat protein 7 (F-box and leucine-rich repeat protein 7)(F-box protein FBL6/FBL7)

				332	hcgritdvgiRYIAKycsklrylna	Xt_ENSXETP00000016434	
				367	hcsritdvgvRYVAKycsrlrylna	Dr_ENSDARP00000084255	
				-	....	Ce	
				651	kcervsdaglkviarrcyklrylna	Dm_FBpp0082655	
				-	....	Sc	
249	1	C	ENSP00000308107<br>ENSG00000173083	477	pypfsnkqvdKYLLRp--lgphgll	Hs_ENSP00000308107	NP_006656 Heparanase Precursor (EC 3.2.-.-)(Heparanase-1)(Hpa1)(Endo-glucoronidase)
				479	phhlfnkqvdKYLIKp--sgpdgll	Bt_ENSBTAP00000007550	
				-	....	Rn	
				469	ppplfrkpvdtyllkp--sgpdgll	Mm_ENSMUSP00000044072	
				458	pkqlwsksvdqylllp--hgkdsil	Gg_ENSGALP00000018245	
				464	psslsgkfideylllp--alasdll	Xt_ENSXETP00000038863	
				478	pahltngsieafvlqsdeageqgly	Dr_ENSDARP00000087587	
				-	....	Ce	
				-	....	Dm	
				-	....	Sc	
250	1	CI	ENSP00000377355<br>ENSG00000067840	640	wkvkvrsdgtRYVAKrpvrdrllka	Hs_ENSP00000377355	PDZ domain-containing protein 4 (PDZ domain-containing RING finger protein 4-like protein)
				635	wkvkvrsdgtRYVAKrpvrdrllka	Bt_ENSBTAP00000052053	
				637	wkvkvrsdgtRYVAKrpvrdrllka	Rn_ENSRNOP00000053214	
				637	wkvkvrsdgtRYVAKrpvrdrllka	Mm_ENSMUSP00000002080	
				-	....	Gg	
				640	wkvkirsdgtryvtkrpvrdrllka	Xt_ENSXETP00000001614	
				-	....	Dr	
				-	....	Ce	
				701	wkvkrrpdgtRYIVKrpvrnrpqva	Dm_FBpp0088159	
				-	....	Sc	
251	1	C	ENSP00000263985<br>ENSG00000059691	286	rtevknlnsiRFLAKai-dyeiqrq	Hs_ENSP00000263985	NP_004555 Probable glutamyl-tRNA(Gln) amidotransferase subunit B, mitochondrial Precursor (Glu-ADT subunit B)(EC 6.3.5.-)(Cytochrome oxidase assembly factor PET112 homolog)
				286	rtevknlnsaRFLAKai-dyeiqrq	Bt_ENSBTAP00000018481	
				-	....	Rn	
				286	rtevknlnslRFLAKai-dyeiqrq	Mm_ENSMUSP00000029726	
				224	rtevkninsirfltkav-dyeiqrq	Gg_ENSGALP00000016403	
				-	....	Xt	
				221	rtevkninsvrhlakaiadyeiqrq	Dr_ENSDARP00000093298	
				245	rteiknmnsirtihtai-nyeiarq	Ce_CE39235	
				244	rtevknigsvrsisqai-tyeinrq	Dm_FBpp0083969	
				248	rvelknlpntssiinai-kyeyqrq	Sc_YBL080C	
252	1	CI	ENSP00000380651<br>ENSG00000102572	325	rptakellkhKFILRnakktsylte	Hs_ENSP00000380651	NP_001027467 Serine/threonine-protein kinase 24 (EC 2.7.11.1)(STE20-like kinase MST3)(MST-3)(Mammalian STE20-like protein kinase 3)
				262	rptakellkhKFIIRnakktsylte	Bt_ENSBTAP00000026195	
				265	rptakellkhKFIIRnakktsylte	Rn_ENSRNOP00000031981	
				272	rptakellkhKFIIRnakktsylte	Mm_ENSMUSP00000078746	
				260	rptakellkhKFILRnakktsylte	Gg_ENSGALP00000027229	
				260	rpsakellkhkfimrsakktsylte	Xt_ENSXETP00000050760	
				268	rptakellkhklivrfakktsylte	Dr_ENSDARP00000095575	
				-	....	Ce	
				261	rptakellkypfikk-akknaylid	Dm_FBpp0082840	
				270	rpsaynllsfefvknititn--lks	Sc_YDR523C	
253	1	C	ENSP00000315137<br>ENSG00000163082	78	raaapeayvqKYVVKnyfyyylfqf	Hs_ENSP00000315137	NP_689599 Sphingosine-1-phosphate phosphatase 2 (SPPase2)(Spp2)(Sphingosine-1-phosphatase 2)(hSPP2)(EC 3.1.3.-)
				-	....	Bt	
				6	-----qasgekhvvknyfyyylfrf	Rn_ENSRNOP00000018882	
				33	-----easgeehvvknyfyyylfrf	Mm_ENSMUSP00000036656	
				5	------gcvqKYVVKnyfyyylfkf	Gg_ENSGALP00000008413	
				-	....	Xt	
				-	....	Dr	
				-	....	Ce	
				-	....	Dm	
				71	dnqsdfvhslqkkhrtpfrdvyfky	Sc_YKR053C	
254	1	CI	ENSP00000353643<br>ENSG00000123562	261	lllgylhdflKYLAKnsaslftasd	Hs_ENSP00000353643	NP_036418 Mortality factor 4-like protein 2 (MORF-related gene X protein)(Transcription factor-like protein MRGX)(MSL3-2 protein)
				260	lllgylhdflKYLAKnaaslftasd	Bt_ENSBTAP00000001731	
				261	lllgylhdflKYLAKnsaslftasd	Rn_ENSRNOP00000003247	
				261	lllgylhdflKYLAKnsaslftasd	Mm_ENSMUSP00000108720	
				-	....	Gg	
				-	....	Xt	
				-	....	Dr	
				-	....	Ce	
				-	....	Dm	
				-	....	Sc	
255	1	C	ENSP00000216951<br>ENSG00000100983	360	aiaealaapsRFVLKpqregggnnl	Hs_ENSP00000216951	NP_000169 Glutathione synthetase (EC 6.3.2.3)(Glutathione synthase)(GSH synthetase)(GSH-S)
				360	aitkaiaapscfvlkpqregggnnl	Bt_ENSBTAP00000004559	
				360	avaealaapshfvlkpqregggnnf	Rn_ENSRNOP00000025657	
				360	aiaealaapshfvlkpqregggnnl	Mm_ENSMUSP00000078630	
				318	iaataiaspeRFVLKpqregggnnl	Gg_ENSGALP00000005180	

				357<br>364<br>375<br>367<br>378	avkaalanpdqfvlkpqregggnnl<br>tvamalanpdqyvlkpqregggnni<br>vvagaqkhpeafvlkpqteggaalh<br>syemalrtpeRFVLKpqregggnnv<br>gkrlalsepsKYVLKpqregggnnv	Xt_ENSXETP00000016282<br>Dr_ENSDARP00000054908<br>Ce_CE18087<br>Dm_FBpp0088741<br>Sc_YOL049W	
256	1	CI	ENSP00000370625<br>ENSG00000047315	83<br>84<br>76<br>83<br>84<br>83<br>-<br>90<br>84<br>87	hasgeveeppRYLLKfeqiylskpt<br>hasgeveeppRYLLKfeqiylskpt<br>hasgeveeppRYLLKfeqiylskpt<br>hasgeveeppRYLLKfeqiylskpt<br>hatgeveeppRYLLKfeqiylskpt<br>httgeveeppRYLLKfeqiylskpt<br>.........................<br>hlgtdmenpakfslkfnqiylskpt<br>htsgevetpprfslkfeqiylskpt<br>httesdnisrkyeisfgkiyvtkpm	Hs_ENSP00000370625<br>Bt_ENSBTAP00000025800<br>Rn_ENSRNOP00000030781<br>Mm_ENSMUSP00000031167<br>Gg_ENSGALP00000018549<br>Xt_ENSXETP00000011733<br>Dr<br>Ce_CE01162<br>Dm_FBpp0082353<br>Sc_YOR151C	NP_000929 DNA-directed RNA polymerase II subunit RPB2 (EC 2.7.7.6)(DNA-directed RNA polymerase II subunit B)(RNA polymerase II subunit B2)(RNA polymerase II subunit 2)(DNA-directed RNA polymerase II 140 kDa polypeptide)
257	1	C	ENSP00000368460<br>ENSG00000067992	5<br>5<br>5<br>5<br>5<br>5<br>5<br>-<br>5<br>-	------mrlfRWLLKq------pvp<br>------mrlfrlllkq------pvp<br>------mrlfyrllkq------pvp<br>------mrlfyrllkq------pvp<br>------mrlcgallks------pip<br>------mrlcRFLLKn------tvp<br>------mrlyafllqn------hlp<br>.........................<br>------mrlfpvrfsaasssmasla<br>.........................	Hs_ENSP00000368460<br>Bt_ENSBTAP00000023706<br>Rn_ENSRNOP00000017370<br>Mm_ENSMUSP00000036604<br>Gg_ENSGALP00000026284<br>Xt_ENSXETP00000047175<br>Dr_ENSDARP00000015430<br>Ce<br>Dm_FBpp0111805<br>Sc	NP_005382 Unknown
258	1	CI	ENSP00000264639<br>ENSG00000108344	92<br>92<br>88<br>88<br>76<br>58<br>61<br>52<br>55<br>51	lekavsgkepRFVLRalrmlpstsr<br>lekavsgkepRFVLRalrmlpstsr<br>lekavsgkepRFVLRalrmlpstsr<br>lekavsgkepRFVLRalrmlpstsr<br>lekavagkepRYVLRalralpstsr<br>ieravsgkepRFVLRalralpstsr<br>iekavsgkepRFVLRalralpstsr<br>ldkg----eehlitrvlqvlpktrk<br>iekgvaskesRFILRvlrnlpntrr<br>iskttltldpryiwrslkdlsslrn	Hs_ENSP00000264639<br>Bt_ENSBTAP00000028608<br>Rn_ENSRNOP00000037928<br>Mm_ENSMUSP00000017365<br>Gg_ENSGALP00000021953<br>Xt_ENSXETP00000040156<br>Dr_ENSDARP00000092860<br>Ce_CE00101<br>Dm_FBpp0080691<br>Sc_YER021W	NP_002800 26S proteasome non-ATPase regulatory subunit 3 (26S proteasome regulatory subunit S3) (Proteasome subunit p58)
259	1	CI	ENSP00000360268<br>ENSG00000059573	761<br>761<br>761<br>761<br>774<br>781<br>729<br>757<br>747<br>422	pvglegllttKWLLRgkdhvvsdfs<br>pvglegllttKWLLRgqdhvvsdfs<br>pvglegllttKWLLRgqdhvvsdfs<br>pvglegllttKWLLRgqdhvvsdfs<br>pvgiegllttKWLLRgdnhvvsdfs<br>pvgiegllttKWILRgenhvvsdfs<br>pvglegllttKWVLRgeghtvadfs<br>pvgvegllttKWLLRgeghlvedfk<br>pvgvegllttkwilegqdhaaadfa<br>pvgldglvsyqyqirgdgqvasdyl	Hs_ENSP00000360268<br>Bt_ENSBTAP00000015703<br>Rn_ENSRNOP00000020940<br>Mm_ENSMUSP00000025979<br>Gg_ENSGALP00000017112<br>Xt_ENSXETP00000048928<br>Dr_ENSDARP00000081244<br>Ce_CE26824<br>Dm_FBpp0078130<br>Sc_YOR323C	NP_002851 Delta-1-pyrroline-5-carboxylate synthetase (P5CS) (Aldehyde dehydrogenase family 18 member A1)
260	1	CI	ENSP00000258787<br>ENSG00000136286	646<br>647<br>716<br>652<br>-<br>-<br>-<br>-<br>-<br>-	agfasrqpysRFLLRykmtceytwp<br>agfasrqpyaRFLLRykmtceytwp<br>agfasrqpypRFLLRykmtceytwp<br>agfasrqpypRFLLRykmtceytwp<br>.........................<br>.........................<br>.........................<br>.........................<br>.........................<br>.........................	Hs_ENSP00000258787<br>Bt_ENSBTAP00000008366<br>Rn_ENSRNOP00000010021<br>Mm_ENSMUSP00000003459<br>Gg<br>Xt<br>Dr<br>Ce<br>Dm<br>Sc	NP_149043 Myosin-Ig
261	1	CI	ENSP00000371352<br>ENSG00000132972	1143<br>1124<br>1113<br>1113<br>962<br>-<br>-<br>-<br>-<br>-	dekgervdvsKYLIKkglalrerri<br>dekgervdvsKYLIKkglalretri<br>dekgervdvsKYLIKkglalrdrrv<br>dekgervdvsKYLIKkglalrerrv<br>deagrlidasehliekglafrntrt<br>.........................<br>.........................<br>.........................<br>.........................<br>.........................	Hs_ENSP00000371352<br>Bt_ENSBTAP00000000345<br>Rn_ENSRNOP00000010795<br>Mm_ENSMUSP00000093469<br>Gg_ENSGALP00000027645<br>Xt<br>Dr<br>Ce<br>Dm<br>Sc	RING finger protein 17 (Tudor domain-containing protein 4)
262	1	CI	ENSP00000350686<br>ENSG00000176915	29<br>29<br>29<br>29<br>28	lgasvlliavRWLVRrlgprpgglg<br>lgasvlliavRWLVRrldrrprglg<br>lgasvlliavRWLVRrlenlsrdpn<br>lgasvlfiavRWLVRrlekrprdln<br>lvacaligavgwllrlldgrtggrg	Hs_ENSP00000350686<br>Bt_ENSBTAP00000000786<br>Rn_ENSRNOP00000053507<br>Mm_ENSMUSP00000031474<br>Gg_ENSGALP00000003353	NP_055929 Ankyrin repeat and LEM domain-containing protein 2

				0<br>0<br>-<br>0<br>-	-------------------------<br>-------------------------<br>.........................<br>-------------------------<br>.........................	Xt_ENSXETP00000051395<br>Dr_ENSDARP00000084954<br>Ce<br>Dm_FBpp0074262<br>Sc	
263	1	CI	ENSP00000187397<br>ENSG00000172995	249<br>88<br>248<br>248<br>256<br>-<br>-<br>-<br>-<br>-	kdekgeesqkRFILKrdnssidked<br>kdekgeesqkRFILKrdnssidked<br>kdekseesqkRFILKrdnssidked<br>kdekseesqkRFILKrdnssidked<br>kdekgeesqkRFILKrdnssidked<br>.........................<br>.........................<br>.........................<br>.........................<br>.........................	Hs_ENSP00000187397<br>Bt_ENSBTAP00000010173<br>Rn_ENSRNOP00000011870<br>Mm_ENSMUSP00000107503<br>Gg_ENSGALP00000019650<br>Xt<br>Dr<br>Ce<br>Dm<br>Sc	NP_057384 cAMP-regulated phosphoprotein 21 (ARPP-21) (Thymocyte cAMP-regulated phosphoprotein)
264	1	CI	ENSP00000313643<br>ENSG00000181085	12<br>13<br>13<br>13<br>13<br>13<br>13<br>12<br>-<br>-	t-vvdprivrRYLLRrqlgqgaygi<br>taevdrhvaqRYLLKrrlgkgaygi<br>aaevdrhvsqRYLIKrrlgkgaygi<br>aaevdrhvaqRYLIKrrlgkgaygi<br>epevdaavaekfemkrrlgkgaygi<br>gpevedhisqkyeikkrlgkgaygi<br>iteveehisskyeikrrlgkgaygi<br>tddvdthihekfdlqkrlgkgaygi<br>.........................<br>.........................	Hs_ENSP00000313643<br>Bt_ENSBTAP00000026467<br>Rn_ENSRNOP00000012460<br>Mm_ENSMUSP00000087098<br>Gg_ENSGALP00000022458<br>Xt_ENSXETP00000029384<br>Dr_ENSDARP00000088451<br>Ce_CE29020<br>Dm<br>Sc	Mitogen-activated protein kinase 15 (EC 2.7.11.24)(Extracellular signal-regulated kinase 8)
265	1	CI	ENSP00000355060<br>ENSG00000074964	596<br>551<br>-<br>594<br>590<br>561<br>-<br>617<br>-<br>-	eisslvplgpKYVVKwntalpqvqv<br>eisslaplgpKYVVKwstalpqvqv<br>.........................<br>eisslvplgpKYVVKwntalpqvqv<br>eisslvplgpKYVVKwstalpqvqv<br>dignlvplgpRYVLKwntplaqiyv<br>.........................<br>--kvlyskmssqsmgflegnrfkll<br>.........................<br>.........................	Hs_ENSP00000355060<br>Bt_ENSBTAP00000000909<br>Rn<br>Mm_ENSMUSP00000101425<br>Gg_ENSGALP00000039879<br>Xt_ENSXETP00000032387<br>Dr<br>Ce_CE39517<br>Dm<br>Sc	NP_060595 Rho guanine nucleotide exchange factor 10-like protein (GrinchGEF)
266	1	CI	ENSP00000369198<br>ENSG00000173698	733<br>691<br>729<br>725<br>624<br>-<br>-<br>-<br>-<br>-	lvkvfntyirKYILKfcivgwgvpa<br>lvkvfntyirKYILKfcifgwgipa<br>lvkvfntyirKYILKfcivgwgipa<br>lvkvfntyirKYILKfcivgwgipa<br>lvkvfntyvrKYILKfcivgwglpa<br>.........................<br>.........................<br>.........................<br>.........................<br>.........................	Hs_ENSP00000369198<br>Bt_ENSBTAP00000000756<br>Rn_ENSRNOP00000039239<br>Mm_ENSMUSP00000108027<br>Gg_ENSGALP00000026589<br>Xt<br>Dr<br>Ce<br>Dm<br>Sc	NP_001073327 G-protein coupled receptor 64 Precursor (Epididymis-specific protein 6) (He6 receptor)
267	1	CI	ENSP00000372860<br>ENSG00000206375	435<br>1031<br>435<br>435<br>-<br>-<br>-<br>-<br>-<br>-	lcgdwlqglhRFVARekimsvlsew<br>lcedwlqglhRFVARekilsalrer<br>lcgdwlqglhRFVARekimctlrer<br>lcgdwlqglhRFVARekimctlreq<br>.........................<br>.........................<br>.........................<br>.........................<br>.........................<br>.........................	Hs_ENSP00000372860<br>Bt_ENSBTAP00000014160<br>Rn_ENSRNOP00000046565<br>Mm_ENSMUSP00000047917<br>Gg<br>Xt<br>Dr<br>Ce<br>Dm<br>Sc	NP_065175 Valyl-tRNA synthetase, mitochondrial Precursor (EC 6.1.1.9)(Valine--tRNA ligase)(ValRS)(Valyl-tRNA synthetase-like)
267	2	I	ENSP00000372860<br>ENSG00000206375	754<br>1351<br>755<br>755<br>-<br>-<br>-<br>-<br>-<br>-	hfcnkiwnalrfilnalgekfvpqp<br>hfcnkiwnalrfilnalgeefipqp<br>hfcnklwnalRFILRalgddfvpqp<br>hfcnklwnalRFVLRalgdnfvpqp<br>.........................<br>.........................<br>.........................<br>.........................<br>.........................<br>.........................	Hs_ENSP00000372860<br>Bt_ENSBTAP00000014160<br>Rn_ENSRNOP00000046565<br>Mm_ENSMUSP00000047917<br>Gg<br>Xt<br>Dr<br>Ce<br>Dm<br>Sc	NP_065175 Valyl-tRNA synthetase, mitochondrial Precursor (EC 6.1.1.9)(Valine--tRNA ligase)(ValRS)(Valyl-tRNA synthetase-like)
268	1	CI	ENSP00000297534<br>ENSG00000164898	55<br>36<br>36<br>36<br>33	grpyrdtaayRYLVKafrahrvtse<br>grpyrdtaayRYLVKafrahrvtse<br>grpyrdtaayRYLVKafrahrvtse<br>grpyrdtaayRYLVKafrahrvtse<br>grayrdtaayrhvlaafrahrvtse	Hs_ENSP00000297534<br>Bt_ENSBTAP00000008919<br>Rn_ENSRNOP00000007544<br>Mm_ENSMUSP00000019833<br>Gg_ENSGALP00000019317	NP_932068 UPF0562 protein C7orf55

				-<br>-<br>37<br>30<br>-	.........................<br>.........................<br>ktfspnsaqykylmeqmkadqvttr<br>ngcikdslaaryilaqykkfatteq<br>.........................	Xt<br>Dr<br>Ce_CE30619<br>Dm_FBpp0110114<br>Sc	
269	1	CI	ENSP00000320675<br>ENSG00000100628	240<br>288<br>288<br>288<br>299<br>182<br>-<br>-<br>-<br>-	aaqsgqlealRFLAKygadintqas<br>aaqsgqlealRFLAKygadintqas<br>aaqsgqlealRFLAKhgadintqas<br>aaqsgqlealRFLAKhgadintqas<br>aaesgqlealRYLAKcgadintqas<br>aaqsgqmealRYIAKcgadintqan<br>.........................<br>.........................<br>.........................<br>.........................	Hs_ENSP00000320675<br>Bt_ENSBTAP00000038371<br>Rn_ENSRNOP00000012045<br>Mm_ENSMUSP00000021617<br>Gg_ENSGALP00000017666<br>Xt_ENSXETP00000022701<br>Dr<br>Ce<br>Dm<br>Sc	NP_057234 Ankyrin repeat and SOCS box protein 2 (ASB-2)
270	1	CI	ENSP00000297537<br>ENSG00000164900	347<br>312<br>-<br>402<br>-<br>-<br>325<br>-<br>-<br>-	ivvpipvhvnRFAVRsqhqqmeqga<br>ivvpipvhvnRFAVRsqhqqmeqga<br>.........................<br>ivvpipvhvnRFAVRsqhqqmeqga<br>.........................<br>.........................<br>ivvpipvhvnRFAVRsqhqqiepgs<br>.........................<br>.........................<br>.........................	Hs_ENSP00000297537<br>Bt_ENSBTAP00000014768<br>Rn<br>Mm_ENSMUSP00000085651<br>Gg<br>Xt<br>Dr_ENSDARP00000096349<br>Ce<br>Dm<br>Sc	NP_001092304 Homeobox protein GBX-1 (Gastrulation and brain-specific homeobox protein 1)
271	1	CI	ENSP00000331310<br>ENSG00000185787	335<br>296<br>336<br>335<br>275<br>296<br>296<br>299<br>397<br>364	lllnylhdflKYLAKnsatlfsasd<br>lllnylhdflKYLAKnsatlfsasd<br>lllnylhdflKYLAKnsatlfsasd<br>lllnylhdflKYLAKnsatlfsasd<br>-------------------------<br>lllnylhdflKYLAKnssllfsasd<br>lllsylqdflKYLVKnssslfsasd<br>rimigvhdlivflnknhgkyyrgss<br>nllthvqdflKFLVKnssiffsmsn<br>llikqtedflvwllmhvdeyfndkd	Hs_ENSP00000331310<br>Bt_ENSBTAP00000038563<br>Rn_ENSRNOP00000019365<br>Mm_ENSMUSP00000082346<br>Gg_ENSGALP00000032976<br>Xt_ENSXETP00000017126<br>Dr_ENSDARP00000060318<br>Ce_CE41465<br>Dm_FBpp0082579<br>Sc_YPR023C	NP_996670 Mortality factor 4-like protein 1 (MORF-related gene 15 protein)(Transcription factor-like protein MRG15)(MSL3-1 protein)
272	1	CI	ENSP00000362095<br>ENSG00000102359	254<br>-<br>255<br>298<br>258<br>250<br>196<br>-<br>-<br>-	ydraynrascKFIVKvqvrrcptlk<br>.........................<br>ydraynrascKFIVKvqvrrcpilk<br>ydraynrascKFIVKvqvrrcpilk<br>ydqpynrasckfrirvqvrrcpvlk<br>ydrahnrascKFIVKvqvrrcpdlt<br>ydqagnmatckfnvhvevrrcpklk<br>.........................<br>.........................<br>.........................	Hs_ENSP00000362095<br>Bt<br>Rn_ENSRNOP00000005020<br>Mm_ENSMUSP00000108929<br>Gg_ENSGALP00000010977<br>Xt_ENSXETP00000014699<br>Dr_ENSDARP00000045808<br>Ce<br>Dm<br>Sc	NP_055282 Sushi repeat-containing protein SRPX2 Precursor
273	1	CI	ENSP00000366939<br>ENSG00000125871	320<br>321<br>317<br>317<br>330<br>330<br>323<br>256<br>-<br>-	daelcsqywtKWLLRleeytekkkn<br>daelcsqywaKWLLRleeytkkekn<br>deelcskywaKWLLRleeytekqkn<br>deelcskywaKWLLRleeytekqkn<br>dpdlcsqywnKWLLRleeymdrn--<br>dtdlclhfwnKWLLRleeykkkkee<br>nskqikqfweKWLLRleefaek---<br>sedemmrtleevteklnefwckvts<br>.........................<br>.........................	Hs_ENSP00000366939<br>Bt_ENSBTAP00000004442<br>Rn_ENSRNOP00000034141<br>Mm_ENSMUSP00000105655<br>Gg_ENSGALP00000014141<br>Xt_ENSXETP00000030226<br>Dr_ENSDARP00000006549<br>Ce_CE41984<br>Dm<br>Sc	NP_443097 Uncharacterized protein C20orf72
274	1	CI	ENSP00000242753<br>ENSG00000123119	133<br>-<br>133<br>133<br>91<br>133<br>130<br>-<br>-<br>-	easnleqfvtRFLLKetlnqlqslq<br>.........................<br>easnleqfvtRFLLKetlnqlqslq<br>easnleqfvtRFLLKetlnqlqslq<br>easnleqfvtRFLLKetlnqlqslq<br>naskleqfvtRFLLKetlnqlqslq<br>eathlqqfvtRFLLKettsqlhslq<br>.........................<br>.........................<br>.........................	Hs_ENSP00000242753<br>Bt<br>Rn_ENSRNOP00000009718<br>Mm_ENSMUSP00000038165<br>Gg_ENSGALP00000025609<br>Xt_ENSXETP00000031947<br>Dr_ENSDARP00000034416<br>Ce<br>Dm<br>Sc	NP_071746 N-terminal EF-hand calcium-binding protein 1 (EF-hand calcium-binding protein 1)(Neuronal calcium-binding protein 1)
275	1	CI	ENSP00000339928<br>ENSG00000132437	443<br>443<br>443<br>443<br>443	ihlvpchlrdKFVLRfaicsrtves<br>ihlvpcslrdRFVLRfaicsrtvel<br>ihlvpcrlrdKFVLRfavcsrtves<br>ihlvpcrlrdKFVLRfavcartves<br>ihlvpchlreKFVLRfaicsrtves	Hs_ENSP00000339928<br>Bt_ENSBTAP00000051814<br>Rn_ENSRNOP00000005851<br>Mm_ENSMUSP00000068525<br>Gg_ENSGALP00000021345	Aromatic-L-amino-acid decarboxylase (AADC)(EC 4.1.1.28)(DOPA decarboxylase)(DDC)

				447<br>443<br>518<br>475<br>-	ihivpcclgdtfvlrfavcartves<br>ihlvpchlaglfvlrfavcarates<br>ihmvpaslgdRFVIRfcvcaenatd<br>ihlvpakikdvyflrmaicsrftqs<br>.........................	Xt_ENSXETP00000010839<br>Dr_ENSDARP00000005382<br>Ce_CE28344<br>Dm_FBpp0080710<br>Sc	
276	1	CI	ENSP00000280481<br>ENSG00000150893	688<br>617<br>675<br>677<br>694<br>310<br>-<br>-<br>-<br>-	hdppnqsglqRFVIRihpvdrlppe<br>hdppnksglqrfmirihpvdrlppe<br>qdppnqsgiqRFVIRihpvdrlppe<br>hdppnqsgiqRFVIRihpvdrlppe<br>ndppnrsgpqtfvirvhpvdrlape<br>hdppnqsdeqvfmvrieplddippe<br>.........................<br>.........................<br>.........................<br>.........................	Hs_ENSP00000280481<br>Bt_ENSBTAP00000022647<br>Rn_ENSRNOP00000031592<br>Mm_ENSMUSP00000088670<br>Gg_ENSGALP00000027482<br>Xt_ENSXETP00000038214<br>Dr<br>Ce<br>Dm<br>Sc	NP_997244 FRAS1-related extracellular matrix protein 2 Precursor (ECM3 homolog)
277	1	CI	ENSP00000219476<br>ENSG00000103197	1739<br>1721<br>1741<br>1747<br>1766<br>1749<br>-<br>-<br>1793<br>-	rsnptdiypsKWIARlrhikrlrqr<br>rsnptdiypsKWIARlrhikrlrqr<br>rsnptdiypsKWIARlrhikrlrqr<br>rsnptdiypsKWIARlrhikrlrqr<br>rsnptdtypsKWIARlrhikrlrhr<br>rsnptdiypsKWIARlrhikkmrpk<br>.........................<br>.........................<br>-vqkkhpyasnwlerlrklkrlrsk<br>.........................	Hs_ENSP00000219476<br>Bt_ENSBTAP00000046373<br>Rn_ENSRNOP00000016221<br>Mm_ENSMUSP00000085764<br>Gg_ENSGALP00000009042<br>Xt_ENSXETP00000034777<br>Dr<br>Ce<br>Dm_FBpp0074588<br>Sc	NP_000539 Tuberin (Tuberous sclerosis 2 protein)
278	1	C	ENSP00000321737<br>ENSG00000179832	1555<br>1525<br>1144<br>1144<br>-<br>958<br>1500<br>1640<br>1617<br>-	dpqatvasacRFALRmcgpnlacee<br>dphapvvtacRFALRmcgpnlecee<br>-------------------------<br>-------------------------<br>.........................<br>-------------------------<br>dpnphvvkackyamricapvvgseq<br>ddfeevrqncansiyrlhglltspn<br>esevaiasacrgtlqrvcrlltapr<br>.........................	Hs_ENSP00000321737<br>Bt_ENSBTAP00000037251<br>Rn_ENSRNOP00000049974<br>Mm_ENSMUSP00000094115<br>Gg<br>Xt_ENSXETP00000012529<br>Dr_ENSDARP00000023371<br>Ce_CE30431<br>Dm_FBpp0071261<br>Sc	NP_115826 HEAT repeat-containing protein KIAA1833
279	1	CI	ENSP00000363193<br>ENSG00000184634	1280<br>1290<br>1249<br>1281<br>1274<br>-<br>1133<br>1689<br>-<br>-	-----ldvyaKYVLRsicqqewvge<br>-----ldvyaKYVLRsicqqewvge<br>-----ldvyaKYVLRsicqqewvge<br>-----ldvyaKYVLRsicqqewvge<br>-----ldiyaKYVLRsicqqewvge<br>.........................<br>-----lvvyaKYVLKsichqewvge<br>yrpqylfniaktvqfviceqdwvtl<br>.........................<br>.........................	Hs_ENSP00000363193<br>Bt_ENSBTAP00000028464<br>Rn_ENSRNOP00000048013<br>Mm_ENSMUSP00000085260<br>Gg_ENSGALP00000009011<br>Xt<br>Dr_ENSDARP00000073783<br>Ce_CE16058<br>Dm<br>Sc	NP_005111 Mediator of RNA polymerase II transcription subunit 12 (Mediator complex subunit 12)(Thyroid hormone receptor-associated protein complex 230 kDa component)(Trap230)(Activator-recruited cofactor 240 kDa component)(ARC240)(CAG repeat protein 45)(OPA-containing protein)(Trinucleotide repeat-containing gene 11 protein)
280	1	CI	ENSP00000267116<br>ENSG00000139645	223<br>223<br>223<br>223<br>223<br>223<br>223<br>-<br>-<br>-	aaasgqievvKYLLRmgaeidepna<br>aaasgqievvKYLLRmgaeidepna<br>aaasgqievvKYLLRmgaeidepna<br>aaasgqievvkhllrmgaeidepna<br>aaasgqievvrhllrlgveidepns<br>aassgqidvvKYLLKlgveidepna<br>aaasghvdvvKYLLRngaeidepna<br>.........................<br>.........................<br>.........................	Hs_ENSP00000267116<br>Bt_ENSBTAP00000015626<br>Rn_ENSRNOP00000045243<br>Mm_ENSMUSP00000014642<br>Gg_ENSGALP00000023054<br>Xt_ENSXETP00000020976<br>Dr_ENSDARP00000030948<br>Ce<br>Dm<br>Sc	NP_775866 Ankyrin repeat domain-containing protein 52
281	1	CI	ENSP00000288235<br>ENSG00000157483	708<br>708<br>709<br>708<br>697<br>715<br>708<br>706<br>-<br>731	yarviqkswrKFVAR----------<br>yarviqkswrKFVAR----------<br>yarviqktwrKFVAR----------<br>yarviqktwrKFVAR----------<br>yaraiqkawrkyaar----------<br>yarsiqkawrKYVAR----------<br>yartiqtawrkycar----------<br>yarviqkawrqfsar----------<br>.........................<br>maariqrawrrflqrridaatkiqr	Hs_ENSP00000288235<br>Bt_ENSBTAP00000028702<br>Rn_ENSRNOP00000016696<br>Mm_ENSMUSP00000034745<br>Gg_ENSGALP00000038539<br>Xt_ENSXETP00000035798<br>Dr_ENSDARP00000052538<br>Ce_CE05763<br>Dm<br>Sc_YMR109W	NP_004989 Myosin-Ie (Myosin-Ic)
282	1	CI	ENSP00000367629<br>ENSG00000130762	500<br>499	vkslplisasRWLLKrgelflv--e<br>vkslplisasRWLLKrgelfvv--e	Hs_ENSP00000367629<br>Bt_ENSBTAP00000027769	NP_055263 Rho guanine nucleotide exchange factor 16

				-<br>504<br>319<br>-<br>-<br>-<br>-<br>-	.........................<br>vkslplisasRWLLKrgelfll--e<br>kkpfplisasRWLLKrgelyllsse<br>.........................<br>.........................<br>.........................<br>.........................<br>.........................	Rn<br>Mm_ENSMUSP00000030898<br>Gg_ENSGALP00000001583<br>Xt<br>Dr<br>Ce<br>Dm<br>Sc	
283	1	CI	ENSP00000264972<br>ENSG00000115085	186<br>186<br>186<br>186<br>186<br>186<br>186<br>-<br>321<br>-	------qtdgKFLLRprke--qgty<br>------qtdgKFLLRprke--pgty<br>------qtdgKFLLRprke--qgty<br>------qtdgKFLLRprke--qgty<br>------qpdgKFLLRdkke--ngay<br>------qpdgkflqkqwre--ravk<br>------qpdgKFLVRerde--mgtf<br>.........................<br>akqpevdtsgcflvrysespaasgl<br>.........................	Hs_ENSP00000264972<br>Bt_ENSBTAP00000007743<br>Rn_ENSRNOP00000023036<br>Mm_ENSMUSP00000027291<br>Gg_ENSGALP00000002252<br>Xt_ENSXETP00000020939<br>Dr_ENSDARP00000006727<br>Ce<br>Dm_FBpp0086382<br>Sc	NP_001070 Tyrosine-protein kinase ZAP-70 (EC 2.7.10.2)(70 kDa zeta-associated protein)(Syk-related tyrosine kinase)
284	1	CI	ENSP00000378303<br>ENSG00000126653	538<br>538<br>527<br>522<br>523<br>-<br>496<br>378<br>377<br>-	neetvmsardRYLARqmarvnakty<br>neetvtsardRYLARqmarinakty<br>neetvmsardRYLARqmarinakty<br>neetvmsardRYLARqmarinakty<br>neetvmsardRYLARqmarvgaksy<br>.........................<br>sdqtvssareRYLARqlarfasksy<br>teedieaarqryferkeqgivvppl<br>vgevlaaalaryeerkrarlq----<br>.........................	Hs_ENSP00000378303<br>Bt_ENSBTAP00000025760<br>Rn_ENSRNOP00000036224<br>Mm_ENSMUSP00000099552<br>Gg_ENSGALP00000006641<br>Xt<br>Dr_ENSDARP00000013430<br>Ce_CE01497<br>Dm_FBpp0073597<br>Sc	Coiled-coil domain-containing protein 55
285	1	CI	ENSP00000370968<br>ENSG00000132382	71<br>75<br>69<br>69<br>62<br>-<br>0<br>-<br>76<br>45	--pkgs--emKYALKrlitglgvgr<br>--pkgss-emKYALKrlitglgggr<br>--psds--emKYALKrlitglgvgr<br>--pnds--emKYALKrlitglgvgr<br>--gede--elKYALRrlveglgatr<br>.........................<br>-------------------------<br>.........................<br>nlkvsqinkavfvvfkkmqgsqltk<br>dlpddae-ewsyvlnrlikglssdr	Hs_ENSP00000370968<br>Bt_ENSBTAP00000009771<br>Rn_ENSRNOP00000021134<br>Mm_ENSMUSP00000044827<br>Gg_ENSGALP00000002078<br>Xt<br>Dr_ENSDARP00000028271<br>Ce<br>Dm_FBpp0070114<br>Sc_YEL055C	NP_001099008 Myb-binding protein 1A
286	1	CI	ENSP00000254942<br>ENSG00000132604	54<br>54<br>54<br>99<br>-<br>-<br>16<br>-<br>-<br>-	gearleeavnRWVLKfyfhealraf<br>gevrleeavnRWVLKfyfhealraf<br>gearleeavnRWVLKfyfhealraf<br>gearleeavnRWVLKfyfhealraf<br>.........................<br>.........................<br>cepsweqivnrwsfdfyafqafsaf<br>.........................<br>.........................<br>.........................	Hs_ENSP00000254942<br>Bt_ENSBTAP00000010016<br>Rn_ENSRNOP00000027695<br>Mm_ENSMUSP00000065586<br>Gg<br>Xt<br>Dr_ENSDARP00000057422<br>Ce<br>Dm<br>Sc	NP_005643 Telomeric repeat-binding factor 2 (TTAGGG repeat-binding factor 2)(Telomeric DNA-binding protein)
287	1	CI	ENSP00000250024<br>ENSG00000129173	124<br>124<br>0<br>124<br>98<br>75<br>-<br>-<br>-<br>-	kekslgllchKFLARypnypnpavn<br>kekslgllchKFLARypnypnpavn<br>-------------------------<br>kekslgllchKFLARypkypnpavn<br>kekslgllchKFLARypdypsaves<br>kekslgllchKFLARypsypnpavn<br>.........................<br>.........................<br>.........................<br>.........................	Hs_ENSP00000250024<br>Bt_ENSBTAP00000023190<br>Rn_ENSRNOP00000036763<br>Mm_ENSMUSP00000112883<br>Gg_ENSGALP00000006396<br>Xt_ENSXETP00000009660<br>Dr<br>Ce<br>Dm<br>Sc	NP_078956 Transcription factor E2F8 (E2F-8)
288	1	CI	ENSP00000323527<br>ENSG00000128585	636<br>637<br>636<br>636<br>636<br>628<br>630<br>-<br>688<br>-	pskdyllrhcKYLIRkhrfeekaqv<br>pskdyllrhcKYLIRkhrfeekaqm<br>pskdyllrhcKYLIRkhrfeekaqm<br>pskdyllrhcKYLIRkhrfeekaqm<br>pskeyllrhcKYLIRkhrfeekaqt<br>pskeyllrhcKYLIRkhrfeeraqt<br>pskeyllrhcKYLIRkyrfeekaqt<br>.........................<br>pkreeilkhcRFLVRklryeemtrq<br>.........................	Hs_ENSP00000323527<br>Bt_ENSBTAP00000013788<br>Rn_ENSRNOP00000016633<br>Mm_ENSMUSP00000026699<br>Gg_ENSGALP00000010260<br>Xt_ENSXETP00000039414<br>Dr_ENSDARP00000033515<br>Ce<br>Dm_FBpp0086998<br>Sc	NP_037387 Muskelin
289	1	CI	ENSP00000220420<br>ENSG00000104055	114<br>114	lcapptaavgRYLLKihidsfqgsv<br>lcappmaavgRYLLKvhikslqgpv	Hs_ENSP00000220420<br>Bt_ENSBTAP00000042850	NP_963925 Protein-glutamine gamma-glutamyltransferase 5 (EC

				112<br>150<br>-<br>119<br>-<br>-<br>-<br>-	lcappmaavgRYLLKvridsyqgsv<br>lcappiaavgRYLLKiridsyqgfv<br>.........................<br>mlsphtaiigqyqlkmqissgt-kt<br>.........................<br>.........................<br>.........................<br>.........................	Rn_ENSRNOP00000015778<br>Mm_ENSMUSP00000099547<br>Gg<br>Xt_ENSXETP00000016007<br>Dr<br>Ce<br>Dm<br>Sc	2.3.2.13)(Transglutaminase-5)(TGase 5)(Transglutaminase X)(TGase X)(TGX)(TG(X))
290	1	C	ENSP00000338711<br>ENSG00000121236	729<br>373<br>379<br>372<br>-<br>-<br>-<br>-<br>-<br>-	vycrtysrhmKYVVRrcanrqnlyt<br>vycrirscnikfavqqstsyenays<br>vcstpv--epmfsfsqysskqgays<br>vytrkr--tlrfdvrqrkgqpngyh<br>.........................<br>.........................<br>.........................<br>.........................<br>.........................<br>.........................	Hs_ENSP00000338711<br>Bt_ENSBTAP00000007366<br>Rn_ENSRNOP00000023043<br>Mm_ENSMUSP00000055058<br>Gg<br>Xt<br>Dr<br>Ce<br>Dm<br>Sc	NP_001003827 Tripartite motif-containing protein 34 (Interferon-responsive finger protein 1)(RING finger protein 21)
291	1	CI	ENSP00000251127<br>ENSG00000102452	1491<br>-<br>1220<br>1491<br>1491<br>-<br>1489<br>1551<br>1826<br>-	regviptfrvKFLLRllrgrlevdl<br>.........................<br>regviptfrvKFLLRllrgrlevdl<br>regviptfrvKFLLRllrgrlevdl<br>regviptfrvKFLLRllrgrlevdl<br>.........................<br>regviptsrvKFLLRllrgrlevdl<br>qkrsipvrrvKFLLRllkgrlevnd<br>qrgvipvrrvKFILRllkgrlecdp<br>.........................	Hs_ENSP00000251127<br>Bt<br>Rn_ENSRNOP00000006624<br>Mm_ENSMUSP00000000201<br>Gg_ENSGALP00000027197<br>Xt<br>Dr_ENSDARP00000069861<br>Ce_CE38941<br>Dm_FBpp0111727<br>Sc	NP_443099 Sodium leak channel non-selective protein (Voltage gated channel-like protein 1)(CanIon)
292	1	CI	ENSP00000355736<br>ENSG00000184786	146<br>106<br>141<br>139<br>93<br>-<br>-<br>-<br>-<br>-	avkefgyhryKFIIKvlfiqktgqa<br>svkefgyhryKFIIKvlfiqktgqa<br>avkefafhryKFIIKvlfiqktgqa<br>avkefayhrykfiiqvlfiqktgqa<br>atkemgfdrykyvvtvlivekagqa<br>.........................<br>.........................<br>.........................<br>.........................<br>.........................	Hs_ENSP00000355736<br>Bt_ENSBTAP00000001210<br>Rn_ENSRNOP00000020552<br>Mm_ENSMUSP00000095011<br>Gg_ENSGALP00000031203<br>Xt<br>Dr<br>Ce<br>Dm<br>Sc	NP_777570 Tctex1 domain-containing protein 3 (T-complex testis-specific protein 3)(T-complex-associated testis-expressed protein 3)(Tcte-3)
293	1	CI	ENSP00000376855<br>ENSG00000175497	636<br>486<br>633<br>636<br>611<br>395<br>-<br>648<br>763<br>-	vevkdqitavKFLLK--lpyidskr<br>vevkdqiaavKFLLK--qpyidskr<br>veakdqiaaiKYLLK--qpyidskr<br>veakdqvaavKYLLK--qpyidskr<br>vevkdqiaavesllk--qpfidpkr<br>vevkdqiaavewllk--epfidpnr<br>.........................<br>vevedqikaikvvlrlyrhlldarr<br>vevsdqlevseylrdn-lhfidsrr<br>.........................	Hs_ENSP00000376855<br>Bt_ENSBTAP00000006894<br>Rn_ENSRNOP00000003527<br>Mm_ENSMUSP00000108225<br>Gg_ENSGALP00000019825<br>Xt_ENSXETP00000001378<br>Dr<br>Ce_CE21196<br>Dm_FBpp0088584<br>Sc	Inactive dipeptidyl peptidase 10 (Dipeptidyl peptidase X)(Dipeptidyl peptidase-like protein 2)(DPL2)(Dipeptidyl peptidase IV-related protein 3)(DPRP-3)
294	1	CI	ENSP00000349883<br>ENSG00000088387	1351<br>1309<br>1311<br>1360<br>1363<br>-<br>1325<br>-<br>1537<br>-	clhqfqymgkRYIAR----------<br>clhqfqymgkRYIARnq--------<br>clhqfqymgkRYIARsq--------<br>clhqfqymgkRYIARnq--------<br>clhqfqymgkRYIARnq--------<br>.........................<br>clhqfrymgkRFIARkinvslltay<br>.........................<br>cllqfryvgkksvvitt--------<br>.........................	Hs_ENSP00000349883<br>Bt_ENSBTAP00000013726<br>Rn_ENSRNOP00000040598<br>Mm_ENSMUSP00000097871<br>Gg_ENSGALP00000027222<br>Xt<br>Dr_ENSDARP00000084607<br>Ce<br>Dm_FBpp0079084<br>Sc	NP_056111 Dedicator of cytokinesis protein 9 (Cdc42 guanine nucleotide exchange factor zizimin-1)
295	1	CI	ENSP00000263726<br>ENSG00000121454	40<br>34<br>40<br>40<br>-<br>-<br>17<br>-<br>-<br>-	cagcnqhildKFILKvldrhwhssc<br>cagcnqhildKFILKvldrhwhssc<br>cagcnqhildKFILKvldrhwhssc<br>cagcnqhildKFILKvldrhwhssc<br>.........................<br>.........................<br>cagcsqhildKFILKvldrhwhskc<br>.........................<br>.........................<br>.........................	Hs_ENSP00000263726<br>Bt_ENSBTAP00000005216<br>Rn_ENSRNOP00000041633<br>Mm_ENSMUSP00000027740<br>Gg<br>Xt<br>Dr_ENSDARP00000057643<br>Ce<br>Dm<br>Sc	NP_203129 LIM/homeobox protein Lhx4 (LIM homeobox protein 4)
296	1	CI	ENSP00000329419<br>ENSG00000184432	64<br>64	evcdlpvraaKFVARknwvvtgadd<br>evcdlpvraaKFVARknwvvtgadd	Hs_ENSP00000329419<br>Bt_ENSBTAP00000019767	NP_004757 Coatomer subunit beta' (Beta'-coat protein)(Beta'-

				-<br>64<br>64<br>63<br>64<br>64<br>64<br>62	.........................<br>evcdlpvraaKFVARknwvvtgadd<br>evcdlpvraaKFVARknwvvtgadd<br>evcdlpvraaKFVARknwvvtgadd<br>evcdlpvrasKFVARknwvitgadd<br>evcdvpvraakfvprkswvvtgsdd<br>evcdvpvrsaRFVARknwiltgsdd<br>qvtetpvragKFIARknwiivgsdd	Rn<br>Mm_ENSMUSP00000035033<br>Gg_ENSGALP00000008578<br>Xt_ENSXETP00000018095<br>Dr_ENSDARP00000044330<br>Ce_CE18673<br>Dm_FBpp0080048<br>Sc_YGL137W	COP)(p102)
297	1	CI	ENSP00000363495<br>ENSG00000204183	69<br>-<br>-<br>99<br>-<br>-<br>-<br>-<br>-<br>-	ralislknrsRFLVRpntrnrafsw<br>.........................<br>.........................<br>ralislknrsRFLVRpntrnkafsw<br>.........................<br>.........................<br>.........................<br>.........................<br>.........................<br>.........................	Hs_ENSP00000363495<br>Bt<br>Rn<br>Mm_ENSMUSP00000105257<br>Gg<br>Xt<br>Dr<br>Ce<br>Dm<br>Sc	Protein GDF5OS, mitochondrial Precursor (Growth/differentiation factor 5 opposite strand transcript protein)
298	1	CI	ENSP00000278911<br>ENSG00000154134	3<br>3<br>3<br>3<br>-<br>-<br>3<br>-<br>-<br>-	--------mlRYLLKtllqmnlfad<br>--------mlRYLLKtllqmnlfad<br>--------mlRYLLKtllqmnlfad<br>--------mlRYLLKtllqmnlfad<br>.........................<br>.........................<br>--------mlRYLIKtllqmnlftd<br>.........................<br>.........................<br>.........................	Hs_ENSP00000278911<br>Bt_ENSBTAP00000005697<br>Rn_ENSRNOP00000051539<br>Mm_ENSMUSP00000110690<br>Gg<br>Xt<br>Dr_ENSDARP00000027377<br>Ce<br>Dm<br>Sc	NP_071765 Roundabout homolog 3 Precursor (Roundabout-like protein 3)
299	1	CI	ENSP00000353010<br>ENSG00000066739	1547<br>1552<br>-<br>1546<br>1547<br>1529<br>-<br>1474<br>-<br>-	hfp---ipviRYVVKevslvwhlyg<br>hfp---ipvvRYLVKevslvwhlyg<br>.........................<br>hfp---ipavRYVVKevslvwhlyg<br>hfp---iplvRYVVKeisliwhlyg<br>hfp---vpvvRYVVKevslvwhlyg<br>.........................<br>tmsgqinpvlryflkdvtvrlslya<br>.........................<br>.........................	Hs_ENSP00000353010<br>Bt_ENSBTAP00000030494<br>Rn<br>Mm_ENSMUSP00000037441<br>Gg_ENSGALP00000018041<br>Xt_ENSXETP00000030481<br>Dr<br>Ce_CE30679<br>Dm<br>Sc	NP_060506 Autophagy-related protein 2 homolog B
300	1	CI	ENSP00000301233<br>ENSG00000167635	169<br>169<br>169<br>169<br>-<br>-<br>-<br>-<br>-<br>-	secgtafgqkKYLIKhqnihtgekp<br>necgtafgqkKYLIKhqnihtgekp<br>necgtafgqkKYLIKhqnihtgekp<br>necgtafgqkKYLIKhqnihtgekp<br>.........................<br>.........................<br>.........................<br>.........................<br>.........................<br>.........................	Hs_ENSP00000301233<br>Bt_ENSBTAP00000047326<br>Rn_ENSRNOP00000008889<br>Mm_ENSMUSP00000058588<br>Gg<br>Xt<br>Dr<br>Ce<br>Dm<br>Sc	NP_001093108 Zinc finger protein OZF (Only zinc finger protein) (Zinc finger protein 146)
301	1	C	ENSP00000353874<br>ENSG00000173366	901<br>897<br>-<br>901<br>-<br>-<br>928<br>-<br>-<br>-	lrgqleecrgRWALRlcleerdwlp<br>lrvqleerrgrralrlcleerdwlp<br>.........................<br>lrvrleerrgrralrlcledrdwlp<br>.........................<br>.........................<br>mlvrlen-rgrwrfqlcleerdwip<br>.........................<br>.........................<br>.........................	Hs_ENSP00000353874<br>Bt_ENSBTAP00000024223<br>Rn<br>Mm_ENSMUSP00000082207<br>Gg<br>Xt<br>Dr_ENSDARP00000064731<br>Ce<br>Dm<br>Sc	NP_059138 Toll-like receptor 9 Precursor (CD289 antigen)
302	1	CI	ENSP00000268182<br>ENSG00000140575	1161<br>1112<br>1163<br>1161<br>1167<br>1126<br>1170<br>944<br>-<br>-	ssvdkipygmRFIAKvlkdslhekf<br>ssvdkipygmRFIAKvlkdslhekf<br>ssvdkipygmRFIAKvlkdslhekf<br>ssvdkipygmRFIAKvlkdslhekf<br>ssvdkipygmRFIAKvlkdslhdkf<br>ssvdkipygm-ltagavesnnvefl<br>ssldnipygmRYIAKvlkdalhekf<br>ksfk-lpkscvymtsyletalrhqf<br>.........................<br>.........................	Hs_ENSP00000268182<br>Bt_ENSBTAP00000017366<br>Rn_ENSRNOP00000018021<br>Mm_ENSMUSP00000032751<br>Gg_ENSGALP00000013987<br>Xt_ENSXETP00000002788<br>Dr_ENSDARP00000078504<br>Ce_CE23636<br>Dm<br>Sc	NP_003861 Ras GTPase-activating-like protein IQGAP1 (p195)
303	1	CI	ENSP00000264042<br>ENSG00000006607	279<br>-	kvrklsfkrkRFLIKlhpevhgpyq<br>.........................	Hs_ENSP00000264042<br>Bt	NP_055623 FERM, RhoGEF and pleckstrin domain-containing

				279<br>279<br>288<br>-<br>-<br>-<br>309<br>-	kvrklsfkrkRFLIKlhpevhgpyq<br>kvrklsfkrkRFLIKlhpevhgpyq<br>kvrklsfkrkRFLIKlhpevcgpyq<br>.........................<br>.........................<br>.........................<br>kirkisfkrkRFLVKlhpegygyyk<br>.........................	Rn_ENSRNOP00000024440<br>Mm_ENSMUSP00000108530<br>Gg_ENSGALP00000009285<br>Xt<br>Dr<br>Ce<br>Dm_FBpp0110258<br>Sc	protein 2 (FERM domain including RhoGEF)(FIR)
304	1	CI	ENSP00000326417<br>ENSG00000136826	219<br>228<br>215<br>225<br>-<br>156<br>162<br>-<br>258<br>-	pqppggglmgKFVLKaslsapgsey<br>pqppggglmgKFVLKaslsapgsey<br>pqppggglmgKFVLKaslstpgsey<br>pqppggglmgKFVLKaslttpgsey<br>.........................<br>pq---aslqgKFVLKttvsmg--dy<br>ghldrleyt-elralntrnqqhltn<br>.........................<br>qlqlelphpnpklspvlphpqlqdy<br>.........................	Hs_ENSP00000326417<br>Bt_ENSBTAP00000027125<br>Rn_ENSRNOP00000022255<br>Mm_ENSMUSP00000103245<br>Gg<br>Xt_ENSXETP00000012646<br>Dr_ENSDARP00000056654<br>Ce<br>Dm_FBpp0080668<br>Sc	Krueppel-like factor 4 (Epithelial zinc finger protein EZF)(Gut-enriched krueppel-like factor)
305	1	CI	ENSP00000322727<br>ENSG00000147044	711<br>693<br>711<br>711<br>705<br>564<br>684<br>746<br>679<br>-	fgkkkkqykdKYLAKhnavfdqldl<br>fgkkkkqykdKYLAKhnavfdqldl<br>fgkkkkqykdKYLAKhnavfdqldl<br>fgkkkkqykdKYLAKhnavfdqldl<br>fgkkkkqykdKYLAKhnavfdqldl<br>fgkkkkqykdKYLAKhnavfdqldl<br>fgkkkkqykdKYLAKhnavfdqldl<br>fnkkkkyyttkylqkhsalfdqldl<br>fgrkkkqcrdKYLAKhnaifdtldv<br>.........................	Hs_ENSP00000322727<br>Bt_ENSBTAP00000026825<br>Rn_ENSRNOP00000004187<br>Mm_ENSMUSP00000112322<br>Gg_ENSGALP00000026115<br>Xt_ENSXETP00000010527<br>Dr_ENSDARP00000040842<br>Ce_CE27131<br>Dm_FBpp0083559<br>Sc	Peripheral plasma membrane protein CASK (hCASK)(EC 2.7.11.1)(Calcium/calmodulin-dependent serine protein kinase)(Lin-2 homolog)
306	1	CI	ENSP00000216327<br>ENSG00000100439	42<br>55<br>55<br>55<br>-<br>41<br>77<br>128<br>83<br>82	earilqclqnKFLARyvslp----n<br>earilqclqnKFLARyvslp----n<br>earilqclqnKFLARyvslp----n<br>earilqclqnKFLARyvslp----n<br>.........................<br>esrilqciknkfsaqyvslp----d<br>eakilacirnevwsrfvtlp----n<br>egrilsalgiKYLARliqip---fk<br>ekkilsyvklpyrgffvdigpavge<br>ptvlihgyaassmafyrtfenlsdn	Hs_ENSP00000216327<br>Bt_ENSBTAP00000022154<br>Rn_ENSRNOP00000012837<br>Mm_ENSMUSP00000044134<br>Gg<br>Xt_ENSXETP00000017566<br>Dr_ENSDARP00000028738<br>Ce_CE08627<br>Dm_FBpp0087910<br>Sc_YLR099C	NP_071343 Abhydrolase domain-containing protein 4 (EC 3.1.1.-)(Lyso-N-acylphosphatidylethanolamine lipase)(Alpha/beta-hydrolase 4)
307	1	CI	ENSP00000364144<br>ENSG00000154144	74<br>74<br>72<br>73<br>-<br>74<br>-<br>-<br>45<br>-	ekflkakeerRYLLKkllqlqalte<br>ekflkakeerRYLLKkllqlqalte<br>ekflkakeerRYLLKkllqihalte<br>ekflkakeerRYLLKkllqihalte<br>.........................<br>dkfirakeerRFLLKrllqlqalse<br>.........................<br>.........................<br>aelsvtrsqrlylierlmfhegle-<br>.........................	Hs_ENSP00000364144<br>Bt_ENSBTAP00000030273<br>Rn_ENSRNOP00000042798<br>Mm_ENSMUSP00000112600<br>Gg<br>Xt_ENSXETP00000020169<br>Dr<br>Ce<br>Dm_FBpp0084165<br>Sc	NP_116200 Transforming growth factor beta regulator 1 (Nuclear interactor of ARF and Mdm2)
308	1	CI	ENSP00000381571<br>ENSG00000104728	639<br>464<br>65<br>615<br>625<br>598<br>-<br>-<br>666<br>-	---------sRYLIRsddmietvyn<br>---------sRYLIRsddvietvyn<br>---------tqplgcss-veelllm<br>---------nRYLVRsddvietvyn<br>---------nRYLIRtddmvetvyn<br>---------sRYLVRsddmvetvfn<br>.........................<br>.........................<br>vsdsagghrpRYLLRednvthmefn<br>.........................	Hs_ENSP00000381571<br>Bt_ENSBTAP00000011327<br>Rn_ENSRNOP00000016745<br>Mm_ENSMUSP00000081225<br>Gg_ENSGALP00000026323<br>Xt_ENSXETP00000025378<br>Dr<br>Ce<br>Dm_FBpp0289665<br>Sc	Rho guanine nucleotide exchange factor 10
308	2	CI	ENSP00000381571<br>ENSG00000104728	693<br>525<br>124<br>669<br>681<br>654<br>-<br>-<br>718<br>-	hdsrvmss-qRYLLKwsvplghvda<br>hdggavtsgqRYLLKwsvplgqaev<br>heshammn-qRYLLKwsvplgqvdv<br>heshavms-qRYLLKwsvplghvdv<br>essgtmttgqRYLLKwsvplgqvev<br>egssavttnqKYLLKwsvplshaev<br>.........................<br>.........................<br>kqshdfgatekltfkwmfpvndvei<br>.........................	Hs_ENSP00000381571<br>Bt_ENSBTAP00000011327<br>Rn_ENSRNOP00000016745<br>Mm_ENSMUSP00000081225<br>Gg_ENSGALP00000026323<br>Xt_ENSXETP00000025378<br>Dr<br>Ce<br>Dm_FBpp0289665<br>Sc	Rho guanine nucleotide exchange factor 10
309	1	C	ENSP00000381162<br>ENSG00000003989	415<br>375	famardgllfRFLARvs-krqspva<br>yamaedgllfkclaqinsktktpii	Hs_ENSP00000381162<br>Bt_ENSBTAP00000013304	NP_003037 Low affinity cationic amino acid transporter 2 (CAT-2)

				422	famardgllfRFLARvs-krqspva	Rn_ENSRNOP00000055811	(CAT2)(Solute carrier family 7 member 2)
				392	famardgllfRFLARvs-krqspva	Mm_ENSMUSP00000112848	
				375	famardgllfsflakls-krqapll	Gg_ENSGALP00000022132	
				376	famardgllfrflsnvs-arqspva	Xt_ENSXETP00000001508	
				374	famardgvlfrflskls-krqspva	Dr_ENSDARP00000053920	
				-	....	Ce	
				366	famsndgllfkflgdisekyktpfk	Dm_FBpp0271785	
				-	....	Sc	
310	1	CI	ENSP00000385673 ENSG00000088538	251	dmregkqiseRFLVRlnknggprnp	Hs_ENSP00000385673	NP_004938 Dedicator of cytokinesis protein 3 (Modifier of cell adhesion)(Presenilin-binding protein)(PBP)
				0	-------------------------	Bt_ENSBTAP00000002440	
				0	-------------------------	Rn_ENSRNOP00000019867	
				251	dmregkqiseRFLVRlnknggprnp	Mm_ENSMUSP00000047652	
				146	dvregkqiserfmvrlnknggprnp	Gg_ENSGALP00000003671	
				-	....	Xt	
				157	dlregkqisekfmvklnknggpknp	Dr_ENSDARP00000086523	
				-	....	Ce	
				255	dgshmrplseRFLVKiskdgfsnyi	Dm_FBpp0288435	
				-	....	Sc	
311	1	C	ENSP00000237449 ENSG00000115423	376	eakyvvrracRFALRaagfvpddca	Hs_ENSP00000237449	NP_001361 Dynein heavy chain 6, axonemal (Axonemal beta dynein heavy chain 6)(Ciliary dynein heavy chain 6)
				205	eakdvvrkacrvalraagfipddca	Bt_ENSBTAP00000033955	
				-	....	Rn	
				373	aakdvvrkscRFALRaagfipddce	Mm_ENSMUSP00000109674	
				-	....	Gg	
				-	....	Xt	
				369	lvmevarnacrnalkefghtpdyge	Dr_ENSDARP00000054387	
				-	....	Ce	
				349	fiaktifractdaiqargfypdd-e	Dm_FBpp0074283	
				-	....	Sc	
312	1	CI	ENSP00000358210 ENSG00000112246	296	vkgqvttkyyRFLAKhggwvwvqsy	Hs_ENSP00000358210	NP_005059 Single-minded homolog 1
				-	....	Bt	
				296	vkgqvttkyyRFLAKhggwvwvqsy	Rn_ENSRNOP00000053843	
				296	vkgqvttkyyRFLAKqggwvwvqsy	Mm_ENSMUSP00000020071	
				296	vkgqvttkyyRFLAKhggwvwvqsy	Gg_ENSGALP00000024893	
				296	vkgqvttkyyRFLAKhggwvwvqsy	Xt_ENSXETP00000005117	
				245	vkgqvttkyyRFLAKqggwvwvqsy	Dr_ENSDARP00000092087	
				-	....	Ce	
				-	....	Dm	
				-	....	Sc	
313	1	CI	ENSP00000258526 ENSG00000136040	1526	neevalteiyKYIVKyfdeiln--k	Hs_ENSP00000258526	NP_005752 Plexin-C1 Precursor (Virus-encoded semaphorin protein receptor)(CD232 antigen)
				1537	neevalteiyKYIIKyfdeiln--k	Bt_ENSBTAP00000014932	
				1532	neevalteiyKYIVKyfdeiln--k	Rn_ENSRNOP00000011178	
				1532	neevalteiyKYIVKyfdeiln--k	Mm_ENSMUSP00000096939	
				1450	neevalmeiyRYIVKyydeivs--k	Gg_ENSGALP00000018429	
				1311	kedvallelykyikryhqvvld--m	Xt_ENSXETP00000051355	
				1014	nesaalrelykymqryftevfifqk	Dr_ENSDARP00000091877	
				-	....	Ce	
				-	....	Dm	
				-	....	Sc	
314	1	CI	ENSP00000350422 ENSG00000123444	136	qltslfeecsRFLARtvqvgnclqv	Hs_ENSP00000350422	NP_057590 Kelch repeat and BTB domain-containing protein 4 (BTB and kelch domain-containing protein 4)
				153	qltslfeecsRFLARtvqvgnclqv	Bt_ENSBTAP00000024595	
				136	qltslfeecsRFLARtvqvgnclqv	Rn_ENSRNOP00000012876	
				152	qltslfeecsRFLARtvqvgnclqv	Mm_ENSMUSP00000107089	
				137	qltalfeecsRFLARtvqvrnclqv	Gg_ENSGALP00000013113	
				139	qltalfeecsRFLVRtvqvknclqi	Xt_ENSXETP00000057289	
				140	qltalfeecsrflsrtvdvrnclqv	Dr_ENSDARP00000091441	
				-	....	Ce	
				-	....	Dm	
				-	....	Sc	
315	1	CI	ENSP00000340883 ENSG00000099956	301	dmsekenspeKFALKlcselglgge	Hs_ENSP00000340883	SWI/SNF-related matrix-associated actin-dependent regulator of chromatin subfamily B member 1 (Integrase interactor 1 protein)(hSNF5)(BAF47)
				280	dmsekenspeKFALKlcselglgge	Bt_ENSBTAP00000048071	
				292	dmsekenspeKFALKlcselglgge	Rn_ENSRNOP00000034916	
				292	dmsekenspeKFALKlcselglgge	Mm_ENSMUSP00000000925	
				293	dmsekenspeKFALKlcselglgge	Gg_ENSGALP00000009608	
				285	dmsekenspeKFALKlcselglgge	Xt_ENSXETP00000048417	
				297	dmsekenspesfalklcselglgge	Dr_ENSDARP00000062189	
				288	dmsdpnnspeefsrnickelglgge	Ce_CE01045	
				277	dmseknnnpeefaiklcaelglgge	Dm_FBpp0078331	
				574	eisnsdncpeefaesmcqelelpge	Sc_YBR289W	
316	1	CI	ENSP00000296257 ENSG00000163904	3	--------myRWLVRilgtifrfcd	Hs_ENSP00000296257	NP_067640 Sentrin-specific protease 2 (EC 3.4.22.-)
				3	--------myKWLIRilgtifrfcd	Bt_ENSBTAP00000002296	

				3<br>3<br>0<br>-<br>-<br>-<br>-<br>-	--------myrwltkvlgtilrlce<br>--------myRWLAKvlgtilrlce<br>-------------------------<br>.........................<br>.........................<br>.........................<br>.........................<br>.........................	Rn_ENSRNOP00000002425<br>Mm_ENSMUSP00000023561<br>Gg_ENSGALP00000040363<br>Xt<br>Dr<br>Ce<br>Dm<br>Sc	(Sentrin/SUMO-specific protease SENP2)(SMT3-specific isopeptidase 2)(Smt3ip2)(Axam2)
317	1	CI	ENSP00000340842<br>ENSG00000188032	10<br>10<br>10<br>10<br>-<br>-<br>80<br>-<br>-<br>-	-lfspitqqlRYLLKkaddfqsyll<br>-mfspiteqlcyllkkaddfqsyll<br>-mfspitdqlRYLLRkaddfqsyll<br>-mfspitdqlRYLLRkaddfqsyll<br>.........................<br>.........................<br>dkisriekqlqyllskadefqtqlv<br>.........................<br>.........................<br>.........................	Hs_ENSP00000340842<br>Bt_ENSBTAP00000022527<br>Rn_ENSRNOP00000007407<br>Mm_ENSMUSP00000062468<br>Gg<br>Xt<br>Dr_ENSDARP00000092184<br>Ce<br>Dm<br>Sc	UPF0575 protein C19orf67
318	1	CI	ENSP00000156471<br>ENSG00000021776	58<br>58<br>58<br>58<br>66<br>14<br>49<br>57<br>73<br>-	iyekeivks-RFAIRkimllefsqy<br>iyekeivks-RFAIRkimllefsqy<br>iyekeivks-RFAIRkimllefsqy<br>iyekeivks-RFAIRkimllefsqy<br>vyakeivks-KFAIRkimllefsqy<br>iyvkeiaas-rfsikkimllefsqy<br>vtnlsfnsh-lfsirkimllefsqy<br>iydnemlkt-sfnsrkimmlefsqy<br>iyneeigdggghsarrinmlefsqy<br>.........................	Hs_ENSP00000156471<br>Bt_ENSBTAP00000016895<br>Rn_ENSRNOP00000011950<br>Mm_ENSMUSP00000047157<br>Gg_ENSGALP00000015978<br>Xt_ENSXETP00000028235<br>Dr_ENSDARP00000094409<br>Ce_CE40670<br>Dm_FBpp0081950<br>Sc	NP_055506 Intron-binding protein aquarius (Intron-binding protein of 160 kDa)(IBP160)
319	1	C	ENSP00000350018<br>ENSG00000100092	404<br>370<br>324<br>404<br>384<br>-<br>-<br>-<br>-<br>-	enlsnlrylmKFLARlaeeqevnkm<br>enlsnlrylmkflaqlaeeqevnkm<br>enfnnlrylmkflallaeeqdvnkm<br>enfnnlrylmkflallaeeqdvnkm<br>dsynnlryliKFLAKlaehqnvnkm<br>.........................<br>.........................<br>.........................<br>.........................<br>.........................	Hs_ENSP00000350018<br>Bt_ENSBTAP00000029247<br>Rn_ENSRNOP00000012480<br>Mm_ENSMUSP00000001226<br>Gg_ENSGALP00000020278<br>Xt<br>Dr<br>Ce<br>Dm<br>Sc	NP_061830 SH3 domain-binding protein 1 (3BP-1)
320	1	CI	ENSP00000064780<br>ENSG00000054967	418<br>425<br>423<br>424<br>-<br>313<br>399<br>-<br>-<br>-	ppaenkaeenRYVVRlsesnlvi--<br>ppaenkaeenRYVVRlsesnlvi--<br>ppaenkaeenRYVVRlsesnlvi--<br>ppaenkaeenRYVVRlsesnlvi--<br>.........................<br>-------------------------<br>nsasakarqeinv------------<br>.........................<br>.........................<br>.........................	Hs_ENSP00000064780<br>Bt_ENSBTAP00000021927<br>Rn_ENSRNOP00000036110<br>Mm_ENSMUSP00000008462<br>Gg<br>Xt_ENSXETP00000007756<br>Dr_ENSDARP00000092468<br>Ce<br>Dm<br>Sc	NP_116260 Tumor necrosis factor receptor superfamily member 19L Precursor (Receptor expressed in lymphoid tissues)
321	1	CI	ENSP00000317156<br>ENSG00000101639	1354<br>1147<br>-<br>659<br>1001<br>-<br>-<br>-<br>-<br>-	dpkvlrifpdKFVLKertqenvtli<br>dpkvlrifpdKFVLKertqedvtii<br>.........................<br>dpkvlrifpdKFVLKertqehitlv<br>dpqvmtvspeKFILRegahemitvt<br>.........................<br>.........................<br>.........................<br>.........................<br>.........................	Hs_ENSP00000317156<br>Bt_ENSBTAP00000017803<br>Rn<br>Mm_ENSMUSP00000089474<br>Gg_ENSGALP00000022468<br>Xt<br>Dr<br>Ce<br>Dm<br>Sc	NP_115518 Centrosomal protein of 192 kDa (Cep192)
322	1	CI	ENSP00000351520<br>ENSG00000197217	69<br>-<br>68<br>69<br>69<br>69<br>69<br>24<br>-<br>-	grltrdkkfqRYLARvtdieatdtn<br>.........................<br>gwlskdkkfqRYLARvtdveatdtn<br>gwlskdkkfqRYLARvtdveatdtn<br>grasrdkrfhRYLARvtdteatdtn<br>ggsyldikyqRYLARvtdmdatdts<br>swlredrhihtrlgrvpdmeatdtn<br>-----------yvveahtspkviad<br>.........................<br>.........................	Hs_ENSP00000351520<br>Bt<br>Rn_ENSRNOP00000022440<br>Mm_ENSMUSP00000065046<br>Gg_ENSGALP00000000530<br>Xt_ENSXETP00000016966<br>Dr_ENSDARP00000056073<br>Ce_CE28748<br>Dm<br>Sc	NP_001122402 Ectonucleoside triphosphate diphosphohydrolase 4 (NTPDase 4)(EC 3.6.1.6)(Uridine-diphosphatase)(UDPase) (Lysosomal apyrase-like protein of 70 kDa)
323	1	CI	ENSP00000355210<br>ENSG00000198792	79<br>91	lrcyscpneqRYIVRilfivpiyaf<br>lrcyscpneqRYIVRilfivpiyaf	Hs_ENSP00000355210<br>Bt_ENSBTAP00000012742	NP_036396 Transmembrane protein 184B (Putative MAPK-

				79<br>79<br>82<br>74<br>91<br>50<br>99<br>-	lrcysrpneqrhivrilfivpiyaf<br>lrcysrpneqrhivrilfivpiyaf<br>lrcyscpneqRYIVRilfivpiyaf<br>lrsyscpneqrhivrilfivpiyaf<br>lryyssspkeqrhivrilfivpiyaf<br>lrfyscpaeqRWIVRilfivpiyaf<br>lrwytnpqeqRWIVRilfivpiyat<br>.........................	Rn_ENSRNOP00000034031<br>Mm_ENSMUSP00000074518<br>Gg_ENSGALP00000020022<br>Xt_ENSXETP00000013922<br>Dr_ENSDARP00000066388<br>Ce_CE31508<br>Dm_FBpp0112035<br>Sc	activating protein FM08)
324	1	CI	ENSP00000369716<br>ENSG00000170004	1850<br>1759<br>1843<br>1844<br>-<br>1812<br>-<br>1572<br>-<br>-	--gnflemknKFLARrfklleqalv<br>--gnflemknKFLARrfklleqalv<br>--gnflemknKFLARrfklleqalv<br>--gnflemknKFLARrfklleqalv<br>.........................<br>--gnflemknKFLARrfklleqalv<br>.........................<br>patnfadvknKFLARrfklleqslv<br>.........................<br>.........................	Hs_ENSP00000369716<br>Bt_ENSBTAP00000019120<br>Rn_ENSRNOP00000012983<br>Mm_ENSMUSP00000058386<br>Gg<br>Xt_ENSXETP00000028484<br>Dr<br>Ce_CE03657<br>Dm<br>Sc	NP_001005271 Chromodomain-helicase-DNA-binding protein 3 (CHD-3)(EC 3.6.1.-)(ATP-dependent helicase CHD3)(Mi-2 autoantigen 240 kDa protein)(Mi2-alpha)(Zinc finger helicase)(hZFH)
325	1	CI	ENSP00000231484<br>ENSG00000113555	984<br>983<br>984<br>984<br>921<br>-<br>-<br>-<br>-<br>-	fqpkpnhrgnKYLAKpg-gsrsaip<br>fqpkpnhrgnKYLAKpg-ssrstip<br>fqpkpnhrgnkysakpg-ssratip<br>fqpkpnhrgnKYLAKpggssrgtip<br>fqpktnhrgnkytakng--sraagl<br>.........................<br>.........................<br>.........................<br>.........................<br>.........................	Hs_ENSP00000231484<br>Bt_ENSBTAP00000011099<br>Rn_ENSRNOP00000026065<br>Mm_ENSMUSP00000025311<br>Gg_ENSGALP00000004006<br>Xt<br>Dr<br>Ce<br>Dm<br>Sc	NP_057664 Protocadherin-12 Precursor (Vascular cadherin-2)(Vascular endothelial cadherin-2)(VE-cadherin-2)(VE-cad-2)
326	1	CI	ENSP00000310094<br>ENSG00000149930	279<br>-<br>279<br>279<br>-<br>-<br>-<br>-<br>-<br>-	rptsevllkhRFVLRerpptvimdl<br>.........................<br>rptsevllkhRFVLRerpptvimdl<br>rptsevllkhRFVLRerpptvimdl<br>.........................<br>.........................<br>.........................<br>.........................<br>.........................<br>.........................	Hs_ENSP00000310094<br>Bt<br>Rn_ENSRNOP00000027080<br>Mm_ENSMUSP00000112963<br>Gg<br>Xt<br>Dr<br>Ce<br>Dm<br>Sc	NP_057235 Serine/threonine-protein kinase TAO2 (EC 2.7.11.1)(Thousand and one amino acid protein 2)(Prostate-derived STE20-like kinase 1)(PSK-1)(Kinase from chicken homolog C)(hKFC-C)
327	1	CI	ENSP00000262189<br>ENSG00000055609	4721<br>4631<br>4727<br>4714<br>4690<br>4642<br>-<br>-<br>2239<br>-	sepkmsahvkRFVLRphtlnststs<br>sepkmsahvkRFVLRphtlnststs<br>aepkmsahvkRFVLRphtlnststs<br>sepkmsahvkRFVLRphtlnststs<br>aepkmsthvkRFVLRphtlnststs<br>sepkmnshvkRFVLRphtlnststs<br>.........................<br>.........................<br>tepkqrqllvwrkphtqrtagscst<br>.........................	Hs_ENSP00000262189<br>Bt_ENSBTAP00000028347<br>Rn_ENSRNOP00000010685<br>Mm_ENSMUSP00000043874<br>Gg_ENSGALP00000010110<br>Xt_ENSXETP00000021447<br>Dr<br>Ce<br>Dm_FBpp0070347<br>Sc	NP_733751 Histone-lysine N-methyltransferase MLL3 (EC 2.1.1.43)(Myeloid/lymphoid or mixed-lineage leukemia protein 3)(Homologous to ALR protein)(Lysine N-methyltransferase 2C)
328	1	C	ENSP00000335272<br>ENSG00000187008	1074<br>857<br>1430<br>1461<br>-<br>-<br>-<br>-<br>-<br>-	vvpenhhhfccylhrvlqrlkshlg<br>vvpenhrhfcsyllrvlqrlqshlq<br>gvseshshfcRYLLRvlqslklrlg<br>avsenhyhfcryllqllrrlkahle<br>.........................<br>.........................<br>.........................<br>.........................<br>.........................<br>.........................	Hs_ENSP00000335272<br>Bt_ENSBTAP00000026493<br>Rn_ENSRNOP00000038974<br>Mm_ENSMUSP00000104865<br>Gg<br>Xt<br>Dr<br>Ce<br>Dm<br>Sc	Polycystic kidney disease protein 1-like 3 Precursor (Polycystin-1L3)(PC1-like 3 protein)
329	1	CI	ENSP00000387010<br>ENSG00000048991	253<br>253<br>253<br>253<br>253<br>-<br>88<br>-<br>526<br>-	kddkgedfqkRYILKrdnssfdkd-<br>kddkgedfqkRYILKrdnssfdkd-<br>kddrgedfqkRYILKrdnssfdkd-<br>kddrgedfqkRYILKrdnssfdkd-<br>kdeksddfqkRYILKrdnssldkd-<br>.........................<br>kddktddfqkRYILKrdnssldqd-<br>.........................<br>rdd-----arksilkrdthsfdevr<br>.........................	Hs_ENSP00000387010<br>Bt_ENSBTAP00000017695<br>Rn_ENSRNOP00000005372<br>Mm_ENSMUSP00000043103<br>Gg_ENSGALP00000019969<br>Xt<br>Dr_ENSDARP00000086415<br>Ce<br>Dm_FBpp0111758<br>Sc	R3H domain-containing protein 1
330	1	CI	ENSP00000352254<br>ENSG00000064419	701<br>667	riverccrclRFAVRcvgkgsaall<br>riverccrclRFAVRcvgkgsaall	Hs_ENSP00000352254<br>Bt_ENSBTAP00000006573	Transportin-3 (Transportin-SR)(TRN-SR)(Importin-12)(Imp12)

				667<br>667<br>631<br>661<br>667<br>698<br>676<br>688	riverccrclRFAVRcvgkgsaall<br>riverccrclRFAVRcvgkgsaall<br>riverccrclRFAVRcvgkgsaall<br>riverccrclRFAVRcvgkgsaall<br>riverccrclRFAVRcvgkgsasll<br>rliehsirscrlifralgaqsmpfv<br>rimertcrlirygirmvrkqammlv<br>iivervakllrriferfhvfcepil	Rn_ENSRNOP00000040222<br>Mm_ENSMUSP00000110906<br>Gg_ENSGALP00000022392<br>Xt_ENSXETP00000032823<br>Dr_ENSDARP00000067160<br>Ce_CE37021<br>Dm_FBpp0077399<br>Sc_YOR160W	
331	1	CI	ENSP00000377021<br>ENSG00000115641	128<br>21<br>18<br>18<br>22<br>19<br>32<br>-<br>-<br>-	hhcneslfgkKYILReespycvvcf<br>hhcedslfgrKYVLReeqpycvacf<br>hhcneslygkKYILKeenphcvacf<br>hhcneslygkKYILKeenphcvacf<br>hyckeslfgkKYILRedspycvkcy<br>hyckeslfgkKYLLReenpycvkcy<br>hyckeslfgkKYVLRednpycvkcy<br>.........................<br>.........................<br>.........................	Hs_ENSP00000377021<br>Bt_ENSBTAP00000001440<br>Rn_ENSRNOP00000023014<br>Mm_ENSMUSP00000008280<br>Gg_ENSGALP00000027068<br>Xt_ENSXETP00000005493<br>Dr_ENSDARP00000061576<br>Ce<br>Dm<br>Sc	NP_963849 Four and a half LIM domains protein 2 (FHL-2) (Skeletal muscle LIM-protein 3) (SLIM 3)(LIM domain protein DRAL)
332	1	CI	ENSP00000325612<br>ENSG00000175221	284<br>-<br>308<br>308<br>314<br>273<br>274<br>-<br>307<br>-	kdkfpaithlKFLARdmseqvllca<br>.........................<br>kdrfpaithlKFLARdmseqvllca<br>kdrfpaithlKFLARdmseqvllca<br>kdkypaithlKFLARdmseqvllca<br>kdkfpavthlKFLARdmseqvllca<br>rdkypavthlkfltrenseqvllca<br>.........................<br>dgrrishlkwtrianedvifiayac<br>.........................	Hs_ENSP00000325612<br>Bt<br>Rn_ENSRNOP00000015257<br>Mm_ENSMUSP00000101017<br>Gg_ENSGALP00000003077<br>Xt_ENSXETP00000046771<br>Dr_ENSDARP00000059751<br>Ce<br>Dm_FBpp0071684<br>Sc	NP_005472 Mediator of RNA polymerase II transcription subunit 16 (Mediator complex subunit 16) (Thyroid hormone receptor-associated protein complex 95 kDa component)(Trap95)(Thyroid hormone receptor-associated protein 5)(Vitamin D3 receptor-interacting protein complex component DRIP92)
333	1	CI	ENSP00000298049<br>ENSG00000101210	423<br>424<br>423<br>423<br>426<br>423<br>423<br>423<br>-<br>-	esfsqypplgRFAVRdmrqtvavgv<br>esfsqypplgRFAVRdmrqtvavgv<br>esfsqypplgRFAVRdmrqtvavgv<br>esfsqypplgRFAVRdmrqtvavgv<br>esfsqypplgRFAVRdmrqtvavgv<br>esfsqypplgRFAVRdmrqtvavgv<br>esfsqypplgRFAVRdmrqtvavgv<br>esftdyaplgRFAVRdmrqtvavgv<br>.........................<br>.........................	Hs_ENSP00000298049<br>Bt_ENSBTAP00000028899<br>Rn_ENSRNOP00000016947<br>Mm_ENSMUSP00000054556<br>Gg_ENSGALP00000009371<br>Xt_ENSXETP00000042318<br>Dr_ENSDARP00000010921<br>Ce_CE01270<br>Dm<br>Sc	NP_001949 Elongation factor 1-alpha 2 (EF-1-alpha-2)(Elongation factor 1 A-2)(eEF1A-2)(Statin S1)
334	1	CI	ENSP00000357040<br>ENSG00000162738	454<br>508<br>454<br>494<br>392<br>454<br>-<br>-<br>-<br>-	agptiqyhkeRWLAKqwtlvseepv<br>agptiqyhkeRWLAKqwtlvseepv<br>agptiqyhkeRWLAKqwtlvseepv<br>agptiqyhkeRWLAKqwtlvseepv<br>agptiqyhkdRWLAKqwtlvseepv<br>pgptiqyhkdRWLAKqwtlvseepv<br>.........................<br>.........................<br>.........................<br>.........................	Hs_ENSP00000357040<br>Bt_ENSBTAP00000008613<br>Rn_ENSRNOP00000006849<br>Mm_ENSMUSP00000106895<br>Gg_ENSGALP00000039941<br>Xt_ENSXETP00000012852<br>Dr<br>Ce<br>Dm<br>Sc	NP_065068 Vang-like protein 2 (Van Gogh-like protein 2) (Strabismus 1)(Loop-tail protein 1 homolog)
335	1	CI	ENSP00000363756<br>ENSG00000204222	169<br>-<br>163<br>163<br>-<br>158<br>-<br>-<br>-<br>-	nwaekhcpmaRYVLKtdddvyvnvp<br>.........................<br>nwvnkycpmaRYILKtdddvyvnvp<br>nwvnkycpmaRYILKtdddvyvnvp<br>.........................<br>swmsqrchgaRFLLKtdddvfvntf<br>.........................<br>.........................<br>.........................<br>.........................	Hs_ENSP00000363756<br>Bt<br>Rn_ENSRNOP00000000550<br>Mm_ENSMUSP00000084823<br>Gg<br>Xt_ENSXETP00000043993<br>Dr<br>Ce<br>Dm<br>Sc	NP_003773 Beta-1,3-galactosyltransferase 4 (Beta-1,3-GalTase 4)(Beta3Gal-T4)(b3Gal-T4)(GalT4)(EC 2.4.1.62)(GAL-T2)(Ganglioside galactosyltransferase)(UDP-galactose:beta-N-acetyl-galactosamine-beta-1,3-galactosyltransferase)
336	1	CI	ENSP00000363349<br>ENSG00000148158	158<br>-<br>107<br>158<br>106<br>167<br>153<br>-<br>-<br>104	pthlipplpeKFVVKgvv-drfsee<br>.........................<br>pthlipplpeKFVVKgvv-drfsee<br>pthlipplpeKFVVKgvv-drfsee<br>pthlipplpeKFVVKgvv-drfsee<br>pthfipplpeKFVVKgvv-drfsee<br>pliysvvipitdcqktva-slfssl<br>.........................<br>.........................<br>ptciipplpdkkvfqyiagdrfsqr	Hs_ENSP00000363349<br>Bt<br>Rn_ENSRNOP00000056178<br>Mm_ENSMUSP00000030080<br>Gg_ENSGALP00000025200<br>Xt_ENSXETP00000004753<br>Dr_ENSDARP00000092196<br>Ce<br>Dm<br>Sc_YJL036W	NP_001013012 Sorting nexin-30

337	1	CI	ENSP00000264380<br>ENSG00000115020	1873<br>1880<br>1443<br>1827<br>1875<br>1327<br>-<br>1276<br>1589<br>2055	aafyated-dRFILKqmprlevqsf<br>aafyated-dRFILKqmprlevqsf<br>-------------------------<br>aafyated-dRFILKqmprlevqsf<br>aafyvted-dRFILKqmprlevqsf<br>tqiyivrdndRFILKqmprlevqsf<br>.........................<br>sffyrtqd-dRFVVKqmsrfeiqsf<br>srfcktld-dRFVLKemnsrdmtif<br>sgflktld-dRFIIKelshaeleaf	Hs_ENSP00000264380<br>Bt_ENSBTAP00000002824<br>Rn_ENSRNOP00000020447<br>Mm_ENSMUSP00000109676<br>Gg_ENSGALP00000014354<br>Xt_ENSXETP00000017585<br>Dr<br>Ce_CE42575<br>Dm_FBpp0088952<br>Sc_YFR019W	NP_055855 FYVE finger-containing phosphoinositide kinase (EC 2.7.1.68)(PIKfyve)(1-phosphatidylinositol-4-phosphate 5-kinase)(PtdIns(4)P-5-kinase)(PIP5K)(Phosphatidylinositol-3-phosphate 5-kinase type III)(p235)
338	1	CI	ENSP00000326342<br>ENSG00000179387	19<br>0<br>19<br>19<br>19<br>19<br>27<br>-<br>20<br>-	fyghffrfwmKWLLRqmtg----kc<br>-------------------------<br>fyghffrfwmKWVLRqmtg----kc<br>fyghffrfwmKWLLRqmtg----kc<br>lyssyfrfwlkwlwrlltg----kc<br>fysnyfrfwlKWVLRqisg----kc<br>iylavflfllsallvevvpsaghgc<br>.........................<br>fifsyirpfikwflhaftr----ls<br>.........................	Hs_ENSP00000326342<br>Bt_ENSBTAP00000018612<br>Rn_ENSRNOP00000037996<br>Mm_ENSMUSP00000052287<br>Gg_ENSGALP00000015972<br>Xt_ENSXETP00000032628<br>Dr_ENSDARP00000068704<br>Ce<br>Dm_FBpp0081128<br>Sc	NP_714913 ELMO domain-containing protein 2
339	1	CI	ENSP00000264381<br>ENSG00000114200	159<br>159<br>160<br>160<br>159<br>160<br>-<br>-<br>203<br>-	gtsslhvydgKFLARvervivvsmn<br>gtsslhvydgKFLARvervivvsmn<br>gtsslpvydgkfltrvervivvsmn<br>gtsslpvydgKFLARvervivvsmn<br>gstslpvydgKFLARvervivvsmn<br>gtssldlydgKFLARtervivvsmn<br>.........................<br>.........................<br>gsatldiynadimaavgnvivasfq<br>.........................	Hs_ENSP00000264381<br>Bt_ENSBTAP00000014794<br>Rn_ENSRNOP00000013279<br>Mm_ENSMUSP00000029367<br>Gg_ENSGALP00000015414<br>Xt_ENSXETP00000014224<br>Dr<br>Ce<br>Dm_FBpp0082248<br>Sc	NP_000046 Cholinesterase Precursor (EC 3.1.1.8) (Acylcholine acylhydrolase) (Choline esterase II) (Butyrylcholine esterase) (Pseudocholinesterase)
340	1	CI	ENSP00000378533<br>ENSG00000131437	170<br>142<br>141<br>142<br>145<br>142<br>138<br>133<br>149<br>173	ghiakaegdtRFLVRvsyleiynee<br>ghiakaegdtRFLVRvsyleiynee<br>ghiakaegdtRFLVRvsyleiynee<br>ghiakaegdtRFLVRvsyleiynee<br>ghiakaegdtRFLVRvsyleiynee<br>ghiakaegdtRFLVRvsyleiynee<br>ghiakaegdtRFLVRvsyleiynee<br>dhiakcqhdttflvrvsyleiynee<br>ghiakakenqKFLVRvsymeiynee<br>dtlelqqnd--yvvkcsfielynee	Hs_ENSP00000378533<br>Bt_ENSBTAP00000035688<br>Rn_ENSRNOP00000009999<br>Mm_ENSMUSP00000113848<br>Gg_ENSGALP00000011091<br>Xt_ENSXETP00000009025<br>Dr_ENSDARP00000096875<br>Ce_CE26143<br>Dm_FBpp0076861<br>Sc_YEL061C	Kinesin-like protein KIF3A (Microtubule plus end-directed kinesin motor 3A)
341	1	CI	ENSP00000368950<br>ENSG00000158019	172<br>172<br>205<br>209<br>172<br>172<br>-<br>-<br>-<br>-	knnwtgefsaRFLLKlpvdfsnipt<br>knnwtgefsaRFLLKlpvdfsnipt<br>knnwtgefsaRFLLKlpvdfsnipt<br>knnwtgefsaRFLLKlpvdfsnipt<br>knnwtgefsaRFLLKlpvdfsnipt<br>knnwtgefsaRFLLKlpvdfsnipi<br>.........................<br>.........................<br>.........................<br>.........................	Hs_ENSP00000368950<br>Bt_ENSBTAP00000041902<br>Rn_ENSRNOP00000005855<br>Mm_ENSMUSP00000069133<br>Gg_ENSGALP00000016287<br>Xt_ENSXETP00000022485<br>Dr<br>Ce<br>Dm<br>Sc	NP_004890 Protein BRE (Brain and reproductive organ-expressed protein)(BRCA1/BRCA2-containing complex subunit 45)
342	1	CI	ENSP00000258081<br>ENSG00000115307	63<br>63<br>92<br>92<br>60<br>-<br>60<br>71<br>-<br>-	calpd-svlrRFVVRtmcavlglva<br>calpd-svfrRFVVRtmcavlglva<br>calpd-svlrRFVVRtmcavlglva<br>calpd-svlrRFVVRtmcavlglva<br>cilpd-gslrrlivrvmcsvlglfv<br>.........................<br>calpd-sivrRFIVRimcsvlglhv<br>cllrksaalrmhtlrimcsilgivv<br>.........................<br>.........................	Hs_ENSP00000258081<br>Bt_ENSBTAP00000026802<br>Rn_ENSRNOP00000010602<br>Mm_ENSMUSP00000090281<br>Gg_ENSGALP00000040529<br>Xt<br>Dr_ENSDARP00000054866<br>Ce_CE24973<br>Dm<br>Sc	Ancient ubiquitous protein 1 Precursor
343	1	CI	ENSP00000326348<br>ENSG00000215155	88<br>78<br>115<br>115<br>91<br>100<br>94<br>-<br>-<br>-	lrsytvpqeqRYIIRlllivpiyaf<br>lrsytvpheqRYIIRllfivpvyaf<br>lrsytvpreqRFVIRllfivpiyaf<br>lrsytapreqRFVIRllfivpiyaf<br>lrnytipkeqRYIIRilfivpiyaf<br>lrnytmpneqRYIIRilfivpiysf<br>lrsytvpneqRYIIRilfivpiyaf<br>.........................<br>.........................<br>.........................	Hs_ENSP00000326348<br>Bt_ENSBTAP00000006135<br>Rn_ENSRNOP00000001718<br>Mm_ENSMUSP00000035399<br>Gg_ENSGALP00000006671<br>Xt_ENSXETP00000039298<br>Dr_ENSDARP00000055153<br>Ce<br>Dm<br>Sc	NP_001091089 Transmembrane protein 184A

344	1	CI	ENSP00000285094<br>ENSG00000154822	544<br>544<br>642<br>671<br>564<br>583<br>545<br>-<br>-<br>-	npgdfvnynkRFLARvfpspmrids<br>npgdfvnynkRFLARvfpspmrids<br>npgdfvnynkRFLARvfpspmrids<br>npgdfvnynkRFLARvfpspmrids<br>npgdfvnynkRFLARvfpspmrids<br>npgdfvnynkrylsrifpspmrids<br>fpedfvsynkrflsrvyptpmrida<br>.........................<br>.........................<br>.........................	Hs_ENSP00000285094<br>Bt_ENSBTAP00000013117<br>Rn_ENSRNOP00000018146<br>Mm_ENSMUSP00000046584<br>Gg_ENSGALP00000018326<br>Xt_ENSXETP00000019835<br>Dr_ENSDARP00000085109<br>Ce<br>Dm<br>Sc	NP_055999 Inactive phospholipase C-like protein 2 (Phospholipase C-L2)(PLC-L(2))(PLC-L2)(Phospholipase C epsilon 2)
345	1	CI	ENSP00000316357<br>ENSG00000124486	418<br>418<br>418<br>418<br>418<br>419<br>418<br>-<br>497<br>-	qyveklekilRFVIKekaltlqdld<br>qyveklekilRFVIKekaltlqdld<br>qyveklekilRFVIKekaltlqdld<br>qyveklekilRFVIKekaltlqdld<br>qyveklekilRFVIKekaltlqdld<br>qyveklekilRFVIKekaltlqdld<br>qyveklekilRFVIKekaltlqdld<br>.........................<br>qyveklekiiRFLIKeqaltlddld<br>.........................	Hs_ENSP00000316357<br>Bt_ENSBTAP00000047095<br>Rn_ENSRNOP00000004814<br>Mm_ENSMUSP00000111126<br>Gg_ENSGALP00000035868<br>Xt_ENSXETP00000054783<br>Dr_ENSDARP00000026588<br>Ce<br>Dm_FBpp0085202<br>Sc	NP_001034679 Probable ubiquitin carboxyl-terminal hydrolase FAF-X (EC 3.1.2.15)(Ubiquitin thioesterase FAF-X)(Ubiquitin-specific-processing protease FAF-X)(Deubiquitinating enzyme FAF-X)(Fat facets protein-related, X-linked)(Ubiquitin-specific protease 9, X chromosome)
345	2	CI	ENSP00000316357<br>ENSG00000124486	662<br>662<br>662<br>662<br>662<br>663<br>662<br>-<br>738<br>-	qevqerlnflRFLLKdgqlwlcapq<br>qevqerlnflRFLLKdgqlwlcapq<br>qevqerlnflRFLLKdgqlwlcapq<br>qevqerlnflRFLLKdgqlwlcapq<br>qevqerlnflRFLLKdgqlwlcapq<br>qevqerlnflRFLLKdgqlwlcapq<br>qevqerlnflRFLLKdgqlwlcapq<br>.........................<br>vqiaerleflKFLLKdgqlwlcadq<br>.........................	Hs_ENSP00000316357<br>Bt_ENSBTAP00000047095<br>Rn_ENSRNOP00000004814<br>Mm_ENSMUSP00000111126<br>Gg_ENSGALP00000035868<br>Xt_ENSXETP00000054783<br>Dr_ENSDARP00000026588<br>Ce<br>Dm_FBpp0085202<br>Sc	NP_001034679 Probable ubiquitin carboxyl-terminal hydrolase FAF-X (EC 3.1.2.15)(Ubiquitin thioesterase FAF-X)(Ubiquitin-specific-processing protease FAF-X)(Deubiquitinating enzyme FAF-X)(Fat facets protein-related, X-linked)(Ubiquitin-specific protease 9, X chromosome)
346	1	CI	ENSP00000258455<br>ENSG00000135972	198<br>198<br>192<br>192<br>150<br>157<br>187<br>174<br>197<br>-	ktvtrdvigsRWLIKeeleemlvek<br>kskskdligsRWLIKeeleemlvek<br>-tesrdligsRWLIKeeleemlvek<br>-aesrdligsRWLIKeeleemlvek<br>qkekknlvgsrwltktelegmllek<br>ntkqmdligsRWLIKeeleemlvek<br>dtkqislsgsRWLLKeemeeilven<br>dlepanlsgsqwvtadklkkklsek<br>enqkleiagfqllpkdqlelllves<br>.........................	Hs_ENSP00000258455<br>Bt_ENSBTAP00000008093<br>Rn_ENSRNOP00000021899<br>Mm_ENSMUSP00000056855<br>Gg_ENSGALP00000027063<br>Xt_ENSXETP00000005503<br>Dr_ENSDARP00000082215<br>Ce_CE24890<br>Dm_FBpp0081263<br>Sc	NP_872578 28S ribosomal protein S9, mitochondrial Precursor (S9mt)(MRP-S9)
347	1	C	ENSP00000364815<br>ENSG00000204394	958<br>960<br>967<br>957<br>-<br>873<br>389<br>738<br>750<br>799	hfcnklwnatKFALRglgkgfvpsp<br>hfcnklwnatKFALRglgkgfvpsp<br>hfcnklwnatKFALRglgkgfvpsp<br>hfcnklwnatKFALRglgkgfvpsa<br>.........................<br>hfcnkiwnatkfamkglgdnysppd<br>hfcnklwnavkfamrtlgegfvpce<br>kfcnklwqvvrfalarisdkpeqkp<br>ffcnklwnatkfallyftgsekfdt<br>kfcnkiyqatkfalmrlgddyqppa	Hs_ENSP00000364815<br>Bt_ENSBTAP00000007404<br>Rn_ENSRNOP00000001160<br>Mm_ENSMUSP00000084572<br>Gg<br>Xt_ENSXETP00000051937<br>Dr_ENSDARP00000018411<br>Ce_CE24685<br>Dm_FBpp0086847<br>Sc_YGR094W	NP_006286 Valyl-tRNA synthetase (EC 6.1.1.9)(Valine--tRNA ligase)(ValRS)(Protein G7a)
348	1	CI	ENSP00000337843<br>ENSG00000117640	284<br>279<br>271<br>276<br>277<br>307<br>297<br>-<br>-<br>-	avlisevlrrKFALKeedisrkgn-<br>avlisevlrrKFALKeedisrkgn-<br>avlisevlrrKFALKeedisrkgn-<br>avlisevlrrKFALKeedisrkgn-<br>avliaevlrrKFALKeedlslkek-<br>vnlisevlrqkfalcdpdsvkne--<br>aslisealrkKFVLKdddismrk--<br>.........................<br>.........................<br>.........................	Hs_ENSP00000337843<br>Bt_ENSBTAP00000040498<br>Rn_ENSRNOP00000022908<br>Mm_ENSMUSP00000099609<br>Gg_ENSGALP00000001472<br>Xt_ENSXETP00000007980<br>Dr_ENSDARP00000018738<br>Ce<br>Dm<br>Sc	Protein FAM54B
349	1	CI	ENSP00000354451<br>ENSG00000183856	1145<br>1149<br>1133<br>1146<br>-<br>1137<br>-<br>-	ssvdqipygmRYVAKvlkatlaekf<br>ssvdqipygmRYVAKvlkttlaekf<br>ssvdhipygirymakvlkttletkf<br>ssvdhipygmrymakvlkttlekkf<br>.........................<br>anvdqipygmRYVAKvlknslsqkf<br>.........................<br>.........................	Hs_ENSP00000354451<br>Bt_ENSBTAP00000009046<br>Rn_ENSRNOP00000029108<br>Mm_ENSMUSP00000071715<br>Gg<br>Xt_ENSXETP00000043986<br>Dr<br>Ce	NP_839943 Ras GTPase-activating-like protein IQGAP3

				-<br>1002	.........................<br>rkvhtipveirylctkifcyaadkn	Dm<br>Sc_YPL242C	
350	1	CI	ENSP00000258416<br>ENSG00000135930	134<br>134<br>128<br>134<br>143<br>134<br>133<br>110<br>124<br>-	weddanknggKWIIRlrkglasrcw<br>weddanknggKWIIRlrkglasrcw<br>weddanknggKWIIRlrkglasrcw<br>weddanknggKWIIRlrkglasrcw<br>weddanknggKWIIRlrkglasrcw<br>weddanknggKWIIRlrkglasrcw<br>weddanksggKWIIRlrkglasrcw<br>wedpanckggKWIIRlkkglstriw<br>wedpanskggqwlirlrknkvdraw<br>.........................	Hs_ENSP00000258416<br>Bt_ENSBTAP00000049866<br>Rn_ENSRNOP00000026646<br>Mm_ENSMUSP00000053403<br>Gg_ENSGALP00000012845<br>Xt_ENSXETP00000032783<br>Dr_ENSDARP00000056326<br>Ce_CE07931<br>Dm_FBpp0289702<br>Sc	NP_004837 Eukaryotic translation initiation factor 4E type 2 (eIF-4E type 2)(eIF4E type 2)(mRNA cap-binding protein type 3)(Eukaryotic translation initiation factor 4E-like 3)(Eukaryotic translation initiation factor 4E homologous protein)(mRNA cap-binding protein 4EHP)(eIF4E-like protein 4E-LP)
351	1	C	ENSP00000354821<br>ENSG00000138399	638<br>638<br>623<br>624<br>622<br>624<br>625<br>-<br>709<br>-	edllkaifniKFLARldsqleils-<br>edllkaifniKFLARldsqleliy-<br>edllkkmfnieflarldsqleilp-<br>edllkkmfnieflarldsqleilp-<br>palirtifdvsflsrldaqlevls-<br>eelikavfnvdflgrldaqletfs-<br>eemireifsvdflakldahletfp-<br>.........................<br>eqlinqvfcvkfiqriedeiqvcys<br>.........................	Hs_ENSP00000354821<br>Bt_ENSBTAP00000014228<br>Rn_ENSRNOP00000029035<br>Mm_ENSMUSP00000072896<br>Gg_ENSGALP00000015983<br>Xt_ENSXETP00000041066<br>Dr_ENSDARP00000073017<br>Ce<br>Dm_FBpp0078813<br>Sc	NP_078898 FAST kinase domain-containing protein 1
352	1	C	ENSP00000327072<br>ENSG00000143498	350<br>350<br>264<br>351<br>352<br>343<br>345<br>-<br>-<br>-	agctknitawKYLAKylknilmgn-<br>agctknitawKYLAKylkqtlmgs-<br>sgctknitawKYLAKylkqtlmes-<br>agcnknitawkylsrivrkvrlrs-<br>agwkknvrawsclarqvkqivisek<br>sgwgysmeawrqigkrlktslkrn-<br>sswktdirawdclltclkkirkkk-<br>.........................<br>.........................<br>.........................	Hs_ENSP00000327072<br>Bt_ENSBTAP00000017928<br>Rn_ENSRNOP00000031942<br>Mm_ENSMUSP00000094808<br>Gg_ENSGALP00000015355<br>Xt_ENSXETP00000032902<br>Dr_ENSDARP00000090955<br>Ce<br>Dm<br>Sc	NP_647603 TATA box-binding protein-associated factor RNA polymerase I subunit A (TATA box-binding protein-associated factor 1A)(TBP-associated factor 1A)(TBP-associated factor RNA polymerase I 48 kDa)(TAFI48)(Transcription factor SL1)
352	2	C	ENSP00000327072<br>ENSG00000143498	419<br>419<br>333<br>420<br>422<br>412<br>416<br>-<br>-<br>-	lgkg--cryfRYILKqdhqilgkki<br>lgkg--ckyfryiskqdhqalkkkm<br>lgkg--cryfRYILKqdhetlkkki<br>lgkg--ckyfRYILKqdhetlkkki<br>lgkd--ckyfkyvshqgckaqvkkf<br>lgpg--cayykavcrlgrktkmkkv<br>inenrmreycrvaaiaekevllqir<br>.........................<br>.........................<br>.........................	Hs_ENSP00000327072<br>Bt_ENSBTAP00000017928<br>Rn_ENSRNOP00000031942<br>Mm_ENSMUSP00000094808<br>Gg_ENSGALP00000015355<br>Xt_ENSXETP00000032902<br>Dr_ENSDARP00000090955<br>Ce<br>Dm<br>Sc	NP_647603 TATA box-binding protein-associated factor RNA polymerase I subunit A (TATA box-binding protein-associated factor 1A)(TBP-associated factor 1A)(TBP-associated factor RNA polymerase I 48 kDa)(TAFI48)(Transcription factor SL1)
353	1	CI	ENSP00000385158<br>ENSG00000170264	326<br>326<br>238<br>240<br>322<br>319<br>-<br>-<br>-<br>-	eallasqkpfKFIAReeqkraarek<br>eallasqkpfKFVAReeqkqavrek<br>aallasqkpfKFIAReeqkqairek<br>aallaslkpfKFIAReeqkqavrek<br>lrllasqkpfkfierekqrdemrkm<br>dillatqkpfqfiereerkktlr-l<br>.........................<br>.........................<br>.........................<br>.........................	Hs_ENSP00000385158<br>Bt_ENSBTAP00000012239<br>Rn_ENSRNOP00000013097<br>Mm_ENSMUSP00000105184<br>Gg_ENSGALP00000014303<br>Xt_ENSXETP00000004758<br>Dr<br>Ce<br>Dm<br>Sc	UPF0564 protein FAM161A
354	1	C	ENSP00000234701<br>ENSG00000016490	787<br>785<br>787<br>787<br>-<br>790<br>-<br>-<br>-<br>-	gddydhgtahKYIIRistsildlrd<br>gddydhgradKYIIRistnilelrd<br>gddydhgrassyiiristsivdlrn<br>gddydhgrasnyiirmstsivdlrd<br>.........................<br>gddldqgtvsdydlrmstspeelrr<br>.........................<br>.........................<br>.........................<br>.........................	Hs_ENSP00000234701<br>Bt_ENSBTAP00000010730<br>Rn_ENSRNOP00000018373<br>Mm_ENSMUSP00000029919<br>Gg<br>Xt_ENSXETP00000001090<br>Dr<br>Ce<br>Dm<br>Sc	NP_001276 Calcium-activated chloride channel regulator 1 Precursor (Calcium-activated chloride channel family member 1)(hCLCA1)(Calcium-activated chloride channel protein 1)(CaCC-1)(hCaCC-1)
355	1	C	ENSP00000348564<br>ENSG00000140326	20<br>20<br>0<br>-<br>20<br>-<br>0	reevsvaavvRWIARstqgsednag<br>keelsvaaavr-iarsaqsseddpr<br>-------------------------<br>.........................<br>seevavgavvrwlrrap-gqrppeg<br>.........................<br>-------------------------	Hs_ENSP00000348564<br>Bt_ENSBTAP00000007561<br>Rn_ENSRNOP00000014664<br>Mm<br>Gg_ENSGALP00000014899<br>Xt<br>Dr_ENSDARP00000061960	NP_612486 Codanin-1

				-<br>25<br>-	.........................<br>peerhllqllaqlekvphhqleqkf<br>.........................	Ce<br>Dm_FBpp0072673<br>Sc	
356	1	CI	ENSP00000361894<br>ENSG00000124713	93<br>95<br>93<br>93<br>54<br>-<br>93<br>-<br>96<br>-	tsvdasdkmlKYALKerwnrrhepa<br>tsvdasdkmlKYALKerwnrrhdpa<br>tsvdasdkmlKYALKerwnrrkepa<br>msvdasdkmlKYALKerwnrrkeps<br>tsgdasdkmlKYALKerwerrkeep<br>.........................<br>vsvdasdkmlKYALKerwerrketa<br>.........................<br>vsvdasdkmlKYALKerwarrneaa<br>.........................	Hs_ENSP00000361894<br>Bt_ENSBTAP00000007273<br>Rn_ENSRNOP00000022307<br>Mm_ENSMUSP00000002846<br>Gg_ENSGALP00000013950<br>Xt<br>Dr_ENSDARP00000007584<br>Ce<br>Dm_FBpp0082120<br>Sc	NP_061833 Glycine N-methyltransferase (EC 2.1.1.20)
357	1	CI	ENSP00000359691<br>ENSG00000146166	185<br>250<br>237<br>239<br>475<br>120<br>349<br>-<br>-<br>-	vtgeplltspRYIAKrqlshlqasg<br>vtgeplltsprhiakrqlsqlqdag<br>vtgeplltspRYIAKrqlrqlqdag<br>vtgeplltspRYIAKrqlrqlqdag<br>vfgnplmtsprhiakkqlselqdng<br>imgdslltypRYLAKqllmqlqesg<br>itgvplrtsprliakmmlgqlqsmg<br>.........................<br>.........................<br>.........................	Hs_ENSP00000359691<br>Bt_ENSBTAP00000035459<br>Rn_ENSRNOP00000016316<br>Mm_ENSMUSP00000059871<br>Gg_ENSGALP00000036230<br>Xt_ENSXETP00000009680<br>Dr_ENSDARP00000025002<br>Ce<br>Dm<br>Sc	NP_057655 Glutamate--ammonia ligase domain-containing protein 1 (Lens glutamine synthase-like) (Lengsin)
358	1	C	ENSP00000369607<br>ENSG00000073910	15<br>0<br>15<br>0<br>14<br>-<br>14<br>-<br>220<br>182	qdsgffeisiKYLLKswsntspvgn<br>-------------------------<br>qdsgffeisiKYLLKswsnaspvgn<br>------------------saspvgn<br>ndrgpffislnfalknqlsaspvgn<br>.........................<br>qdsgffeisiksllkswgspspig-<br>.........................<br>qssavtyqslpvsrqsyasppalii<br>kfiryaenklnmclqqldmaeppiv	Hs_ENSP00000369607<br>Bt_ENSBTAP00000035604<br>Rn_ENSRNOP00000045830<br>Mm_ENSMUSP00000084454<br>Gg_ENSGALP00000027526<br>Xt<br>Dr_ENSDARP00000044293<br>Ce<br>Dm_FBpp0076146<br>Sc_YIL129C	Protein furry homolog
359	1	C	ENSP00000334437<br>ENSG00000186364	283<br>284<br>279<br>301<br>-<br>282<br>283<br>-<br>-<br>-	-dkervstgtKFALKlwlqhlgrtp<br>-srervstgtkfaltlwlqhlgrks<br>edkervstgtKFALKlwlqhlgr--<br>edkerigagtkfalqlwlqhlgr--<br>.........................<br>-averistgtkyalslwletlpkap<br>-diervstgtkfalelwlktlehha<br>.........................<br>.........................<br>.........................	Hs_ENSP00000334437<br>Bt_ENSBTAP00000028830<br>Rn_ENSRNOP00000031407<br>Mm_ENSMUSP00000029742<br>Gg<br>Xt_ENSXETP00000040431<br>Dr_ENSDARP00000006375<br>Ce<br>Dm<br>Sc	NP_001012776 Putative nucleoside diphosphate-linked moiety X motif 17, mitochondrial Precursor (Nudix motif 17)(EC 3.6.1.-)
360	1	CI	ENSP00000324892<br>ENSG00000174437	984<br>984<br>986<br>984<br>984<br>983<br>977<br>-<br>985<br>-	lpvilmdetlKFVARnyle-pgkec<br>lpvilmdetlKFVARnyle-pgkec<br>lpvilmdetlKFVARnyle-pgkec<br>lpvilmdetlKFVARnyleqpgkec<br>lpvilldetlKYVARnyle-pgkds<br>lpvilldetlKYVARnyle-pgkec<br>lpvilldevlkfaarnyld-kpkdl<br>.........................<br>ipvvlldetlKFVARkiad--ges-<br>.........................	Hs_ENSP00000324892<br>Bt_ENSBTAP00000001838<br>Rn_ENSRNOP00000024347<br>Mm_ENSMUSP00000031423<br>Gg_ENSGALP00000038523<br>Xt_ENSXETP00000045259<br>Dr_ENSDARP00000047200<br>Ce<br>Dm_FBpp0072121<br>Sc	NP_733765 Sarcoplasmic/endoplasmic reticulum calcium ATPase 2 (SERCA2)(EC 3.6.3.8)(Calcium pump 2)(Calcium-transporting ATPase sarcoplasmic reticulum type, slow twitch skeletal muscle isoform)(SR Ca(2+)-ATPase 2) (Endoplasmic reticulum class 1/2 Ca(2+) ATPase)
361	1	CI	ENSP00000320754<br>ENSG00000130413	141<br>141<br>137<br>136<br>-<br>-<br>73<br>-<br>-<br>-	ieatdketetKWAIKkvnkekagss<br>ieaidkeretKWAIKkvnkekagss<br>ieatdketgaKWAIKkvnkekagss<br>feaidketgaKWAIKkvnkekagss<br>.........................<br>.........................<br>ceathietqrKWAIKkvnkekagts<br>.........................<br>.........................<br>.........................	Hs_ENSP00000320754<br>Bt_ENSBTAP00000042938<br>Rn_ENSRNOP00000019597<br>Mm_ENSMUSP00000102356<br>Gg<br>Xt<br>Dr_ENSDARP00000091419<br>Ce<br>Dm<br>Sc	NP_112168 Serine/threonine-protein kinase 33 (EC 2.7.11.1)
362	1	CI	ENSP00000262811<br>ENSG00000099308	392<br>-<br>-<br>414<br>219	ylvrhrdtrqRFAIKkinkqnlilr<br>.........................<br>.........................<br>ylvrhrdtrqRFAIKkinkqnlilr<br>ylvrhretrqRFALKkinkqnlilr	Hs_ENSP00000262811<br>Bt<br>Rn<br>Mm_ENSMUSP00000046075<br>Gg_ENSGALP00000005525	NP_055831 Microtubule-associated serine/threonine-protein kinase 3 (EC 2.7.11.1)

				424<br>-<br>-<br>-<br>-	ylvrhkemrqrfamkkinkqnlilr<br>.........................<br>.........................<br>.........................<br>.........................	Xt_ENSXETP00000028316<br>Dr<br>Ce<br>Dm<br>Sc	
363	1	CI	ENSP00000351416<br>ENSG00000132466	1403<br>1272<br>1393<br>1399<br>-<br>1289<br>-<br>1541<br>-<br>-	afrkghvkvvRYLVKevnqfpsdse<br>afrkghvkvvRYLVKevnqfpsdse<br>afrkghvkvvRYLVKevnqfpsdse<br>afrkghvkvvRYLVKevnqfpsdse<br>.........................<br>flfqghvkvvRYLVKevnqfpsdse<br>.........................<br>afrkghveivkymvnsakqfpneqd<br>.........................<br>.........................	Hs_ENSP00000351416<br>Bt_ENSBTAP00000006467<br>Rn_ENSRNOP00000032591<br>Mm_ENSMUSP00000014421<br>Gg<br>Xt_ENSXETP00000017818<br>Dr<br>Ce_CE06305<br>Dm<br>Sc	NP_115593 Ankyrin repeat domain-containing protein 17 (Gene trap ankyrin repeat protein) (Serologically defined breast cancer antigen NY-BR-16)
364	1	C	ENSP00000266544<br>ENSG00000139180	299<br>299<br>299<br>299<br>302<br>302<br>296<br>325<br>318<br>-	lpfplplfayRWVARvfeispfepw<br>lpyplphfayrwigrlfeispfepw<br>ipyplprfvyswigrlfglspfepw<br>ipyplplfvyswigklfglspfepw<br>fpyplprplyhliarffeispfepw<br>vpypiprplyrlisrffeinpfepw<br>mpyplprplyhlvarffemnpfepw<br>mpdpyfmaltmatelygkvfkckvp<br>krwgymrydmrwdptfllkaklnsf<br>.........................	Hs_ENSP00000266544<br>Bt_ENSBTAP00000007189<br>Rn_ENSRNOP00000034135<br>Mm_ENSMUSP00000085523<br>Gg_ENSGALP00000036475<br>Xt_ENSXETP00000018561<br>Dr_ENSDARP00000042738<br>Ce_CE26163<br>Dm_FBpp0077859<br>Sc	NP_004993 NADH dehydrogenase
365	1	CI	ENSP00000218006<br>ENSG00000101890	681<br>681<br>681<br>681<br>667<br>575<br>683<br>-<br>-<br>-	lksrncvvdgRFVLKvtdygfndil<br>lksrncvvdgRFVLKvtdygfndil<br>lksrncvvdgRFVLKvtdygfnnil<br>lksrncvvdgRFVLKvtdygfndil<br>lksrncvvdgRFVLKitdygynell<br>lksrncvvdgRFVLKvtdygynevl<br>lksrncvvdgRFVLKitdygyneil<br>.........................<br>.........................<br>.........................	Hs_ENSP00000218006<br>Bt_ENSBTAP00000036727<br>Rn_ENSRNOP00000049500<br>Mm_ENSMUSP00000044521<br>Gg_ENSGALP00000001168<br>Xt_ENSXETP00000003311<br>Dr_ENSDARP00000090688<br>Ce<br>Dm<br>Sc	NP_001513 Retinal guanylyl cyclase 2 Precursor (RETGC-2) (EC 4.6.1.2)(Guanylate cyclase 2F, retinal)(Guanylate cyclase F)(GC-F)(Rod outer segment membrane guanylate cyclase 2)(ROS-GC2)
366	1	CI	ENSP00000362174<br>ENSG00000163874	227<br>224<br>227<br>227<br>193<br>181<br>179<br>-<br>-<br>-	ggkrvvcyddRFIVKlayesdgivv<br>ggkrvvcyddRFIVKlafesdgivv<br>ggkrvvcyddRFIVKlayesdgvvv<br>ggkrvvcyddRFIVKlafesdgvvv<br>ggkrvvcyddRFIVKlahesdgivv<br>ggkrlvcyddRFIVKlaiqcdgviv<br>ggkrvvcyddRFIVKlahdldgiiv<br>.........................<br>.........................<br>.........................	Hs_ENSP00000362174<br>Bt_ENSBTAP00000015041<br>Rn_ENSRNOP00000012314<br>Mm_ENSMUSP00000037172<br>Gg_ENSGALP00000003140<br>Xt_ENSXETP00000025039<br>Dr_ENSDARP00000006113<br>Ce<br>Dm<br>Sc	NP_079355 Zinc finger CCCH domain-containing protein 12A (MCP-induced protein 1)
367	1	CI	ENSP00000262817<br>ENSG00000105696	88<br>91<br>81<br>81<br>20<br>-<br>73<br>-<br>-<br>-	ergcrlfsicRFVARsskpnatqte<br>ergcrlfsicRFVARsskpnatqae<br>ergcrlfsicRFVARssrpnatete<br>ergcrlfsicRFVAKssrpnatete<br>yrgcrlfsichfvdasaglnttrae<br>.........................<br>hrgcrlysicqfvngntgintskee<br>.........................<br>.........................<br>.........................	Hs_ENSP00000262817<br>Bt_ENSBTAP00000010124<br>Rn_ENSRNOP00000029378<br>Mm_ENSMUSP00000043659<br>Gg_ENSGALP00000034392<br>Xt<br>Dr_ENSDARP00000016439<br>Ce<br>Dm<br>Sc	NP_036241 Transmembrane protein 59-like Precursor (Brain-specific membrane-anchored protein)
368	1	CI	ENSP00000256594<br>ENSG00000134202	82<br>83<br>82<br>81<br>-<br>-<br>78<br>-<br>-<br>-	nkitqsnailRYIARkhnmcgetee<br>nrltqsnailRYIARkhnmcgdtee<br>nkitqsnailRYIARkhnmcgdtee<br>nkitqsnailRYIARkhnmcgdtee<br>.........................<br>.........................<br>rkivqsnaimRYIARkhnlcgetee<br>.........................<br>.........................<br>.........................	Hs_ENSP00000256594<br>Bt_ENSBTAP00000002406<br>Rn_ENSRNOP00000025589<br>Mm_ENSMUSP00000004134<br>Gg<br>Xt<br>Dr_ENSDARP00000046181<br>Ce<br>Dm<br>Sc	NP_000840 Glutathione S-transferase Mu 3 (EC 2.5.1.18) (GSTM3-3)(GST class-mu 3) (hGSTM3-3)
369	1	CI	ENSP00000246190<br>ENSG00000125967	143<br>140<br>134<br>134<br>130	raskvdqfvtRFLLRetvsqlqalq<br>raskvdqfvtRFLLRetvsqlqalq<br>qaskvdqfvtRFLLRetanqlqalq<br>qaskvdqfvtRFLLRetvnqlqalq<br>tsskmeqfltRFLLRetmhqlqslq	Hs_ENSP00000246190<br>Bt_ENSBTAP00000005154<br>Rn_ENSRNOP00000022115<br>Mm_ENSMUSP00000000895<br>Gg_ENSGALP00000004831	NP_112509 N-terminal EF-hand calcium-binding protein 3 (Amyloid beta A4 protein-binding family A member 2-binding

				-<br>-<br>-<br>-<br>-	.........................<br>.........................<br>.........................<br>.........................<br>.........................	Xt<br>Dr<br>Ce<br>Dm<br>Sc	protein)(Neuronal calcium-binding protein 3)(X11L-binding protein 51)(Nek2-interacting protein 1)
370	1	CI	ENSP00000290510<br>ENSG00000110811	377<br>319<br>375<br>374<br>351<br>203<br>372<br>-<br>-<br>-	pglgprediqRFILRslgekrqlyy<br>pglgprediqRFVLRslgekrqlyy<br>pdlgpredvqRFILRslgekrqlyy<br>pdlgprediqRFILRslgekrqlyy<br>tavtarenirhyvqrslmekkliyy<br>ggk-preiiatyvhqsllekrllyy<br>qqtgpapeiskyvnralqekkllyf<br>.........................<br>.........................<br>.........................	Hs_ENSP00000290510<br>Bt_ENSBTAP00000037562<br>Rn_ENSRNOP00000021578<br>Mm_ENSMUSP00000023958<br>Gg_ENSGALP00000023344<br>Xt_ENSXETP00000025255<br>Dr_ENSDARP00000075077<br>Ce<br>Dm<br>Sc	NP_055077 Prolyl 3-hydroxylase 3 Precursor (EC 1.14.11.7)(Leprecan-like protein 2)(Protein B)
371	1	CI	ENSP00000383626<br>ENSG00000215738	126<br>-<br>-<br>-<br>-<br>125<br>-<br>-<br>-<br>-	lnwnkddregRFVLKnendaippkk<br>.........................<br>.........................<br>.........................<br>.........................<br>lnwnkddregRFVLKnendifpakk<br>.........................<br>.........................<br>.........................<br>.........................	Hs_ENSP00000383626<br>Bt<br>Rn<br>Mm<br>Gg<br>Xt_ENSXETP00000050352<br>Dr<br>Ce<br>Dm<br>Sc	Putative uncharacterized protein MLLT4 (Uncharacterized protein ENSP00000383625) Fragment
372	1	CI	ENSP00000356655<br>ENSG00000203734	782<br>713<br>-<br>709<br>-<br>-<br>-<br>-<br>-<br>-	wgcptlsevnRYLIRvqdvaqlhcc<br>wgcptlseanRYLIRvqdvaqlhcc<br>.........................<br>cgcptlseanRYLIRtqdvvqlhcc<br>.........................<br>.........................<br>.........................<br>.........................<br>.........................<br>.........................	Hs_ENSP00000356655<br>Bt_ENSBTAP00000038945<br>Rn<br>Mm_ENSMUSP00000093497<br>Gg<br>Xt<br>Dr<br>Ce<br>Dm<br>Sc	NP_001071174 Putative guanine nucleotide exchange factor LFDH (Lung-specific F-box and DH domain-containing protein)
373	1	CI	ENSP00000352925<br>ENSG00000196367	3197<br>3200<br>1879<br>3196<br>3157<br>1878<br>-<br>3332<br>3108<br>3099	crhqnesksrKYLAKvlwllsfddd<br>crhqnesksrKYLAKvlwllsfddd<br>crhqnesksrKYLAKvlwllsfddd<br>crhqnesksrKYLAKvlwllsfddd<br>crhqnesksrKYLAKvlwllsfddd<br>crhqnesksrKYLAKvlwllsfddd<br>.........................<br>arvdndikarkpiakilwlskhlna<br>srnqiesktrKYIAKvlwflsydnn<br>aglyknskirellcrilwlisidda	Hs_ENSP00000352925<br>Bt_ENSBTAP00000009365<br>Rn_ENSRNOP00000031526<br>Mm_ENSMUSP00000091668<br>Gg_ENSGALP00000005678<br>Xt_ENSXETP00000043361<br>Dr<br>Ce_CE36692<br>Dm_FBpp0085431<br>Sc_YHR099W	Transformation/transcription domain-associated protein (350/400 kDa PCAF-associated factor)(PAF350/400)(STAF40)(Tra1 homolog)
374	1	CI	ENSP00000280191<br>ENSG00000150628	116<br>116<br>-<br>115<br>73<br>107<br>78<br>-<br>-<br>-	vkldnwaqleKFLARkkfklpkeli<br>vkldnwaqleKFLARkklklpkeli<br>.........................<br>vkldnwaqieKFLAKkkfklpkeli<br>aklsnwsqlrRFLAKqefdiaqeli<br>nrlsnwsqlekiidkykfnqgrqil<br>akqsnwsqiekffvkqnislvkemi<br>.........................<br>.........................<br>.........................	Hs_ENSP00000280191<br>Bt_ENSBTAP00000046266<br>Rn<br>Mm_ENSMUSP00000033917<br>Gg_ENSGALP00000031560<br>Xt_ENSXETP00000023557<br>Dr_ENSDARP00000052561<br>Ce<br>Dm<br>Sc	NP_653245 Spermatogenesis-associated protein 4 (Testis spermatocyte apoptosis-related gene 2 protein)(Testis and spermatogenesis cell-related protein 2)
375	1	CI	ENSP00000246117<br>ENSG00000125912	374<br>374<br>377<br>374<br>375<br>371<br>367<br>375<br>374<br>-	aedvlaweheRFAIRrlpaftlshl<br>aedmlaweheRFAVRrlpaftlshl<br>addvlaweheRFAIRrlpaftlshl<br>addvlaweheRFAIRrlpaftlshl<br>aedilaweheRFAIRrlpaftishl<br>aedtlaweheRFAIRrlpaftvshl<br>addtlawehe rfgirrlpaftlshl<br>tt-vsawehekfnikrmpaitlstl<br>adtklaweherfsikrypsftlssv<br>.........................	Hs_ENSP00000246117<br>Bt_ENSBTAP00000027665<br>Rn_ENSRNOP00000007110<br>Mm_ENSMUSP00000020463<br>Gg_ENSGALP00000021774<br>Xt_ENSXETP00000031376<br>Dr_ENSDARP00000076240<br>Ce_CE41012<br>Dm_FBpp0079578<br>Sc	NP_064555 Nicalin Precursor (Nicastrin-like protein)
376	1	CI	ENSP00000304429<br>ENSG00000162627	99<br>117<br>99<br>99<br>130	ptliipplpeKFIVKgmverfnddf<br>ptliipplpeKFIVKgmverfnddf<br>ptliipplpeKFIVKgmverfnddf<br>ptliipplpeKFIVKgmverfnddf<br>ptliipplpekfvmkgmverfsdef	Hs_ENSP00000304429<br>Bt_ENSBTAP00000042058<br>Rn_ENSRNOP00000022947<br>Mm_ENSMUSP00000029639<br>Gg_ENSGALP00000008787	NP_057060 Sorting nexin-7

				151<br>-<br>-<br>-<br>-	ptliipplpeKFIVRgmverfteef<br>.........................<br>.........................<br>.........................<br>.........................	Xt_ENSXETP00000018796<br>Dr<br>Ce<br>Dm<br>Sc	
377	1	CI	ENSP00000325136<br>ENSG00000138029	16<br>17<br>17<br>17<br>17<br>14<br>0<br>0<br>12<br>-	ypfknlptasKWALRfsirplscss<br>ytlknlpntsKWALRfcmrplssss<br>stfrnlsttsKWALRfsvrplscss<br>stfrnlsttsKWALRssirplscss<br>htirnlpassawavrvfarslscss<br>qtirslpt----acklssrsiscss<br>-------------------------<br>----------------mlravstsf<br>nvcr---kgnafnllnvarkvqcra<br>.........................	Hs_ENSP00000325136<br>Bt_ENSBTAP00000013310<br>Rn_ENSRNOP00000014637<br>Mm_ENSMUSP00000110434<br>Gg_ENSGALP00000026663<br>Xt_ENSXETP00000006963<br>Dr_ENSDARP00000074190<br>Ce_CE00561<br>Dm_FBpp0072135<br>Sc	NP_000174 Trifunctional enzyme subunit beta, mitochondrial Precursor (TP-beta)
378	1	CI	ENSP00000263956<br>ENSG00000119041	784<br>624<br>781<br>780<br>811<br>813<br>-<br>926<br>757<br>926	gltfihmasqKYVLRrhalivqgfs<br>gltfihmasqKYVLKkfsclmhgfs<br>gltfihmasqKYVLKrhaltvqgfs<br>gltfihmasqKYVLKrhaltvqgfs<br>gltfihmasqKYVLKrhallvqgfs<br>gltfvhmasqKFVLKrhallvqgfs<br>.........................<br>altfthmsckkdlssrhligirgia<br>avafnqlslqkkvlrksasvaqava<br>glshihramqrltaqrhfqifhglr	Hs_ENSP00000263956<br>Bt_ENSBTAP00000010228<br>Rn_ENSRNOP00000002864<br>Mm_ENSMUSP00000039420<br>Gg_ENSGALP00000038586<br>Xt_ENSXETP00000057291<br>Dr<br>Ce_CE29016<br>Dm_FBpp0086164<br>Sc_YGR047C	NP_036218 General transcription factor 3C polypeptide 3 (Transcription factor IIIC subunit gamma)(TF3C-gamma)(TFIIIC 102 kDa subunit)(TFIIIC102)
379	1	CI	ENSP00000377779<br>ENSG00000185518	362<br>375<br>362<br>362<br>384<br>391<br>366<br>-<br>-<br>333	iqsstgtwyqRWLVRfktifkqvwd<br>iqsstgtwyqRWLVRfkttfkqvwe<br>iqsstgtwyqRWLVRfmtifkqvwd<br>iqsstgtwyqRWLVRfmtifkqvwd<br>iqsstgtwyqRWLVRitttfkqvwd<br>iqsstgtwfqrwfvrimtvikqiwe<br>iqtatgtafqRWVVRtltltklvik<br>.........................<br>.........................<br>nhppkasfkd---------------	Hs_ENSP00000377779<br>Bt_ENSBTAP00000038597<br>Rn_ENSRNOP00000015084<br>Mm_ENSMUSP00000082254<br>Gg_ENSGALP00000011089<br>Xt_ENSXETP00000016588<br>Dr_ENSDARP00000080251<br>Ce<br>Dm<br>Sc_YML123C	NP_055663 Synaptic vesicle glycoprotein 2B
380	1	CI	ENSP00000299853<br>ENSG00000058600	364<br>364<br>364<br>364<br>366<br>363<br>367<br>399<br>376<br>-	dfvmwkftqsRWVVRkevatvtklc<br>hchmwkftqsRWVVRkevaavtklc<br>dfvmwkftqsRWVVRkevaavtklc<br>dfvmwkftqsRWVVRkevaavtklc<br>dfvmwkftqdRWVVRkevaavtklc<br>dfvmwrftksRWVVRkeiaavtklc<br>dfvmwrftiertlmrkevaaiiklp<br>dlalclidgghrvtrltlmtcfkls<br>dyilfrfsrtpylfraqvmaatqlp<br>.........................	Hs_ENSP00000299853<br>Bt_ENSBTAP00000043258<br>Rn_ENSRNOP00000046447<br>Mm_ENSMUSP00000033173<br>Gg_ENSGALP00000011329<br>Xt_ENSXETP00000036356<br>Dr_ENSDARP00000096063<br>Ce_CE01426<br>Dm_FBpp0077946<br>Sc	NP_060589 DNA-directed RNA polymerase III subunit RPC5 (RNA polymerase III subunit C5)(DNA-directed RNA polymerase III 80 kDa polypeptide)
381	1	CI	ENSP00000347721<br>ENSG00000182557	203<br>206<br>207<br>207<br>207<br>-<br>-<br>-<br>-<br>-	savtmltgnwRWALRvmpcleaval<br>savaeltgnwRWALRimpcleaval<br>stvaeltgnwRWALRimpcldaval<br>stvaeltgnwRWALRlmpcldamal<br>ssmahvtgdwhwafrvtpcmgglal<br>.........................<br>.........................<br>.........................<br>.........................<br>.........................	Hs_ENSP00000347721<br>Bt_ENSBTAP00000034997<br>Rn_ENSRNOP00000021425<br>Mm_ENSMUSP00000090617<br>Gg_ENSGALP00000002144<br>Xt<br>Dr<br>Ce<br>Dm<br>Sc	NP_872344 Protein spinster homolog 3
382	1	CI	ENSP00000264276<br>ENSG00000003393	1370<br>-<br>1359<br>1364<br>1364<br>1343<br>-<br>-<br>1226<br>-	vekydd--irKYLIKacdtplhplg<br>.........................<br>vekydd--ikKYLIKacdtplhplg<br>vekydd--ikKYLIKacdtplhplg<br>lekyen--ikKYLIKacdtplhplg<br>mdkydk--ikmylinacetpvhplg<br>.........................<br>.........................<br>lteqdvtsirlyleqafkdrhhply<br>.........................	Hs_ENSP00000264276<br>Bt<br>Rn_ENSRNOP00000036116<br>Mm_ENSMUSP00000027178<br>Gg_ENSGALP00000013692<br>Xt_ENSXETP00000027118<br>Dr<br>Ce<br>Dm_FBpp0078116<br>Sc	NP_065970 Alsin (Amyotrophic lateral sclerosis protein 2)(Amyotrophic lateral sclerosis 2 chromosomal region candidate gene 6 protein)
383	1	CI	ENSP00000378879<br>ENSG00000196296	1048<br>990<br>-<br>985<br>-	lpvigldeilKFVARnyleg-----<br>lpvigldeilKFVARnyledpeder<br>.........................<br>lpvigldellKFIARnyleg-----<br>.........................	Hs_ENSP00000378879<br>Bt_ENSBTAP00000019704<br>Rn<br>Mm_ENSMUSP00000032974<br>Gg	NP_004311 Sarcoplasmic/endoplasmic reticulum calcium ATPase 1 (SERCA1)(EC 3.6.3.8)(Calcium

				985<br>985<br>986<br>-<br>-	ipvilldellKFVARnylegks---<br>fpvilidealKFVARnyleekdqnl<br>lpvllldeilKFIARnyidgkpetv<br>.........................<br>.........................	Xt_ENSXETP00000021343<br>Dr_ENSDARP00000043931<br>Ce_CE18884<br>Dm<br>Sc	pump 1)(Calcium-transporting ATPase sarcoplasmic reticulum type, fast twitch skeletal muscle isoform)(SR Ca(2+)-ATPase 1)(Endoplasmic reticulum class 1/2 Ca(2+) ATPase)
384	1	CI	ENSP00000264649<br>ENSG00000033627	129<br>129<br>129<br>129<br>129<br>129<br>129<br>141<br>129<br>142	krnfleltelKFILRktqqffdeae<br>krnfleltelKFILRktqqffde--<br>krnfleltelKFILRktqqffdeae<br>krnfleltelKFILRktqqffdeae<br>krnfleltelKFILRktqqffde--<br>krnfleltelKFILRktqqffde--<br>kknfleltelkhilrrtqqffde--<br>kknfseltelkhilrktqtffeevd<br>krnfleltelkhilrktqvffdesv<br>evqkndleqyrfilqsgdefflkgd	Hs_ENSP00000264649<br>Bt_ENSBTAP00000025588<br>Rn_ENSRNOP00000052113<br>Mm_ENSMUSP00000099399<br>Gg_ENSGALP00000005145<br>Xt_ENSXETP00000016620<br>Dr_ENSDARP00000041713<br>Ce_CE30573<br>Dm_FBpp0084743<br>Sc_YOR270C	NP_001123492 V-type proton ATPase 116 kDa subunit a isoform 1 (V-ATPase 116 kDa isoform a1)(Vacuolar proton translocating ATPase 116 kDa subunit a isoform 1)(Clathrin-coated vesicle/synaptic vesicle proton pump 116 kDa subunit)(Vacuolar proton pump subunit 1)(Vacuolar adenosine triphosphatase subunit Ac116)
385	1	CI	ENSP00000385083<br>ENSG00000119537	190<br>189<br>190<br>190<br>190<br>190<br>190<br>-<br>193<br>184	lfgftaysasKFAIRglaealqmev<br>lfgytaysssKFALRglaealqmev<br>lfgftaysssKFAIRglaealqmev<br>lfgftaysssKFAIRglaealqmev<br>lfgytaysptKFALRglaealqmev<br>lfgytaysptKFALRglaealqmev<br>lfgytayspsKFALRglaealqmem<br>.........................<br>iygygpysatKYALRamaetiames<br>fvgysqyapakaaikslvailrqel	Hs_ENSP00000385083<br>Bt_ENSBTAP00000010155<br>Rn_ENSRNOP00000003728<br>Mm_ENSMUSP00000010049<br>Gg_ENSGALP00000036388<br>Xt_ENSXETP00000052055<br>Dr_ENSDARP00000009670<br>Ce<br>Dm_FBpp0084241<br>Sc_YBR265W	NP_002026 3-ketodihydrosphingosine reductase Precursor (KDS reductase)(EC 1.1.1.102)(3-dehydrosphinganine reductase)(Follicular variant translocation protein 1)(FVT-1)
386	1	C	ENSP00000275730<br>ENSG00000146828	515<br>515<br>-<br>515<br>110<br>229<br>521<br>568<br>546<br>525	sqallfhqvrKYLLRldvrkdhvkf<br>sqallfhqvrKYLLRldvrkdhvkf<br>.........................<br>sqallfhqvrKYLLRldvrkehvkf<br>sqalifhqvrkyllmldvrkdhvkf<br>sqalifhqvrkyllLldvrkdhvkf<br>sqalifhqvrkyllmldsrkdhvkf<br>sqaliyhqvrkyllLldvrkehvky<br>sqalmfhqvrkyllmldprkdhvkf<br>sqsliyhqvrKYLLRlr--qdniky	Hs_ENSP00000275730<br>Bt_ENSBTAP00000004493<br>Rn<br>Mm_ENSMUSP00000038106<br>Gg_ENSGALP00000012806<br>Xt_ENSXETP00000039825<br>Dr_ENSDARP00000079352<br>Ce_CE18193<br>Dm_FBpp0080630<br>Sc_YBR235W	NP_064631 Solute carrier family 12 member 9 (Potassium-chloride transporter 9)(Cation-chloride cotransporter-interacting protein 1)(CCC-interacting protein 1)(hCIP1)(Cation-chloride cotransporter 6)(hCCC6)(WO3.3)
387	1	CI	ENSP00000358400<br>ENSG00000112159	29<br>29<br>29<br>29<br>28<br>-<br>-<br>0<br>34<br>26	-----rselgRFLAKqvwtpqdrqc<br>-----rselgRFLVKqvwtpqdrqc<br>-----sgelnRFLAKsvwtpqdrqc<br>-----sgelnRFLAKsvwtpqdrqc<br>-----gaelsrflgkqtwtqqdrqc<br>.........................<br>.........................<br>-------------------------<br>ednvfsqslqkfikkqkyvpqdver<br>------safpsdaieapfhfsnkes	Hs_ENSP00000358400<br>Bt_ENSBTAP00000002157<br>Rn_ENSRNOP00000037995<br>Mm_ENSMUSP00000071569<br>Gg_ENSGALP00000025327<br>Xt<br>Dr<br>Ce_CE30377<br>Dm_FBpp0111985<br>Sc_YLR106C	NP_055426 Midasin (MIDAS-containing protein)
387	2	CI	ENSP00000358400<br>ENSG00000112159	1666<br>1667<br>1661<br>1661<br>1667<br>-<br>-<br>1270<br>1713<br>1671	tallarkeclKFLIKrlakivrlte<br>tallarreclKFLIKklskigrlte<br>tallareeclKFLIKklskvvrlte<br>tallareeclKFLIKklskvvrltq<br>tallarekcltflcekmsqfleltd<br>.........................<br>.........................<br>dpvevfddaltmlhselvktqllgq<br>vellk-----snivkqaikqaslil<br>nendlkslrteciiqllklcg----	Hs_ENSP00000358400<br>Bt_ENSBTAP00000002157<br>Rn_ENSRNOP00000037995<br>Mm_ENSMUSP00000071569<br>Gg_ENSGALP00000025327<br>Xt<br>Dr<br>Ce_CE30377<br>Dm_FBpp0111985<br>Sc_YLR106C	NP_055426 Midasin (MIDAS-containing protein)
388	1	CI	ENSP00000254657<br>ENSG00000132326	139<br>146<br>137<br>137<br>189<br>61<br>205<br>42<br>-<br>-	g-kastlatlKYALRsvkqvkanee<br>g-kastlatlKYALRsvkqvkanee<br>g-kastlatlKYALRsvkqvkanee<br>g-kastlatlKYALRsvkqvkanee<br>g-kssvlttlKYALKsikqvkanee<br>s-ksgtaaalkyalhciqqvqanee<br>gsksttlntlKYALRcvrqveanee<br>eegatlsppntwsssvefldddadd<br>.........................<br>.........................	Hs_ENSP00000254657<br>Bt_ENSBTAP00000002448<br>Rn_ENSRNOP00000027507<br>Mm_ENSMUSP00000066620<br>Gg_ENSGALP00000008844<br>Xt_ENSXETP00000018104<br>Dr_ENSDARP00000088777<br>Ce_CE37237<br>Dm<br>Sc	NP_073728 Period circadian protein homolog 2 (Circadian clock protein PERIOD 2)(hPER2)
389	1	CI	ENSP00000358854	304	netmldfrtsKFVLRisrdsyqllk	Hs_ENSP00000358854	NP_008882 Transcription

			ENSG00000148835	286	netmldfrtsKFVLRisrdsyqllk	Bt_ENSBTAP00000012808	initiation factor TFIID subunit 5 (Transcription initiation factor TFIID 100 kDa subunit)(TAF(II)100)(TAFII-100)(TAFII100)
				302	netmldfrtsKFVLRisrdsyqllk	Rn_ENSRNOP00000027482	
				305	netmldfrtsKFVLRisrdsyqllk	Mm_ENSMUSP00000026027	
				284	netmldfrtsKFVLRisrdsyqllk	Gg_ENSGALP00000013398	
				281	netildfrtsKFVLRisrdsyqllk	Xt_ENSXETP00000020911	
				260	nealldfrtsRFVLRisrdsyqllk	Dr_ENSDARP00000015825	
				170	nehvhilrenKFLVRlsrptlkhle	Ce_CE39361	
				206	ndlvvameqdKFVIRmsrdshslfk	Dm_FBpp0087335	
				241	nevasafqshkyritmskttlnlll	Sc_YBR198C	
390	1	C	ENSP00000358039 ENSG00000107669	99	shkkvlkkmlKFLAKgevpkgsce-	Hs_ENSP00000358039	NP_008972 Arginyl-tRNA--protein transferase 1 (Arginyltransferase 1)(R-transferase 1)(EC 2.3.2.8)(Arginine-tRNA--protein transferase 1)
				92	shkkvlkkmlKFLAKgeiskgnce-	Bt_ENSBTAP00000004132	
				99	shkkvlkkmlKFLAKgeiskgnce-	Rn_ENSRNOP00000031141	
				99	shkkvlkkmlKFLAKgeiskgnce-	Mm_ENSMUSP00000033139	
				98	shkkvlkkmlkfiskgdiskavse-	Gg_ENSGALP00000015450	
				99	shkkvlkkvqkylyvgevshskneg	Xt_ENSXETP00000034011	
				-	....	Dr	
				127	sqkramrqmneflatg---------	Ce_CE32465	
				92	snkrilrrinrflrdg-----kre-	Dm_FBpp0085627	
				122	elkkcisrfatritsedycpaavas	Sc_YGL017W	
391	1	CI	ENSP00000269844 ENSG00000141956	1028	kgygcsicnrRFALKatyhahmvih	Hs_ENSP00000269844	NP_071398 PR domain zinc finger protein 15 (PR domain-containing protein 15)(Zinc finger protein 298)
				658	kgygcsichrRFALKatyhahmvih	Bt_ENSBTAP00000028324	
				671	kghgcsichrRFALKatyhahmvih	Rn_ENSRNOP00000041592	
				671	kghgcsichrRFALKatyhahmvih	Mm_ENSMUSP00000113791	
				663	kgygcsicnrRFALKatyhahmvih	Gg_ENSGALP00000025970	
				661	ngydcsichrRFALKatyhahmvih	Xt_ENSXETP00000000120	
				-	....	Dr	
				-	....	Ce	
				-	....	Dm	
				-	....	Sc	
392	1	CI	ENSP00000255380 ENSG00000133019	466	keat---lakRFALKtrsqitkrkr	Hs_ENSP00000255380	NP_000731 Muscarinic acetylcholine receptor M3
				466	keat---lakRFALKtrsqitkrkr	Bt_ENSBTAP00000010601	
				465	keat---lakRFALKtrsqitkrkr	Rn_ENSRNOP00000019662	
				465	keat---lakRFALKtrsqitkrkr	Mm_ENSMUSP00000055579	
				516	keat---lakKFALKtrsqitkrkr	Gg_ENSGALP00000017509	
				413	keia---lakrfaaktrnqitkrkr	Xt_ENSXETP00000035440	
				430	patt---qasalllnagqrsrkart	Dr_ENSDARP00000096858	
				497	kqspfkngrilknfssqerksekeq	Ce_CE35800	
				690	ggaagggvggahalmnarnaakkkk	Dm_FBpp0072275	
				-	....	Sc	
393	1	C	ENSP00000338461 ENSG00000100243	156	sgllvyqgkgKFAIRpdkksnpiir	Hs_ENSP00000338461	NP_001123291 NADH-cytochrome b5 reductase 3 (Cytochrome b5 reductase)(B5R)(EC 1.6.2.2)(Diaphorase-1)
				158	ngllvyqgkgKFAIRpdkksdpvik	Bt_ENSBTAP00000021956	
				157	ngllvyqgkgKFAIRadkksnpvvr	Rn_ENSRNOP00000012878	
				133	ngllvyqgkgKFAIRadkksnpvvr	Mm_ENSMUSP00000055473	
				156	sgllvykgkgefairpekkadpvtk	Gg_ENSGALP00000022816	
				158	sglltysgrgtfqirpdkksppvtk	Xt_ENSXETP00000036013	
				153	sgllvykgkgsfairpdkksdavik	Dr_ENSDARP00000014732	
				164	qgnivykghglfsikadkkaepknr	Ce_CE13277	
				170	sgrlqylgngtfsikklrkdppkhv	Dm_FBpp0075810	
				-	....	Sc	
394	1	CI	ENSP00000348828 ENSG00000114790	654	s--------sRWLVKrgeltayved	Hs_ENSP00000348828	NP_056410 SH3 domain-containing guanine exchange factor
				653	s--------sRWLVKrgeltayved	Bt_ENSBTAP00000017878	
				652	s--------sRWLVKrgeltayved	Rn_ENSRNOP00000019554	
				652	s--------sRWLVKrgeltayved	Mm_ENSMUSP00000078281	
				426	s--------sRWLVKrgeltayved	Gg_ENSGALP00000016838	
				652	s--------sRWLLKrgeltayved	Xt_ENSXETP00000016947	
				-	....	Dr	
				882	n--------sRYLVKkgvltqlver	Ce_CE25936	
				827	vgvpragakpRFLVKrgelthllwr	Dm_FBpp0075031	
				-	....	Sc	
395	1	CI	ENSP00000355890 ENSG00000136628	56	viftdvnsilRYLARvattag-lyg	Hs_ENSP00000355890	NP_004437 Bifunctional aminoacyl-tRNA synthetase (Proliferation-inducing gene 32 protein)
				56	vvftdinsilRYLARiataag-lyg	Bt_ENSBTAP00000009064	
				56	vvftdtnsilRYLARvaatsg-lyg	Rn_ENSRNOP00000003252	
				56	vaftdvnsilRYLARiattsg-lyg	Mm_ENSMUSP00000045841	
				56	vsftnvnsivRYLARvaasag-lyg	Gg_ENSGALP00000015623	
				56	isftdvhsivRYLARlapdkg-lsg	Xt_ENSXETP00000005896	
				41	vqfsdvnsitRYLARvapalg-lyg	Dr_ENSDARP00000079714	
				60	ellsndveiariiaqstdaadsllg	Ce_CE06580	
				58	lvchsnndvlralaraapdyk-lyg	Dm_FBpp0083898	
				-	....	Sc	
396	1	CI	ENSP00000365437	168	eilmhcqttlKYAIKtghpryfnql	Hs_ENSP00000365437	NP_001127838 Glutamate

			ENSG00000136750	168<br>168<br>168<br>76<br>-<br>166<br>-<br>-<br>-	eilmhcqttlKYAIKtghpryfnql<br>eilthcqttlKYAIKtghpryfnql<br>eilthcqttlKYAIKtghpryfnql<br>eillncrttlKYAIKtghpryfnql<br>.........................<br>diliscratlKYAIKtahpryfnql<br>.........................<br>.........................<br>.........................	Bt_ENSBTAP00000010598<br>Rn_ENSRNOP00000024901<br>Mm_ENSMUSP00000028123<br>Gg_ENSGALP00000012254<br>Xt<br>Dr_ENSDARP00000034747<br>Ce<br>Dm<br>Sc	decarboxylase 2 (EC 4.1.1.15)(Glutamate decarboxylase 65 kDa isoform)(65 kDa glutamic acid decarboxylase)(GAD-65)
397	1	CI	ENSP00000259037<br>ENSG00000136521	119<br>119<br>119<br>119<br>116<br>116<br>116<br>98<br>110<br>-	hweyykhpisRWIARnfydspekiy<br>hweyfkhpisRWIARtffdgpekny<br>hweyykhpisRWIARtfydgpekny<br>hweyykhpisRWIARnfydgpekny<br>hweyykhpisRWIARylldppekny<br>hweyykhpitrwlcrnlfdspekey<br>hweyykhpitRWIARyvydspvkdy<br>hwqfertpirqwwakwfgvsdvehh<br>hweyekhpisrfisryilnsdqqny<br>.........................	Hs_ENSP00000259037<br>Bt_ENSBTAP00000048795<br>Rn_ENSRNOP00000016051<br>Mm_ENSMUSP00000029217<br>Gg_ENSGALP00000014752<br>Xt_ENSXETP00000015780<br>Dr_ENSDARP00000095073<br>Ce_CE29186<br>Dm_FBpp0075755<br>Sc	NP_002483 NADH dehydrogenase
398	1	C	ENSP00000328062<br>ENSG00000136051	246<br>259<br>246<br>246<br>252<br>243<br>246<br>217<br>185<br>-	iqeeklkpfeKFLLKlegqlldgmi<br>iqeeklkpfeKFLLKlegqlldgmi<br>iqeerlkpfeKFLLKlegqlldgmi<br>iqeeklkpfeKFLLKlegqlldgmi<br>iqedklkpfeklllklecqlldgmi<br>iqedklkpfeklllklesqlldgml<br>ipeeklkpfeklifklegqlldsmi<br>aqrkaydegaraqedaqsmilkgnc<br>-anlelnglstslmdien-litkdf<br>.........................	Hs_ENSP00000328062<br>Bt_ENSBTAP00000041052<br>Rn_ENSRNOP00000011326<br>Mm_ENSMUSP00000039322<br>Gg_ENSGALP00000020654<br>Xt_ENSXETP00000003997<br>Dr_ENSDARP00000082748<br>Ce_CE42423<br>Dm_FBpp0074003<br>Sc	NP_056090 Uncharacterized protein KIAA1033
399	1	CI	ENSP00000220509<br>ENSG00000104142	698<br>698<br>698<br>698<br>698<br>663<br>698<br>662<br>731<br>664	asphrvhydlKYALRlcaehghhra<br>asphrvhydlKYALRlcaehghhra<br>asphrvhydlKYALRlcaehghhra<br>asphrvhydlKYALRlcaehghhra<br>tnpnrihydlKYALRlcaehghhha<br>tnanrihydlKYALRlcaehghnra<br>thvsdihydlKYALRlcsehgylqa<br>tirsdlpyeldfamrtceqfkiepc<br>rdeslvhydiyyahkvctdldvkea<br>mksnenkydlnfqlrlslkfkktkt	Hs_ENSP00000220509<br>Bt_ENSBTAP00000015368<br>Rn_ENSRNOP00000018717<br>Mm_ENSMUSP00000036915<br>Gg_ENSGALP00000013826<br>Xt_ENSXETP00000046616<br>Dr_ENSDARP00000094129<br>Ce_CE32071<br>Dm_FBpp0070259<br>Sc_YLR148W	NP_065908 Vacuolar protein sorting-associated protein 18 homolog (hVPS18)
400	1	CI	ENSP00000266066<br>ENSG00000120057	292<br>296<br>289<br>289<br>269<br>288<br>291<br>272<br>-<br>-	yrwdkknkemKFAVKfmfsypcsly<br>yrwdkknkemKFAVKfmfsypcsly<br>yrwdkknkemKFAVKfmfsypcsly<br>yrwdkknkemKFAVKfmfsypcsly<br>ypwqkhnkemKFAVKfmfsypcply<br>ykwdkkskdmkyavnfmysypcsdt<br>hkwdkkskelKFAIKyiksqqcp--<br>lpwqke-knfkraihqfqrlncqsl<br>.........................<br>.........................	Hs_ENSP00000266066<br>Bt_ENSBTAP00000024709<br>Rn_ENSRNOP00000020182<br>Mm_ENSMUSP00000018966<br>Gg_ENSGALP00000012135<br>Xt_ENSXETP00000053187<br>Dr_ENSDARP00000056995<br>Ce_CE39983<br>Dm<br>Sc	NP_003006 Secreted frizzled-related protein 5 Precursor (sFRP-5)(Secreted apoptosis-related protein 3)(SARP-3)(Frizzled-related protein 1b)(FRP-1b)
401	1	CI	ENSP00000383960<br>ENSG00000076685	224<br>224<br>224<br>224<br>224<br>232<br>226<br>217<br>351<br>-	lkektvenleKYVVKdgklplllsr<br>lkektvenleKYVVKdgklplllsr<br>lkektvenleKYVVKdgklplllsr<br>lkektvenleKYVVKdgklplllsr<br>lkektlenleKYVVKdgklplllsr<br>lkektlkdleKYVIKdprtpvlltr<br>lkektvenleKYVVKdaklplllsr<br>mkkqvmenpeKYVIRderaprflsq<br>lkrrttqnmahyvkkderlptvlsr<br>.........................	Hs_ENSP00000383960<br>Bt_ENSBTAP00000017090<br>Rn_ENSRNOP00000027351<br>Mm_ENSMUSP00000084180<br>Gg_ENSGALP00000013294<br>Xt_ENSXETP00000041119<br>Dr_ENSDARP00000057573<br>Ce_CE33740<br>Dm_FBpp0074345<br>Sc	NP_036361 Cytosolic purine 5'-nucleotidase (EC 3.1.3.5)(5'-nucleotidase cytosolic II)
402	1	CI	ENSP00000383592<br>ENSG00000215711	276<br>189<br>289<br>287<br>-<br>217<br>287<br>278<br>396<br>-	nfprsprirkRFLLRrilemlfftq<br>nfprsprirkRFLLRrllemlfltq<br>nfprsprirkRFLLRrvlemlfftq<br>nfprsprirkRFLLRrvlemlfftq<br>.........................<br>nfprsprirkRFLLRrlvemltqtq<br>nfprsprirkRFLLRrllemlflmq<br>kfprllrirkhflikrtveliflsf<br>nfprtsrvrkRFLLKrllevvigvn<br>.........................	Hs_ENSP00000383592<br>Bt_ENSBTAP00000037256<br>Rn_ENSRNOP00000035110<br>Mm_ENSMUSP00000023214<br>Gg<br>Xt_ENSXETP00000003061<br>Dr_ENSDARP00000042516<br>Ce_CE16185<br>Dm_FBpp0089387<br>Sc	NP_036211 Diacylglycerol O-acyltransferase 1 (EC 2.3.1.20)(Diglyceride acyltransferase)(ACAT-related gene product 1)
403	1	CI	ENSP00000356581	968	lvkvfntyirRYILKfciigwglpa	Hs_ENSP00000356581	NP_940971 Probable G-protein

			ENSG00000112414	938<br>969<br>939<br>935<br>-<br>945<br>-<br>-<br>-	lvkvfntyirRYILKfcivgwgvpi<br>lvkvfntyihRYILKfciigwglpa<br>lvkvfntyihRYILKfciigwglpa<br>lvkvfntyirRYILKfciigwglpa<br>.........................<br>lvkvfntyirRYILKfcivgwgvpa<br>.........................<br>.........................<br>.........................	Bt_ENSBTAP00000037559<br>Rn_ENSRNOP00000029263<br>Mm_ENSMUSP00000043055<br>Gg_ENSGALP00000036714<br>Xt<br>Dr_ENSDARP00000093506<br>Ce<br>Dm<br>Sc	coupled receptor 126 Precursor
404	1	CI	ENSP00000366988<br>ENSG00000089006	264<br>264<br>264<br>264<br>258<br>245<br>259<br>-<br>-<br>-	laleeptvikKYLLKvaelfeklrk<br>laleeptvikKYLLKvaelfeklrk<br>laleeptvikKYLLKvaelfeklrk<br>laleeptvikKYLLKvaelfeklrk<br>laseeptvikKYLLKvaelfeklrk<br>vaeeentqvrKYLLKlaelfeklrk<br>vaadnnsafkkhlekcvevfeklrk<br>.........................<br>.........................<br>.........................	Hs_ENSP00000366988<br>Bt_ENSBTAP00000039205<br>Rn_ENSRNOP00000008934<br>Mm_ENSMUSP00000028909<br>Gg_ENSGALP00000032624<br>Xt_ENSXETP00000001722<br>Dr_ENSDARP00000018231<br>Ce<br>Dm<br>Sc	NP_055241 Sorting nexin-5
405	1	CI	ENSP00000367220<br>ENSG00000138071	196<br>185<br>191<br>191<br>190<br>191<br>197<br>191<br>191<br>190	rldiagrditRYLIKllllrgyafn<br>rldiagrditRYLIKllllrgyafn<br>rldiagrditRYLIKllllrgyafn<br>rldiagrditRYLIKllllrgyafn<br>rldiagrditRYLIKllllrgyafn<br>rldiagrditRYLIKllllrgyafn<br>rldiagrditRYLIKllllrgyafn<br>rldiagrditKYLIKlllqrgynfn<br>rldiagrditRYLIKllllrgyafn<br>rldvagrdvtrhlidllsrrgyafn	Hs_ENSP00000367220<br>Bt_ENSBTAP00000012872<br>Rn_ENSRNOP00000006607<br>Mm_ENSMUSP00000000137<br>Gg_ENSGALP00000038669<br>Xt_ENSXETP00000030571<br>Dr_ENSDARP00000075857<br>Ce_CE06111<br>Dm_FBpp0074070<br>Sc_YDL029W	Actin-related protein 2 (Actin-like protein 2)
406	1	CI	ENSP00000384244<br>ENSG00000204652	38<br>-<br>-<br>-<br>-<br>-<br>-<br>38<br>-<br>-	rcvpkdkaikKFVIRniveaaavrd<br>.........................<br>.........................<br>.........................<br>.........................<br>.........................<br>.........................<br>rccpkdkaikKFVVRniveaaavrd<br>.........................<br>.........................	Hs_ENSP00000384244<br>Bt<br>Rn<br>Mm<br>Gg<br>Xt<br>Dr<br>Ce_CE16012<br>Dm<br>Sc	Ribosomal protein S26 Fragment
407	1	CI	ENSP00000353910<br>ENSG00000033170	333<br>333<br>333<br>333<br>334<br>336<br>334<br>318<br>379<br>-	pavwwvsqfvKYLIRpqpwlekeie<br>pavwwvsqfvKYLIRpqpwlekeie<br>pavwwvsqfvKYLIRpqpwlekeie<br>pavwwvsqfvKYLIRpqpwlekeie<br>pavwwvsqfvKYLIRpqpwlekeie<br>pavwwvsqfvKYLIRpqpwlekeie<br>pavwwvsqfvKYLIRpqawlmkemq<br>ppaffvgtfisylmrfnsatqekld<br>pivwwvgqflKYLLRpqpttrdflt<br>.........................	Hs_ENSP00000353910<br>Bt_ENSBTAP00000026406<br>Rn_ENSRNOP00000011220<br>Mm_ENSMUSP00000054530<br>Gg_ENSGALP00000037734<br>Xt_ENSXETP00000016641<br>Dr_ENSDARP00000017854<br>Ce_CE32580<br>Dm_FBpp0073457<br>Sc	NP_835367 Alpha-(1,6)-fucosyltransferase (alpha1-6FucT) (EC 2.4.1.68)(Glycoprotein 6-alpha-L-fucosyltransferase)(GDP-fucose--glycoprotein fucosyltransferase)(GDP-L-Fuc:N-acetyl-beta-D-glucosaminide alpha1,6-fucosyltransferase) (Fucosyltransferase 8)
408	1	C	ENSP00000257177<br>ENSG00000134744	405<br>342<br>-<br>425<br>353<br>157<br>-<br>-<br>-<br>-	rlygsslt--RFALKssdvnidikf<br>qiskihlmtfpfalysdpwniftlv<br>.........................<br>rlygsslt--KFALKssdvnidikf<br>rmygsclt--rfafktsdinidikf<br>rmygsslt--rfaftssdinidvkf<br>.........................<br>.........................<br>.........................<br>.........................	Hs_ENSP00000257177<br>Bt_ENSBTAP00000003525<br>Rn<br>Mm_ENSMUSP00000095538<br>Gg_ENSGALP00000017275<br>Xt_ENSXETP00000017666<br>Dr<br>Ce<br>Dm<br>Sc	NP_001009881 Zinc finger CCHC domain-containing protein 11
409	1	CI	ENSP00000384245<br>ENSG00000121988	1074<br>1001<br>896<br>1064<br>1081<br>-<br>-<br>-<br>-<br>-	laskhgsditRFLVKk---------<br>lasnhgsditRFLVKk---------<br>latkhgsditRFLVKk---------<br>latkhgsditRFLVKk---------<br>latkygcditkffvkk---------<br>.........................<br>.........................<br>.........................<br>.........................<br>.........................	Hs_ENSP00000384245<br>Bt_ENSBTAP00000038188<br>Rn_ENSRNOP00000005355<br>Mm_ENSMUSP00000083806<br>Gg_ENSGALP00000019932<br>Xt<br>Dr<br>Ce<br>Dm<br>Sc	NP_115519 Zinc finger Ran-binding domain-containing protein 3 (EC 3.6.1.-)

410	1	CI	ENSP00000378720<br>ENSG00000126777	521<br>521<br>523<br>524<br>519<br>-<br>567<br>-<br>-<br>-	aaqq--dlqsKFVAKe-------ne<br>aaqq--dlqsKFVAKe-------ne<br>aaqq--dlqsKFVAKe-------ne<br>aaqq--dlqsKFVAKe-------ne<br>aaqqginvqnklvakd-------ne<br>.........................<br>kiqs--elqnqimvaesemasknke<br>.........................<br>.........................<br>.........................	Hs_ENSP00000378720<br>Bt_ENSBTAP00000041243<br>Rn_ENSRNOP00000016449<br>Mm_ENSMUSP00000022391<br>Gg_ENSGALP00000019799<br>Xt<br>Dr_ENSDARP00000041446<br>Ce<br>Dm<br>Sc	NP_001072989 Kinectin (Kinesin receptor)(CG-1 antigen)
411	1	CI	ENSP00000384288<br>ENSG00000005810	1790<br>1790<br>1787<br>1787<br>1728<br>-<br>50<br>1599<br>2113<br>-	dkvvplkenvKYAVRlrnyg-srta<br>dkvvplkenvKYAVRlrnyg-srta<br>dkvvplkenvKYAVRlrnyg-srta<br>dkvvplkenvKYAVRlrnyg-srta<br>dkvvplkenvKYAVRlrnyg-srta<br>.........................<br>dkavplkenvKYAVRlrnyg-srta<br>peyvllspgvtyavkvnmmkntktf<br>dhpvhikenaRYALRlcsqg-artc<br>.........................	Hs_ENSP00000384288<br>Bt_ENSBTAP00000025100<br>Rn_ENSRNOP00000014583<br>Mm_ENSMUSP00000045930<br>Gg_ENSGALP00000027295<br>Xt<br>Dr_ENSDARP00000044834<br>Ce_CE06730<br>Dm_FBpp0073787<br>Sc	NP_055872 Probable E3 ubiquitin-protein ligase MYCBP2 (EC 6.3.2.-)(Myc-binding protein 2)(Protein associated with Myc)(Pam/highwire/rpm-1 protein)
411	2	CI	ENSP00000384288<br>ENSG00000005810	2432<br>2432<br>2430<br>2427<br>2372<br>-<br>688<br>2171<br>3025<br>-	sepq-pnkvrKFVAKds---aglri<br>tepq-pnkvrKFVAKds---aglri<br>adpq-pnkirKFVAKds---aglri<br>adpq-pnkirKFVAKds---aglri<br>aepq-pnkvrKFVAKds---aglri<br>.........................<br>aepq-pskvrKFVAKds---aglrv<br>rrgaqmtvartvsipfssdfsgirm<br>rrpqapsklrrfqarhs---sglri<br>.........................	Hs_ENSP00000384288<br>Bt_ENSBTAP00000025100<br>Rn_ENSRNOP00000014583<br>Mm_ENSMUSP00000045930<br>Gg_ENSGALP00000027295<br>Xt<br>Dr_ENSDARP00000044834<br>Ce_CE06730<br>Dm_FBpp0073787<br>Sc	NP_055872 Probable E3 ubiquitin-protein ligase MYCBP2 (EC 6.3.2.-)(Myc-binding protein 2)(Protein associated with Myc)(Pam/highwire/rpm-1 protein)
412	1	CI	ENSP00000305826<br>ENSG00000160087	266<br>250<br>250<br>262<br>250<br>250<br>266<br>203<br>274<br>246	vgfaafaytvKYVLRsiaqe-----<br>vgfaafaytvKYVLRsiaqe-----<br>vgfaafaytvKYVLRsiaqe-----<br>vgfaafaytvKYVLRsiaqe-----<br>vgfaafaytvKYVLRsiaqe-----<br>vgfaafaytvKYVLRsiaqe-----<br>vgfaafaytvKYVLRsiaqe-----<br>-----waf-----------------<br>icfaifalivnyviknlnqe-----<br>igiaiflflvglfmk----------	Hs_ENSP00000305826<br>Bt_ENSBTAP00000021109<br>Rn_ENSRNOP00000026752<br>Mm_ENSMUSP00000024056<br>Gg_ENSGALP00000002860<br>Xt_ENSXETP00000002268<br>Dr_ENSDARP00000081358<br>Ce_CE33205<br>Dm_FBpp0082929<br>Sc_YER100W	NP_919296 Ubiquitin-conjugating enzyme E2 J2 (EC 6.3.2.19)(Non-canonical ubiquitin-conjugating enzyme 2)(NCUBE2)
413	1	CI	ENSP00000216446<br>ENSG00000100558	268<br>268<br>268<br>268<br>270<br>-<br>272<br>-<br>-<br>-	ghkrknwkvrRFVLRkdpaflhyyd<br>ghkrknwkvrRFVLRkdpaflhyyd<br>ghkrknwkvrRFVLRkdpaflhyyd<br>ghkrknwkvrRFVLRkdpaflhyyd<br>ghkrknwkvrKFVLRadpaflhyyd<br>.........................<br>ghkvknwkvrlfvlraepgflhyyd<br>.........................<br>.........................<br>.........................	Hs_ENSP00000216446<br>Bt_ENSBTAP00000021623<br>Rn_ENSRNOP00000014125<br>Mm_ENSMUSP00000021544<br>Gg_ENSGALP00000015553<br>Xt<br>Dr_ENSDARP00000010322<br>Ce<br>Dm<br>Sc	NP_057529 Pleckstrin-2
414	1	CI	ENSP00000364220<br>ENSG00000158816	445<br>438<br>437<br>437<br>431<br>187<br>-<br>-<br>-<br>-	mggtnilsplKWVIRqpvhrghprl<br>mggtnilsplKWVIRqpvlrgcprl<br>mggtnifsplKWVLRqplrqghprl<br>mggtnmlsplKWVLRqplrrghprl<br>tgspnilsplKWIVRqpirrghprl<br>mggtnilsplnwifrqpvcrgyprl<br>.........................<br>.........................<br>.........................<br>.........................	Hs_ENSP00000364220<br>Bt_ENSBTAP00000017965<br>Rn_ENSRNOP00000022282<br>Mm_ENSMUSP00000030533<br>Gg_ENSGALP00000016627<br>Xt_ENSXETP00000038130<br>Dr<br>Ce<br>Dm<br>Sc	NP_001034589 Putative VWFA-repeat-containing protein FLJ32784 Precursor
415	1	CI	ENSP00000216407<br>ENSG00000100519	68<br>82<br>68<br>68<br>82<br>82<br>68<br>85<br>69<br>116	gevlkqlteeKFIVKatngpryvvg<br>gevlkqlteeKFIVKatngpryvvg<br>gevlkqlteeKFIVKatngpryvvg<br>gevlkqlteeKFIVKatngpryvvg<br>gevlkqlteeKFIVKatngpryvvg<br>gevlkqlteeKFIVKatngpryvvg<br>gevlkqlteeKFIVKatngpryvvg<br>gevlkqlseeKFIVKatngpryvvg<br>gevlkqltedKFIVKatngpryvvg<br>gevmkelseeKYIVKassgpryivg	Hs_ENSP00000216407<br>Bt_ENSBTAP00000020886<br>Rn_ENSRNOP00000009649<br>Mm_ENSMUSP00000022380<br>Gg_ENSGALP00000020250<br>Xt_ENSXETP00000018862<br>Dr_ENSDARP00000053833<br>Ce_CE29280<br>Dm_FBpp0070890<br>Sc_YOR259C	NP_002797 26S protease regulatory subunit S10B (Proteasome 26S subunit ATPase 6)(Proteasome subunit p42)

416	1	CI	ENSP00000233838<br>ENSG00000115486	683<br>683<br>683<br>683<br>-<br>675<br>-<br>-<br>649<br>-	rntpfherffRFLLRklyvfrrsfl<br>rnapfherlvRFLLRklfifrrsfl<br>rnsplherflRFVLRklyvfrrsfl<br>rnspfherflRFVLRklyvfrrsfl<br>.........................<br>rnatllqrferfcgkkyyllrrsfl<br>.........................<br>.........................<br>--vtnyqqflghign----------<br>.........................	Hs_ENSP00000233838<br>Bt_ENSBTAP00000027811<br>Rn_ENSRNOP00000017928<br>Mm_ENSMUSP00000070109<br>Gg<br>Xt_ENSXETP00000002717<br>Dr<br>Ce<br>Dm_FBpp0072701<br>Sc	NP_000812 Vitamin K-dependent gamma-carboxylase (EC 6.4.-.-) (Gamma-glutamyl carboxylase) (Vitamin K gamma glutamyl carboxylase)
417	1	CI	ENSP00000350541<br>ENSG00000172901	750<br>749<br>549<br>559<br>716<br>699<br>-<br>-<br>-<br>-	niydiysllkRYLLKrlnliwniys<br>nnhdiypllkKYLLKrlisiwntys<br>-------------------------<br>-------------------------<br>knyevypllkKYLLKrmlpiyryya<br>elwktp---kKYILKrispiyqrfa<br>.........................<br>.........................<br>.........................<br>.........................	Hs_ENSP00000350541<br>Bt_ENSBTAP00000022140<br>Rn_ENSRNOP00000005252<br>Mm_ENSMUSP00000025358<br>Gg_ENSGALP00000003642<br>Xt_ENSXETP00000040582<br>Dr<br>Ce<br>Dm<br>Sc	NP_776161 Laeverin (EC 3.4.-.-) (CHL2 antigen)
417	2	CI	ENSP00000350541<br>ENSG00000172901	902<br>901<br>549<br>559<br>871<br>817<br>-<br>-<br>-<br>-	ievvassevgRYVAKdflvnnwqav<br>ieavaasevgRYVAKdflinnwqav<br>-------------------------<br>-------------------------<br>ldyvlskdighriawefvtenwsfl<br>-------------------------<br>.........................<br>.........................<br>.........................<br>.........................	Hs_ENSP00000350541<br>Bt_ENSBTAP00000022140<br>Rn_ENSRNOP00000005252<br>Mm_ENSMUSP00000025358<br>Gg_ENSGALP00000003642<br>Xt_ENSXETP00000040582<br>Dr<br>Ce<br>Dm<br>Sc	NP_776161 Laeverin (EC 3.4.-.-) (CHL2 antigen)
418	1	CI	ENSP00000261942<br>ENSG00000113194	115<br>115<br>16<br>115<br>122<br>115<br>115<br>-<br>125<br>124	ftyytildifRFALRfirpdprsr-<br>ftyytildifRFALRfirpdprnr-<br>ftyytildifRFALRfirpdprsr-<br>ftyytildifRFALRfirpdprsr-<br>ftyytlldifRFALRfirpdprsr-<br>ityytlldifRFAVRfirpdprsr-<br>ftyytlldifRFALRfirpdprgr-<br>.........................<br>yfystltsivsafvnlgggnearl-<br>neaqavtcsegngnnddgsntdsts	Hs_ENSP00000261942<br>Bt_ENSBTAP00000023592<br>Rn_ENSRNOP00000023701<br>Mm_ENSMUSP00000026991<br>Gg_ENSGALP00000005262<br>Xt_ENSXETP00000023916<br>Dr_ENSDARP00000033367<br>Ce<br>Dm_FBpp0080670<br>Sc_YDL091C	NP_055428 FAS-associated factor 2 (UBX domain-containing protein 3B)(UBX domain-containing protein 8)(Protein ETEA)
419	1	CI	ENSP00000230053<br>ENSG00000112309	128<br>131<br>-<br>129<br>120<br>129<br>123<br>-<br>-<br>-	daaarselvsRFLARaglpsthlhv<br>daaarselvsRFLARaglpsthlhv<br>.........................<br>dratrselvssflaraglpnthlhv<br>daatrselvsrfvagaglpcthlhv<br>dsvhptelvsrflagagvtsthlyv<br>danshtelvsRFLARcgvrythlnv<br>.........................<br>.........................<br>.........................	Hs_ENSP00000230053<br>Bt_ENSBTAP00000027741<br>Rn<br>Mm_ENSMUSP00000066582<br>Gg_ENSGALP00000036271<br>Xt_ENSXETP00000038790<br>Dr_ENSDARP00000050302<br>Ce<br>Dm<br>Sc	NP_542780 Galactosylgalactosylxylosylprotein 3-beta-glucuronosyltransferase 2 (EC 2.4.1.135)(Beta-1,3-glucuronyltransferase 2)(UDP-glucuronosyltransferase-S) (Glucuronosyltransferase-S) (GlcAT-S)(GlcAT-D)
420	1	CI	ENSP00000365771<br>ENSG00000152767	114<br>57<br>57<br>114<br>61<br>-<br>-<br>100<br>-<br>-	irr-pkhvvvKFVVKffppdhtqlq<br>irr-pkhvvvKFVVKffppdhtqlq<br>irr-pkhvlvKFVVKffppdhtqlq<br>irr-pkhvvvKFVVKffppdhtqlq<br>irr-pkhvvvKFVVKffppdhaqlq<br>.........................<br>.........................<br>itngstdakfyfvvkfytpnpidle<br>.........................<br>.........................	Hs_ENSP00000365771<br>Bt_ENSBTAP00000000167<br>Rn_ENSRNOP00000015271<br>Mm_ENSMUSP00000026635<br>Gg_ENSGALP00000027234<br>Xt<br>Dr<br>Ce_CE43269<br>Dm<br>Sc	FERM, RhoGEF and pleckstrin domain-containing protein 1 (Chondrocyte-derived ezrin-like protein)
420	2	CI	ENSP00000365771<br>ENSG00000152767	275<br>218<br>218<br>275<br>222<br>-<br>-<br>264<br>-<br>-	kvrklsfkrkRFLIKlrpdansayq<br>kvrklsfkrkRFLIKlrpdvnssyq<br>kvrklsfkrkRFLIKlrpdvnssyq<br>kvrklsfkrkRFLIKlrpdvnssyq<br>kvrklsfkrkRFLIKlrpdvnssfq<br>.........................<br>.........................<br>rirklsfkrkkllvklhpdsyqylk<br>.........................<br>.........................	Hs_ENSP00000365771<br>Bt_ENSBTAP00000000167<br>Rn_ENSRNOP00000015271<br>Mm_ENSMUSP00000026635<br>Gg_ENSGALP00000027234<br>Xt<br>Dr<br>Ce_CE43269<br>Dm<br>Sc	FERM, RhoGEF and pleckstrin domain-containing protein 1 (Chondrocyte-derived ezrin-like protein)

421	1	CI	ENSP00000261169<br>ENSG00000048540	34<br>23<br>23<br>23<br>25<br>-<br>23<br>-<br>-<br>-	cagcnrkikdRYLLKaldkywhedc<br>cagcnrkikdRYLLKaldkywhedc<br>cagcnrkikdRYLLKaldkywhedc<br>cagcnrkikdRYLLKaldkywhedc<br>cagcnrkikdRYLLKaldkywhedc<br>.........................<br>cagcnrkikdRYLLKaldkywhedc<br>.........................<br>.........................<br>.........................	Hs_ENSP00000261169<br>Bt_ENSBTAP00000040639<br>Rn_ENSRNOP00000010623<br>Mm_ENSMUSP00000069425<br>Gg_ENSGALP00000009582<br>Xt<br>Dr_ENSDARP00000015268<br>Ce<br>Dm<br>Sc	NP_061110 LIM domain only protein 3 (Neuronal-specific transcription factor DAT1) (Rhombotin-3)
422	1	CI	ENSP00000369014<br>ENSG00000173376	416<br>271<br>415<br>416<br>403<br>418<br>359<br>377<br>421<br>-	fqlrgkpka-KYLVRlkgnkkgasm<br>fqlrgkpka-KYLIRlkgnkkgasm<br>fqlrgkpkg-KYLIRlkgnkkgasm<br>fqlrgkpkg-KYLIRlkgnrkgask<br>fqlrgkaka-KYLIRlkgnkkgasm<br>fqlrgkpka-KYLIRlkgskkgasm<br>fqlrgkpka-KYLIRlrgsrkgast<br>fvvtnirsg-hlrfqivnddetpkt<br>irqkgvlpgerylmrfepsnddeal<br>.........................	Hs_ENSP00000369014<br>Bt_ENSBTAP00000014058<br>Rn_ENSRNOP00000009094<br>Mm_ENSMUSP00000051297<br>Gg_ENSGALP00000019482<br>Xt_ENSXETP00000004498<br>Dr_ENSDARP00000085965<br>Ce_CE34416<br>Dm_FBpp0072170<br>Sc	NP_078850 Fibronectin type-III domain-containing protein C4orf31 Precursor
423	1	CI	ENSP00000348349<br>ENSG00000066933	104<br>104<br>104<br>-<br>105<br>101<br>-<br>127<br>-<br>-	lenrlsgedyRFLLReknldgsihy<br>lenrlsgedyRFLLReknldgsihy<br>lenrlsgedyRFLLReknldgsihy<br>.........................<br>lenrtsgedyRFLLReknldgsihy<br>leargsgedyRFLLReknmdgsihy<br>.........................<br>vvvdsatpknrfvfrhrgyr--aat<br>.........................<br>.........................	Hs_ENSP00000348349<br>Bt_ENSBTAP00000009780<br>Rn_ENSRNOP00000015963<br>Mm<br>Gg_ENSGALP00000003229<br>Xt_ENSXETP00000011382<br>Dr<br>Ce_CE23779<br>Dm<br>Sc	NP_008832 Myosin-IXa (Unconventional myosin-9a)
424	1	CI	ENSP00000259335<br>ENSG00000136813	934<br>757<br>749<br>757<br>751<br>-<br>753<br>718<br>758<br>781	sllalgftvgRYLAKkk--------<br>sllalgftvgRYLAKkk--------<br>sllalgftvgRYLAKkr--------<br>sllalgftvgRYLAKkr--------<br>sllalgftvgRYLAKgk--------<br>.........................<br>ailalgymvgrymnkkr--------<br>eipglaftcasmlvpvlgg------<br>wllvvghtfnrkiamlkqenktkdf<br>tkiaqsqtmaetyvasy--------	Hs_ENSP00000259335<br>Bt_ENSBTAP00000041797<br>Rn_ENSRNOP00000019802<br>Mm_ENSMUSP00000103185<br>Gg_ENSGALP00000025258<br>Xt<br>Dr_ENSDARP00000083382<br>Ce_CE00639<br>Dm_FBpp0087116<br>Sc_YHL030W	NP_001073867 Proteasome-associated protein ECM29 homolog (Ecm29)
425	1	C	ENSP00000286719<br>ENSG00000156194	32<br>32<br>32<br>32<br>34<br>37<br>30<br>95<br>18<br>-	aaaliqrwyrRYVARlemrrrctws<br>aaaliqrwyrRYVARlemrrrctws<br>aaaliqrwyrrymarlemrrrctwn<br>aaaliqrwyrrymarlemrrrctwn<br>aavliqrwyrRYVARlemrrrctwr<br>aailiqrwyrfytarlemrrrytln<br>aailiqrwyrryvahlemrrrytwn<br>sailiqkwyrrcearlearrratwq<br>aaifiqkwyrrhqarremqrrcnwq<br>.........................	Hs_ENSP00000286719<br>Bt_ENSBTAP00000020539<br>Rn_ENSRNOP00000003122<br>Mm_ENSMUSP00000031359<br>Gg_ENSGALP00000018767<br>Xt_ENSXETP00000014755<br>Dr_ENSDARP00000009463<br>Ce_CE27999<br>Dm_FBpp0074602<br>Sc	NP_006230 Serine/threonine-protein phosphatase with EF-hands 2 (PPEF-2)(EC 3.1.3.16)
426	1	C	ENSP00000386405<br>ENSG00000170379	291<br>292<br>292<br>292<br>-<br>212<br>289<br>-<br>-<br>-	kmgpfllnavRWLARgqtgkvgvnt<br>slgpfllnavRWLAKgqtgkvgvnt<br>kmepfllnairwlsrdqgktigvnk<br>kmepfllnairwlsrgqednigvnt<br>.........................<br>elkqlilntiswldighqrriavhe<br>tmstfminavkwldegrqgvvgilp<br>.........................<br>.........................<br>.........................	Hs_ENSP00000386405<br>Bt_ENSBTAP00000007200<br>Rn_ENSRNOP00000050765<br>Mm_ENSMUSP00000031879<br>Gg<br>Xt_ENSXETP00000048647<br>Dr_ENSDARP00000029748<br>Ce<br>Dm<br>Sc	Protein FAM115C (Protein FAM139A)
426	2	C	ENSP00000386405<br>ENSG00000170379	435<br>436<br>436<br>436<br>-<br>360<br>437<br>-<br>-<br>-	hengn--leksclaklrvdgaaflq<br>leggn--leknwlaklrvdgaaflq<br>hkggn--lekkyfgklgvdgagflq<br>hkegn--lekrysgklgvdgagflq<br>.........................<br>kdtelpppfctwvtkltrdvtaflr<br>lgqkltdqeqdylkqlgndcasylr<br>.........................<br>.........................<br>.........................	Hs_ENSP00000386405<br>Bt_ENSBTAP00000007200<br>Rn_ENSRNOP00000050765<br>Mm_ENSMUSP00000031879<br>Gg<br>Xt_ENSXETP00000048647<br>Dr_ENSDARP00000029748<br>Ce<br>Dm<br>Sc	Protein FAM115C (Protein FAM139A)

427	1	CI	ENSP00000307697 ENSG00000168306	449	entvlylqvaRFLVKsylqtqmspg	Hs_ENSP00000307697	NP_003491 Peroxisomal acyl-coenzyme A oxidase 2 (EC 1.17.99.3)(3-alpha,7-alpha,12-alpha-trihydroxy-5-beta-cholestanoyl-CoA 24-hydroxylase)(3-alpha,7-alpha,12-alpha-trihydroxy-5-beta-cholestanoyl-CoA oxidase)(Trihydroxycoprostanoyl-CoA oxidase)(THCA-CoA oxidase)(THCCox)
				450	entvlylqtaRFLIKsylntqkspg	Bt_ENSBTAP00000005472	
				449	entvlylqvarflmksylqaqaspg	Rn_ENSRNOP00000010260	
				449	entvlylqvarflmksylqaqvspg	Mm_ENSMUSP00000022271	
				449	entilllqtaRFLIKcfsaasag--	Gg_ENSGALP00000011541	
				444	entvlhlqaaRFLIKcyaaarsg--	Xt_ENSXETP00000051094	
				-	....	Dr	
				-	....	Ce	
				-	....	Dm	
				-	....	Sc	
428	1	CI	ENSP00000303147 ENSG00000168906	299	kvdrsaayaaRWVAKslvkgglcrr	Hs_ENSP00000303147	NP_005902 S-adenosylmethionine synthetase isoform type-2 (AdoMet synthetase 2)(EC 2.5.1.6)(Methionine adenosyltransferase 2)(Methionine adenosyltransferase II)(MAT-II)
				299	kvdrsaayaaRWVAKslvkgglcrr	Bt_ENSBTAP00000027808	
				299	kvdrsaayaaRWVAKslvkgglcrr	Rn_ENSRNOP00000018170	
				299	kvdrsaayaaRWVAKslvkgglcrr	Mm_ENSMUSP00000087118	
				-	....	Gg	
				300	kvdrsaayaaRWVAKslvksglcrr	Xt_ENSXETP00000002716	
				300	kvdrsaayaaRWVAKslvkaklcrr	Dr_ENSDARP00000053962	
				287	kvdrsaayaaRWVAKslvksglcrr	Ce_CE03957	
				310	kvdrsaayaaRWVAKslvkaglckr	Dm_FBpp0088446	
				-	....	Sc	
429	1	CI	ENSP00000376453 ENSG00000172292	57	cifmvrlifeRFVAKpcaialniqa	Hs_ENSP00000376453	LAG1 longevity assurance homolog 6
				-	....	Bt	
				282	cifmvrlifeRFIAKpcavalniqa	Rn_ENSRNOP00000035208	
				57	cifmvrlifeRFIAKpcaialniqa	Mm_ENSMUSP00000028426	
				57	cifmirllfeRFIAKpcalglkvqa	Gg_ENSGALP00000017712	
				57	cifcirlffeRFIAKpcalalkiqa	Xt_ENSXETP00000035379	
				57	cmfmirlvfeRFIARpcalglkiqa	Dr_ENSDARP00000070109	
				-	....	Ce	
				-	....	Dm	
				-	....	Sc	
430	1	CI	ENSP00000339450 ENSG00000096063	344	vfevlghhllKWIIKsnyqglplpc	Hs_ENSP00000339450	Serine/threonine-protein kinase SRPK1 (EC 2.7.11.1)(Serine/arginine-rich protein-specific kinase 1)(SR-protein-specific kinase 1)(SFRS protein kinase 1)
				174	vfevlghhllKWIIKsnyqglplpc	Bt_ENSBTAP00000022396	
				174	vfevlghhllKWIIKsnyqglplpc	Rn_ENSRNOP00000000612	
				174	vfevlghhllKWIIKsnyqglplpc	Mm_ENSMUSP00000004987	
				174	vfevlghhllKWIIKsnyqglplpc	Gg_ENSGALP00000001306	
				174	vfevlghhllKWIIKsnyqgvplpc	Xt_ENSXETP00000000907	
				176	vfevlghhllKWIIKsnyqglplpc	Dr_ENSDARP00000063161	
				516	vfevlgcnllkliirsnyrglhleq	Ce_CE36580	
				264	vfevlgdnllklirksnyrgiplan	Dm_FBpp0086515	
				-	....	Sc	
431	1	CI	ENSP00000221980 ENSG00000105376	81	lrrngtqrglRWLARqlvdirepet	Hs_ENSP00000221980	NP_003250 Intercellular adhesion molecule 5 Precursor (ICAM-5)(Telencephalin)
				82	lrrngtqkglRWLARqlvdirepet	Bt_ENSBTAP00000036018	
				81	lrrngtqrglRWLARqlvdirepet	Rn_ENSRNOP00000028096	
				81	lrrngtqrglRWLARqlvdirepet	Mm_ENSMUSP00000019616	
				-	....	Gg	
				-	....	Xt	
				-	....	Dr	
				-	....	Ce	
				-	....	Dm	
				-	....	Sc	
432	1	CI	ENSP00000356473 ENSG00000146414	1635	igqtkptivhRFLIKatieermqam	Hs_ENSP00000356473	NP_001036148 E3 ubiquitin-protein ligase SHPRH (EC 6.3.2.-)(EC 3.6.1.-)(SNF2, histone-linker, PHD and RING finger domain-containing helicase)
				1636	igqtkptivhRFLIKatieermqam	Bt_ENSBTAP00000010673	
				1629	igqtkptivhRFLIKatieermqam	Rn_ENSRNOP00000019893	
				1622	igqtkptivhRFLIKatieermqam	Mm_ENSMUSP00000039422	
				1630	igqtkstivhRFLIKatieermqtm	Gg_ENSGALP00000020055	
				-	....	Xt	
				-	....	Dr	
				1546	fgqkrdmnvyhllvdgsldielret	Ce_CE37666	
				1223	fgqkrptkvhrfivnetieenilsl	Dm_FBpp0076706	
				-	....	Sc	
433	1	CI	ENSP00000278590 ENSG00000149289	337	qgrrvvcyddRFIVKlafesdgiiv	Hs_ENSP00000278590	NP_203748 Zinc finger CCCH domain-containing protein 12C (MCP-induced protein 3)
				81	qgrrvvcyddRFIVKlafesdgiiv	Bt_ENSBTAP00000011504	
				67	qgrrvvcyddRFIVKlafesdgiiv	Rn_ENSRNOP00000055841	
				81	qgrrvvcyddRFIVKlafesdgiiv	Mm_ENSMUSP00000047887	
				214	qgrrvvcyddRFIVKlafesdgiiv	Gg_ENSGALP00000027652	
				-	....	Xt	
				-	....	Dr	

				315<br>222<br>-	ngrrvvchddRYILRtaelkdaviv<br>dgkrvscyddRFILKlavetdgivv<br>.........................	Ce_CE40689<br>Dm_FBpp0083248<br>Sc	
434	1	CI	ENSP00000278550<br>ENSG00000149256	2571<br>2407<br>2634<br>2640<br>2297<br>2631<br>2627<br>-<br>-<br>-	ssgsvfgkgvKFALKdgrvttdiis<br>ssgsvfgkgvKFALKdgrvatdiis<br>ssgsifgkgvKFALKdgrvttdiis<br>ssgsifgkgvKFALKdgrvttdiis<br>-------------------------<br>tlgsifgkgvkfamkdgrvttdiis<br>tgtslfgkgvkvairegrveadiis<br>.........................<br>.........................<br>.........................	Hs_ENSP00000278550<br>Bt_ENSBTAP00000045891<br>Rn_ENSRNOP00000052240<br>Mm_ENSMUSP00000102787<br>Gg_ENSGALP00000027844<br>Xt_ENSXETP00000033455<br>Dr_ENSDARP00000016395<br>Ce<br>Dm<br>Sc	NP_001092286 Teneurin-4 (Ten-4)(Tenascin-M4)(Ten-m4)(Protein Odd Oz/ten-m homolog 4)
435	1	C	ENSP00000332313<br>ENSG00000184886	399<br>398<br>-<br>398<br>400<br>400<br>-<br>398<br>408<br>390	llgdiilsfaKFLIKgalvpcswkl<br>llgdiilsfaKFVIKeaavpcswkl<br>.........................<br>llsgiilsfaqflikgslvpcswkl<br>vvadlmlvftkllvkgsavpscwra<br>fvcdlimvlakylvpgsripgtwvi<br>.........................<br>mtfatgvclsiqmfntvawcssfpq<br>fgaidfainsvmpwdpnpwedhekg<br>hpysvsrrfanlpytlwvitynllf	Hs_ENSP00000332313<br>Bt_ENSBTAP00000045720<br>Rn<br>Mm_ENSMUSP00000103715<br>Gg_ENSGALP00000038373<br>Xt_ENSXETP00000004357<br>Dr<br>Ce_CE33941<br>Dm_FBpp0072678<br>Sc_YJL091C	NP_848612 Phosphatidylinositol-glycan biosynthesis class W protein (PIG-W)(EC 2.3.-.-)
436	1	CI	ENSP00000309031<br>ENSG00000175073	408<br>409<br>407<br>407<br>402<br>-<br>371<br>-<br>-<br>-	viggdrslqdKYLLRlvaameevfm<br>viggdrslqdKYLLRlvaameevfm<br>viggdrslqdKYLLRlvaameevfm<br>viggdrslqdKYLLRlvaameevfm<br>viggdrslqdkylmrlvaameevfm<br>.........................<br>viggdrslqdKYLLRlvsamedvfm<br>.........................<br>.........................<br>.........................	Hs_ENSP00000309031<br>Bt_ENSBTAP00000008802<br>Rn_ENSRNOP00000009136<br>Mm_ENSMUSP00000051248<br>Gg_ENSGALP00000024983<br>Xt<br>Dr_ENSDARP00000078952<br>Ce<br>Dm<br>Sc	NP_079330 Deubiquitinating protein VCIP135 (EC 3.4.22.-)(Valosin-containing protein p97/p47 complex-interacting protein p135)(Valosin-containing protein p97/p47 complex-interacting protein 1)
437	1	CI	ENSP00000301284<br>ENSG00000167674	483<br>482<br>-<br>479<br>-<br>476<br>490<br>581<br>-<br>-	eklqklhseiKFALKvdspdvkrcl<br>eklqklhseiKFALKvdnpdvkrcl<br>.........................<br>erlqklhseiKFALKvdnpdvrkcl<br>.........................<br>eklqklhseiKFALKvdnpdiqkcl<br>eklqklhtdiKFALKvdnpdiekcl<br>eeddgypslkrkvgrpskndikirl<br>.........................<br>.........................	Hs_ENSP00000301284<br>Bt_ENSBTAP00000017626<br>Rn<br>Mm_ENSMUSP00000002911<br>Gg<br>Xt_ENSXETP00000052142<br>Dr_ENSDARP00000062364<br>Ce_CE37700<br>Dm<br>Sc	NP_001001520 Hepatoma-derived growth factor-related protein 2 (HRP-2)(Hepatoma-derived growth factor 2)
438	1	CI	ENSP00000384136<br>ENSG00000137710	254<br>254<br>253<br>254<br>254<br>-<br>-<br>-<br>-<br>-	eirnisfndkKFVIKpidkkapdfv<br>eirnisfndkKFVIKpidkkapdfv<br>rlcavts-dfyfllklrvvkdtdfv<br>eirnisfndkKFVIKpidkkapdfv<br>eirnisfndkKFVIKpidkkapdfv<br>.........................<br>.........................<br>.........................<br>.........................<br>.........................	Hs_ENSP00000384136<br>Bt_ENSBTAP00000033759<br>Rn_ENSRNOP00000035840<br>Mm_ENSMUSP00000000590<br>Gg_ENSGALP00000021028<br>Xt<br>Dr<br>Ce<br>Dm<br>Sc	NP_002897 Radixin
439	1	CI	ENSP00000264051<br>ENSG00000066248	488<br>364<br>394<br>488<br>384<br>441<br>-<br>-<br>-<br>-	iksvpiishsRWLLKqgelqqmsgp<br>iksvpiishsRWLLKqgelqqmsgp<br>iksvpiishsRWLLKqgelqqmsgp<br>iksvpiishsRWLLKqgelqqmsgp<br>iksvpiishsRWLLKqgelqqmngp<br>iksvpiishsrwlmkqgelqqmsgp<br>.........................<br>.........................<br>.........................<br>.........................	Hs_ENSP00000264051<br>Bt_ENSBTAP00000011773<br>Rn_ENSRNOP00000022485<br>Mm_ENSMUSP00000066894<br>Gg_ENSGALP00000002287<br>Xt_ENSXETP00000030380<br>Dr<br>Ce<br>Dm<br>Sc	NP_062824 Ephexin-1 (Eph-interacting exchange protein)(Neuronal guanine nucleotide exchange factor)
440	1	CI	ENSP00000216373<br>ENSG00000100485	912<br>869<br>912<br>913<br>912<br>915<br>915	avelsqdhfkKYLVKlksinppcvp<br>avelsqdhfkKYLVKlksinppcvp<br>avelsqdhfkKYLVKlksinppcvp<br>avelsqdhfkKYLVKlksinppcvp<br>avelsqdhfkKYLAKlksinppcvp<br>avelsqdhfkKYLAKlksinppcvp<br>avelsqdhfkKYLAKlksinppcvp	Hs_ENSP00000216373<br>Bt_ENSBTAP00000007299<br>Rn_ENSRNOP00000006425<br>Mm_ENSMUSP00000044866<br>Gg_ENSGALP00000020023<br>Xt_ENSXETP00000020048<br>Dr_ENSDARP00000079754	NP_008870 Son of sevenless homolog 2 (SOS-2)

				-<br>963<br>-	.........................<br>crelsddhlkkyqerlrsinppcvp<br>.........................	Ce<br>Dm_FBpp0080118<br>Sc	
441	1	CI	ENSP00000374923<br>ENSG00000211753	28<br>-<br>-<br>28<br>-<br>-<br>-<br>-<br>-<br>-	lvdvkvtqssRYLVKrtgekvflec<br>.........................<br>.........................<br>lvdmkvtqmpRYLIKrmgenvllec<br>.........................<br>.........................<br>.........................<br>.........................<br>.........................<br>.........................	Hs_ENSP00000374923<br>Bt<br>Rn<br>Mm_ENSMUSP00000100097<br>Gg<br>Xt<br>Dr<br>Ce<br>Dm<br>Sc	T-cell receptor beta V gene segment
442	1	CI	ENSP00000056217<br>ENSG00000050327	1389<br>1423<br>-<br>-<br>-<br>-<br>-<br>-<br>-<br>-	ckifplisqsRWLVKsgeltalefs<br>ckifplisqsRWLVKsgeltalels<br>.........................<br>.........................<br>.........................<br>.........................<br>.........................<br>.........................<br>.........................<br>.........................	Hs_ENSP00000056217<br>Bt_ENSBTAP00000002373<br>Rn<br>Mm<br>Gg<br>Xt<br>Dr<br>Ce<br>Dm<br>Sc	NP_005426 Rho guanine nucleotide exchange factor 5 (Guanine nucleotide regulatory protein TIM)(Oncogene TIM)(p60 TIM)(Transforming immortalized mammary oncogene)
443	1	CI	ENSP00000281038<br>ENSG00000137513	353<br>353<br>353<br>353<br>354<br>-<br>361<br>304<br>341<br>366	wgadlrteheKYLVKhcgnipvfvi<br>wgadlhteheKYLVKhcgdipvfvi<br>wgvdlqteheKYLVRhcgnipvfvi<br>wgvdlqteheKYLVRhcgnipvfvi<br>wgcdlqteheKYLVKhcgevpvfvi<br>.........................<br>wgcdlqteheKFLVKhcgntpvfvv<br>aksgfskkneldlvklhddhpifvt<br>lnegfskdqelflvahcg-apvfvv<br>wgqplqtehekflageyfkspvfvt	Hs_ENSP00000281038<br>Bt_ENSBTAP00000048918<br>Rn_ENSRNOP00000015784<br>Mm_ENSMUSP00000044937<br>Gg_ENSGALP00000027851<br>Xt<br>Dr_ENSDARP00000085172<br>Ce_CE28801<br>Dm_FBpp0086205<br>Sc_YCR024C	NP_078954 Probable asparaginyl-tRNA synthetase, mitochondrial Precursor (EC 6.1.1.22) (Asparagine--tRNA ligase) (AsnRS)
444	1	CI	ENSP00000364158<br>ENSG00000165138	95<br>48<br>86<br>86<br>91<br>0<br>-<br>-<br>-<br>-	aaaggheplvRFLLRrgasvnsrnh<br>aaaggheplvRFLLRrgasvnsrnh<br>aaaggheplvRFLLRrgasvnsrnh<br>aaaggheplvRFLLRrgasvnsrnh<br>aaaggheqlvRFLLRkgasvqstnh<br>-------------------------<br>.........................<br>.........................<br>.........................<br>.........................	Hs_ENSP00000364158<br>Bt_ENSBTAP00000006765<br>Rn_ENSRNOP00000056754<br>Mm_ENSMUSP00000095691<br>Gg_ENSGALP00000021524<br>Xt_ENSXETP00000013613<br>Dr<br>Ce<br>Dm<br>Sc	Ankyrin repeat and SAM domain-containing protein 6 (Sterile alpha motif domain-containing protein 6)(SamCystin)(Ankyrin repeat domain-containing protein 14)
445	1	CI	ENSP00000385276<br>ENSG00000218336	1794<br>1457<br>1809<br>1810<br>1662<br>-<br>1807<br>-<br>-<br>-	ktekiyddhrKFLLRiaydtsghpt<br>ktekiyddhrKFLLRiaydtsghpt<br>ktekiyddhrKFLLRiaydtsghpt<br>ktekiyddhrKFLLRiaydtsghpt<br>ktekiyddhrKFLLRiaydtsghpt<br>.........................<br>rtekiyddhrKFLLKivydasghpt<br>.........................<br>.........................<br>.........................	Hs_ENSP00000385276<br>Bt_ENSBTAP00000039755<br>Rn_ENSRNOP00000017625<br>Mm_ENSMUSP00000033965<br>Gg_ENSGALP00000037420<br>Xt<br>Dr_ENSDARP00000020642<br>Ce<br>Dm<br>Sc	NP_001073946 Teneurin-3 (Ten-3)(Tenascin-M3)(Ten-m3)(Protein Odd Oz/ten-m homolog 3)
446	1	C	ENSP00000337926<br>ENSG00000064607	137<br>136<br>134<br>134<br>139<br>-<br>-<br>-<br>-<br>-	klghfrsqdwKFALRgsweqdfghp<br>klghfrsqdwkfalhgsweqdfahs<br>nlghfpsqdwklalrgsweqdlghs<br>nlghfpsqdwklalrgsweqdlghs<br>eyghpasrdreygrpashdqdyghp<br>.........................<br>.........................<br>.........................<br>.........................<br>.........................	Hs_ENSP00000337926<br>Bt_ENSBTAP00000017672<br>Rn_ENSRNOP00000027457<br>Mm_ENSMUSP00000091167<br>Gg_ENSGALP00000004808<br>Xt<br>Dr<br>Ce<br>Dm<br>Sc	NP_055699 Putative splicing factor, arginine/serine-rich 14 (Arginine/serine-rich-splicing factor 14)
447	1	CI	ENSP00000379340<br>ENSG00000163810	661<br>-<br>639<br>647<br>686<br>660<br>-	kctpiktgpkKFIVKlsskqvkein<br>.........................<br>ectpvktgprKFIVKfisrqvkevh<br>ectpvkagpqKFIVKfisrqvkevh<br>ictptrvgekkivarltsnqvkdis<br>icaprrpgekkivaklissqikgis<br>.........................	Hs_ENSP00000379340<br>Bt<br>Rn_ENSRNOP00000052677<br>Mm_ENSMUSP00000026893<br>Gg_ENSGALP00000019372<br>Xt_ENSXETP00000018585<br>Dr	Protein-glutamine gamma-glutamyltransferase 4 (EC 2.3.2.13)(Transglutaminase-4)(TGase-4)(Prostate transglutaminase)(TGP)(TG(P))

				-<br>-<br>-	.........................<br>.........................<br>.........................	Ce<br>Dm<br>Sc	(Prostate-specific transglutaminase)(Fibrinoligase)
448	1	C	ENSP00000375693<br>ENSG00000062822	272<br>271<br>268<br>270<br>-<br>0<br>265<br>241<br>252<br>276	gcnwlelpagKYALRlk--------<br>gcnwlelpagKYILRpe--------<br>gcnwlelpagkyvrrae--------<br>gcnwlelpagkyvrrae--------<br>.........................<br>-------------------------<br>gccwielpkgkyqlreerregqtds<br>gcgwieipagkcrilsn--------<br>gcnwielpmghwrirnshs------<br>gmswitlpkgkysmiep--------	Hs_ENSP00000375693<br>Bt_ENSBTAP00000014714<br>Rn_ENSRNOP00000026797<br>Mm_ENSMUSP00000039776<br>Gg<br>Xt_ENSXETP00000019806<br>Dr_ENSDARP00000055858<br>Ce_CE09308<br>Dm_FBpp0075277<br>Sc_YDL102W	DNA polymerase delta catalytic subunit (EC 2.7.7.7)(DNA polymerase subunit delta p125)
449	1	CI	ENSP00000337518<br>ENSG00000108592	317<br>317<br>317<br>317<br>-<br>-<br>-<br>321<br>319<br>326	sllnwrtklrRYVAKklkeqakald<br>sllnwrtklrRYVAKklkeqakald<br>sllnwrtklrRYVAKklkeqakald<br>sllnwrtklrRYVAKklkeqakald<br>.........................<br>.........................<br>.........................<br>vllrwrksml----etleeqrkave<br>glvqwwkdvr----elfveketplv<br>milrwrkiar----eilg-------	Hs_ENSP00000337518<br>Bt_ENSBTAP00000028047<br>Rn_ENSRNOP00000013295<br>Mm_ENSMUSP00000021048<br>Gg<br>Xt<br>Dr<br>Ce_CE23795<br>Dm_FBpp0073943<br>Sc_YCL054W	NP_060117 Putative rRNA methyltransferase 3 (EC 2.1.1.-)(rRNA (uridine-2'-O-)-methyltransferase 3)
450	1	CI	ENSP00000265562<br>ENSG00000076201	28<br>28<br>28<br>28<br>-<br>1<br>28<br>35<br>28<br>-	gdfhfqpavkKFVLKnygenpeayn<br>gdfhfqpavkKFVLKnygenpeayn<br>gdfhfqsavkKFVLKnygenpeayn<br>gdfhfqsavkKFVLKnygenpeayn<br>.........................<br>----------qfvlknygenpenyn<br>gefqfsptvkqfilknygedpdnyn<br>pnfdfrlrmkeyilltfnadphydd<br>pegtsfaalkkyiaefyhedpeays<br>.........................	Hs_ENSP00000265562<br>Bt_ENSBTAP00000003600<br>Rn_ENSRNOP00000052992<br>Mm_ENSMUSP00000039580<br>Gg<br>Xt_ENSXETP00000056989<br>Dr_ENSDARP00000093424<br>Ce_CE41728<br>Dm_FBpp0075450<br>Sc	NP_056281 Tyrosine-protein phosphatase non-receptor type 23 (EC 3.1.3.48)(His domain-containing protein tyrosine phosphatase)(HD-PTP)(Protein tyrosine phosphatase TD14)(PTP-TD14)
451	1	CI	ENSP00000265193<br>ENSG00000069018	789<br>790<br>851<br>790<br>790<br>-<br>785<br>-<br>-<br>-	yqkimkrlikRYVLKaqvdrendev<br>yqkimkrlikRYVLKaqvdrendev<br>yqkimkrlikRYVLKaqvdrendev<br>yqkimkrlikRYVLKaqvdrendev<br>yqkimkrlikRYVLKaqvdrendev<br>.........................<br>yqklmkrlikRYVLKaqidsesnei<br>.........................<br>.........................<br>.........................	Hs_ENSP00000265193<br>Bt_ENSBTAP00000019490<br>Rn_ENSRNOP00000048235<br>Mm_ENSMUSP00000022023<br>Gg_ENSGALP00000039397<br>Xt<br>Dr_ENSDARP00000063618<br>Ce<br>Dm<br>Sc	NP_065122 Short transient receptor potential channel 7 (TrpC7)(TRP7 protein)
452	1	CI	ENSP00000269214<br>ENSG00000141446	676<br>677<br>676<br>679<br>691<br>-<br>-<br>162<br>-<br>-	riimvlpedpKYALKkvdeiremvd<br>riimvlpedpKYALKkvdeiremvd<br>riimvlpedpKYALKkvdeiremvd<br>riimvlpedpKYALKkvdeiremvd<br>riimvlpddpKYALKkveeiremvd<br>.........................<br>.........................<br>kvyylatfadgpfkklfnehmkkin<br>.........................<br>.........................	Hs_ENSP00000269214<br>Bt_ENSBTAP00000031022<br>Rn_ENSRNOP00000032270<br>Mm_ENSMUSP00000025142<br>Gg_ENSGALP00000024046<br>Xt<br>Dr<br>Ce_CE27932<br>Dm<br>Sc	NP_443143 N-acetyltransferase ESCO1 (EC 2.3.1.-)(Establishment of cohesion 1 homolog 1)(ECO1 homolog 1)(ESO1 homolog 1)(Establishment factor-like protein 1)(EFO1p)(hEFO1)(CTF7 homolog 1)
453	1	CI	ENSP00000251472<br>ENSG00000105613	322<br>326<br>322<br>322<br>-<br>-<br>0<br>-<br>687<br>134	eghlvktdipRYIIRqlgltrdpfp<br>eghlvktdipRYIIRqlgltrdpfp<br>eghlvktdipRYIIRqlgltrdpfp<br>eghlvktdipRYIIRqlgltrdpfp<br>.........................<br>.........................<br>--------------sqlgltrdple<br>.........................<br>ampvikadipqyiihklglnrdpia<br>dn----------------iagsphm	Hs_ENSP00000251472<br>Bt_ENSBTAP00000007991<br>Rn_ENSRNOP00000004646<br>Mm_ENSMUSP00000105363<br>Gg<br>Xt<br>Dr_ENSDARP00000063937<br>Ce<br>Dm_FBpp0075315<br>Sc_YOL100W	NP_055790 Microtubule-associated serine/threonine-protein kinase 1 (EC 2.7.11.1)(Syntrophin-associated serine/threonine-protein kinase)
454	1	CI	ENSP00000338207<br>ENSG00000166407	70<br>34<br>23<br>34<br>34<br>-<br>33	cagcnrkikdRYLLKa-----ldky<br>cagcnrkikdRYLLKa-----ldqy<br>cagcnrkikdRYLLKa-----ldky<br>cagcnrkikdRYLLKa-----ldky<br>cagcnrkikdRYLLKa-----ldky<br>.........................<br>cagcnrkikdRYLLKa-----ldky	Hs_ENSP00000338207<br>Bt_ENSBTAP00000042937<br>Rn_ENSRNOP00000019690<br>Mm_ENSMUSP00000037079<br>Gg_ENSGALP00000009581<br>Xt<br>Dr_ENSDARP00000044207	NP_002306 Rhombotin-1 (LIM domain only protein 1)(Cysteine-rich protein TTG-1)(T-cell translocation protein 1)

				18<br>-<br>-	cagckakiaeedveknkvvfflnrm<br>.........................<br>.........................	Ce_CE05315<br>Dm<br>Sc	
455	1	C	ENSP00000303486<br>ENSG00000171282	1708<br>2080<br>887<br>2344<br>-<br>1555<br>-<br>-<br>-<br>-	gpglaagvpsRFLARlsvssssgs<br>gpglapgvpsRFLARlsvssssgs<br>gpglatgvpsrfltrlsmssssgs<br>gpglatgvpsrfltrlsmssssgs<br>.........................<br>egdlparvpsqflagltvssssgs<br>.........................<br>.........................<br>.........................<br>.........................	Hs_ENSP00000303486<br>Bt_ENSBTAP00000022317<br>Rn_ENSRNOP00000051860<br>Mm_ENSMUSP00000043643<br>Gg<br>Xt_ENSXETP00000032876<br>Dr<br>Ce<br>Dm<br>Sc	Unknown
456	1	CI	ENSP00000345270<br>ENSG00000128268	485<br>486<br>489<br>489<br>-<br>478<br>483<br>-<br>458<br>-	adpsehmyapKYLLKnydrfhylld<br>adpsehmyapKYLLKnydqfrylld<br>adpsehmyapKYLLKnydqfrylle<br>adpsehmyapKYLLKnydqfrylle<br>.........................<br>adphehmyapkyimentqrfsylvk<br>sdpkehmyapkymlehydlyrylle<br>.........................<br>reqsekyfapeealqhssqygqllv<br>.........................	Hs_ENSP00000345270<br>Bt_ENSBTAP00000016591<br>Rn_ENSRNOP00000023434<br>Mm_ENSMUSP00000043077<br>Gg<br>Xt_ENSXETP00000035182<br>Dr_ENSDARP00000053183<br>Ce<br>Dm_FBpp0080068<br>Sc	NP_001091740 Beta-1,4-mannosyl-glycoprotein 4-beta-N-acetylglucosaminyltransferase (EC 2.4.1.144)(N-glycosyl-oligosaccharide-glycoprotein N-acetylglucosaminyltransferase III)(N-acetylglucosaminyltransferase III)(GlcNAc-T III)(GNT-III)
457	1	CI	ENSP00000355636<br>ENSG00000135801	162<br>157<br>162<br>162<br>162<br>162<br>161<br>-<br>-<br>-	nfklrafldnKYVVRlqedsynyli<br>nfrlrafldnKYVVRlqedsysyll<br>nfqlrafldnKYVVRlqedsynyli<br>nfqlrafldnKYVVRlqedsynyli<br>nlklrafldnKYVIRlqedsynyll<br>ncklrafldnkyvvclqedsyhyll<br>npklcalldhkyvvyltdqaysyll<br>.........................<br>.........................<br>.........................	Hs_ENSP00000355636<br>Bt_ENSBTAP00000050976<br>Rn_ENSRNOP00000024491<br>Mm_ENSMUSP00000090726<br>Gg_ENSGALP00000018059<br>Xt_ENSXETP00000050395<br>Dr_ENSDARP00000038258<br>Ce<br>Dm<br>Sc	NP_055224 TAF5-like RNA polymerase II p300/CBP-associated factor-associated factor 65 kDa subunit 5L (PCAF-associated factor 65 beta)(PAF65-beta)
458	1	CI	ENSP00000335615<br>ENSG00000011114	465<br>-<br>465<br>465<br>465<br>465<br>481<br>-<br>500<br>-	ylqaseqdilKYLIKwgehqlmkri<br>.........................<br>ylqaseqdilKYLIKwgehqlmkri<br>ylqaseqdilKYLIKwgehqlmkri<br>ylqaseqdilKYLIKwgehqlmkri<br>ylqasehdilKYLIKwgeyqlmkri<br>ylqaseqdilKYVVKwgehqlikrm<br>.........................<br>flqaselevlqavlkwgeqelirrm<br>.........................	Hs_ENSP00000335615<br>Bt<br>Rn_ENSRNOP00000011518<br>Mm_ENSMUSP00000046951<br>Gg_ENSGALP00000017609<br>Xt_ENSXETP00000022696<br>Dr_ENSDARP00000013865<br>Ce<br>Dm_FBpp0073924<br>Sc	NP_001002860 BTB/POZ domain-containing protein 7
459	1	CI	ENSP00000314949<br>ENSG00000181222	417<br>-<br>417<br>417<br>-<br>-<br>414<br>411<br>409<br>403	rrgnsqypgaKYIIRdngdridlrf<br>.........................<br>rrgnsqypgaKYIIRdngdridlrf<br>rrgnsqypgaKYIIRdngdridlrf<br>.........................<br>.........................<br>rrgnsqypgaKYIIRdngdridlrf<br>nrgdtqypgak----engarvdlry<br>rrgnsqypgaKYIVRdngeridlrf<br>rngpnehpgaKYVIRdsgdridlry	Hs_ENSP00000314949<br>Bt<br>Rn_ENSRNOP00000046047<br>Mm_ENSMUSP00000050771<br>Gg<br>Xt<br>Dr_ENSDARP00000071962<br>Ce_CE28300<br>Dm_FBpp0073387<br>Sc_YDL140C	NP_000928 DNA-directed RNA polymerase II subunit RPB1 (RNA polymerase II subunit B1)(EC 2.7.7.6)(DNA-directed RNA polymerase II subunit A)(DNA-directed RNA polymerase III largest subunit)
460	1	CI	ENSP00000315674<br>ENSG00000087269	518<br>530<br>524<br>521<br>539<br>-<br>420<br>475<br>517<br>471	e--sas-daiKFVLRdamhemeemi<br>e--sas-davKFVLRdamhemegtv<br>e--sas-daiRFVLRdamhemeemi<br>e--sas-dsiRFVLRdamhemeemi<br>e--aas-gniKFVLRdaahemeevi<br>.........................<br>y--svccaqintlvqrkthtelklt<br>qyg-------vrcvralirqhwkgr<br>e--rms-----ntllevikekyeef<br>r-------elseecrdyinemqary	Hs_ENSP00000315674<br>Bt_ENSBTAP00000041827<br>Rn_ENSRNOP00000016347<br>Mm_ENSMUSP00000038382<br>Gg_ENSGALP00000025188<br>Xt<br>Dr_ENSDARP00000044183<br>Ce_CE27000<br>Dm_FBpp0082928<br>Sc_YDL148C	NP_003694 Nucleolar protein 14 (Nucleolar complex protein 14)
461	1	CI	ENSP00000309334<br>ENSG00000174748	20<br>20<br>20<br>20<br>22<br>20<br>20	lwrkkqsdvmRFLLRvrcwqyrqls<br>lwrkkqsdvmRFLLRvrcwqyrqls<br>lwrkkqsdvmRFLLRvrcwqyrqls<br>lwrkkqsdvmRFLLRvrcwqyrqls<br>lwrkkqsdvmRFLLRvrcwqyrqls<br>lwrkkqsdvmRFLLRvrcwqyrqls<br>lwrkkqsdvmRFLLRvrcwqyrqls	Hs_ENSP00000309334<br>Bt_ENSBTAP00000044213<br>Rn_ENSRNOP00000010759<br>Mm_ENSMUSP00000108217<br>Gg_ENSGALP00000036963<br>Xt_ENSXETP00000008399<br>Dr_ENSDARP00000037569	NP_002939 60S ribosomal protein L15

				20<br>49<br>20	iwrkkqsdalRYLLRirtwhyrqls<br>lyrkkqsdvmRYLLRirvwqyrqlt<br>lqrkkqsdvlrflqrvrvweyrqkn	Ce_CE12148<br>Dm_FBpp0112464<br>Sc_YLR029C	
462	1	CI	ENSP00000378174<br>ENSG00000146197	611<br>595<br>576<br>595<br>568<br>-<br>-<br>-<br>-<br>-	kmlrksinqdRFLLRlagldyelah<br>kmlrksinqdRFLLRlagldyelah<br>kmlrksinqdRFLLRlagldyelah<br>kmlrksinqdRFLLRlagldyelah<br>kalkksinqeRFLLRfagmeyevar<br>.........................<br>.........................<br>.........................<br>.........................<br>.........................	Hs_ENSP00000378174<br>Bt_ENSBTAP00000012158<br>Rn_ENSRNOP00000000593<br>Mm_ENSMUSP00000038366<br>Gg_ENSGALP00000004206<br>Xt<br>Dr<br>Ce<br>Dm<br>Sc	Signal peptide, CUB and EGF-like domain-containing protein 3 Precursor
463	1	CI	ENSP00000216264<br>ENSG00000100422	433<br>-<br>433<br>433<br>384<br>463<br>528<br>455<br>565<br>-	irkcsrfnflRFLIRhtnqq--dqf<br>.........................<br>irkcsrfnflRFLIRhtnqe--dqf<br>irkcsrfnflRFLIRhtnqe--dqf<br>vrkcsrfdflRYLVRhtnkd--dqf<br>vrkcsrldflrhlirhtsnk--dqf<br>vrkcsrvdflrhllrhtnkk--dqf<br>vprisrfhnmqfmrkvamyggkqly<br>vkktsllnnvrfllntagrsg-dir<br>.........................	Hs_ENSP00000216264<br>Bt<br>Rn_ENSRNOP00000022882<br>Mm_ENSMUSP00000038203<br>Gg_ENSGALP00000030103<br>Xt_ENSXETP00000050375<br>Dr_ENSDARP00000091226<br>Ce_CE18241<br>Dm_FBpp0078446<br>Sc	NP_073603 Ceramide kinase (hCERK)(EC 2.7.1.138)(Acylsphingosine kinase)(Lipid kinase 4)(LK4)
464	1	CI	ENSP00000378503<br>ENSG00000213588	391<br>420<br>383<br>377<br>-<br>327<br>-<br>-<br>721<br>-	---fgclcgkRFAVKpkrdrhimlt<br>---fgclcgkRFAVKpkrdrhimlt<br>---faclcgkRFAVKpkrdrhimlt<br>---faclcgkRFAVKpkrdrhimlt<br>.........................<br>---fgclcgkRFAVKpkrdrhimlt<br>.........................<br>.........................<br>nceykckelnmraircsrqqhmmsh<br>.........................	Hs_ENSP00000378503<br>Bt_ENSBTAP00000048532<br>Rn_ENSRNOP00000000574<br>Mm_ENSMUSP00000112778<br>Gg<br>Xt_ENSXETP00000043904<br>Dr<br>Ce<br>Dm_FBpp0083063<br>Sc	NP_689948 Zinc finger and BTB domain-containing protein 9
465	1	CI	ENSP00000374444<br>ENSG00000143669	2972<br>2967<br>2857<br>2958<br>1882<br>-<br>2959<br>-<br>1631<br>-	lqrcyltipnKYLLRdrqksedvvk<br>lqrcyltipnKYLLRdrqksedvvk<br>lqrcyltipnKYLLRdrqksegvlk<br>lqrcyltipnKYLLRdrqksegvlr<br>lqrcyltipnkyllpdrqkqegiik<br>.........................<br>lqrcyltipnKYLLKdrrkldd sik<br>.........................<br>lrrchldidrrffmneyrpgqghre<br>.........................	Hs_ENSP00000374444<br>Bt_ENSBTAP00000022355<br>Rn_ENSRNOP00000017270<br>Mm_ENSMUSP00000106188<br>Gg_ENSGALP00000017876<br>Xt<br>Dr_ENSDARP00000090224<br>Ce<br>Dm_FBpp0072734<br>Sc	Lysosomal-trafficking regulator (Beige homolog)
466	1	CI	ENSP00000370345<br>ENSG00000152520	662<br>-<br>608<br>608<br>659<br>708<br>655<br>552<br>713<br>606	kdptwsetgdRYLLKlfrdhlfhqv<br>.........................<br>kdptwsetgdRYLLKlfrdhlfhqv<br>kdptwsetgdRYLLKlfrdhlfhqv<br>kdptwsetgdRYLLKlfrdhlfhqv<br>kdpawsetgdRYLLKlfrdhlfhqv<br>kdptwsetgdRYLLKlfrdhlfhqv<br>tddawsetgdrfmlklfrdyvfhqv<br>ldctwsetgdrymlklfrdylfhsv<br>idinwsesgtkfpiilfydyvfhqv	Hs_ENSP00000370345<br>Bt<br>Rn_ENSRNOP00000040388<br>Mm_ENSMUSP00000082708<br>Gg_ENSGALP00000027557<br>Xt_ENSXETP00000025796<br>Dr_ENSDARP00000075759<br>Ce_CE37601<br>Dm_FBpp0072861<br>Sc_YKL025C	PAB-dependent poly(A)-specific ribonuclease subunit 3 (hPan3)
467	1	CI	ENSP00000233242<br>ENSG00000084674	1930<br>1935<br>1899<br>1899<br>1892<br>-<br>1836<br>-<br>2018<br>-	wgehtgqlysKFLLKaeplaftfsh<br>wgqhtgqlysKFLLKaeplaftfsh<br>wgehtgqmysKFLLKaeplaltfsh<br>wgehtgqlysKFLLKaeplalivsh<br>lgehtgdlyskilfkaeplaftfsh<br>.........................<br>ygkhsgqvssKFLLKaeplaiaksh<br>.........................<br>llkrk---efvfqlhgkmsnyklgv<br>.........................	Hs_ENSP00000233242<br>Bt_ENSBTAP00000038799<br>Rn_ENSRNOP00000039779<br>Mm_ENSMUSP00000035761<br>Gg_ENSGALP00000026550<br>Xt<br>Dr_ENSDARP00000062792<br>Ce<br>Dm_FBpp0079441<br>Sc	NP_000375 Apolipoprotein B-100 Precursor (Apo B-100)
468	1	CI	ENSP00000301919<br>ENSG00000171100	120<br>124<br>119<br>120<br>120<br>119<br>153	ditckdmrnlRFALKqeghsrrdmf<br>ditckdlrnlRFALKqeghsrrdmf<br>ditckdlrnlRFALKqeghsrrdif<br>ditckdlrnlRFALKqeghsrrdmf<br>ditckdmrnlRFALKqeghsrrdif<br>ditckdmrnlRFALKqevhsrkqif<br>eitckdmrnlRFALKqeghsrrdif	Hs_ENSP00000301919<br>Bt_ENSBTAP00000018789<br>Rn_ENSRNOP00000003401<br>Mm_ENSMUSP00000033700<br>Gg_ENSGALP00000014791<br>Xt_ENSXETP00000016895<br>Dr_ENSDARP00000054696	NP_000243 Myotubularin (EC 3.1.3.48)

				118<br>-<br>-	tiyckdyrvyrftcnpassdrknvc<br>.........................<br>.........................	Ce_CE30707<br>Dm<br>Sc	
469	1	C	ENSP00000260665<br>ENSG00000138095	9<br>9<br>9<br>9<br>0<br>9<br>9<br>10<br>9<br>-	--maallrsaRWLLRagaaprlpls<br>--maallrsaRWLLRataapylpls<br>--msallrparwllgaaavprlpls<br>--maallrparwllgaaaaprlpls<br>-------------------------<br>--msallagaRFLLRpglralpapc<br>--maallrsarllktssasliqvig<br>-mlglsrnglrslarrrglssaaaq<br>--masilrtgkllryfagftrnvlv<br>.........................	Hs_ENSP00000260665<br>Bt_ENSBTAP00000021773<br>Rn_ENSRNOP00000008200<br>Mm_ENSMUSP00000107927<br>Gg_ENSGALP00000016182<br>Xt_ENSXETP00000020435<br>Dr_ENSDARP00000046797<br>Ce_CE05283<br>Dm_FBpp0080637<br>Sc	NP_573566 Leucine-rich PPR motif-containing protein, mitochondrial Precursor (130 kDa leucine-rich protein)(LRP 130)(GP130)
470	1	CI	ENSP00000370388<br>ENSG00000087470	309<br>264<br>296<br>289<br>283<br>283<br>282<br>285<br>282<br>314	ypslanrngtKYLARtlnrllmhhi<br>ypslanrngtKYLARtlnrllmhhi<br>ypslanrngtKYLARtlnrllmhhi<br>ypslanrngtKYLARtlnrllmhhi<br>ypslanrngtKYLARtlnrllmhhi<br>ypslanrngtKYLARtlnrllmhhi<br>ypslanrngtKYLARtlnrllmhhi<br>yptlasrngtpylakrlnmllmhhi<br>yptlatrngtpylaktlnrllmhhi<br>yrtistkcgtRYLAKllnqtllshi	Hs_ENSP00000370388<br>Bt_ENSBTAP00000037777<br>Rn_ENSRNOP00000002478<br>Mm_ENSMUSP00000093944<br>Gg_ENSGALP00000021058<br>Xt_ENSXETP00000026804<br>Dr_ENSDARP00000006315<br>Ce_CE30173<br>Dm_FBpp0077424<br>Sc_YLL001W	Dynamin-1-like protein (EC 3.6.5.5)(Dynamin-like protein)(Dnm1p/Vps1p-like protein)(DVLP)(Dynamin family member proline-rich carboxyl-terminal domain less)(Dymple)(Dynamin-related protein 1)(Dynamin-like protein 4)(Dynamin-like protein IV)(HdynIV)
471	1	CI	ENSP00000312222<br>ENSG00000174306	816<br>804<br>540<br>811<br>726<br>794<br>945<br>-<br>-<br>-	pevvrwfgdsRYALKngqlkwyedy<br>pevvrwfgdsRYALKngqlkwyedy<br>-------------------------<br>pevvrwfgdsRYALKngqlkwyedy<br>aevirwfgdsrygykngqlkwyeny<br>tdvvrwfgdcrysfkngqlrwydry<br>pevvrwfgdcRYVLKngqlkwlesy<br>.........................<br>.........................<br>.........................	Hs_ENSP00000312222<br>Bt_ENSBTAP00000023393<br>Rn_ENSRNOP00000034076<br>Mm_ENSMUSP00000045019<br>Gg_ENSGALP00000033850<br>Xt_ENSXETP00000042167<br>Dr_ENSDARP00000005108<br>Ce<br>Dm<br>Sc	NP_055850 Zinc fingers and homeoboxes protein 3 (Zinc finger and homeodomain protein 3)(Triple homeobox protein 1)
472	1	CI	ENSP00000381800<br>ENSG00000214701	42<br>-<br>-<br>-<br>47<br>-<br>-<br>-<br>-<br>-	vlsvgfneagRYALRlsaenplqvg<br>.........................<br>.........................<br>.........................<br>vlsaqfnrrgRYALRlsvenpllrd<br>.........................<br>.........................<br>.........................<br>.........................<br>.........................	Hs_ENSP00000381800<br>Bt<br>Rn<br>Mm<br>Gg_ENSGALP00000034159<br>Xt<br>Dr<br>Ce<br>Dm<br>Sc	Unknown
473	1	CI	ENSP00000368966<br>ENSG00000138741	858<br>788<br>846<br>847<br>872<br>836<br>-<br>-<br>-<br>-	yqqimkrlikRYVLKaqvdkendev<br>yqqimkrlikRYVLKaqvdkendev<br>yqqimkrlikRYVLKaqvdkendev<br>yqqimkrlikRYVLKaqvdkendev<br>yqqimkrlikRYVLKaqvdkendev<br>yqqimkrlikRYVLRaqvdkendev<br>.........................<br>.........................<br>.........................<br>.........................	Hs_ENSP00000368966<br>Bt_ENSBTAP00000039660<br>Rn_ENSRNOP00000046270<br>Mm_ENSMUSP00000029271<br>Gg_ENSGALP00000019344<br>Xt_ENSXETP00000057914<br>Dr<br>Ce<br>Dm<br>Sc	NP_001124170 Short transient receptor potential channel 3 (TrpC3)(Transient receptor protein 3)(TRP-3)(hTrp-3)(hTrp3)
474	1	CI	ENSP00000354421<br>ENSG00000198855	416<br>416<br>416<br>416<br>411<br>412<br>406<br>457<br>428<br>-	anegdvrpfiRFIAKctettldtll<br>anegdvrpfiRFIAKctettldtll<br>anegdvrpfiRFIAKctevtldtll<br>anegdvrpfiRFIAKctevtldtll<br>anegdvrpfiRFIAKctettldmll<br>anegdvrpfiRFIAKctestldlll<br>anegdvrpfiRFIAKctemtldtll<br>anlgdlrpfvRYVAKhseasiqryi<br>anegdirpfvrfiadctektldlyl<br>.........................	Hs_ENSP00000354421<br>Bt_ENSBTAP00000015620<br>Rn_ENSRNOP00000000892<br>Mm_ENSMUSP00000071719<br>Gg_ENSGALP00000007747<br>Xt_ENSXETP00000038311<br>Dr_ENSDARP00000051605<br>Ce_CE28722<br>Dm_FBpp0078887<br>Sc	NP_009007 FIC domain-containing protein (Huntingtin-interacting protein E)(Huntingtin yeast partner E)(Huntingtin-interacting protein 13)
475	1	CI	ENSP00000375951<br>ENSG00000135912	717<br>716<br>715<br>712<br>-<br>300	awe--sssrqKWIVKppasargigi<br>awe--sgsrqKWIVKppasargigi<br>awe--sssrqKWIVKppasargigi<br>awe--sssrqKWIVKppasargigi<br>.........................<br>awee-ggnrqKWIVKppasargmgi	Hs_ENSP00000375951<br>Bt_ENSBTAP00000024502<br>Rn_ENSRNOP00000023067<br>Mm_ENSMUSP00000037406<br>Gg<br>Xt_ENSXETP00000030386	NP_055455 Tubulin polyglutamylase TTLL4 (EC 6.-.-.-)(Tubulin--tyrosine ligase-like protein 4)

				-<br>-<br>569<br>-	.........................<br>.........................<br>vwpknaskltKWIVKppasargtgi<br>.........................	Dr<br>Ce<br>Dm_FBpp0077190<br>Sc	
475	2	CI	ENSP00000375951<br>ENSG00000135912	833<br>832<br>831<br>828<br>-<br>416<br>-<br>-<br>685<br>-	nademacqghKWALKalwnylsqkg<br>nedetacqghKWALKalwsylsqkg<br>nadetacqghKWALKalwnylsqkg<br>nadetacqghKWALKalwnylsqkg<br>.........................<br>npdqtacqghKWALKalwnylnqkg<br>.........................<br>.........................<br>nedfnacqghkwtlqslwsclenrg<br>.........................	Hs_ENSP00000375951<br>Bt_ENSBTAP00000024502<br>Rn_ENSRNOP00000023067<br>Mm_ENSMUSP00000037406<br>Gg<br>Xt_ENSXETP00000030386<br>Dr<br>Ce<br>Dm_FBpp0077190<br>Sc	NP_055455 Tubulin polyglutamylase TTLL4 (EC 6.-.-.-)(Tubulin--tyrosine ligase-like protein 4)
476	1	CI	ENSP00000385169<br>ENSG00000179912	254<br>254<br>254<br>254<br>255<br>249<br>-<br>239<br>-<br>108	kdekntefqqRFILKrddasmdrdd<br>kdekntefqqRFILKrddasmdrdd<br>kdekntefqqRFILKrddasmdree<br>kdekntefqqRFILKrddasmdrdd<br>kdeknaefpqRFILKrddtsmdrdd<br>kdekscefqqRFILKrddasmdrdd<br>.........................<br>rhdn---------------------<br>.........................<br>qlnkkpe------------------	Hs_ENSP00000385169<br>Bt_ENSBTAP00000024431<br>Rn_ENSRNOP00000031193<br>Mm_ENSMUSP00000076303<br>Gg_ENSGALP00000023145<br>Xt_ENSXETP00000037400<br>Dr<br>Ce_CE23454<br>Dm<br>Sc_YDL189W	R3H domain-containing protein 2
477	1	CI	ENSP00000375913<br>ENSG00000120332	511<br>471<br>472<br>472<br>466<br>-<br>436<br>-<br>-<br>-	swdpvqavidKYVVRytsadgdtke<br>swdpvravidkymvryisadgetkd<br>swdpvradidKYVVRyishdgetke<br>swdpvradidKYVVRyiapdgetke<br>swn----------------------<br>.........................<br>lydipmrktdiflvtinqvglehaf<br>.........................<br>.........................<br>.........................	Hs_ENSP00000375913<br>Bt_ENSBTAP00000020785<br>Rn_ENSRNOP00000003452<br>Mm_ENSMUSP00000039452<br>Gg_ENSGALP00000007212<br>Xt<br>Dr_ENSDARP00000089509<br>Ce<br>Dm<br>Sc	NP_071376 Tenascin-N Precursor (TN-N)
477	2	CI	ENSP00000375913<br>ENSG00000120332	776<br>735<br>824<br>824<br>522<br>-<br>711<br>-<br>-<br>-	swdpvratidRYVVRytsak-----<br>swdpvqavidRYLVRytsa------<br>swdpveadidRYVVRytsvdgetre<br>swdpveadidRYVVRytsvdgetre<br>-------------------------<br>.........................<br>ftsahladvqsillrvlsas-----<br>.........................<br>.........................<br>.........................	Hs_ENSP00000375913<br>Bt_ENSBTAP00000020785<br>Rn_ENSRNOP00000003452<br>Mm_ENSMUSP00000039452<br>Gg_ENSGALP00000007212<br>Xt<br>Dr_ENSDARP00000089509<br>Ce<br>Dm<br>Sc	NP_071376 Tenascin-N Precursor (TN-N)
477	3	C	ENSP00000375913<br>ENSG00000120332	796<br>754<br>1000<br>1000<br>522<br>-<br>729<br>-<br>-<br>-	-------------------------<br>-------------------------<br>swdpvqadidRYVVRytsadgeske<br>swdpvqadidRYVVRytsadgeske<br>-------------------------<br>.........................<br>-------------------------<br>.........................<br>.........................<br>.........................	Hs_ENSP00000375913<br>Bt_ENSBTAP00000020785<br>Rn_ENSRNOP00000003452<br>Mm_ENSMUSP00000039452<br>Gg_ENSGALP00000007212<br>Xt<br>Dr_ENSDARP00000089509<br>Ce<br>Dm<br>Sc	NP_071376 Tenascin-N Precursor (TN-N)
478	1	CI	ENSP00000269554<br>ENSG00000141720	146<br>155<br>146<br>146<br>103<br>152<br>-<br>-<br>139<br>492	gtrflttydrRFVIKtvssedvaem<br>gtrflttydrRFVIKtvssedvaem<br>gtrflttydrRFVIKtvssedvaem<br>gtrflttydrRFVIKtvssedvaem<br>garflttydrRFVIKavssedvaem<br>gsrflttydrRFVIKtissedvaem<br>.........................<br>.........................<br>gaqfyqsydkffiiksltseeierm<br>gsffyysrdyKYIIKtihhsehihl	Hs_ENSP00000269554<br>Bt_ENSBTAP00000008517<br>Rn_ENSRNOP00000017989<br>Mm_ENSMUSP00000018691<br>Gg_ENSGALP00000002449<br>Xt_ENSXETP00000051185<br>Dr<br>Ce<br>Dm_FBpp0099992<br>Sc_YDR208W	NP_003550 Phosphatidylinositol-5-phosphate 4-kinase type-2 beta (EC 2.7.1.149)(Phosphatidylinositol-5-phosphate 4-kinase type II beta)(1-phosphatidylinositol-5-phosphate 4-kinase 2-beta)(PtdIns(5)P-4-kinase isoform 2-beta)(PIP4KII-beta)(Diphosphoinositide kinase 2-beta)
479	1	CI	ENSP00000348573<br>ENSG00000127914	3779<br>3773<br>3637<br>3651	vrvsiaisrmKFLVRrwhrvtgsvs<br>vrvsmaisrmKFLVRrwhrvtssgs<br>vrvsiaisrmKFLVRrwqqvtstss<br>vrvsiaisrmKFLVRrwqqvtstss	Hs_ENSP00000348573<br>Bt_ENSBTAP00000009791<br>Rn_ENSRNOP00000010118<br>Mm_ENSMUSP00000046129	NP_005742 A-kinase anchor protein 9 (Protein kinase A-anchoring protein 9)(PRKA9)(A-

				3786<br>-<br>-<br>-<br>-<br>-	vrvsiaisrmKFLVRrwhrvtgsgi<br>.........................<br>.........................<br>.........................<br>.........................<br>.........................	Gg_ENSGALP00000015160<br>Xt<br>Dr<br>Ce<br>Dm<br>Sc	kinase anchor protein 450 kDa)(AKAP 450)(A-kinase anchor protein 350 kDa)(AKAP 350)(hgAKAP 350)(AKAP 120-like protein)(Protein hyperion)(Protein yotiao)(Centrosome- and Golgi-localized PKN-associated protein)(CG-NAP)
480	1	CI	ENSP00000265044<br>ENSG00000114850	81<br>81<br>0<br>81<br>83<br>81<br>81<br>76<br>86<br>-	ylvafayknvKFVLKhkvaqkreda<br>ylvafayknvKFVLKhkvaqkreda<br>------------vlsfrvaqkreda<br>ylvafayknvKFVLKhkvaqkreda<br>ylvafayknvKFVLKhkvaqkreda<br>ylvafayknvKFVLKhkvaqkreda<br>ylvafayknvKFVLKhkvaqkreda<br>yllslacknqkcllkhqivmkrgsa<br>ylmatayknikfqlkhkiagrreea<br>.........................	Hs_ENSP00000265044<br>Bt_ENSBTAP00000024583<br>Rn_ENSRNOP00000014912<br>Mm_ENSMUSP00000029414<br>Gg_ENSGALP00000016682<br>Xt_ENSXETP00000057703<br>Dr_ENSDARP00000023871<br>Ce_CE21634<br>Dm_FBpp0079495<br>Sc	NP_009038 Translocon-associated protein subunit gamma (TRAP-gamma)(Signal sequence receptor subunit gamma)(SSR-gamma)
481	1	C	ENSP00000234389<br>ENSG00000116032	336<br>341<br>333<br>336<br>-<br>338<br>-<br>-<br>-<br>-	lqpagpespgRFLARflantsfqgr<br>lpppepessgrlltrflantsfwgr<br>lktggseatgrtlarflgntsfqgr<br>lktggsestartlarflsntsfqgr<br>.........................<br>kqtkqnessgsylsrflantsfsgq<br>.........................<br>.........................<br>.........................<br>.........................	Hs_ENSP00000234389<br>Bt_ENSBTAP00000035962<br>Rn_ENSRNOP00000017064<br>Mm_ENSMUSP00000048576<br>Gg<br>Xt_ENSXETP00000046705<br>Dr<br>Ce<br>Dm<br>Sc	NP_619635 Glutamate
482	1	CI	ENSP00000262450<br>ENSG00000116254	1787<br>1762<br>1781<br>1785<br>1707<br>-<br>1715<br>-<br>-<br>-	hkgnylemknKFLARrfkllleqalv<br>hkgnylemknKFLARrfkllleqalv<br>hkgnylemknKFLARrfkllleqalv<br>hkgnylemknKFLARrfkllleqalv<br>hkgnylemknKFLARrfkascrs--<br>.........................<br>hkgnylemknKFLARrfkllleqalv<br>.........................<br>.........................<br>.........................	Hs_ENSP00000262450<br>Bt_ENSBTAP00000010688<br>Rn_ENSRNOP00000024732<br>Mm_ENSMUSP00000030775<br>Gg_ENSGALP00000023295<br>Xt<br>Dr_ENSDARP00000047289<br>Ce<br>Dm<br>Sc	NP_056372 Chromodomain-helicase-DNA-binding protein 5 (CHD-5)(EC 3.6.1.-)(ATP-dependent helicase CHD5)
483	1	CI	ENSP00000351015<br>ENSG00000196712	609<br>590<br>589<br>609<br>609<br>-<br>611<br>-<br>648<br>683	wlreilicrnKFLLKnkqadrssch<br>wlreilicrnKFLLKnkqadrssch<br>wlreilicrnKFLLKnkqadrssch<br>wlreilicrnKFLLKnkqadrssch<br>wlreilicrnKFLLKnkqsdrtsch<br>.........................<br>wlreilicrnKFLLKnkvktivice<br>.........................<br>wlreilicrntflqrhkd-------<br>sirsilflmtmtssisqidsniasv	Hs_ENSP00000351015<br>Bt_ENSBTAP00000015699<br>Rn_ENSRNOP00000049106<br>Mm_ENSMUSP00000071289<br>Gg_ENSGALP00000009067<br>Xt<br>Dr_ENSDARP00000074778<br>Ce<br>Dm_FBpp0084326<br>Sc_YBR140C	NP_001035957 Neurofibromin (Neurofibromatosis-related protein NF-1)
484	1	CI	ENSP00000376644<br>ENSG00000071794	1013<br>962<br>953<br>953<br>-<br>-<br>894<br>-<br>-<br>1124	lgqkqeviitKFIVKdsveenmlki<br>lgqkqeviitKFIVKdsveenmlki<br>lgqkqeviitKFIVKdsveenmlki<br>lgqkqeviitKFIVKdsveenmlki<br>.........................<br>.........................<br>lgqsrdvvitKFIVKdsveenmvki<br>.........................<br>.........................<br>igqtnsvkvmrfiiqdsieekmlri	Hs_ENSP00000376644<br>Bt_ENSBTAP00000034398<br>Rn_ENSRNOP00000000095<br>Mm_ENSMUSP00000002502<br>Gg<br>Xt<br>Dr_ENSDARP00000032185<br>Ce<br>Dm<br>Sc_YLR032W	NP_620636 Helicase-like transcription factor (EC 3.6.1.-)(SWI/SNF-related matrix-associated actin-dependent regulator of chromatin subfamily A member 3)(Sucrose nonfermenting protein 2-like 3)(DNA-binding protein/plasminogen activator inhibitor 1 regulator)(HIP116)(RING finger protein 80)
485	1	CI	ENSP00000256935<br>ENSG00000134516	745<br>747<br>495<br>745<br>747<br>744<br>745<br>-	rtlkaleyvfKFIVRsrtlfsqlye<br>rtlkaleyvfKFIVRsrtlfsqlye<br>rtlkaleyvfKFIVRsrrlfsqlye<br>rtlkaleyvfKFIVRsrtlfsqlye<br>rtlkaleyvfKFIVRsrtlfsqlye<br>rtlkaleyvfKFIVRsrtlfsqlye<br>rtlkaleyifKFIVRsrmlyshllk<br>.........................	Hs_ENSP00000256935<br>Bt_ENSBTAP00000019460<br>Rn_ENSRNOP00000009525<br>Mm_ENSMUSP00000090884<br>Gg_ENSGALP00000003234<br>Xt_ENSXETP00000043103<br>Dr_ENSDARP00000097023<br>Ce	NP_004937 Dedicator of cytokinesis protein 2

				-<br>-	.........................<br>.........................	Dm<br>Sc	
486	1	CI	ENSP00000308165<br>ENSG00000135218	96<br>96<br>96<br>96<br>96<br>98<br>97<br>-<br>-<br>-	gpytyr---vRFLAKenvtqdaedn<br>gpytyr---vRYLAKenitqdpeth<br>gpytyr---vRYLAKenitqdpkds<br>gpytyr---vRYLAKenitqdpedh<br>gpytyr---vrylpkenitenp-ng<br>gpytyriqsvrylpkenitqle-nn<br>gpytyr---vryipktnitfnd-nn<br>.........................<br>.........................<br>.........................	Hs_ENSP00000308165<br>Bt_ENSBTAP00000023750<br>Rn_ENSRNOP00000008319<br>Mm_ENSMUSP00000080974<br>Gg_ENSGALP00000013734<br>Xt_ENSXETP00000017231<br>Dr_ENSDARP00000050752<br>Ce<br>Dm<br>Sc	NP_001001547 Platelet glycoprotein 4 (Platelet glycoprotein IV)(GPIV)(Glycoprotein IIIb)(GPIIIB)(Leukocyte differentiation antigen CD36)(PAS IV)(PAS-4)(Platelet collagen receptor)(Fatty acid translocase)(FAT)(Thrombospondin receptor)(CD36 antigen)
487	1	CI	ENSP00000373601<br>ENSG00000157703	76<br>50<br>202<br>202<br>201<br>173<br>219<br>-<br>-<br>-	asviiptigwRWLIRvasipgiili<br>asvvvptigwRWLIRiasipgiili<br>asvviptigwRWLIRiasipgiili<br>asvviptigwRWLIRiasipgiili<br>asvvnptigwRWLIRiasipgiili<br>gsvliptlgwRWLIRfasipgiili<br>gmtvvptmgwrwmirfsvipslvli<br>.........................<br>.........................<br>.........................	Hs_ENSP00000373601<br>Bt_ENSBTAP00000041790<br>Rn_ENSRNOP00000017909<br>Mm_ENSMUSP00000093743<br>Gg_ENSGALP00000020873<br>Xt_ENSXETP00000035010<br>Dr_ENSDARP00000075190<br>Ce<br>Dm<br>Sc	NP_777619 Putative transporter SVOPL (SVOP-like protein)(SV2-related protein-like)
488	1	CI	ENSP00000263963<br>ENSG00000114757	428<br>365<br>417<br>461<br>405<br>-<br>399<br>-<br>-<br>-	knwikqnpkyKYLVKsk---kgspg<br>knwikqnpkyKYLVKsk---kgspg<br>knwikqnpkyKYLVKnk---kgspg<br>knwikqnpkyKYLVKnk---kgspg<br>rnwikqnpkyKYIAKsk---kgspa<br>.........................<br>lgwirhnpkykhllksrthlqgspg<br>.........................<br>.........................<br>.........................	Hs_ENSP00000263963<br>Bt_ENSBTAP00000017592<br>Rn_ENSRNOP00000038305<br>Mm_ENSMUSP00000103860<br>Gg_ENSGALP00000014497<br>Xt<br>Dr_ENSDARP00000095068<br>Ce<br>Dm<br>Sc	NP_057643 PEX5-related protein (Peroxisome biogenesis factor 5-like)(Peroxin-5-related protein)(Pex5Rp)(PEX5-like protein)(PEX2-related protein)
489	1	CI	ENSP00000356218<br>ENSG00000159348	46<br>46<br>46<br>46<br>-<br>-<br>45<br>-<br>-<br>72	pqvtlldpneKYLLRlldkttvshn<br>ppvtlldpnekyqlrlldkttvnhn<br>pqvtlqdpdeKYLLRlldkttvshn<br>pqvtlqdpdeKYLLRlldkttvshn<br>.........................<br>.........................<br>ilitlidpsekyklrlvdkeiishd<br>.........................<br>.........................<br>krrrslypdrwtsleledqtiiskn	Hs_ENSP00000356218<br>Bt_ENSBTAP00000026548<br>Rn_ENSRNOP00000057400<br>Mm_ENSMUSP00000027726<br>Gg<br>Xt<br>Dr_ENSDARP00000003519<br>Ce<br>Dm<br>Sc_YML125C	NP_057327 NADH-cytochrome b5 reductase 1 (b5R.1)(EC 1.6.2.2)(NAD(P)H:quinone oxidoreductase type 3 polypeptide A2)(Humb5R2)
490	1	CI	ENSP00000363200<br>ENSG00000138293	429<br>440<br>436<br>440<br>427<br>-<br>416<br>-<br>-<br>-	dencekealyKWLLKkegkdkngmp<br>dencekealcKWLLKkegkdkngmp<br>ddscekeamhKWLLKkegkdkngmp<br>ddncekeavyKWLLKkggkdkngmp<br>dencgkealcKWLLKkegkdkngvp<br>.........................<br>edncgkealsawllkkegrdkngvp<br>.........................<br>.........................<br>.........................	Hs_ENSP00000363200<br>Bt_ENSBTAP00000026068<br>Rn_ENSRNOP00000026803<br>Mm_ENSMUSP00000111743<br>Gg_ENSGALP00000003210<br>Xt<br>Dr_ENSDARP00000013664<br>Ce<br>Dm<br>Sc	NP_005428 Nuclear receptor coactivator 4 (NCoA-4)(70 kDa androgen receptor coactivator)(70 kDa AR-activator)(Ret-activating protein ELE1)
490	2	CI	ENSP00000363200<br>ENSG00000138293	558<br>570<br>566<br>569<br>562<br>-<br>534<br>-<br>-<br>-	nlsqlssgedKWLLRkkaqevllns<br>nlsqlssgedKWLLRkkakgvllds<br>nlsqfssgedKWLLRkkaqevflns<br>slsqfpsgedKWLLRkkaqeaflns<br>n-dnpppvedkwllhkkaqe-----<br>.........................<br>qdgkdnveedKWLLRkrvsa-----<br>.........................<br>.........................<br>.........................	Hs_ENSP00000363200<br>Bt_ENSBTAP00000026068<br>Rn_ENSRNOP00000026803<br>Mm_ENSMUSP00000111743<br>Gg_ENSGALP00000003210<br>Xt<br>Dr_ENSDARP00000013664<br>Ce<br>Dm<br>Sc	NP_005428 Nuclear receptor coactivator 4 (NCoA-4)(70 kDa androgen receptor coactivator)(70 kDa AR-activator)(Ret-activating protein ELE1)
491	1	CI	ENSP00000298728<br>ENSG00000181090	792<br>815<br>815<br>821<br>773<br>784	aaennhleavKYLIKagalvdpkda<br>aaennhldavKYLIKagalvdpkda<br>aaennhldavKYLIKagaqvdpkda<br>aaennhldavKYLIKagaqvdpkda<br>aaennhletvKYLIKagalvdpkda<br>aaennhldtvKYLVKggalidpkds	Hs_ENSP00000298728<br>Bt_ENSBTAP00000016118<br>Rn_ENSRNOP00000030189<br>Mm_ENSMUSP00000100002<br>Gg_ENSGALP00000013792<br>Xt_ENSXETP00000036915	NP_079033 Histone-lysine N-methyltransferase, H3 lysine-9 specific 5 (EC 2.1.1.43)(Histone H3-K9 methyltransferase 5)(H3-K9-HMTase 5)(Euchromatic

				577<br>-<br>-<br>-	acnnnhlenvKYLLKagassahkdm<br>.........................<br>.........................<br>.........................	Dr_ENSDARP00000089135<br>Ce<br>Dm<br>Sc	histone-lysine N-methyltransferase 1)(Eu-HMTase1)(G9a-like protein 1)(GLP1)(Lysine N-methyltransferase 1D)
492	1	CI	ENSP00000263967<br>ENSG00000121879	249<br>249<br>244<br>249<br>250<br>166<br>-<br>343<br>-<br>-	klcvleyqg-KYILKvcgcdeyfle<br>klcvleyqg-KYILKvcgcdeyfle<br>klcvleyqg-KYILKvcgcdeyfle<br>klcvleyqg-KYILKvcgcdeyfle<br>klcvleyqg-KYILKvcgcdeylle<br>klcvleyqg-KYILKvcgcdeylle<br>.........................<br>dvkltsydgvrselesyrcpgfvvr<br>.........................<br>.........................	Hs_ENSP00000263967<br>Bt_ENSBTAP00000012168<br>Rn_ENSRNOP00000037275<br>Mm_ENSMUSP00000103878<br>Gg_ENSGALP00000038717<br>Xt_ENSXETP00000015756<br>Dr<br>Ce_CE43431<br>Dm<br>Sc	NP_006209 Phosphatidylinositol-4,5-bisphosphate 3-kinase catalytic subunit alpha isoform (EC 2.7.1.153)(PI3-kinase p110 subunit alpha)(PtdIns-3-kinase p110)(PI3K)
492	2	CI	ENSP00000263967<br>ENSG00000121879	651<br>651<br>635<br>651<br>652<br>568<br>-<br>738<br>-<br>-	yeqyldnllvRFLLKkaltnqrigh<br>yeqyldnllvRFLLKkaltnqrigh<br>yeqyldnllvRFLLKkaltnqrigh<br>yeqyldnllvRFLLKkaltnqrigh<br>yeqyldnqlvRFLLKkaltnqrigh<br>yeqyldnhlvRFLLKkalvnqrigh<br>.........................<br>yepraqsevgmmlltralcdyrigh<br>.........................<br>.........................	Hs_ENSP00000263967<br>Bt_ENSBTAP00000012168<br>Rn_ENSRNOP00000037275<br>Mm_ENSMUSP00000103878<br>Gg_ENSGALP00000038717<br>Xt_ENSXETP00000015756<br>Dr<br>Ce_CE43431<br>Dm<br>Sc	NP_006209 Phosphatidylinositol-4,5-bisphosphate 3-kinase catalytic subunit alpha isoform (EC 2.7.1.153)(PI3-kinase p110 subunit alpha)(PtdIns-3-kinase p110)(PI3K)
493	1	CI	ENSP00000370231<br>ENSG00000155158	490<br>393<br>-<br>449<br>394<br>389<br>-<br>418<br>-<br>-	riagksipteKFAVRkarrysaslp<br>riagksipteKFAVRkarryspslp<br>.........................<br>riagkslpteKFAVRkarrysppsg<br>riagksipteKFAVRkarryasaq-<br>riagksipteKFAVRksrryasdn-<br>.........................<br>riagksipvekycgrkakrftttn-<br>.........................<br>.........................	Hs_ENSP00000370231<br>Bt_ENSBTAP00000032361<br>Rn<br>Mm_ENSMUSP00000099887<br>Gg_ENSGALP00000008685<br>Xt_ENSXETP00000007165<br>Dr<br>Ce_CE01846<br>Dm<br>Sc	NP_689787 Tetratricopeptide repeat protein 39B (TPR repeat protein 39B)
494	1	C	ENSP00000301807<br>ENSG00000168016	1703<br>1811<br>674<br>634<br>1800<br>-<br>-<br>-<br>-<br>-	kpnyksfrfyRFALKeyihflfene<br>kphyksfrsyrsalkeyihvlfqne<br>kanykffrsyRFALKeyihdlfqke<br>kpnyksfrsfrcalkeyihdlfqke<br>gfsnitskpcrallneyisfkfkde<br>.........................<br>.........................<br>.........................<br>.........................<br>.........................	Hs_ENSP00000301807<br>Bt_ENSBTAP00000028598<br>Rn_ENSRNOP00000040406<br>Mm_ENSMUSP00000077697<br>Gg_ENSGALP00000019669<br>Xt<br>Dr<br>Ce<br>Dm<br>Sc	Unknown
495	1	CI	ENSP00000368030<br>ENSG00000197785	539<br>490<br>490<br>490<br>498<br>487<br>426<br>-<br>497<br>-	rerlvrmyfdKYVLKpategkqrlk<br>rerlvrmyfdKYVLKpategkqrlk<br>rerlvrmyfdKYVLKpategkqrlk<br>rerlvrmyfdKYVLKpategkqrlk<br>rerlvrmyfdrhvlkpategkqrlk<br>rerlvrlyfdkyvlqpasegkqrlk<br>rerlvrlyfdryvlepatggrqrlk<br>.........................<br>rerllrlyfdkyvlqpaaagakrfk<br>.........................	Hs_ENSP00000368030<br>Bt_ENSBTAP00000012676<br>Rn_ENSRNOP00000046842<br>Mm_ENSMUSP00000030903<br>Gg_ENSGALP00000002302<br>Xt_ENSXETP00000028394<br>Dr_ENSDARP00000096661<br>Ce<br>Dm_FBpp0082728<br>Sc	NP_060658 ATPase family AAA domain-containing protein 3A
496	1	CI	ENSP00000354724<br>ENSG00000185619	187<br>187<br>186<br>186<br>190<br>187<br>-<br>-<br>166<br>-	saqatvlhlkKFIAKkln--lssfn<br>saqatvlhlkKFIAKkln--lssfn<br>saqatvlhlkKFIAKkln--lssfn<br>saqatvlhlkKFIAKkln--lssfn<br>saqatvlhlkKFIAKkln--lssfn<br>saqatvlhlkKFIAKkln--lssfn<br>.........................<br>.........................<br>ssqatithlkklvakkilngiekyr<br>.........................	Hs_ENSP00000354724<br>Bt_ENSBTAP00000051366<br>Rn_ENSRNOP00000000071<br>Mm_ENSMUSP00000041790<br>Gg_ENSGALP00000024701<br>Xt_ENSXETP00000010700<br>Dr<br>Ce<br>Dm_FBpp0075071<br>Sc	NP_006306 Polycomb group RING finger protein 3 (RING finger protein 3A)
497	1	CI	ENSP00000302166<br>ENSG00000169427	137<br>-<br>137<br>137<br>137	slgermntfvRYLLKrikkccgmrn<br>.........................<br>slgermntfvRYLLKrikkccgmrn<br>slgermntfvRYLLKrikkccgmrn<br>slgermntfvKYLLKrikkccgmrs	Hs_ENSP00000302166<br>Bt<br>Rn_ENSRNOP00000012408<br>Mm_ENSMUSP00000038729<br>Gg_ENSGALP00000026055	NP_057685 Potassium channel subfamily K member 9 (Acid-sensitive potassium channel protein TASK-3)(TWIK-related

				137<br>43<br>137<br>138<br>-	slgermntfvRFLLKklkrcfrlrk<br>slgermntfvKYLLKrikkccgmri<br>sigermntfaakllrfirraagkqp<br>sigerlnkfasviirrakrasgarc<br>.........................	Xt_ENSXETP00000049379<br>Dr_ENSDARP00000087420<br>Ce_CE28297<br>Dm_FBpp0081523<br>Sc	acid-sensitive K(+) channel 3) (Two pore potassium channel KT3.2)
498	1	CI	ENSP00000379531<br>ENSG00000103534	454<br>252<br>-<br>416<br>116<br>-<br>103<br>-<br>-<br>-	tsvlsyfnflRWLLKfnifsfilnf<br>tsvlsyfnflRWLLKfnifsfivtf<br>.........................<br>tsvlsyfsflRWLLKfnifsfvmnf<br>tsvlsyfiflkwllnfnifsflinf<br>.........................<br>tsvlsyflflKWLLKfnvfsfvinf<br>.........................<br>.........................<br>.........................	Hs_ENSP00000379531<br>Bt_ENSBTAP00000037277<br>Rn<br>Mm_ENSMUSP00000112434<br>Gg_ENSGALP00000011186<br>Xt<br>Dr_ENSDARP00000021162<br>Ce<br>Dm<br>Sc	NP_001098718 Transmembrane channel-like protein 5
499	1	CI	ENSP00000261200<br>ENSG00000069431	593<br>593<br>-<br>591<br>596<br>599<br>599<br>-<br>618<br>-	tplfllstvvRFAVKaiisvqklne<br>tplfllstvvRFAVKaivsvqklne<br>.........................<br>tplfllstvvRFAVKaiisvqklne<br>tplfllstvvRFAVKaivsvqklne<br>tplfllstvvRFAVKalvsvqklne<br>tplfllstvvRFAVKalvsvqklse<br>.........................<br>vpllifpitvpiiiaarvstrrler<br>.........................	Hs_ENSP00000261200<br>Bt_ENSBTAP00000040663<br>Rn<br>Mm_ENSMUSP00000098390<br>Gg_ENSGALP00000021593<br>Xt_ENSXETP00000001948<br>Dr_ENSDARP00000014386<br>Ce<br>Dm_FBpp0288707<br>Sc	NP_064693 ATP-binding cassette transporter sub-family C member 9 (Sulfonylurea receptor 2)
500	1	CI	ENSP00000296046<br>ENSG00000163751	406<br>407<br>406<br>406<br>-<br>-<br>-<br>-<br>-<br>-	ptcretmlavKFIAKyilkhts---<br>ptcketmlalKFIVKyilkhts---<br>ptcketmlsvKFIAKyilkhts---<br>ptcketmlsvKFIAKyilknts---<br>.........................<br>.........................<br>.........................<br>.........................<br>.........................<br>.........................	Hs_ENSP00000296046<br>Bt_ENSBTAP00000019982<br>Rn_ENSRNOP00000014970<br>Mm_ENSMUSP00000001921<br>Gg<br>Xt<br>Dr<br>Ce<br>Dm<br>Sc	NP_001861 Mast cell carboxypeptidase A Precursor (MC-CPA)(EC 3.4.17.1) (Carboxypeptidase A3)
500	2	CI	ENSP00000296046<br>ENSG00000163751	410<br>411<br>410<br>410<br>-<br>-<br>-<br>-<br>-<br>-	etmlavkfiaKYILKhts-------<br>etmlalkfivKYILKhts-------<br>etmlsvkfiaKYILKhts-------<br>etmlsvkfiaKYILKnts-------<br>.........................<br>.........................<br>.........................<br>.........................<br>.........................<br>.........................	Hs_ENSP00000296046<br>Bt_ENSBTAP00000019982<br>Rn_ENSRNOP00000014970<br>Mm_ENSMUSP00000001921<br>Gg<br>Xt<br>Dr<br>Ce<br>Dm<br>Sc	NP_001861 Mast cell carboxypeptidase A Precursor (MC-CPA)(EC 3.4.17.1) (Carboxypeptidase A3)
501	1	CI	ENSP00000323246<br>ENSG00000165891	153<br>152<br>154<br>154<br>151<br>151<br>158<br>-<br>-<br>-	kqkslgllcqKFLARypsyplstek<br>kqkslgllcqKFLARypsyplstek<br>kqkslgllcqKFLARypsyplstek<br>kqkslgllcqKFLARypsyplstek<br>kqkslgllcqKFLARypsyplstek<br>kqkslgllcqKFLARypsypistek<br>kqkslgllcqkflalypdypesses<br>.........................<br>.........................<br>.........................	Hs_ENSP00000323246<br>Bt_ENSBTAP00000022518<br>Rn_ENSRNOP00000030798<br>Mm_ENSMUSP00000073453<br>Gg_ENSGALP00000016680<br>Xt_ENSXETP00000031323<br>Dr_ENSDARP00000020301<br>Ce<br>Dm<br>Sc	NP_976328 Transcription factor E2F7 (E2F-7)
502	1	CI	ENSP00000371789<br>ENSG00000177764	165<br>165<br>161<br>161<br>-<br>76<br>175<br>134<br>-<br>-	a---agpgkgRFLVRicfqgdeg-a<br>agsaagpgkgRFLVRicfqgdeg-a<br>g-----pgkgRFLVRicfqgdes-a<br>g-----pgkgRFLVRicfqgdes-a<br>.........................<br>-----ipsnrqncvklvwggkke-e<br>lpsmivlgenrgyihyqgmpklcrk<br>t-----tnfkegeiiikkevedgki<br>.........................<br>.........................	Hs_ENSP00000371789<br>Bt_ENSBTAP00000002733<br>Rn_ENSRNOP00000009859<br>Mm_ENSMUSP00000096813<br>Gg<br>Xt_ENSXETP00000012312<br>Dr_ENSDARP00000058430<br>Ce_CE27916<br>Dm<br>Sc	NP_149080 Zinc finger CCHC domain-containing protein 3
503	1	CI	ENSP00000340226<br>ENSG00000162761	45<br>0<br>45<br>45<br>45	cegcqrvildRFLLRlndsfwheqc<br>-------------------------<br>cegcqrvisdRFLLRlndsfwheqc<br>cegcqrvisdRFLLRlndsfwheqc<br>cegcqrviadRFLLRlndslwherc	Hs_ENSP00000340226<br>Bt_ENSBTAP00000015947<br>Rn_ENSRNOP00000006163<br>Mm_ENSMUSP00000107008<br>Gg_ENSGALP00000005401	NP_796373 LIM homeobox transcription factor 1 alpha (LIM/homeobox protein LMX1A)

				32	cegcervicdRFLLRisdslwheqc	Xt_ENSXETP00000028861	(LIM/homeobox protein 1.1) (LMX-1.1)
				-	....	Dr	
				-	....	Ce	
				285	cegcgqkihdrflmnvgdanwheqc	Dm_FBpp0075685	
				-	....	Sc	
