# Supplementary material for: The role of PCNA as a scaffold protein in cellular signaling is functionally conserved between yeast and humans
Source: FEBS Open Bio. 2018 May 31;8(7):1135–45. doi: 10.1002/2211-5463.12442 (PMC6026702; doi:10.1002/2211-5463.12442)
Supplement: Supplementary file 4 — Table S1. (C) Output from confind, APIM‐Sc. [file FEB4-8-1135-s004.pdf]

**The role of PCNA as a scaffold protein in cellular signaling is functionally conserved between yeast and humans**

Camilla Olaisen[1], Hans Fredrik N. Kvitvang[2], Sungmin Lee[2], Eivind Almaas[2], Per Bruheim[2], Finn Drabløs[1], and Marit Otterlei[1*].

[1]Department of Clinical and Molecular Medicine, Faculty of Medicine and Health Sciences, Norwegian University of Science and Technology (NTNU), Trondheim, Norway.

[2]Department of Biotechnology and Food Science, Faculty of Natural Sciences, Norwegian University of Science and Technology (NTNU), Trondheim, Norway.

## Supplementary Table S1 C: Output from confind, APIM-Sc

Shown below as pdf. Also found on web at

<http://tare.medisin.ntnu.no/pcna/index.php>

# confind

```
confind (c) Finn Drablos, NTNU 2007,2012
Version 02.10.2012
Program started Fri Dec 21 13:49:03 CET 2012
----------
Configuration file for APIM motif

Version FD 02-10-2012

Datasets are from Inparanoid 7.0. Gene descriptions are
taken from Inparanoid 7.0. Localisation data are from eSLDB.

Using K as equivalence for KR in consensus
Using F as equivalence for FYW in consensus
Using L as equivalence for LVI in consensus
----------
Found 5884 sequences in ./lib/S.cerevisiae.fa
Got match in 280 sequences against pattern [KR][FYW](([LVI][ALVI])|([ALVI][LVI]))[KR]
Removed 0 entries with no info in ./ref/eSLDB_Saccharomyces_cerevisiae_TS.txt matching .*
There are 280 entries left
Found 195 orthologs in Kl (Kluyveromyces lactis)
Found 183 orthologs in Ca (Candida albicans)
Found 168 orthologs in Yl (Yarrowia lipolytica)
Found 155 orthologs in Af (Aspergillus fumigatus)
Found 140 orthologs in Nc (Neurospora crassa)
Found 143 orthologs in Sp (Schizosaccharomyces pombe)
Found 123 orthologs in Cn (Cryptococcus neoformans)
Found 114 orthologs in Ro (Rhizopus oryzae)
Removed 60 entries without orthologs
There are 220 entries left
Created 69 new sequence files
Added 355 new orthologs to these sequence files
Ran ClustalW on 69 library files
Removed 136 sequences where pattern was not conserved
There are 84 entries left
Missing information on gene for 0 proteins
Removed 0 duplicate sequences representing the same gene
There are 84 entries left
Description is missing for 0 genes
----------
Pro - Protein number (green if on priority list)
# - Motif number i protein
CI - Entry is accepted according to Consensus or Individual pattern hits
Protein ID / Gene ID - IDs from input data
Pos - Position of motif in sequence
Alignment - Alignment of motif (UPPER case) and flanking region (lower case)
   lower case only - no hit by motif, "-----" - gaps, "....." - missing protein
Ortho ID - ID of Orthologs, with organism code (see above)
Description
```

| Pro | # | CI | Protein ID<br>Gene ID | Pos | Alignment | Ortho ID | Description |
|---|---|---|---|---|---|---|---|
| 1 | 1 | CI | YDR051C<br>YDR051C | 315<br>302<br>218<br>206<br>-<br>-<br>-<br>-<br>- | mvmeldesinRYVLRtvlpkwtdce<br>ivmeldekddRYVLRttlpkwc---<br>lvmkkddetqkyhlktrlltwddve<br>iimkrpehthkfellnkmetwhele<br>.........................<br>.........................<br>.........................<br>.........................<br>......................... | Sc_YDR051C<br>Kl_KLLA0F18810g<br>Ca_CAL0004865<br>Yl_YALI0F11781g<br>Af<br>Nc<br>Sp<br>Cn<br>Ro | Protein of unknown function; interacts with Hsp82p in two-hybrid assay; deletion confers sensitivity to Nickel |

| | | | | | | | |
|---|---|---|---|---|---|---|---|
| 2 | 1 | CI | YML127W<br>RSC9 | 466<br>451<br>496<br>495<br>471<br>-<br>599<br>-<br>458 | gk-------kRFVIKgiqprfkalg<br>sk-------kKFVIRgiqprrkals<br>pgeenqapkkKFIIKgiqprqfpvs<br>gt-------kKFIIKgiqprkepvd<br>at--------KFIIKgirpletayt<br>.........................<br>pv--------eyvingikrrkfpts<br>.........................<br>en----------------------- | Sc_YML127W<br>Kl_KLLA0D10461g<br>Ca_CAL0001606<br>Yl_YALI0F29997g<br>Af_AFUA_6G07810.t1<br>Nc<br>Sp_SPBC1703.02<br>Cn<br>Ro_RO3T_09289 | Component of the RSC chromatin remodeling complex; DNA-binding protein involved in the synthesis of rRNA and in transcriptional repression and activation of genes regulated by the Target of Rapamycin (TOR) pathway |
| 3 | 1 | CI | YBR118W<br>TEF2 | 421<br>421<br>421<br>422<br>456<br>422<br>421<br>421<br>421 | eafseypplgRFAVRdmrqtvavgv<br>eafsdypplgRFAVRdmrqtvavgv<br>eaftdypplgRFAVRdmrqtvavgv<br>eafteypplgRFAVRdmrqtvavgv<br>esftdypplgRFAVRdmrqtvavgv<br>eaftdypplgRFAVRdmrqtvavgv<br>eaftdyaplgRFAVRdmrqtvavgv<br>etyadypplgRFAVRdmrqtvavgv<br>eaytdypplgRFAVRdmrqtvavgv | Sc_YBR118W<br>Kl_KLLA0B08998g<br>Ca_CAL0004558<br>Yl_YALI0C09141g<br>Af_AFUA_1G06390.t1<br>Nc_NCUT_01155<br>Sp_SPBC839.15c<br>Cn_cn13119<br>Ro_RO3T_10089 | Translational elongation factor EF-1 alpha; also encoded by TEF1; functions in the binding reaction of aminoacyl-tRNA (AA-tRNA) to ribosomes |
| 4 | 1 | CI | YPR166C<br>MRP2 | 42<br>42<br>42<br>38<br>40<br>40<br>32<br>28<br>26 | keneilvkslKFIARnmnlptklrl<br>adnevtvkalKFIARnttlpprsrm<br>aehevtrnalKYIARneelpararv<br>aqqeinrralksisqnttlpmkqri<br>eqyeperqalRYIIRnttlpqrvra<br>leaeperqalRYVIRnttlpartra<br>aeheverqsnlyiyrnpelplrvrl<br>eqmevqrraflyvarnttlpatvrh<br>adneivrqafkyitrnetmparvrh | Sc_YPR166C<br>Kl_KLLA0B10648g<br>Ca_CAL0004723<br>Yl_YALI0D21626g<br>Af_AFUA_2G02270.t1<br>Nc_NCUT_00569<br>Sp_SPAC23H3.07c<br>Cn_cn02283<br>Ro_RO3T_05687 | Mitochondrial ribosomal protein of the small subunit |
| 5 | 1 | CI | YOR112W<br>CEX1 | 426<br>419<br>449<br>451<br>433<br>406<br>422<br>468<br>359 | terqlnnellRFLAKtqvdsdveir<br>terqlnndllrvlaktqvdkdigir<br>svkqvnqellrilaksqmdpkpsir<br>nsriknndllRYLAKthndkepgir<br>sdrtingellKFLARtandeqpgir<br>sdrtingdllRYLAKtandeqpgir<br>skktlnnellrslavvqndqhptlr<br>sdrilnndllrvlakmqmdtepsir<br>neriinydllKYLAKlqmdpepgir | Sc_YOR112W<br>Kl_KLLA0F23694g<br>Ca_CAL0001199<br>Yl_YALI0E27698g<br>Af_AFUA_1G09620.t1<br>Nc_NCUT_03812<br>Sp_SPAC15A10.13<br>Cn_cn08320<br>Ro_RO3T_01512 | Cytoplasmic component of the nuclear aminoacylation-dependent tRNA export pathway; interacts with nuclear pore component Nup116p; copurifies with tRNA export receptors Los1p and Msn5p, as well as eIF-1a and the RAN GTPase Gsp1p |
| 6 | 1 | C | YHR099W<br>TRA1 | 1939<br>1953<br>1929<br>1961<br>1891<br>1880<br>1884<br>1802<br>1761 | allrsshveaRYLVKqsldvltpvl<br>ailrvhqiearfmvkesldllapvv<br>allrthqtdsRYLVKqaldilapvm<br>allktsqnearqlvkqaldllapvl<br>allrahqnegkalvtqaldvlapvl<br>sllktnqnegrtlvtqalelmapvm<br>silktyqpevrafiefslasllsvl<br>gvlrlkdtdnrvlyrqaidtlassl<br>allrahssearvlvkqgldiiapvl | Sc_YHR099W<br>Kl_KLLA0F22066g<br>Ca_CAL0003086<br>Yl_YALI0C02057g<br>Af_AFUA_5G02570.t1<br>Nc_NCUT_04286<br>Sp_SPBP16F5.03c<br>Cn_cn01601<br>Ro_RO3T_02880 | Subunit of SAGA and NuA4 histone acetyltransferase complexes; interacts with acidic activators (e.g., Gal4p) which leads to transcription activation; similar to human TRRAP, which is a cofactor for c-Myc mediated oncogenic transformation |
| 6 | 2 | C | YHR099W<br>TRA1 | 2624<br>2616<br>2649<br>2668<br>2647<br>2638<br>2548<br>2465<br>2528 | s-lelpphlvkylaisynawyqsin<br>s-lelpphlvrylgitfnawyqsid<br>d-lqlppfaveclasnfdawsqgih<br>g-llmpphllkylgksfdgwfeavk<br>prfkvpphvmkylsrtydawytaas<br>pecrlpphvlkfeaktfdawytalc<br>aklelpphlvkylgklygvyhesvs<br>vpitlpptlvRFLAKnfnawyvgfe<br>pvvqlpahlvkylgkthncwhiaie | Sc_YHR099W<br>Kl_KLLA0F22066g<br>Ca_CAL0003086<br>Yl_YALI0C02057g<br>Af_AFUA_5G02570.t1<br>Nc_NCUT_04286<br>Sp_SPBP16F5.03c<br>Cn_cn01601<br>Ro_RO3T_02880 | Subunit of SAGA and NuA4 histone acetyltransferase complexes; interacts with acidic activators (e.g., Gal4p) which leads to transcription activation; similar to human TRRAP, which is a cofactor for c-Myc mediated oncogenic transformation |
| 7 | 1 | C | YPR119W<br>CLB2 | 346<br>392<br>323 | ctedeikegeKFILKtlkfnlnypn<br>cdeeeikegeKFILKtlefnlnypn<br>ytpeevvqaekymltilnfdlnypn | Sc_YPR119W<br>Kl_KLLA0D15543g<br>Ca_CAL0004596 | B-type cyclin involved in cell cycle progression; activates |

| | | | | | | | |
|---|---|---|---|---|---|---|---|
| | | | | 380<br>347<br>358<br>321<br>353<br>346 | ypedeilraerfilqvlqfnlsypn<br>ftdkeildaerhilatleynmsypn<br>fteaeilsaerfilstlnydlsypn<br>ydeeeilqaeRYILRvlefnlaypn<br>ytveellkaerymlstlqfdmsypn<br>idedelvraecfilqvldfrlcyan | Yl_YALI0B15180g<br>Af_AFUA_4G12160.t1<br>Nc_NCUT_01937<br>Sp_SPBC582.03<br>Cn_cn10336<br>Ro_RO3T_03479 | Cdc28p to promote the transition from G2 to M phase; accumulates during G2 and M, then targeted via a destruction box motif for ubiquitin-mediated degradation by the proteasome |
| 8 | 1 | C | YHL029C<br>YHL029C | 188<br>148<br>248<br>288<br>-<br>-<br>-<br>-<br>- | iietlknaivKFLLKwskifkyesg<br>----lkecvwqflrkwnkickyesg<br>---ilenaimkftlkwgkiikydss<br>rfdiyedaiesfltky--hvkyesg<br>.........................<br>.........................<br>.........................<br>.........................<br>......................... | Sc_YHL029C<br>Kl_KLLA0D03861g<br>Ca_CAL0004930<br>Yl_YALI0C00473g<br>Af<br>Nc<br>Sp<br>Cn<br>Ro | Cytoplasmic protein required for replication of Brome mosaic virus in S. cerevisiae, which is a model system for studying replication of positive-strand RNA viruses in their natural hosts |
| 9 | 1 | C | YGL131C<br>SNT2 | 249<br>266<br>298<br>192<br>353<br>449<br>213<br>0<br>128 | nifyfdelfdrytlkyykvystdki<br>ncfyfnqlfdrytlkyyqmektdqm<br>nqfyfdklfdrymikfydvlstanl<br>nhfyftqfydRYILRfyeivpthtm<br>dsfwydkmfdryihryyevvptkkv<br>dcfwfeklydryiqknyeviptkqi<br>ksyyfdrlfdqninkvfdvvpvtqv<br>-------------------------<br>dhfyynqlydryiqrvydvvpcetv | Sc_YGL131C<br>Kl_KLLA0B02178g<br>Ca_CAL0002960<br>Yl_YALI0E30547g<br>Af_AFUA_1G05240.t1<br>Nc_NCUT_07306<br>Sp_SPAC3H1.12c<br>Cn_cn04066<br>Ro_RO3T_01596 | DNA binding protein with similarity to the S. pombe Snt2 protein |
| 10 | 1 | C | YHR137W<br>ARO9 | 385<br>402<br>397<br>403<br>-<br>-<br>-<br>-<br>- | mkeamfegwirwimqiaskynhrkn<br>fngdkvrawlswvmkvageythrrn<br>-----vdgwlewilkmrlnysyrkd<br>--------wlrwclllrnqylerrn<br>.........................<br>.........................<br>.........................<br>.........................<br>......................... | Sc_YHR137W<br>Kl_KLLA0D11110g<br>Ca_CAL0002883<br>Yl_YALI0C05258g<br>Af<br>Nc<br>Sp<br>Cn<br>Ro | Aromatic aminotransferase II, catalyzes the first step of tryptophan, phenylalanine, and tyrosine catabolism |
| 11 | 1 | CI | YPL137C<br>GIP3 | 947<br>908<br>1020<br>-<br>884<br>-<br>-<br>-<br>- | stkvkkdttkRYLLKkyiekfhilh<br>saklqedstkRYLLKrywekihtih<br>kgkkedikmqriisdsvlertrsir<br>.........................<br>gddicededepapdedyviggtgvv<br>.........................<br>.........................<br>.........................<br>......................... | Sc_YPL137C<br>Kl_KLLA0D07216g<br>Ca_CAL0003186<br>Yl<br>Af_AFUA_1G12900.t1<br>Nc<br>Sp<br>Cn<br>Ro | Glc7-interacting protein whose overexpression relocalizes Glc7p from the nucleus and prevents chromosome segregation; may interact with ribosomes, based on co-purification experiments |
| 12 | 1 | CI | YOR259C<br>RPT4 | 116<br>113<br>107<br>89<br>72<br>69<br>67<br>84<br>75 | gevmkelseeKYIVKassgpryivg<br>gevmkelseeKYIVKassgpryivg<br>gevlkqlddeRYIVKtssgpryivg<br>gevlkqldeeRFIVKasngpryivg<br>gevlkqldeeRFIVKassgpryvvg<br>gevlkqlddeRFIVKassgpryvvg<br>gevlkqldseRFIVKassgpryvvg<br>gevlkqlddeRFIVKassgpryvvs<br>gevlkqlddeRFIVKntsgpryvvg | Sc_YOR259C<br>Kl_KLLA0C09592g<br>Ca_CAL0005827<br>Yl_YALI0E21868g<br>Af_AFUA_6G06780.t1<br>Nc_NCUT_07349<br>Sp_SPCC1682.16<br>Cn_cn10016<br>Ro_RO3T_05069 | One of six ATPases of the 19S regulatory particle of the 26S proteasome involved in the degradation of ubiquitinated substrates; required for spindle pole body duplication; localized mainly to the nucleus throughout the cell cycle |
| 13 | 1 | C | YER077C<br>YER077C | 631<br>663<br>900<br>-<br>-<br>-<br>-<br>-<br>- | kgte----rkKFLARrhllrlkqil<br>aedsnsllkrkflfrrhlirlrkri<br>tntsvvvstypklmeqeifryifvk<br>.........................<br>.........................<br>.........................<br>.........................<br>.........................<br>......................... | Sc_YER077C<br>Kl_KLLA0C07689g<br>Ca_CAL0000929<br>Yl<br>Af<br>Nc<br>Sp<br>Cn<br>Ro | Putative protein of unknown function; green fluorescent protein (GFP)-fusion protein localizes to the mitochondrion |

| | | | | | | | |
|---|---|---|---|---|---|---|---|
| 14 | 1 | C | YDR457W<br>TOM1 | 383<br>387<br>407<br>381<br>484<br>513<br>367<br>396<br>78 | ishgllfqilRYIAKtlrea-----<br>mshgllfqilkyisklitenn----<br>vshgilfqclrhiwkmvkdqk----<br>vshgllmhiirtlisdlkerr----<br>vnhgvlmfltrkavnelasde--rd<br>vnhgillyviriavaemkedapvdd<br>vsyglmmnlvrdfsknlenpn----<br>vnhgvlvtffrhlndrlvagea---<br>anhgiimqilrkvntneeysqs--- | Sc_YDR457W<br>Kl_KLLA0B01804g<br>Ca_CAL0001517<br>Yl_YALI0B05940g<br>Af_AFUA_4G10780.t1<br>Nc_NCUT_08465<br>Sp_SPAC19D5.04<br>Cn_cn08122<br>Ro_RO3T_06596 | E3 ubiquitin ligase of the hect-domain class; has a role in mRNA export from the nucleus and may regulate transcriptional coactivators |
| 14 | 2 | C | YDR457W<br>TOM1 | 517<br>521<br>542<br>511<br>628<br>658<br>504<br>528<br>200 | relayirsllklvlkllstd-----<br>rqiafirsllklvlkllscd-----<br>rqanyirnlmklvadliqsd-----<br>yragwlktilkfvlqvmptg-----<br>fqqqtlrwmfrfvnhimqhn----g<br>yhqqtlkwllKFIHHvmantysfdg<br>dryfllknllkfvlhliqsg-----<br>cannplktilrsiqrlmqas-----<br>drlsaikamlRFLLRmmess----- | Sc_YDR457W<br>Kl_KLLA0B01804g<br>Ca_CAL0001517<br>Yl_YALI0B05940g<br>Af_AFUA_4G10780.t1<br>Nc_NCUT_08465<br>Sp_SPAC19D5.04<br>Cn_cn08122<br>Ro_RO3T_06596 | E3 ubiquitin ligase of the hect-domain class; has a role in mRNA export from the nucleus and may regulate transcriptional coactivators |
| 15 | 1 | C | YCR033W<br>SNT1 | 484<br>525<br>473<br>867<br>-<br>-<br>79<br>720<br>452 | qkrnkiiseqKYLLKkairnfseyp<br>kpreervknqkylltkpieylfdyp<br>khhesganylkyslarpivdfnefp<br>lprservaemrylrsiplkstrdyk<br>.........................<br>.........................<br>lqekylqniyaltqnqlfknvedys<br>geeqdelgaekarelnliasvkaaq<br>eenmddwseeekwskplynsieeyt | Sc_YCR033W<br>Kl_KLLA0C03564g<br>Ca_CAL0003554<br>Yl_YALI0B20262g<br>Af<br>Nc<br>Sp_SPAC22E12.19<br>Cn_cn01044<br>Ro_RO3T_09691 | Subunit of the Set3C deacetylase complex that interacts directly with the Set3C subunit, Sif2p; putative DNA-binding protein |
| 16 | 1 | C | YOL049W<br>GSH2 | 166<br>165<br>157<br>166<br>174<br>170<br>166<br>167<br>89 | glsekvdrlhsylnra-nkydpkg-<br>glstkvgelhdylnks-gnysdng-<br>glsskigqlhqylngt-gqydskyn<br>gltskvadlhrylaat-gaygdda-<br>glsslvrklhselltsppgypisyp<br>glstqtsllhkhlaqh--eypllss<br>gvskavsnlhaycsqs-glyrkpl-<br>alsqragelhKYLAKasrnyydis-<br>slsvrtselhrfllstlkgyggnq- | Sc_YOL049W<br>Kl_KLLA0F07557g<br>Ca_CAL0002106<br>Yl_YALI0C17831g<br>Af_AFUA_5G06610.t1<br>Nc_NCUT_06031<br>Sp_SPAC3F10.04<br>Cn_cn10268<br>Ro_RO3T_00729 | Glutathione synthetase, catalyzes the ATP-dependent synthesis of glutathione (GSH) from gamma-glutamylcysteine and glycine; induced by oxidative stress and heat shock |
| 16 | 2 | C | YOL049W<br>GSH2 | 378<br>374<br>371<br>376<br>400<br>400<br>383<br>393<br>252 | rlal--sepsKYVLKpqregggnnv<br>klal--tepenyvlkpqregggnni<br>klaf--eepeKFVLKpqregggnnv<br>qlar--tepeRFVLKpqregggnni<br>elalnpetavnhvlkpqregggnnv<br>slatsptqcqkyvmkpqregggnnf<br>klaf--ekpedfvlkpqregggnnt<br>klar--qepeRFVLKpqregggnni<br>emal--ekpdelvmkpqreggghni | Sc_YOL049W<br>Kl_KLLA0F07557g<br>Ca_CAL0002106<br>Yl_YALI0C17831g<br>Af_AFUA_5G06610.t1<br>Nc_NCUT_06031<br>Sp_SPAC3F10.04<br>Cn_cn10268<br>Ro_RO3T_00729 | Glutathione synthetase, catalyzes the ATP-dependent synthesis of glutathione (GSH) from gamma-glutamylcysteine and glycine; induced by oxidative stress and heat shock |
| 17 | 1 | CI | YJL107C<br>YJL107C | 345<br>-<br>-<br>758<br>-<br>-<br>-<br>-<br>- | vhiadilqrhRFILRmcralmmyga<br>.........................<br>.........................<br>vhiaellqrqRFILRmcralmlyga<br>.........................<br>.........................<br>.........................<br>.........................<br>......................... | Sc_YJL107C<br>Kl<br>Ca<br>Yl_YALI0E24651g<br>Af<br>Nc<br>Sp<br>Cn<br>Ro | Putative protein of unknown function; expression is induced by activation of the HOG1 mitogen-activated signaling pathway and this induction is Hog1p/Pbs2p dependent; YJL107C and adjacent ORF, YJL108C are merged in related fungi |
| 18 | 1 | C | YNR019W<br>ARE2 | 546<br>499<br>513<br>447<br>482<br>478<br>441 | fsriwnipvhKFLLRhvyhssmssf<br>fsrlwnrpvhsfllrhvyhssisay<br>fanqwnrcvhKFLLRhvyhssisaf<br>fsrewnipvyqflkrhvyhssisaf<br>fsrewnipvhhflrrhvyfpslsyf<br>fardwnrpvhnfllrhvyhssisam<br>fsrewnkpvhvflmrhvyhssisgf | Sc_YNR019W<br>Kl_KLLA0C09152g<br>Ca_CAL0006272<br>Yl_YALI0F06578g<br>Af_AFUA_1G06040.t1<br>Nc_NCUT_03654<br>Sp_SPAC13G7.05 | Acyl-CoA:sterol acyltransferase, isozyme of Are1p; endoplasmic reticulum enzyme that contributes the major sterol esterification |

| | | | | | | | |
|---|---|---|---|---|---|---|---|
| | | | | 511<br>- | farkwnkpvhtfllrhvyastmttl<br>......................... | Cn_cn06362<br>Ro | activity in the presence of oxygen |
| 19 | 1 | CI | YGL163C<br>RAD54 | 549<br>544<br>496<br>454<br>454<br>461<br>504<br>473<br>446 | qlqklstivsKFIIRrtndilakyl<br>rlrqlsnivsKFIIRrtnnilakyl<br>klnelsqmvsKFIIRrtndilskyl<br>kllemasivspliirrtndilskyl<br>rlaelsgivnKFIIRrtndilskyl<br>clvellaivnKFIIRrtndilskyl<br>klaelakivnRFIIRrtndilskyl<br>klkelgglvsKFIIRrtndllskyl<br>kvaefwkivsrftirrtndilskyl | Sc_YGL163C<br>Kl_KLLA0A03069g<br>Ca_CAL0001568<br>Yl_YALI0B07513g<br>Af_AFUA_6G12910.t1<br>Nc_NCUT_03186<br>Sp_SPAC15A10.03c<br>Cn_cn04353<br>Ro_RO3T_02508 | DNA-dependent ATPase, stimulates strand exchange by modifying the topology of double-stranded DNA; involved in the recombinational repair of double-strand breaks in DNA during vegetative growth and meiosis; member of the SWI/SNF family |
| 20 | 1 | CI | YGL084C<br>GUP1 | 416<br>443<br>437<br>423<br>466<br>462<br>437<br>432<br>254 | fwrawhrsynKWVVRyiyiplggsk<br>fwrawhrsynKWVIRyiyiplggsg<br>fwrawhrsynRWVIRyiyipmggsg<br>fwrawhrsfnRWIIRyvygpiggss<br>fwrgwhrsfnRWIVRylyiplggga<br>fwrswhrsfyrwtlryiyiplgg--<br>fwrawhrsfnRWLIRyiyvplggsn<br>fwrswhrsfnlwvvryiyvpvggsk<br>----------------------gtk | Sc_YGL084C<br>Kl_KLLA0E19185g<br>Ca_CAL0000013<br>Yl_YALI0E30283g<br>Af_AFUA_2G05040.t1<br>Nc_NCUT_02107<br>Sp_SPAC24H6.01c<br>Cn_cn06354<br>Ro_RO3T_14327 | Plasma membrane protein involved in remodeling GPI anchors; member of the MBOAT family of putative membrane-bound O-acyltransferases; proposed to be involved in glycerol transport |
| 21 | 1 | C | YOR255W<br>OSW1 | 111<br>107<br>-<br>-<br>-<br>-<br>-<br>-<br>- | nlsrlklskrKYALRfyhneifgnn<br>ghlrswisgrkfsmkrivnnas---<br>.........................<br>.........................<br>.........................<br>.........................<br>.........................<br>.........................<br>......................... | Sc_YOR255W<br>Kl_KLLA0C09779g<br>Ca<br>Yl<br>Af<br>Nc<br>Sp<br>Cn<br>Ro | Protein involved in sporulation; required for the construction of the outer spore wall layers; required for proper localization of Spo14p |
| 22 | 1 | C | YOR176W<br>HEM15 | 81<br>77<br>71<br>70<br>99<br>92<br>74<br>90<br>80 | isakyqktiaKYIAKfrtpkiekqy<br>isakyqpiiaKYIARfrtpkiekqy<br>fg-rfqnilaKFIAKrrtpkieehy<br>lg-pfqgplakfiawrrtpviekhy<br>lg-rlqsylgpliakrrtpkiqrqy<br>lg-rlqnylgpliskrrtpkiqkqy<br>lgy-fqnslgKFIAKrrtpkvqnhy<br>lp--fqpllapliakrrtpsieeqy<br>fp--mqsmaakfiatrrtpqikeqy | Sc_YOR176W<br>Kl_KLLA0F20141g<br>Ca_CAL0001790<br>Yl_YALI0F19470g<br>Af_AFUA_5G07750.t1<br>Nc_NCUT_08421<br>Sp_SPCC320.09<br>Cn_cn06304<br>Ro_RO3T_05836 | Ferrochelatase, a mitochondrial inner membrane protein, catalyzes the insertion of ferrous iron into protoporphyrin IX, the eighth and final step in the heme biosynthetic pathway |
| 23 | 1 | C | YAL026C<br>DRS2 | 668<br>672<br>676<br>655<br>718<br>720<br>626<br>697<br>542 | lvqggadlgyKFIIRkgnsvtvll-<br>lvqgaadlgfrfdirrpnsvsist-<br>lvqgaadlgykftirrpkgvtien-<br>lvdgaatlgyafamrkpktigvdvk<br>lvegaaalgyrftnrrprsvlfttn<br>lvdgavqmgyRFVARkpraviiean<br>lvkgaasigyKFLARkphlvtvsif<br>lvagaemlgyrfqtrkpksvfidvn<br>lvkgasmlnykfhtrkpnsiactqr | Sc_YAL026C<br>Kl_KLLA0A04015g<br>Ca_CAL0004962<br>Yl_YALI0E34551g<br>Af_AFUA_2G08850.t1<br>Nc_NCUT_00357<br>Sp_SPBC887.12<br>Cn_cn01358<br>Ro_RO3T_04221 | Aminophospholipid translocase (flippase) that maintains membrane lipid asymmetry in post-Golgi secretory vessicles; contributes to clathrin-coated vesicle formation and endocytosis; mutations in human homolog ATP8B1 result in liver disease |
| 24 | 1 | C | YJL109C<br>UTP10 | 1534<br>1539<br>1583<br>1419<br>1551<br>1555<br>1420<br>1783<br>1869 | ieasvheisnsyvlkmndkvfrplf<br>ieasvhqianvyvlklndkvfrplf<br>lessfhgcaiafvmklndksfrplf<br>vqnlfikcliaivlklndktfrplf<br>tedalndvtikmiyklndttfrpif<br>iellinetslkmiyklndaafrpmf<br>vetqavnvflkfvmklsdttfrplf<br>veesaigsflelvtklneptfkplf<br>vegsvitafldlvmklnetlfkplf | Sc_YJL109C<br>Kl_KLLA0F21208g<br>Ca_CAL0003770<br>Yl_YALI0E29506g<br>Af_AFUA_6G13370.t1<br>Nc_NCUT_00342<br>Sp_SPBC23E6.04c<br>Cn_cn08172<br>Ro_RO3T_09841 | Nucleolar protein, component of the small subunit (SSU) processome containing the U3 snoRNA that is involved in processing of pre-18S rRNA |
| 24 | 2 | C | YJL109C<br>UTP10 | 1664<br>1669 | -------sigKYLVKaigalasnns<br>-------vigKYLVKaigslatnns | Sc_YJL109C<br>Kl_KLLA0F21208g | Nucleolar protein, component of the small subunit (SSU) |

| | | | | | | | |
|---|---|---|---|---|---|---|---|
| | | | | 1712<br>1537<br>1685<br>1686<br>1545<br>1919<br>2008 | -------sigKYLVKsvstfvtdvs<br>----vapenskllikavvalcelas<br>s---taatviaeavpaitelavaad<br>-----adnctndlvlavvelaaaad<br>-------lddkvlvkaivelasvas<br>p----spqsprpissclanlagstt<br>tsadylarmntyivpcigqmavtvs | Ca_CAL0003770<br>Yl_YALI0E29506g<br>Af_AFUA_6G13370.t1<br>Nc_NCUT_00342<br>Sp_SPBC23E6.04c<br>Cn_cn08172<br>Ro_RO3T_09841 | processome containing the U3 snoRNA that is involved in processing of pre-18S rRNA |
| 25 | 1 | C | YBR203W<br>COS111 | 534<br>381<br>387<br>408<br>-<br>-<br>-<br>-<br>- | pfsthhpyanKFLLKyapykdlplg<br>--rshlpftnkfflkyahlrdlplg<br>----shplinkfllhystskdlpig<br>-fstahpisspflrqystskdvpvg<br>.........................<br>.........................<br>.........................<br>.........................<br>......................... | Sc_YBR203W<br>Kl_KLLA0E23079g<br>Ca_CAL0005497<br>Yl_YALI0B01166g<br>Af<br>Nc<br>Sp<br>Cn<br>Ro | Protein required for resistance to the antifungal drug ciclopirox olamine; not related to the subtelomerically-encoded COS family; the authentic, non-tagged protein is detected in highly purified mitochondria in high-throughput studies |
| 26 | 1 | C | YBR073W<br>RDH54 | 534<br>540<br>431<br>555<br>518<br>-<br>-<br>546<br>- | rskemieitkRFILRrtnailekyl<br>rsdqlieitkRFILRrtneilqqyl<br>isqqlieltqsfilrrtqailanyl<br>qsarlssitgqfilrrtadilsrfl<br>rneelreltskfmlrrtvdilakyl<br>.........................<br>.........................<br>randlaklskefvlrrtaavlenyl<br>......................... | Sc_YBR073W<br>Kl_KLLA0F11814g<br>Ca_CAL0004359<br>Yl_YALI0E24431g<br>Af_AFUA_1G15120.t1<br>Nc<br>Sp<br>Cn_cn03333<br>Ro | DNA-dependent ATPase, stimulates strand exchange by modifying the topology of double-stranded DNA; involved in recombinational repair of DNA double-strand breaks during mitosis and meiosis; proposed to be involved in crossover interference |
| 27 | 1 | CI | YDR489W<br>SLD5 | 137<br>133<br>102<br>130<br>100<br>89<br>83<br>-<br>- | lcmetelerlKFVIRsyircrlski<br>lcmeaelerlKFLVRsfircrlski<br>miietdlerlnyivrlyirtrlskl<br>liveteleriKYLIRsylrvrllki<br>silqtdlsrtqflirsflrqrlakl<br>iviqtelerfKYLVRsflrtriaki<br>vlmqtelervKFVLRsymrtrinki<br>.........................<br>......................... | Sc_YDR489W<br>Kl_KLLA0F11121g<br>Ca_CAL0000861<br>Yl_YALI0B17820g<br>Af_AFUA_6G08210.t1<br>Nc_NCUT_09082<br>Sp_SPBP4H10.21c<br>Cn<br>Ro | Subunit of the GINS complex (Sld5p, Psf1p, Psf2p, Psf3p), which is localized to DNA replication origins and implicated in assembly of the DNA replication machinery |
| 28 | 1 | C | YOL008W<br>COQ10 | 45<br>46<br>26<br>34<br>79<br>-<br>14<br>86<br>- | ----htireqRYVLRkainappstv<br>----sskdeqhyvlkrnirgtpnev<br>-------kpqsyeiskilhgspkqv<br>--------pttfsvtqrfnyppgli<br>----dnnksrvltatrtlpyqpsal<br>.........................<br>-------------asrlmpykpsfl<br>qgvevdgevqryharkilpysqaql<br>......................... | Sc_YOL008W<br>Kl_KLLA0C06050g<br>Ca_CAL0004083<br>Yl_YALI0A02563g<br>Af_AFUA_6G07220.t1<br>Nc<br>Sp_SPCC16A11.07<br>Cn_cn05160<br>Ro | Coenzyme Q (ubiquinone) binding protein, functions in the delivery of Q<sub>6</sub> to its proper location for electron transport during respiration; START domain protein with homologs in bacteria and eukaryotes |
| 29 | 1 | C | YDR125C<br>ECM18 | 244<br>206<br>259<br>-<br>-<br>-<br>-<br>-<br>- | ghsfggylsfKYAVKypnsvnklcl<br>ghsyggyltfkyslkypdnvhklcl<br>ghsfggywcgsyalkypenvnnlvl<br>.........................<br>.........................<br>.........................<br>.........................<br>.........................<br>......................... | Sc_YDR125C<br>Kl_KLLA0F19624g<br>Ca_CAL0000311<br>Yl<br>Af<br>Nc<br>Sp<br>Cn<br>Ro | Protein of unknown function, similar to Rlp24p |
| 30 | 1 | C | YFL049W<br>SWP82 | 40<br>26<br>-<br>0<br>-<br>-<br>- | vpkilirehervilkqilqildqde<br>vpeyviknhelfilkttlsilnnpe<br>.........................<br>-------------------------<br>.........................<br>.........................<br>......................... | Sc_YFL049W<br>Kl_KLLA0D05885g<br>Ca<br>Yl_YALI0E06369g<br>Af<br>Nc<br>Sp | Member of the SWI/SNF chromatin remodeling complex in which it plays an as yet unidentified role; has identifiable counterparts in closely related yeast species; |

| | | | | | | | |
|---|---|---|---|---|---|---|---|
| | | | | -<br>- | .........................<br>......................... | Cn<br>Ro | abundantly expressed in many growth conditions; paralog of Npl6p |
| 31 | 1 | C | YPR018W<br>RLF2 | 583<br>562<br>559<br>500<br>628<br>669<br>531<br>797<br>- | rssgkgdlprKWVIKdaqnwenlra<br>kst-kgd--rrwevkdmnnwehlks<br>ydk-kss---kweikaemkekycs-<br>rqgpksd--kRWVLKdi--------<br>rvg-qrevdkkwvcn----------<br>vfaskhpdmvraqikasfesmtekq<br>rkgksvs--dgwiikenfasllss-<br>regrskdsvwrvyreawiaaglepa<br>......................... | Sc_YPR018W<br>Kl_KLLA0D02090g<br>Ca_CAL0004223<br>Yl_YALI0F21637g<br>Af_AFUA_5G03720.t1<br>Nc_NCUT_03891<br>Sp_SPBC29A10.03c<br>Cn_cn03648<br>Ro | Largest subunit (p90) of the Chromatin Assembly Complex (CAF-1) with Cac2p and Msi1p that assembles newly synthesized histones onto recently replicated DNA; involved in the maintenance of transcriptionally silent chromatin |
| 32 | 1 | C | YPR031W<br>NTO1 | 605<br>580<br>601<br>637<br>827<br>824<br>529<br>-<br>602 | kvfdivkspqKYLLKinvldifiks<br>iindiafhpiRYILKdvvwnnltkt<br>dvvdsvyfplkqlieltimrlnnkf<br>elirtlllpvtalclrlcdemteqd<br>sivdtvyfpifpllwpiyekaqgld<br>dfvdscyfpvyemlipalqkaiqsd<br>kflnlnyfpaqrllqdtlvkiidld<br>.........................<br>ayldlilfpldyiigpvldqise-- | Sc_YPR031W<br>Kl_KLLA0E06491g<br>Ca_CAL0003263<br>Yl_YALI0D25674g<br>Af_AFUA_7G05250.t1<br>Nc_NCUT_00042<br>Sp_SPBC17D11.04c<br>Cn<br>Ro_RO3T_03265 | Subunit of the NuA3 histone acetyltransferase complex that acetylates histone H3; contains PHD finger domain that interacts with methylated histone H3 |
| 33 | 1 | C | YLL040C<br>VPS13 | 2321<br>2291<br>2266<br>2392<br>2359<br>2401<br>2262<br>2348<br>2300 | llskvieiapryiisntldipievc<br>rftkiveispryifrnstsevieim<br>nltkvisitpryifvnkleeelqiv<br>kltrfieltprfiihnclkeelrir<br>kmtkvvtispRFILKnklseeilvr<br>kmvktvtlapryviqnrlgedinir<br>sktnivtitsRFIVRnktrwslvia<br>kltkvitiapRFLVKniysrpikir<br>klnklitltprfvlsnqmdqniryr | Sc_YLL040C<br>Kl_KLLA0E00705g<br>Ca_CAL0001755<br>Yl_YALI0F06791g<br>Af_AFUA_4G11560.t1<br>Nc_NCUT_05723<br>Sp_SPBC21C3.01c<br>Cn_cn03502<br>Ro_RO3T_13642 | Protein of unknown function; heterooligomeric or homooligomeric complex; peripherally associated with membranes; homologous to human COH1; involved in sporulation, vacuolar protein sorting and protein-Golgi retention |
| 34 | 1 | C | YLR045C<br>STU2 | 479<br>460<br>448<br>426<br>427<br>405<br>425<br>438<br>- | ktpqirmectqlfnasmk---eekd<br>kvpqvkivcskflfellsnwtptet<br>ktpqnkiasanfvkrcls------t<br>ktpqvrieslnfilrlls------t<br>knpqvkqetlKFLIRclr------t<br>knpqvkegtmKFLVRclr------t<br>knpqiksscfslfsrsfs------n<br>knpqvkegtlkflhrslq------t<br>......................... | Sc_YLR045C<br>Kl_KLLA0E16325g<br>Ca_CAL0003948<br>Yl_YALI0E28371g<br>Af_AFUA_6G12980.t1<br>Nc_NCUT_05461<br>Sp_SPCC895.07<br>Cn_cn07265<br>Ro | Microtubule-associated protein (MAP) of the XMAP215/Dis1 family; regulates microtubule dynamics during spindle orientation and metaphase chromosome alignment; interacts with spindle pole body component Spc72p |
| 35 | 1 | C | YOR126C<br>IAH1 | 57<br>59<br>63<br>53<br>65<br>53<br>82<br>51<br>35 | lqrgfkgytsRWALKilpeilkhes<br>vqrgfsgynsRWALKllpkileqeq<br>inrgfsgynseharqilpkileset<br>lvrgysgyntdqavqffhhilehek<br>inrgfggystahavkvfpkffpkpe<br>vnrglsgyntsnalkllpqifsppg<br>dvwgfsgytsrhvlrylpeipleid<br>inrgfggynsewaipvfeqvfatkk<br>vnrgfsgfntdwampilkqllptte | Sc_YOR126C<br>Kl_KLLA0E02509g<br>Ca_CAL0000954<br>Yl_YALI0B07227g<br>Af_AFUA_2G08920.t1<br>Nc_NCUT_00404<br>Sp_SPCC126.10<br>Cn_cn12157<br>Ro_RO3T_08748 | Isoamyl acetate-hydrolyzing esterase, required in balance with alcohol acetyltransferase to maintain optimal amounts of isoamyl acetate, which is particularly important in sake brewing |
| 36 | 1 | CI | YNL080C<br>EOS1 | 160<br>147<br>-<br>114<br>-<br>-<br>85<br>-<br>- | syaildslmvRWIVKystvaailrm<br>sysildslmvRWIIKyatmaaifrm<br>.........................<br>ayavldglmirwivtystpaaivrm<br>.........................<br>.........................<br>sfvlangfllkwlihysigptiirl<br>.........................<br>......................... | Sc_YNL080C<br>Kl_KLLA0E17909g<br>Ca<br>Yl_YALI0D01936g<br>Af<br>Nc<br>Sp_SPAPB17E12.08<br>Cn<br>Ro | Protein involved in N-glycosylation; deletion mutation confers sensitivity to exidative stress and shows synthetic lethality with mutations in the spindle checkpoint genes BUB3 and MAD1; YNL080C is not an essential gene |

| | | | | | | | |
|---|---|---|---|---|---|---|---|
| 37 | 1 | CI | YBR235W<br>YBR235W | 525<br>513<br>-<br>533<br>580<br>466<br>539<br>-<br>- | sqsliyhqvrKYLLRlr--qdniky<br>sqsliyhqvrKYLLRlr--qdnvky<br>.........................<br>sqallyhqvrKYLLRlr--qdhvkf<br>sqsliyhqvrKYLLRlr--qehvkf<br>sqnliyhqvrKYLLRlk--pehikf<br>sqgiiyhqlrkyllqtnkarenikf<br>.........................<br>......................... | Sc_YBR235W<br>Kl_KLLA0F10549g<br>Ca<br>Yl_YALI0B04378g<br>Af_AFUA_4G07770.t1<br>Nc_NCUT_03469<br>Sp_SPBC18H10.16<br>Cn<br>Ro | Putative ion transporter, similar to mammalian electroneutral Na(+)-(K+)-C1- cotransporter family; YBR235W is not an essential gene |
| 38 | 1 | CI | YGL099W<br>LSG1 | 485<br>494<br>504<br>554<br>505<br>485<br>449<br>548<br>465 | ygsadeprasRYILKdyvngkllyv<br>fgaadesrasRYILKdyvngkllyi<br>fgsadepraaRYILKdyvngkllyv<br>qgnpdesraaRYVLKdyvngkllyc<br>qgqpdesraaRYVLKdyvngkllfc<br>lgqpdqsravRYILKdyvngkllyc<br>hgtpddsraarillkdyvngkllyv<br>fgmpdtsraaRYVLKdyvnakllfa<br>qgnpdearaaRYILKdyvngkllfc | Sc_YGL099W<br>Kl_KLLA0F06666g<br>Ca_CAL0005034<br>Yl_YALI0C21956g<br>Af_AFUA_5G06510.t1<br>Nc_NCUT_03922<br>Sp_SPAC3F10.16c<br>Cn_cn03586<br>Ro_RO3T_09763 | Putative GTPase involved in 60S ribosomal subunit biogenesis; required for the release of Nmd3p from 60S subunits in the cytoplasm |
| 39 | 1 | CI | YKL112W<br>ABF1 | 428<br>254<br>-<br>-<br>-<br>-<br>-<br>-<br>- | iarrittykaRFVLKkkkmgeyndl<br>iarrvttykaRFVLKrkkdgvy---<br>.........................<br>.........................<br>.........................<br>.........................<br>.........................<br>.........................<br>......................... | Sc_YKL112W<br>Kl_KLLA0F02970g<br>Ca<br>Yl<br>Af<br>Nc<br>Sp<br>Cn<br>Ro | DNA binding protein with possible chromatin-reorganizing activity involved in transcriptional activation, gene silencing, and DNA replication and repair |
| 40 | 1 | CI | YDL191W<br>RPL35A | 115<br>115<br>115<br>118<br>181<br>119<br>117<br>122<br>115 | rkkqiafpqrKYAIKa---------<br>rkkqiafpqrKYAIKa---------<br>rkqriafpqrKFAIKa---------<br>tkkqthfpqrRYALKa---------<br>rkkeihfpqrKYAVKtnvvsgseme<br>kkrtthfplrKFAVKaa--------<br>ikkeryfplrKYALKa---------<br>hkknihfpkrkialka---------<br>qkkeahfplrKYAVKa--------- | Sc_YDL191W<br>Kl_KLLA0F05247g<br>Ca_CAF0007060<br>Yl_YALI0A09922g<br>Af_AFUA_1G10510.t1<br>Nc_NCUT_01988<br>Sp_SPCC613.05c<br>Cn_cn01742<br>Ro_RO3T_11228 | Protein component of the large (60S) ribosomal subunit, identical to Rpl35Bp and has similarity to rat L35 ribosomal protein |
| 41 | 1 | CI | YKL103C<br>LAP4 | 261<br>268<br>259<br>235<br>262<br>269<br>-<br>-<br>- | lfgkhcihllRYVAKlagve-vsel<br>lygkhsihllRYIAKllsvk-vsai<br>lygkhplkllRYIAKlanvs-vgdi<br>lvgkhsidllrtlskhsgva-vkdm<br>faatqperlvkiiskelgitdydti<br>fvstqppklvkliasqlkledytni<br>.........................<br>.........................<br>......................... | Sc_YKL103C<br>Kl_KLLA0F13574g<br>Ca_CAL0006379<br>Yl_YALI0C10494g<br>Af_AFUA_5G03990.t1<br>Nc_NCUT_03897<br>Sp<br>Cn<br>Ro | Vacuolar aminopeptidase, often used as a marker protein in studies of autophagy and cytosol to vacuole targeting (CVT) pathway |
| 42 | 1 | C | YKL176C<br>LST4 | 199<br>146<br>242<br>-<br>-<br>-<br>-<br>-<br>- | ----------qyn-riaislcipri<br>----------kstlriaisccvtdd<br>dtsknnnvnsrfaigivipleslgs<br>.........................<br>.........................<br>.........................<br>.........................<br>.........................<br>......................... | Sc_YKL176C<br>Kl_KLLA0A06622g<br>Ca_CAL0002848<br>Yl<br>Af<br>Nc<br>Sp<br>Cn<br>Ro | Protein possibly involved in a post-Golgi secretory pathway; required for the transport of nitrogen-regulated amino acid permease Gap1p from the Golgi to the cell surface |
| 42 | 2 | C | YKL176C<br>LST4 | 237<br>185<br>281<br>-<br>-<br>-<br>- | wltqtqkmligfltknrimqentgn<br>fldqcentllKYLAKn--------<br>ytinlqkliykkllvhlnqgltyds<br>.........................<br>.........................<br>.........................<br>......................... | Sc_YKL176C<br>Kl_KLLA0A06622g<br>Ca_CAL0002848<br>Yl<br>Af<br>Nc<br>Sp | Protein possibly involved in a post-Golgi secretory pathway; required for the transport of nitrogen-regulated amino acid permease Gap1p from the Golgi to the cell surface |

| | | | | | | | |
|---|---|---|---|---|---|---|---|
| | | | | -<br>- | .........................<br>......................... | Cn<br>Ro | |
| 43 | 1 | C | YDL169C<br>UGX2 | 219<br>180<br>-<br>-<br>-<br>-<br>-<br>-<br>- | dsdtpennhlKWLIK----------<br>esetlenkllkslvrs---------<br>.........................<br>.........................<br>.........................<br>.........................<br>.........................<br>.........................<br>......................... | Sc_YDL169C<br>Kl_KLLA0E19493g<br>Ca<br>Yl<br>Af<br>Nc<br>Sp<br>Cn<br>Ro | Protein of unknown function, transcript accumulates in response to any combination of stress conditions |
| 44 | 1 | C | YJL090C<br>DPB11 | 57<br>57<br>57<br>53<br>78<br>-<br>58<br>-<br>- | vgsttntnkfKFAVKhrfdiifidi<br>vg-elftakynfavknrtdivfvsp<br>vg-drdtekyrfcikyrpdiifida<br>vg-nqdsekylfashqrldmtflhp<br>vg-eintakyKFVARersdvvvlkp<br>.........................<br>ag-dfdtpkykfaaksrpdikimss<br>.........................<br>......................... | Sc_YJL090C<br>Kl_KLLA0F14300g<br>Ca_CAL0004555<br>Yl_YALI0E21978g<br>Af_AFUA_5G13380.t1<br>Nc<br>Sp_SPAC23C4.18c<br>Cn<br>Ro | Subunit of DNA Polymerase II Epsilon complex; has BRCT domain, required on the prereplicative complex at replication origins for loading DNA polymerases to initiate DNA synthesis, also required for S/M checkpoint control |
| 45 | 1 | C | YPR105C<br>COG4 | 390<br>347<br>402<br>324<br>457<br>482<br>353<br>452<br>155 | qilqnwsmysrfysvkwnefsdlhp<br>nflqnwsmycqffsmkwlqftngsv<br>sifenwtlycrfitvkylqepkhrq<br>milnrwalyrkflagfegg------<br>vmlsrwslycrfiaetcnvryltas<br>imlsrwsayshllaqkc--------<br>aivskwniskifisrlvlrlsqsdg<br>alggrwalfrrfiwsriadededes<br>elmnsylvideyllkks-------- | Sc_YPR105C<br>Kl_KLLA0F08184g<br>Ca_CAL0005896<br>Yl_YALI0D03113g<br>Af_AFUA_2G05890.t1<br>Nc_NCUT_02785<br>Sp_SPCC338.13<br>Cn_cn13146<br>Ro_RO3T_08984 | Essential component of the conserved oligomeric Golgi complex (Cog1p through Cog8p), a cytosolic tethering complex that functions in protein trafficking to mediate fusion of transport vesicles to Golgi compartments |
| 46 | 1 | CI | YGL137W<br>SEC27 | 62<br>62<br>62<br>62<br>62<br>62<br>63<br>64<br>64 | qvtetpvragKFIARknwiivgsdd<br>plcdapvragRFIARknwvvvgsdd<br>qvtelpvragKFIARknwivvgsdd<br>evadvplragRFIARknwiivgsdd<br>eltdvpvragRFIARknwivcgsdd<br>eltdvpvragRFIARknwiicgsdd<br>dindvpiracafiarknwfvcgsdd<br>evtdvpvrcvRYIARknwfvsgsdd<br>evsetpvraakfvprknwiitgadd | Sc_YGL137W<br>Kl_KLLA0B01958g<br>Ca_CAL0005039<br>Yl_YALI0C21802g<br>Af_AFUA_2G10610.t1<br>Nc_NCUT_07117<br>Sp_SPBC16C6.13c<br>Cn_cn02021<br>Ro_RO3T_06159 | Essential beta'-coat protein of the COPI coatomer, involved in ER-to-Golgi and Golgi-to-ER transport; contains WD40 domains that mediate cargo selective interactions; 45% sequence identity to mammalian beta'-COP |
| 47 | 1 | CI | YLR180W<br>SAM1 | 286<br>-<br>-<br>-<br>-<br>298<br>-<br>301<br>286 | kvdrsaayaaRWVAKslvaaglckr<br>.........................<br>.........................<br>.........................<br>.........................<br>kvdrsaayvgRWIAKslvaaglarr<br>.........................<br>kvdrsaaytaRWIAKslvaaglarr<br>kvdrsaaytaRWIAKslvnaklcrr | Sc_YLR180W<br>Kl<br>Ca<br>Yl<br>Af<br>Nc_NCUT_02041<br>Sp<br>Cn_cn01393<br>Ro_RO3T_03742 | S-adenosylmethionine synthetase, catalyzes transfer of the adenosyl group of ATP to the sulfur atom of methionine; one of two differentially regulated isozymes (Sam1p and Sam2p) |
| 48 | 1 | C | YBR038W<br>CHS2 | 576<br>531<br>633<br>596<br>650<br>952<br>-<br>652<br>462 | cwelvakrdaKWVLKyvkeatgetd<br>cwelvakrdeawvlkyvkeatgetd<br>cwelvskrndnwvlkfvksatgetd<br>cwelvaklnekwmlkyvpsatgetd<br>cwelvakreeRWVLKfvksavgetd<br>cwelvakrgeRWVLKyvkgctgetd<br>.........................<br>cwelvskrqckwklhyvksayaitd<br>cyelvakkeanwvlhyvsssygetd | Sc_YBR038W<br>Kl_KLLA0D03542g<br>Ca_CAL0002701<br>Yl_YALI0B16324g<br>Af_AFUA_4G04180.t1<br>Nc_NCUT_05007<br>Sp<br>Cn_cn03728<br>Ro_RO3T_16229 | Chitin synthase II, requires activation from zymogenic form in order to catalyze the transfer of N-acetylglucosamine (GlcNAc) to chitin; required for the synthesis of chitin in the primary septum during cytokinesis |
| 49 | 1 | CI | YPR080W<br>TEF1 | 421<br>-<br>421<br>- | eafseypplgRFAVRdmrqtvavgv<br>.........................<br>eaftdyypplgRFAVRdmrqtvavgv<br>......................... | Sc_YPR080W<br>Kl<br>Ca_CAL0004916<br>Yl | Translational elongation factor EF-1 alpha; also encoded by TEF2; functions in the binding |

| | | | | | | | |
|---|---|---|---|---|---|---|---|
| | | | | -<br>-<br>421<br>-<br>421 | .........................<br>.........................<br>eaftdyaplgRFAVRdmrqtvavgv<br>.........................<br>eaytdypplgRFAVRdmrqtvavgv | Af<br>Nc<br>Sp_SPAC23A1.10<br>Cn<br>Ro_RO3T_15046 | reaction of aminoacyl-tRNA (AA-tRNA) to ribosomes |
| 50 | 1 | CI | YGR240C<br>PFK1 | 876<br>854<br>897<br>-<br>691<br>-<br>-<br>-<br>- | psskdrvtasRFAVKcikfieqwnk<br>psskdrviasRFAVKcvkfieqwnk<br>psandrvmavkfslkamefietrng<br>.........................<br>pspmdriralrmavkcmlhlenyag<br>.........................<br>.........................<br>.........................<br>......................... | Sc_YGR240C<br>Kl_KLLA0A05544g<br>Ca_CAL0003055<br>Yl<br>Af_AFUA_4G00960.t1<br>Nc<br>Sp<br>Cn<br>Ro | Alpha subunit of heterooctameric phosphofructokinase involved in glycolysis, indispensable for anaerobic growth, activated by fructose-2,6-bisphosphate and AMP, mutation inhibits glucose induction of cell cycle-related genes |
| 51 | 1 | CI | YDR208W<br>MSS4 | 492<br>413<br>436<br>839<br>481<br>381<br>375<br>497<br>253 | gsffyysrdyKYIIKtihhsehihl<br>gsffyfsrdyKYIIKtihhsehkhl<br>gsffyysrdfRFIIKtihhsehkql<br>gsffyfsrdyRFIIKtihhsehkql<br>gsffyfsrdyKYIIKtihhsehkll<br>gsffyfsrdyKYIIKtihhaehkfl<br>gsffyfsrdyRFIIKtihhsehkfl<br>gsffyfsrdyRFIIKtiahsehkfl<br>gsffyysrdyRFIIKtihytehkfm | Sc_YDR208W<br>Kl_KLLA0A04301g<br>Ca_CAL0002340<br>Yl_YALI0F23573g<br>Af_AFUA_3G06080.t1<br>Nc_NCUT_03131<br>Sp_SPAC19G12.14<br>Cn_cn01363<br>Ro_RO3T_04373 | Phosphatidylinositol-4-phosphate 5-kinase, involved in actin cytoskeleton organization and cell morphogenesis; multicopy suppressor of stt4 mutation |
| 52 | 1 | C | YBR245C<br>ISW1 | 89<br>27<br>25<br>-<br>-<br>66<br>-<br>-<br>- | pphdpesnkkRYLLKd---angk-k<br>pnsiae-kkeryllplrdlasgkrk<br>----rnklsfqdnaskyftktekqq<br>.........................<br>.........................<br>pvdgrrkrseanqlrrsifgkkhdr<br>.........................<br>.........................<br>......................... | Sc_YBR245C<br>Kl_KLLA0F06710g<br>Ca_CAL0001816<br>Yl<br>Af<br>Nc_NCUT_03554<br>Sp<br>Cn<br>Ro | Member of the imitation-switch (ISWI) class of ATP-dependent chromatin remodeling complexes; ATPase that forms a complex with Ioc2p and Ioc4p to regulate transcription elongation, and a complex with Ioc3p to repress transcription initiation |
| 53 | 1 | C | YMR176W<br>ECM5 | 692<br>741<br>813<br>741<br>786<br>757<br>573<br>-<br>540 | lrfssea--akwtskmgflpgldvn<br>vnnipda--elwraenkllpsilpf<br>lnyales--eKWLAKqniipgfstf<br>ldg-paqecaslyskysqlpafsye<br>epwgamg--verlqafrrhpcfshd<br>epfghag--verlqtfrrqpcfshd<br>ikdgllnssisvlksmripssvsye<br>.........................<br>vdyglec--vkrykefrrqpcfshd | Sc_YMR176W<br>Kl_KLLA0C16203g<br>Ca_CAL0001611<br>Yl_YALI0C16390g<br>Af_AFUA_5G03430.t1<br>Nc_NCUT_04019<br>Sp_SPBP19A11.06<br>Cn<br>Ro_RO3T_13099 | Non-essential protein of unknown function, contains ATP/GTP-binding site motif A; null mutant exhibits cellular volume up to four times greater than wild-type, also large drooping buds with elongated necks |
| 54 | 1 | C | YPL106C<br>SSE1 | 623<br>623<br>626<br>619<br>-<br>-<br>632<br>626<br>615 | -degfdsikaKYIAKyeelaslgni<br>-ddgydstkgKYIAKyeelasignv<br>-edgedstkaKYIAKyeelasignv<br>-gagedakkalyiakyeelasignl<br>.........................<br>.........................<br>-eegedttkavytakledlmrvggp<br>sedgedatksayvqkldalkamgdp<br>-degydasksvyveklealkkignp | Sc_YPL106C<br>Kl_KLLA0E24597g<br>Ca_CAL0001523<br>Yl_YALI0E13255g<br>Af<br>Nc<br>Sp_SPAC110.04c<br>Cn_cn13198<br>Ro_RO3T_06487 | ATPase that is a component of the heat shock protein Hsp90 chaperone complex; binds unfolded proteins; member of the heat shock protein 70 (HSP70) family; localized to the cytoplasm |
| 54 | 2 | C | YPL106C<br>SSE1 | 641<br>641<br>644<br>637<br>-<br>-<br>650 | laslgniirgRYLAKeeekkqairs<br>lasignvirgRYLAKeeekkqalra<br>lasignvikgRYLAKeqekkeqyrq<br>lasignlikgrynakqeeerqakva<br>.........................<br>.........................<br>lmrvggpirqryldaeeakrqk--- | Sc_YPL106C<br>Kl_KLLA0E24597g<br>Ca_CAL0001523<br>Yl_YALI0E13255g<br>Af<br>Nc<br>Sp_SPAC110.04c | ATPase that is a component of the heat shock protein Hsp90 chaperone complex; binds unfolded proteins; member of the heat shock protein 70 (HSP70) family; localized to the cytoplasm |

| | | | | | | | |
|---|---|---|---|---|---|---|---|
| | | | | 644 | lkamgdpivlrwkesedrpraaaal | Cn_cn13198 | |
| | | | | 633 | lkkignpvveryreaeerphaeral | Ro_RO3T_06487 | |
| 55 | 1 | C | YNL248C RPA49 | 241 | lelfpyqnnsKYVAKkldsltqp-- | Sc_YNL248C | RNA polymerase I subunit A49 |
| | | | | 238 | leyfpyt-kfqyvanklptltqa-- | Kl_KLLA0C02959g | |
| | | | | 220 | leklpft-ksefiakqlpilisq-- | Ca_CAL0004338 | |
| | | | | 234 | vdmlpyr-tsqmvrdslykitdkt- | Yl_YALI0E05225g | |
| | | | | 290 | -aglpvattsryvsrrveavvksg- | Af_AFUA_5G06280.t1 | |
| | | | | 277 | lediqvp--sRFVAKrvsnvassd- | Nc_NCUT_06254 | |
| | | | | 257 | akllpyr-hslfinerfqrllsie- | Sp_SPAC2F3.03c | |
| | | | | 256 | asalpykrsswlqyksravanikdk | Cn_cn02440 | |
| | | | | 249 | kshlayr-dskfindrlmaiiaasg | Ro_RO3T_15617 | |
| 56 | 1 | C | YBL037W APL3 | 73 | hdrlggyqrkKYVAKlayiyitsnt | Sc_YBL037W | Alpha-adaptin, large subunit of the clathrin associated protein complex (AP-2); involved in vesicle mediated transport |
| | | | | 58 | -sglngyqrkKYVAKmayiyittna | Kl_KLLA0B12243g | |
| | | | | 56 | --nlngyqkkkyvckliyiylignp | Ca_CAL0004290 | |
| | | | | 54 | --nlsgyqkkkyvgkllyiyilg-- | Yl_YALI0C18623g | |
| | | | | 50 | --nlngyqkkkyvckllyvyiqg-- | Af_AFUA_4G04310.t1 | |
| | | | | 66 | --slsgyhkkkyvckllyiyilg-- | Nc_NCUT_02613 | |
| | | | | 52 | --tlsaydrkkyvskllyiymlg-- | Sp_SPBC691.03c | |
| | | | | 51 | --nldgyqkkKYLAKvvftyilg-- | Cn_cn07414 | |
| | | | | 0 | ------------------myilg-- | Ro_RO3T_03040 | |
| 57 | 1 | CI | YFR019W FAB1 | 2055 | gsgflktlddRFIIKelshaeleaf | Sc_YFR019W | 1-phosphatidylinositol-3-phosphate 5-kinase; vacuolar membrane kinase that generates phosphatidylinositol (3,5)P2, which is involved in vacuolar sorting and homeostasis |
| | | | | 1833 | gsaflktlddRFVIKelshseldaf | Kl_KLLA0D04598g | |
| | | | | 2402 | gssflktldnRYILKelskselesf | Ca_CAL0005404 | |
| | | | | 1818 | gsaflktlddrlvvkqlspaeldaf | Yl_YALI0E29161g | |
| | | | | 2256 | nslflktlddRFILKslspietqaf | Af_AFUA_6G07440.t1 | |
| | | | | 2296 | ksvflktlddrlvmkslspvetsaf | Nc_NCUT_01238 | |
| | | | | 1709 | gsaflktfdkKYILKvlsrlesdsl | Sp_SPBC3E7.01 | |
| | | | | 2197 | gsaflksrddRFIAKeitryemdal | Cn_cn04307 | |
| | | | | 1997 | gsaflktkddrllmkqmskyeldaf | Ro_RO3T_10566 | |
| 58 | 1 | C | YNL193W YNL193W | 455 | lvnssdnelrRYAIKeste-----k | Sc_YNL193W | Putative protein of unknown function; exhibits a two-hybrid interaction with Yhr151cp in a large-scale analysis |
| | | | | 377 | minradnelsrcllkqtelnqlqgq | Kl_KLLA0F20944g | |
| | | | | 428 | iiaradielqrssinnyss------ | Ca_CAL0001137 | |
| | | | | - | ......................... | Yl | |
| | | | | 399 | hmargdvemhrwrlglapwey---- | Af_AFUA_5G04150.t1 | |
| | | | | - | ......................... | Nc | |
| | | | | 367 | lisradleiqr-------------- | Sp_SPAC926.02 | |
| | | | | - | ......................... | Cn | |
| | | | | - | ......................... | Ro | |
| 59 | 1 | CI | YHR059W FYV4 | 85 | ekgvn-iqqrKYILKqvhnyr---- | Sc_YHR059W | Protein of unknown function, required for survival upon exposure to K1 killer toxin |
| | | | | 78 | ekgip-iqqrryilnqveklr---- | Kl_KLLA0C10164g | |
| | | | | 104 | nmgid-trarRYLLRwrhkfln--- | Ca_CAL0004161 | |
| | | | | 75 | ekgie-ardrryimtqlyryk---- | Yl_YALI0E32747g | |
| | | | | 92 | evgieparqrRYLLRkrekfrqgiy | Af_AFUA_7G03730.t1 | |
| | | | | - | ......................... | Nc | |
| | | | | 80 | kegid-vrtrkyiasqrnrfk---- | Sp_SPBC4B4.11 | |
| | | | | - | ......................... | Cn | |
| | | | | - | ......................... | Ro | |
| 60 | 1 | C | YKL028W TFA1 | 14 | piddivknllKFVVRgfyggsfvlv | Sc_YKL028W | TFIIE large subunit, involved in recruitment of RNA polymerase II to the promoter, activation of TFIIH, and promoter opening |
| | | | | 14 | pieetikallsfvirqfypnsyvlv | Kl_KLLA0E18657g | |
| | | | | 10 | -mddsvrslirfvsrgfystpyvli | Ca_CAL0005399 | |
| | | | | 9 | --mdnikrllqyvtrgfydtksilv | Yl_YALI0F26367g | |
| | | | | 9 | --mdlantlirtvvrafyetrqili | Af_AFUA_2G08450.t1 | |
| | | | | 9 | --mdiaktlircvmrafystqeili | Nc_NCUT_04339 | |
| | | | | 13 | napeivqrlikmimrafyetrhiif | Sp_SPAC458.07 | |
| | | | | 20 | eiqklcsdlvyqvaysfydvpyiii | Cn_cn03337 | |
| | | | | 0 | ------------------------- | Ro_RO3T_01562 | |
| 61 | 1 | CI | YLR106C MDN1 | 1276 | nsfatlrdlfRWALRdavgyeqlaa | Sc_YLR106C | Huge dynein-related AAA-type ATPase (midasin), forms extended pre-60S particle with the Rix1 complex (Rix1p-Ipi1p-Ipi3p), may mediate ATP- |
| | | | | 1275 | nsfatlrdlfRWALRpavgyeelaa | Kl_KLLA0F19888g | |
| | | | | 1287 | nsfatlrdlfrwamrdavgyeelaa | Ca_CAL0003858 | |
| | | | | 1177 | -afatlrdlfrwagrncdnneelam | Yl_YALI0B08338g | |
| | | | | 1268 | nsfatlrdlfRWALRraddreqlav | Af_AFUA_2G12150.t1 | |
| | | | | 1300 | nsfatlrdlfRWALRaaetrqdiad | Nc_NCUT_06238 | |

| | | | | | | | |
|---|---|---|---|---|---|---|---|
| | | | | 1094<br>1290<br>- | nsfatlrdlfrwafreavgyqqlae<br>qsfatlrdlfrwaergaigyqqlae<br>......................... | Sp_SPCC737.08<br>Cn_cn10211<br>Ro | dependent remodeling of 60S subunits and subsequent export from nucleoplasm to cytoplasm |
| 62 | 1 | CI | YBR108W<br>YBR108W | 27<br>28<br>-<br>-<br>-<br>-<br>-<br>-<br>- | sagkygyqgtKYVAKtgykaskkhy<br>sagkygyqgtKYVAKagykaskthy<br>.........................<br>.........................<br>.........................<br>.........................<br>.........................<br>.........................<br>......................... | Sc_YBR108W<br>Kl_KLLA0B09460g<br>Ca<br>Yl<br>Af<br>Nc<br>Sp<br>Cn<br>Ro | Protein interacting with Rsv167p; null mutant displays decreased frequency of mitochondrial genome loss (petite formation) and severe growth defect in minimal glycerol media |
| 63 | 1 | C | YJL012C<br>VTC4 | 296<br>297<br>304<br>295<br>308<br>338<br>294<br>342<br>891 | dwtgeksvkaRFALKerhvndflkg<br>dwtgeksvkaRFALKekyvndflhg<br>dwtgeksvkaRFALKekkvnqflsg<br>dwtgeksvkarfnlkekhvndfmag<br>dwtgeksvkarfslkekhvnaylsg<br>dwtgeksvkaRFALKeklvnpylrg<br>dwtgeksvkarfplkekyvnaflrg<br>dwtgeksvkerftikegkmndfisg<br>swtgeksvkarfpikekylnaflkg | Sc_YJL012C<br>Kl_KLLA0F22627g<br>Ca_CAL0004171<br>Yl_YALI0C10945g<br>Af_AFUA_2G09040.t1<br>Nc_NCUT_09629<br>Sp_SPCC1322.14c<br>Cn_cn04250<br>Ro_RO3T_16785 | Vacuolar membrane protein involved in vacuolar polyphosphate accumulation; functions as a regulator of vacuolar H+-ATPase activity and vacuolar transporter chaperones; involved in non-autophagic vacuolar fusion |
| 64 | 1 | C | YOL129W<br>VPS68 | 136<br>128<br>134<br>136<br>126<br>132<br>123<br>141<br>129 | lsgsivvliiKFLVKdyntyptlgm<br>isgslvvlivKFLLKdytsyptlgm<br>lagsfmvfilkylmnhy-tfptlgm<br>massiavlvlkyalpgy-smptlgm<br>lagsvtvmvlKYLIKqy-plptlyf<br>maggvtvfvlKYVVKdv-gfpalrm<br>lggsftvfilkyvvagy-egksllm<br>lagslcvlilkyivpdytg--ytyy<br>lagsccvlivkyiihldssqpyiny | Sc_YOL129W<br>Kl_KLLA0E07415g<br>Ca_CAL0005014<br>Yl_YALI0D20944g<br>Af_AFUA_6G04760.t1<br>Nc_NCUT_03901<br>Sp_SPBC8D2.02c<br>Cn_cn02565<br>Ro_RO3T_00165 | Vacuolar membrane protein of unknown function involved in vacuolar protein sorting; also detected in the mitochondria |
| 65 | 1 | CI | YMR109W<br>MYO5 | 520<br>518<br>532<br>521<br>553<br>520<br>525<br>526<br>518 | tnphfdlrsnKFVIKhyagdvtydi<br>tnahfdlrssKFVIKhyagdvtydi<br>snrhfedrrgKFIIKhyagdvtydv<br>qnphfeqrqrKFVIKhyagdvtydv<br>-npnfenrqgqfiikhyagdvsyav<br>-nphltprqgafivkhyagdvtysv<br>-nphfeqrqnqfivkhyagdvtysi<br>-npnfeargnKFLIKhyagdvlytv<br>-nphfesrgsKFLIRhyagdvlyni | Sc_YMR109W<br>Kl_KLLA0B12562g<br>Ca_CAL0001808<br>Yl_YALI0E02046g<br>Af_AFUA_8G05660.t1<br>Nc_NCUT_01269<br>Sp_SPBC146.13c<br>Cn_cn09230<br>Ro_RO3T_15775 | One of two type I myosins; contains proline-rich tail homology 2 (TH2) and SH3 domains; MYO5 deletion has little effect on growth, but myo3 myo5 double deletion causes severe defects in growth and actin cytoskeleton organization |
| 66 | 1 | CI | YML098W<br>TAF13 | 69<br>69<br>83<br>73<br>-<br>75<br>74<br>-<br>78 | qrnklrledfKFALRkdpiklgrae<br>krnkikvedfRFVLRkdevklgrae<br>nrtrvklndlaftlrndpmklarfr<br>grqkikvddfKFLLRndprklgrae<br>.........................<br>grqkvkfedfefamrrnprfmgkiq<br>-rnkvkvddfKFALRddpkklgrve<br>.........................<br>-rgkvkvedfKFVLRkdtkklarve | Sc_YML098W<br>Kl_KLLA0D10219g<br>Ca_CAL0002660<br>Yl_YALI0D09625g<br>Af<br>Nc_NCUT_02632<br>Sp_SPCC1494.02c<br>Cn<br>Ro_RO3T_03275 | TFIID subunit (19 kDa), involved in RNA polymerase II transcription initiation, similar to histone H4 with atypical histone fold motif of Spt3-like transcription factors |
| 67 | 1 | CI | YDL164C<br>CDC9 | 300<br>251<br>314<br>285<br>434<br>433<br>300<br>337<br>243 | -------ieaKFLIRsleskrigl<br>-------yeaKFLIRsleskrigl<br>-------seaKFLIRslqgklrigs<br>-------neaKFIIRslegklrigl<br>--------eaKFIVRflegklrlgl<br>tkdkggpseaKFIVRflegklrlgl<br>--------epKYLIRalegklrlql<br>--------eaKFIIRslegklrigl<br>--------eaKYIIRqlegklrigl | Sc_YDL164C<br>Kl_KLLA0D12496g<br>Ca_CAL0006047<br>Yl_YALI0F01034g<br>Af_AFUA_2G09010.t1<br>Nc_NCUT_06250<br>Sp_SPAC20G8.01<br>Cn_cn08403<br>Ro_RO3T_07347 | DNA ligase found in the nucleus and mitochondria, an essential enzyme that joins Okazaki fragments during DNA replication; also acts in nucleotide excision repair, base excision repair, and recombination |

| | | | | | | | |
|---|---|---|---|---|---|---|---|
| 68 | 1 | C | YLR382C<br>NAM2 | 569<br>556<br>521<br>503<br>613<br>613<br>555<br>-<br>492 | ehailhllysRFIAKflgsinawsd<br>ehailhllysrfiskftasigmwdg<br>ehailhllysRFIAKflsdcglwdg<br>ehailhllyaRFVAKfladkgywsg<br>ehailhllyarfiykflsqtelfpe<br>ehailhllysrfiykflmtssfagk<br>ehsilhllysrffskfmkdiglwng<br>.........................<br>ehailhllysrfiskvllkqgayqq | Sc_YLR382C<br>Kl_KLLA0E05325g<br>Ca_CAL0001358<br>Yl_YALI0F02299g<br>Af_AFUA_6G04910.t1<br>Nc_NCUT_03498<br>Sp_SPAC4G8.09<br>Cn<br>Ro_RO3T_15723 | Mitochondrial leucyl-tRNA synthetase, also has a direct role in splicing of several mitochondrial group I introns; indirectly required for mitochondrial genome maintenance |
| 68 | 2 | C | YLR382C<br>NAM2 | 695<br>678<br>643<br>624<br>741<br>746<br>677<br>-<br>596 | wdeskivgierwlqkvlhltknils<br>wdeskivgierwlermlkfc---is<br>wqeeqisgtdrwlrrviqlgdsite<br>wdegkivgikRWLLKvksitesvvk<br>wddtkivgierwfgrlwklvldakq<br>wdeskitgvtrwlsrvhdlvqkiac<br>wnenaimgtkrwltkiwncvhqlle<br>.........................<br>wddtsivgmqRWLAKvlkls----- | Sc_YLR382C<br>Kl_KLLA0E05325g<br>Ca_CAL0001358<br>Yl_YALI0F02299g<br>Af_AFUA_6G04910.t1<br>Nc_NCUT_03498<br>Sp_SPAC4G8.09<br>Cn<br>Ro_RO3T_15723 | Mitochondrial leucyl-tRNA synthetase, also has a direct role in splicing of several mitochondrial group I introns; indirectly required for mitochondrial genome maintenance |
| 69 | 1 | I | YLR256W<br>HAP1 | 622<br>503<br>477<br>416<br>-<br>-<br>-<br>-<br>- | tpvealelcqKYLIKfdelssisnn<br>tpietveivrKFLIKfdeissfans<br>inidvvsvaqecfnqfnimr-----<br>awtewlkgidhcleqamvcvr----<br>.........................<br>.........................<br>.........................<br>.........................<br>......................... | Sc_YLR256W<br>Kl_KLLA0F22990g<br>Ca_CAL0002550<br>Yl_YALI0C18667g<br>Af<br>Nc<br>Sp<br>Cn<br>Ro | Zinc finger transcription factor involved in the complex regulation of gene expression in response to levels of heme and oxygen; the S288C sequence differs from other strain backgrounds due to a Ty1 insertion in the carboxy terminus |
| 70 | 1 | CI | YLR089C<br>ALT1 | 124<br>62<br>48<br>40<br>38<br>22<br>-<br>48<br>- | edvnenvlkaKYAVRgaipmraeel<br>ddvnenvlkaKYAVRgripmraeel<br>hdinpqtveaKYAVRgkipiiadel<br>kdlnpqvvnaqyavrgklavradei<br>dninpnvkaaKYAVRgelavkaeey<br>dninphvraaKYAVRgelavkseef<br>.........................<br>dtinpavqavhyavrgelaikadky<br>......................... | Sc_YLR089C<br>Kl_KLLA0F19162g<br>Ca_CAL0004796<br>Yl_YALI0D06325g<br>Af_AFUA_6G07770.t1<br>Nc_NCUT_03635<br>Sp<br>Cn_cn07331<br>Ro | Putative alanine transaminase (glutamic pyruvic transaminase); the authentic, non-tagged protein is detected in highly purified mitochondria in high-throughput studies |
| 71 | 1 | C | YKR026C<br>GCN3 | 75<br>75<br>75<br>75<br>86<br>97<br>88<br>98<br>60 | slragcdifmRFVLRnlhl------<br>slragcdifmRFVLKnthl------<br>slsagcdlfmRFVLRntnv------<br>slsagcdlfmRFVLRnire------<br>glsagtdlfqrylittlqrpgqlgp<br>pllagadlfeqyllrslrg----qt<br>slsagcdifqrfvtrslhd------<br>gvragcqlwerffalfpgv------<br>------------------------- | Sc_YKR026C<br>Kl_KLLA0F13970g<br>Ca_CAL0000120<br>Yl_YALI0C18161g<br>Af_AFUA_5G11340.t1<br>Nc_NCUT_05286<br>Sp_SPCC11E10.07c<br>Cn_cn03560<br>Ro_RO3T_06935 | Alpha subunit of the translation initiation factor eIF2B, the guanine-nucleotide exchange factor for eIF2; activity subsequently regulated by phosphorylated eIF2; first identified as a positive regulator of GCN4 expression |
| 72 | 1 | C | YHL030W<br>ECM29 | 363<br>352<br>384<br>316<br>361<br>359<br>301<br>-<br>243 | klrsltlsfirhvaklnyknlnpas<br>rvklaalvfirhvaknnhlalrett<br>rlrslaiqfirklgqspvpeenlek<br>ysraaiiqfvnwivkhsneelflrh<br>klrtqiftfttwvvrmgsp-sdlkl<br>kvhralfefinwvarigaskpefsk<br>smqprliqftrwvvdkadpnflkpk<br>.........................<br>slspvapiivsgllkfinqleeaqg | Sc_YHL030W<br>Kl_KLLA0E00727g<br>Ca_CAL0004949<br>Yl_YALI0C03828g<br>Af_AFUA_6G06540.t1<br>Nc_NCUT_00776<br>Sp_SPAC1782.01<br>Cn<br>Ro_RO3T_02505 | Major component of the proteasome; tethers the proteasome core particle to the regulatory particle, and enhances the stability of the proteasome |
| 73 | 1 | CI | YDR502C<br>SAM2 | 288<br>288<br>289<br>290<br>291<br>- | kvdrsaayaaRWVAKslvaaglckr<br>kvdrsaayaaRWVAKslvhagickr<br>kvdrsaayaaRWVAKslvtaglakr<br>kvdrsaayaaRWVAKslvkaglarr<br>kvdrsaayvgRWIAKslvhaglarr<br>......................... | Sc_YDR502C<br>Kl_KLLA0C01782g<br>Ca_CAL0001034<br>Yl_YALI0B14509g<br>Af_AFUA_1G10630.t1<br>Nc | S-adenosylmethionine synthetase, catalyzes transfer of the adenosyl group of ATP to the sulfur atom of methionine; one of two differentially |

| | | | | | | | |
|---|---|---|---|---|---|---|---|
| | | | | 286<br>-<br>- | kvdrsaayaaRWIAKslvaaglarr<br>.........................<br>......................... | Sp_SPBC14F5.05c<br>Cn<br>Ro | regulated isozymes (Sam1p and Sam2p) |
| 74 | 1 | CI | YGR124W<br>ASN2 | 450<br>-<br>450<br>443<br>498<br>460<br>437<br>-<br>445 | mikpkegrieKYILRkafdttdepd<br>.........................<br>liqp--gkieKYILRkafdtsdepd<br>iidk--dhiekysirkafdtsdepg<br>mitk--erieKYILRkafdtsdepd<br>mitk--ekleKYILRkafdtkdqpe<br>my--ingrkeKYILRkafdtthdss<br>.........................<br>lhdrsqgrmeKYILRkafddes--- | Sc_YGR124W<br>Kl<br>Ca_CAL0003290<br>Yl_YALI0A13387g<br>Af_AFUA_4G06900.t1<br>Nc_NCUT_03787<br>Sp_SPBC119.10<br>Cn<br>Ro_RO3T_15933 | Asparagine synthetase, isozyme of Asn1p; catalyzes the synthesis of L-asparagine from L-aspartate in the asparagine biosynthetic pathway |
| 75 | 1 | C | YHR116W<br>COX23 | 91<br>62<br>74<br>60<br>-<br>-<br>-<br>36<br>- | ddpenpvnkyKFALKadsqyydpce<br>ddpestlnryRFAVKgaseyydpcq<br>dnpkshrhkykwsmkepskfydpce<br>ddptamsnrvqflakadsqyydpca<br>.........................<br>.........................<br>.........................<br>eipedykktfr-grgtvskfvdpce<br>......................... | Sc_YHR116W<br>Kl_KLLA0F24024g<br>Ca_CAL0003316<br>Yl_YALI0B10274g<br>Af<br>Nc<br>Sp<br>Cn_cn14137<br>Ro | Mitochondrial intermembrane space protein that functions in mitochondrial copper homeostasis, essential for functional cytochrome oxidase expression; homologous to Cox17p |
| 76 | 1 | C | YPL125W<br>KAP120 | 763<br>762<br>745<br>745<br>775<br>1398<br>750<br>767<br>- | tfqdifkqmsKYLLKlredsfqlvl<br>afteilkhaasyllllrddsytill<br>laseifqvignylskmrddayaifi<br>-----htllsifgkymdilsfdvvq<br>rlpllvsfetllqsttrqrigvvpr<br>rrptlaalvktldarsreqsqtgar<br>-----ftifeklndllddvknetlq<br>---qaiasnlamalststgnesavi<br>......................... | Sc_YPL125W<br>Kl_KLLA0D06633g<br>Ca_CAL0003089<br>Yl_YALI0B00198g<br>Af_AFUA_1G03590.t1<br>Nc_NCUT_01223<br>Sp_SPCC1322.06<br>Cn_cn12143<br>Ro | Karyopherin with a role in the assembly or export of 60S ribosomal subunits |
| 77 | 1 | C | YOR260W<br>GCD1 | 406<br>338<br>306<br>312<br>428<br>418<br>344<br>407<br>295 | nnlnaymdanRFVLKiksqtmftkn<br>nslsaymesnRYVLKik-qtaiqqr<br>nnlpvlmeanryfmkkqaiaksasq<br>nnlsaymemnrvilkarakanataq<br>dtsalllsvslrlaklesieevg-r<br>dtaqlllqislqlaklpsieevgsa<br>nnlpnyfelnkciak---------<br>nslagywelnrrfikslssttpaak<br>ntiasyselnryvtk---------q | Sc_YOR260W<br>Kl_KLLA0C09570g<br>Ca_CAL0005825<br>Yl_YALI0E21846g<br>Af_AFUA_1G16660.t1<br>Nc_NCUT_02713<br>Sp_SPAC4D7.09<br>Cn_cn08385<br>Ro_RO3T_10989 | Gamma subunit of the translation initiation factor eIF2B, the guanine-nucleotide exchange factor for eIF2; activity subsequently regulated by phosphorylated eIF2; first identified as a negative regulator of GCN4 expression |
| 78 | 1 | CI | YDL136W<br>RPL35B | 115<br>-<br>-<br>-<br>-<br>-<br>-<br>-<br>115 | rkkqiafpqrKYAIKa---------<br>.........................<br>.........................<br>.........................<br>.........................<br>.........................<br>.........................<br>.........................<br>qkkeahfplrKYAVKa--------- | Sc_YDL136W<br>Kl<br>Ca<br>Yl<br>Af<br>Nc<br>Sp<br>Cn<br>Ro_RO3T_13637 | Protein component of the large (60S) ribosomal subunit, identical to Rpl35Ap and has similarity to rat L35 ribosomal protein |
| 79 | 1 | CI | YJL187C<br>SWE1 | 469<br>421<br>-<br>447<br>-<br>754<br>592<br>820<br>- | ---------kKYAIKaikp--nkyn<br>---------lKYAIKsvrp--tkhn<br>.........................<br>gssviltppnRYAVKkmkypfagpk<br>.........................<br>fssftstp-grhtpevekvfavkki<br>---------lKYAVKklkvkfsgpk<br>---------glwavkkargmfdgar<br>......................... | Sc_YJL187C<br>Kl_KLLA0B07579g<br>Ca<br>Yl_YALI0F01716g<br>Af<br>Nc_NCUT_03764<br>Sp_SPCC18B5.03<br>Cn_cn07077<br>Ro | Protein kinase that regulates the G2/M transition by inhibition of Cdc28p kinase activity; localizes to the nucleus and to the daughter side of the mother-bud neck; homolog of S. pombe Wee1p; potential Cdc28p substrate |
| 80 | 1 | C | YDR421W<br>ARO80 | 698<br>684<br>842<br>773 | rqgmlkympvRWVIRiirsiafivk<br>klkmlkympvrwvmrivrsivfmik<br>rfkmlrfmpvrlltrfiraaafivr<br>rmealkyapvRWVVRivhatvflfk | Sc_YDR421W<br>Kl_KLLA0A01804g<br>Ca_CAL0001342<br>Yl_YALI0C18645g | Zinc finger transcriptional activator of the Zn2Cys6 family; activates transcription |

| | | | | | | | |
|---|---|---|---|---|---|---|---|
| | | | | 758<br>817<br>-<br>749<br>- | pgdhlkhapvrtcfrilsgmifilk<br>pkgylkhapvrtyfriisgamfllk<br>.........................<br>qmgslrylpsrylinisyaavfalk<br>......................... | Af_AFUA_5G13310.t1<br>Nc_NCUT_00452<br>Sp<br>Cn_cn08195<br>Ro | of aromatic amino acid catabolic genes in the presence of aromatic amino acids |
| 81 | 1 | CI | YMR162C<br>DNF3 | 1266<br>1185<br>1332<br>619<br>1163<br>1100<br>1280<br>-<br>- | rvsdysigqfRFLLKllfvhgryny<br>rssdysiaqfRYLLKllfvhgryny<br>rsadyaiaqfRFLLKlllvngryny<br>rssdfaiaqfsyllklllvhghwny<br>risdysiaqfRFLLKlllvhgrwny<br>risdfsisqfrflqrllfvhgrwny<br>rssdfsigrfKFLIKllfchgrwsy<br>.........................<br>......................... | Sc_YMR162C<br>Kl_KLLA0E01651g<br>Ca_CAL0003528<br>Yl_YALI0E04169g<br>Af_AFUA_4G10210.t1<br>Nc_NCUT_03292<br>Sp_SPAC821.13c<br>Cn<br>Ro | Aminophospholipid translocase (flippase) that maintains membrane lipid asymmetry in post-Golgi secretory vessicles; localizes to the trans-Golgi network; likely involved in protein transport; type 4 P-type ATPase |
| 82 | 1 | CI | YDL140C<br>RPO21 | 403<br>403<br>403<br>408<br>418<br>417<br>409<br>419<br>- | rngpnehpgaKYVIRdsgdridlry<br>rngpnehpgaKYVIResgdridlry<br>rngpnehpgaKYVIRdtgdridlry<br>rngpnehpgaKYVIRdtgeridlry<br>angpnehpgaRYIVRdngeridlrh<br>qngpnehpgaKYVIRsdgsrvdlrh<br>rngpdehpgaKYIIRdtgeridlry<br>nngpatypgaryyvkdtgervdlky<br>......................... | Sc_YDL140C<br>Kl_KLLA0F05071g<br>Ca_CAL0000919<br>Yl_YALI0C16566g<br>Af_AFUA_1G14680.t1<br>Nc_NCUT_01261<br>Sp_SPBC28F2.12<br>Cn_cn05376<br>Ro | RNA polymerase II largest subunit B220, part of central core; phosphorylation of C-terminal heptapeptide repeat domain regulates association with transcription and splicing factors; similar to bacterial beta-prime |
| 83 | 1 | CI | YPR145W<br>ASN1 | 451<br>451<br>-<br>-<br>-<br>-<br>-<br>467<br>- | --kegr--ieKYILRkafdttgepd<br>--aegk--ieKYILRkafdttdepd<br>.........................<br>.........................<br>.........................<br>.........................<br>.........................<br>vdedgrpkmeKYILRkafdcs--pe<br>......................... | Sc_YPR145W<br>Kl_KLLA0B10868g<br>Ca<br>Yl<br>Af<br>Nc<br>Sp<br>Cn_cn12090<br>Ro | Asparagine synthetase, isozyme of Asn2p; catalyzes the synthesis of L-asparagine from L-aspartate in the asparagine biosynthetic pathway |
| 84 | 1 | C | YBL004W<br>UTP20 | 2062<br>2063<br>2075<br>2101<br>2129<br>2162<br>2067<br>2175<br>2288 | qskgrlekqfkfmvdnlqyptesgr<br>qskgrlekqfrfmlnnlqypsqegr<br>qgkgrlekqfkylvsnltypteegr<br>qsrdrlnaefqflihnldypfdsgr<br>qaknrwakqlsflaknldykhqegr<br>qkrnrwekqlkfivanlkyereggr<br>qgktrlskqisfilknleyefapgr<br>qgkgrlksqmtffaqnlqytyeagr<br>qgkgrlkkqmsfiiknlefefesgr | Sc_YBL004W<br>Kl_KLLA0E17425g<br>Ca_CAL0002346<br>Yl_YALI0D22440g<br>Af_AFUA_6G08240.t1<br>Nc_NCUT_02205<br>Sp_SPBC56F2.04<br>Cn_cn12221<br>Ro_RO3T_14432 | Component of the small-subunit (SSU) processome, which is involved in the biogenesis of the 18S rRNA |
