# Supplementary material for: The role of PCNA as a scaffold protein in cellular signaling is functionally conserved between yeast and humans
Source: FEBS Open Bio. 2018 May 31;8(7):1135–45. doi: 10.1002/2211-5463.12442 (PMC6026702; doi:10.1002/2211-5463.12442)
Supplement: Supplementary file 5 — Table S2. Annotation clusters from enrichment analysis with DAVID. [file FEB4-8-1135-s005.pdf]

Supplementary Table S2. Annotation clusters from enrichment analysis with DAVID

Individual enrichment terms with p-value <=0.05 are marked with pink background.

Annotation Cluster 1	Enrichment Score: 3.224											
Category	Term	Count	%	PValue	Genes	List Total	Pop Hits	Pop Total	Fold Enrichment	Bonferroni	Benjamini	FDR
INTERPRO	IPR011989:Armadillo-like helical	8	10	3.23E-05	YJL109C, YHL030W, YOR112W, YHR099W, YLR045C, YBL037W, YBL004W, YPL125W	72	66	5053	8.50673401	0.006048	0.006048	0.04003
INTERPRO	IPR016024:Armadillo-type fold	9	11	1.33E-04	YJL109C, YHL030W, YOR112W, YHR099W, YLR045C, YBL037W, YDR457W, YBL004W, YPL125W	72	110	5053	5.74204545	0.024692	0.012423	0.16486
UP_SEQ_FEATURE	repeat:HEAT 1	4	5	0.005434	YHL030W, YOR112W, YLR045C, YBL004W	80	27	5872	10.8740741	0.748043	0.748043	6.84593
UP_SEQ_FEATURE	repeat:HEAT 2	4	5	0.005434	YHL030W, YOR112W, YLR045C, YBL004W	80	27	5872	10.8740741	0.748043	0.748043	6.84593

Annotation Cluster 2	Enrichment Score: 2.554											
Category	Term	Count	%	PValue	Genes	List Total	Pop Hits	Pop Total	Fold Enrichment	Bonferroni	Benjamini	FDR
UP_KEYWORDS	ATP-binding	20	25	7.15E-04	YLR106C, YPL106C, YLR382C, YGR240C, YMR162C, YBR245C, YGR124W, YGL163C, YBR073W, YLR180W, YOR259C, YDR502C, YDR208W, YMR109W, YOL049W, YDL164C, YPR145W, YAL026C, YJL187C, YFR019W	80	651	5914	2.27112135	0.081541	0.081541	0.81592
GOTERM_MF_DIRECT	GO:0005524~ATP binding	20	25	7.93E-04	YLR106C, YPL106C, YLR382C, YGR240C, YMR162C, YBR245C, YGR124W, YGL163C, YBR073W, YLR180W, YOR259C, YDR502C, YDR208W, YMR109W, YOL049W, YDL164C, YPR145W, YAL026C, YJL187C, YFR019W	66	668	4873	2.21057884	0.099453	0.099453	0.9224
UP_KEYWORDS	Nucleotide-binding	21	26	0.001851	YGL099W, YLR106C, YPL106C, YLR382C, YGR240C, YMR162C, YBR245C, YGR124W, YGL163C, YBR073W, YLR180W, YOR259C, YDR502C, YDR208W, YMR109W, YOL049W, YDL164C, YPR145W, YAL026C, YJL187C, YFR019W	80	760	5914	2.04266447	0.19787	0.070859	2.10128
GOTERM_MF_DIRECT	GO:0000166~nucleotide binding	21	26	0.004631	YGL099W, YLR106C, YPL106C, YLR382C, YGR240C, YMR162C, YBR245C, YGR124W, YGL163C, YBR073W, YLR180W, YOR259C, YDR502C, YDR208W, YMR109W, YOL049W, YDL164C, YPR145W, YAL026C, YJL187C, YFR019W	66	834	4873	1.85911271	0.458147	0.263894	5.27638
UP_SEQ_FEATURE	nucleotide phosphate-binding region:ATP	11	14	0.035142	YDR502C, YOR259C, YMR109W, YOL049W, YLR106C, YGR240C, YBR245C, YJL187C, YGL163C, YBR073W, YLR180W	80	390	5872	2.07025641	0.999883	0.951051	37.2252

Annotation Cluster 3	Enrichment Score: 1.680											
Category	Term	Count	%	PValue	Genes	List Total	Pop Hits	Pop Total	Fold Enrichment	Bonferroni	Benjamini	FDR

UP_KEYWORDS	Ligase	7	8.8	0.006005	YOL049W, YDL164C, YPR145W, YLR382C, YDR457W, YGR124W, YGL131C	80	124	5914	4.17318548	0.511681	0.164059	6.67107
GOTERM_MF_DIRECT	GO:0016874~ligase activity	7	8.8	0.006058	YOL049W, YDL164C, YPR145W, YLR382C, YDR457W, YGR124W, YGL131C	66	125	4873	4.13466667	0.551599	0.234599	6.84947
GOTERM_MF_DIRECT	GO:0042803~protein homodimerization activity	3	3.8	0.046366	YOL049W, YPR145W, YGR124W	66	26	4873	8.51923077	0.998102	0.591498	42.5573
INTERPRO	IPR014729:Rossmann-like alpha/beta/alpha sandwich fold	3	3.8	0.112528	YPR145W, YLR382C, YGR124W	72	41	5053	5.1351626	1	0.870009	77.2617

Annotation Cluster 4	Enrichment Score: 1.636											
Category	Term	Count	%	PValue	Genes	List Total	Pop Hits	Pop Total	Fold Enrichment	Bonferroni	Benjamini	FDR
INTERPRO	IPR017884:SANT domain	3	3.8	0.003906	YCR033W, YBR245C, YGL131C	72	7	5053	30.077381	0.520865	0.168017	4.73967
INTERPRO	IPR001005:SANT/Myb domain	3	3.8	0.02576	YCR033W, YBR245C, YGL131C	72	18	5053	11.6967593	0.992601	0.420247	27.6599
SMART	SM00717:SANT	3	3.8	0.031389	YCR033W, YBR245C, YGL131C	29	18	1776	10.2068966	0.615862	0.380212	23.7893
INTERPRO	IPR009057:Homeodomain-like	3	3.8	0.090344	YCR033W, YBR245C, YGL131C	72	36	5053	5.84837963	1	0.831388	69.1119

Annotation Cluster 5	Enrichment Score: 1.617											
Category	Term	Count	%	PValue	Genes	List Total	Pop Hits	Pop Total	Fold Enrichment	Bonferroni	Benjamini	FDR
GOTERM_BP_DIRECT	GO:0006351~transcription, DNA-templated	15	19	0.008387	YDL140C, YPR031W, YDR421W, YML098W, YLR256W, YML127W, YBR245C, YJL109C, YHR099W, YNL248C, YFL049W, YKL112W, YDR457W, YKL028W, YGL131C	74	528	5514	2.11686118	0.878231	0.878231	10.3643
UP_KEYWORDS	Transcription	15	19	0.009335	YDL140C, YPR031W, YDR421W, YML098W, YLR256W, YML127W, YBR245C, YJL109C, YHR099W, YNL248C, YFL049W, YKL112W, YDR457W, YKL028W, YGL131C	80	528	5914	2.10014205	0.672436	0.169736	10.1922
GOTERM_MF_DIRECT	GO:0003677~DNA binding	14	18	0.011087	YCR033W, YDL140C, YDR421W, YML098W, YLR256W, YML127W, YBR245C, YGL163C, YBR073W, YMR176W, YNL248C, YDL164C, YKL112W, YGL131C	66	489	4873	2.11383776	0.770469	0.254979	12.2074
GOTERM_BP_DIRECT	GO:0006355~regulation of transcription, DNA-templated	13	16	0.033718	YCR033W, YPR031W, YHR099W, YDR421W, YML098W, YLR256W, YFL049W, YKL112W, YML127W, YBR245C, YDR457W, YKL028W, YGL131C	74	509	5514	1.90309563	0.999811	0.986261	35.9553
UP_KEYWORDS	Transcription regulation	12	15	0.050922	YPR031W, YHR099W, YDR421W, YML098W, YLR256W, YFL049W, YKL112W, YML127W, YBR245C, YDR457W, YKL028W, YGL131C	80	477	5914	1.85974843	0.99801	0.540418	45.067

UP_KEYWORDS	Nucleus	29	36	0.13254	YML098W, YJL090C, YML127W, YBR073W, YJL109C, YMR176W, YNL248C, YDL164C, YKL028W, YCR033W, YHL030W, YDL140C, YPR031W, YDR421W, YLR106C, YLR256W, YBR245C, YBL004W, YGL163C, YPL125W, YDR489W, YHR099W, YKL112W, YFL049W, YDR051C, YPR018W, YDR457W, YJL187C, YGL131C	80	1723	5914	1.2442397	1	0.727891	80.4018

Annotation Cluster 6	Enrichment Score: 1.463												
Category	Term	Count	%	PValue	Genes	List Total	Pop Hits	Pop Total	Fold Enrichment	Bonferroni	Benjamini	FDR
INTERPRO	IPR011011:Zinc finger, FYVE/PHD-type	4	5	0.002949	YPR031W, YMR176W, YFR019W, YGL131C	72	21	5053	13.3677249	0.426064	0.168962	3.59792
UP_SEQ_FEATURE	zinc finger region:PHD-type	3	3.8	0.009086	YPR031W, YMR176W, YGL131C	80	11	5872	20.0181818	0.900675	0.684841	11.2017
INTERPRO	IPR019787:Zinc finger, PHD-finger	3	3.8	0.011731	YPR031W, YMR176W, YGL131C	72	12	5053	17.5451389	0.891225	0.358338	13.6193
INTERPRO	IPR013083:Zinc finger, RING/FYVE/PHD-type	5	6.3	0.013402	YPR031W, YMR176W, YKL028W, YFR019W, YGL131C	72	66	5053	5.31670875	0.920861	0.344762	15.4136
INTERPRO	IPR001965:Zinc finger, PHD-type	3	3.8	0.018163	YPR031W, YMR176W, YGL131C	72	15	5053	14.0361111	0.96813	0.388781	20.3415
SMART	SM00249:PHD	3	3.8	0.022177	YPR031W, YMR176W, YGL131C	29	15	1776	12.2482759	0.489728	0.489728	17.3904
UP_KEYWORDS	Zinc	9	11	0.287902	YPR031W, YDL140C, YMR176W, YDR421W, YLR256W, YKL028W, YFR019W, YGL131C, YKL103C	80	469	5914	1.41860341	1	0.840642	97.9591
GOTERM_MF_DIRECT	GO:0008270~zinc ion binding	6	7.5	0.293613	YPR031W, YMR176W, YDR421W, YLR256W, YGL131C, YKL103C	66	271	4873	1.63468635	1	0.984565	98.2732
UP_KEYWORDS	Zinc-finger	5	6.3	0.475334	YPR031W, YMR176W, YKL028W, YFR019W, YGL131C	80	265	5914	1.39481132	1	0.922577	99.9384

Annotation Cluster 7	Enrichment Score: 1.253												
Category	Term	Count	%	PValue	Genes	List Total	Pop Hits	Pop Total	Fold Enrichment	Bonferroni	Benjamini	FDR
UP_KEYWORDS	Magnesium	9	11	0.007804	YDR502C, YDL140C, YOL049W, YDL164C, YAL026C, YGR240C, YMR162C, YJL187C, YLR180W	80	216	5914	3.08020833	0.606355	0.170109	8.58847
GOTERM_MF_DIRECT	GO:0046872~metal ion binding	17	21	0.039292	YPR031W, YDL140C, YDR421W, YLR256W, YGR240C, YMR162C, YLR180W, YDR502C, YMR176W, YOL049W, YDL164C, YAL026C, YJL187C, YKL028W, YGL131C, YFR019W, YKL103C	66	763	4873	1.6450415	0.994964	0.585991	37.3794
UP_KEYWORDS	Metal-binding	15	19	0.110073	YDL140C, YPR031W, YDR421W, YLR256W, YGR240C, YLR180W, YDR502C, YMR176W, YOL049W, YDL164C, YJL187C, YKL028W, YGL131C, YFR019W, YKL103C	80	739	5914	1.50050744	0.999999	0.716791	73.7274
UP_KEYWORDS	Zinc	9	11	0.287902	YPR031W, YDL140C, YMR176W, YDR421W, YLR256W, YKL028W, YFR019W, YGL131C, YKL103C	80	469	5914	1.41860341	1	0.840642	97.9591

Annotation Cluster 8	Enrichment Score: 1.134

Category	Term	Count	%	PValue	Genes	List Total	Pop Hits	Pop Total	Fold Enrichment	Bonferroni	Benjamini	FDR
UP_KEYWORDS	Magnesium	9	11	0.007804	YDR502C, YDL140C, YOL049W, YDL164C, YAL026C, YGR240C, YMR162C, YJL187C, YLR180W	80	216	5914	3.08020833	0.606355	0.170109	8.58847
UP_KEYWORDS	Isopeptide bond	9	11	0.199882	YDR502C, YDL140C, YKL112W, YPL106C, YGR240C, YJL012C, YJL187C, YBR073W, YLR180W	80	422	5914	1.57659953	1	0.77105	92.2383
UP_KEYWORDS	Ubl conjugation	9	11	0.256687	YDR502C, YDL140C, YKL112W, YPL106C, YGR240C, YJL012C, YJL187C, YBR073W, YLR180W	80	453	5914	1.46870861	1	0.813803	96.6628
Annotation Cluster 9	Enrichment Score: 0.808											
Category	Term	Count	%	PValue	Genes	List Total	Pop Hits	Pop Total	Fold Enrichment	Bonferroni	Benjamini	FDR
GOTERM_CC_DIRECT	GO:0005739~mitochondrion	21	26	0.105166	YPR166C, YDL140C, YLR106C, YLR256W, YLR382C, YBR203W, YGR240C, YHR059W, YPL137C, YLR089C, YOR176W, YOL129W, YJL109C, YOL008W, YDL164C, YDR125C, YER077C, YKL028W, YFR019W, YHR116W, YLL040C	76	1180	5850	1.36987065	0.999997	0.999997	71.739
UP_KEYWORDS	Transit peptide	9	11	0.119136	YOL008W, YPR166C, YDL164C, YLR382C, YHR059W, YER077C, YLR089C, YOR176W, YHR116W	80	370	5914	1.79817568	1	0.715762	76.6358
UP_SEQ_FEATURE	transit peptide:Mitochondrion	8	10	0.144155	YOL008W, YPR166C, YDL164C, YLR382C, YHR059W, YLR089C, YOR176W, YHR116W	80	324	5872	1.81234568	1	0.992722	86.8145
UP_KEYWORDS	Mitochondrion	13	16	0.324248	YPR166C, YLR382C, YGR240C, YHR059W, YOR176W, YLR089C, YOL129W, YOL008W, YJL109C, YDR125C, YDL164C, YER077C, YHR116W	80	767	5914	1.2529661	1	0.868377	98.8805
Annotation Cluster 10	Enrichment Score: 0.767											
Category	Term	Count	%	PValue	Genes	List Total	Pop Hits	Pop Total	Fold Enrichment	Bonferroni	Benjamini	FDR
GOTERM_BP_DIRECT	GO:0016569~covalent chromatin modification	4	5	0.15529	YHR099W, YFL049W, YML127W, YBR245C	74	103	5514	2.89372868	1	0.985287	88.8349
UP_KEYWORDS	Chromatin regulator	4	5	0.158339	YHR099W, YFL049W, YML127W, YBR245C	80	103	5914	2.87087379	1	0.745265	86.1348
GOTERM_BP_DIRECT	GO:0045944~positive regulation of transcription from RNA polymerase II promoter	5	6.3	0.203552	YHR099W, YDR421W, YFL049W, YKL112W, YBR245C	74	176	5514	2.11686118	1	0.987434	94.8007
Annotation Cluster 11	Enrichment Score: 0.739											
Category	Term	Count	%	PValue	Genes	List Total	Pop Hits	Pop Total	Fold Enrichment	Bonferroni	Benjamini	FDR
GOTERM_BP_DIRECT	GO:0000480~endonucleolytic cleavage in 5'-ETS of tricistronic rRNA transcript (SSU-rRNA, 5,8S rRNA, LSU-rRNA)	3	3.8	0.062822	YJL109C, YDR457W, YBL004W	74	31	5514	7.21098518	1	0.960997	56.9533

GOTERM_BP_DIRECT	GO:0000472~endonucleolytic cleavage to generate mature 5'-end of SSU-rRNA from (SSU-rRNA, 5,8S rRNA, LSU-rRNA)	3	3.8	0.066451	YJL109C, YDR457W, YBL004W	74	32	5514	6.98564189	1	0.943022	59.0692
GOTERM_BP_DIRECT	GO:0000447~endonucleolytic cleavage in ITS1 to separate SSU-rRNA from 5,8S rRNA and LSU-rRNA from tricistronic rRNA transcript (SSU-rRNA, 5,8S rRNA, LSU-rRNA)	3	3.8	0.110393	YJL109C, YDR457W, YBL004W	74	43	5514	5.19861722	1	0.974151	78.1206
GOTERM_BP_DIRECT	GO:0006364~rRNA processing	5	6.3	0.287272	YJL109C, YLR106C, YML127W, YDR457W, YBL004W	74	205	5514	1.81740277	1	0.996462	98.7716
UP_KEYWORDS	rRNA processing	4	5	0.408587	YJL109C, YML127W, YDR457W, YBL004W	80	173	5914	1.70924855	1	0.892715	99.7571
GOTERM_CC_DIRECT	GO:0005730~nucleolus	4	5	0.682676	YJL109C, YNL248C, YDR457W, YBL004W	76	271	5850	1.13614294	1	0.999957	99.9998
Annotation Cluster 12	Enrichment Score: 0.671											
Category	Term	Count	%	PValue	Genes	List Total	Pop Hits	Pop Total	Fold Enrichment	Bonferroni	Benjamini	FDR
GOTERM_MF_DIRECT	GO:0016740~transferase activity	14	18	0.121157	YHR137W, YDL140C, YGR240C, YLR089C, YLR180W, YDR502C, YDR208W, YNR019W, YNL248C, YDR125C, YBR038W, YDR457W, YJL187C, YFR019W	66	690	4873	1.49806763	1	0.881277	77.8669
UP_KEYWORDS	Transferase	13	16	0.193199	YHR137W, YDR502C, YDR208W, YNR019W, YDL140C, YNL248C, YDR125C, YBR038W, YGR240C, YJL187C, YLR089C, YFR019W, YLR180W	80	681	5914	1.41119677	1	0.777482	91.4618
KEGG_PATHWAY	sce01100:Metabolic pathways	12	15	0.413761	YHR137W, YDR502C, YDR208W, YDL140C, YNL248C, YOL049W, YPR145W, YGR240C, YGR124W, YLR089C, YOR176W, YLR180W	33	685	2194	1.16469808	1	0.9909	99.3237
Annotation Cluster 13	Enrichment Score: 0.605											
Category	Term	Count	%	PValue	Genes	List Total	Pop Hits	Pop Total	Fold Enrichment	Bonferroni	Benjamini	FDR
INTERPRO	IPR000330:SNF2-related	3	3.8	0.023105	YBR245C, YGL163C, YBR073W	72	17	5053	12.3848039	0.987658	0.42267	25.1756
UP_SEQ_FEATURE	domain:Helicase C-terminal	3	3.8	0.277592	YBR245C, YGL163C, YBR073W	80	77	5872	2.85974026	1	0.999733	98.5479
UP_SEQ_FEATURE	domain:Helicase ATP-binding	3	3.8	0.287615	YBR245C, YGL163C, YBR073W	80	79	5872	2.78734177	1	0.99959	98.7893
INTERPRO	IPR014001:Helicase, superfamily 1/2, ATP-binding domain	3	3.8	0.294744	YBR245C, YGL163C, YBR073W	72	77	5053	2.73430736	1	0.995792	98.6864
INTERPRO	IPR001650:Helicase, C-terminal	3	3.8	0.299973	YBR245C, YGL163C, YBR073W	72	78	5053	2.69925214	1	0.994244	98.8023
INTERPRO	IPR027417:P-loop containing nucleoside triphosphate hydrolase	7	8.8	0.315313	YOR259C, YGL099W, YMR109W, YLR106C, YBR245C, YGL163C, YBR073W	72	329	5053	1.49320331	1	0.993821	99.0901

SMART	SM00487:DEXDc	3	3.8	0.34459	YBR245C, YGL163C, YBR073W	29	77	1776	2.38602777	0.999997	0.985374	97.2649
SMART	SM00490:HELICc	3	3.8	0.34459	YBR245C, YGL163C, YBR073W	29	77	1776	2.38602777	0.999997	0.985374	97.2649
UP_KEYWORDS	Helicase	3	3.8	0.378007	YBR245C, YGL163C, YBR073W	80	98	5914	2.2630102	1	0.886192	99.5671
GOTERM_MF_DIRECT	GO:0004386~helicase activity	3	3.8	0.387091	YBR245C, YGL163C, YBR073W	66	100	4873	2.215	1	0.990104	99.6709

Annotation Cluster 14	Enrichment Score: 0.602											
Category	Term	Count	%	PValue	Genes	List Total	Pop Hits	Pop Total	Fold Enrichment	Bonferroni	Benjamini	FDR
GOTERM_BP_DIRECT	GO:0015031~protein transport	9	11	0.141941	YOL129W, YGL099W, YGL137W, YPR105C, YBL037W, YKL176C, YPL125W, YLL040C, YKL103C	74	390	5514	1.71954262	1	0.985768	86.3125
UP_KEYWORDS	Protein transport	9	11	0.146238	YOL129W, YGL099W, YGL137W, YPR105C, YBL037W, YKL176C, YPL125W, YLL040C, YKL103C	80	389	5914	1.71034704	1	0.739167	83.67
GOTERM_BP_DIRECT	GO:0006810~transport	13	16	0.398155	YOL129W, YGL084C, YGL099W, YGL137W, YPR105C, YDR051C, YBL037W, YDR457W, YKL176C, YPL125W, YLL040C, YBR235W, YKL103C	74	818	5514	1.18420009	1	0.999134	99.8634
UP_KEYWORDS	Transport	13	16	0.471486	YOL129W, YGL084C, YGL099W, YGL137W, YPR105C, YDR051C, YBL037W, YDR457W, YKL176C, YPL125W, YLL040C, YBR235W, YKL103C	80	853	5914	1.12664127	1	0.926957	99.9331

Annotation Cluster 15	Enrichment Score: 0.583											
Category	Term	Count	%	PValue	Genes	List Total	Pop Hits	Pop Total	Fold Enrichment	Bonferroni	Benjamini	FDR
GOTERM_CC_DIRECT	GO:0005774~vacuolar membrane	5	6.3	0.155824	YOL129W, YJL012C, YKL176C, YFR019W, YBR235W	76	163	5850	2.36115596	1	0.999936	85.4338
UP_KEYWORDS	Vacuole	5	6.3	0.213703	YOL129W, YJL012C, YFR019W, YBR235W, YKL103C	80	178	5914	2.07654494	1	0.778158	93.6434
GOTERM_CC_DIRECT	GO:0005773~vacuole	5	6.3	0.279551	YOL129W, YJL012C, YFR019W, YBR235W, YKL103C	76	209	5850	1.8414757	1	0.999913	97.598
GOTERM_CC_DIRECT	GO:0000329~fungal-type vacuole membrane	4	5	0.49893	YOL129W, YJL012C, YFR019W, YBR235W	76	207	5850	1.48741419	1	0.999987	99.9614

Annotation Cluster 16	Enrichment Score: 0.464											
Category	Term	Count	%	PValue	Genes	List Total	Pop Hits	Pop Total	Fold Enrichment	Bonferroni	Benjamini	FDR
GOTERM_MF_DIRECT	GO:0016787~hydrolase activity	12	15	0.260898	YOR259C, YGL099W, YMR109W, YDR125C, YDR051C, YOR126C, YAL026C, YMR162C, YBR245C, YGL163C, YBR073W, YKL103C	66	661	4873	1.34039334	1	0.981512	97.0702
INTERPRO	IPR027417:P-loop containing nucleoside triphosphate hydrolase	7	8.8	0.315313	YOR259C, YGL099W, YMR109W, YLR106C, YBR245C, YGL163C, YBR073W	72	329	5053	1.49320331	1	0.993821	99.0901
UP_KEYWORDS	Hydrolase	10	13	0.49423	YGL099W, YMR109W, YDR051C, YOR126C, YAL026C, YMR162C, YBR245C, YGL163C, YBR073W, YKL103C	80	643	5914	1.14968896	1	0.920736	99.9596

Annotation Cluster 17	Enrichment Score: 0.436											
Category	Term	Count	%	PValue	Genes	List Total	Pop Hits	Pop Total	Fold Enrichment	Bonferroni	Benjamini	FDR

GOTERM_BP_DIRECT	GO:0006281~DNA repair	5	6.3	0.248902	YHR099W, YDL164C, YKL112W, YGL163C, YBR073W	74	192	5514	1.94045608	1	0.99397	97.5724
UP_KEYWORDS	DNA repair	4	5	0.342719	YDL164C, YKL112W, YGL163C, YBR073W	80	155	5914	1.90774194	1	0.875161	99.1851
UP_KEYWORDS	DNA damage	4	5	0.408587	YDL164C, YKL112W, YGL163C, YBR073W	80	173	5914	1.70924855	1	0.892715	99.7571
GOTERM_BP_DIRECT	GO:0006974~cellular response to DNA damage stimulus	4	5	0.517535	YDL164C, YKL112W, YGL163C, YBR073W	74	206	5514	1.44686434	1	0.999829	99.9923

Annotation Cluster 18	Enrichment Score: 0.368											
Category	Term	Count	%	PValue	Genes	List Total	Pop Hits	Pop Total	Fold Enrichment	Bonferroni	Benjamini	FDR
GOTERM_MF_DIRECT	GO:0016301~kinase activity	5	6.3	0.294762	YDR208W, YHR099W, YGR240C, YJL187C, YFR019W	66	206	4873	1.7920712	1	0.978537	98.3057
UP_KEYWORDS	Kinase	4	5	0.516744	YDR208W, YGR240C, YJL187C, YFR019W	80	204	5914	1.4495098	1	0.927369	99.976
GOTERM_BP_DIRECT	GO:0016310~phosphorylation	4	5	0.517535	YDR208W, YGR240C, YJL187C, YFR019W	74	206	5514	1.44686434	1	0.999829	99.9923

Annotation Cluster 19	Enrichment Score: 0.268											
Category	Term	Count	%	PValue	Genes	List Total	Pop Hits	Pop Total	Fold Enrichment	Bonferroni	Benjamini	FDR
UP_KEYWORDS	Golgi apparatus	4	5	0.486307	YGL137W, YPR105C, YAL026C, YLL040C	80	195	5914	1.51641026	1	0.922469	99.9517
GOTERM_CC_DIRECT	GO:0000139~Golgi membrane	3	3.8	0.532186	YGL137W, YPR105C, YAL026C	76	138	5850	1.67334096	1	0.999934	99.9823
GOTERM_CC_DIRECT	GO:0005794~Golgi apparatus	4	5	0.608779	YGL137W, YPR105C, YAL026C, YLL040C	76	243	5850	1.26705653	1	0.99994	99.9977

Annotation Cluster 20	Enrichment Score: 0.208											
Category	Term	Count	%	PValue	Genes	List Total	Pop Hits	Pop Total	Fold Enrichment	Bonferroni	Benjamini	FDR
GOTERM_BP_DIRECT	GO:0042254~ribosome biogenesis	4	5	0.500872	YJL109C, YGL099W, YNL248C, YBL004W	74	201	5514	1.48285599	1	0.999831	99.988
GOTERM_CC_DIRECT	GO:0005730~nucleolus	4	5	0.682676	YJL109C, YNL248C, YDR457W, YBL004W	76	271	5850	1.13614294	1	0.999957	99.9998
UP_KEYWORDS	Ribosome biogenesis	3	3.8	0.696314	YJL109C, YNL248C, YBL004W	80	179	5914	1.23896648	1	0.980541	99.9999

Annotation Cluster 21	Enrichment Score: 0.198											
Category	Term	Count	%	PValue	Genes	List Total	Pop Hits	Pop Total	Fold Enrichment	Bonferroni	Benjamini	FDR
GOTERM_BP_DIRECT	GO:0007067~mitotic nuclear division	3	3.8	0.521507	YPR119W, YDR457W, YJL187C	74	131	5514	1.70641634	1	0.99977	99.9931
GOTERM_BP_DIRECT	GO:0007049~cell cycle	5	6.3	0.594054	YPR119W, YDL164C, YJL090C, YDR457W, YJL187C	74	310	5514	1.20183086	1	0.999945	99.9992
UP_KEYWORDS	Cell cycle	5	6.3	0.595591	YPR119W, YDL164C, YJL090C, YDR457W, YJL187C	80	308	5914	1.20008117	1	0.953954	99.9969
GOTERM_BP_DIRECT	GO:0051301~cell division	3	3.8	0.741809	YPR119W, YDL164C, YJL187C	74	197	5514	1.13472356	1	0.999999	100
UP_KEYWORDS	Cell division	3	3.8	0.743354	YPR119W, YDL164C, YJL187C	80	196	5914	1.1315051	1	0.987402	100

Annotation Cluster 22	Enrichment Score: 0.070											
Category	Term	Count	%	PValue	Genes	List Total	Pop Hits	Pop Total	Fold Enrichment	Bonferroni	Benjamini	FDR
GOTERM_CC_DIRECT	GO:0005783~endoplasmic reticulum	8	10	0.725941	YGL084C, YNR019W, YPR105C, YAL026C, YJL012C, YNL080C, YMR162C, YPL137C	76	639	5850	0.9636768	1	0.999974	100
UP_KEYWORDS	Endoplasmic reticulum	4	5	0.892331	YNR019W, YPR105C, YNL080C, YPL137C	80	381	5914	0.77611549	1	0.998887	100

GOTERM_CC_DIRECT	GO:0005789~endoplasmic reticulum membrane	3	3.8	0.953739	YNR019W, YPR105C, YNL080C	76	366	5850	0.63093184	1	1	100

Annotation Cluster 23	Enrichment Score: 0.035											
Category	Term	Count	%	PValue	Genes	List Total	Pop Hits	Pop Total	Fold Enrichment	Bonferroni	Benjamini	FDR
GOTERM_CC_DIRECT	GO:0005783~endoplasmic reticulum	8	10	0.725941	YGL084C, YNR019W, YPR105C, YAL026C, YJL012C, YNL080C, YMR162C, YPL137C	76	639	5850	0.9636768	1	0.999974	100
UP_SEQ_FEATURE	topological domain:Extracellular	4	5	0.824774	YGL084C, YBR038W, YMR162C, YBR235W	80	327	5872	0.89785933	1	1	100
UP_SEQ_FEATURE	topological domain:Cytoplasmic	7	8.8	0.917682	YGL084C, YBR038W, YAL026C, YJL012C, YNL080C, YMR162C, YBR235W	80	693	5872	0.74141414	1	1	100
GOTERM_CC_DIRECT	GO:0016020~membrane	17	21	0.960227	YGL084C, YPR105C, YGR240C, YNL080C, YJL012C, YMR162C, YOR176W, YOL129W, YOL008W, YBR108W, YNR019W, YGL137W, YBR038W, YAL026C, YBL037W, YFR019W, YBR235W	76	1734	5850	0.75464396	1	1	100
GOTERM_CC_DIRECT	GO:0016021~integral component of membrane	12	15	0.9694	YOL129W, YGL084C, YJL109C, YNR019W, YBR038W, YAL026C, YJL012C, YNL080C, YMR162C, YDR457W, YPL125W, YBR235W	76	1325	5850	0.69712016	1	1	100
UP_KEYWORDS	Membrane	17	21	0.970718	YGL084C, YPR105C, YGR240C, YNL080C, YJL012C, YMR162C, YOR176W, YOL129W, YOL008W, YBR108W, YNR019W, YGL137W, YBR038W, YAL026C, YBL037W, YFR019W, YBR235W	80	1712	5914	0.73406834	1	0.999943	100
UP_SEQ_FEATURE	transmembrane region	9	11	0.993568	YOL129W, YGL084C, YNR019W, YBR038W, YAL026C, YJL012C, YNL080C, YMR162C, YBR235W	80	1164	5872	0.56752577	1	1	100
UP_KEYWORDS	Transmembrane helix	9	11	0.993795	YOL129W, YGL084C, YNR019W, YBR038W, YAL026C, YJL012C, YNL080C, YMR162C, YBR235W	80	1176	5914	0.56575255	1	0.999999	100
UP_KEYWORDS	Transmembrane	9	11	0.994153	YOL129W, YGL084C, YNR019W, YBR038W, YAL026C, YJL012C, YNL080C, YMR162C, YBR235W	80	1182	5914	0.56288071	1	0.999999	100
