# Supplementary material for: The role of PCNA as a scaffold protein in cellular signaling is functionally conserved between yeast and humans
Source: FEBS Open Bio. 2018 May 31;8(7):1135–45. doi: 10.1002/2211-5463.12442 (PMC6026702; doi:10.1002/2211-5463.12442)
Supplement: Supplementary file 6 — Table S3. Enrichment for functional categories in APIM‐containing yeast proteins. Multiple FunCat functional categories (FunCat IDs) are considered for each protein, as shown in Table S1. Functional categories found in the PPI network (Table S3) are highlighted in bold. [file FEB4-8-1135-s006.pdf]

The role of PCNA as a scaffold protein in cellular signaling is functionally conserved between yeast and humans

Camilla Olaisen[1], Hans Fredrik N. Kvitvang[2], Sungmin Lee[2], Eivind Almaas[2], Per Bruheim[2], Finn Drabløs[1], and Marit Otterlei[1*].

[1]Department of Clinical and Molecular Medicine, Faculty of Medicine and Health Sciences, Norwegian University of Science and Technology (NTNU), Trondheim, Norway.

[2]Department of Biotechnology and Food Science, Faculty of Natural Sciences, Norwegian University of Science and Technology (NTNU), Trondheim, Norway.

Supplementary Table S3. Enrichment for functional categories in APIM-containing yeast proteins. Multiple FunCat functional categories (FunCat IDs) are considered for each protein, as shown in Supplementary Table S1. Functional categories found in the PPI network (Supplementary Table S3) are highlighted in bold.

FunCat ID	Function	Number of associated APIM-containing proteins	p-value
**1**	Metabolism	21	0.72043
**2**	Energy	5	0.65813
**10**	Cell cycle and DNA processing	18	0.24729
**11**	Transcription	18	0.30608
**12**	Protein synthesis	10	0.20258
**14**	Protein fate (folding, modification, destination)	17	<1E-5
**16**	Protein with binding function or cofactor requirement (structural or catalytic)	18	<1E-5
18	Regulation of metabolism and protein function	2	<1E-5
**20**	Cellular transport, transport facilitation and transport routes	14	<1E-5
**30**	Cellular communication/signal transduction mechanism	2	<1E-5
32	Cell rescue, defense and virulence	6	<1E-5
34	Interaction with the environment	6	<1E-5
40	Cell fate	2	<1E-5
41	Development (systemic)	3	<1E-5
**42**	Biogenesis of cellular components	14	<1E-5
43	Cell type differentiation	8	<1E-5
