# Supplementary material for: The role of PCNA as a scaffold protein in cellular signaling is functionally conserved between yeast and humans
Source: FEBS Open Bio. 2018 May 31;8(7):1135–45. doi: 10.1002/2211-5463.12442 (PMC6026702; doi:10.1002/2211-5463.12442)
Supplement: Supplementary file 7 — Table S4. Enrichment for APIM‐containing yeast proteins in PPI clusters. Cluster ID 2 and 3 are significantly enriched (highlighted in bold). The third column shows FunCat functional categories (FunCat IDs) of APIM‐containing yeast proteins in each PPI cluster. The star sign means enriched for that category at 5% (*) and 1% (**) level. See Table S2 for FunCat IDs. [file FEB4-8-1135-s007.pdf]

The role of PCNA as a scaffold protein in cellular signaling is functionally conserved between yeast and humans

Camilla Olaisen[1], Hans Fredrik N. Kvitvang[2], Sungmin Lee[2], Eivind Almaas[2], Per Bruheim[2], Finn Drabløs[1], and Marit Otterlei[1]*.

[1]Department of Clinical and Molecular Medicine, Faculty of Medicine and Health Sciences, Norwegian University of Science and Technology (NTNU), Trondheim, Norway.

[2]Department of Biotechnology and Food Science, Faculty of Natural Sciences, Norwegian University of Science and Technology (NTNU), Trondheim, Norway.

PPI cluster ID	p-value	FunCat IDs
0	0.98338	1, 10, 14, 42
1	0.16594	1, 10, 11, 14, 16, 20
2	**0.03124**	1, 10, 16, 20
3	**0.00690**	1, 12*, 14*, 16**
4	0.96722	1, 2, 10, 14, 16, 20, 30, 42
5	1.00000	-
6	0.60762	1, 11, 12*, 14, 16, 42*
7	0.61792	16, 20, 42
8	0.24407	1, 10, 11*
9	0.41102	42
10	0.42907	16
11	0.62986	1, 16

Supplementary Table S4. Enrichment for APIM-containing yeast proteins in PPI clusters.

Cluster ID 2 and 3 are significantly enriched (highlighted in bold). The third column shows FunCat functional categories (FunCat IDs) of APIM-containing yeast proteins in each PPI cluster. The star sign means enriched for that category at 5% (*) and 1% (**) level. See Supplementary Table S2 for FunCat IDs.
